# A Multidimensional Diversity‐Oriented Synthesis Strategy for Structurally Diverse and Complex Macrocycles

**DOI:** 10.1002/anie.201605460

**Published:** 2016-08-03

**Authors:** Feilin Nie, Dominique L. Kunciw, David Wilcke, Jamie E. Stokes, Warren R. J. D. Galloway, Sean Bartlett, Hannah F. Sore, David R. Spring

**Affiliations:** ^1^ Department of ChemistryUniversity of CambridgeLensfield RoadCambridgeCB2 1EWUK

**Keywords:** diversity-oriented synthesis, macrocycles, molecular diversity, synthesis design, synthetic methods

## Abstract

Synthetic macrocycles are an attractive area in drug discovery. However, their use has been hindered by a lack of versatile platforms for the generation of structurally (and thus shape) diverse macrocycle libraries. Herein, we describe a new concept in library synthesis, termed multidimensional diversity‐oriented synthesis, and its application towards macrocycles. This enabled the step‐efficient generation of a library of 45 novel, structurally diverse, and highly‐functionalized macrocycles based around a broad range of scaffolds and incorporating a wide variety of biologically relevant structural motifs. The synthesis strategy exploited the diverse reactivity of aza‐ylides and imines, and featured eight different macrocyclization methods, two of which were novel. Computational analyses reveal a broad coverage of molecular shape space by the library and provides insight into how the various diversity‐generating steps of the synthesis strategy impact on molecular shape.

Macrocyclic compounds are associated with a diverse range of biological activities and they have proven therapeutic potential.^1^ However, synthetic macrocycles are widely considered to be underexplored within drug discovery. This has been attributed to concerns regarding synthetic tractability.^2^ Despite the success of early work on macrocycle library synthesis,^3^ there remains a dearth of robust synthetic platforms for the efficient generation of macrocyclic libraries with high levels of structural diversity; in particular, variation in the nature of macrocyclic ring scaffolds is often limited.2b–2c, 4 This is significant in the context of biological screening, since the range of biological activities displayed by a library correlates to its overall shape diversity, which in turn is directly related to its structural (principally scaffold) diversity.^5^, ^6^, ^7^ Diversity‐oriented synthesis (DOS) targets structurally (including scaffold) diverse molecule collections.^5^, ^8^ Several DOS‐type strategies specifically aimed at macrocycles have emerged, including two‐directional synthesis,2c ring‐expansion methods^9^ and domain shuffling.^10^ Many macrocycle DOS campaigns are based around the three‐phase build/couple/pair (B/C/P) strategy.^7^, ^11^ Recently, we reported the development of multidimensional coupling, which involves the use of a diverse set of branching reactions on a pluripotent functional group in the couple phase and leads to a greater structural (including scaffold) diversity in the final macrocycle library.^4^ We envisaged a more advanced DOS concept featuring the extensive application of branching multidimensional diversification throughout the synthesis (Scheme 1). Starting from a pluripotent functional group (the primary branching point), compound generation would be associated with the concomitant installation of functional groups that could act as pluripotent handles for further multidimensional diversification. This would enable the use of pluripotent handles in the couple phase (for further rounds of multidimensional coupling or as additional branching points), in the pair phase (for divergent cyclizations) and modification phase (for divergent scaffold modifications). It was anticipated that application of this multidimensional concept to macrocycle library synthesis would facilitate the rapid generation of high levels of structural complexity and diversity.

**Scheme 1:**
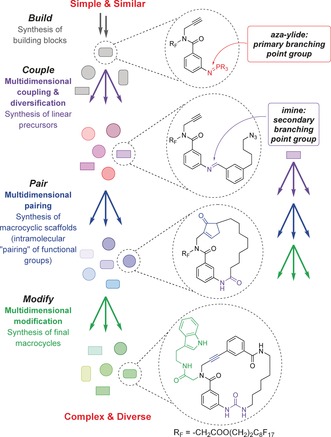
Outline of the multidimensional DOS concept applied to macrocycles. Multiple arrows represent many branching reactions, but only one representative product is shown at each stage.

Herein, we report the development a multidimensional DOS strategy towards highly functionalized macrocycles which uses fluorous‐tagged azido building blocks and exploits the pluripotent reactivity of aza‐ylides and imines (Scheme 1).

The DOS commenced with the transformation of building block **2** into **3 a**,**b**. The aza‐ylide group served as the primary branching point and **3 a**,**b** were reacted in situ in diverse aza‐Wittig‐type transformations to form **4**–**9** (Scheme 2). A further round of multidimensional coupling with **5** generated **11**–**14**, with various handles installed concomitantly to enable diverse macrocyclizations in the pair stage of the DOS. The imine functional group was identified as a suitable secondary branch point for the one‐pot generation of an extensive set of structurally diverse and bio‐relevant coupling motifs. Accordingly, **3 b** was converted to imines **15**–**16**. An Ugi multicomponent reaction of **15** afforded **17**, and Staudinger ketene cycloaddition and Strecker reactions of **16** generated **18** and **22**, respectively. Compound **16** was also subjected to various aza‐Diels–Alder reactions to access **19**–**21,** which featured a range of stereochemically and structurally diverse coupling motifs. Pure samples of the Povarov reaction products **21 a** and **21 d** were isolated and the mixture of **21 c**,**d** was used in the pair phase. Full stereochemical assignment of **21 a**–**d** was made retrospectively by NMR analysis of their macrocyclic products (see Figures S2–4 in the Supporting Information) and X‐ray crystallography of one product (**49 b**, Figure S5).^12^

**Scheme 2:**
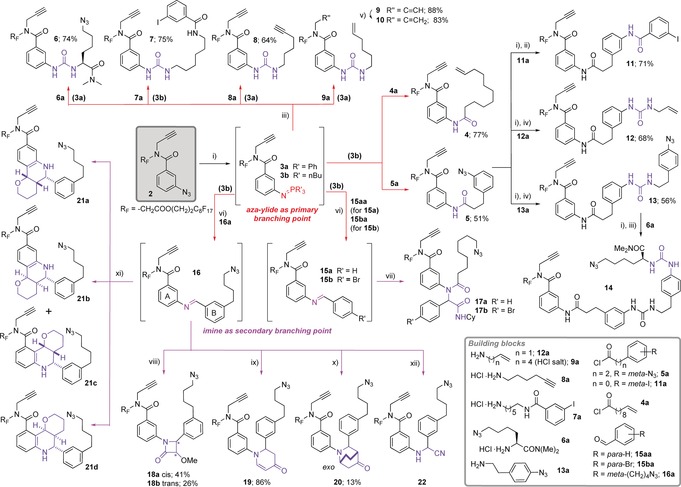
The couple phase of the DOS. Reagents and conditions: i) PBu_3_ or PPh_3_, 4 Å molecular sieves, THF, RT; ii) THF, RT, then quenched with MeOH‐H_2_O; iii) CO_2_, DIPEA, THF, RT; iv) CO_2_, THF, RT; v) Lindlar catalyst, HO(CH_2_)_2_S(CH_2_)_2_S(CH_2_)_2_OH, H_2_, MeOH, RT; vi) 40 °C, THF; vii) 8‐azidooctanoic acid, 60 °C, 1.5 h, then isocyanocyclohexane, 60 °C, MeOH; viii) methoxyacetyl chloride, NEt_3_, 0 °C then 40 °C, CH_2_Cl_2_; ix) Danishefsky's diene, Yb(OTf)_3_, 40 °C, THF; x) 2‐cyclohexen‐1‐one, Yb(OTf)_3_, 40 °C, THF; xi) 3,4‐dihydro‐2*H*‐pyran, Yb(OTf)_3_, 40 °C, THF; xii) TMS‐CN, Yb(OTf)_3_, 55 °C, THF. R_F_=CH_2_COO(CH_2_)_2_C_8_F_17_. Stereochemistry of **20** detemined by NOESY (see Figure S1; DIPEA=diisopropylethylamine, OTf=triflate, and TMS=trimethylsilyl).

In the pair stage of the DOS, the linear cyclization precursors were “folded” into diverse macrocycles with different ring architectures making use of the pluripotency of alkynes. Six known macrocyclisation methods were used initially (Scheme 3 a–f). The Pauson–Khand reaction has been used in the synthesis of medium rings (up to 11 atoms),^13^ but it has not yet been extended to macrocycles. We investigated it as a new method for macrocyclization of **4** (Scheme 3 g). After fluorous solid‐phase extraction (F‐SPE), optimized Pauson–Khand conditions (see Table S1) produced a mixture of structurally unusual macrocycles **23 a**,**b** containing a cyclopentenone motif; these can be separated by HPLC, but we used the mixture in the modify phase).

**Scheme 3:**
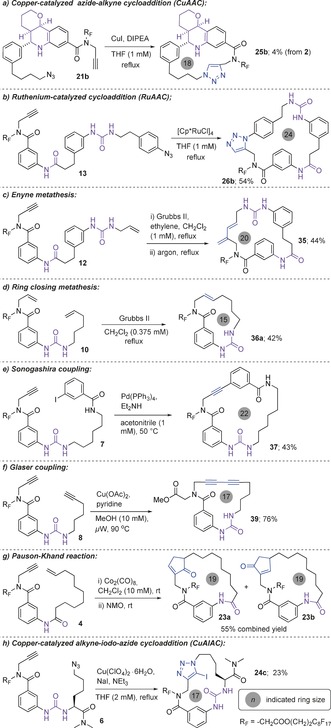
Representative examples of the macrocyclizations utilized in the pair phase. Regiochemistry of **35** determined by NOESY (see Figure S6).

Iodo‐alkynes may be cyclized into their corresponding 5‐iodotriazoles, thereby generating another handle for macrocycle functionalization. Current strategies require the synthesis of the linear iodinated analog.^14^ Here, the direct cycloaddition of azido‐alkyne **6** into 5‐iodotriazole **24 c** was achieved using an external iodide source^15^ (Scheme 3 h). The complete list of divergent macrocyclizations is presented in Scheme S1 and Tables S2 and S3 in the Supporting Information. Attempted RuAAC macrocyclization of the linear precursors featuring amine linkers resulted in decomposition of the starting material, and as such was not pursued.

The macrocyclic scaffolds could be modified divergently by use of their latent functionalities. The fluorous tag was cleaved by transesterification, ester–amide exchange (examples in Scheme 4 a), ester reduction, and ester hydrolysis (see Tables S4 and S5). Annulation of the alkyne in **54 a** to form **61** was achieved using RuAAC (Scheme 4 a). Sonogashira coupling of **44 a** yielded **62** (Scheme 4 b), dihydroxylation of **53 a** afforded **63**, and acylation generated **64 a**,**b** (Scheme 4 c).

**Scheme 4:**
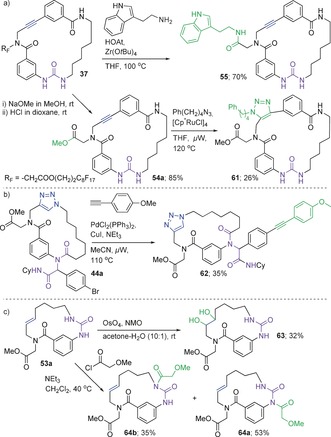
Some examples of the divergent transformations used in the modify stage of the DOS. Regiochemistry of **61** determined by NOESY (see Figure S7).

Overall, using the strategy outlined above and a limited number of building blocks, we synthesized a multidimensional DOS library of 45 novel, structurally diverse and complex macrocycles, based around a broad range of scaffolds and incorporating a wide variety of biologically relevant structural motifs (see the Supporting Information for full details). The presence of a fluorous‐tag on the common precursor **2** allowed for generic purification during the DOS by F‐SPE.^16^ Final compounds were isolated in sufficient quantities (typically milligram) for unambiguous structural determination and future screening in a variety of biological assays. Relative to our previous work,^4^ the new strategy furnished a library with a significantly greater variety of structural motifs and a larger range of macrocyclic ring sizes (15‐ to 33‐ membered rings).

Principal moments of inertia (PMI) plots are commonly used to visualize the shape diversity of compounds in “molecular shape space” spanned by the three basic extreme shape types “rod‐like”, “disk‐like” and “spherical”. Figure 1 a is a PMI plot of a series of the DOS macrocycles derived from multidimensional coupling with **16** and which differ only in the linking motif installed in the coupling phase at the secondary branching point. The broad coverage of molecular shape space by this relatively small set of macrocycles validates the use of a secondary branching point as an efficient means to generate shape diversity. Figure 1 b shows the shapes of three different groups of macrocycles (groups 1–3). The members of each group differ only in the nature of the pairing motif present. There is shape diversity present within each group, which demonstrates that the pairing step can influence final molecular shape. Figure 1 c shows the influence of three series of post‐pairing modifications upon macrocycle shape: appendage and macrocyclic annulation (series 1), scaffold modification (series 2) and appendage replacement (series 3). The members of each series differ only in the use or nature of any post‐pairing modification. The data indicates that post‐pairing modification may influence molecular shape more significantly than previously thought and may therefore represent a strategy for “tuning” the molecular shape (and thus target binding profile) of a given “parent” scaffold. A comparative PMI analysis of the DOS library with some other molecular collections (see Supporting Information, Figure S8) indicated a high level of overall molecular shape diversity, which was comparable to that of a natural product set, with more prominent spherical characteristics than a drug reference set.

**Figure 1:**
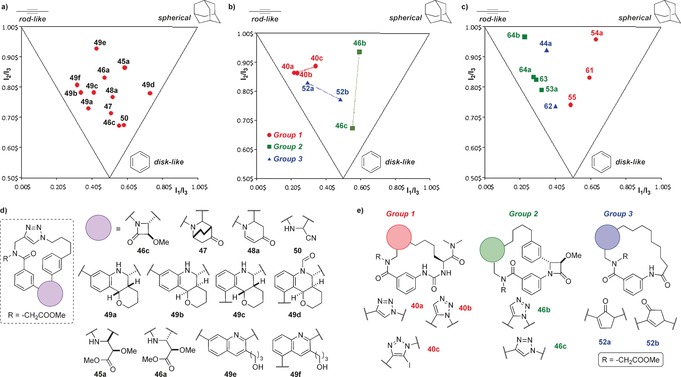
PMI plots of some selected compounds. a) Compounds which differ only in the linking motif installed at the secondary branching point. b) Three different groups of macrocycles (groups 1–3), the members of which differ only in the pairing motif present. Members of each group are linked together by a solid line. c) Three different series of compounds (Series 1–3) whose members differ only in the nature of any post‐pairing modification. d,e) Macrocycles shown in Figure 1 a and b. Macrocycles in Figure 1 c are shown in Scheme 4.

In summary, we have described a multidimensional DOS strategy for the step‐efficient generation of structurally complex and diverse macrocycles from simple and similar starting materials. Macrocyles of this sort are of significant interest from both a biological and synthetic perspective and their successful generation provides a validation of the multidimensional DOS concept. We report two new macrocyclization methodologies (the Pauson–Khand reaction and CuAIAC with an external iodine source), which, we envisage will prove valuable in a wider synthetic context. PMI analysis indicated that the library has a relatively high level of shape diversity, thus demonstrating the utility of the multidimensional approach for the compound‐efficient coverage of substantial molecular shape space. Interesting insights were gained into the impact that the various diversity‐generating elements of the DOS have upon molecular shape; this new understanding may provide a basis for the deliberate synthesis of macrocycles with tailored molecular shapes.

Multidimensional DOS integrates the B/C/P approach with branching diversification. The result is a modular and highly divergent concept for library synthesis that features systemic “product‐equals‐substrate” reactivity; functional groups generated in products become the new pluripotent handles for the next stage of the synthesis, which facilitates diversification at every branching point. This allows for the rapid generation of diversity and complexity from simple and similar starting materials. Thus, it is anticipated that the multidimensional concept will have broad strategic value in the DOS of other molecular libraries. Work is underway to expand this strategy to the synthesis of 12‐ to 14‐ membered rings and to study the substrate scope of the new macrocyclization methods reported herein.

*Dedicated to Professor Stuart L. Schreiber on the occasion of his 60th birthday*

## Supporting information

As a service to our authors and readers, this journal provides supporting information supplied by the authors. Such materials are peer reviewed and may be re‐organized for online delivery, but are not copy‐edited or typeset. Technical support issues arising from supporting information (other than missing files) should be addressed to the authors.

Supplementary
