# Supplementary material for: A Multidimensional Diversity‐Oriented Synthesis Strategy for Structurally Diverse and Complex Macrocycles
Source: Angew Chem Int Ed Engl. 2016 Aug 3;55(37):11139–43. doi: 10.1002/anie.201605460 (PMC5025730; doi:10.1002/anie.201605460)
Supplement: Supplementary file 1 — Supplementary [file ANIE-55-11139-s001.pdf]

Angewandte Chemie
Eine Zeitschrift der Gesellschaft Deutscher Chemiker

Supporting Information

A Multidimensional Diversity-Oriented Synthesis Strategy for Structurally Diverse and Complex Macrocycles

*Feilin Nie, Dominique L. Kunciw, David Wilcke, Jamie E. Stokes, Warren R. J. D. Galloway, Sean Bartlett, Hannah F. Sore, and David R. Spring**

anie_201605460_sm_miscellaneous_information.pdf

Table of contents

Supplementary Schemes, Tables and Figures **3**

In the *pair* stage **3**

General details 3

Scheme S1. Cyclization of 21a-d. 4

Table S1. Optimization of the Pauson-Khand Macrocyclization Reaction for Compound 4 5

Table S2 Macrocyclisations using CuAAC, CuAIAC and RuAAC. 6

Table S3 Other macrocyclisations and products. 10

In the *modify* stage **12**

Table S4 Transesterification. 12

Table S5 Ester-amide exchange, ester reduction and hydrolysis. 16

Determination of stereochemistry **18**

Figure S1 NOESY analysis of product 20. 18

Figure S2 NOESY analysis of product 21a. 19

Figure S3 NOESY analysis of product 25b. 20

Figure S4 NOESY analysis of product 25c. 21

Figure S5 Crystal structure of macrocycle 49b (generated from 25b). 22

Figure S6 NOESY analysis of macrocycle 35 at 120 °C (top) and 27 °C (bottom). 23

Figure S7 NOESY analysis of macrocycle 61. 24

List of final macrocycles **25**

Cheminformatic analysis – Principal Moment of Inertia (PMI) **27**

General details 27

Compound collections analysed. 27

Collection 1: Macrocycle DOS library 27

Collection 2: 40 high-profile synthetic drugs currently produced by the pharmaceutical industry 28

Collection 3: 60 randomly selected natural products 30

Collection 4: 36 macrocyclic-based compounds in clinical development as of April 2013 35

Table S6 Normalised PMI ratio (npr) values of conformers of the DOS library and three reference collections with the lowest energy (energy level = 0 kcal/mol). 40

Figure S8. Comparative PMI plot of DOS library with other molecular collections 44

Analysis of comparative PMI plot 44

Table S7 Chemical structures of conformers of the DOS library and three reference collections in SMILE. 46

Table S8 PCA of the DOS library and three reference collections. 57

Figure S9. Comparative PCA plot of DOS library with other molecular collections 61

Table S9 Standard deviation and contribution of each principal component of variance. 62

Table S10 Component loadings for PCA of DOS library with three reference sets. 63

General experimental methods **64**

General procedures **67**

Synthetic procedures and data **70**

In the *build* stage **70**

In the *couple* stage **70**

In the *pair* stage **92**

In the *modify* stage **123**

References 160

NMR Spectra 160

Supplementary Schemes, Tables and Figures

In the *pair* stage

General details

- Macrocycles derived from **21a-d** are shown in **Scheme S1**.
- The optimization of the Pauson-Khand Macrocyclization Reaction is shown in **Table S1**.
- A list of all azide alkyne cycloaddition macrocyclisations (apart from macrocycles derived from **21a-d**), are shown in **Table S2**, and other macrocyclisations are shown in **Table S3**.
- The aim of the macrocyclic library was to develop a strategy to synthesise rings ranging from 15- to 33- membered rings. This strategy could be applied to smaller 12- to 14- membered macrocycles with the use of different building blocks and chemistry. We are currently working towards the synthesis of a library of smaller natural product like macrocyclic rings.
- Another limitation of our current chemistry was the synthesis of 1,5-triazoles with an amine present in the linear precursor. Attempted RuAAC macrocyclisation coupling conditions of the linear precursors featuring amine linkers resulted in decomposition of the starting material, and as such was not pursued.
- The yield of the macrocyclisation can vary depending on the method used. In most cases each macrocyclisation method was only used in one example therefore we cannot correlate this with substrate dependence. In the case of the Huisgen cycloaddition macrocyclisation reactions the yields were relatively consistent. Further work will be conducted to investigate the effect of substrate dependence on each macrocyclisation method used.

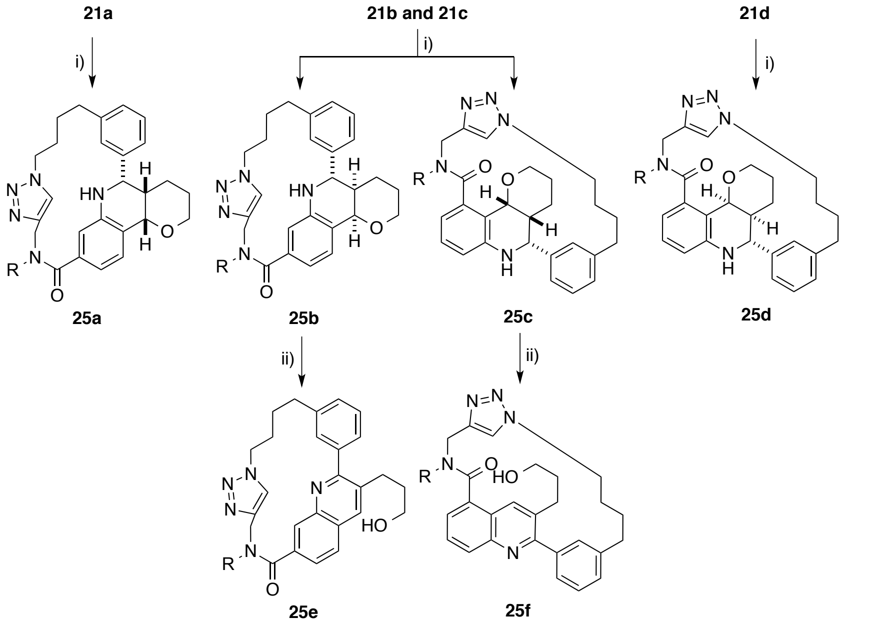

Scheme S1. Cyclization of 21a-d.

CuAAC of **21a-d** led to **25a-d**, and **25e-f** were formed during VT-NMR recording.

Reagents and conditions: i) CuI, DIPEA, reflux, THF; ii) VT-NMR, DMSO-d_6, 120 °C.

R = $-CH_2COOCH_2CH_2C_8F_{17}$

Table S1. Optimization of the Pauson-Khand Macrocyclization Reaction for Compound 4

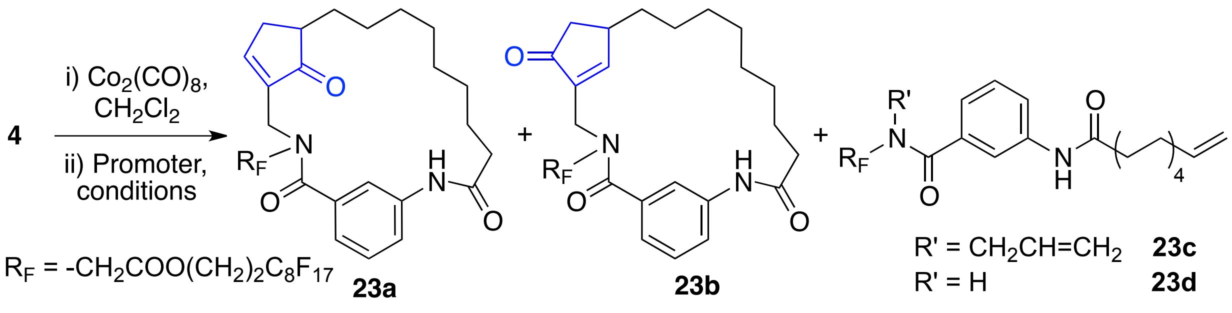

Entry	Promoter	Conds[a]	Remarks or 23a:b:c:d[b]	Yield (23a:b)
1	TMANO	A	Trace products detected	n.d.
2	NMO	A	Substantial by-products **23c-d**	n.d.
3	TMANO	B	Trace products detected	n.d.
4	NMO	B	47:33:9:4[c]	11%:7%[d]
5	NMO	C	35:25:13:27	29% (1.4:1)[e]
6	NMO	C	41:30:20:9	42% (1.4:1)[e]
**7**	**NMO**	**D**	**52:34:8:6**	**55%** (1.5:1)[e]

[a] Reactions conducted at 6.7 mM except entry 5 (10 mM) and entry 7 (8.3 mM). *Conditions: A*: promoter (6 eq), rt, o/n and then promoter (6 eq), reflux, 1 day. *B*: promoter (6 eq), rt, 1 day. *C*: promoter (6 eq), rt, o/n and then promoter (6 eq), rt, 2 hours (entry 5), 4 hours (entry 6). *D*: promoter (10 eq), rt, 3 hours. [b]The product ratio was determined by HPLC using UV absorbance peak areas at 220 nm. [c]An un-identifiable byproduct (7%) was also found. [d]Isolated yield after preparatory HPLC. [e]Combined yield of **23a-b** after fluorous solid phase extraction (F-SPE); the ratio is in the brackets.

Analysis: NMO proved a better promoter than TMANO in conjunction with $Co_2(CO)_8$ (Table S1, entries 1-4); higher reaction temperatures, longer reaction times, and higher substrate concentrations led to the formation of byproducts **23c-d** (entries 5-6). However, using 10 eq of NMO effected complete conversion in 3 hours and out-competed side-reactions (entry 7).

Table S2 Macrocyclisations using CuAAC, CuAIAC and RuAAC.

En.	Linear precursor	Cond.[a]	Time	Product(s)	Yield (%)
1		A	over night	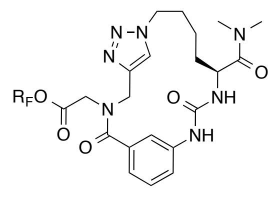 **24a**	70
2	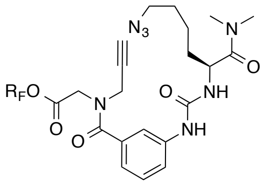 **6**	B	6 h	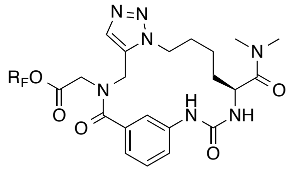 **24b**	33
3		C	3 days	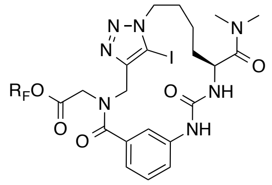 **24c**	23
				**24a**	33
4	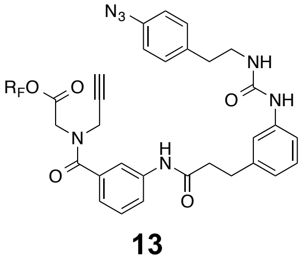 **13**	A	1.5 days	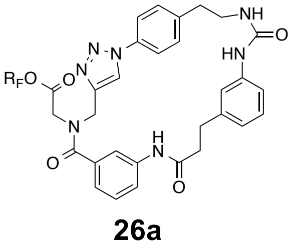 **26a**	60
5		B	5 h	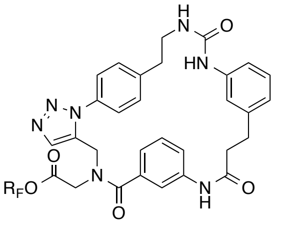 **26b**	54

6	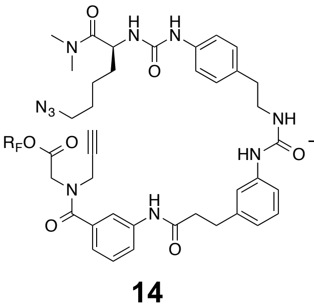 **14**	A	1 day	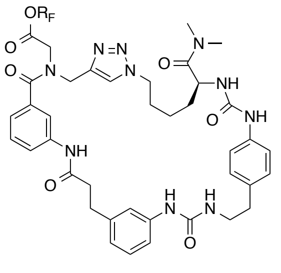 **27a**	31 over two steps from **13**
7		B	1 day	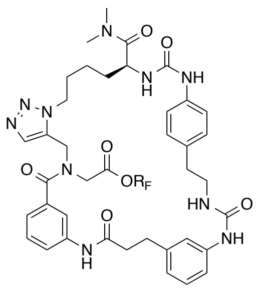 **27b**	12 over two steps from **13**
8	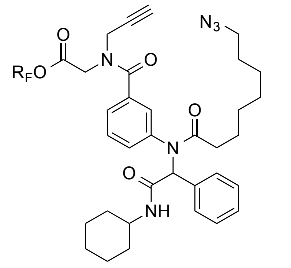 **17a**	A	over night	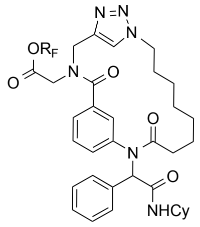 **28a**	18 over two steps from **2**
9		B[b]	2 days	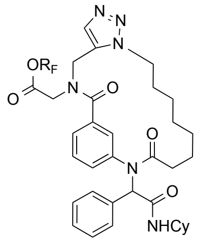 **28b**	47 over two steps from **2**
10	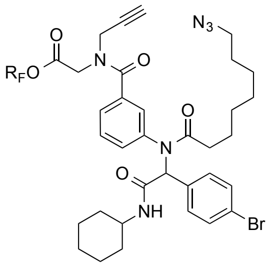 **17b**	A	over night	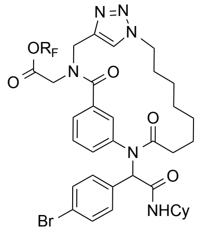 **29a**	24 over two steps from **2**
11		B	over night	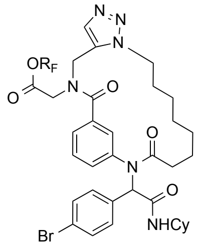 **29b**	78 over two steps from **2**

12	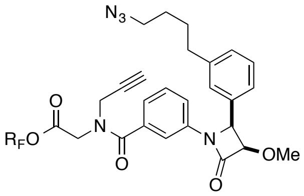 **18a**	A	1 day	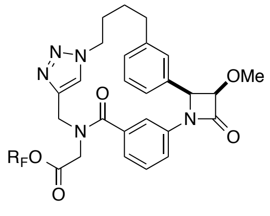 **30a**	61
13		B[c]	2 days	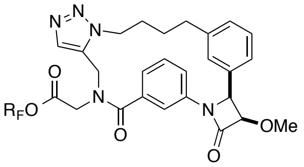 **30b**	33
				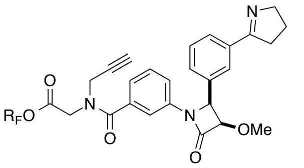 **30c**	6
14	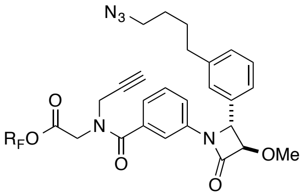 **18b**	A	1 day	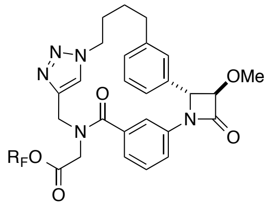 **31a**	91
15		B[c]	4 days	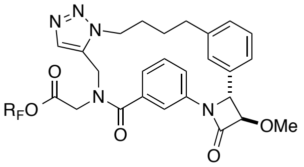 **31b**	7
				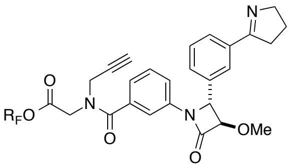 **31c**	9
16[d]	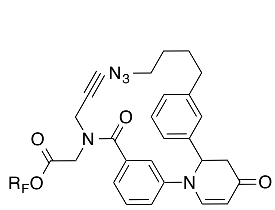 **19**	A	20 h	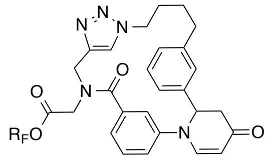 **32a**	33
17[d]		B	2 days	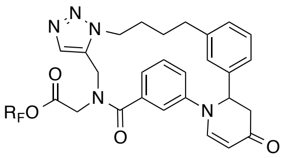 **32b**	19

18	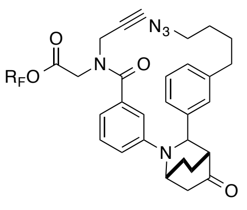 **20**	A	over night	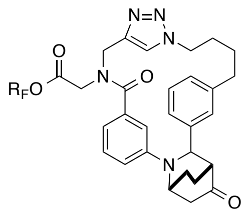 **33**	65
19	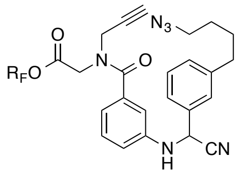 **22**	A	over night	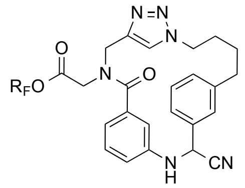 **34**	71 over two steps from **2**

[a] Condition A: reactant (1.0 eq, 1 mM), CuI (2.0 eq), DIPEA (3.0 eq), THF, reflux. Condition B: reactant (1.0 eq, 1 mM), $[Cp^{*}RuCl]_4$ (0.1 eq), THF, reflux. Condition C: reactant (1.0 eq, 5 mM), $Cu(ClO_4)_2 \cdot 6H_2O$ (6.0 eq), NaI (12 eq) and TEA (9 eq), THF, reflux.

[b] $[Cp^{*}RuCl]_4$ (0.2 eq) was added.

[c] $[Cp^{*}RuCl]_4$ (0.4 eq) was added.

[d] Structures **32a** and **32b** have been reported in our previous paper.[1]

Table S3 Other macrocyclisations and products.

En.	Linear precursor	Cond.[a]	Product(s)	Yield (%)
1	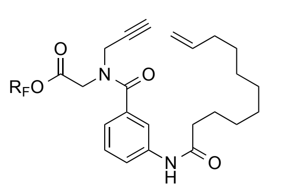 **4**	i) $Co_2(CO)_8$ (1.2 eq), CH_2Cl_2; ii) NMO (10 eq), rt, 3 h, CH_2Cl_2	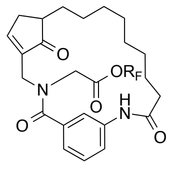 **23a**	55 in total
			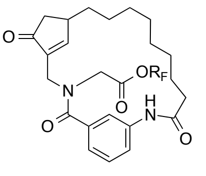 **23b**	
2	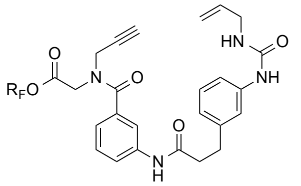 **12**	i) Grubbs II, ethylene, CH_2Cl_2, reflux, 5 h; ii) Grubbs II, Ar, reflux, overnight	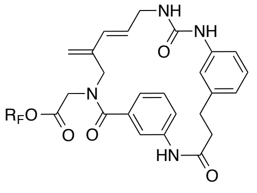 **35**	44
3	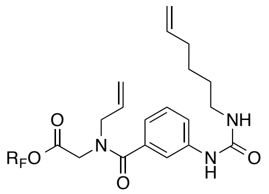 **10**	Grubbs II, CH_2Cl_2, reflux, 6 h	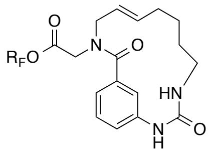 **36a**	42
			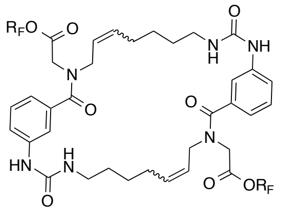 **36b**	4
4	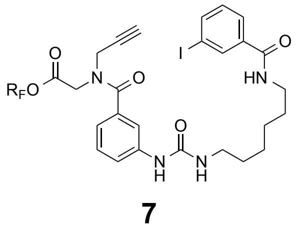 **7**	$Pd(PPh_3)_4$ (0.5 eq), $HNEt_2$ (1.5 eq), acetonitrile, 50 °C.	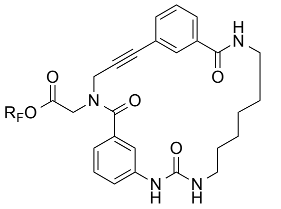 **37**	43
5	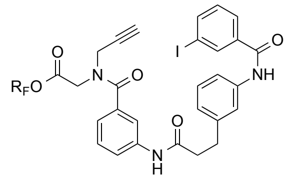 **11**		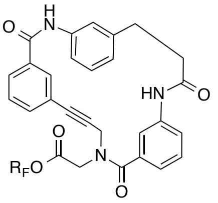 **38**	39

6	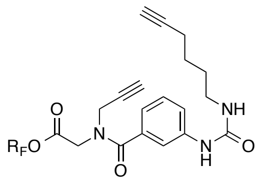 **8**	$Cu(OAc)_2$ (2 eq), pyridine (4 eq), λW 90 °C, methanol, conc. (6.7 mM)	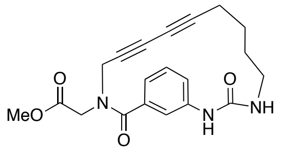 **39**	76

In the *modify* stage

Table S4 Transesterification.

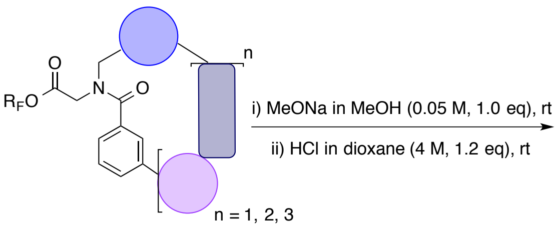

En	SM	Product	Yield (%)	En	SM	Product	Yield (%)
1	**24a**	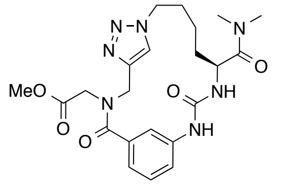 **40a**	quant.	2	**24b**	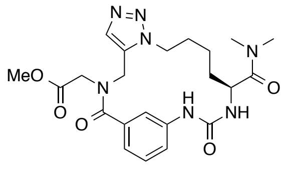 **40b**	77
3	**24c**	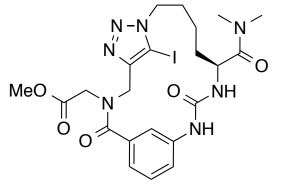 **40c**	57			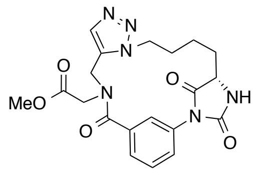 **40d**	11
4	**26a**	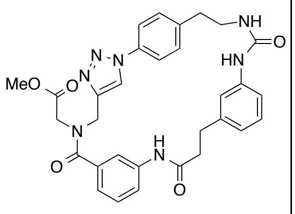 **41a**	72	5	**26b**	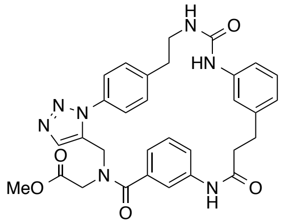 **41b**	82
6	**27a**	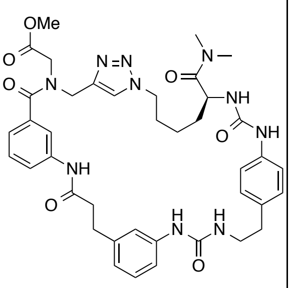 **42a**	73	7	**27b**	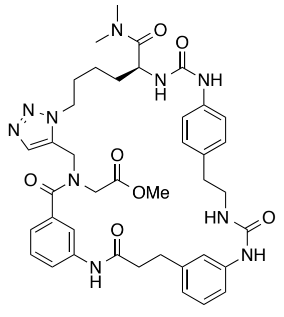 **42b**	98

8	**28a**	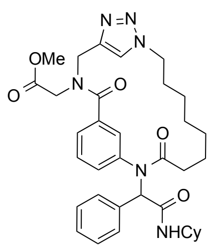<br>**43a**	65	9	**28b**	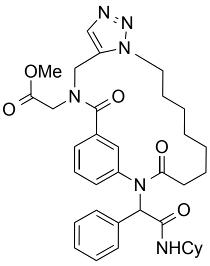<br>**43b**	53
10	**29a**	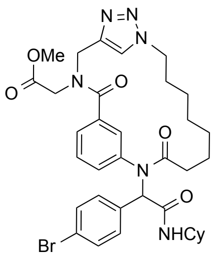<br>**44a**	58	11	**29b**	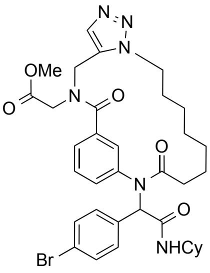<br>**44b**	6
12	**30a**	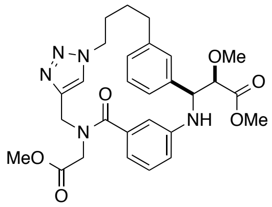<br>**45a**	quant.	14	**31a**	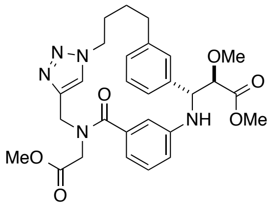<br>**46a**	55
13	**30b**	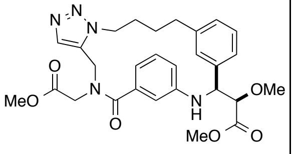<br>**45b**	78			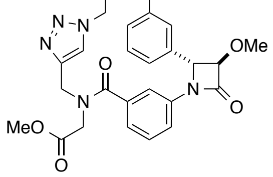<br>**46c**	4
15	**31b**	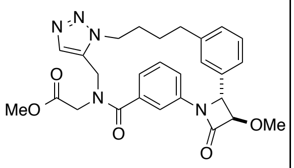<br>**46b**	50	16	**33**	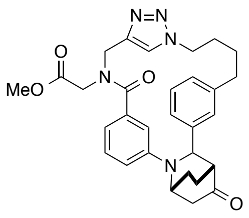<br>**47**	67
17	**32a**	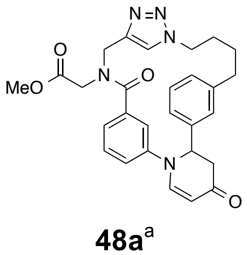<br>**48a**[a]	65	18	**32b**	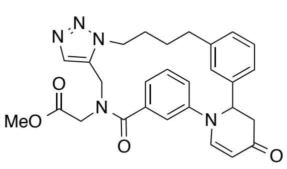<br>**48b**[a]	95

19	**25e**	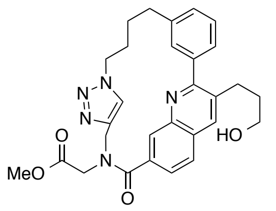 **49e**	36	20	**25f**	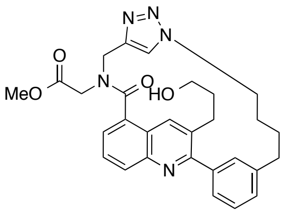 **49f**	64
21	**25a**	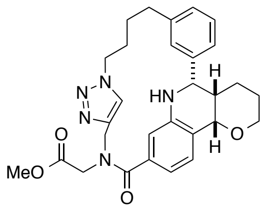 **49a**	40	22	**25b**	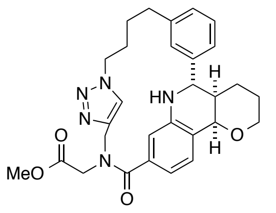 **49b**	83
		**49e**	20				
23	**25c**	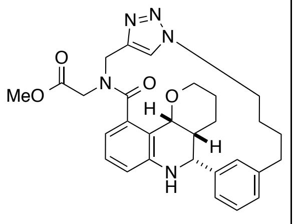 **49c**	17	24	**25d**	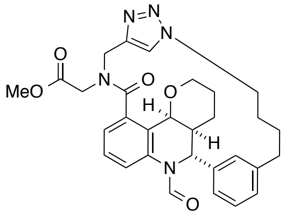 **49d**	9
		**49f**	13			**49f**	50
25	**34**	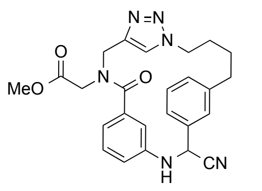 **50**	89	26	**35**	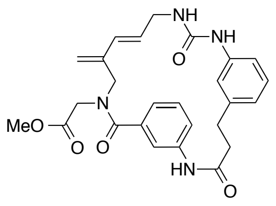 **51**	78
27	**23a**	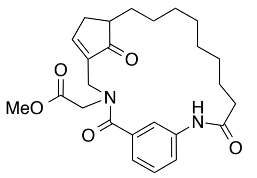 **52a**	24 over two steps[b]	28	**23b**	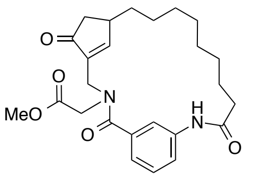 **52b**	13 over two steps[b]
31	**36a**	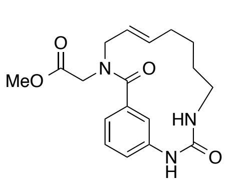 **53a**	91	32	**36b**	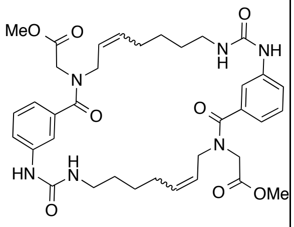 **53b**	86

29	**37**	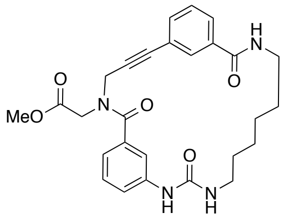 **54a**	85	30	**38**	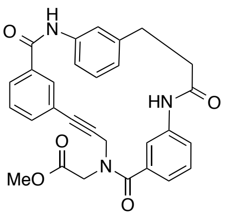 **54b**	67

[a] Structures **48a** and **48b** have been reported in our previous work.

[b] Yield was calculated from **4**.

Table S5 Ester-amide exchange, ester reduction and hydrolysis.

En.	SM	Conditions[a]	Product(s)	Yield (%)
1	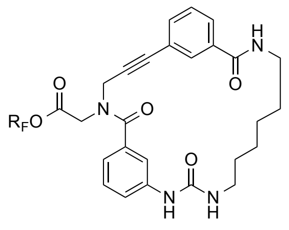 **37**	Tryptamine (3.0 eq), $Zr(OtBu)_4$ (3.0 eq), HOAt (3.0 eq), THF, 100 °C in sealed tube	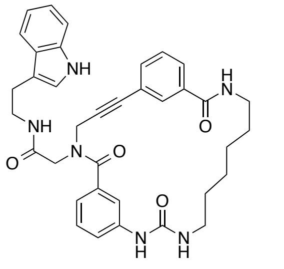 **55**	70
2	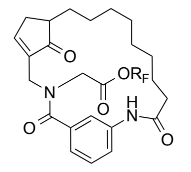 **23a**		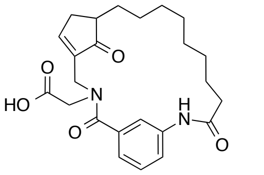 **56a**	71
3	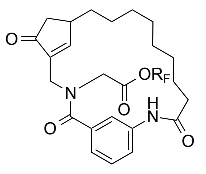 **23b**	i) LiOH (5 eq), THF/H_2O (10:1, v/v); ii) HCl in dioxane (4 M)	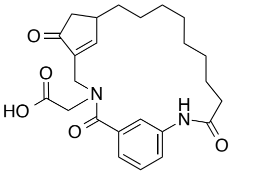 **56b**	94
4	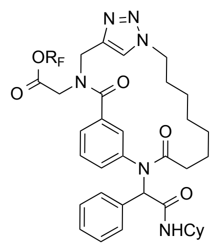 **28a**		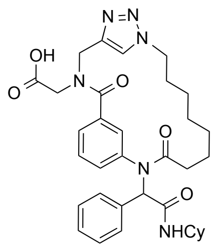 **57**	80
5	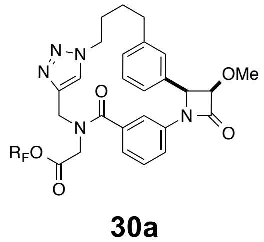 **30a**	i) $LiBH_4$ (3.6 eq), THF; ii) HCl in dioxane (4 M, 1.2 eq)	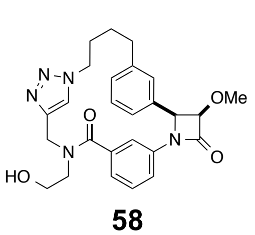 **58**	15

6	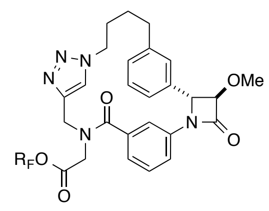 **31a**	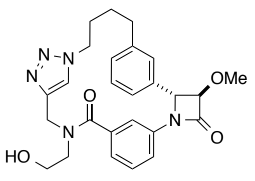 **59a**	12
		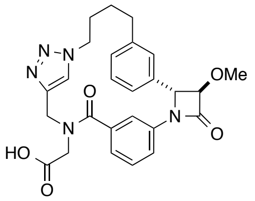 **59b**	12

Determination of stereochemistry

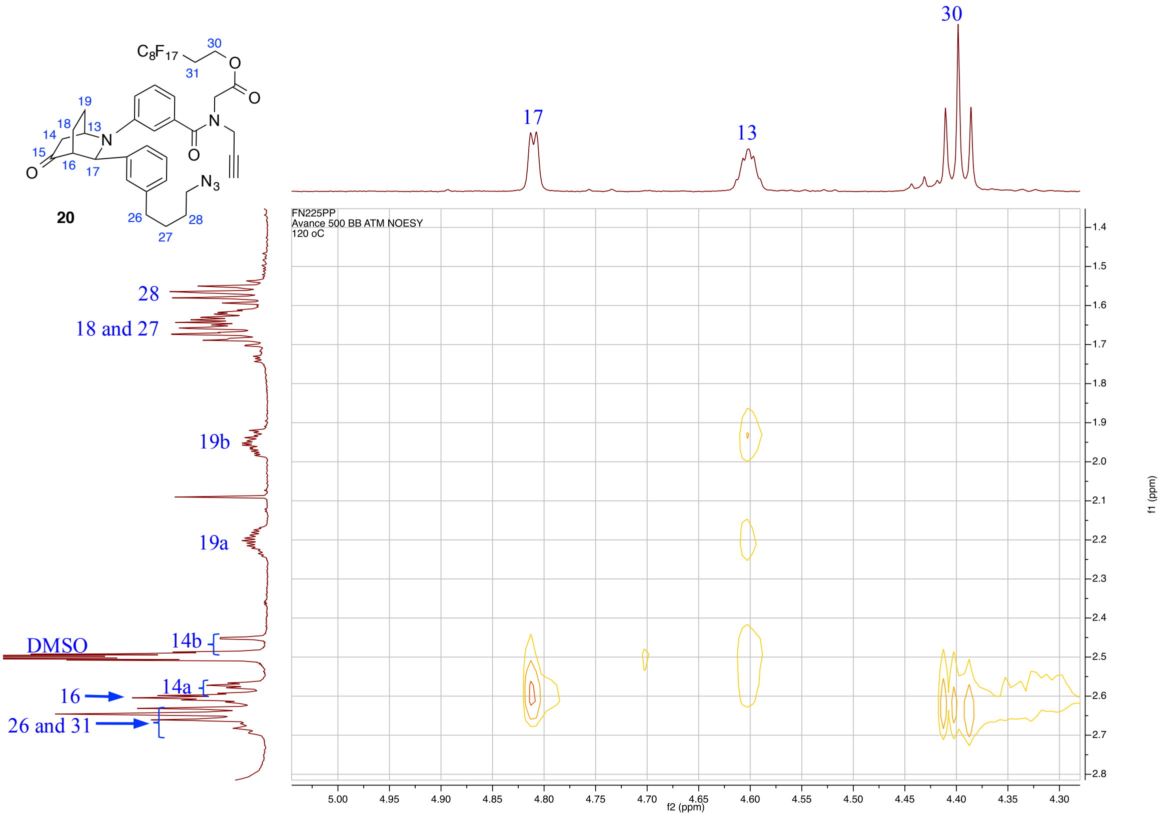

Figure S1 NOESY analysis of product 20.

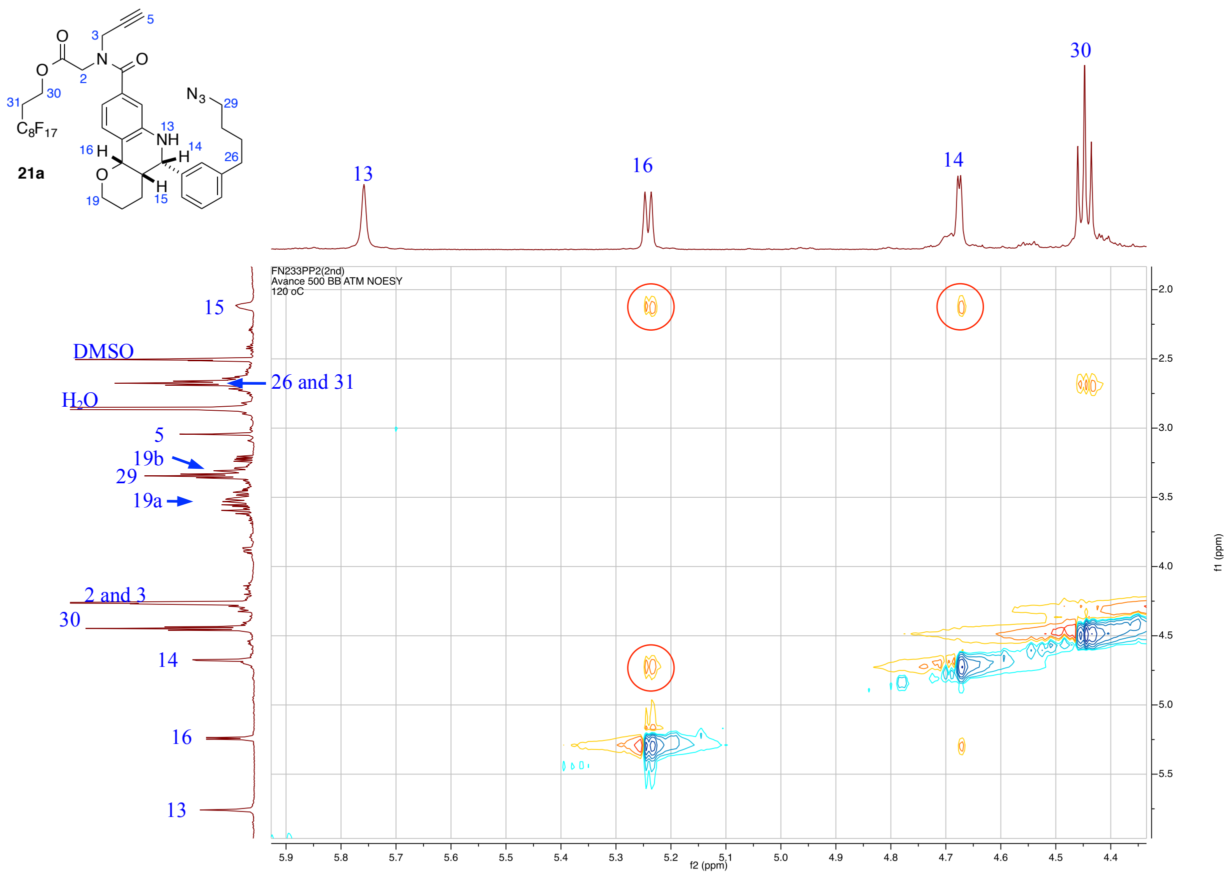

Figure S2 NOESY analysis of product 21a.

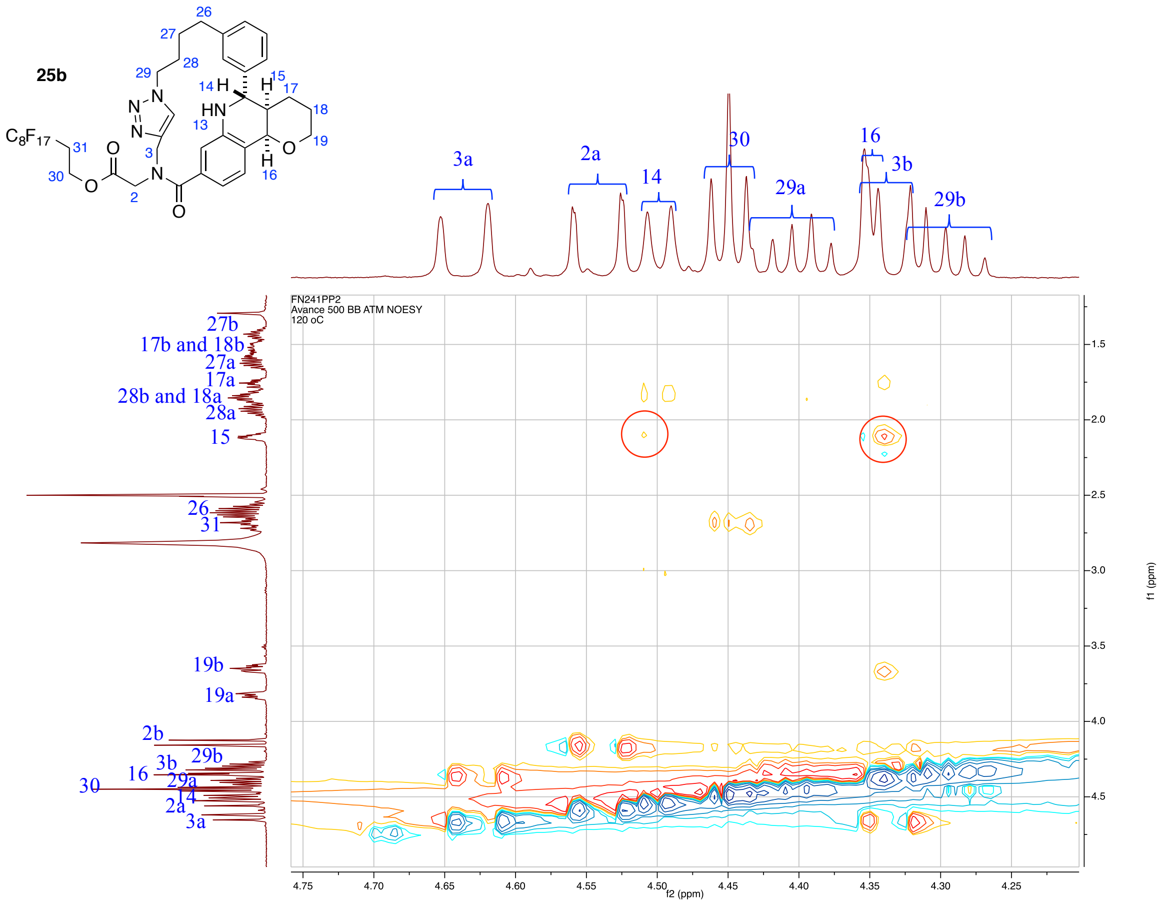

Figure S3 NOESY analysis of product 25b.

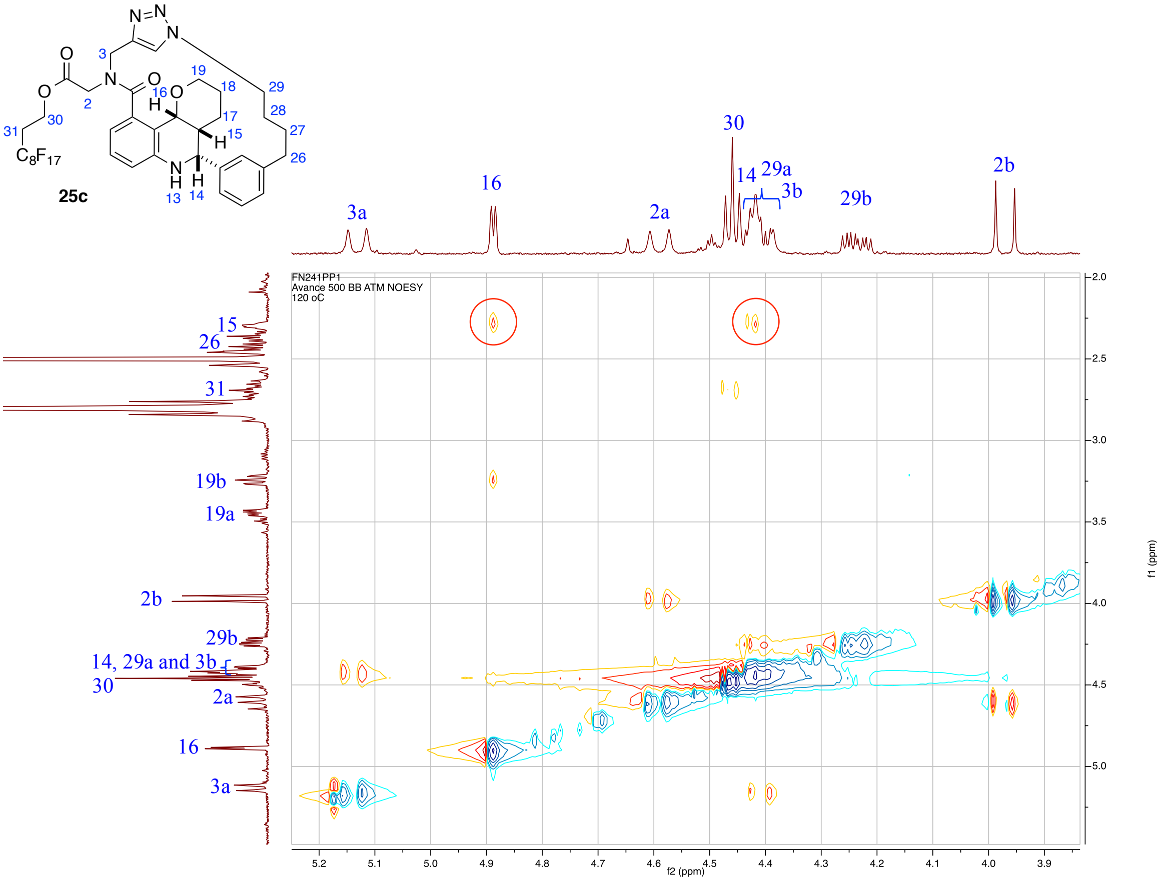

Figure S4 NOESY analysis of product 25c.

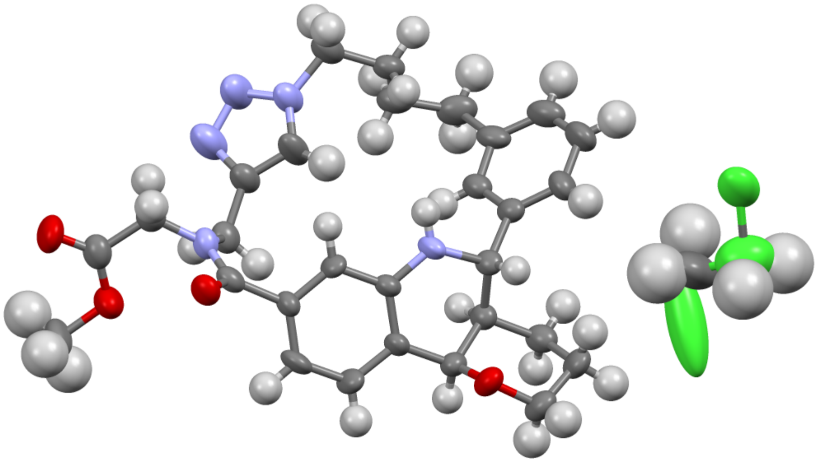

Figure S5 Crystal structure of macrocycle 49b (generated from 25b).

CCDC 1458977 contains the supplementary crystallographic data for this paper. These data can be obtained free of charge from The Cambridge Crystallographic Data Centre via www.ccdc.cam.ac.uk/data_request/cif.

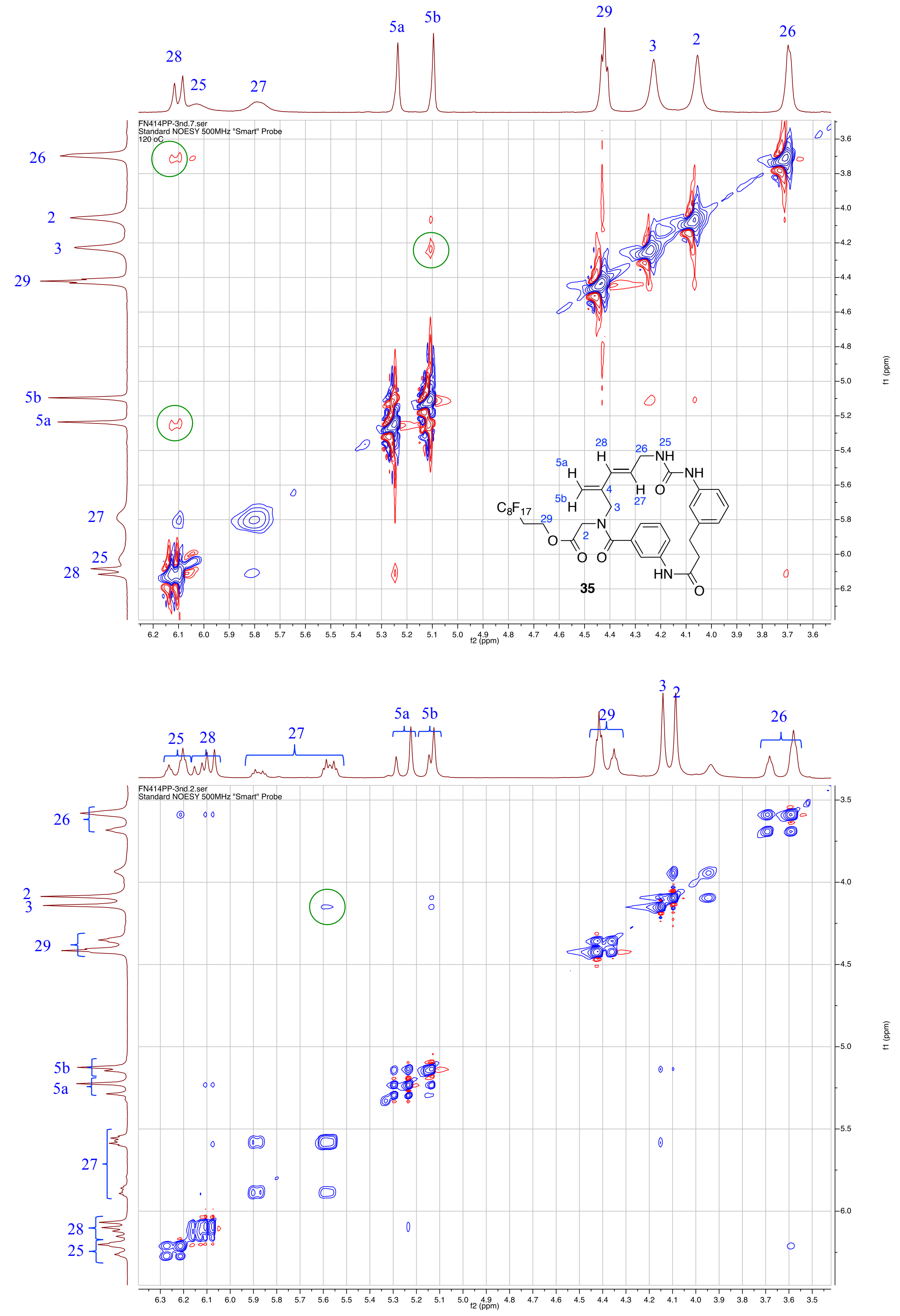

Figure S6 NOESY analysis of macrocycle 35 at 120 °C (top) and 27 °C (bottom).

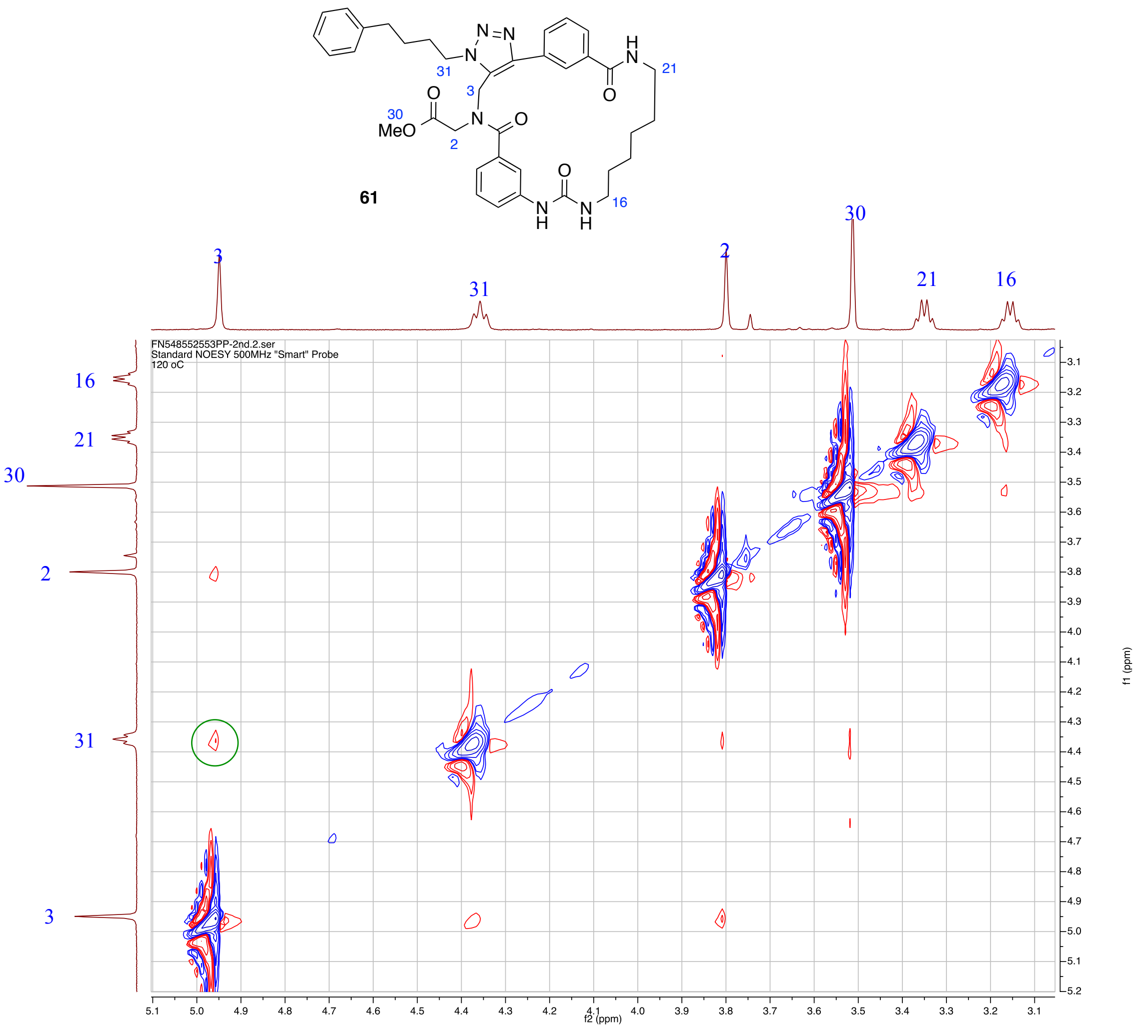

Figure S7 NOESY analysis of macrocycle 61.

List of final macrocycles

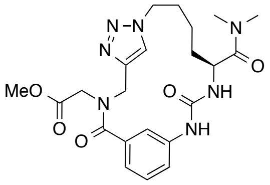
40a

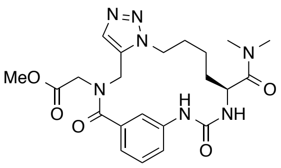
40b

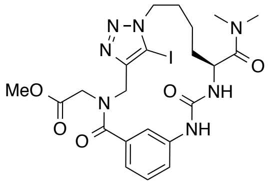
40c

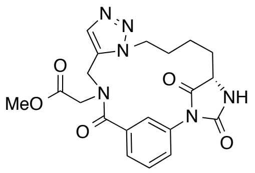
40d

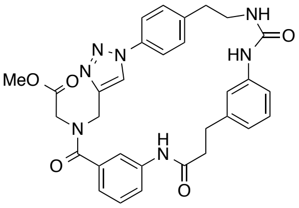
41a

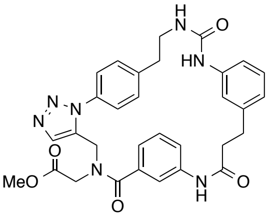
41b

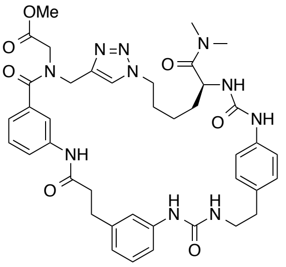
42a

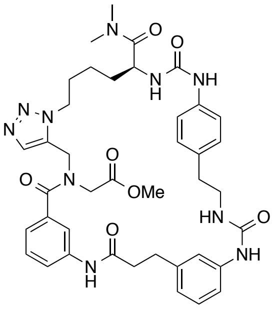
42b

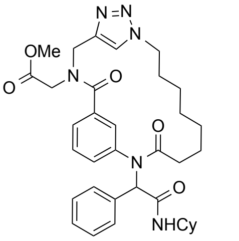
43a

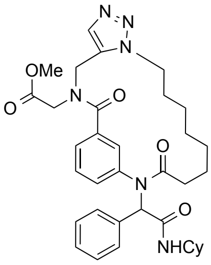
43b

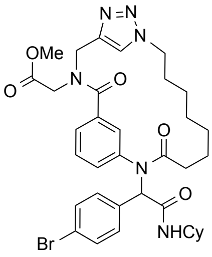
44a

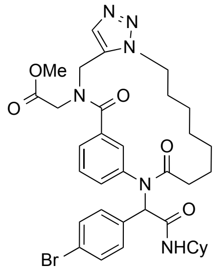
44b

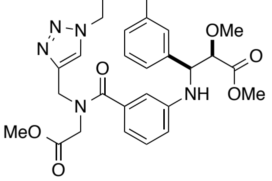
45a

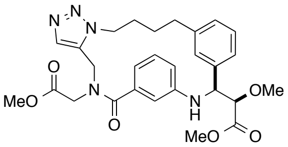
45b

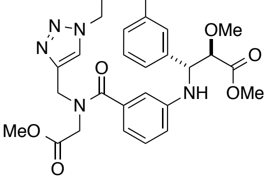
46a

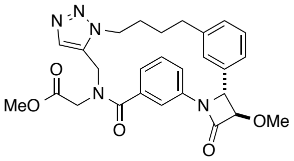
46b

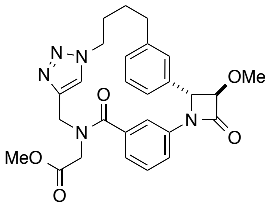
46c

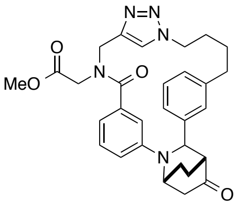
47

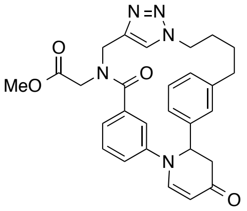
48a

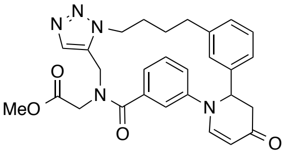
48b

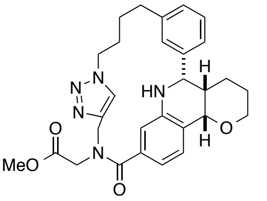
49a

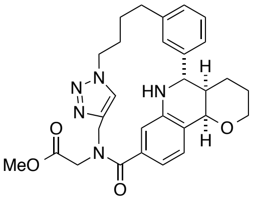
49b

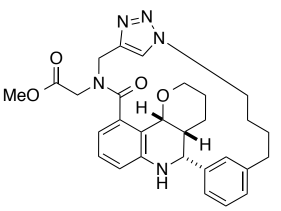
49c

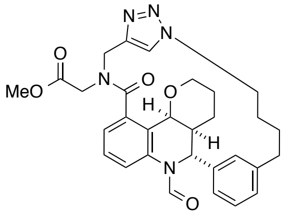
49d

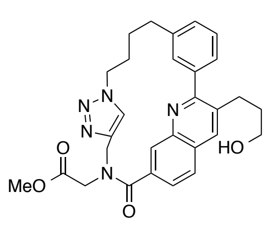

49e

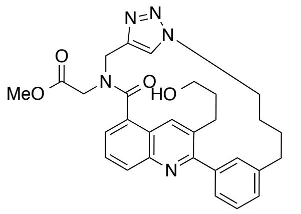

49f

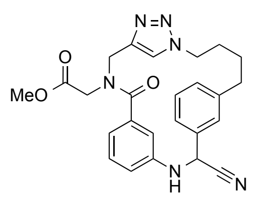

50

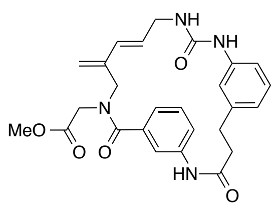

51

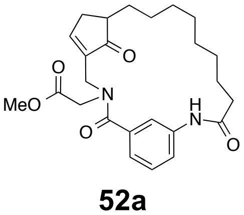

52a

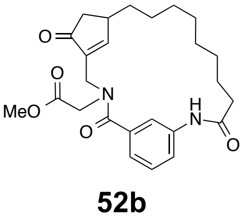

52b

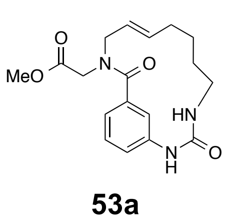

53a

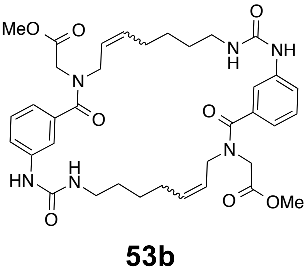

53b

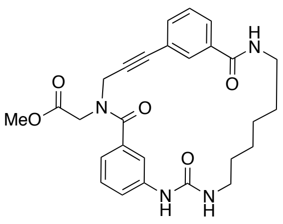

54a

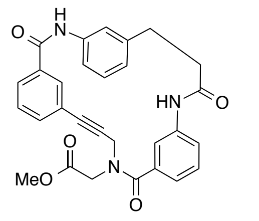

54b

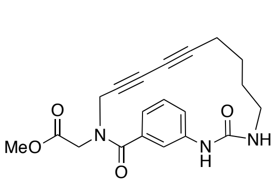

39

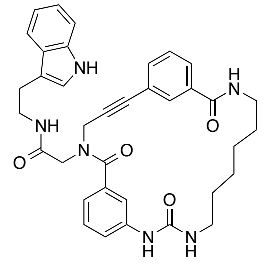

55

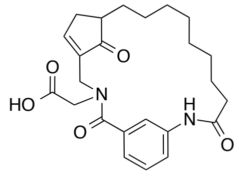

56a

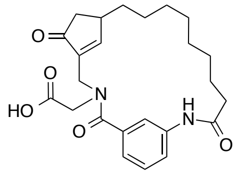

56b

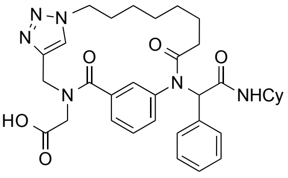

57

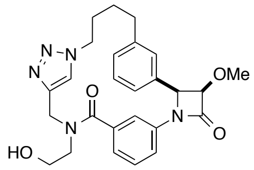

58

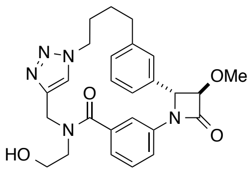

59a

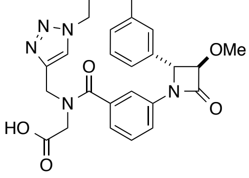

59b

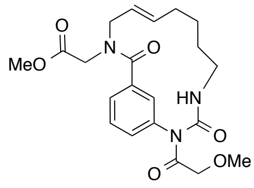

64a

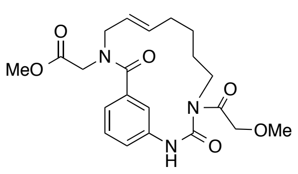

64b

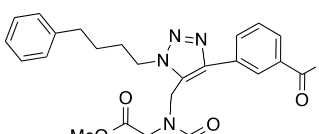

61

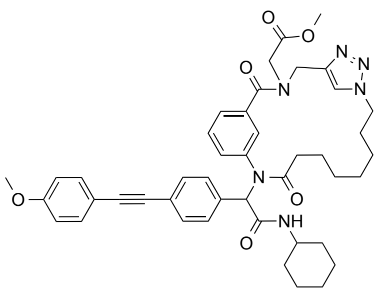

62

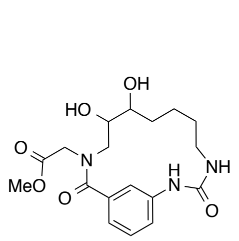

63

Macrocycles **48a** and **48b** have been reported in our previous paper.[1]

Cheminformatic analysis – Principal Moment of Inertia (PMI)

General details

Principal Moment of Inertia (PIM) was performed using Molecular Operating Environment (MOE) software package version 2012.10 from the Chemical Computing Group. Merck molecular force field 94X (MMFF94x), an all-atom force field parameterised for small organic molecules with the Generalised Born solvation model, was used to minimise the energy potential of the library members. A LowModeMD search was employed for the conformation generation. Detailed settings for conformational search are listed below.

Rejection Limit	100
RMS Gradient	0.005
Iteration Limit	10000
MM Iteration Limit	500
RMSD Limit	0.15
Energy window	3
Conformation Limit	100

Only the conformer with the lowest energy was retained for principal moment of inertia (PMI) calculations. Normalized PMI ratios (I1/I3 and I2/I3) of these conformers were obtained from MOE and then plotted on a triangular graph, with the coordinates (0,1), (0.5,0.5) and (1,1) representing a perfect rod, disc and sphere respectively (Figure S8).

Compound collections analysed

Collection 1: Macrocycle DOS library

This work, see "List of Final Macrocycles"

Collection 2: 40 high-profile synthetic drugs currently produced by the pharmaceutical industry

See: F. Kopp, C. F Stratton, L. B. Akella and D. S. Tan, *Nat. Chem. Biol.*, **2012**, *8*, 358.

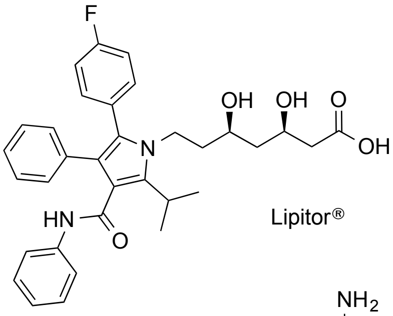
Lipitor®

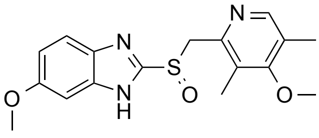
Nexium®

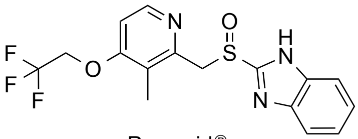
Prevacid®

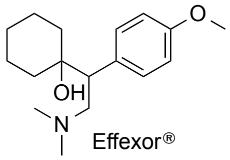
Effexor®

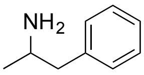
Adderall®

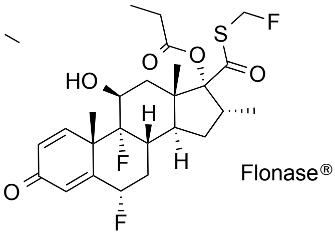
Flonase®

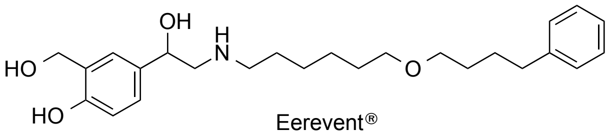
Eerevent®

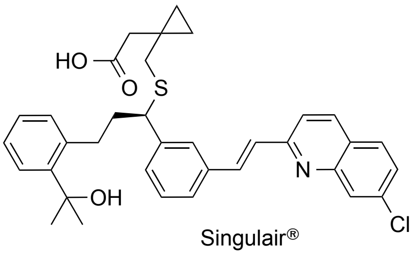
Singulair®

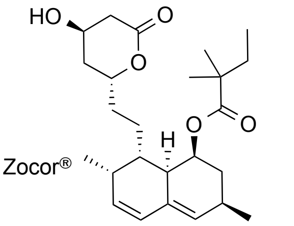
Zocor®

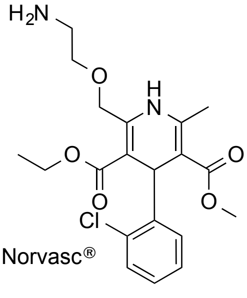
Norvasc®

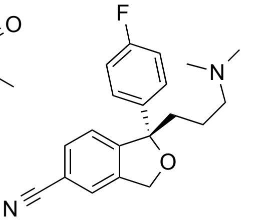
Lexapro®

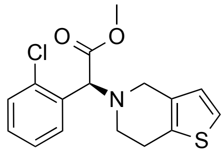
Plavix®

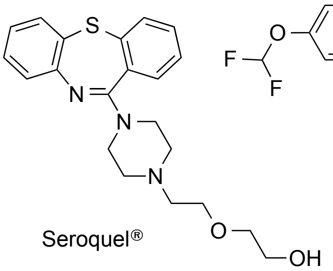
Seroquel®

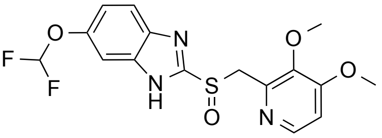
Protonix®

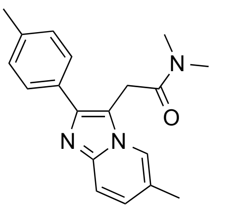
Ambien®

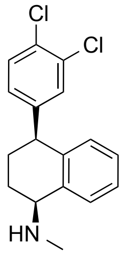
Zoloft®

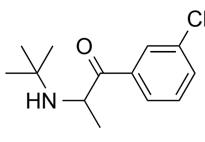
Wellbutrin®

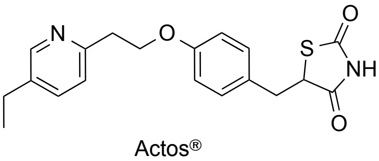
Actos®

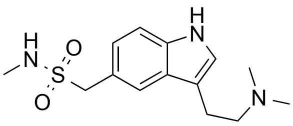
Imitrex®

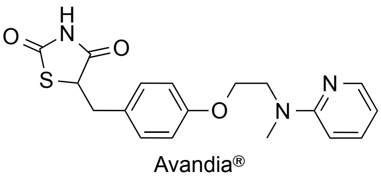
Avandia®

Collection 2: 40 high-profile synthetic drugs currently produced by the pharmaceutical industry (cont.)

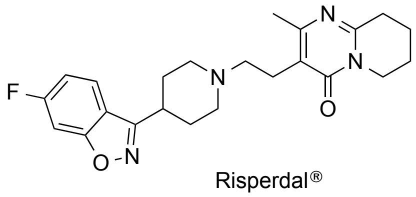

Risperdal®

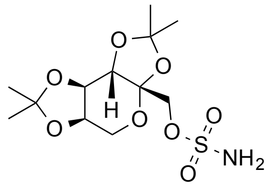

Topomax®

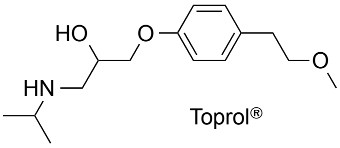

Toprol®

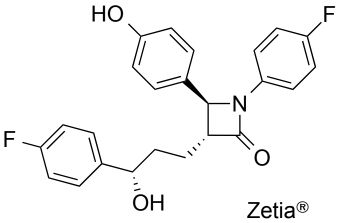

Zetia®

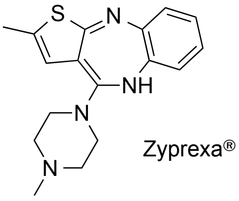

Zyprexa®

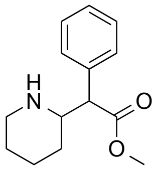

Concerta®

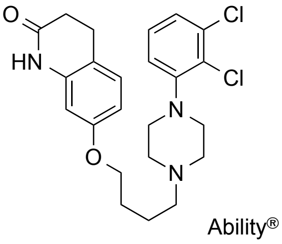

Ability®

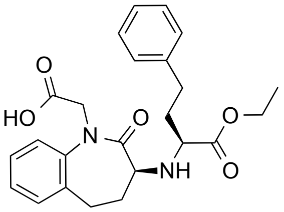

Benazepril®

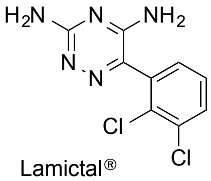

Lamictal®

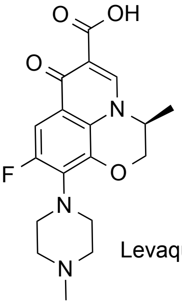

Levaquin®

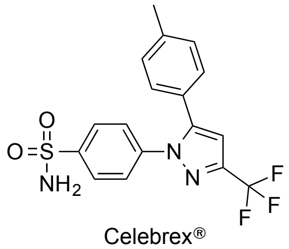

Celebrex®

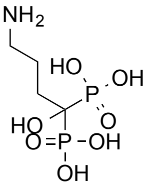

Fosamax®

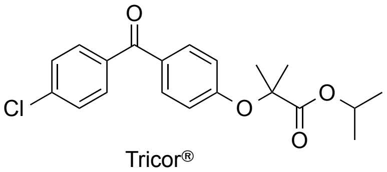

Tricor®

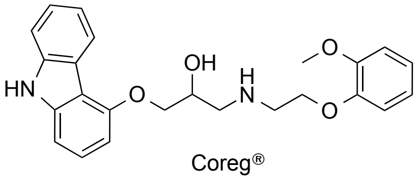

Coreg®

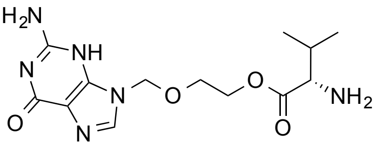

Valtrex®

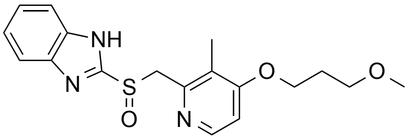

Aciphex®

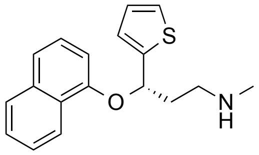

Cymbalta®

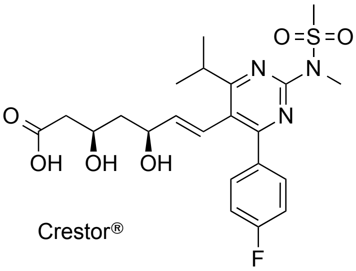

Crestor®

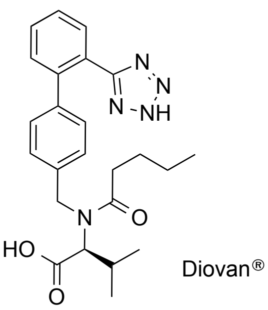

Diovan®

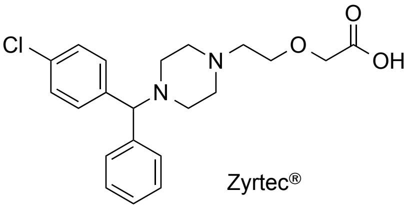

Zyrtec®

Collection 3: 60 randomly selected natural products

See: F. Kopp, C. F Stratton, L. B. Akella and D. S. Tan, *Nat. Chem. Biol.*, **2012**, *8*, 358.

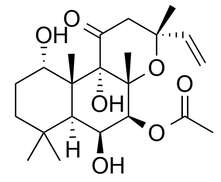

forskolin

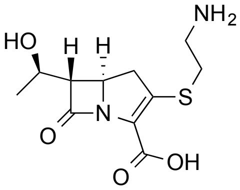

thienamycin

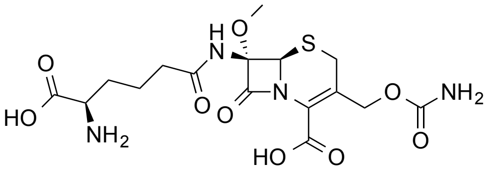

cephamycin C

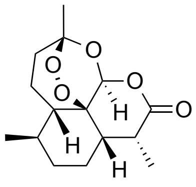

artemisinin

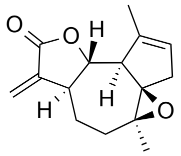

arglabin

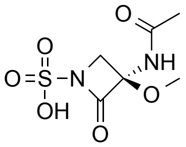

SQ26180

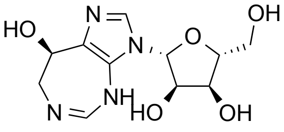

coformycin

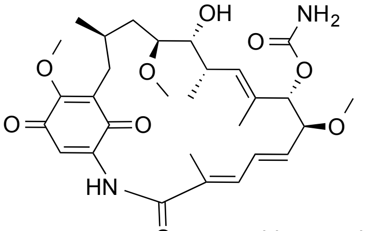

geldanamycin

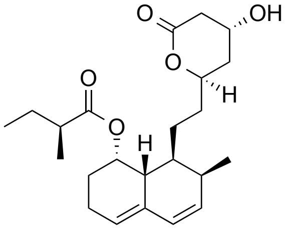

compactin

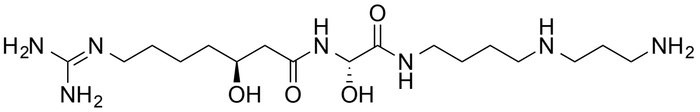

spergualin

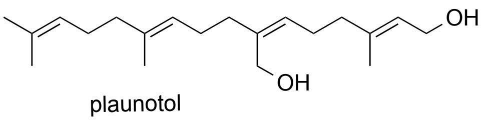

plaunotol

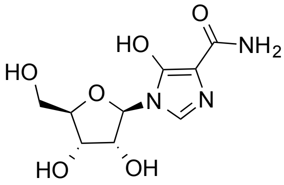

mizoribine

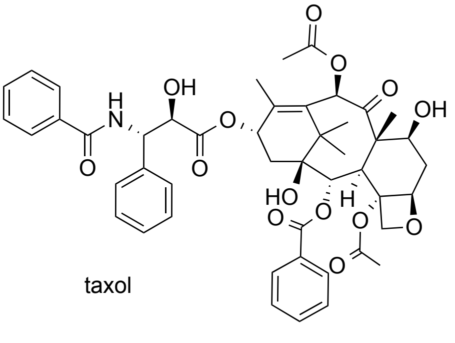

taxol

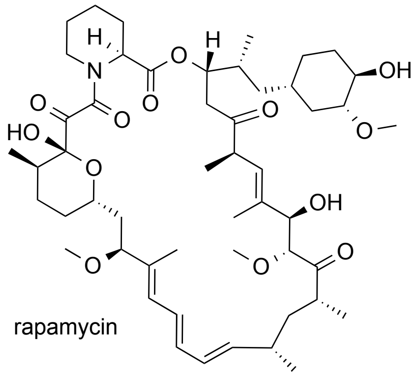

rapamycin

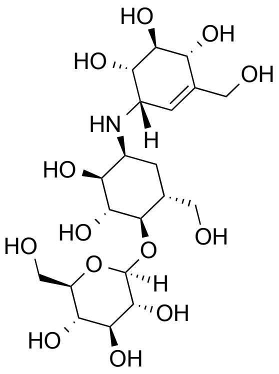

validamycin

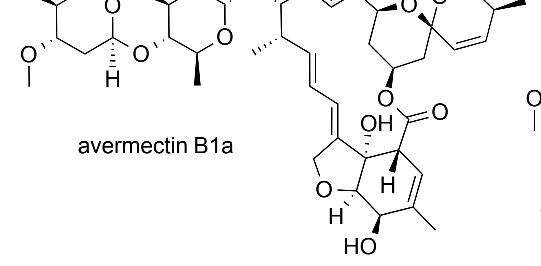

avermectin B1a

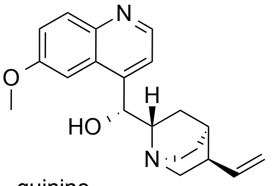

quinine

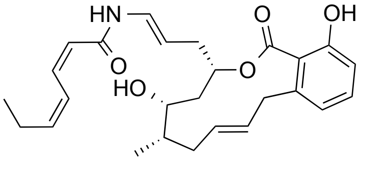

salicylihalamide A

Collection 3: 60 randomly selected natural products (cont.)

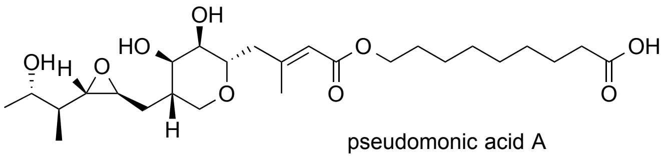

pseudomonic acid A

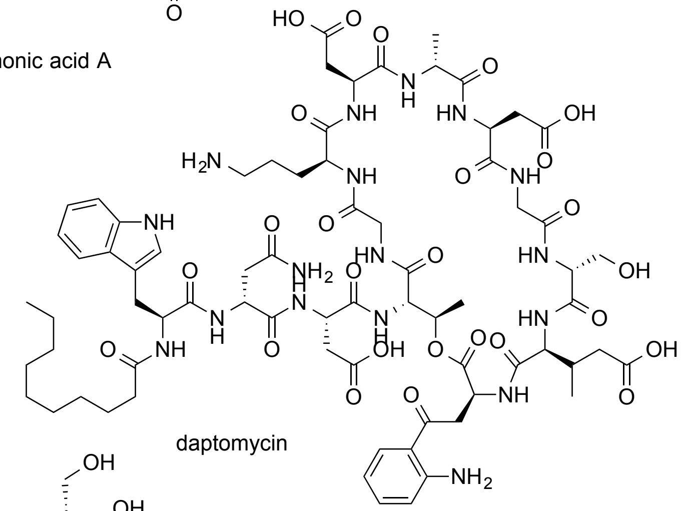

daptomycin

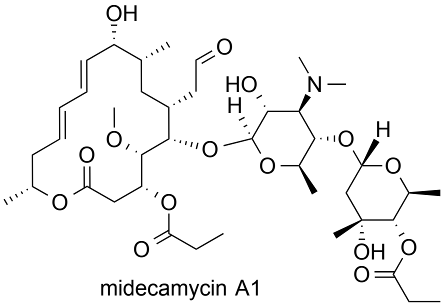

midecamycin A1

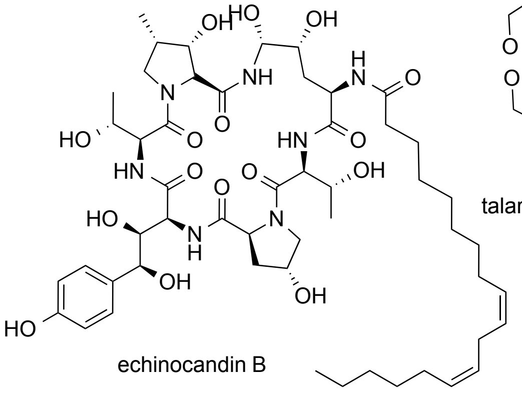

echinocandin B

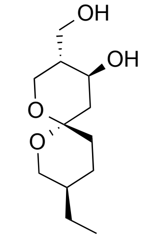

talaromycin B

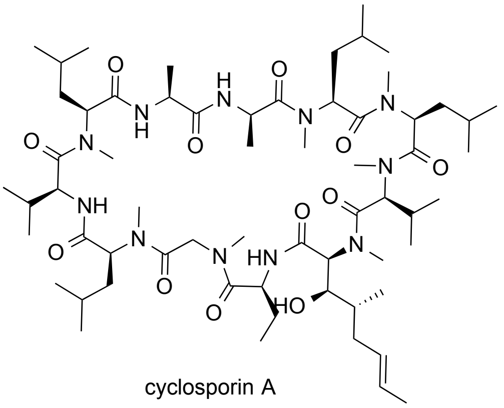

cyclosporin A

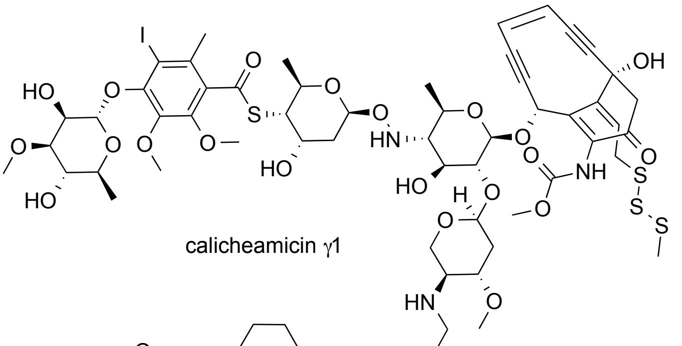

calicheamicin γ1

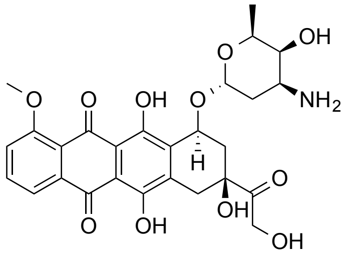

adriamycin

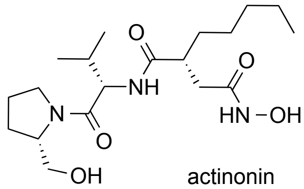

actinonin

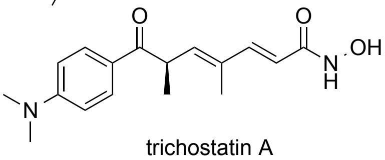

trichostatin A

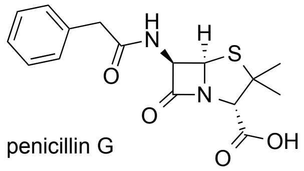

penicillin G

Collection 3: 60 randomly selected natural products (cont.)

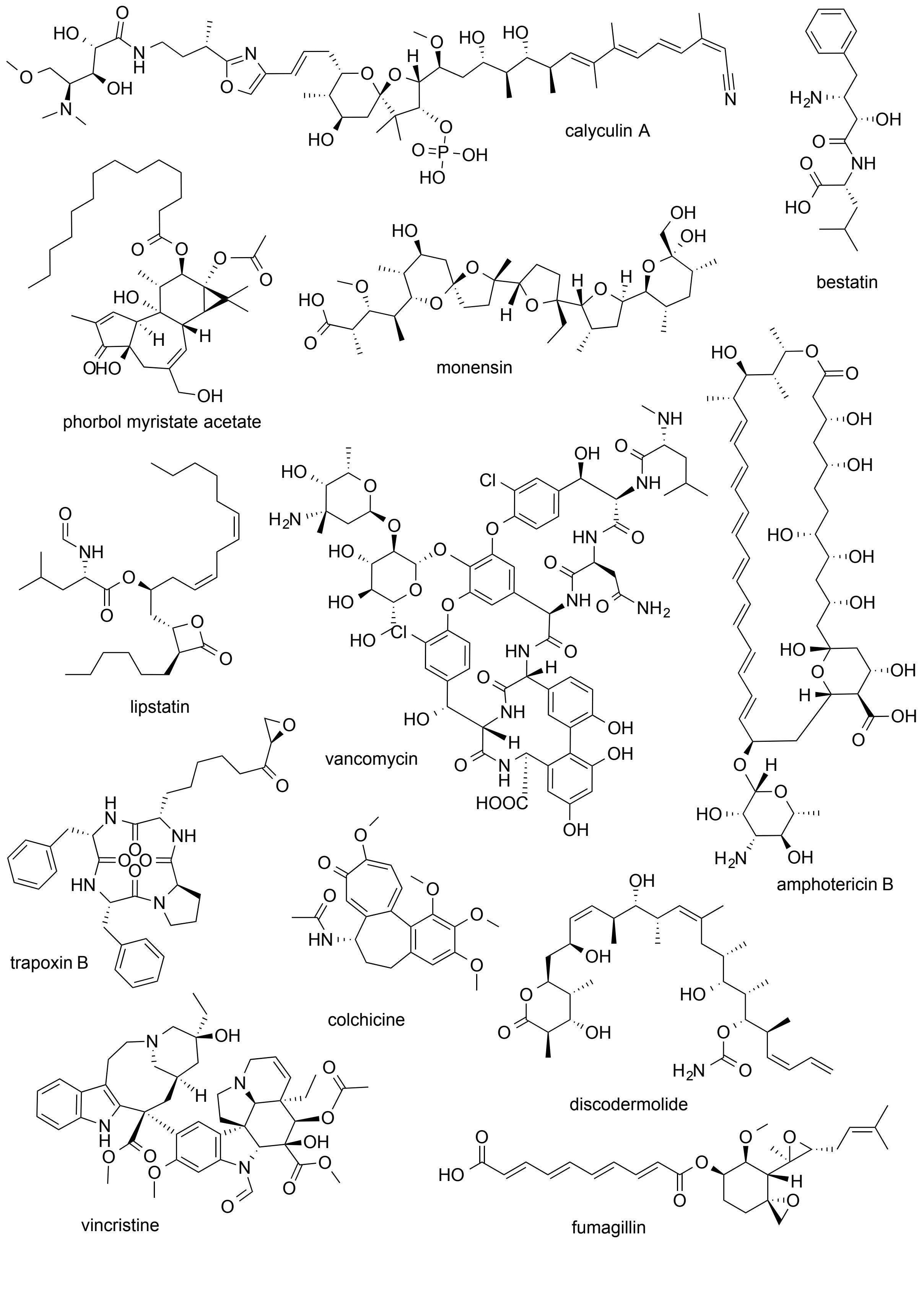

Collection 3: 60 randomly selected natural products (cont.)

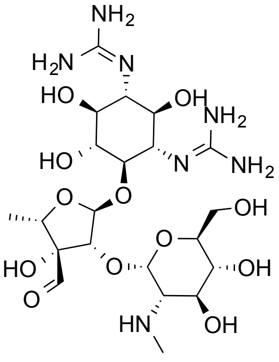

streptomycin

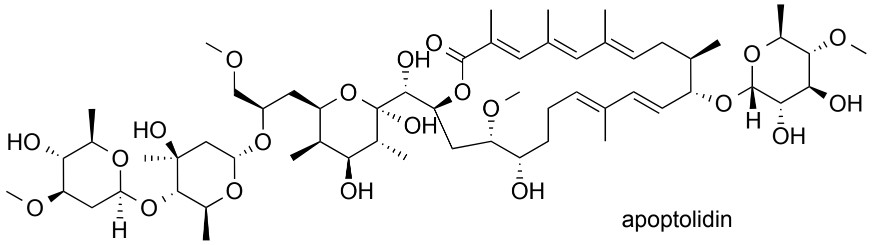

apoptolidin

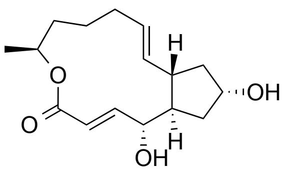

brefeldin A

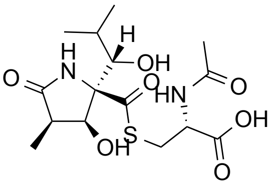

lactacystin

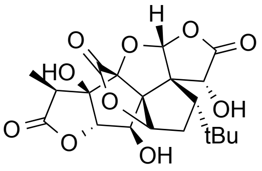

ginkgolide B

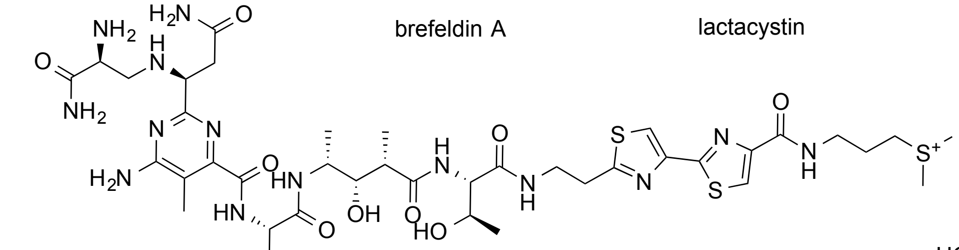
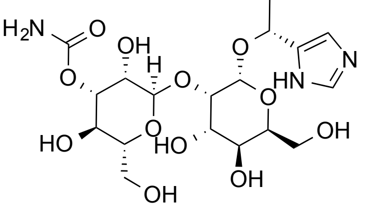

bleomycin A2

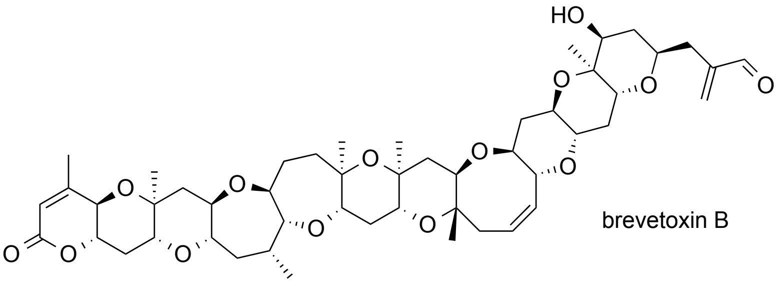

brevetoxin B

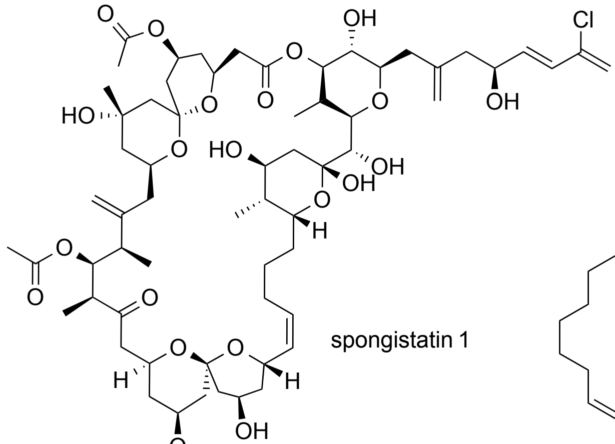

spongistatin 1

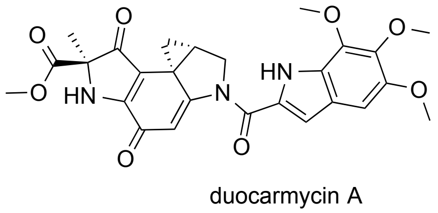

duocarmycin A

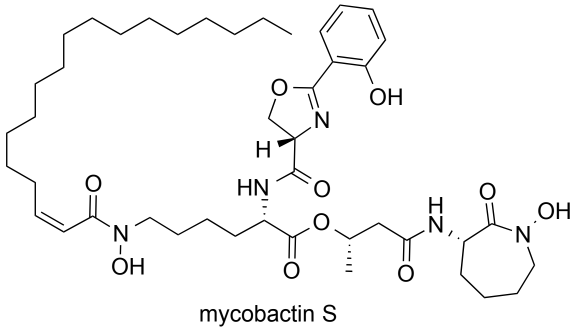

mycobactin S

Collection 3: 60 randomly selected natural products (cont.)

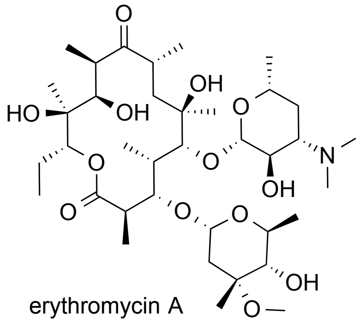

erythromycin A

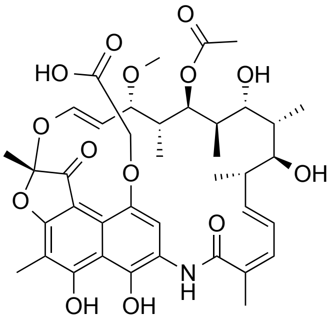

rifamycin B

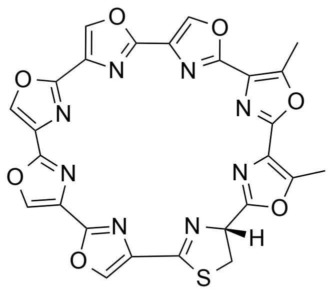

telomestatin

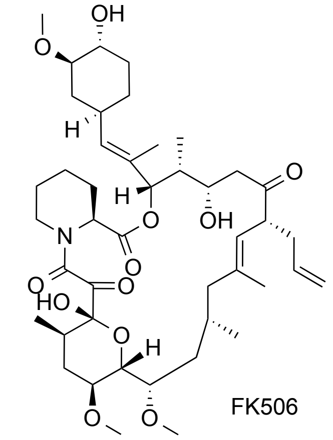

FK506

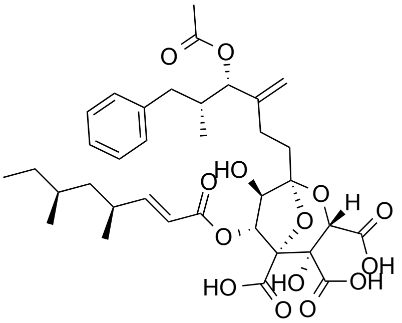

zaragozic acid A

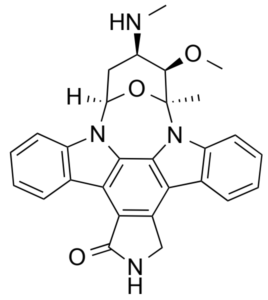

staurosporine

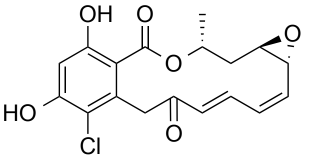

radicicol

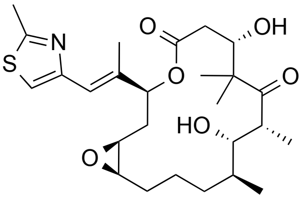

epothilone A

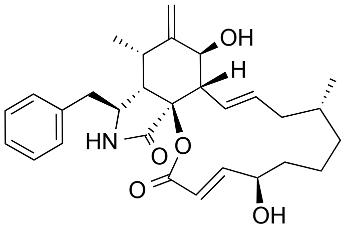

cytochalasin B

Collection 4: 36 macrocyclic-based compounds in clinical development as of April 2013

See: F. Giordanetto and J. Kihlberg, *J. Med. Chem.*, **2014**, *57*, 278-295.

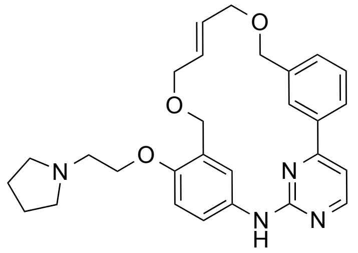

Pacritinib (ONX0803, SB1518)

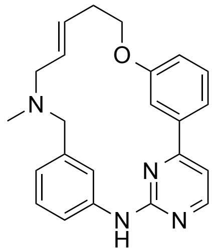

SB1317/TG02

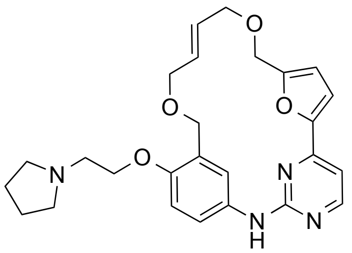

SB1578/CT1578

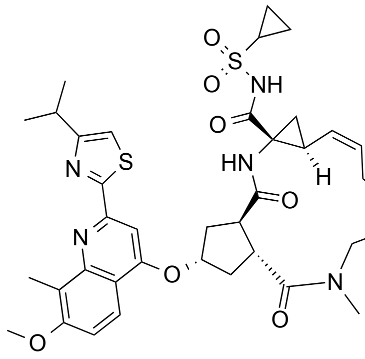

Simeprevir (TMC435)

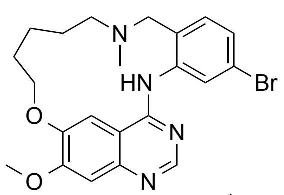

JNJ 26483327

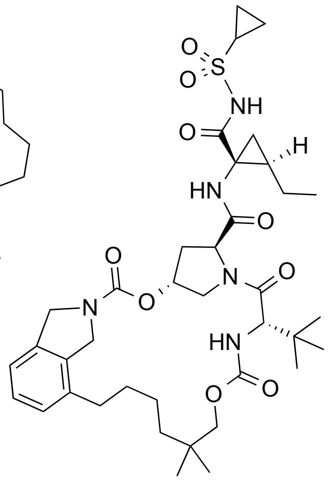

Vaniprevir (MK 7009)

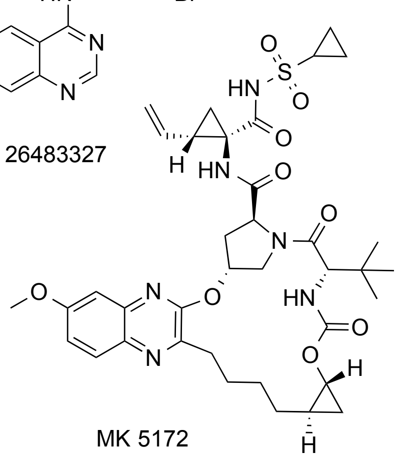

MK 5172

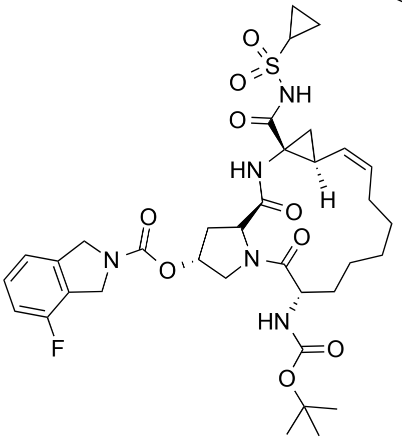

Danoprevir (R7227, ITMN191)

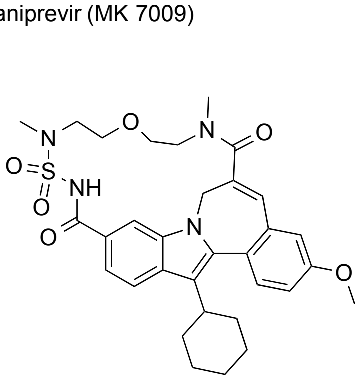

TMC647055

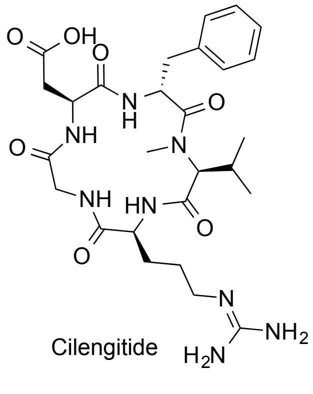

Cilengitide

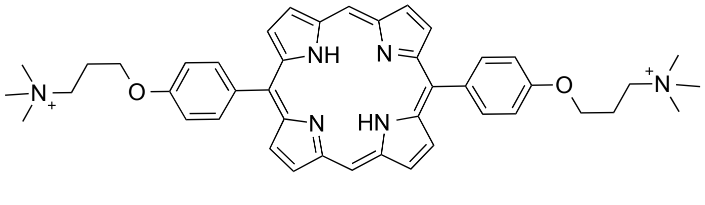

Exeporfinium (XF-73)

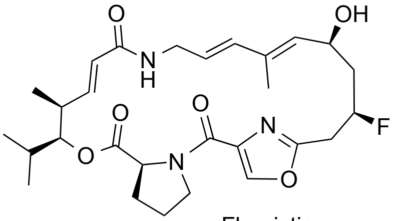

Flopristin

Collection 4: 36 macrocyclic-based compounds in clinical development (cont.)

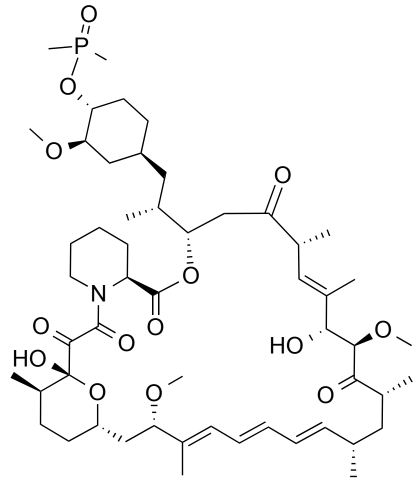

Ridaforolimus (AP23573, Deforolimus, MK 8669)

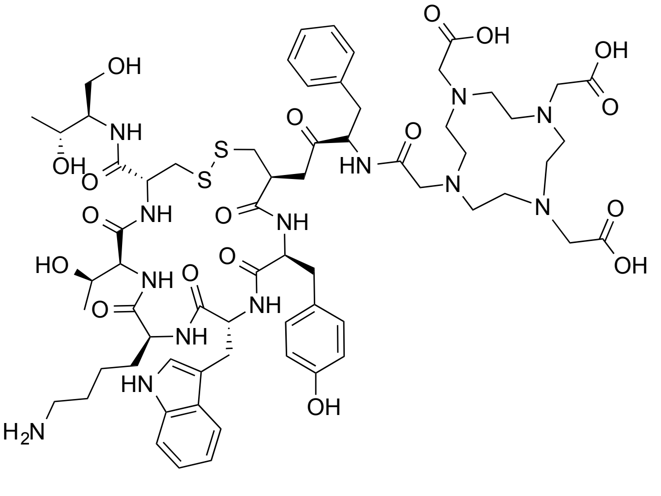

Yttrium 90-labelled edotreotide (90Y-SMT 487)

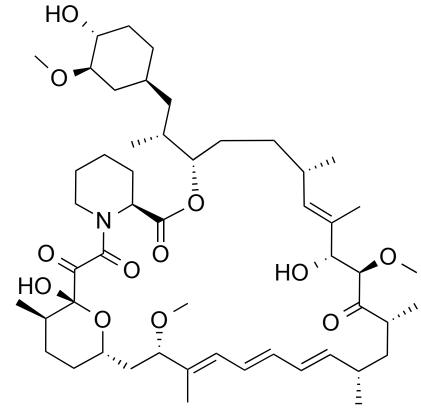

Myolimus

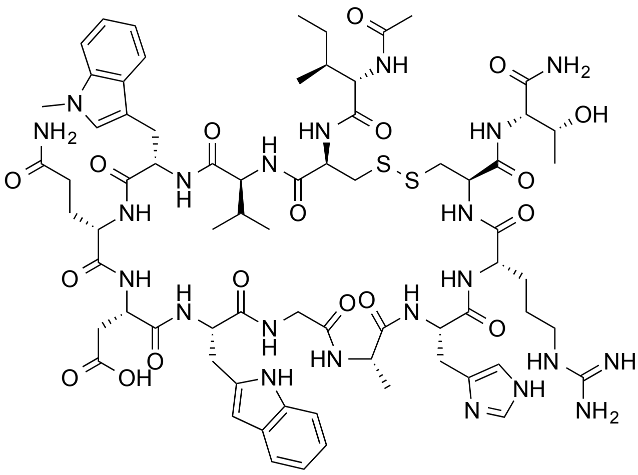

L-Threoninamide

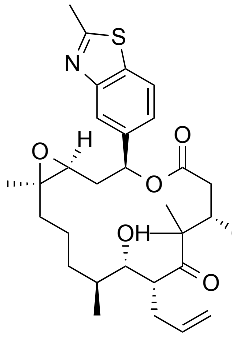

Sagopilone (BAY86-5302)

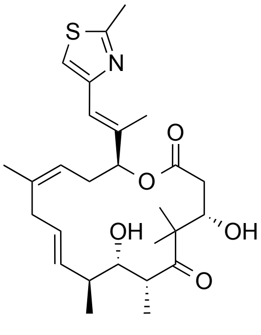

KOS 1584

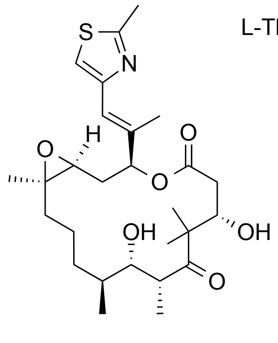

Patupilone (Epothilone B, EPO906)

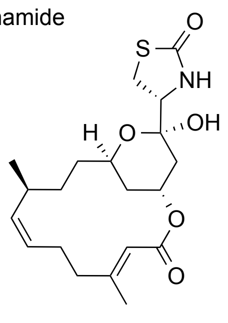

Latrunculin B (INS 116544)

Collection 4: 36 macrocyclic-based compounds in clinical development (cont.)

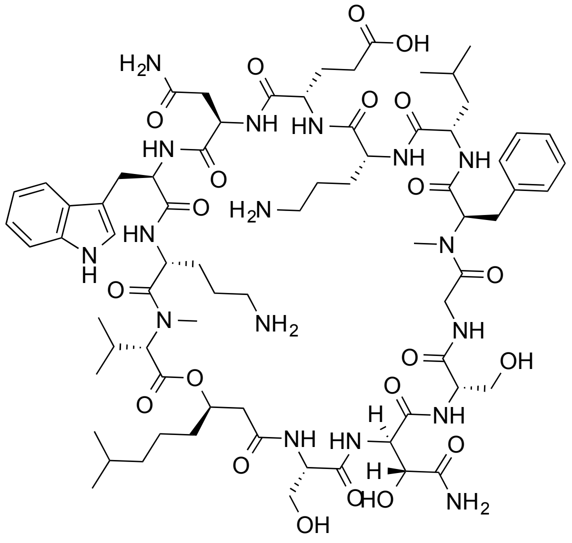

Lotilibcin (JA 002, WAP-8294A2)

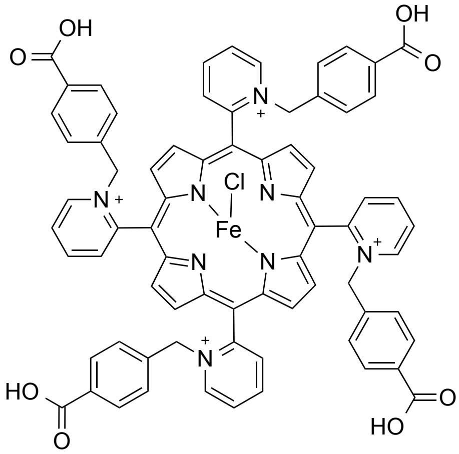

INO 4885

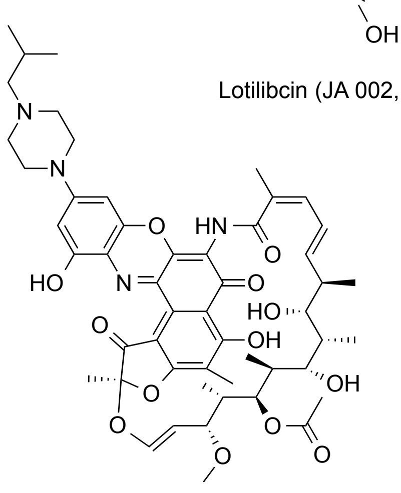

Rifalazi (BAI 1648, KRM 1648, PA 1648)

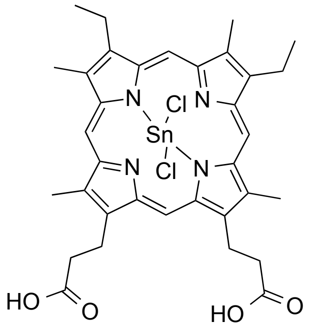

Stannsoporfin (Sn mesoporphyrin)

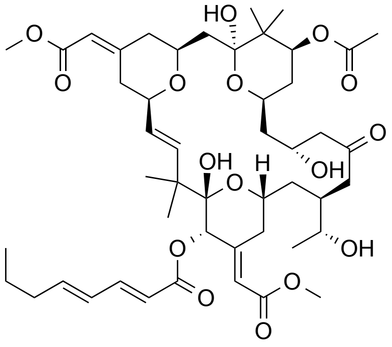

Bryostatin 1

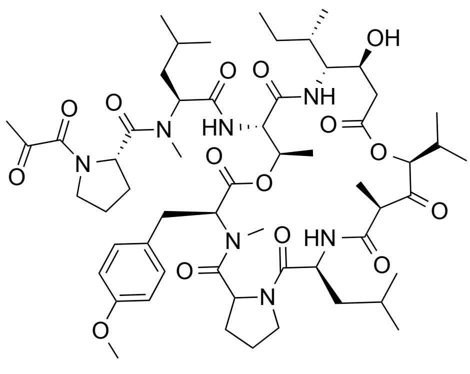

Pllitidepsin (Aplidine, Dehydrodidemnin B)

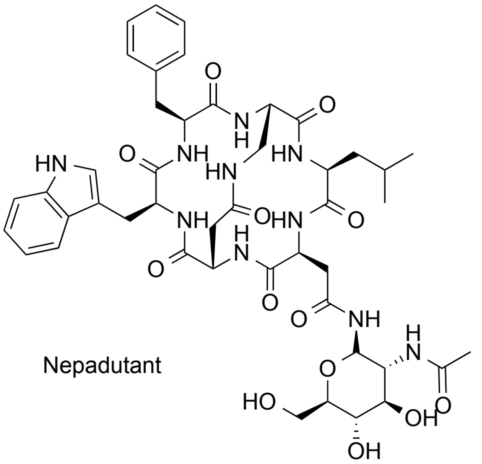

Nepadutant

Collection 4: 36 macrocyclic-based compounds in clinical development (cont.)

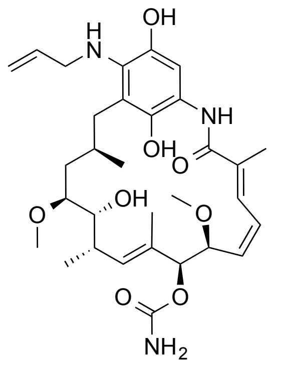

Retasspimycin (IPI 504, MEDI 561)

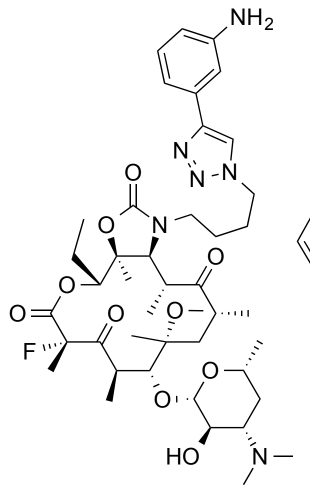

Solithromycin (CEM 101, OP-1068)

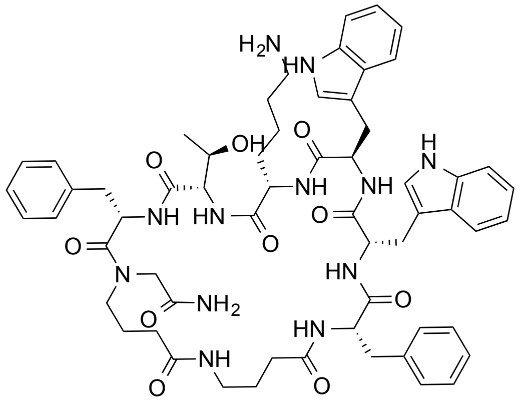

Somatoprim (DG3173, PTR 3173)

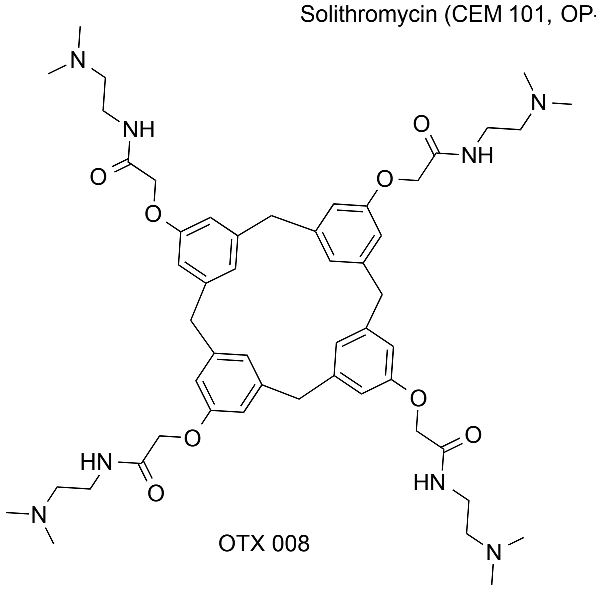

OTX 008

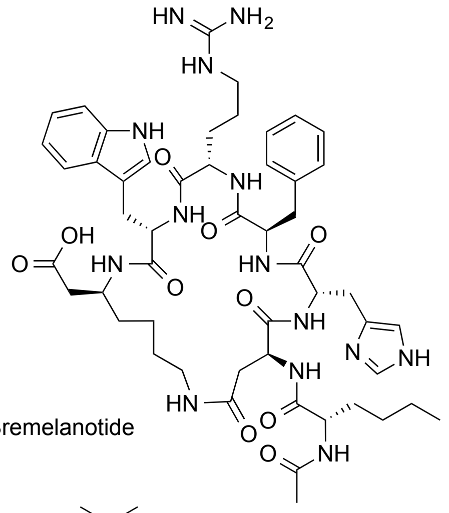

Bremelanotide

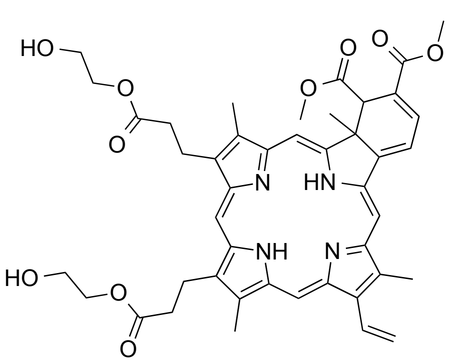

Lemuteporfin

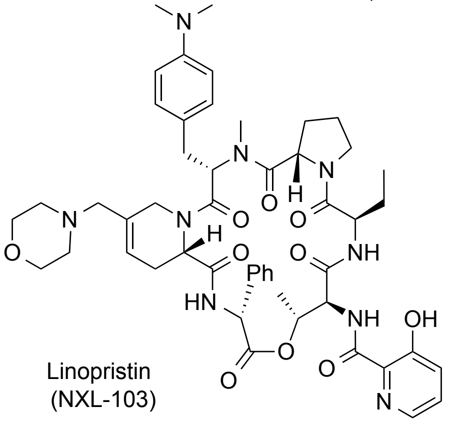

Linopristin
(NXL-103)

Collection 4: 36 macrocyclic-based compounds in clinical development (cont.)

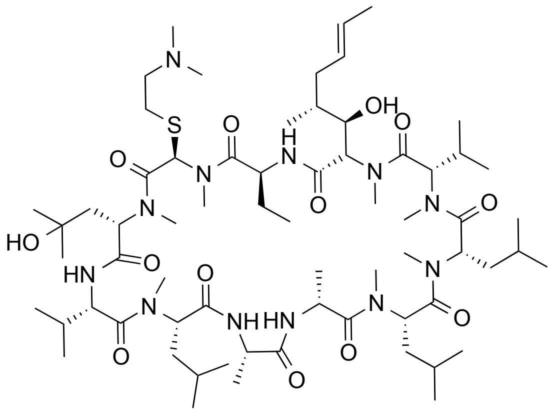

SCY 635

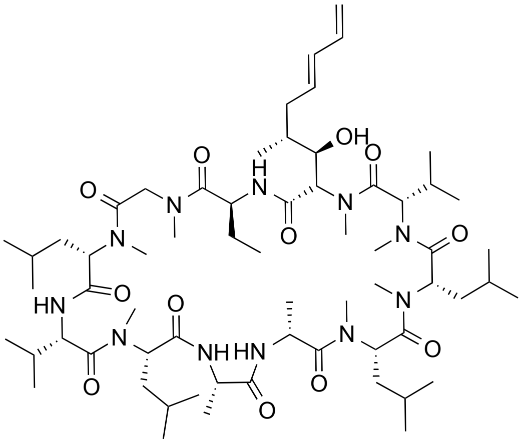

Voclosporin (ISA 247)

Table S6 Normalised PMI ratio (npr) values of conformers of the DOS library and three reference collections with the lowest energy (energy level = 0 kcal/mol).

DOS library

Compound	npr1	npr2	Compound	npr1	npr2
**43a**	0.3083	0.7773	**42b**	0.3426	0.8701
**43b**	0.6046	0.9065	**47**	0.5100	0.7133
**57**	0.5915	0.8507	**49a**	0.3393	0.7817
**62**	0.4029	0.7370	**49c**	0.4111	0.7819
**44b**	0.6075	0.7673	**49b**	0.3800	0.7297
**50**	0.5829	0.6743	**49e**	0.3166	0.8069
**44a**	0.3524	0.9226	**49f**	0.4273	0.9269
**48a**	0.5178	0.7662	**45a**	0.5866	0.8639
**48b**	0.5022	0.8329	**45b**	0.5297	0.9010
**54b**	0.5581	0.5911	**58**	0.4959	0.6502
**41a**	0.4803	0.6644	**59a**	0.5437	0.6718
**51**	0.3705	0.8382	**46a**	0.4729	0.8302
**40d**	0.3354	0.8437	**46b**	0.5930	0.9360
**42a**	0.4968	0.9552	**61**	0.5937	0.8300
**54a**	0.4886	0.7408	**39**	0.2724	0.8415
**53a**	0.3242	0.7900	**56a**	0.4669	0.7406
**40a**	0.2173	0.8626	**56b**	0.3784	0.7491
**40b**	0.2378	0.8619	**52a**	0.4857	0.7713
**53b**	0.4741	0.6937	**52b**	0.2964	0.8289
**63**	0.2923	0.8237	**55**	0.6318	0.9569
**40c**	0.3439	0.8862	**64b**	0.2273	0.9657
**64a**	0.2778	0.8326	**46c**	0.5536	0.6726
**41b**	0.5190	0.7549	**59b**	0.5643	0.6733
			**49d**	0.7296	0.7790

40 top selling drugs

Compound	npr1	npr2	Compound	npr1	npr2
Lipitor	0.3343	0.8427	Topomax	0.3721	0.7907
Nexium	0.2387	0.7858	Toprol	0.0854	0.9449

Prevacid	0.1367	0.9103	Zetia	0.3674	0.8320
Flonase	0.2843	0.9666	Fosamax	0.6565	0.7739
Servent	0.8749	0.9282	Ability	0.4836	0.6354
Singulair	0.3979	0.7155	Levaquin	0.2100	0.8459
Effexor	0.3994	0.7418	Lamictal	0.2412	0.9155
Plavix	0.3507	0.8350	Celebrex	0.3738	0.6824
Zocor	0.3846	0.7750	Benazepril	0.3379	0.9290
Norvasc	0.4396	0.8183	Zyrtec	0.3208	0.8402
Lexapro	0.4172	0.7481	Coreg	0.6401	0.7545
Seroquel	0.2078	0.9130	Valtrex	0.4538	0.8509
Protonix	0.2323	0.8070	Adderall	0.2184	0.9253
Ambien	0.3818	0.6870	Aciphex	0.1239	0.9138
Actos	0.1733	0.8826	Cymbalta	0.3327	0.7663
Zoloft	0.3094	0.9498	Crestor	0.3525	0.8687
Wellbutrin	0.1861	0.9472	Diovan	0.3509	0.9594
Avandia	0.0876	0.9585	Tricor	0.1028	0.9422
Risperdal	0.2654	0.7797	Concerta	0.5477	0.6565
Zyprexa	0.4262	0.6254	Imitrex	0.2068	0.9075

60 randomly selected natural products

Compound	npr1	npr2	Compound	npr1	npr2
Taxol	0.4444	0.7558	Ginkgolide B	0.4546	0.8718
Actinonin	0.4418	0.7805	Vancomycin	0.5097	0.6634
Discodermolide	0.1283	0.9329	Amphotericin B	0.1342	0.9067
Validamycin	0.2010	0.9501	Radicicol	0.4995	0.8727
Monensin	0.3209	0.8721	Salicylihalamide A	0.1935	0.8944
Calyculin A	0.4042	0.9305	Telomestatin	0.4927	0.5148
Coformycin	0.3093	0.8134	Rifamycin B	0.4922	0.7587
Arglabin	0.3932	0.6626	Apoptolidin	0.1922	0.8755
Mizoribine	0.2479	0.8433	Midecamycin A1	0.3650	0.9474
Forskolon	0.5081	0.7477	Zaragozic acid A	0.5011	0.7235
SQ 26180	0.3285	0.9244	Talaromycin B	0.1546	0.9504
Cephamycin C	0.5613	0.6949	Spongistatin 1	0.4968	0.8135
Avermectin B1a	0.3723	0.8151	Brevetoxin B	0.0410	0.9818
Adriamycin	0.3135	0.7704	Quinine	0.3647	0.8711

Phorbol myristate acetate	0.4660	0.7501	Mycobactin S	0.3865	0.9065
Thienamycin	0.3015	0.8545	Duocarmycin A	0.1237	0.9519
Cyclosporin A	0.4809	0.8960	Bleomycin A2	0.3651	0.9343
FK506	0.4472	0.8793	Brefeldin A	0.3068	0.7850
Trapoxin B	0.7165	0.9000	Cytochalasin B	0.4974	0.6762
Vincristine	0.5370	0.9655	Epothilone A	0.3116	0.8340
Colchicine	0.4272	0.8346	Lactacystin	0.3764	0.8347
Trichostatin A	0.2197	0.8615	Calicheamicin γ1	0.1774	0.9247
Fumagillin	0.0865	0.9668	Artemisinin	0.5476	0.6380
Staurosporine	0.4822	0.6733	Compactin	0.3930	0.7646
Erythromycin A	0.4902	0.7797	Lipstatin	0.4059	0.8457
Streptomycin	0.3162	0.9282	Pseudomonic acid A	0.3896	0.6714
Penicillin G	0.3061	0.9575	Daptomycin	0.5603	0.8611
Sperguallin	0.2793	0.8633	Bestatin	0.3910	0.7358
Rapamycin	0.6347	0.8330	Plaunotol	0.4702	0.6467
Echinocandin B	0.6140	0.8022	Geldanamycin	0.3478	0.7321

36 macrocyclic-based compounds in clinical development as of April 2013

Compound	npr1	npr2	Compound	npr1	npr2
SB 1317	0.4896	0.5512	TMC 647055	0.3184	0.7683
Pacritinib	0.3653	0.7422	Myolimus	0.4363	0.7694
Cilengitide	0.5259	0.8177	Ridaforolimus	0.3823	0.7865
Danoprevir	0.2637	0.8449	Nepadutant	0.2073	0.8926
Simeprevir	0.3020	0.8366	Retasspimycin	0.3770	0.8243
Vaniprevir	0.3093	0.8674	Solithromycin	0.4557	0.7657
SB 1578	0.3743	0.7333	90Y-SMT 487	0.7873	0.9303
MK 5172	0.4262	0.8258	L-Threoninamide	0.6347	0.8304
INO 4885	0.5670	0.7101	Bryostatin 1	0.4800	0.9631
Exeporfinium	0.0495	0.9676	Rifalazi	0.5443	0.6787
Latrunculin B	0.3334	0.8268	Lotilibcin	0.7892	0.9631
KOS 1584	0.2308	0.8601	Somatoprim	0.5858	0.6996
Patupilone	0.3284	0.7878	Flopristin	0.4072	0.8351
Sagopilone	0.4580	0.7477	Linopristin	0.4665	0.8746

Lemuteporfin	0.5257	0.6533	Bremelanotide	0.5761	0.8825
Stannsoporfin	0.4822	0.7402	Pllitidepsin	0.5758	0.7715
JNJ 26483327	0.2843	0.7538	SCY 635	0.5172	0.8594
OXT 008	0.4640	0.7134	Voclosporin	0.4682	0.8587

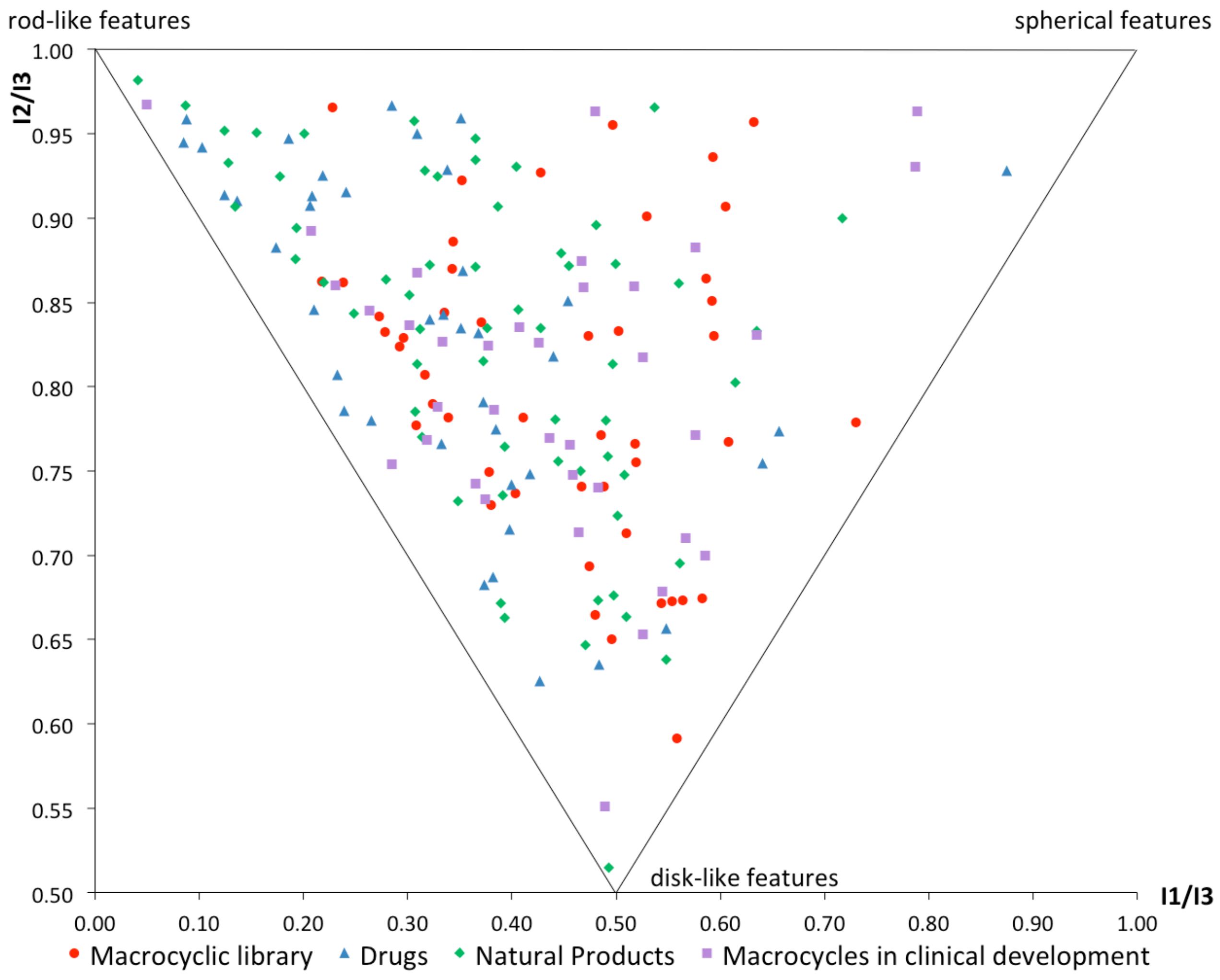

Figure S8. Comparative PMI plot of DOS library with other molecular collections

PMI plot illustrating the molecular shape diversity of the DOS library and three reference libraries. Macrocyclic DOS library (Collection 1; red dots); 40 high-profile synthetic drugs currently produced by the pharmaceutical industry drugs (Collection 2;[2] blue triangle); 60 natural products representing a diverse range of biological activities and biosynthetic origins (Collection 3;[2] green rhombus); 36 macrocyclic-based compounds in clinical development as of April 2013 (Collection 4;[3] purple square). X axis (npr1): PMI1/ PMI3; Y axis (npr2): PMI2/ PMI3.

Analysis of comparative PMI plot

The synthetic drug (non-macrocyclic) collection predominately contains compounds with rod-like shapes with some disk-like features. The natural products and macrocyclic-based compound collections span a significantly wider range of molecular shape space, from sp^2 features extending into spherical features. The synthesised macrocyclic DOS library also displays a high level of

molecular shape diversity and covers a molecular shape space comparatively as broad as natural products, overlapping to a substantial extent with the drug collection as well as the macrocycles in clinical development.

Table S7 Chemical structures of conformers of the DOS library and three reference collections in SMILE.

DOS library

**43a**	O=C1N(c2cc(ccc2)C(=O)N(Cc2nnn(CCCCCCC1)c2)CC(OC)=O)[C@H](C(=O)NC1CCCCC1)c1ccccc1
**43b**	O=C1N(c2cc(ccc2)C(=O)N(Cc2n(nnc2)CCCCCCC1)CC(OC)=O)[C@@H](C(=O)NC1CCCCC1)c1ccccc1
**57**	O=C1N(c2cc(ccc2)C(=O)N(Cc2nnn(CCCCCCC1)c2)CC(=O)[O-])[C@H](C(=O)NC1CCCCC1)c1ccccc1
**62**	O(C)c1ccc(cc1)C#Cc1ccc(cc1)[C@@H](N1c2cc(ccc2)C(=O)N(Cc2nnn(CCCCCCCC1=O)c2)CC(OC)=O)C(=O)NC1CCCCC1
**44b**	Brc1ccc(cc1)[C@@H](N1c2cc(ccc2)C(=O)N(Cc2n(nnc2)CCCCCCCC1=O)CC(OC)=O)C(=O)NC1CCCCC1
**50**	O=C1N(Cc2nnn(CCCCc3cc(ccc3)[C@H](Nc3cc1ccc3)C#N)c2)CC(OC)=O
**44a**	Brc1ccc(cc1)[C@H](N1c2cc(ccc2)C(=O)N(Cc2nnn(CCCCCCCC1=O)c2)CC(OC)=O)C(=O)NC1CCCCC1
**48a**	O=C1C=CN2[C@@H](C1)c1cc(CCCCn3nnc(CN(CC(OC)=O)C(=O)c4cc2ccc4)c3)ccc1
**48b**	O=C1C=CN2[C@@H](C1)c1cc(CCCCn3nncc3CN(CC(OC)=O)C(=O)c3cc2ccc3)ccc1
**54b**	O=C1N(CC#Cc2cc(ccc2)C(=O)Nc2cc(CCC(=O)Nc3cc1ccc3)ccc2)CC(OC)=O
**41a**	O=C1N(Cc2nnn(-c3ccc(CCNC(=O)Nc4cc(CCC(=O)Nc5cc1ccc5)ccc4)cc3)c2)CC(OC)=O
**51**	O=C1N(CC(\C=C\CNC(=O)Nc2cc(CCC(=O)Nc3cc1ccc3)ccc2)=C)CC(OC)=O
**40d**	O=C1N(Cc2n(nnc2)CCCC[C@@H]2NC(=O)N(c3cc1ccc3)C2=O)CC(OC)=O
**42a**	O=C1N(Cc2nnn(CCCC[C@H](NC(=O)Nc3ccc(CCNC(=O)Nc4cc(CCC(=O)Nc5cc1ccc5)ccc4)cc3)C(=O)N(C)C)c2)CC(OC)=O
**54a**	O=C1N(CC#Cc2cc(ccc2)C(=O)NCCCCCCNC(=O)Nc2cc1ccc2)CC(OC)=O
**53a**	O=C1N(C\C=C\CCCCNC(=O)Nc2cc1ccc2)CC(OC)=O
**40a**	O=C1N(Cc2nnn(CCCC[C@H](NC(=O)Nc3cc1ccc3)C(=O)N(C)C)c2)CC(OC)=O
**40b**	O=C1N(Cc2n(nnc2)CCCC[C@H](NC(=O)Nc2cc1ccc2)C(=O)N(C)C)CC(OC)=O
**53b**	O=C1N(C\C=C/CCCCNC(=O)Nc2cc(ccc2)C(=O)N(C\C=C/CCCCNC(=O)Nc2cc1ccc2)CC(OC)=O)CC(OC)=O
**63**	O=C1N(C[C@@H](O)[C@H](O)CCCCNC(=O)Nc2cc1ccc2)CC(OC)=O
**40c**	Ic1n2nnc1CN(CC(OC)=O)C(=O)c1cc(NC(=O)N[C@@H](CCCC2)C(=O)N(C)C)ccc1
**64a**	O=C1N(C\C=C\CCCCNC(=O)N(c2cc1ccc2)C(=O)COC)CC(OC)=O
**41b**	O=C1N(Cc2n(nnc2)-c2ccc(CCNC(=O)Nc3cc(CCC(=O)Nc4cc1ccc4)ccc3)cc2)CC(OC)=O
**42b**	O=C1N(Cc2n(nnc2)CCCC[C@H](NC(=O)Nc2ccc(CCNC(=O)Nc3cc(CCC(=O)Nc4cc1ccc4)ccc3)cc2)C(=O)N(C)C)CC(OC)=O
**47**	O=C1[C@@H]2[C@H]3N([C@@H](C1)CC2)c1cc(ccc1)C(=O)N(Cc1nnn(CCCCc2cc3ccc2)c1)CC(OC)=O

**49a**	O1[C@H]2[C@H]([C@@H]3Nc4c2ccc(c4)C(=O)N(Cc2nnn(CCCCc4cc3ccc4)c2)CC(OC)=O)CCC1
**49c**	O1[C@@H]2[C@@H]([C@H]3Nc4c2c(ccc4)C(=O)N(Cc2nnn(CCCCc4cc3ccc4)c2)CC(OC)=O)CCC1
**49b**	O1[C@@H]2[C@@H]([C@@H]3Nc4c2ccc(c4)C(=O)N(Cc2nnn(CCCCc4cc3ccc4)c2)CC(OC)=O)CCC1
**49e**	O=C1N(Cc2nnn(CCCCc3cc(-c4nc5c(cc4CCCO)ccc1c5)ccc3)c2)CC(OC)=O
**49f**	O=C1N(Cc2nnn(CCCCc3cc(-c4nc5c(c1ccc5)cc4CCCO)ccc3)c2)CC(OC)=O
**45a**	O=C1N(Cc2nnn(CCCCc3cc(ccc3)[C@H](Nc3cc1ccc3)[C@@H](OC)C(OC)=O)c2)CC(OC)=O
**45b**	O=C1N(Cc2n(nnc2)CCCCc2cc(ccc2)[C@H](Nc2cc1ccc2)[C@@H](OC)C(OC)=O)CC(OC)=O
**58**	O=C1N2[C@@H](c3cc(CCCCn4nnc(CN(CCO)C(=O)c5cc2ccc5)c4)ccc3)[C@H]1OC
**59a**	O=C1N2[C@H](c3cc(CCCCn4nnc(CN(CCO)C(=O)c5cc2ccc5)c4)ccc3)[C@H]1OC
**46a**	O=C1N(Cc2nnn(CCCCc3cc(ccc3)[C@@H](Nc3cc1ccc3)[C@@H](OC)C(OC)=O)c2)CC(OC)=O
**46b**	O=C1N2[C@H](c3cc(CCCCn4nncc4CN(CC(OC)=O)C(=O)c4cc2ccc4)ccc3)[C@H]1OC
**61**	O=C1N(Cc2n(nnc2-c2cc(ccc2)C(=O)NCCCCCCNC(=O)Nc2cc1ccc2)CCCCc1ccccc1)CC(OC)=O
**39**	O=C1N(CC#CC#CCCCCNC(=O)Nc2cc1ccc2)CC(OC)=O
**56a**	O=C1C=2CN(CC(=O)[O-])C(=O)c3cc(NC(=O)CCCCCCCC[C@@H](C1)C=2)ccc3
**56b**	O=C1[C@H]2CCCCCCCCC(=O)Nc3cc(ccc3)C(=O)N(CC1=CC2)CC(=O)[O-]
**52a**	O=C1C=2CN(CC(OC)=O)C(=O)c3cc(NC(=O)CCCCCCCC[C@H](C1)C=2)ccc3
**52b**	O=C1[C@@H]2CCCCCCCCC(=O)Nc3cc(ccc3)C(=O)N(CC1=CC2)CC(OC)=O
**55**	O=C1N(CC#Cc2cc(ccc2)C(=O)NCCCCCCNC(=O)Nc2cc1ccc2)CC(=O)NCCc1c2c([nH]c1)cccc2
**64b**	O=C1N(C\C=C\CCCCN(C(=O)COC)C(=O)Nc2cc1ccc2)CC(OC)=O
**46c**	O=C1N2[C@H](c3cc(CCCCn4nnc(CN(CC(OC)=O)C(=O)c5cc2ccc5)c4)ccc3)[C@H]1OC
**59b**	O=C1N2[C@H](c3cc(CCCCn4nnc(CN(CC(=O)[O-])C(=O)c5cc2ccc5)c4)ccc3)[C@H]1OC
**49d**	O1[C@H]2[C@H]([C@H]3N(c4c2c(ccc4)C(=O)N(Cc2nnn(CCCCc4cc3ccc4)c2)CC(OC)=O)C=O)CCC1

40 Top selling drugs

Lipitor	Fc1ccc(cc1)-c1n(CC[C@@H](O)C[C@@H](O)CC(=O)[O-])c(C(C)C)c(C(=O)Nc2ccccc2)c1-c1ccccc1
Nexium	S(=O)(Cc1ncc(C)c(OC)c1C)c1[nH]c2cc(OC)ccc2n1
Prevacid	S(=O)(Cc1nccc(OCC(F)(F)F)c1C)c1[nH]c2c(n1)cccc2
Flonase	S(C(=O)[C@]1(OC(=O)CC)[C@@]2([C@H]([C@@H]3C[C@H](F)C4=CC(=O)C=C[C@]4(C)[C@@]3(F)[C@@H](O)C2)C[C@H]1C)C)CF
Servent	Oc1ccc(cc1CO)[C@H](O)C[NH2+]CCCCCCOCCCCc1ccccc1
Singulair	Clc1cc2nc(ccc2cc1)\C=C\c1cc(ccc1)[C@H](SCC1(CC1)CC(=O)[O-])CCc1ccccc1C(O)(C)C

Effexor	O(C)c1ccc(cc1)[C@@H](C[NH+](C)C)C1(O)CCCCC1
Plavix	Clc1ccccc1[C@H]([NH+]1CCc2sccc2C1)C(OC)=O
Zocor	O1[C@@H](C[C@@H](O)CC1=O)CC[C@@H]1[C@@H]2C(=C[C@@H](C[C@@H]2OC(=O)C(CC)(C)C)C)C=C[C@@H]1C
Norvasc	Clc1ccccc1[C@@H]1C(C(OCC)=O)=C(NC(C)=C1C(OC)=O)COCC[NH3+]
Lexapro	Fc1ccc(cc1)[C@@]1(OCc2cc(ccc12)C#N)CCC[NH+](C)C
Seroquel	S1c2c(cccc2)C(=Nc2c1cccc2)N1CC[NH+](CC1)CCOCCO
Protonix	S(=O)(Cc1nccc(OC)c1OC)c1[nH]c2cc(OC(F)F)ccc2n1
Ambien	O=C(N(C)C)Cc1n2C=C(C=Cc2nc1-c1ccc(cc1)C)C
Actos	S1[C@H](Cc2ccc(OCCc3ncc(cc3)CC)cc2)C(=O)[N-]C1=O
Zoloft	Clc1cc(ccc1Cl)[C@@H]1CC[C@H]([NH2+]C)c2c1cccc2
Wellbutrin	Clc1cc(ccc1)C(=O)[C@H]([NH2+]C(C)(C)C)C
Avandia	S1[C@@H](Cc2ccc(OCCN(C)c3ncccc3)cc2)C(=O)[N-]C1=O
Risperdal	Fc1cc2onc(c2cc1)C1CC[NH+](CC1)CCC=1C(=O)N2C(=NC=1C)CCCC2
Zyprexa	S1C2=Nc3c(NC(N4CC[NH+](CC4)C)=C2C=C1C)cccc3
Topomax	S(OC[C@]12OC(O[C@H]1[C@@H]1OC(O[C@@H]1CO2)(C)C)(C)C)(=O)(=O)N
Toprol	O(C[C@H](O)C[NH2+]C(C)C)c1ccc(cc1)CCOC
Zetia	Fc1ccc(cc1)[C@@H](O)CC[C@@H]1[C@H](N(C1=O)c1ccc(F)cc1)c1ccc(O)cc1
Fosamax	P(=O)([O-])([O-])C(P(=O)([O-])[O-])(O)CCC[NH3+]
Ability	Clc1c(N2CC[NH+](CC2)CCCCOc2cc3NC(=O)CCc3cc2)cccc1Cl
Levaquin	Fc1cc2c3N(C=C(C(=O)[O-])C2=O)[C@H](COc3c1N1CC[NH+](CC1)C)C
Lamictal	Clc1c(cccc1Cl)-c1nnc(nc1N)N
Celebrex	S(=O)(=O)(N)c1ccc(-n2nc(cc2-c2ccc(cc2)C)C(F)(F)F)cc1
Benazepril	O=C1N(c2c(CC[C@@H]1[NH2+][C@@H](CCc1ccccc1)C(OCC)=O)cccc2)CC(=O)[O-]
Zyrtec	Clc1ccc(cc1)[C@H]([NH+]1CC[NH+](CC1)CCOCC(=O)[O-])c1ccccc1
Coreg	O(CC[NH2+]C[C@@H](O)COc1c2c3c([nH]c2ccc1)cccc3)c1ccccc1OC
Valtrex	O=C1N=C(Nc2n(cnc12)COCCOC(=O)[C@@H]([NH3+])C(C)C)N
Adderall	[NH3+][C@@H](Cc1ccccc1)C
Aciphex	S(=O)(Cc1nccc(OCCCOC)c1C)c1[nH]c2c(n1)cccc2
Cymbalta	s1cccc1[C@@H](Oc1c2c(ccc1)cccc2)CC[NH2+]C
Crestor	S(=O)(=O)(N(C)c1nc(-c2ccc(F)cc2)c(\C=C\[C@@H](O)C[C@@H](O)CC(=O)[O-])c(n1)C(C)C)C
Diovan	O=C(N([C@@H](C(C)C)C(=O)[O-])Cc1ccc(cc1)-c1ccccc1-c1n[n-]nn1)CCCC

Tricor	Clc1ccc(cc1)C(=O)c1ccc(OC(C(OC(C)C)=O)(C)C)cc1
Concerta	O(C(=O)[C@H]([C@H]1[NH2+]CCCC1)c1ccccc1)C
Imitrex	S(=O)(=O)(NC)Cc1cc2c([nH]cc2CC[NH+](C)C)cc1

60 randomly selected natural products

Taxol	O1[C@@H]2C[C@H](O)[C@@]3([C@H]([C@H](OC(=O)c4ccccc4)[C@]4(O)C[C@H](OC(=O)[C@H](O)[C@@H](NC(=O)c5ccccc5)c5ccccc5)C(=C([C@@H](OC(=O)C)C3=O)C4(C)C)C)[C@]2(OC(=O)C)C1)C
Actinonin	OC[C@H]1N(CCC1)C(=O)[C@@H](NC(=O)[C@H](CCCCC)CC(=O)N[O-])C(C)C
Discodermolide	O1[C@@H](C[C@H](O)\C=C/[C@@H]([C@H](O)[C@H](\C=C(/C[C@@H]([C@@H](O)[C@@H]([C@@H](OC(=O)N)[C@H](\C=C/C=C)C)C)C)\C)C)C)[C@H](C)[C@H](O)[C@@H](C)C1=O
Validamycin	O1[C@H](CO)[C@@H](O)[C@H](O)[C@@H](O)[C@@H]1O[C@H]1[C@H](O)[C@@H](O)[C@@H]([NH2+][C@H]2C=C(CO)[C@@H](O)[C@H](O)[C@H]2O)C[C@@H]1CO
Monensin	O1[C@@H]([C@H](C[C@@H](C)[C@]1(O)CO)C)[C@@H]1O[C@@H]([C@]2(O[C@H](CC2)[C@]2(O[C@]3(O[C@H]([C@H]([C@@H](OC)[C@@H](C(=O)[O-])C)C)[C@H](C)[C@@H](O)C3)CC2)C)CC)[C@H](C1)C
Calyculin A	P(O[C@H]1[C@H](O[C@@]2(O[C@@H](C\C=C\c3nc(oc3)[C@H](CCNC(=O)[C@@H](O)[C@@H](O)[C@@H]([NH+](C)C)COC)C)[C@@H](C)[C@H](O)C2)C1(C)C)[C@@H](OC)C[C@H](O)[C@@H]([C@H](O)[C@@H](\C=C(\C(=C\C=C\C(=C/C#N)\C)\C)/C)C)C)(=O)([O-])[O-]
Coformycin	O1[C@H](CO)[C@@H](O)[C@@H](O)[C@@H]1n1c2NC=[NH+]C[C@@H](O)c2nc1
Arglabin	O1[C@H]2[C@@H](CC[C@@]3(O[C@]34[C@@H]2C(=CC4)C)C)C(=C)C1=O
Mizoribine	O1[C@H](CO)[C@@H](O)[C@@H](O)[C@@H]1n1cnc(C(=O)N)c1O
Forskolon	O1[C@@]2(C)[C@@](O)([C@@]3([C@@H]([C@H](O)[C@@H]2OC(=O)C)C(CC[C@@H]3O)(C)C)C)C(=O)C[C@@]1(C=C)C
SQ 26180	S(=O)(=O)([O-])N1C[C@](OC)(NC(=O)C)C1=O
Cephamycin C	S1[C@H]2N(C(C(=O)[O-])=C(C1)COC(=O)N)C(=O)[C@@]2(OC)NC(=O)CCC[C@@H]([NH3+])C(=O)[O-]
Avermectin B1a	O1[C@@H]2C[C@@]3(O[C@H]([C@H](CC)C)[C@H](C=C3)C)O[C@H](C\C=C(/C)\[C@@H](O[C@@H]3O[C@@H](C)[C@H](O[C@@H]4O[C@@H](C)[C@H](O)[C@@H](OC)C4)[C@@H](OC)C3)[C@H](\C=C\C=C\3/CO[C@@H]4[C@H](O)C(=C[C@H]([C@]/34O)C1=O)C)C)C2

Adriamycin	O1[C@@H](C)[C@@H](O)[C@@H]([NH3+])C[C@@H]1O[C@@H]1c2c(C[C@](O)(C1)C(=O)CO)c(O)c1c(C(=O)c3c(cccc3OC)C1=O)c2O
Phorbol myristate acetate	O=C1[C@]2(O)[C@@H](C=C1C)[C@]1(O)[C@H]([C@H]3[C@@](OC(=O)C)([C@H](OC(=O)CCCCCCCCCCCCC)[C@H]1C)C3(C)C)C=C(C2)CO
Thienamycin	S(CC[NH3+])C=1C[C@H]2N(C=1C(=O)[O-])C(=O)[C@@H]2[C@H](O)C
Cyclosporin A	O=C1N(C)[C@@H]([C@H](O)[C@@H](C\C=C\C)C)C(=O)N[C@@H](CC)C(=O)N(CC(=O)N(C)[C@@H](CC(C)C)C(=O)N[C@@H](C(C)C)C(=O)N(C)[C@@H](CC(C)C)C(=O)N[C@@H](C)C(=O)N[C@H](C)C(=O)N(C)[C@@H](CC(C)C)C(=O)N(C)[C@@H](CC(C)C)C(=O)N(C)[C@H]1C(C)C)C
FK506	O1[C@H](/C(=C/[C@H]2C[C@@H](OC)[C@H](O)CC2)/C)[C@H](C)[C@@H](O)CC(=O)[C@@H](\C=C(\C[C@@H](C[C@H](OC)[C@H]2O[C@](O)([C@@H](C[C@@H]2OC)C)C(=O)C(=O)N2[C@@H](CCCC2)C1=O)C)/C)CC=C
Trapoxin B	O1C[C@H]1C(=O)CCCCC[C@@H]1NC(=O)[C@@H]2N(CCC2)C(=O)[C@@H](NC(=O)[C@@H](NC1=O)Cc1ccccc1)Cc1ccccc1
Vincristine	O(C)c1cc2N([C@@H]3[C@]4([C@H]5[NH+](CC=C[C@@]5(CC)[C@@H](OC(=O)C)[C@]3(O)C(OC)=O)CC4)c2cc1[C@@]1(c2[nH]c3c(c2CC[N@H+]2C[C@@](O)(C[C@@H](C1)C2)CC)cccc3)C(OC)=O)C=O
Colchicine	O(C)C1=CC=C2c3c(cc(OC)c(OC)c3OC)CC[C@H](NC(=O)C)C2=CC1=O
Trichostatin A	O=C([C@@H](\C=C(\C=C\C(=O)N[O-])/C)C)c1ccc(N(C)C)cc1
Fumagillin	O1[C@](C)([C@H]2[C@H](OC)[C@H](OC(=O)\C=C\C=C\C=C\C=C\C(=O)[O-])CC[C@@]23OC3)[C@H]1C\C=C(\C)/C
Staurosporine	O1[C@H]2n3c4c(c5c(CNC5=O)c5c6c(n(c45)[C@]1(C)[C@H](OC)[C@H]([NH2+]C)C2)cccc6)c1c3cccc1
Erythromycin A	O1[C@H](CC)[C@](O)(C)[C@H](O)[C@@H](C)C(=O)[C@@H](C[C@](O)(C)[C@H](O[C@@H]2O[C@@H](C[C@H]([NH+](C)C)[C@H]2O)C)[C@@H](C)[C@H](O[C@@H]2O[C@@H](C)[C@H](O)[C@](OC)(C2)C)[C@@H](C)C1=O)C
Streptomycin	O1[C@@H](CO)[C@H](O)[C@@H](O)[C@H]([NH2+]C)[C@@H]1O[C@@H]1[C@@](O)(C=O)[C@@H](O[C@H]1O[C@@H]1[C@@H](\[NH+]=C(\N)/N)[C@H](O)[C@@H](\[NH+]=C(\N)/N)[C@H](O)[C@H]1O)C
Penicillin G	S1[C@H]2N([C@@H](C(=O)[O-])C1(C)C)C(=O)[C@H]2NC(=O)Cc1ccccc1
Sperguallin	O=C(N[C@@H](O)C(=O)NCCCC[NH2+]CCC[NH3+])C[C@@H](O)CCCC\[NH+]=C(\N)/N
Rapamycin	O1[C@@H](CC(=O)[C@@H](\C=C(/C)\[C@@H](O)[C@@H](OC)C(=O)[C@@H](C[C@@H](\C=C\C=C\C=C(/C)\[C@@H](OC)C[C@H]2O[C@](O)([C@@H](CC2)C)C(=O)C(=O)N2[

	C@@H](CCCC2)C1=O)C)C)C)[C@@H](C[C@H]1C[C@@H](OC)[C@H](O)CC1)C
Echinocandin B	Oc1ccc(cc1)[C@H](O)[C@@H](O)[C@@H]1NC(=O)[C@H]2N(C[C@H](O)C2)C(=O)[C@@H](NC(=O)[C@@H](NC(=O)CCCCCCC\C=C/C\C=C/CCCCC)C[C@@H](O)[C@@H](O)NC(=O)[C@H]2N(C[C@H](C)[C@H]2O)C(=O)[C@@H](NC1=O)[C@H](O)C)[C@H](O)C
Ginkgolide B	O1[C@@H]2[C@@](O)([C@]34O[C@@H]5OC(=O)[C@H](O)[C@]56[C@]3([C@H](OC4=O)C[C@H]6C(C)(C)C)[C@H]2O)[C@H](C)C1=O
Vancomycin	Clc1c2Oc3cc4[C@@H](NC(=O)[C@@H](NC(=O)[C@H](NC(=O)[C@H]([NH2+]C)CC(C)C)[C@H](O)c(c1)cc2)CC(=O)N)C(=O)N[C@@H]1c2cc(-c5c(cc(O)cc5O)[C@H](NC(=O)[C@@H](NC1=O)[C@H](O)c1cc(Cl)c(Oc(c4)c3O[C@@H]3O[C@H](CO)[C@@H](O)[C@H](O)[C@H]3O[C@@H]3O[C@@H](C)[C@@H](O)[C@@]([NH3+])(C3)C)cc1)C(=O)[O-])c(O)cc2
Amphotericin B	O1[C@@H](C)[C@H](C)[C@H](O)[C@H](\C=C\C=C\C=C\C=C\C=C\C=C\C=C\[C@H](O[C@@H]2O[C@H](C)[C@@H](O)[C@H]([NH3+])[C@@H]2O)C[C@@H]2O[C@@](O)(C[C@H](O)[C@H]2C(=O)[O-])C[C@@H](O)C[C@@H](O)[C@H](O)CC[C@@H](O)C[C@@H](O)CC1=O)C
Radicicol	Clc1c2c(C(O[C@@H](C[C@H]3O[C@@H]3/C=C\C=C\C(=O)C2)C)=O)c(O)cc1O
Salicylihalamide A	O1[C@H](C[C@@H](O)[C@H](C\C=C\Cc2c(C1=O)c(O)ccc2)C)C\C=C\NC(=O)/C=C\C=C/CC
Telomestatin	S1C2=N[C@H](c3oc(c(n3)-c3oc(c(n3)-c3occ(n3)-c3occ(n3)-c3occ(n3)-c3occ(n3)-c3occ2n3)C)C)C1
Rifamycin B	O1c2c3c4c(c(O)c2C)c(O)c(NC(=O)/C(=C\C=C\[C@H](C)[C@H](O)[C@@H](C)[C@@H](O)[C@@H](C)[C@H](OC(=O)C)[C@H](C)[C@@H](OC)\C=C\O[C@]1(C)C3=O)/C)cc4OCC(=O)[O-]
Apoptolidin	O1[C@@H](C[C@H](OC)[C@@H](O)CC\C=C(\C=C\[C@H](O[C@@H]2O[C@@H](C)[C@H](OC)[C@@H](O)[C@@H]2O)[C@@H](C\C=C(\C=C(\C=C(/C)\C1=O)/C)/C)C)/C)[C@@H](O)[C@@]1(O[C@H](C[C@@H](O[C@@H]2O[C@@H](C)[C@H](O[C@@H]3O[C@H](C)[C@@H](O)[C@H](OC)C3)[C@@](O)(C2)C)COC)[C@H](C)[C@H](O)[C@H]1C)O
Midecamycin A1	O1[C@@H](C\C=C\C=C\[C@H](O)[C@@H](C[C@H](CC=O)[C@H](O[C@@H]2O[C@H](C)[C@@H](O[C@@H]3O[C@@H](C)[C@H](OC(=O)CC)[C@](O)(C3)C)[C@H]([NH+](C)C)[C@H]2O)[C@@H](OC)[C@H](OC(=O)CC)CC1=O)C)C
Zaragozic acid A	O1[C@@]2(C(=O)[O-])[C@](O)(C(O)=O)[C@H](O[C@]1(CCC([C@@H](OC(=O)C)[C@@H](Cc1ccccc1)C)=C)[C@H](O)[C@H]2OC(=O)\C=C\[C@H](C[C@H](CC)C)C)C(=O)[O-]
Talaromycin B	O1C[C@H](CO)[C@@H](O)C[C@]12OC[C@@H](CC2)CC

Spongistatin 1	ClC(\C=C\[C@@H](O)CC(C[C@H]1O[C@H]2[C@H](O)[C@@]3(O[C@H](CCC\C=C/[C@@H]4O[C@@]5(O[C@H](CC(=O)[C@@H](C)[C@@H](OC(=O)C)[C@H](C)C(C[C@@H]6O[C@@]7(O[C@@H](CC(O[C@@H]([C@@H]1O)[C@H]2C)=O)C[C@H](OC(=O)C)C7)C[C@@](O)(C6)C)=C)C[C@H](OC)C5)C[C@@H](O)C4)[C@H](C)[C@@H](O)C3)O)=C)=C
Brevetoxin B	O1[C@@H]2[C@H](O[C@@]3([C@H](O[C@@H]4[C@H](O[C@@H]5[C@H](O[C@H]6C[C@H]7O[C@@]8([C@H](O[C@@H]9[C@H](O[C@@H]%10[C@H](O[C@@]%11([C@H](O[C@@H](C[C@@H]%11O)CC(C=O)=C)C%10)C)C9)C=CC8)C[C@@]7(O[C@@]6(CC5)C)C)C)[C@@H](C4)C)C3)C2)C)C(=CC1=O)C
Quinine	O(C)c1cc2c(nccc2[C@@H](O)[C@H]2[N@@H+]3C[C@@H]([C@H](C2)CC3)C=C)cc1
Mycobactin S	O1C[C@H](N=C1c1ccccc1O)C(=O)N[C@@H](CCCCN([O-])C(=O)\C=C/CCCCCCCCCCCCCCC)C(O[C@H](CC(=O)N[C@H]1CCCCN([O-])C1=O)C)=O
Duocarmycin A	O(C)c1c(OC)c2[nH]c(cc2cc1OC)C(=O)N1C=2[C@]3([C@H](C3)C1)C1=C(N[C@](C(OC)=O)(C)C1=O)C(=O)C=2
Bleomycin A2	[S+](CCCNC(=O)c1nc(sc1)-c1nc(sc1)CCNC(=O)[C@@H](NC(=O)[C@H]([C@H](O)[C@H](NC(=O)[C@@H](NC(=O)c1nc(nc(N)c1C)[C@@H]([NH2+]C[C@H]([NH3+])C(=O)N)CC(=O)N)[C@@H](O[C@@H]1O[C@@H](CO)[C@@H](O)[C@H](O)[C@@H]1O[C@H]1O[C@H](CO)[C@@H](O)[C@H](OC(=O)N)[C@@H]1O)c1[nH]cnc1)C)C)[C@H](O)C)(C)C
Brefeldin A	O1[C@H](CCC\C=C\[C@H]2[C@@H](C[C@@H](O)C2)[C@H](O)\C=C\C1=O)C
Cytochalasin B	O1[C@@]23[C@@H]([C@H](C)C(=C)[C@@H](O)[C@@H]2\C=C\C[C@@H](CCC[C@@H](O)\C=C\C1=O)C)[C@@H](NC3=O)Cc1ccccc1
Epothilone A	s1cc(nc1C)\C=C(/C)\[C@H]1OC(=O)C[C@H](O)C(C)(C)C(=O)[C@H](C)[C@@H](O)[C@H](CCC[C@H]2O[C@H]2C1)C
Lactacystin	S(C(=O)[C@@]1(NC(=O)[C@H](C)[C@@H]1O)[C@@H](O)C(C)C)C[C@H](NC(=O)C)C(=O)[O-]
Calicheamicin γ1	Ic1c(C)c(C(S[C@@H]2[C@H](O[C@@H](ON[C@H]3[C@H](O)[C@@H](O[C@@H]4OC[C@H]([NH2+]CC)[C@@H](OC)C4)[C@@H](O[C@@H]3C)O[C@@H]3C=4\C(=C/CSSSC)\[C@@](O)(CC(=O)C=4NC(OC)=O)C#C\C=C/C#C3)C[C@@H]2O)C)=O)c(OC)c(OC)c1O[C@@H]1O[C@@H](C)[C@H](O)[C@@H](OC)[C@H]1O
Artemisinin	O1[C@@H]2O[C@@]3(OO[C@]24[C@@H](CC[C@H]([C@@H]4CC3)C)[C@@H](C)C1=O)C
Compactin	O1[C@@H](C[C@@H](O)CC1=O)CC[C@@H]1[C@@H]2C(C=C[C@@H]1C)=CCC[C@@H]2OC(=O)[C@H](CC)C
Lipstatin	O1[C@@H](C[C@@H](OC(=O)[C@@H](NC=O)CC(C)C)C\C=C/C\C=C/CCCCC)[C@H](CCC

	CCC)C1=O
Pseudomonic acid A	O1[C@@H](C[C@H]2CO[C@@H](C\C(=C\C(OCCCCCCCCC(=O)[O-])=O)\C)[C@H](O)[C@@H]2O)[C@@H]1[C@H]([C@@H](O)C)C
Daptomycin	O1[C@H](C)[C@H](NC(=O)[C@@H](NC(=O)[C@H](NC(=O)[C@@H](NC(=O)CCCCCCCCC)Cc2c3c([nH]c2)cccc3)CC(=O)N)CC(=O)[O-])C(=O)NCC(=O)N[C@@H](CCC[NH3+])C(=O)N[C@@H](CC(=O)[O-])C(=O)N[C@H](C)C(=O)N[C@@H](CC(=O)[O-])C(=O)NCC(=O)N[C@H](CO)C(=O)N[C@@H]([C@@H](CC(=O)[O-])C)C(=O)N[C@@H](CC(=O)c2ccccc2N)C1=O
Bestatin	O=C(N[C@@H](CC(C)C)C(=O)[O-])[C@@H](O)[C@H]([NH3+])Cc1ccccc1
Plaunotol	OC/C(=C\CC\C(=C\CO)\C)/CC\C=C(\CC\C=C(\C)/C)/C
Geldanamycin	O(C)C1=C2C[C@H](C[C@H](OC)[C@H](O)[C@H](\C=C(/C)\[C@H](OC(=O)N)[C@@H](OC)\C=C\C=C(/C)\C(=O)NC(=CC1=O)C2=O)C)C

36 macrocyclic-based compounds in clinical development as of April 2013

SB 1317	O1CC\C=C\C[NH+](Cc2cc(Nc3nc(-c4cc1ccc4)ccn3)ccc2)C
Pacritinib	O1Cc2cc(Nc3nc(-c4cc(COC\C=C\C1)ccc4)ccn3)ccc2OCC[NH+]1CCCC1
Cilengitide	O=C1N(C)[C@@H](C(C)C)C(=O)N[C@@H](CCC\[NH+]=C(\N)/N)C(=O)NCC(=O)N[C@@H](CC(=O)[O-])C(=O)N[C@@H]1Cc1ccccc1
Danoprevir	S(=O)([O-])(=NC(=O)[C@]12NC(=O)[C@H]3N(C[C@H](OC(=O)N4Cc5c(C4)cccc5F)C3)C(=O)[C@@H](NC(OC(C)(C)C)=O)CCCCC\C=C/[C@@H]1C2)C1CC1
Simeprevir	s1cc(nc1-c1nc2c(ccc(OC)c2C)c(O[C@H]2C[C@@H]3[C@@H](C2)C(=O)N[C@]2([C@H](\C=C/CCCCN(C)C3=O)C2)C(=O)N=S(=O)([O-])C2CC2)c1)C(C)C
Vaniprevir	S(=O)([O-])(=NC(=O)[C@@]1(NC(=O)[C@H]2N3C[C@H](OC(=O)N4Cc5c(CCCCC(COC(=O)N[C@@H](C(C)(C)C)C3=O)(C)C)cccc5C4)C2)C[C@H]1CC)C1CC1
SB 1578	O1Cc2cc(Nc3nc(-c4oc(COC\C=C\C1)cc4)ccn3)ccc2OCC[NH+]1CCCC1
MK 5172	S(=O)([O-])(=NC(=O)[C@@]1(NC(=O)[C@H]2N3C[C@H](Oc4nc5cc(OC)ccc5nc4CCCC[C@H]4[C@H](OC(=O)N[C@@H](C(C)(C)C)C3=O)C4)C2)C[C@H]1C=C)C1CC1
INO 4885	[Fe]1(Cl)n2c3ccc2C(=C2N=C(C=C2)C(=c2n1c(cc2)=C(C1=NC(C=C1)=C3c1[n+](cccc1)Cc1ccc

	(cc1)C(=O)[O-])c1[n+](cccc1)Cc1ccc(cc1)C(=O)[O-])c1[n+](cccc1)Cc1ccc(cc1)C(=O)[O-])c1[n+](cccc1)Cc1ccc(cc1)C(=O)[O-]
Exeporfinium	O(CCC[N+](C)(C)C)c1ccc(cc1)C=1C2=N\C(=C/c3[nH]c(cc3)\C(=C\3/N=C(C=c4[nH]c=1cc4)C=C/3)\c1ccc(OCCC[N+](C)(C)C)cc1)\C=C2
Latrunculin B	S1C[C@H](NC1=O)[C@@]1(O[C@@H]2CC[C@@H](\C=C/CC\C(=C/C(O[C@@H](C1)C2)=O)\C)C)O
KOS 1584	s1cc(nc1C)\C=C(/C)\[C@H]1OC(=O)C[C@H](O)C(C)(C)C(=O)[C@H](C)[C@@H](O)[C@H](\C=C/C\C(=C/C1)\C)C
Patupilone	s1cc(nc1C)\C=C(/C)\[C@H]1OC(=O)C[C@H](O)C(C)(C)C(=O)[C@H](C)[C@@H](O)[C@H](CCC[C@]2(O[C@H]2C1)C)C
Sagopilone	s1c2c(nc1C)cc(cc2)[C@H]1OC(=O)C[C@H](O)C(C)(C)C(=O)[C@H](CC=C)[C@@H](O)[C@H](CCC[C@]2(O[C@H]2C1)C)C
Lemuteporfin	O(C(=O)CCC=1/C=2/N=C(\C=C\3/N\C(=C/C4=N\C(=C/c5[nH]c(\C=2)c(CCC(OCCO)=O)c5C)\C(C=C)=C4C)\C2=CC=C(C(OC)=O)[C@H](C(OC)=O)[C@]2/3C)C=1C)CCO
Stannsoporfin	[Sn]1(Cl)(Cl)n2c3=CC4=N\C(=C/c5n1c(\C=C\1/N=C(C=c2c(C)c3CC)C(C)=C/1CCC(=O)[O-])c(CCC(=O)[O-])c5C)\C(CC)=C4C
JNJ 26483327	Brc1cc2Nc3ncnc4cc(OC)c(OCCCCC[NH+](Cc2cc1)C)cc34
OXT 008	O(CC(=O)NCC[NH+](C)C)c1cc2Cc3cc(OCC(=O)NCC[NH+](C)C)cc(Cc4cc(OCC(=O)NCC[NH+](C)C)cc(Cc5cc(OCC(=O)NCC[NH+](C)C)cc(Cc(c1)c2)c5)c4)c3
TMC 647055	S1(=O)([O-])=NC(=O)c2cc3n4c(-c5c(cc(OC)cc5)C=C(C4)C(=O)N(CCOCCN1C)C)c(c3cc2)C1CCCCC1
Myolimus	O1[C@@H](CC[C@@H](\C=C(/C)\[C@@H](O)[C@@H](OC)C(=O)[C@@H](C[C@@H](\C=C\C=C\C=C(/C)\[C@@H](OC)C[C@H]2O[C@](O)([C@@H](CC2)C)C(=O)C(=O)N2[C@@H](CCCC2)C1=O)C)C)C)[C@@H](C[C@H]1C[C@@H](OC)[C@H](O)CC1)C
Ridaforolimus	P(O[C@@H]1CC[C@H](C[C@H]1OC)C[C@@H](C)[C@H]1OC(=O)[C@H]2N(CCCC2)C(=O)C(=O)[C@@]2(O[C@H](C[C@H](OC)/C(=C/C=C/C=C\[C@H](C[C@@H](C)C(=O)[C@H](OC)[C@H](O)/C(=C/[C@@H](C)C(=O)C1)/C)C)/C)CC[C@H]2C)O)(=O)(C)C
Nepadutant	O1[C@H](CO)[C@H](O)[C@H](O)[C@@H](NC(=O)C)[C@@H]1NC(=O)C[C@@H]1NC(=O)[C@@H](NC(=O)[C@H]2NC(=O)[C@@H](NC(=O)[C@@H](NC(=O)[C@@H](NC1=O)CC(=O)NC2)Cc1c2c([nH]c1)cccc2)Cc1ccccc1)CC(C)C
Retasspimycin	Oc1cc2NC(=O)/C(=C/C=C\[C@H](OC)[C@@H](OC(=O)N)/C(=C/[C@H](C)[C@@H](O)[C@@H](OC)C[C@@H](Cc(c1NCC=C)c2O)C)/C)/C
Solithromycin	F[C@@]1(C)C(=O)[C@H](C)[C@@H](O[C@@H]2O[C@@H](C[C@H]([NH+](C)C)[C@H]2O)C)[C@](OC)(C[C@@H](C)C(=O)[C@@H]([C@H]2N(CCCCn3nnc(c3)-

	c3cc(N)ccc3)C(O[C@]2(C)[C@H](OC1=O)CC)=O)C)C
90Y-SMT 487	S1SC[C@H](NC(=O)[C@@H](NC(=O)[C@@H](NC(=O)[C@H](NC(=O)[C@@H](NC(=O)[C@H](C1)CC(=O)[C@H](NC(=O)C[NH+]1CC[NH+](CC[NH+](CC[NH+](CC1)CC(=O)[O-])CC(=O)[O-])CC(=O)[O-])Cc1ccccc1)Cc1ccc(O)cc1)Cc1c2c([nH]c1)cccc2)CCCC[NH3+])[C@H](O)C)C(=O)N[C@@H]([C@H](O)C)CO
L-Threoninamide	S1SC[C@H](NC(=O)[C@@H](NC(=O)[C@@H](NC(=O)[C@@H](NC(=O)CNC(=O)[C@@H](NC(=O)[C@@H](NC(=O)[C@@H](NC(=O)[C@@H](NC(=O)[C@@H](NC(=O)[C@@H](NC(=O)[C@@H](NC(=O)C)[C@H](CC)C)C1)C(C)C)Cc1c2c(n(c1)C)cccc2)CCC(=O)N)CC(=O)[O-])Cc1[nH]c2c(c1)cccc2)C)Cc1nc[nH]c1)CCCNC(=[NH2+])N)C(=O)N[C@@H]([C@H](O)C)C(=O)N
Bryostatin 1	O1[C@@H]2C\C(=C/C(OC)=O)\[C@H](OC(=O)\C=C\C=C\CCC)[C@]1(O)C(\C=C\[C@@H]1O[C@@H](C\C(\C1)=C\C(OC)=O)C[C@@]1(O[C@@H](C[C@H](OC(=O)C)C1(C)C)C[C@@H](O)CC(=O)C[C@@H](C2)[C@H](O)C)O)(C)C
Rifalazi	O1c2c3c4c(c(O)c2C)C(=O)C(NC(=O)/C(=C\C=C\[C@H](C)[C@H](O)[C@@H](C)[C@@H](O)[C@@H](C)[C@H](OC(=O)C)[C@H](C)[C@@H](OC)\C=C\O[C@]1(C)C3=O)/C)=C1Oc2c(N=C14)c(O)cc(N1CC[NH+](CC1)CC(C)C)c2
Lotilibcin	O1[C@@H](CC(=O)N[C@@H](CO)C(=O)N[C@H]([C@@H](O)C(=O)N)C(=O)N[C@@H](CO)C(=O)NCC(=O)N(C)[C@H](Cc2ccccc2)C(=O)N[C@@H](CC(C)C)C(=O)N[C@H](CCC[NH3+])C(=O)N[C@@H](CCC(=O)[O-])C(=O)N[C@H](CC(=O)N)C(=O)N[C@H](Cc2c3c([nH]c2)cccc3)C(=O)N[C@H](CCC[NH3+])C(=O)N(C)[C@@H](C(C)C)C1=O)CCCC(C)C
Somatoprim	O=C1N(CCCC(=O)NCCCC(=O)N[C@@H](Cc2ccccc2)C(=O)N[C@@H](Cc2c3c([nH]c2)cccc3)C(=O)N[C@H](Cc2c3c([nH]c2)cccc3)C(=O)N[C@@H](CCCC[NH3+])C(=O)N[C@@H]([C@H](O)C)C(=O)N[C@H]1Cc1ccccc1)CC(=O)N
Flopristin	F[C@@H]1C[C@@H](O)\C=C(\C=C\CNC(=O)\C=C\[C@@H](C)[C@H](OC(=O)[C@@H]2N(CCC2)C(=O)c2nc(oc2)C1)C(C)C)/C
Linopristin	O1[C@H](C)[C@H](NC(=O)c2ncccc2O)C(=O)N[C@H](CC)C(=O)N2[C@@H](CCC2)C(=O)N(C)[C@@H](Cc2ccc(N(C)C)cc2)C(=O)N2[C@@H](CC=C(C2)C[NH+]2CCOCC2)C(=O)N[C@@H](c2ccccc2)C1=O
Bremelanotide	O=C1N[C@@H](CCCNC(=[NH2+])N)C(=O)N[C@@H](Cc2c3c([nH]c2)cccc3)C(=O)N[C@@H](CCCCNC(=O)C[C@H](NC(=O)[C@@H](NC(=O)C)CCCC)C(=O)N[C@@H](Cc2nc[nH]c2)C(=O)N[C@@H]1Cc1ccccc1)CC(=O)[O-]
Pllitidepsin	O1[C@H](C)[C@H](NC(=O)[C@H](N(C(=O)[C@H]2N(CCC2)C(=O)C(=O)C)C)CC(C)C)C(=O)N

	[C@H]([C@H](CC)C)[C@@H](O)CC(O[C@@H](C(C)C)C(=O)[C@H](C)C(=O)N[C@@H](CC(C)C)C(=O)N2[C@@H](CCC2)C(=O)N(C)[C@@H](Cc2ccc(OC)cc2)C1=O)=O
SCY 635	S(CC[NH+](C)C)[C@H]1N(C)C(=O)[C@@H](NC(=O)[C@@H](N(C)C(=O)[C@@H](N(C)C(=O)[C@@H](N(C)C(=O)[C@@H](N(C)C(=O)[C@H](NC(=O)[C@@H](NC(=O)[C@@H](N(C)C(=O)[C@@H](NC(=O)[C@@H](N(C)C1=O)CC(O)(C)C)C(C)C)CC(C)C)C)CC(C)C)CC(C)C)C(C)C)[C@H](O)[C@@H](C\C=C\C)C)CC
Voclosporin	O=C1N(C)[C@@H]([C@H](O)[C@@H](C\C=C\C=C)C)C(=O)N[C@@H](CC)C(=O)N(CC(=O)N(C)[C@@H](CC(C)C)C(=O)N[C@@H](C(C)C)C(=O)N(C)[C@@H](CC(C)C)C(=O)N[C@@H](C)C(=O)N[C@H](C)C(=O)N(C)[C@@H](CC(C)C)C(=O)N(C)[C@@H](CC(C)C)C(=O)N(C)[C@H]1C(C)C)C

Table S8 PCA of the DOS library and three reference collections.

DOS library

Compound	PC1	PC2	PC3	Compound	PC1	PC2	PC3
**43a**	0.0091	-1.1711	-0.1920	**42b**	0.4975	-0.9678	-1.1280
**43b**	-0.0393	-1.1290	-0.2407	**47**	-0.3568	-0.9075	-0.3782
**57**	-0.0672	-1.0401	-0.3446	**49a**	-0.3455	-0.9155	-0.3596
**62**	0.3748	-1.9766	-0.1045	**49c**	-0.3558	-0.9569	-0.3071
**44b**	0.0620	-1.3739	-0.0456	**49b**	-0.3451	-0.9200	-0.3540
**50**	-0.4540	-0.4029	-0.8199	**49e**	-0.2855	-0.8797	-0.7422
**44a**	0.0620	-1.3494	-0.0761	**49f**	-0.2934	-0.8849	-0.7350
**48a**	-0.4512	-0.7830	-0.4607	**45a**	-0.2309	-0.4111	-0.4405
**48b**	-0.4314	-0.7563	-0.4953	**45b**	-0.2121	-0.3649	-0.4998
**54b**	-0.4083	-0.7230	-0.3782	**58**	-0.4156	-0.4771	-0.6090
**41a**	-0.1186	-0.9062	-1.0379	**59a**	-0.4088	-0.4363	-0.6605
**51**	-0.4163	-0.0740	-0.4036	**46a**	-0.2209	-0.3707	-0.4920
**40d**	-0.5141	0.2910	-0.8507	**46b**	-0.3598	-0.5181	-0.5914
**42a**	0.5079	-0.9770	-1.1170	**61**	0.1457	-1.2972	-0.7798
**54a**	-0.3749	-0.1599	-0.3401	**39**	-0.7059	0.4501	-0.2347
**53a**	-0.7820	0.5568	-0.2488	**56a**	-0.5968	0.0374	0.1508
**40a**	-0.3498	0.4188	-0.8733	**56b**	-0.5928	0.0924	0.1088
**40b**	-0.3536	0.4230	-0.8783	**52a**	-0.5601	-0.1208	0.3417
**53b**	0.2294	-0.3146	-0.2500	**52b**	-0.5573	-0.1000	0.3424
**63**	-0.5565	1.0815	-0.4219	**55**	-0.0124	-1.0426	-0.8797
**40c**	-0.2303	0.1621	-0.6843	**64b**	-0.5413	0.5409	-0.1107
**64a**	-0.5433	0.5696	-0.1390	**46c**	-0.3490	-0.5245	-0.5846
**41b**	-0.1005	-0.8742	-1.0797	**59b**	-0.3823	-0.3535	-0.7883
				**49d**	-0.2864	-0.8635	-0.3676

40 top selling drugs

Compound	PC1	PC2	PC3	Compound	PC1	PC2	PC3
Lipitor	-0.1201	-1.1366	-0.1384	Topomax	-0.6595	1.1507	0.3073
Nexium	-0.7001	-0.0395	-0.4658	Toprol	-0.9113	0.7963	0.1258
Prevacid	-0.7109	-0.1036	-0.5583	Zetia	-0.6645	-0.6676	-0.0077

Flonase	-0.4442	0.2021	1.0559	Fosamax	-1.0095	2.5938	-0.8789
Servent	-0.4425	0.1901	0.0719	Ability	-0.6537	-0.6162	-0.0576
Singulair	-0.2214	-1.8841	0.3426	Levaquin	-0.8161	0.4937	-0.5138
Effexor	-1.0124	0.4534	0.1247	Lamictal	-1.0066	0.3703	-0.9882
Plavix	-1.0102	-0.2148	0.1129	Celebrex	-0.7390	-0.3005	-0.6958
Zocor	-0.5959	0.2242	1.1679	Benazepril	-0.5496	0.0121	-0.1233
Norvasc	-0.5702	0.4333	0.0752	Zyrtec	-0.7237	0.0505	-0.2806
Lexapro	-0.9222	-0.2109	-0.1536	Coreg	-0.5468	-0.4730	-0.4009
Seroquel	-0.6925	-0.2579	-0.3520	Valtrex	-0.6419	1.2059	-0.9613
Protonix	-0.5866	0.1914	-0.6371	Adderall	-1.4445	0.7570	-0.1110
Ambien	-0.9839	-0.3020	-0.1197	Aciphex	-0.6227	-0.1165	-0.3677
Actos	-0.7803	-0.2168	-0.0837	Cymbalta	-0.9963	-0.5049	0.0077
Zoloft	-1.0996	-0.5463	0.2543	Crestor	-0.2914	0.4515	-0.3567
Wellbutrin	-1.1904	0.3163	0.2970	Diovan	-0.4910	-0.5620	-0.6217
Avandia	-0.7763	-0.0651	-0.3194	Tricor	-0.8245	-0.4526	0.5158
Risperdal	-0.7320	-0.5798	-0.0759	Concerta	-1.1426	0.5432	0.0749
Zyprexa	-0.9988	-0.0583	-0.3332	Imitrex	-0.9053	0.7930	-0.7033

60 randomly selected natural products

Compound	PC1	PC2	PC3	Compound.	PC1	PC2	PC3
Taxol	0.8311	-0.8848	0.7257	Ginkgolide B	-0.3468	1.1049	0.3572
Actinonin	-0.4806	1.0056	0.2180	Vancomycin	3.1707	-0.1983	-1.3445
Discodermolide	0.2802	0.9070	1.3943	Amphotericin B	1.3896	1.7353	1.3865
Validamycin	0.3783	3.2935	-0.4645	Radicicol	-0.7484	0.4423	0.3208
Monensin	0.3420	0.3217	1.8668	Salicylihalamide A	-0.4386	0.0662	0.6639
Calyculin A	1.5435	0.5773	1.2399	Telomestatin	0.1160	-2.2906	-1.5746
Coformycin	-0.6994	1.7178	-0.9424	Rifamycin B	0.5503	0.0751	0.9047
Arglabin	-1.1346	0.5400	0.5191	Apoptolidin	2.0916	0.6639	2.8634
Mizoribine	-0.7121	1.9657	-0.9068	Midecamycin A1	0.8213	0.8814	1.8929
Forskolon	-0.5313	0.8477	0.8427	Zaragozic acid A	0.5775	0.7621	0.6660
SQ 26180	-1.0253	1.8328	-0.5038	Talaromycin B	-1.0418	1.1197	0.4432
Cephamycin C	-0.2328	2.0052	-0.9599	Spongistatin 1	2.0228	0.0165	2.8118
Avermectin B1a	0.9331	-0.2458	2.4744	Brevetoxin B	0.9341	-1.0532	2.6432
Adriamycin	0.1155	0.8095	-0.3578	Quinine	-0.8258	0.1056	-0.1228
Phorbol	0.1503	-0.2733	1.5667	Mycobactin S	0.9781	-0.9428	1.0360

myristate acetate							
Thienamycin	-0.9081	1.5742	-0.4528	Duocarmycin A	-0.1524	0.0629	-0.3472
Cyclosporin A	1.7663	-0.2614	1.4055	Bleomycin A2	3.6011	2.2347	-2.5483
FK506	0.6715	0.1566	1.9303	Brefeldin A	-0.9617	0.9447	0.6002
Trapoxin B	0.0130	-0.3717	-0.0267	Cytochalasin B	-0.3844	-0.0359	0.6803
Vincristine	0.6382	-0.8764	-0.0658	Epothilone A	-0.4055	0.2173	0.9594
Colchicine	-0.5918	0.3279	0.2009	Lactacystin	-0.4632	1.8167	-0.1653
Trichostatin A	-0.8729	0.5260	-0.0473	Calicheamicin γ1	2.5715	-0.1393	1.7419
Fumagillin	-0.3240	0.2480	1.0273	Artemisinin	-0.9707	0.4497	0.8504
Staurosporine	-0.4751	-1.3972	-0.4768	Compactin	-0.6438	0.3940	1.0500
Erythromycin A	0.6074	1.1479	1.6916	Lipstatin	-0.0848	-0.1680	1.3777
Streptomycin	0.6368	3.4811	-1.4905	Pseudomonic acid A	-0.0315	1.0648	0.9476
Penicillin G	-0.7869	0.6313	-0.2746	Daptomycin	3.8505	1.7269	-2.1933
Sperguallin	-0.1369	2.4384	-1.3273	Bestatin	-0.7323	1.1700	-0.3704
Rapamycin	0.9181	-0.0220	2.1745	Plaunotol	-0.8016	0.5395	0.8079
Echinocandin B	1.9912	1.0701	0.1340	Geldanamycin	-0.0108	0.8802	0.6687

36 macrocyclid-based compounds in clinical evelopment as of April 2013

Compound	PC1	PC2	PC3	Compound	PC1	PC2	PC3
SB 1317	-0.7908	-0.7667	-0.5187	TMC 647055	-0.2292	-1.2875	-0.1062
Pacritinib	-0.4558	-1.0246	-0.3214	Myolimus	0.8545	-0.3575	2.4304
Cilengitide	0.1633	1.3851	-1.2335	Ridaforolimus	1.0884	-0.3365	2.6382
Danoprevir	0.2727	-0.6601	0.3868	Nepadutant	1.5494	0.6757	-1.2063
Simeprevir	0.3000	-1.3348	0.2027	Retasspimycin	0.0979	0.6588	0.3099
Vaniprevir	0.3109	-0.9054	0.5867	Solithromycin	0.7276	-0.3033	0.5836
SB 1578	-0.4578	-0.8699	-0.3416	90Y-SMT 487	2.8203	0.9123	-2.1838
MK 5172	0.4224	-0.6795	0.0470	L-Threoninamide	3.6769	0.4259	-2.7214
INO 4885	1.3916	-4.5966	-2.3165	Bryostatin 1	1.0618	0.1207	1.9507
Exeporfinium	0.1385	-2.1630	-0.2480	Rifalazi	1.0035	-1.0259	0.8470
Latrunculin B	-0.6163	0.5114	0.6029	Lotilibcin	3.4787	1.1750	-1.7592
KOS 1584	-0.4829	0.1639	0.9756	Somatoprim	1.7467	-1.2201	-1.5396
Patupilone	-0.3774	0.1183	1.0324	Flopristin	-0.3162	0.1844	0.4619
Sagopilone	-0.2594	-0.3306	0.8508	Linopristin	0.9880	-0.7991	-0.5613
Lemuteporfin	0.5593	-0.5154	0.3391	Bremelanotide	1.6969	0.1411	-1.9854

Stannsoporfin	-0.0678	-1.0003	-0.1969	Pllitidepsin	1.6091	-0.4490	1.2437
JNJ 26483327	-0.6378	-0.7373	-0.3539	SCY 635	2.1725	-0.0858	1.3214
OXT 008	1.1621	-0.5977	-1.4664	Voclosporin	1.8108	-0.3844	1.4797

a) PC1 *versus* PC2

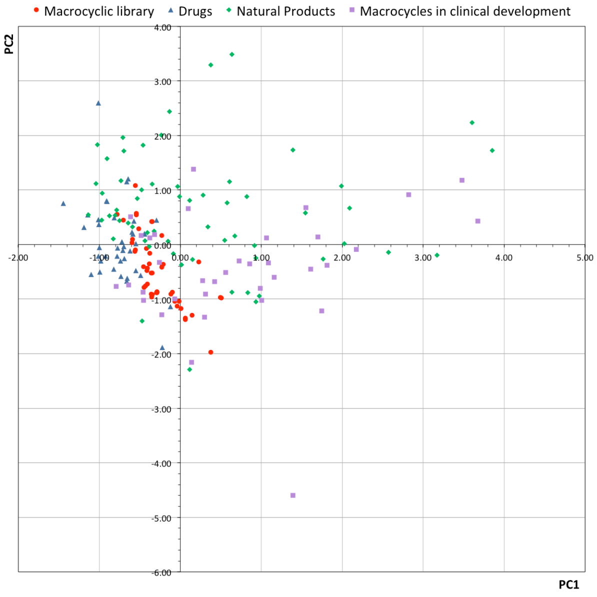

b) PC1 *versus* PC3

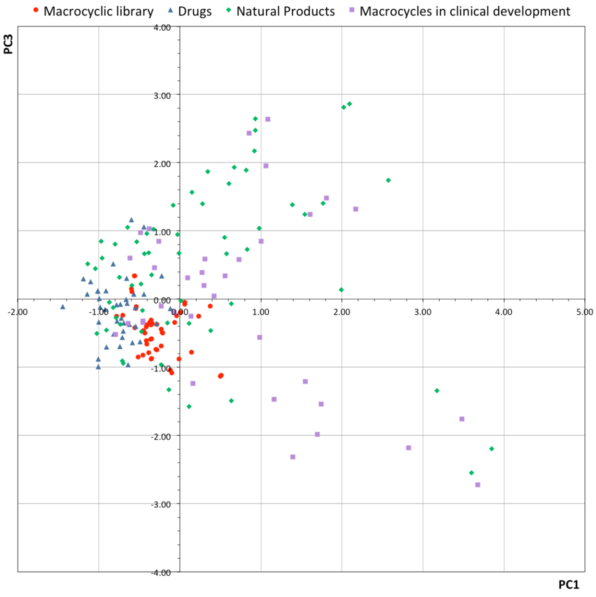

c) PC2 *versus* PC3

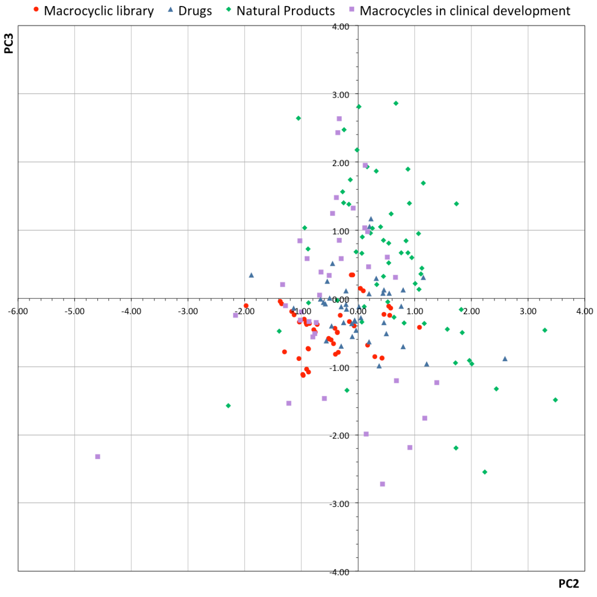

Figure S9. Comparative PCA plot of DOS library with other molecular collections

a) PC1 *versus* PC2; b) PC1 *versus* PC3; c) PC2 *versus* PC3. Macrocyclic DOS library (red dots), 40 synthetic drugs (blue triangle), 60 natural products (green rhombus) and 36 macrocyclic-based compounds in clinical development (purple square).

Table S9 Standard deviation and contribution of each principal component of variance.

PC#	Deviation[a]	Condition[b]	Proportion of variance	% Variance[c]
PC1	3.234	1.000	61.514	61.514
PC2	1.573	4.229	14.546	76.060
PC3	1.349	5.744	10.710	86.770
PC4	0.964	11.243	5.471	92.241
PC5	0.602	28.888	2.130	94.371
PC6	0.494	42.864	1.435	95.806

[a] The standard deviation of the data along the principal component vector.

[b] Condition number of the covariance matrix if the principal component list were terminated at that row.

[c] Percentage of the variance retained if the component list were truncated at that row.

Table S10 Component loadings for PCA of DOS library with three reference sets.

Descriptors	PC1	PC2	PC3	PC4	PC5	PC6
ASA_H	0.0004	-0.0008	0.0010	-0.0008	0.0008	-0.0024
ASA_P	0.0007	0.0011	-0.0014	0.0020	-0.0008	0.0070
a_acc	0.0194	0.0162	0.0180	0.0578	0.0732	0.1215
a_aro	0.0017	-0.0317	-0.0419	0.0141	-0.0195	0.0578
a_don	0.0228	0.0406	-0.0341	0.0105	0.0510	0.1599
a_nN	0.0169	-0.0133	-0.0974	-0.0588	0.2175	-0.1490
a_nO	0.0176	0.0188	0.0235	0.0411	-0.0380	0.0492
b_rotN	0.0103	0.0065	-0.0071	-0.0652	-0.1651	0.0131
chiral	0.0121	0.0201	0.0487	0.0554	-0.0119	-0.0744
KierFlex	0.0141	0.0056	0.0185	-0.0415	0.0226	0.0044
logS	-0.0239	0.0870	-0.0203	0.0700	0.0934	-0.1242
mr	0.0119	-0.0122	0.0031	-0.0097	0.0198	-0.0325
rings	0.0196	-0.1263	-0.0361	0.3084	-0.2407	-0.3152
SlogP	-0.0088	-0.0815	0.0787	-0.0123	0.1021	0.2989
TPSA	0.0008	0.0009	-0.0010	0.0003	0.0007	0.0011
vol	0.0003	-0.0002	0.0002	-0.0003	0.0007	-0.0010
Weight	0.0003	-0.0002	0.0001	-0.0001	0.0004	-0.0006

Top contributing parameters to each principal component are marked in grey, the darker grey, the more contribution in each column. The values were normalised automatically by the MOE software.

General experimental methods

All non-aqueous reactions were carried out under nitrogen or argon with dry and freshly distilled solvents using oven-dried glassware unless otherwise stated. Room temperature (rt) refers to ambient temperature. A temperature of 0 °C was maintained using an ice-water bath. A temperature of -78 °C was maintained using an acetone-dry ice bath. Reactions under microwave heating were performed in sealed vials using a CEM Discover SP microwave reactor.

All reagents and solvents were used as obtained from commercial sources unless otherwise stated. CH_3ONa (0.05 M) was prepared by dissolving sodium methoxide in dry methanol under argon. LiOH (0.5 M) was prepared by dissolving lithium hydroxide in water. Tetrahydrofuran was dried over Na wire and distilled from a mixture of calcium hydride and lithium aluminium hydride with triphenylmethane as indicator. Dichloromethane, methanol, acetonitrile, and ethyl acetate were distilled from calcium hydride. Diethyl ether was distilled from a combination of calcium hydride and lithium aluminium hydride. Petroleum ether refers to the distilled fraction between 40-60 °C. Pd/C refers to palladium on activated charcoal (10% Pd basis).

Yields refer to chromatographically and spectroscopically pure compounds unless otherwise stated. Reactions were monitored using thin layer chromatography (TLC) or low resolution mass spectra (LRMS). Yields around 100% were recorded as quantitative (quant.).

Thin layer chromatography (TLC) was carried out on glass plates pre-coated with Merck silica gel 60 F254, visualised by the quenching of UV fluorescence (λ_{max} = 254 nm) or by staining with potassium permanganate. Retention factors (R_f) are quoted to 0.01.

Low resolution mass spectra (LRMS) were recorded using liquid chromatography and mass spectroscopy (LCMS). Only molecular ions are reported. ESI refers to the electrospray ionisation technique.
LCMS System 1: Agilent 1200 series LC with an ESCi Multi-Mode Ionisation Waters ZQ spectrometer using MassLynx 4.0 software; LC system: solvent A: 10 mM NH_4OAc + 0.1% HCOOH in water; solvent B: 95% acetonitrile + 5% water + 0.05% HCOOH; column: Supelcosil^{TM} ABZ+PLUS column (33 mm × 4.6 mm, 3 µm); gradient: 5-100% B over 4 min at flow rate of 1 mL·min^{-1}; detector: DAD 190 nm - 600 nm, interval 2.0 nm. LCMS System 2: Waters ACQUITY H-Class UPLC with an ESCi Multi-Mode Ionisation Waters SQ Detector 2 spectrometer using MassLynx 4.1 software; LC system: solvent A: 2mM NH_4OAc in water/acetonitrile (95:5); solvent B: acetonitrile; solvent C: 2% formic acid; column: ACQUITY UPLC CSH C18 (2.1 mm × 50 mm, 1.7

μm, 130 Å) at 40 °C; gradient: 5-95% B with constant 5% C over 1 min at flow rate of 0.6 mL·min^{-1}; detector: PDA eλ Detector 220 nm - 800 nm, interval 1.2 nm.

Flash column chromatography (FCC) was carried out using either slurry-packed Merck 9385 Kieselgel 60 silca gel (230 - 400 mesh) under a positive pressure of nitrogen, or RediSep Rf normal phase silica column on the Combi*Flash* Rf flash chromatography system.

Fluorous-solid phase extraction (F-SPE) was carried out using dry-packed FluoroFlash^{TM} Silica (40 μm, 60 Å) under a positive pressure of nitrogen. 5% of sample mixture by weight was loaded onto the column using DMSO (1 mL per 1 g fluorous silica) as the loading solvent. MeOH/H_2O (80:20, v/v) was used as the fluorophobic wash to elute the nonfluorous organic compounds. The fluorous compounds were then eluted using a fluorophilic second wash such as MeOH. Fluorous silica was regenerated by washing thoroughly with acetone.

Analytical high performace liguid chromatography (HPLC) was run on an Agilent 1260 Infinity machine, using a Supelcosil^{TM} ABZ+PLUS column (150 mm × 4.6 mm, 3 μm) with a linear gradient system (solvent A: 0.05% (v/v) TFA in water, solvent B: 0.05% (v/v) TFA in acetonitrile) over 15 min at a flow rate of 1 mL·min^{-1}, and UV detection (λ_{max} = 220 nm and 254 nm). Retention times (t_r) are reported to the nearest 0.01 min. Peak area percentages are calculated for the UV absorbance at 220 nm.

Preparative high performace liquid chromatography (HPLC) was run on an Agilent 1260 Infinity machine, using a Supelcosil^{TM} ABZ+PLUS column (250 mm × 21.2 mm, 5 μm) with a linear gradient system (solvent A: 0.1% (v/v) TFA in water, solvent B: 0.05% (v/v) TFA in acetonitrile) over 20 min at a flow rate of 20 mL·min^{-1}, visualised by UV absorbance (λ_{max} = 254 nm).

Melting points (m.p.) were obtained using a Büchi Melting Point B-545 melting point apparatus and are uncorrected.

Optical rotations were recorded on a Perkin Elmer 343 polarimeter. $[\alpha]^T_D$ values are reported in 10^{-1}·deg·cm^2·g^{-1} at 589 nm, concentration (c) is given in g·dL^{-1}.

Infrared (IR) spectra were recorded on a Perkin-Elmer Spectrum One (FT-IR) spectrometer with internal referencing as neat films. Selected absorption maxima (ν_{max}) are reported in wavenumbers (cm^{-1}) and the following abbreviations are used: w, weak; m, medium; s, strong; br, broad.

Magnetic resonance spectra (NMR) were recorded using an internal deuterium lock at ambient probe temperatures (unless otherwise stated) on Bruker DPX-400, Bruker Avance DRX-400, Bruker Avance 500 BB-ATM and Bruker Avance 500 Cryo Ultrashield spectrometers. ^{1}H NMR and ^{13}C NMR spectra assignments are supported by DEPT-135 editing, COSY (2D, ^{1}H-^{1}H correlations), HMQC or HSQC (2D, one bond ^{1}H-^{13}C correlations), HMBC (2D, multi-bonds ^{1}H-^{13}C correlations), NOESY (2D, nulear Overhauser effect) and ROESY (2D, through space correlations via spin-spin relaxation), spectra where necessary, or by analogy to fully interpreted spectra of related compounds.

In proton magnetic resonance spectra (^{1}H NMR): chemical shifts (δ) are quoted in ppm to the nearest 0.01 ppm and are referenced to the residual non-deuterated solvent peak (chloroform-*d*: 7.26, dimethyl sulfoxide-d_6: 2.50, methanol-d_4: 4.87); coupling constants (J) are reported in Hertz to the nearest 0.1 Hz. Data are reported as follows: chemical shift, multiplicity [br, broad; s, singlet; d, doublet; t, triplet; q, quartet; quint, quintet; m, multiplet; or as a combination of these (e.g. dd, dt, etc.)], coupling constant(s), integration and assignment. Diastereotopic protons are assigned as H-a and H-b, where the H-a indicates the lower field proton.

In carbon magnetic resonance spectra (^{13}C NMR): chemical shifts (δ) are quoted in ppm to the nearest 0.1 ppm and are referenced to the deuterated solvent (chloroform-*d*: 77.2, dimethyl sulfoxide-d_6: 39.5, methanol-d_4: 49.0); coupling constants (J) are reported in Hertz to the nearest 0.1 Hz. Data are reported as follows: chemical shift, multiplicity [t, triplet], coupling constant and assignment.

High resolution mass spectra (HRMS) were obtained with a Micromass Q-TOF mass spectrometer or a Waters LCT Premier Time of Flight mass spectrometer. Reported mass values are within the error limits of ± 5 ppm mass units. Only molecular ions are reported. ESI refers to the electrospray ionisation technique.

Compounds were named using ChemBio Draw 14.0 and may not be in agreement with IUPAC guidelines.

General procedures

GSP-1 Synthesis of azido compounds via diazonium salt

The aromatic amine (1.0 eq) was suspended or dissolved in water. Concentrated sulfuric acid (4 mL) and $NaNO_2$ (1.05 eq) were added at 0 °C. The reaction mixture was stirred at 0 °C for 20 min and a solution of NaN_3 (1.05 eq) dissolved in water at 0 °C added dropwise. Then EtOAc was added and the two layers were separated. The aqueous layer was extracted with EtOAc. The combined organic layers were washed with water and brine, dried over $MgSO_4$, filtered and concentrated.

GSP-2 Synthesis of azido compounds via S_N2 reactions

Reactant (1.0 eq) was dissolved in CH_2Cl_2 (20 mM); triethylamine (1.2 eq) and 4-dimethylaminopyridine (0.05 eq) were added. 4-Toluenesulfonyl chloride (1.2 eq) was added at 0 °C. After stirring at rt overnight, the mixture was washed with 5% aq. HCl, sat. aq. $NaHCO_3$ and brine, dried over Na_2SO_4, filtered and concentrated under reduced pressure. The residue was either purified by FCC or used without further purification. The (crude) product was dissolved in anhydrous DMF (30 mL) and NaN_3 (1.2 eq) added. After stirring at rt overnight, the mixture was partitioned between Et_2O and water. The organic phase was washed with sat. aq. $NaHCO_3$ and brine, dried over $MgSO_4$, filtered and concentrated under reduced pressure. The crude product was purified by FCC.

GSP-3 Synthesis of the aza-ylides

Procedure a) the azido building block (1.0 eq) was dissolved in THF with 4 Å molecular sieves. PBu_3 (1.0 eq) was added. The reaction mixture was stirred at room temperature until TLC indicated complete turnover (typically 1-2 h). If starting material was not completely consumed, additional PBu_3 was added in portions (typically 0.05 eq) until TLC indicated complete turnover. This aza-ylide was used as a solution.

Procedure b) the azido building block (1.0 eq) and PPh_3 (1.0 eq) were dissolved in THF with 4 Å molecular sieves. The reaction mixture was stirred at room temperature until TLC indicated complete turnover (typically 3-4 h). If starting material was not completely consumed, additional PPh_3 was added in portions (typically 0.05 eq) until TLC indicated complete turnover. This aza-ylide was used as a solution.

GSP-4 Synthesis of the ureas

The azido amine hydrochloride (1.1-1.3 eq) was dissolved or suspended in THF and DIPEA (1.2-4.0 eq) added. The mixture was added to the solution of the aza-ylide. And then argon atmosphere was exchanged to CO_2. The mixture was stirred at room temperature or at elevated temperature overnight. The solvent was removed under a stream of nitrogen and the residue purified by F-SPE where applicable and/ or FCC.

GSP-5 Synthesis of the amides

The carboxylic acid (1.6 eq) was dissolved in CH_2Cl_2. Oxalyl chloride (2.4-3.6 eq) and a few drops of DMF were added sequentially. The reaction solution was stirred at room temperature until TLC indicated complete turnover (typically 2-3 h). The solvent was removed under reduced pressure and the residue dissolved in CH_2Cl_2 or THF and was added to the solution of the aza-ylide. The mixture was stirred at room temperature overnight and quenched by the addition of MeOH. The solvent was removed under a stream of nitrogen and the residue dissolved in EtOAc. The organic phase was washed with 5% aq. HCl, sat. aq. $NaHCO_3$ and brine, dried over $MgSO_4$, filtered and concentrated under reduced pressure. The crude product was purified by FCC.

GSP-6 Cu-catalysed azide alkyne cycloadditions

The linear precursor (1.0 eq) was dissolved in THF at a concentration of 1 mM and DIPEA (3.0 eq) added. Argon was bubbled through the solution for 20 min. CuI (2.0 eq) was added and the reaction mixture refluxed until analytical HPLC indicated complete turnover. The solvent was removed under a stream of nitrogen and the residue purified by FCC and/ or preparative HPLC if necessary.

GSP-7 Ru-catalysed azide-alkyne cycloadditions

The linear precursor (1.0 eq) was dissolved in THF at a concentration of 1 mM. Argon was bubbled through the solution for 20 min. $[Cp^*RuCl]_4$ (0.1 eq) was added and the reaction mixture refluxed until analytical HPLC indicated complete turnover. The reaction solvent was removed under a stream of nitrogen and the residue purified by FCC and/ or preparative HPLC.

GSP-8 Transesterification

The fluorous-tagged macrocycle (1.0 eq) was dissolved in MeOH and 0.05 M MeONa in MeOH (1.0 eq) added. The reaction mixture was stirred at room temperature until TLC or LCMS indicated

complete turnover. HCl in dioxane (4 M; 1.2 eq) was added to neutralise the reaction. The solvent was removed under a stream of nitrogen and the residue purified by FCC and/ or preparative HPLC if necessary.

GSP-9 Hydrolysis

The fluorous-tagged macrocycle (1.0 eq) was dissolved in THF (20 mM) and LiOH (5.0 eq) in water (half of the THF volume) was added at 0 °C. After stirring at room temperature until TLC or LCMS indicated complete turnover (typically 2 h), the reaction was neutralised by the addition of HCl in dioxane (4 M) and stirred for another 10 min. The solvent was removed under a stream of nitrogen and the residue purified using preparative HPLC.

Synthetic procedures and data

In the *build* stage

3,3,4,4,5,5,6,6,7,7,8,8,9,9,10,10,10-Heptadecafluorodecyl *N*-(3-azidobenzoyl)-*N*-(prop-2-yn-1-yl)glycinate (2)

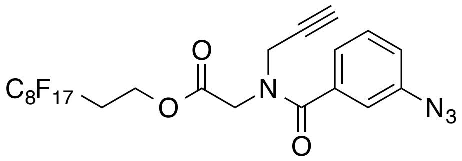

2 was synthesised according to our previous paper.[1]

In the *couple* stage

3,3,4,4,5,5,6,6,7,7,8,8,9,9,10,10,10-Heptadecafluorodecyl *N*-(prop-2-yn-1-yl)-*N*-(3-(undec-10-enamido)benzoyl)glycinate (4)

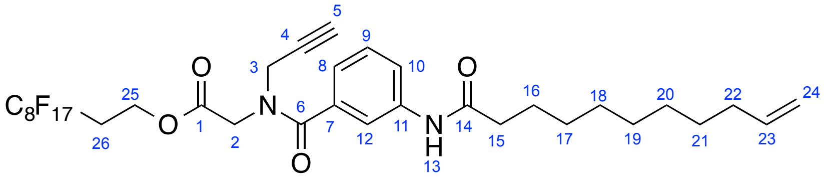

2 (73 mg, 0.10 mmol, 1.0 eq) was reacted according to GSP-3a using PBu_3 (32 µL, 0.13 mmol, 1.2 eq). 10-undecenoic acid (29 mg, 0.16 mmol, 1.5 eq) was reacted according to GSP-5 using oxalyl chloride (136 µL, 1.61 mmol, 2.4 eq). The resulting ylide was reacted with the acyl chloride (**4a**) in THF. The reaction mixture was stirred at rt overnight. Additional acyl chloride which was prepared from 10-undecenoic acid (29 mg, 0.16 mmol, 1.5 eq) according to GSP-5 using oxalyl chloride (136 µL, 1.61 mmol, 2.4 eq) was added. The reaction mixture was stirred at rt overnight. After F-SPE and then FCC (petroleum ether/EtOAc 3:1), **4** (65 mg, 0.077 mmol, 77%) was obtained as a white solid.

TLC R_f = 0.14 (petroleum ether/EtOAc 3:1); **HPLC** t_r = 13.63 min (60-100% B), peak area 97%; **IR** ν_{max} (neat)/cm^{-1} = 1752 m (C=O), 1665 m, 1649 m, 1553 m, 1418 w, 1196 s (C-F), 1144 s (C-F); **^{1}H**

NMR (500 MHz, DMSO-d_6, 90 °C) δ = 9.71 (s, 1H; H-13), 7.73 (t, J = 1.9 Hz, 1H; H-12), 7.68-7.57 (m, 1H; H-10), 7.35 (t, J = 7.8 Hz, 1H; H-9), 7.06 (d, J = 7.6 Hz, 1H; H-8), 5.80 (ddt, J = 17.0, 10.2, 6.6 Hz, 1H; H-23), 4.99 (dq, J = 17.2, 1.8 Hz, 1H; H-24_{trans}), 4.93 (ddt, J = 10.2, 2.4, 1.3 Hz, 1H; H-24_{cis}), 4.43 (t, J = 6.1 Hz, 2H; H-25), 4.22 (s, 4H; H-2 and H-3), 3.13 (t, J = 2.4 Hz, 1H; H-5), 2.67 (tt, J = 19.2, 6.3 Hz, 2H; H-26), 2.31 (t, J = 7.4 Hz, 2H; H-15), 2.02 (tq, J = 6.8, 1.4 Hz, 2H; H-22), 1.62 (quint, J = 7.2 Hz, 2H; H-16), 1.47-1.19 (m, 10H; H-21, H-17, H-18, H-19 and H-20); **^{13}C NMR** (125 MHz, DMSO-d_6, 90 °C) due to interconversion, C-2 and C-3 are missing, δ = 171.4 (C-14), 170.3 (C-6), 168.1 (C-1), 139.3 (C-11), 138.5 (C-23), 135.0 (C-7), 128.5 (C-9), 120.7 (C-8), 120.6 (C-10), 117.4 (C-12), 114.0 (C-24), 78.2 (C-4), 74.8 (C-5), 56.6 (C-25), 36.2 (C-15), 32.7 (C-22), 29.8 (t, J = 21.4 Hz; C-26), 28.3 × 2 (two carbons from C-17, C-18, C-19, C-20 and C-21), 28.1 (C-17, C-18, C-19, C-20 or C-21), 28.0 (C-17, C-18, C-19, C-20 or C-21), 27.9 (C-17, C-18, C-19, C-20 or C-21), 24.7 (C-16); **HRMS** (ESI+) m/z = 845.2212 $[M+H]^+$ found, $C_{33}H_{34}F_{17}N_2O_4^+$ required 845.2242.

3-(3-Azidophenyl)propanoic acid (5a1)

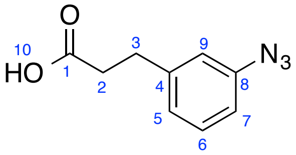

3-(3-Aminophenyl) propionic acid (1.56 g, 10.0 mmol, 1.0 eq) was reacted according to GSP-1. After aqueous workup, **5a1** (1.63 g, 8.53 mmol, 85%) was obtained as an off-white solid.

TLC R_f = 0.63 (CH_2Cl_2/MeOH 10:1); **mp** 72-76 °C (CH_2Cl_2); **IR** ν_{max} (neat)/cm^{-1} = 2929 brs (OH), 2112 s (N_3), 1693 s (C=O), 1606 m, 1581 m, 1489 m, 1434 m, 1414 m, 1288 s; **^{1}H NMR** (400 MHz, DMSO-d_6) δ = 12.14 (brs, 1H; H-10), 7.31 (t, J = 7.8 Hz, 1H; H-6), 7.06 (d, J = 7.7 Hz, 1H; H-5), 6.99 (t, J = 1.9 Hz, 1H; H-9), 6.94 (dd, J = 7.9, 2.3 Hz, 1H; H-7), 2.82 (t, J = 7.6 Hz, 2H; H-3), 2.54 (t, J = 7.6 Hz, 2H; H-2); **^{13}C NMR** (100 MHz, DMSO-d_6) δ = 173.6 (C-1), 143.2 (C-4), 139.2 (C-8), 129.9 (C-6), 125.2 (C-5), 118.9 (C-9), 116.8 (C-7), 34.9 (C-2), 30.1 (C-3); **HRMS** (ESI+) m/z = 190.0621 $[M+H]^+$ found, $C_9H_8N_3O_2^+$ required 190.0622.

3,3,4,4,5,5,6,6,7,7,8,8,9,9,10,10,10-Heptadecafluorodecyl *N*-(3-(3-(3-azidophenyl) propanamido)benzoyl)-*N*-(prop-2-yn-1-yl)glycinate (5)

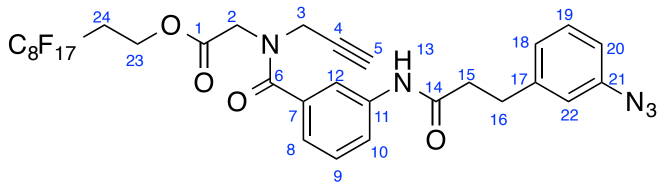

2 (714 mg, 1.01 mmol, 1.0 eq) was reacted according to GSP-3a using PBu_3 (304 µL, 1.22 mmol, 1.2 eq). **5a1** (290 mg, 1.52 mmol, 1.5 eq) reacted according to GSP-5 using oxalyl chloride (205 µL, 2.42 mmol, 2.4 eq) and THF. The resulting ylide was reacted with the acyl chloride **5a** in THF. The reaction mixture was stirred at rt overnight. In this case, no aqueous workup was performed. After FCC (petroleum ether/EtOAc 2:1), **5** (437 mg, 0.513 mmol, 51%) was obtained as a yellow wax.

TLC R_f = 0.25 (petroleum ether/EtOAc 2:1); **HPLC** t_r = 13.30 min (50-100% B), peak area 99%; **IR** ν_{max} (neat)/cm^{-1} = 2111 m (N_3), 1753 w (C=O), 1633 w, 1588 w, 1550 w, 1487 w, 1451 m, 1198 s (C-F), 1146 s (C-F); **^{1}H NMR** (500 MHz, DMSO-d_6, 90 °C) δ = 9.79 (s, 1H; H-13), 7.71 (t, J = 1.8 Hz, 1H; H-12), 7.61 (d, J = 8.0 Hz, 1H; H-10), 7.36 (t, J = 7.9 Hz, 1H; H-9), 7.32 (t, J = 7.8 Hz, 1H; H-19), 7.10-7.05 (m, 2H; H-18 and H-8), 6.99 (appq, J = 1.9 Hz, 1H; H-22), 6.93 (dd, J = 8.0, 1.8 Hz, 1H; H-20), 4.43 (t, J = 6.1 Hz, 2H; H-23), 4.25-4.20 (brs, 4H; H-2 and H-3), 3.12 (t, J = 2.4 Hz, 1H; H-5), 2.96 (t, J = 7.6 Hz, 2H; H-16), 2.73-2.61 (m, 4H; H-24 and H-15); **^{13}C NMR** (125 MHz, DMSO-d_6, 90 °C) due to interconversion, C-2 and C-3 are missing, δ = 169.9 × 2 (C-14 and C-6), 167.8 (C-1), 143.0 (C-17), 139.0 × 2 (C-21 and C-11), 134.9 (C-7), 129.3 (C-19), 128.3 (C-9), 124.7 (C-18), 120.6 (C-8), 120.3 (C-10), 118.4 (C-22), 117.2 (C-12), 116.3 (C-20), 78.0 (C-4), 74.7 (C-5), 56.4 (C-23), 37.1 (C-15), 30.0 (C-16), 29.6 (t, J = 20.6 Hz; C-24); **HRMS** (ESI+) m/z = 874.1263 $[M+Na]^+$ found, $C_{31}H_{22}F_{17}N_5O_4Na^+$ required 874.1293.

3,3,4,4,5,5,6,6,7,7,8,8,9,9,10,10,10-Heptadecafluorodecyl (*S*)-*N*-(3-(3-(6-azido-1-(methylamino)-1-oxohexan-2-yl)ureido)benzoyl)-*N*-(prop-2-yn-1-yl)glycinate (6)

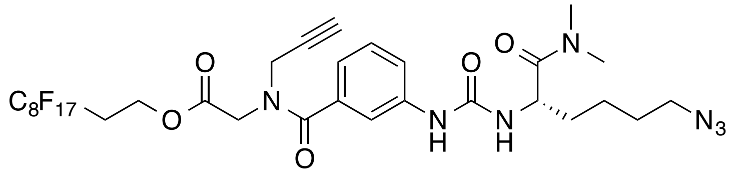

6 was synthesised according to our previous paper.[1]

tert-Butyl (6-(3-iodobenzamido)hexyl)carbamate (7a1)

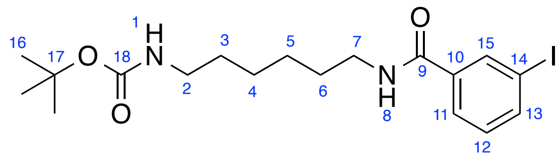

3-Iodobenzoic acid (574 mg, 2.31 mmol, 1.5 eq) was suspended in CH_2Cl_2 (6 mL). Oxalyl chloride (313 µL, 3.70 mmol, 2.4 eq) and catalytic amounts of DMF (5-10 drops) were added. After stirring until TLC indicated complete turnover (small samples of the reaction mixture were quenched with MeOH for that purpose), the solvent was removed under reduced pressure and the acyl chloride was used without further purification. *tert*-Butyl (6-aminohexyl)carbamate hydrochloride (389 mg, 1.54 mmol, 1.0 eq) was dissolved in CH_2Cl_2 (15 mL) and THF (3 mL) followed by the addition of excess sat. aq. $NaHCO_3$. The freshly prepared acyl chloride dissolved in CH_2Cl_2 (5 mL) and THF (3 mL) was added. After vigorous stirring for 2 h, the organic solvent was removed under reduced pressure and EtOAc added. The mixture was washed with 1 N aq. HCl, sat. aq. $NaHCO_3$, brine, dried over $MgSO_4$, filtered and the solvent removed under reduced pressure. After purification by Combi*Flash* (gradient of petroleum ether/EtOAc from 10:1 to 1:10 over 15 min), **7a1** (597 mg, 1.34 mmol, 87%) was obtained as a white solid.

TLC R_f = 0.10 (petroleum ether/EtOAc 3:1); **mp** 102-105 °C (CH_2Cl_2); **IR** ν_{max} (neat)/cm^{-1} = 3352 m, 3317 m, 2935 m, 2872 m, 1682 s (C=O)^{amide}, 1630 m (C=O)^{carbamate}, 1521 s, 1282 m, 1249 m, 1170 m; **^{1}H NMR** (400 MHz, $CDCl_3$) δ = 8.13 (s, 1H; H-15), 7.81 (dt, J = 7.9, 1.4 Hz, 1H; H-13), 7.75 (d, J = 7.8 Hz, 1H; H-11), 7.16 (t, J = 7.8 Hz, 1H; H-12), 6.38 (brs, 1H; H-8), 4.54 (brs, 1H; H-1), 3.43 (td, J = 7.0, 5.8 Hz, 2H; H-7), 3.13 (appq, J = 6.6 Hz, 2H; H-2), 1.68-1.57 (m, 2H; H-6), 1.55-1.45 (m, 2H; H-3), 1.43 (s, 9H; H-16), 1.41-1.31 (m, 4H; H-4 and H-5); **^{13}C NMR** (100 MHz, $CDCl_3$) δ = 166.1 (C-9), 156.3 (C-18), 140.2 (C-13), 136.8 (C-10), 136.2 (C-15), 130.2 (C-12), 126.3 (C-11), 94.3 (C-14), 79.2 (C-17), 40.1 (C-2), 39.8 (C-7), 30.1 (C-3), 29.4 (C-6), 28.5 (C-16), 26.2 (C-4 or C-5), 26.0 (C-4 or C-5).

***N*-(6-Aminohexyl)-3-iodobenzamide (7a)**

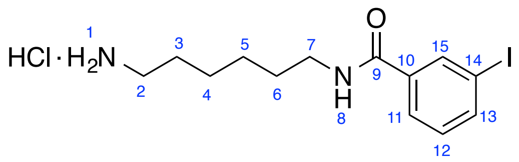

To the solution of **7a1** (597 mg, 1.34 mmol) in CH_2Cl_2 (25 mL) was added HCl in 1,4-dioxane (4 M; 6 mL). After stirring until TLC indicated complete turnover, the white precipitate was collected by vacuum filtration and triturated with CH_2Cl_2. **7a** (440 mg, 1.15 mmol, 90%) was obtained as a white solid.

mp 204-205 °C (CH_2Cl_2); **IR** ν_{max} (neat)/cm^{-1} = 3315 m (NH), 2924 s, 2867 m, 1630 s (C=O), 1533 s, 1475 m, 1341 m, 1301 m; **^{1}H NMR** (400 MHz, DMSO-d_6) δ = 8.61 (t, J = 5.6 Hz, 1H; H-8), 8.19 (t, J = 1.8 Hz, 1H; H-15), 7.97 (brs, 2H; H-1), 7.89-7.87 (m, 1H; H-13), 7.90-7.82 (m, 1H; H-11), 7.26 (t, J = 7.8 Hz, 1H; H-12), 3.24 (appq, J = 6.6 Hz, 2H; H-7), 2.81-2.66 (m, 2H; H-2), 1.66-1.42 (m, 4H; H-3 and H-6), 1.42-1.19 (m, 4H; H-4 and H-5); **^{13}C NMR** (100 MHz, DMSO-d_6) δ = 164.5 (C-9), 139.5 (C-13), 136.6 (C-10), 135.6 (C-15), 130.4 (C-12), 126.6 (C-11), 94.7 (C-14), 39.6 (C-7, coincides with solvent signal), 38.6 (C-2), 28.8 (C-6), 26.9 (C-3), 26.0 (C-4 or C-5), 25.5 (C-4 or C-5); **HRMS** (ESI+) m/z = 347.0618 $[M+H]^+$ found, $C_{13}H_{20}N_2O^{127}I^+$ required 347.0615.

3,3,4,4,5,5,6,6,7,7,8,8,9,9,10,10,10-Heptadecafluorodecyl *N*-(3-(3-(6-(3-iodobenzamido)hexyl)ureido)benzoyl)-*N*-(prop-2-yn-1-yl)glycinate (7)

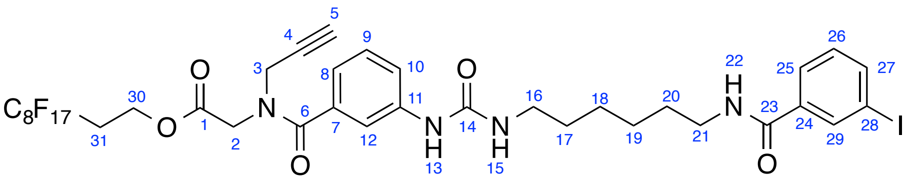

2 (593 mg, 0.842 mmol, 1.0 eq, dissolved in THF (6 mL)) was reacted according to GSP-3a using PBu_3 (252 µL, 1.01 mmol, 1.2 eq). The resulting ylide was reacted with **7a** (386 mg, 1.01 mmol, 1.2 eq, suspended in a mixed solvent of THF (6 mL) and DMF (12 mL)) and DIPEA (286 µL, 1.64 mmol, 2.0 eq) according to GSP-4. The solvent was removed under a stream of nitrogen and the residue dissolved in EtOAc. The organic phase was washed with 1 N aq. HCl, sat. aq. $NaHCO_3$ and brine, dried over $MgSO_4$, filtered and concentrated under reduced pressure. After purification by FCC (petroleum ether/EtOAc 1:2), **7** (661 mg, 0.629 mmol, 75%) was obtained as a white foam.

TLC R_f = 0.39 (petroleum ether/EtOAc 1:2); **HPLC** t_r = 13.58 min (50-100% B), peak area 99%; **mp** 95 °C decomposition (H_2O); **IR** ν_{max} (neat)/cm^{-1} = 1741 w (C=O), 1630 m, 1552 m, 1460 w, 1201 s (C-F), 1146 s (C-F); **^{1}H NMR** (500 MHz, DMSO-d_6, 90 °C) δ = 8.33 (s, 1H; H-13), 8.23 (brs, 1H; H-22), 8.17 (t, J = 1.7 Hz, 1H; H-29), 7.91-7.78 (m, 2H; H-27 and H-25), 7.59-7.51 (t, J = 1.7 Hz, 1H; H-12), 7.40 (d, J = 8.0 Hz, 1H; H-10), 7.27 (t, J = 7.9 Hz, 1H; H-9), 7.24 (t, J = 7.9 Hz, 1H; H-26), 6.92 (d, J = 7.4 Hz, 1H; H-8), 6.00 (t, J = 5.6 Hz, 1H; H-15), 4.43 (t, J = 6.1 Hz, 2H; H-30), 4.22

(appbrs, 4H; H-2 and H-3), 3.27 (td, J = 7.0, 5.3 Hz, 2H; H-21), 3.17-3.06 (m, 3H; H-5 and H-16), 2.67 (tt, J = 19.2, 6.6 Hz, 2H; H-31), 1.57 (quint, J = 7.1 Hz, 2H; H-20), 1.48 (quint, J = 7.0 Hz, 2H; H-17), 1.43-1.31 (m, 4H; H-18 and 19); **^{13}C NMR** (125 MHz, DMSO-d_6, 90 °C) due to interconversion, C-2 and C-3 are missing, δ = 170.2 (C-6), 167.9 (C-1), 164.3 (C-23), 154.7 (C-14), 140.5 (C-11), 138.9 (C-27), 136.7 (C-24), 135.2 (C-29), 134.8 (C-7), 129.8 (C-26), 128.2 (C-9), 126.1 (C-25), 118.8 (C-10), 118.5 (C-8), 115.6 (C-12), 93.5 (C-28), 78.1 (C-4), 74.7 (C-5), 56.4 (C-30), 38.9 (C-21), 38.7 (C-16), 29.6 (t, J = 21.4 Hz; C-31), 29.2 (C-17), 28.5 (C-20), 25.8 (C-18 or C-19), 25.6 (C-18 or C-19); **HRMS** (ESI+) m/z = 1051.1218 $[M+H]^+$ found, $C_{36}H_{33}F_{17}N_4O_5{}^{127}I^+$ required 1051.1219.

Hex-5-yn-1-amine hydrochloride (8a)

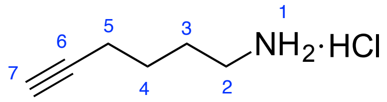

5-Hexyn1-ol (3.31 mL, 30.0 mmol, 1.0 eq) and pyridine (3.20 mL, 39.0 mmol, 1.3 eq) were dissolved in anhydrous CH_2Cl_2 (75 ml) and tosyl chloride (7.44 g, 39.0 mmol, 1.3 eq) was added in portions over 5 min at 0 °C. After stirring at rt for 18 h, CH_2Cl_2 (60 mL) was added and the organic phase was washed with 0.1 M aq. HCl (2 × 90 mL), sat. aq. $NaHCO_3$ (100 mL) and brine (100 mL), dried over Na_2SO_4, filtered and concentrated under reduced pressure to afford the crude tosylate as a colourless oil (8.60 g) which was used without further purification. The crude tosylate was dissolved in DMF (60 ml) and sodium azide (2.89 g, 44.5 mmol, 1.5 eq) was added. The mixture was stirred at rt overnight. Et_2O (40 mL) and water (20 mL) were added and the mixture was stirred for 5 min. The phases were separated and the aqueous phase was extracted with Et_2O (3 × 40 mL). The organic phases were combined and washed with water (50 mL), sat. aq. $NaHCO_3$ (50 mL) and water (50 mL), dried over Na_2SO_4 and filtered. The solution was concentrated under reduced pressure to leave 20-30 mL of solvent. The solution of crude azide was used in the next step without further purification. THF (75 ml) and PPh_3 (8.66 g, 33.0 mmol, 1.10 eq) were added to the ethereal solution of crude azide and the mixture stirred for 1.5 h. Water (1.5 mL, 83.3 mmol, 2.8 eq) was added and the solution stirred at rt for 4 days. The mixture was concentrated under reduced pressure to reduce the solvent volume by half and 3 M aq. HCl was added to adjust the pH to 1 and the aqueous mixture was washed with CH_2Cl_2 (3 × 90 mL). Na_2CO_3 (s) was added to the aqueous phase until pH ≥ 12, and the aqueous phase extracted with CH_2Cl_2 (20 × 30 mL). The organic phase was dried over $MgSO_4$, filtered, and 4 M HCl in dioxane (10.5 mL, 42 mmol, 1.4 equiv.) was added. The cloudy solution was concentrated under reduced pressure to afford a white solid which was triturated with Et_2O (2 × 60 mL) to give **8a** (1.0 g, 7.5 mmol, 25% over 3 steps) as a white solid.

TLC R_f = 0.07 (MeOH/CH_2Cl_2 1:9); **IR** (neat) n_{max} = 3220 s (NH_3^+), 2957 s, 2882 s, 1609 m, 1488 s, 1465 m; **^{1}H NMR** (400 MHz, DMSO-d_6) δ = 8.13 (s, 3H; NH_3^+), 2.81 (t, J = 2.6 Hz, 1H; H-7), 2.79-2.70 (m, 2H; H-2), 2.18 (td, J = 7.0, 2.7 Hz, 2H; H-5), 1.65 (quint, J = 7.5 Hz, 2H; H-3), 1.49 (quint, J = 7.1 Hz, 2H; H-4); **^{13}C NMR** (100 MHz, DMSO-d_6) δ = 84.0 (C-6), 71.6 (C-7), 38.2 (C-2), 26.1 (C-3), 24.9 (C-4), 17.3 (C-5); **HRMS** (ESI+) m/z = 98.0966 $[M+H]^+$ found, $C_6H_{12}N^+$ required 98.0964.

3,3,4,4,5,5,6,6,7,7,8,8,9,9,10,10,10-Heptadecafluorodecyl *N*-(3-(3-(hex-5-yn-1-yl)ureido)benzoyl)-*N*-(prop-2-yn-1-yl)glycinate (8)

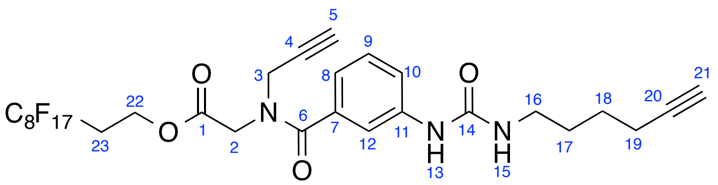

2 (2.4 g, 3.4 mmol, 1.0 eq) was reacted according to GSP-3a using PBu_3 (1.02 mL, 4.08 mmol, 1.2 eq). The resulting ylide was reacted with **8a** (547 mg, 4.11 mmol, 1.2 eq) according to GSP-4. After FCC (petroleum ether/EtOAc 1:1), **8** (1.76 g, 2.20 mmol, 65%) was obtained as a yellow foam.

HPLC t_r = 9.04 min (60-100% B), peak area 97%; **IR** ν_{max} (neat)/cm^{-1} = 1753 w (C=O), 1629 w, 1555 m, 1198 s (C-F), 1146 s (C-F); **^{1}H NMR** (500 MHz, DMSO-d_6, 90 °C) δ = 8.34 (s, 1H; H-13), 7.54 (s, 1H; H-12), 7.40 (d, J = 8.1 Hz, 1H; H-10), 7.28 (t, J = 7.8 Hz, 1H; H-9), 6.92 (d, J = 7.5 Hz, 1H; H-8), 6.03 (t, J = 5.7 Hz, 1H; H-15), 4.43 (t, J = 6.1 Hz, 2H; H-22), 4.22 (apps, 4H; H-2 and H-3), 3.16-3.10 (m, 3H; H-5 and H-16), 2.68 (tt, J = 19.0, 6.0 Hz, 2H; H-23), 2.55 (t, J = 2.7 Hz, 1H; H-21), 2.20 (td, J = 6.7, 2.7 Hz, 2H; H-19), 1.60-1.48 (m, 4H; H-17 and H-18); **^{13}C NMR** (125 MHz, DMSO-d_6, 90 °C) due to interconversion, C-2 and C-3 are missing, δ = 170.1 (C-6), 167.8 (C-1), 154.7 (C-14), 140.4 (C-11), 134.8 (C-7), 128.2 (C-9), 118.8 (C-10), 118.5 (C-8), 115.6 (C-12), 83.9 (C-20), 78.1 (C-4), 74.7 (C-5), 70.0 (C-21), 56.3 (C-22), 38.2 (C-16), 29.6 (C-23), 28.4 (C-17), 25.0 (C-18), 17.0 (C-19); **HRMS** (ESI+) m/z = 824.1418 $[M+H]^+$ found, $C_{29}H_{24}F_{17}N_3O_4Na^+$ required 824.1388.

3,3,4,4,5,5,6,6,7,7,8,8,9,9,10,10,10-Heptadecafluorodecyl *N*-(3-(3-(hex-5-en-1-yl) ureido)benzoyl)-*N*-(prop-2-yn-1-yl)glycinate (9)

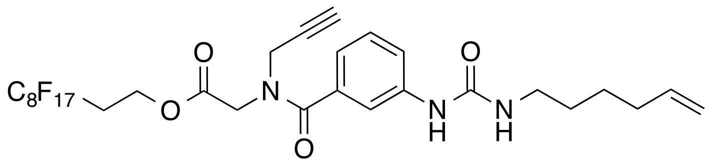

9 was synthesised according to our previous paper.[1]

3,3,4,4,5,5,6,6,7,7,8,8,9,9,10,10,10-Heptadecafluorodecyl *N*-allyl-*N*-(3-(3-(hex-5-en-1-yl)ureido)benzoyl)glycinate (10)

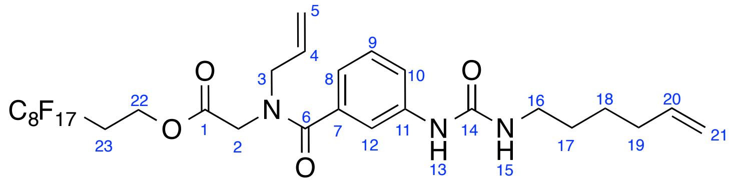

9 (100 mg, 0.124 mmol, 1.0 eq) was dissolved in methanol (6 mL) and 3,6-dithiaoctane-1,8-diol (4.5 mg, 0.25 mmol, 0.2 eq) was added. The reaction atmosphere was exchanged to N_2. Lindlar catalyst (10% w/w; 10 mg) was added and the reaction atmosphere exchanged to H_2. The mixture was stirred at rt for 12 h. Analytic HPLC indicated complete turnover. The Lindlar catalyst was filtered off through a pad of celite and the filtrate concentrated under reduced pressure. After FCC (CH_2Cl_2/EtOAc 4:1 → 3:1), **10** (83 mg, 0.10 mmol, 83%) was obtained as a colourless film.

TLC R_f = 0.16 (CH_2Cl_2/EtOAc 4:1); **HPLC** t_r = 14.38 min (50-100% B), peak area 94%; **IR** ν_{max} (neat)/cm^{-1} = 1748 m (C=O), 1641 m (C=O), 1561 m, 1197 s (C-F), 1144 s (C-F); **^{1}H NMR** (500 MHz, DMSO-d_6, 90 °C) δ = 8.31 (s, 1H; H-13), 7.53 (s, 1H; H-12), 7.36 (d, J = 7.7 Hz, 1H; H-10), 7.25 (t, J = 7.8 Hz, 1H; H-9), 6.88 (d, J = 7.5 Hz, 1H; H-8), 5.99 (t, J = 5.3 Hz, 1H; H-15), 5.89-5.78 (m, 2H; H-4 and H-20), 5.27-5.14 (m, 2H; H-5), 5.02 (d, J = 16.8 Hz, 1H; H-21$_{trans}$), 4.96 (d, J = 10.2 Hz, 1H; H-21$_{cis}$), 4.42 (t, J = 6.0 Hz, 2H; H-22), 4.10 (s, 2H; H-2), 3.97 (s, 2H; H-3), 3.11 (q, J = 6.4 Hz, 2H; H-16), 2.67 (tt, J = 19.2, 6.3 Hz, 2H; H-23), 2.07 (q, J = 7.0, 6.6 Hz, 2H; H-19), 1.54-1.45 (m, 2H; H-17), 1.47-1.37 (m, 2H; H-18); **^{13}C NMR** (125 MHz, DMSO-d_6, 90 °C) due to interconversion, C-2 and C-3 are missing, δ = 170.6 (C-6), 168.2 (C-1), 154.7 (C-14), 140.3 (C-11), 138.1 (C-20), 135.6 (C-7), 132.8 (C-4), 128.1 (C-9), 118.4 × 2 (C-8 and C-10), 117.1 (C-5), 115.4 (C-12), 114.0 (C-21), 56.2

(C-22), 38.6 (C-16), 32.2 (C-19), 29.6 (t, J = 20.6 Hz; C-23), 28.7 (C-17), 25.2 (C-18); **HRMS** (ESI+) m/z = 806.1871 $[M+H]^+$ found, $C_{29}H_{29}F_{17}N_3O_4^+$ required 806.1881.

3,3,4,4,5,5,6,6,7,7,8,8,9,9,10,10,10-Heptadecafluorodecyl *N*-(3-(3-(3-(3-iodobenzamido)phenyl)propanamido)benzoyl)-*N*-(prop-2-yn-1-yl)glycinate (11)

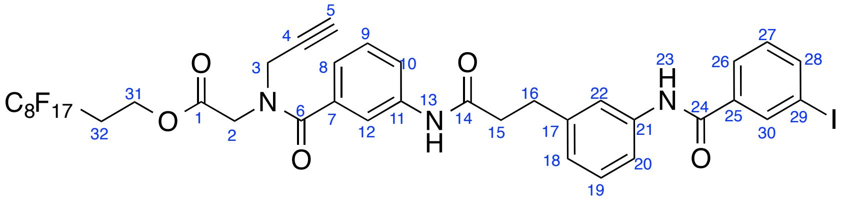

5 (670 mg, 0.787 mmol, 1.0 eq) was reacted according to GSP-3a using PBu_3 (235 µL, 0.944 mmol, 1.2 eq). 3-Iodobenzoic acid (293 mg, 1.18 mmol, 1.5 eq) reacted according to GSP-5 using oxalyl chloride (160 µL, 1.89 mmol, 2.4 eq) and THF. The resultant ylide reacted with acyl chloride **11a**. The reaction mixture was stirred at rt overnight and TLC indicated incomplete turnover. Additional acyl chloride **11a** was prepared from 3-iodobenzoic acid (147 mg, 0.590 mmol, 0.75 eq) according to GSP-5 using oxalyl chloride (80 µL, 0.95 mmol, 1.2 eq), and THF was added. The reaction mixture was stirred at rt overnight. After FCC (petroleum ether/EtOAc 3:2) and lyophilisation, **11** (586 mg, 0.555 mmol, 71%) was obtained as a white solid.

TLC R_f = 0.23 (petroleum ether/EtOAc 3:2); **HPLC** t_r = 8.73 min (70-100% B), peak area 99%; **mp** 100-105 °C (H_2O); **IR** ν_{max} (neat)/cm^{-1} = 1752 w (C=O), 1641 m, 1543 m, 1438 w, 1199 s (C-F), 1146 s (C-F); **^{1}H NMR** (500 MHz, DMSO-d_6, 90 °C) δ = 10.02 (s, 1H; H-23), 9.82 (s, 1H; H-13), 8.32 (t, J = 1.7 Hz, 1H; H-30), 7.97 (dt, J = 7.8, 1.3 Hz, 1H; H-26), 7.93 (dt, J = 7.9, 1.3 Hz, 1H; H-28), 7.75 (t, J = 1.8 Hz, 1H; H-12), 7.69 (t, J = 1.8 Hz, 1H; H-22), 7.64 (d, J = 8.2 Hz, 1H; H-10), 7.59 (d, J = 8.4 Hz, 1H; H-20), 7.36 (t, J = 7.9 Hz, 1H; H-9), 7.32 (t, J = 7.8 Hz, 1H; H-27), 7.26 (t, J = 7.8 Hz, 1H; H-19), 7.08 (d, J = 7.6 Hz, 1H; H-8), 7.02 (d, J = 7.6 Hz, 1H; H-18), 4.43 (t, J = 6.2 Hz, 2H; H-31), 4.24 (s, 2H; H-2 or H-3), 4.23 (s, 2H; H-2 or H-3), 3.11 (t, J = 2.4 Hz, 1H; H-5), 2.97 (t, J = 7.5 Hz, 2H; H-16), 2.74-2.60 (m, 4H; H-32 and H-15); **^{13}C NMR** (125 MHz, DMSO-d_6, 90 °C) due to interconversion, C-2 and C-3 are missing δ = 170.1 (C-14), 169.9 (C-6), 167.8 (C-1), 163.4 (C-24) 141.2 (C-17), 139.4 (C-28), 139.1 (C-11), 138.5 (C-21), 136.8 (C-25), 135.5 (C-30), 134.9 (C-7), 129.9 (C-27), 128.3 (C-9), 127.9 (C-19), 126.6 (C-26), 123.3 (C-18), 120.5 (C-8), 120.4 (C-10), 120.2 (C-22), 118.0 (C-20), 117.3 (C-12), 93.5 (C-29), 78.0 (C-4), 74.7 (C-5), 56.4 (C-31), 37.4 (C-15), 30.5 (C-16), 29.6 (t, J = 21.4 Hz; C-32); **HRMS** (ESI+) m/z = 1056.0847 $[M+H]^+$ found, $C_{38}H_{28}F_{17}N_3O_5{}^{127}I^+$ required 1056.0797.

3,3,4,4,5,5,6,6,7,7,8,8,9,9,10,10,10-Heptadecafluorodecyl *N*-(3-(3-(3-(3-allylureido) phenyl)propanamido)benzoyl)-*N*-(prop-2-yn-1-yl)glycinate (12)

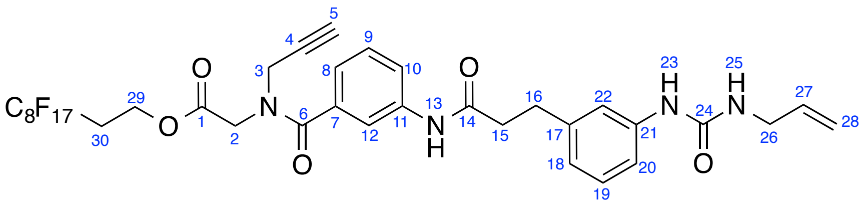

5 (343 mg, 0.403 mmol, 1.0 eq) was reacted according to GSP-3a using PBu_3 (121 µL, 0.484 mmol, 1.2 eq). The resulting ylide was reacted with allylamine (42 µL, 0.56 mmol, 1.4 eq) according to GSP-4 without addition of DIPEA. After FCC (petroleum ether/EtOAc 2:3), **12** (248 mg, 0.273 mmol, 68%) was obtained as a pale yellow foam.

TLC R_f = 0.15 (petroleum ether/EtOAc 1:1); **HPLC** t_r = 11.11 min (50-100% B), peak area 97%; **mp** 53 °C decomposition (H_2O); **IR** ν_{max} (neat)/cm^{-1} = 1751 w (C=O), 1646 m, 1553 m, 1490 w, 1433 w, 1200 s (C-F), 1146 s (C-F); **^{1}H NMR** (500 MHz, DMSO-d_6, 90 °C) δ = 9.79 (s, 1H; H-13), 8.17 (s, 1H; H-23), 7.72 (t, J = 1.8 Hz, 1H; H-12), 7.62 (d, J = 8.6 Hz, 1H; H-10), 7.36 (t, J = 7.9 Hz, 1H; H-9), 7.31 (t, J = 1.9 Hz, 1H; H-22), 7.20 (dd, J = 8.2, 1.1 Hz, 1H; H-20), 7.12 (t, J = 7.8 Hz, 1H; H-19), 7.07 (d, J = 7.6 Hz, 1H; H-8), 6.80 (dt, J = 7.6, 1.3 Hz, 1H; H-18), 6.07 (t, J = 5.8 Hz, 1H; H-25), 5.88 (ddt, J = 17.1, 10.5, 5.3 Hz, 1H; H-27), 5.18 (dq, J = 17.2, 1.8 Hz, 1H; H-28$_{trans}$), 5.07 (dq, J = 10.4, 1.6 Hz, 1H; H-28$_{cis}$), 4.43 (t, J = 6.1 Hz, 2H; H-29), 4.23 (s, 2H; H-2 or H-3), 4.22 (s, 2H; H-3 or H-2), 3.74 (tt, J = 5.6, 1.7 Hz, 2H; H-26), 3.12 (t, J = 2.4 Hz, 1H; H-5), 2.88 (dd, J = 8.7, 6.8 Hz, 2H; H-16), 2.68 (tt, J = 19.5, 6.4 Hz, 2H; H-30), 2.62 (t, J = 7.2 Hz, 2H; H-15); **^{13}C NMR** (125 MHz, DMSO-d_6, 90 °C) due to interconversion, C-2 and C-3 are missing, δ = 170.1 (C-14), 169.9 (C-6), 167.8 (C-1), 154.6 (C-24), 141.1 (C-17), 140.1 (C-21), 139.0 (C-11), 135.8 (C-27), 134.8 (C-7), 128.3 (C-9), 127.9 (C-19), 120.6 (C-18), 120.5 (C-8), 120.4 (C-10), 117.5 (C-22), 117.2 (C-12), 115.4 (C-20), 114.2 (C-28), 78.0 (C-4), 74.7 (C-5), 56.4 (C-29), 41.1 (C-26), 37.4 (C-15), 30.5 (C-16), 29.6 (t, J = 20.9 Hz; C-30); **HRMS** (ESI+) m/z = 909.1940 $[M+H]^+$ found, $C_{35}H_{30}F_{17}N_4O_5^+$ required 909.1939.

2-(4-Azidophenyl)ethan-1-amine (13a)

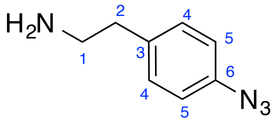

4-(2-Aminoethyl) aniline (521 mg, 3.83 mmol, 1.0 eq) was reacted according to GSP-1. After stirring for 10 min at 0 °C, 25% aq. NaOH was added to adjusted the mixture to basic. EtOAc was added, the layers were separated and the aqueous layer extracted with EtOAc (5 × 120 mL). The combined organic layers were washed with water and brine, dried over $MgSO_4$, filtered and concentrated under reduced pressure. **13a** (542 mg, 3.34 mmol, 87%) was obtained as a red oil.

TLC R_f = 0.12 (CH_2Cl_2/MeOH 10:1); **IR** ν_{max} (neat)/cm^{-1} = 3243 brs, 2925 w, 2865 w, 2097 s (N_3), 1579 m, 1506 s, 1286 s, 815 m; **^{1}H NMR** (400 MHz, DMSO-d_6) δ = 7.24 (d, J = 8.4 Hz, 2H; H-4 × 2), 7.03 (d, J = 8.4 Hz, 2H; H-5 × 2), 2.73 (t, J = 6.9 Hz, 2H; H-1), 2.61 (t, J = 7.2 Hz, 2H; H-2); **^{13}C NMR** (100 MHz, DMSO-d_6) δ = 137.6 (C-3), 136.8 (C-6), 130.2 (C-4 × 2), 118.9 (C-5 × 2), 43.6 (C-1), 39.0 (C-2, coincides with solvent signal); **HRMS** (ESI+) m/z = 163.0977 [M+H]$^+$ found, $C_8H_{11}N_4^+$ required 163.0978.

3,3,4,4,5,5,6,6,7,7,8,8,9,9,10,10,10-Heptadecafluorodecyl *N*-(3-(3-(3-(3-(4-azidophenethyl)ureido)phenyl)propanamido)benzoyl)-*N*-(prop-2-yn-1-yl)glycinate (13)

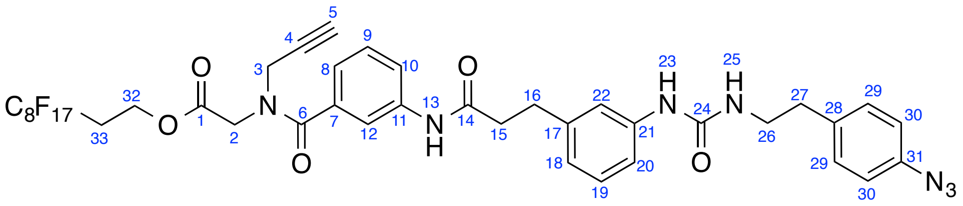

5 (402 mg, 0.472 mmol, 1.0 eq) was reacted according to GSP-3a using PBu_3 (142 µL, 0.570 mmol, 1.2 eq), and subsequently with **13a** (107 mg, 0.661 mmol, 1.4 eq) according to GSP-4 without addition of DIPEA. After FCC (petroleum ether/EtOAc 2:3), **13** (269 mg, 0.265 mmol, 56%) was obtained as a colourless foam.

TLC R_f = 0.37 (petroleum ether/EtOAc 1:1); **HPLC** t_r= 11.28 min (60-100% B), peak area 97%; **IR** ν_{max} (neat)/cm^{-1} = 2114 m (N_3), 1753 w (C=O), 1651 w (C=O), 1558 m, 1507 w, 1438 w, 1201 s (C-F), 1147 s (C-F); **^{1}H NMR** (500 MHz, DMSO-d_6, 70 °C) δ = 9.89 (s, 1H; H-13), 8.22 (s, 1H; H-23), 7.74 (t, J = 1.8 Hz, 1H; H-12), 7.62 (d, J = 7.8 Hz, 1H; H-10), 7.36 (t, J = 7.9 Hz, 1H; H-9), 7.31-7.27 (m, 3H; H-22 and H-29), 7.19 (dd, J = 8.2, 2.2 Hz, 1H; H-20), 7.11 (t, J = 7.8 Hz, 1H; H-19), 7.09-7.01 (m, 3H; H-30 and H-8), 6.78 (dt, J = 7.5, 1.3 Hz, 1H; H-18), 5.99 (t, J = 5.7 Hz, 1H; H-25), 4.42 (t, J = 6.1 Hz, 2H; H-32), 4.22 (appbrs, 4H; H-2 and H-3), 3.34 (q, J = 7.0 Hz, 2H; H-26), 3.19 (apps, 1H; H-5), 2.87 (t, J = 7.7 Hz, 2H; H-16), 2.76 (t, J = 7.1 Hz, 2H; H-27), 2.68 (tt, J = 19.7, 5.9 Hz, 2H; H-33), 2.61 (t, J = 7.9 Hz, 2H; H-15); **^{13}C NMR** (125 MHz, DMSO-d_6, 70 °C) due to interconversion,

C-2, C-3 and C-5 are missing, δ = 170.3 (C-14), 170.0 (C-6), 168.0 (C-1), 154.8 (C-24), 141.2 (C-17), 140.2 (C-21), 139.2 (C-11), 136.9 (C-31), 136.4 (C-28), 134.9 (C-7), 129.8 (C-29 × 2), 128.4 (C-9), 128.2 (C-19), 120.6 × 2 (C-18 and C-8) 120.4 (C-10), 118.7 (C-30 × 2), 117.4 (C-22), 117.2 (C-12), 115.3 (C-20), 78.2 (C-4), 56.5 (C-32), 40.2 (C-26), 37.6 (C-15), 34.9 (C-27), 30.6 (C-16), 29.5 (t, J = 21.8 Hz; C-33); **HRMS** (ESI+) m/z = 1014.2263 $[M+H]^+$ found, $C_{40}H_{33}F_{17}N_7O_5^+$ required 1014.2266.

(*S*)-2-Amino-6-azido-*N*,*N*-dimethylhexanamide hydrochloride (6a)

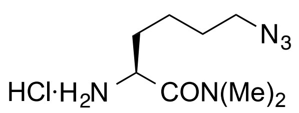

6a was synthesised according to our previous paper.[1]

3,3,4,4,5,5,6,6,7,7,8,8,9,9,10,10,10-Heptadecafluorodecyl (*R*)-*N*-(3-(3-(3-(3-(4-(3-(6-azido-1-(dimethylamino)-1-oxohexan-2-yl)ureido)phenethyl)ureido)phenyl) propanamido)benzoyl)-*N*-(prop-2-yn-1-yl)glycinate (14)

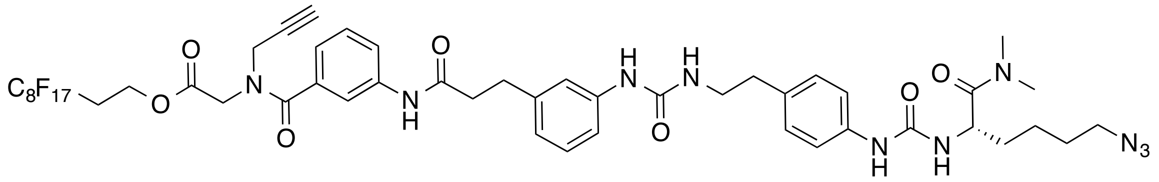

13 (152 mg, 0.150 mmol, 1.0 eq) was reacted according to GSP-3a using PBu_3 (45 µL, 0.18 mmol, 1.2 eq). The resulting ylide was reacted with **6a** (49 mg, 0.21 mmol, 1.4 eq) according to GSP-4 using DIPEA (52 µL, 0.30 mmol, 2.0 eq). After F-SPE, **14** (150 mg) was obtained as a pale yellow solid and was used in the next step without further purification.

TLC R_f = 0.075 (EtOAc); **HPLC** t_r = 11.85 min (50-100% B), peak area 83%; **HRMS** (ESI+) m/z = 1235.3408 $[M+Na]^+$ found, $C_{49}H_{49}F_{17}N_{10}O_7Na^+$ required 1235.3406.

3,3,4,4,5,5,6,6,7,7,8,8,9,9,10,10,10-Heptadecafluorodecyl *N*-(3-(8-azido-*N*-(2-(cyclohexylamino)-2-oxo-1-phenylethyl)octanamido)benzoyl)-*N*-(prop-2-yn-1-yl)glycinate (17a)

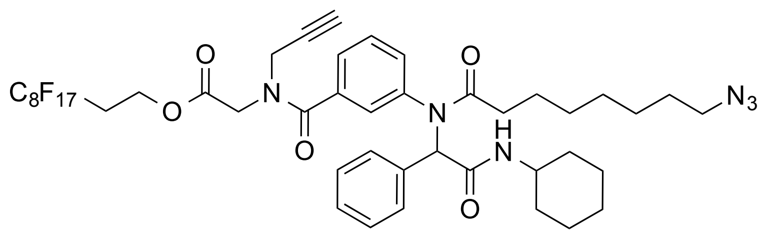

2 (0.10 g, 0.14 mmol, 1.0 eq) was reacted according to GSP-3a using PBu_3 (53 µL, 0.22 mmol, 1.2 eq). Benzaldehyde (22 µL, 0.21 mmol, 1.5 eq) was added, and the reaction was stirred at 40 °C until LCMS indicated completed turnover (typically 5 h). The solvent was removed by a flow of air, and the residue was redissolved in dry MeOH (2.0 mL). 8-azidooctanoic acid (7.8 mg, not pure, max 0.17 mmol) was added, and the reaction was stirred for 1.5 h under argon at 60 °C. Isocyanocyclohexane (5.3 µL, 0.17 mmol, 1.2 eq) was added, and the reaction was stirred at 60 °C overnight. Additional equivalents of 8-azidooctanoic acid (7.8 mg, not pure, max 0.17 mmol) and isocyanocyclohexane (5.3 µL, 0.17 mmol, 1.2 eq) were added, and the reaction was continually stirred under argon at 60 °C overnight. After LCMS indicated completed turnover, the solvent was removed under reduced pressure, and the residue was purified by F-SPE. **17a** (67 mg, not pure, max 0.065 mmol) was isolated as a golden wax and was used without further purification.

TLC R_f = 0.64 (petroleum ether/EtOAc 1:1); **HPLC** t_r = 15.10 min (50-100% B); **HRMS** (ESI+) *m/z* = 1083.3083 $[M+Na]^+$ found, $C_{44}H_{45}F_{17}N_6O_5Na^+$ required 1083.3078.

3,3,4,4,5,5,6,6,7,7,8,8,9,9,10,10,10-Heptadecafluorodecyl *N*-(3-(8-azido-*N*-(1-(4-bromophenyl)-2-(cyclohexylamino)-2-oxoethyl)octanamido)benzoyl)-*N*-(prop-2-yn-1-yl)glycinate (17b)

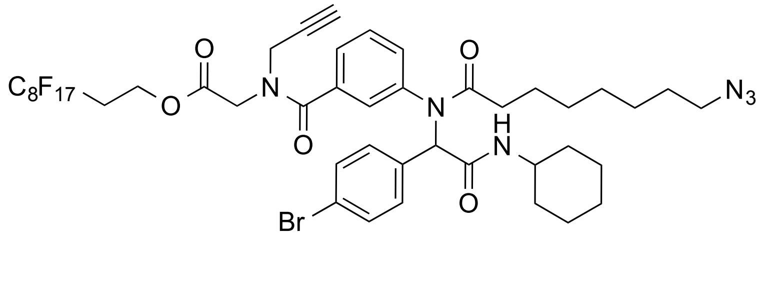

2 (0.10 g, 0.14 mmol, 1.0 eq) was reacted according to GSP-3a using PBu_3 (53 µL, 0.22 mmol, 1.2 eq). 4-bromobenzaldehyde (9.8 mg, 0.053 mmol, 1.5 eq) was added, and the reaction was stirred at 40 °C until LCMS indicated completed turnover (typically 5 h). The solvent was removed by a flow of

air, and the residue was redissolved in dry MeOH. 8-Azidooctanoic acid (7.8 mg, not pure, max 0.17 mmol) was added, and the reaction was stirred for 1.5 h under argon at 60 °C. Isocyanocyclohexane (5.3 µL, 0.17 mmol, 1.2 eq) was added, and the reaction was stirred overnight under argon at 60 °C. Additional equivalents of 8-azidooctanoic acid (7.8 mg, not pure, max 0.17 mmol) and isocyanocyclohexane (5.3 µL, 0.17 mmol, 1.2 eq) were added, and the reaction was continually stirred under argon at 60 °C overnight. After LCMS indicated completed turnover, the solvent was removed under reduced pressure, and the residue purified by F-SPE. **17b** (87 mg, not pure, max 0.077 mmol) was obtained as a white powder and was used without further purification.

TLC R_f = 0.40 (petroleum ether/EtOAc 1:3); **HPLC** t_r = 12.47 min (70-100% B); **HRMS** (ESI+) *m/z* = 1161.2182 $[M+Na]^+$ found, $C_{44}H_{45}BrF_{17}N_6O_5Na^+$ required 1161.2183.

3-(4-Hydroxybut-1-yn-1-yl)benzaldehyde (16a1)

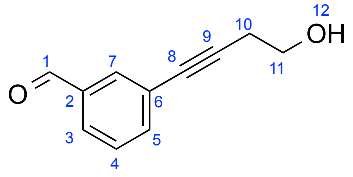

3-Bromobenzaldehyde (1.13 mL, 10.0 mmol, 1.0 eq) was dissolved in anhydrous DMF (12 mL), 3-butyn-1-ol (1.14 mL, 15.0 mmol, 1.5 eq) and triethylamine (6.9 mL, 50 mmol, 5.0 eq) were added. Argon was bubbled through the solution for 10 min. CuI (95 mg, 0.50 mmol, 0.05 eq) and $PdCl_2(PPh_3)_2$ (351 mg, 0.50 mmol, 0.05 eq) were added. The mixture was stirred at rt overnight. The mixture was partitioned between Et_2O and 10% aq. HCl. The organic phase was collected and washed with 10% aq. HCl (3 ×), sat. aq. $NaHCO_3$ and brine, dried over $MgSO_4$, filtered and concentrated under reduced pressure. After FCC (petroleum ether/EtOAc 3:1 → 2:1), **16a1** (1.36 g, 7.81 mmol, 78%) was obtained as a yellow oil.

TLC R_f = 0.24 (petroleum ether/EtOAc 3:1); **IR** ν_{max} (neat)/cm^{-1} = 1695 s (C=O), 1598 w, 1575 m, 1280 m, 1157 m, 1042 s; **^{1}H NMR** (400 MHz, $CDCl_3$) δ = 9.98 (s, 1H; H-1), 7.91 (t, *J* = 1.5 Hz, 2H; H-7), 7.80 (dt, *J* = 7.7, 1.4 Hz, 1H; H-3), 7.66 (dt, *J* = 7.7, 1.4 Hz, 1H; H-5), 7.47 (t, *J* = 7.7 Hz, 1H; H-4), 3.84 (q, *J* = 5.8 Hz, 2H; H-11), 2.72 (t, *J* = 6.3 Hz, 2H; H-10), 1.79 (brs, 1H; H-12); **^{13}C NMR** (100 MHz, $CDCl_3$) δ = 191.7 (C-1), 137.4 (C-5), 136.6 (C-2), 133.2 (C-7), 129.2 (C-4), 128.9 (C-3), 124.8 (C-6), 88.5 (C-9), 81.2 (C-8), 61.2 (C-11), 23.9 (C-10); **HRMS** (ESI+) *m/z* = 175.0746 $[M+H]^+$ found, $C_{11}H_{11}O_2^+$ required 175.0754.

3-(4-Hydroxybutyl)benzaldehyde (16a2)

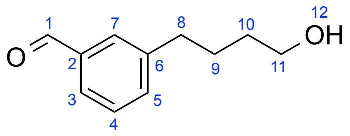

16a1 (3.47 g, 18.8 mmol) was dissolved in EtOAc (110 mL). Pd/C (10% w/w) was added and the reaction atmosphere was exchanged to H_2. The mixture was stirred at rt over two days. NMR indicated complete turnover. Pd/C was filtered off through a pad of celite and the filtrate concentrated under reduced pressure. After FCC (petroleum ether/EtOAc 2:1), **16a2** (1.21 g, 6.78 mmol, 34%) was obtained as a yellow oil.

TLC R_f = 0.57 (petroleum ether/EtOAc 2:1); **IR** ν_{max} (neat)/cm^{-1} = 3381 brs (OH), 2933 m, 2861 m, 1693 s (C=O), 1603 w, 1586 w, 1241 m, 1142 m, 1057 m; **^{1}H NMR** (400 MHz, $CDCl_3$) δ = 10.00 (s, 1H; H-1), 7.80-7.63 (m, 2H; H-3 and H-7), 7.51-7.39 (m, 2H; H-4 and H-5), 3.68 (t, J = 6.4 Hz, 2H; H-11), 2.74 (t, J = 7.6 Hz, 2H; H-8), 1.86-1.69 (m, 2H; H-9), 1.69-1.57 (m, 2H; H-10), 1.48 (brs, 1H; H-12); **^{13}C NMR** (100 MHz, $CDCl_3$) δ = 192.7 (C-1), 143.6 (C-6), 136.8 (C-2), 134.8 (C-5), 129.4 (C-7), 129.2 (C-4), 127.9 (C-3), 62.8 (C-11), 35.5 (C-8), 32.3 (C-10), 27.5 (C-9); **HRMS** (ESI+) m/z = 179.1061 $[M+H]^+$ found, $C_{11}H_{15}O_2^+$ required 179.1067.

3-(4-Azidobutyl)benzaldehyde (16a)

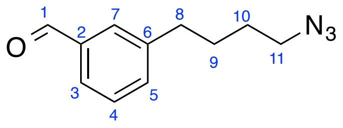

16a2 (1.21 g, 6.79 mmol, 1.0 eq) was reacted according to GSP-2. After FCC (petroleum ether/EtOAc 15:1), **16a** (0.81 g, 4.0 mmol, 59%) was obtained as a colourless oil.

TLC R_f = 0.30 (petroleum ether/EtOAc 9:1); **IR** ν_{max} (neat)/cm^{-1} = 2939 w, 2862 w, 2089 s (N_3), 1694 s (C=O), 1604 m, 1586 m, 1450 w, 1241 m; **^{1}H NMR** (400 MHz, $CDCl_3$) δ = 10.01 (s, 1H; H-1), 7.77-7.65 (m, 2H; H-3 and H-7), 7.52-7.42 (m, 2H; H-4 and H-5), 3.31 (t, J = 6.7 Hz, 2H; H-11), 2.74 (t, J = 7.6 Hz, 2H; H-8), 1.82-1.70 (m, 2H; H-9), 1.68-1.59 (m, 2H; H-10); **^{13}C NMR** (100 MHz, $CDCl_3$) δ = 192.6 (C-1), 143.1 (C-6), 136.8 (C-2), 134.8 (C-5), 129.3 (C-4 or C-7), 129.2 (C-4 or C-7), 128.1 (C-3), 51.4 (C-11), 35.2 (C-8), 28.5 (C-9 or C-10), 28.4 (C-10 or C-9).

3,3,4,4,5,5,6,6,7,7,8,8,9,9,10,10,10-Heptadecafluorodecyl *N*-(3-((2*S,3*R**)-2-(3-(4-azidobutyl)phenyl)-3-methoxy-4-oxoazetidin-1-yl)benzoyl)-*N*-(prop-2-yn-1-yl)glycinate (18a)**

and **3,3,4,4,5,5,6,6,7,7,8,8,9,9,10,10,10-heptadecafluorodecyl *N*-(3-((2*R**,3*R**)-2-(3-(4-azidobutyl)phenyl)-3-methoxy-4-oxoazetidin-1-yl)benzoyl)-*N*-(prop-2-yn-1-yl) glycinate (18b)**
Structures see below.

2 (326 mg, 0.463 mmol, 1.0 eq) was reacted according to GSP-3a using PBu_3 (139 µL, 0.555 mmol, 1.2 eq). **16a** (140 mg, 0.694 mmol, 1.5 eq) dissolved in THF (3 mL) was added. The reaction mixture was stirred at 40 °C overnight. The solvent was removed under a stream of nitrogen and the residue dissolved in CH_2Cl_2 and transferred to a sealed tube. Triethylamine (1 mL, 7 mmol, 16 eq) was added at 0 °C followed by methoxyacetyl chloride (0.21 mL, 2.3 mmol, 5 eq). The reaction mixture was then heated at 40 °C overnight. The mixture was washed with 10% aq. HCl, sat. aq. $NaHCO_3$ and brine, dried over $MgSO_4$, filtered and concentrated under reduced pressure. After FCC (petroleum ether/EtOAc 5:2), **18b** (111 mg, 0.119 mmol, 26%) and **18a** (176 mg, 0.188 mmol, 41%) were both obtained as colourless films.

18a

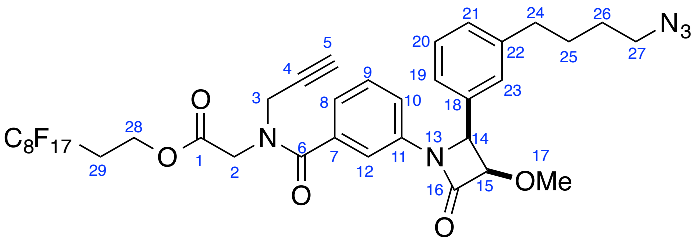

TLC R_f = 0.26 (petroleum ether/EtOAc 2:1); **HPLC** t_r = 13.53 min (50-100% B), peak area 100%; **mp** 56 °C decomposition (H_2O); **IR** ν_{max} (neat)/cm^{-1} = 2097 m (N_3), 1753 s (C=O), 1650 m (C=O), 1604 w, 1583 w, 1454 m, 1374 m, 1236 s, 1199 s (C-F), 1146 s (C-F), 1115 s; **^{1}H NMR** (500 MHz, DMSO-d_6, 120 °C) δ = 7.40 (t, J = 1.9 Hz, 1H; H-12), 7.37 (t, J = 7.8 Hz, 1H; H-9), 7.32-7.30 (m, 1H; H-10), 7.27 (t, J = 7.5 Hz, 1H; H-20), 7.21 (t, J = 1.6 Hz, 1H; H-23), 7.20-7.15 (m, 2H; H-19 and H-21), 7.11 (dt, J = 7.5, 1.4 Hz, 1H; H-8), 5.42 (d, J = 5.0 Hz, 1H; H-14), 4.98 (d, J = 5.0 Hz, 1H; H-15), 4.41 (t, J = 6.2 Hz, 2H; H-28), 4.17 (s, 2H; H-2), 4.13 (t, J = 3.0 Hz, 2H; H-3), 3.28 (t, J = 6.8 Hz, 2H; H-27), 3.14 (s, 3H; H-17), 3.00 (t, J = 2.5 Hz, 1H; H-5), 2.73-2.58 (m, 4H; H-29 and H-24), 1.70-1.61 (m, 2H; H-25), 1.59-1.49 (m, 2H; H-26); **^{13}C NMR** (125 MHz, DMSO-d_6, 27 °C) major rotamer signals only, δ = 169.7 (C-6), 168.2 (C-1), 164.5 (C-16), 141.9 (C-22), 137.1 (C-7 or C-11), 135.4

(C-7 or C-11), 133.5 (C-18), 129.6 (C-9), 128.4 × 2 (C-21 and C-20), 127.8 (C-23), 125.3 (C-19), 122.1 (C-8), 118.6 (C-10), 115.4 (C-12), 84.5 (C-15), 78.5 (C-4), 76.0 (C-5), 60.8 (C-14), 57.7 (C-17), 56.8 (C-28), 50.4 (C-27), 46.6 (C-2), 39.9 (C-3, coincides with solvent signal), 34.3 (C-24), 29.4 (C-29), 27.9 (C-25), 27.6 (C-26); **HRMS** (ESI+) *m/z* = 936.2065 $[M+H]^+$ found, $C_{36}H_{31}F_{17}N_5O_5^+$ required 936.2048.

18b

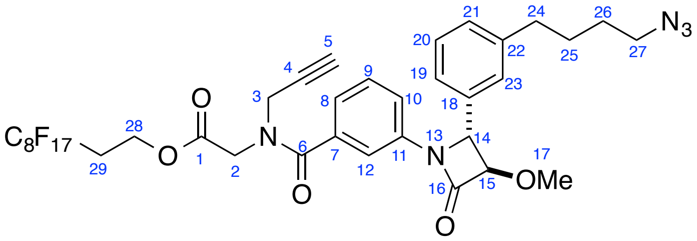

TLC R_f = 0.41 (petroleum ether/EtOAc 2:1); **HPLC** t_r = 13.77 min (50-100% B), peak area 100%; **mp** 50 °C decomposition (H_2O); **IR** ν_{max} (neat)/cm^{-1} = 2098 m (N_3), 1755 m (C=O), 1651 m (C=O), 1605 w, 1584 w, 1453 m, 1374 m, 1236 s, 1199 s (C-F), 1145 s (C-F); **^{1}H NMR** (500 MHz, DMSO-d_6, 120 °C) δ = 7.37 (t, *J* = 1.6 Hz, 1H; H-12), 7.36 (t, *J* = 7.7 Hz, 1H; H-9), 7.31 (t, *J* = 7.7 Hz, 1H; H-20), 7.29-7.27 (m, 1H; H-19), 7.24 (t, *J* = 1.6 Hz, 1H; H-23), 7.22 (dt, *J* = 7.7, 1.6 Hz, 1H; H-10), 7.19 (dt, *J* = 7.5, 1.6 Hz, 1H; H-21), 7.12 (dt, *J* = 7.6, 1.4 Hz, 1H; H-8), 5.14 (d, *J* = 1.9 Hz, 1H; H-14), 4.56 (d, *J* = 1.9 Hz, 1H; H-15), 4.41 (t, *J* = 6.1 Hz, 2H; H-28), 4.16 (s, 2H; H-2), 4.12 (t, *J* = 2.9 Hz, 2H; H-3), 3.52 (s, 3H; H-17), 3.28 (t, *J* = 6.7 Hz, 2H; H-27), 3.01 (t, *J* = 2.5 Hz, 1H; H-5), 2.72-2.58 (m, 4H; H-29 and H-24), 1.65 (dquint, *J* = 7.5, 2.2 Hz, 2H; H-25), 1.55 (dquint, *J* = 7.1, 1.3 Hz, 2H; H-26); **^{13}C NMR** (125 MHz, DMSO-d_6, 27 °C) major rotamer signals only, δ = 169.6 (C-6), 168.1 (C-1), 164.0 (C-16), 142.8 (C-22), 136.7 (C-7 or C-11), 136.1 (C-18), 135.3 (C-7 or C-11), 129.6 (C-9), 129.1 (C-20), 128.6 (C-21), 126.3 (C-23), 123.8 (C-19), 122.2 (C-8), 118.8 (C-10), 115.8 (C-12), 90.4 (C-15), 78.5 (C-4), 76.0 (C-5), 61.8 (C-14), 57.1 (C-17), 56.7 (C-28), 50.4 (C-27), 46.6 (C-2), 39.9 (C-3, coincides with solvent signal), 34.3 (C-24), 29.4 (C-29), 27.8 (C-25), 27.7 (C-26); **HRMS** (ESI+) *m/z* = 936.2089 $[M+H]^+$ found, $C_{36}H_{31}F_{17}N_5O_5^+$ required 936.2048.

3,3,4,4,5,5,6,6,7,7,8,8,9,9,10,10,10-Heptadecafluorodecyl *N*-(3-(2-(3-(4-azidobutyl) phenyl)-4-oxo-3,4-dihydropyridin-1(2*H*)-yl)benzoyl)-*N*-(prop-2-yn-1-yl)glycinate (19)

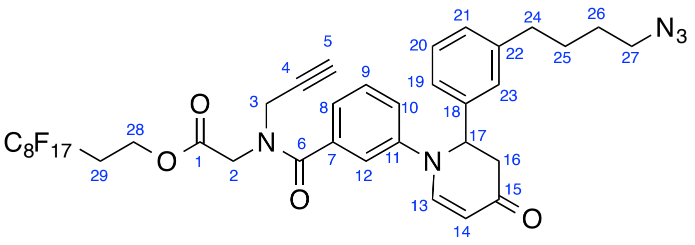

2 (85 mg, 0.12 mmol, 1.0 eq) was reacted according to GSP-3a using PBu_3 (36 µL, 0.14 mmol, 1.2 eq). **16a** (36 mg, 0.18 mmol, 1.5 eq) dissolved in THF (3 mL) was added. The reaction mixture was stirred at 40 °C for 6 h. $Yb(OTf)_3$ (82 mg, 0.13 mmol, 1.1 eq) and Danishefsky's diene (33 µL, 0.17 mmol, 1.4 eq) were added and the mixture stirred at 40 °C overnight. The solvent was removed under a stream of nitrogen and the residue dissolved in EtOAc. The organic phase was washed with 5% aq. HCl, sat. aq. $NaHCO_3$ and brine, dried over $MgSO_4$, filtered and concentrated under reduced pressure. After FCC (petroleum ether/EtOAc 1:1), **19** (96 mg, 0.10 mmol, 86%) was obtained as a colourless film.

TLC R_f = 0.17 (petroleum ether/EtOAc 1:1); **HPLC** t_r = 10.58 min (60-100% B), peak area 90%. **IR** ν_{max} (neat)/cm^{-1} = 2931 w, 2096 m (N_3), 1752 m (C=O), 1646 m (C=O), 1573 s, 1199 s (C-F), 1146 s (C-F); **^{1}H NMR** (500 MHz, DMSO-d_6, 120 °C) δ = 7.85 (dd, J = 7.9, 0.9 Hz, 1H; H-13), 7.38 (t, J = 7.9 Hz, 1H; H-9), 7.27 (ddd, J = 8.3, 2.5, 0.9 Hz, 1H; H-10), 7.24-7.19 (m, 2H; H-20 and H-12), 7.15-7.11 (m, 3H; H-23, H-8 and H-19), 7.08 (d, J = 7.5 Hz, 1H; H-21), 5.48 (dd, J = 6.9, 4.1 Hz, 1H; H-17), 5.13 (dd, J = 7.9, 0.9 Hz, 1H; H-14), 4.42 (t, J = 6.2 Hz, 2H; H-28), 4.19 (s, 2H; H-2), 4.14 (s, 2H; H-3), 3.29 (t, J = 6.7 Hz, 2H; H-27), 3.18 (dd, J = 16.4, 7.1 Hz, 1H; H-16a), 3.03 (t, J = 2.4 Hz, 1H; H-5), 2.74-2.55 (m, 5H; H-29, H-16b and H-24), 1.70-1.59 (m, 2H; H-25), 1.59-1.47 (m, 2H; H-26); **^{13}C NMR** (125 MHz, DMSO-d_6, 120 °C) due to interconversion, C-2 and C-3 are missing, δ = 188.4 (C-15), 169.3 (C-6), 167.5 (C-1), 147.4 (C-13), 144.0 (C-11), 141.7 (C-22), 138.2 (C-18), 135.5 (C-7), 128.9 (C-9), 127.9 (C-20), 126.8 (C-21), 125.6 (C-23), 123.1 (C-19), 121.3 (C-8), 120.1 (C-10), 116.9 (C-12), 101.9 (C-14), 77.8 (C-4), 74.4 (C-5), 60.1 (C-17), 56.2 (C-28), 50.2 (C-27), 42.8 (C-16), 33.8 (C-24), 29.7 (t, J = 20.9 Hz; C-29), 27.1 (C-25 or C-26), 26.8 (C-25 or C-26); **HRMS** (ESI+) m/z = 932.2092 $[M+H]^+$ found, $C_{37}H_{31}N_5O_4F_{17}^+$ required 932.2099.

3,3,4,4,5,5,6,6,7,7,8,8,9,9,10,10,10-Heptadecafluorodecyl *N*-(3-((1*R,4*R**)-3-(3-(4-azidobutyl)phenyl)-5-oxo-2-azabicyclo[2.2.2]octan-2-yl)benzoyl)-*N*-(prop-2-yn-1-yl)glycinate (20)**

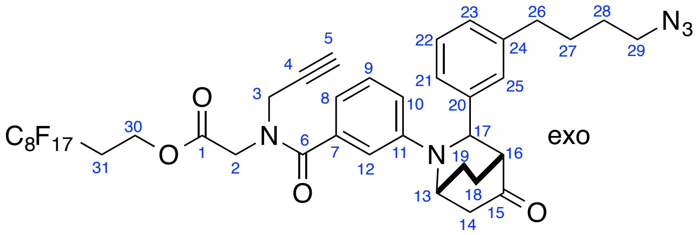

2 (136 mg, 0.193 mmol, 1.0 eq) was reacted according to GSP-3a using PBu_3 (58 µL, 0.23 mmol, 1.2 eq). **16a** (44 mg, 0.22 mmol, 1.12 eq) dissolved in THF (5 mL) was added. The reaction mixture was stirred at 40 °C for 5 h. $Yb(OTf)_3$ (120 mg, 0.193 mmol, 1.0 eq) and 2-cyclohexen-1-one (111 µL, 1.15 mmol, 6.0 eq) were added and the mixture stirred at 40 °C overnight. The solvent was removed under a stream of nitrogen and the residue dissolved in EtOAc. The organic phase was washed with sat. aq. $NaHCO_3$ and brine, dried over $MgSO_4$, filtered and concentrated under reduced pressure. After FCC (petroleum ether/EtOAc 3:1), **20** (24 mg, 0.025 mmol, 13%) was obtained as a colourless film.

TLC R_f = 0.23 (petroleum ether/EtOAc 3:1); **HPLC** t_r = 14.07 min (50-100% B), peak area 93%; **IR** ν_{max} (neat)/cm^{-1} = 2099 m (N_3), 1752 m (C=O), 1728 m (C=O), 1647 m, 1598 m, 1456 m, 1199 s (C-F), 1145 s (C-F); **^{1}H NMR** (500 MHz, DMSO-d_6, 120 °C) δ = 7.29 (t, J = 7.5 Hz, 1H; H-22), 7.25 (s, 1H; H-25), 7.23 (d, J = 7.7 Hz, 1H; H-21 or H-23), 7.15 (t, J = 8.0 Hz, 1H; H-9), 7.13 (d, J = 7.7 Hz, 1H; H-21 or H-23), 6.72-6.65 (m, 3H; H-8, H-10 and H-12), 4.81 (d, J = 2.7 Hz, 1H; H-17), 4.63-4.59 (m, 1H; H-13), 4.40 (t, J = 6.2 Hz, 2H; H-30), 4.16-4.04 (m, 4H; H-2 and H-3), 3.30 (t, J = 6.8 Hz, 2H; H-29), 3.00 (t, J = 2.4 Hz, 1H; H-5), 2.70-2.56 (m, 6H; H-31, H-26, H-16 and H-14a), 2.49-2.45 (m; H-14b, coincides with solvent signal), 2.20 (ddt, J = 13.4, 8.6, 2.8 Hz, 1H; H-19a), 1.99-1.91 (m, 1H; H-19b), 1.71-1.60 (m, 4H; H-18 and H-27), 1.60-1.53 (m, 2H; H-28); **HRMS** (ESI+) m/z = 960.2421 $[M+H]^+$ found, $C_{39}H_{35}F_{17}N_5O_4^+$ required 960.2412.

3,3,4,4,5,5,6,6,7,7,8,8,9,9,10,10,10-Heptadecafluorodecyl *N*-((4*aS,5*S**,10*bS**)-5-(3-(4-azidobutyl)phenyl)-3,4,4*a*,5,6,10*b*-hexahydro-2*H*-pyrano[3,2-*c*]quinoline-8-carbonyl)-*N*-(prop-2-yn-1-yl)glycinate (21a)**,

an inseparable mixture of **3,3,4,4,5,5,6,6,7,7,8,8,9,9,10,10,10-heptadecafluorodecyl *N*-((4*aR**,5*S**, 10*bR**)-5-(3-(4-azidobutyl)phenyl)-3,4,4*a*,5,6,10*b*-hexahydro-2*H*-pyrano[3,2-*c*] quinolone-8-carbonyl)-*N*-(prop-2-yn-1-yl)glycinate (21b)** and **3,3,4,4,5,5,6,6,7,7,8,8,9,9,10,10,10-heptadecafluorodecyl *N*-((4*aR**,5*R**,10*bR**)-5-(3-(4-azidobutyl)phenyl)-3,4,4*a*,5,6,10*b*-hexahydro-2*H*-pyrano[3,2-*c*]quinoline-10-carbonyl)-*N*-(prop-2-yn-1-yl)glycinate (21c)**

and **3,3,4,4,5,5,6,6,7,7,8,8,9,9,10,10,10-heptadecafluorodecyl *N*-((4*aS**,5*R**,10*bS**)-5-(3-(4-azidobutyl)phenyl)-3,4,4*a*,5,6,10*b*-hexahydro-2*H*-pyrano[3,2-*c*]quinoline-10-carbonyl)-*N*-(prop-2-yn-1-yl)glycinate (21d)**
Structures see below.

2 (451 mg, 0.640 mmol, 1.0 eq) was reacted according to GSP-3a using PBu_3 (192 µL, 0.77 mmol, 1.2 eq). **16a** (193 mg, 0.950 mmol, 1.5 eq) dissolved in THF (5 mL) was added. The reaction mixture was stirred at 40 ^{o}C for 5 h. $Yb(OTf)_3$ (496 mg, 0.800 mmol, 1.25 eq) and 3,4-dihydro-2*H*-pyran (233 µl, 2.56 mmol, 4.0 eq) were added and the mixture stirred at 40 ^{o}C for 5 h. The solvent was removed under a stream of nitrogen and the residue dissolved in EtOAc. The organic phase was washed with sat. aq. $NaHCO_3$ and brine, dried over $MgSO_4$, filtered and concentrated under reduced pressure. After FCC (petroleum ether/EtOAc 3:1), three fractions, **21a** (87 mg), an inseparable mixture of **21b** and **21c** (152 mg, an inseparable mixture of two isomers), and **21d** (71 mg) were all obtained as colourless waxes.

21a

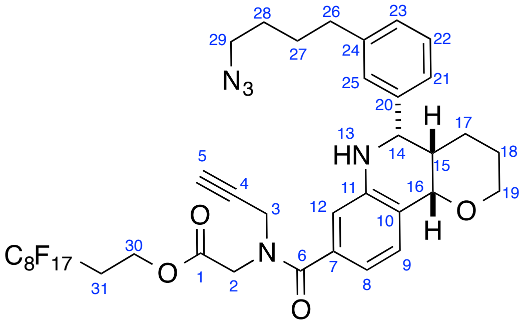

TLC R_f = 0.56 (petroleum ether/EtOAc 3:1); **HPLC** t_r = 15.12 min (50-100% B, without addition of TFA), peak area 81%; **IR** ν_{max} (neat)/cm^{-1} = 2093 m (N_3), 1753 m (C=O), 1643 m, 1452 m, 1199 s

(C-F), 1146 s (C-F); **^{1}H NMR** (500 MHz, DMSO-d_6, 120 °C) Clean NMR spectra could not be obtained due to the existence of rotamers at a temperature range of 25-70 °C and partial aromatisation at higher temperatures, δ = 7.33-7.23 (m, 4H; H-22, H-25, H-9 and H-21), 7.13 (d, J = 7.3 Hz, 1H; H-23), 6.80 (d, J = 1.5 Hz, 1H; H-12), 6.68 (dd, J = 7.8, 1.5 Hz, 1H; H-8), 5.76 (s, 1H; H-13), 5.24 (d, J = 5.6 Hz, 1H; H-16), 4.68 (d, J = 2.6 Hz, 1H; H-14), 4.45 (t, J = 6.2 Hz, 2H; H-30), 4.27 (apps, 2H, H-2 or H-3), 4.26 (apps, H-2 or H-3), 3.54-3.50 (m, 1H; H-19a), 3.35 (t, J = 6.8 Hz, 2H; H-29), 3.30 (dd, J = 11.3, 3.0 Hz, 1H; H-19b), 3.04 (t, J = 2.4 Hz, 1H; H-5), 2.76-2.59 (m, 4H; H-31 and H-26), 2.19-2.06 (m, 1H; H-15), 1.76-1.69 (m, 2H; H-27), 1.68-1.59 (m, 2H; H-28), 1.48-1.35 (m, 3H; H-17a and H-18), 1.24-1.16 (m, 1H; H-17b); **HRMS** (ESI+) m/z = 948.2452 $[M+H]^+$ found, $C_{38}H_{35}F_{17}N_5O_4^+$ required 948.2412.

Inseparable mixture of **21b** and **21c**

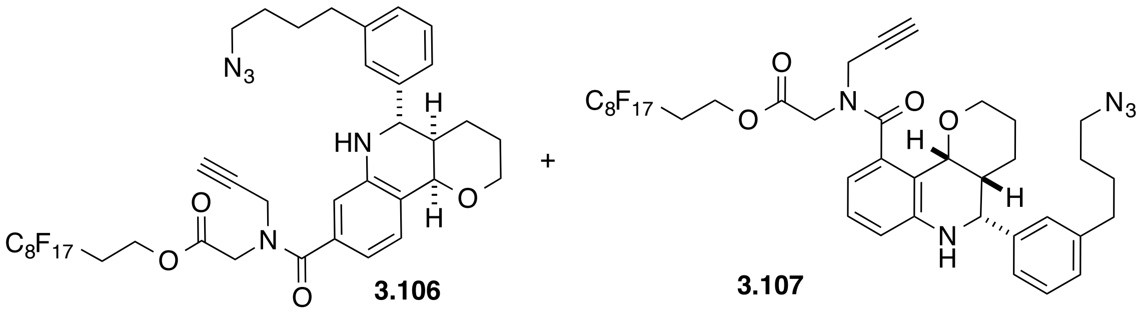

3.106 **3.107**

TLC R_f = 0.43 (petroleum ether/EtOAc 3:1); **HPLC** t_r = 14.97 min (50-100% B, without addition of TFA), peak area 83%; **LCMS** (ESI+) m/z = 948.3 $[M+H]^+$.

21d

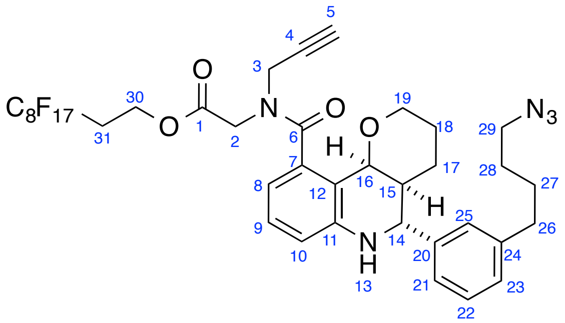

TLC R_f = 0.64 (petroleum ether/EtOAc 3:1); **HPLC** t_r = 15.08 min (50-100% B, without addition of TFA), peak area 98%; **IR** ν_{max} (neat)/cm^{-1} = 2093 m (N_3), 1757 m (C=O), 1647 m, 1595 m, 1448 m, 1202 s (C-F), 1147 s (C-F); **^{1}H NMR** (500 MHz, DMSO-d_6, 120 °C) Clean NMR spectra could not be

obtained due to the existence of rotamers at a temperature range of 25-90 °C and partial decomposition at higher temperatures, δ = 7.30 (t, J = 7.5 Hz, 1H; H-22), 7.27 (t, J = 1.5 Hz, 1H; H-25), 7.25 (dt, J = 7.6, 1.5 Hz, 1H; H-21), 7.17 (d, J = 7.4 Hz, 1H; H-23), 7.02 (brs, 1H; H-9), 6.70 (d, J = 8.1 Hz, 1H; H-10 or H-8), 6.35 (brs, 1H; H-8 or H-10), 5.82 (brs; H-13), 4.61 (d, J = 11.1 Hz, 1H; H-14), 4.50-4.38 (m, 3H; H-30 and H-16), 4.05 (brs, 2H; H-3), 3.93 (dd, J = 11.4, 4.3 Hz, 1H; H-19a), 3.60 (s, 2H; H-2), 3.59-3.57 (m, 1H; H-19b), 3.34 (t, J = 6.8 Hz, 2H; H-29), 2.97 (s, 1H; H-5), 2.65 (m, 4H; H-31 and H-26), 1.92 (dq, J = 8.0, 2.4 Hz, 1H; H-15), 1.84-1.76 (m, 1H; H-18a), 1.75-1.66 (m, 2H; H-27), 1.66-1.56 (m, 3H; H-28 and H-17a), 1.37 (d, J = 12.4 Hz, 1H; H-17b), 1.31-1.19 (m, 1H; H-18b); **HRMS** (ESI+) m/z = 948.2435 $[M+H]^+$ found, $C_{38}H_{35}F_{17}N_5O_4^+$ required 948.2412.

3,3,4,4,5,5,6,6,7,7,8,8,9,9,10,10,10-heptadecafluorodecyl N-(3-(((3-(4-azidobutyl)phenyl)(cyano)methyl)amino)benzoyl)-N-(prop-2-yn-1-yl)glycinate (22)

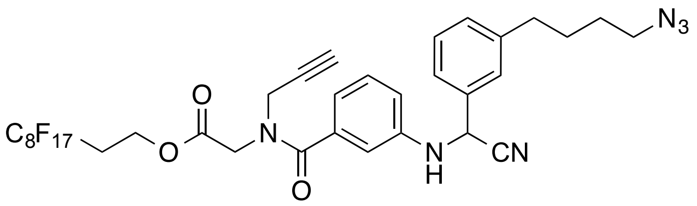

2 (0.10 g, 0.14 mmol, 1.0 eq) was reacted according to GSP-3a using PBu_3 (53 µL, 0.22 mmol, 1.5 eq). **16a** (43 mg, 0.22 mmol, 1.5 eq) was added and the reaction was stirred at 40 °C overnight. Upon completed turnover by LCMS, TMS-CN (72 µL, 0.56 mmol, 4.0 eq) and $Yb(OTf)_3$ (0.36 g, 0.56 mmol, 4.0 eq) were added, and the mixture was stirred at 55 °C overnight. After the solvent was removed by a flow of air, the residue was purified by F-SPE. **22** (0.068 g, not pure, max 0.076 mmol) was obtained as a white solid.

TLC R_f = 0.23 (PE/EtOAc 4:1); **HRMS** (ESI+) m/z = 891.1976 $[M+H]^+$ found, $C_{34}H_{28}F_{17}N_6O_3^+$ required 891.1951.

In the *pair* stage

3,3,4,4,5,5,6,6,7,7,8,8,9,9,10,10,10-Heptadecafluorodecyl 2-((9*S*,*Z*)-9-(dimethylcarbamoyl)-4,7-dioxo-11*H*-3,6,8-triaza-1(4,1)-triazola-5(1,3)-benzenacyclotridecaphane-3-yl)acetate (24a)

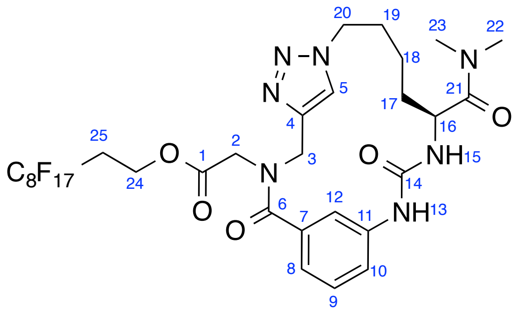

6 (62 mg, 0.069 mmol, 1.0 eq) was reacted according to GSP-6 overnight. After FCC (EtOAc), **24a** (43 mg, 0.048 mmol, 70%) was obtained as a yellow solid.

TLC R_f = 0.11 (EtOAc); **HPLC** t_r = 8.14 min (50-100% B), peak area 99%; **mp** 122 °C decomposition (H_2O); **IR** ν_{max} (neat)/cm^{-1} = 1750 w (C=O), 1635 m (C=O), 1548 m, 1422 w, 1200 s (C-F), 1146 s (C-F); **^{1}H NMR** (500 MHz, DMSO-d_6, 90 °C) δ = 8.60 (s, 1H; H-13), 8.00 (s, 1H; H-5), 7.92 (s, 1H; H-12), 7.31 (t, J = 7.8 Hz, 1H; H-9), 7.02 (dt, J = 7.6, 1.4 Hz, 1H; H-8), 6.85 (dd, J = 7.8, 2.4 Hz, 1H; H-10), 6.31 (d, J = 8.4 Hz, 1H; H-15), 4.74 (ddd, J = 11.2, 8.5, 2.6 Hz, 1H; H-16), 4.53-4.39 (m, 5H; H-3, H-24 and H-20a), 4.41-4.29 (m, 2H; H-20b and H-2a), 4.14 (d, J = 17.0 Hz, 1H; H-2b), 2.94 (brs; H-22 and H-23, coincides with water signal), 2.68 (tt, J = 18.9, 6.1 Hz, 2H; H-25), 2.40-2.30 (m, 1H; H-19a), 1.79-1.71 (m, 1H; H-19b), 1.71-1.62 (m, 1H; H-17a), 1.47 (ddt, J = 14.0, 10.7, 5.0 Hz, 1H; H-17b), 1.39-1.28 (m, 2H; H-18); **^{13}C NMR** (125 MHz, DMSO-d_6, 90 °C) due to interconversion, C-22 and C-23 are missing, δ = 171.2 × 2 (C-21 and C-6), 168.1 (C-1), 154.2 (C-14), 143.1 (C-4), 139.2 (C-11), 134.9 (C-7), 128.7 (C-9), 121.7 (C-5), 119.6 (C-8), 119.2 (C-10), 116.3 (C-12), 56.1 (C-24), 49.7 (C-20), 47.4 (C-2), 46.8 (C-16), 46.1 (C-3), 33.2 (C-17), 29.7 (t, J = 20.6 Hz; C-25), 27.8 (C-19), 22.0 (C-18); **HRMS** (ESI+) m/z = 926.1994 $[M+Na]^+$ found, $C_{31}H_{30}F_{17}N_7O_5Na^+$ required 926.1929.

3,3,4,4,5,5,6,6,7,7,8,8,9,9,10,10,10-Heptadecafluorodecyl (*S*)-2-(9-(dimethylcarbamoyl)-4,7-dioxo-11*H*-3,6,8-triaza-1(5,1)-triazola-5(1,3)-benzenacyclotridecaphane-3-yl)acetate (24b)

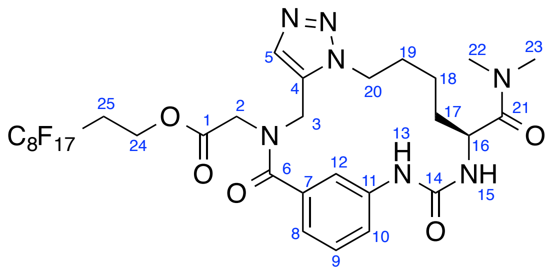

6 (87 mg, 0.096 mmol, 1.0 eq) was reacted according to GSP-7 for 6 h. After FCC (EtOAc then CH_2Cl_2/MeOH 15:1), **24b** (29 mg, 0.032 mmol, 33%) was obtained as a yellow solid.

TLC R_f = 0.34 (CH_2Cl_2/MeOH 15:1); **HPLC** t_r= 7.25 min (50-100% B), peak area 94%; **mp** 115 °C decomposition (H_2O); **IR** ν_{max} (neat)/cm^{-1} = 1747 w (C=O), 1638 m (C=O), 1403 w, 1201 s (C-F), 1146 s (C-F); **^{1}H NMR** (500 MHz, DMSO-d_6, 90 °C) δ = 8.25 (s, 1H; H-13), 7.69 (s, 1H; H-5), 7.45 (t, J = 1.8 Hz, 1H; H-12), 7.38 (t, J = 7.8 Hz, 1H; H-9), 7.18 (brs, 1H; H-8), 7.09 (dd, J = 8.1, 2.0 Hz, 1H; H-10), 6.17 (brs, 1H; H-15), 4.80 (brs, 2H; H-3), 4.58 (td, J = 8.9, 3.5 Hz, 1H; H-16), 4.42 (t, J = 6.1 Hz, 2H; H-24), 4.25-4.02 (m, 4H; H-2 and H-20), 2.93 (brs; H-22 and H-23, coincides with water signal), 2.66 (tt, J = 19.3, 6.2 Hz, 2H; H-25), 1.94 (ddt, J = 17.3, 12.7, 6.6 Hz, 1H; H-19a), 1.78 (ddt, J = 16.5, 13.5, 6.7 Hz, 1H; H-19b), 1.68 (dtd, J = 14.8, 7.6, 3.6 Hz, 1H; H-17a), 1.48 (dq, J = 14.3, 7.2 Hz, 1H; H-17b), 1.33-1.17 (m, 2H; H-18); **^{13}C NMR** (125 MHz, DMSO-d_6, 90 °C) due to interconversion, C-4, C-5, C-8, C-22 and C-23 are missing, δ = 171.1 (C-21), 170.0 (C-6), 168.0 (C-1), 155.3 (C-14), 139.3 (C-11), 134.3 (C-7), 129.0 (C-9), 123.5 (C-10), 119.1 (C-12), 56.3 (C-24), 49.2 (C-16), 48.0 (C-2), 47.3 (C-20), 30.6 (C-17), 29.6 (t, J = 21.0 Hz; C-25), 28.4 (C-19), 21.8 (C-18); **HRMS** (ESI+) m/z = 904.2114 $[M+H]^+$ found, $C_{31}H_{31}F_{17}N_7O_5^+$ required 904.2110.

3,3,4,4,5,5,6,6,7,7,8,8,9,9,10,10,10-Heptadecafluorodecyl 2-((9*S*,*E*)-9-(dimethylcarbamoyl)-15-iodo-4,7-dioxo-11*H*-3,6,8-triaza-1(4,1)-triazola-5(1,3)-benzenacyclotridecaphane-3-yl)acetate (24c)

and **3,3,4,4,5,5,6,6,7,7,8,8,9,9,10,10,10-heptadecafluorodecyl 2-((9*S*,*Z*)-9-(dimethylcarbamoyl)-4,7-dioxo-11*H*-3,6,8-triaza-1(4,1)-triazola-5(1,3)-benzenacyclotridecaphane-3-yl)acetate (24a)**

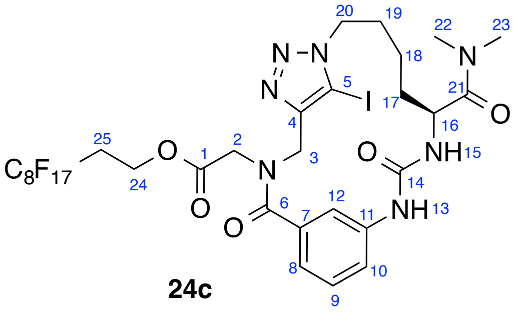

24c

and

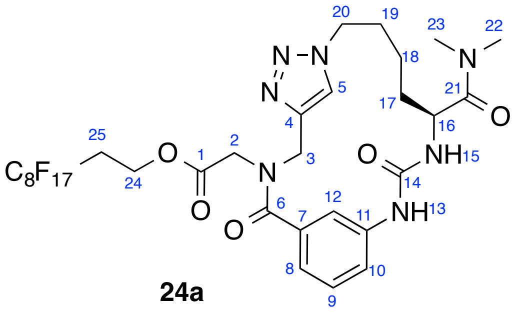

24a

6 (28 mg, 0.031 mmol, 1.0 eq) was dissolved in THF (30 mL), $Cu(ClO_4)_2 \cdot 6H_2O$ (10 µL, 0.062 mmol, 2.0 eq) and NaI (19 mg, 0.12 mmol, 4.0 eq) were added. After 5 min triethylamine (13 µL, 0.093 mmol, 3.0 eq) was added. The mixture was heated to reflux overnight. HPLC indicated only starting material left. $Cu(ClO_4)_2 \cdot 6H_2O$ (10 µL, 0.062 mmol, 2.0 eq), NaI (19 mg, 0.12 mmol, 4.0 eq) and triethylamine (13 µL, 0.093 mmol, 3.0 eq) were added and continue refluxing overnight. HPLC indicated only trace of product formed. Solvent was removed under a stream of nitrogen gas and re-dissolved in THF (15 mL). $Cu(ClO_4)_2 \cdot 6H_2O$ (10 µL, 62 µmol, 2.0 eq), NaI (19 mg, 0.12 mmol, 4.0 eq) and triethylamine (13 µL, 0.093 mmol, 3.0 eq) were added. The reaction mixture continued refluxing for one day. Solvent was removed and the residue purified by F-SPE followed by preparative HPLC (50-90% B), **24c** (7.3 mg, 7.1 µmol, 23%) and byproduct **24a** (9.1 mg, 0.010 mmol, 33%) were both obtained as white solid.

Product **24c**

TLC R_f = 0.18 (EtOAc); **HPLC** t_r = 8.63 min (50-100% B), peak area 100%; **IR** ν_{max} (neat)/cm^{-1} = 1621 m (C=O), 1462 w, 1201 s (C-F), 1146 s (C-F); **^{1}H NMR** (500 MHz, DMSO-d_6, 120 °C) δ = 8.45 (s, 1H; H-13), 7.54 (s, 1H; H-12), 7.29 (t, J = 7.8 Hz, 1H; H-9), 7.03 (d, J = 7.5 Hz, 1H; H-8), 6.84 (d, J = 7.8 Hz, 1H; H-10), 6.16 (d, J = 8.4 Hz, 1H; H-15), 4.63 (dt, J = 8.4, 2.7 Hz, 1H; H-16), 4.60-4.51 (m, 1H; H-2a), 4.54-4.49 (m, 1H; H-20a), 4.50-4.39 (m, 3H; H-24 and H-3a), 4.35 (d, J = 17.1 Hz, 1H; H-2b), 4.33-4.20 (m, 1H; H-20b), 2.93 (s; H-22 and H-23, coincides with water signal), 2.70 (tt, J = 19.2, 6.3 Hz, 2H; H-25), 2.29-2.11 (m, 1H; H-19a), 1.96-1.77 (m, 1H; H-19b), 1.76-1.56 (m, 1H; H-17a), 1.56-1.35 (m, 3H; H-17b and H-18); **^{13}C NMR** (125 MHz, DMSO-d_6, 27 °C) major rotamer signals only, δ = 171.5 × 2 (C-21 and C-6), 168.8 (C-1), 154.1 (C-14), 146.6 (C-4), 140.0 (C-11),

135.1 (C-7), 129.1 (C-9), 119.7 (C-8), 118.4 (C-10), 114.5 (C-12), 80.7 (C-5), 56.6 (C-24), 49.8 (C-20), 48.7 (C-2), 46.9 (C-16), 46.6 (C-3), 36.4 (C-22 or C-23), 35.0 (C-22 or C-23), 34.0 (C-17), 29.8 (C-19), 29.5 (t, *J* = 20.9 Hz; C-25), 23.2 (C-18); **HRMS** (ESI+) *m/z* = 1030.1043 $[M+H]^+$ found, $C_{31}H_{30}F_{17}N_7O_5{}^{127}I_1{}^+$ required 1030.1076.

Byproduct **24a**

TLC R_f = 0.12 (EtOAc); ^{1}**H NMR** (500 MHz, DMSO-d_6, 27 °C) δ = 8.79 (s, 1H; H-13), 8.11 (s, 1H; H-5), 7.96 (s, 1H; H-12), 7.31 (t, *J* = 7.8 Hz, 1H; H-9), 6.99 (d, *J* = 7.5 Hz, 1H; H-8), 6.82 (d, *J* = 8.0 Hz, 1H; H-10), 6.44 (d, *J* = 8.5 Hz, 1H; H-15), 4.70 (t, *J* = 9.9 Hz, 1H; H-16), 4.54-4.26 (m, 7H; H-3, H-24, H-20 and H-2a), 4.04 (d, *J* = 17.1 Hz, 1H; H-2b), 2.99 (s, 3H; H-22 or H-23), 2.85 (s, 3H; H-23 or H-22), 2.70 (t, *J* = 19.7 Hz, 2H; H-25), 2.38 (d, *J* = 13.1 Hz, 1H; H-19a), 1.78-1.51 (m, 2H; H-19b and H-17a), 1.49-1.35 (m, 1H; H-17b), 1.34-1.20 (m, 1H; H-18a), 1.20-1.03 (m, 1H; H-18b); ^{13}**C NMR** (125 MHz, DMSO-d_6, 27 °C) δ = 171.8 (C-6 or C-21), 171.5 (C-21 or C-6), 168.7 (C-1), 154.4 (C-14), 143.7 (C-4), 139.5 (C-7 or C-11), 135.0 (C-11 or C-7), 129.3 (C-9), 122.2 (C-5), 56.6 (C-24), 50.1 (C-20), 47.7 (C-2, C-3, C-16, C-22 or C-23), 46.8 (C-2, C-3, C-16, C-22 or C-23), 36.4 (C-2, C-3, C-16, C-22 or C-23), 35.0 (C-2, C-3, C-16, C-22 or C-23), 33.7 (C-17), 29.5 (t, *J* = 22.7 Hz; C-25), 28.1 (C-19), 22.3 (C-18); **LCMS** (ESI+) *m/z* = 904.2 $[M+H]^+$ found.

3,3,4,4,5,5,6,6,7,7,8,8,9,9,10,10,10-Heptadecafluorodecyl (*Z*)-2-(5,8,13-trioxo-21*H*-4,7,12,14-tetraaza-2(1,4)-triazola-1(1,4),6,11(1,3)-tribenzenacyclohexadecaphane-4-yl)acetate (26a)

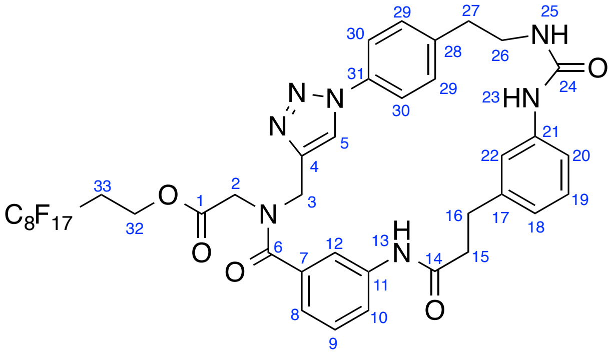

13 (25 mg, 0.025 mmol, 1.0 eq) was reacted according to GSP-6 for 1.5 days. After preparative HPLC (60-100% B), **26a** (15 mg, 0.015 mmol, 60%) was obtained as a white powder.

TLC R_f = 0.61 (CH_2Cl_2/MeOH 10:1); **HPLC** t_r = 8.70 min (60-100% B), peak area 98%; **mp** 195-200 °C (H_2O); **IR** ν_{max} (neat)/cm^{-1} = 1749 w (C=O), 1652 m (C=O), 1533 m, 1424 m, 1200 s (C-F), 1146 s (C-F); ^{1}**H NMR** (500 MHz, DMSO-d_6, 90 °C) δ = 9.81 (s, 1H; H-13), 8.53 (s, 1H; H-5), 7.95 (s,

1H; H-23), 7.83 (t, J = 1.8 Hz, 1H; H-12), 7.80 (d, J = 8.2 Hz, 2H; H-30), 7.49-7.42 (m, 3H; H-29 and H-10), 7.38 (t, J = 7.9 Hz, 1H; H-9), 7.13-7.06 (m, 3H; H-19, H-22 and H-8), 6.95 (appbrs, 1H; H-20), 6.77 (d, J = 7.5 Hz, 1H; H-18), 5.88 (s, 1H; H-25), 4.64 (s, 2H; H-3), 4.43 (t, J = 6.1 Hz, 2H; H-32), 4.31 (s, 2H; H-2), 3.50 (q, J = 5.5 Hz, 2H; H-26), 2.95-2.86 (m, 2H; H-27), 2.82 (t, J = 7.3 Hz, 2H; H-16), 2.67 (tt, J = 19.7, 6.3 Hz, 2H; H-33), 2.63 (t, J = 7.1 Hz, 2H; H-15); **^{13}C NMR** (125 MHz, DMSO-d_6, 90 °C) due to interconversion, C-5, C-15 and C-20 is missing, δ = 170.3 (C-6), 170.2 (C-14), 168.1 (C-1), 154.9 (C-24), 143.6 (C-4), 141.2 (C-17), 140.3 (C-28), 139.7 (C-21), 138.7 (C-11), 135.3 (C-7), 134.6 (C-31), 129.7 (C-29 × 2), 128.5 (C-9), 128.0 (C-19), 120.9 (C-18), 120.8 (C-8), 120.0 (C-10), 119.5 × 3 (C-30 × 2 and C-22), 117.1 (C-12), 56.2 (C-32), 39.2 (C-26, coincides with solvent signal), 34.5 (C-27), 30.1 (C-16), 29.60 (t, J = 21.1 Hz; C-33); **HRMS** (ESI+) m/z = 1014.2256 $[M+H]^+$ found, $C_{40}H_{33}F_{17}N_7O_5^+$ required 1014.2266.

3,3,4,4,5,5,6,6,7,7,8,8,9,9,10,10,10-Heptadecafluorodecyl 2-(5,8,13-trioxo-21*H*-4,7,12,14-tetraaza-2(1,5)-triazola-1(1,4),6,11(1,3)-tribenzenacyclohexadecaphane-4-yl)acetate (26b)

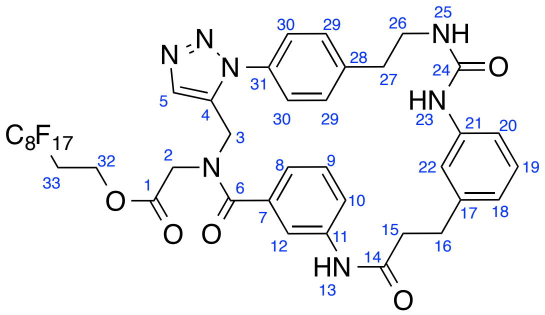

13 (39 mg, 0.038 mmol, 1.0 eq) was reacted according to GSP-7 for 5 hours. After FCC (petroleum ether/EtOAc 1:3), **26b** (21 mg, 0.021 mmol, 54%) was obtained as a pale brown foam.

TLC R_f = 0.31 (EtOAc); **HPLC** t_r = 9.27 min (50-100% B), peak area 99%; **mp** 98 °C decomposition (H_2O); **IR** ν_{max} (neat)/cm^{-1} = 1753 w (C=O), 1650 m (C=O), 1552 m, 1201 s (C-F), 1147 s (C-F); **^{1}H NMR** (500 MHz, DMSO-d_6, 120 °C) δ = 9.48 (s, 1H; H-13), 7.90 (s, 1H; H-5), 7.83 (s, 1H; H-23), 7.66 (t, J = 1.8 Hz, 1H; H-12), 7.44 (d, J = 8.1 Hz, 1H; H-10), 7.38-7.30 (m, 3H; H-29 and H-20), 7.25 (d, J = 8.0 Hz, 2H; H-30), 7.21 (t, J = 7.9 Hz, 1H; H-9), 7.10 (t, J = 7.8 Hz, 1H; H-19), 6.95 (s, 1H; H-22), 6.83 (d, J = 7.6 Hz, 1H; H-8), 6.78 (d, J = 7.5 Hz, 1H; H-18), 5.64 (t, J = 5.4 Hz, 1H; H-25), 4.64 (s, 2H; H-3), 4.39 (t, J = 6.2 Hz, 2H; H-32), 4.15 (s, 2H; H-2), 3.44 (q, J = 5.8 Hz, 2H; H-26), 2.90 (H-27 and H-16, coincides with water signal), 2.72-2.55 (m, 4H; H-15 and H-33); **^{13}C NMR** (125 MHz, DMSO-d_6, 27 °C) major rotamer signals only, δ = 171.4 (C-6), 170.6 (C-14), 168.8 (C-1), 155.0 (C-24), 141.7 (C-17 or C-28), 141.6 (C-17 or C-28), 140.3 (C-21), 139.6 (C-11), 135.3 (C-7 or

C-4), 135.2 (C-4 or C-7), 133.4 (C-31), 132.3 (C-5), 130.0 (C-29 × 2), 128.8 × 2 (C-19 and C-9), 124.6 (C-30 × 2), 121.5 (C-18), 120.4 (C-10), 119.4 (C-8), 117.1 (C-22), 116.6 (C-12), 115.2 (C-20), 56.8 (C-32), 47.6 (C-2), 45.5 (C-3), 39.2 (C-26, coincides with solvent signal), 37.9 (C-15), 33.8 (C-27), 30.0 (C-16), 29.47 (t, J = 20.7 Hz; H-33); **HRMS** (ESI+) m/z = 1036.2083 $[M+Na]^+$ found, $C_{40}H_{32}F_{17}N_7O_5Na^+$ required 1036.2086.

3,3,4,4,5,5,6,6,7,7,8,8,9,9,10,10,10-Heptadecafluorodecyl 2-((20*S*,*Z*)-20-(dimethylcarbamoyl)-4,7,12,18-tetraoxo-11*H*-3,6,11,13,17,19-hexaaza-1(4,1)-triazola-5,10(1,3),16(1,4)-tribenzenacyclotetracosaphane-3-yl)acetate (27a)

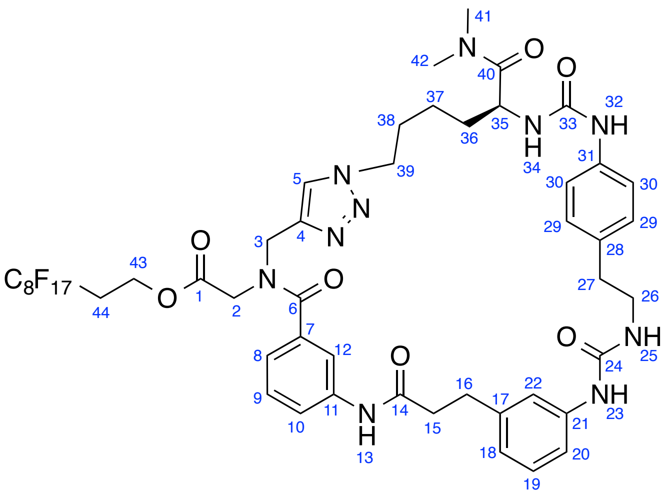

14 (22 mg, not pure, max. 0.018 mmol, 1.0 eq) was reacted according to GSP-6 for 1 day. After preparative HPLC (50-100% B), **27a** (6.3 mg, 5.2 µmol, 31%, over two steps from **13**) was obtained as a white solid.

TLC R_f = 0.56 (CH_2Cl_2/MeOH 10:1); **HPLC** t_r = 9.79 min (50-100% B), peak area 100%; **mp** 142 °C decomposition (H_2O); **IR** ν_{max} (neat)/cm^{-1} = 1749 w (C=O), 1631 m (C=O), 1547 m, 1201 s (C-F), 1147 s (C-F); **^{1}H NMR** (500 MHz, DMSO-d_6, 120 °C) δ = 9.57 (s, 1H; H-13), 8.30 (s, 1H; H-32), 7.96 (brs, 1H; H-23), 7.86 (s, 1H; H-5), 7.72 (d, J = 8.1 Hz, 1H; H-10), 7.54 (s, 1H; H-12), 7.32 (t, J = 7.9 Hz, 1H; H-9), 7.30-7.25 (m, 3H; H-30 × 2 and H-20), 7.13-7.03 (m, 5H; H-19, H-29 × 2, H-22 and H-8), 6.77 (d, J = 7.6 Hz, 1H; H-18), 6.19 (brs, 1H; H-34), 5.61 (brs, 1H; H-25), 4.72 (appbrs, 1H; H-35), 4.60 (s, 2H; H-3), 4.40 (t, J = 6.1 Hz, 2H; H-43), 4.32 (t, J = 7.0 Hz, 2H; H-39), 4.16 (s, 2H; H-2), 3.39 (q, J = 6.3 Hz, 2H; H-26), 2.97 (coincides with water signal; H-41 and H-42), 2.87 (t, J = 7.3 Hz; H-16, coincides with water signal), 2.70 (t, J = 6.0 Hz, 2H; H-27), 2.67-2.58 (m, 4H; H-15 and H-44), 1.94 (dt, J = 14.2, 6.8 Hz, 1H; H-38a), 1.87 (dt, J = 14.0, 7.0 Hz, 1H; H-38b), 1.72 (ddt, J = 13.4, 7.5,

4.7 Hz, 1H; H-36a), 1.53 (dq, J = 14.9, 7.6 Hz, 1H; H-36b), 1.38 (quint, J = 7.5 Hz, 2H; H-37); ^{13}**C NMR** (125 MHz, DMSO-d_6, 120 °C) due to interconversion, C-2 and C-3 are missing, δ = 171.4 (C-40), 170.1 (C-6), 170.0 (C-14), 167.9 (C-1), 154.6 (C-24 or C-33), 154.3 (C-24 or C-33), 141.8 (C-4), 141.0 (C-17), 140.0 (C-21), 138.8 (C-11), 137.8 (C-31), 135.4 (C-7), 132.0 (C-28), 128.2 (C-29 × 2), 128.0 (C-9), 127.7 (C-19), 122.4 (C-5), 120.7 (C-18), 120.5 (C-8), 120.2 (C-10), 117.8 (C-30 × 2), 117.6 (C-22), 117.3 (C-12), 115.6 (C-20), 56.1 (C-43), 48.8 (C-39), 48.0 (C-35), 39.6 (C-26, coincides with solvent signal), 37.2 (C-15), 35.2 (C-41 and C-42), 34.1 (C-27), 31.5 (C-36), 30.0 (C-16), 29.65 (t, J = 21.1 Hz; C-44), 28.5 (C-38), 21.4 (C-37); **HRMS** (ESI+) m/z = 607.1854 $[M+2H]^{2+}$ found, $C_{49}H_{51}F_{17}N_{10}O_7^{2+}$ required 607.1830.

3,3,4,4,5,5,6,6,7,7,8,8,9,9,10,10,10-Heptadecafluorodecyl (*S*)-2-(20-(dimethylcarbamoyl)-4,7,12,18-tetraoxo-11*H*-3,6,11,13,17,19-hexaaza-1(5,1)-triazola-5,10(1,3),16(1,4)-tribenzenacyclotetracosaphane-3-yl)acetate (27b)

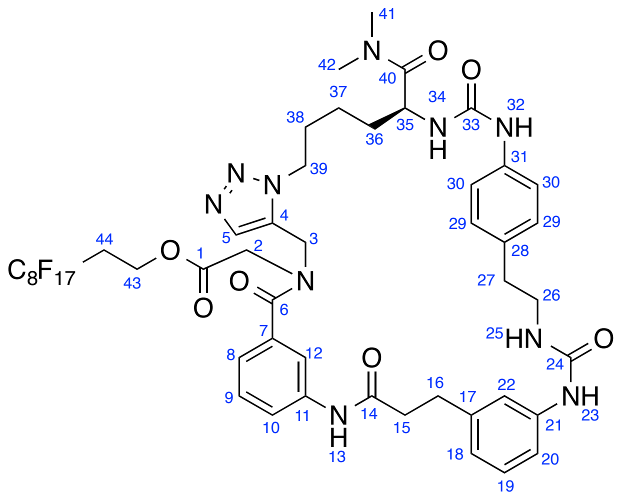

14 (88 mg, not pure, max. 0.073 mmol, 1.0 eq) was reacted according to GSP-7 for 1 day. After preparative HPLC (50-90% B), **27b** (13 mg, 0.011 mmol, 12%, over two steps from **13**) was obtained as a white solid.

TLC R_f = 0.43 (CH_2Cl_2/MeOH 10:1); **HPLC** t_r = 8.22 min (50-100% B), peak area 100%; **mp** 147 °C decomposition (H_2O); **IR** ν_{max} (neat)/cm^{-1} = 1646 m (C=O), 1547 m, 1202 s (C-F), 1148 m (C-F); ^{1}**H NMR** (500 MHz, DMSO-d_6, 120 °C) δ = 9.57 (s, 1H; H-13), 8.32 (s, 1H; H-32), 7.93 (s, 1H; H-23), 7.64 (s, 1H; H-5), 7.64-7.58 (m, 2H; H-10 and H-12), 7.29 (dd, J = 17.8, 9.5 Hz, 4H; H-9, H-30 × 2 and H-20), 7.13-7.03 (m, 3H; H-19 and H-29 × 2), 6.99 (d, J = 7.6 Hz, 1H; H-8), 6.95 (t, J = 1.8 Hz, 1H; H-22), 6.76 (d, J = 7.5 Hz, 1H; H-18), 6.20 (brs, 1H; H-34), 5.57 (brs, 1H; H-25), 4.80 (d, J =

16.3 Hz, 1H; H-3a), 4.76 (d, J = 16.2 Hz, 1H; H-3b), 4.72 (s, 1H; H-35), 4.34 (t, J = 6.2 Hz, 2H; H-43), 4.26 (t, J = 7.6 Hz, 2H; H-39), 4.10 (s, 2H; H-2), 3.41 (appq, J = 7.6 Hz, 2H; H-26), 2.97 (s; H-41 and H-42, coincides with water signal), 2.86 (t, J = 6.9 Hz, 2H; H-16), 2.70 (t, J = 5.9 Hz, 2H; H-27), 2.66-2.53 (m, 4H; H-15 and H-44), 1.95-1.77 (m, 2H; H-38), 1.71 (dt, J = 12.8, 7.8 Hz, 1H; H-36a), 1.53 (dt, J = 14.5, 8.0 Hz, 1H; H-36b), 1.39 (quint, J = 7.5 Hz, 2H; H-37); **^{13}C NMR** (125 MHz, DMSO-d_6, 120 °C) major rotamer signals only, due to interconversion, C-3 is missing, δ = 171.8 (C-40), 171.2 (C-6), 170.5 (C-14), 168.7 (C-1), 154.9 (C-24), 154.6 (C-33), 141.8 (C-17), 140.4 (C-21), 139.5 (C-11), 138.4 (C-31), 135.4 (C-7), 134.0 (C-5), 132.5 (C-4), 132.0 (C-28), 129.1 (C-29 × 2), 128.9 (C-9), 128.6 (C-19), 121.2 (C-18), 120.2 (C-8), 119.9 (C-10), 117.6 (C-30 × 2), 117.1 (C-22), 116.7 (C-12), 115.1 (C-20), 57.0 (C-43), 50.1 (C-2), 48.2 (C-35), 47.3 (C-39), 39.8 (C-26, coincides with solvent signal), 37.6 (C-15 and C-3), 36.6 (C-41 or C-42), 35.1 (C-42 or C-41), 34.4 (C-27), 32.3 (C-36), 29.9 (C-16), 29.5 (C-38), 29.2 (C-44), 22.3 (C-37); **HRMS** (ESI+) m/z = 1213.3568 $[M+H]^+$ found, $C_{49}H_{50}F_{17}N_{10}O_7^+$ required 1213.3587.

3,3,4,4,5,5,6,6,7,7,8,8,9,9,10,10,10-Heptadecafluorodecyl (*Z*)-2-(6-(2-(cyclohexylamino)-2-oxo-1-phenylethyl)-4,7-dioxo-1^1*H*-3,6-diaza-1(4,1)-triazola-5(1,3)-benzenacyclotetradecaphane-3-yl)acetate (28a)

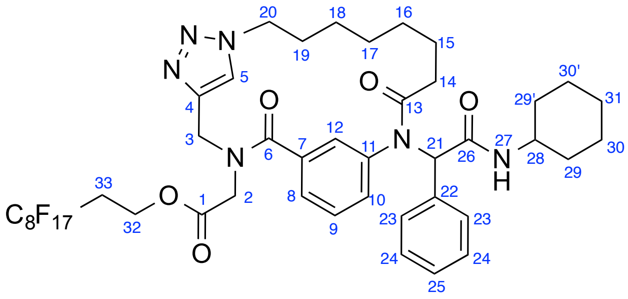

17a (75 mg, not pure, max 0.071 mmol) was reacted according to GSP-6 overnight. After preparative HPLC (70-100% B), **28a** (19 mg, 0.018 mmol, 18% over two steps from **2**) was obtained as a white solid.

TLC R_f = 0.65 (CH_2Cl_2/MeOH 40:1); **HPLC** t_r = 8.72 min (70-100% B), peak area 100%; **IR** ν_{max} (neat)/cm^{-1} = 3310 w (N-H), 1751 m (C=O), 1638 m (C=O); **^{1}H NMR** (500 MHz, DMSO-d_6, 120 °C) δ = 7.92 (s, 1H; H-5), 7.33 (m, 2H; H-12, H-27), 7.27-7.18 (m, 3H; H-8, H-9, H-10), 7.19-7.05 (m, 1H; H-25), 7.15-7.12 (m, 2H; H-24), 7.12-7.05 (m, 2H; H-23), 6.10 (s, 1H; H-21), 4.61 (d, J = 16.0 Hz, 1H; H-3a), 4.57 (d, J = 16.1 Hz, 1H; H-3b), 4.44 (t, J = 6.2 Hz, 2H; H-32), 4.39 (t, J = 6.1 Hz, 2H; H-20), 4.15 (d, J = 17.5 Hz, 1H; H-2a), 4.04 (d, J = 17.2 Hz, 1H; H-2b), 3.66 (brs, coincides with water

signal; H-28), 2.67 (tt, J = 6.4, 18.9 Hz, 2H; H-33), 1.90 (t, J = 6.9 Hz, 2H; H-14), 1.87-1.76 (m, 3H; H-19 and either H-29a or H-29a), 1.75-1.67 (m, 2H; either H-29a or H-29'a, and either H-30a or H-30'a), 1.66-1.59 (m, 1H; H-30a or H-30'a), 1.58-1.52 (m, 1H; H-31a), 1.43-1.34 (m, 2H; H-15), 1.34-1.22 (m, 3H; H-30b and H-30'b, and either H-29b or H-29'b), 1.21-0.93 (m, 8H; H-31b, H-16, H-17, H-18, and either H-29b or H-29'b); **^{13}C NMR** (125 MHz, DMSO-d_6 ,120 °C) δ = 172.4 (C-13), 170.2 (C-6), 169.0 (C-26), 168.8 (C-1), 143.5 (C-4), 140.8 (C-11), 136.1 (C-22), 135.8 (C-7), 132.3 (C-8 or C-10), 130.3 (C-23 × 2), 129.6 (C-12), 128.9 (C-9), 128.1 (C-24 × 2), 127.8 (C-25), 126.3 (C-8 or C-10), 123.3 (C-5), 64.5 (C-21), 57.2 (C-32), 49.8 (C-20), 48.5 (C-28), 34.2 (C-14), 32.5 (C-29 or C-29'), 32.4 (C-29 or C-29'), 30.7 (t, J = 17.5 Hz; C-33), 28.9 (C-19), 28.4 (C-16), 27.5 (C-17), 25.7 (C-18), 25.6 (C-31), 24.9 (C-15), 24.7 (C-30 or C-30'), 24.6 (C-30 or C-30'); **HRMS** (ESI+) m/z = 1061.3265 $[M+H]^+$ found, $C_{44}H_{46}F_{17}N_6O_5^+$ required 1061.3298.

3,3,4,4,5,5,6,6,7,7,8,8,9,9,10,10,10-heptadecafluorodecyl 2-(6-(2-(cyclohexylamino)-2-oxo-1-phenylethyl)-4,7-dioxo-1^1*H*-3,6-diaza-1(5,1)-triazola-5(1,3)-benzenacyclotetradecaphane-3-yl)acetate (28b)

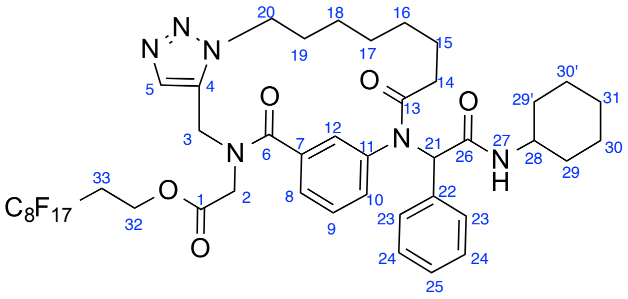

17a (0.10 g, not pure, max 0.094 mmol) was reacted according to GSP-7 for 2 days using $[RuCp^*Cl]_4$ (0.2 eq). After preparative HPLC (70-100% B), **28b** (70 mg, 0.066 mmol, 47% over two steps from **2**) was obtained as a yellow solid.

HPLC t_r = 8.59 min (70-100% B), peak area 100%; **IR** ν_{max} (neat)/cm^{-1} = 3287 w (N-H), 1756 m (C=O), 1647 m (C=O); **^{1}H NMR** (500 MHz, DMSO-d_6, 120 °C) δ = 7.67 (s, 1H; H-5), 7.46-7.30 (m, 2H; H-12, H-27), 7.30-7.14 (m, 3H; H-8, H-9, H-10), 7.14-7.10 (m, 3H; H-24, H-25), 7.09-7.05 (m, 2H; H-23), 6.14 (s, 1H; H-21), 4.74 (m, 2H; H-3), 4.40 (t, J = 6.2 Hz, 2H; H-32), 4.18 (t, J = 7.1 Hz, 2H; H-20), 4.07 (d, J = 17.5 Hz, 1H; H-2a), 3.99 (d, coincides with water signal; H-2b), 3.62 (m, 1H; H-28), 2.64 (tt, J = 19.0, 6.2 Hz, 2H; H-33), 1.94 (t, J = 7.5 Hz, 2H; H-14), 1.82-1.64 (m, 5H; H-19, H-29a, H-29'a, and either H-30a or H-30'a), 1.64-1.57 (m, 1H; H-30a or H-30'a), 1.57-1.50 (m, 1H; H-31a), 1.48-1.36 (m, 2H; H-15), 1.34-1.20 (m, 3H; H-30b, H-30'b, and either H-29b or H-29'b),

1.20-1.06 (m, 6H; H-16, H-18, H-31b, and either H-29b or H-29’b), 1.06-0.95 (m, 2H; H-17); **^{13}C NMR** (125 MHz, DMSO-d_6, 120 °C) δ = 171.4 (C-13), 169.4 (C-6), 167.8 (C-26), 167.5 (C-1), 139.7 (C-11), 135.2 (C-22), 134.1 (C-7), 132.9 (C-5), 132.2 (C-12), 131.7 (C-4), 129.3 (C-23 × 2), 128.4 (C-9), 128.2 (C-8 or C-10), 127.1 (C-24 × 2), 126.9 (C-25), 125.9 (C-8 or C-10), 63.0 (C-21), 56.3 (C-32), 47.5 (C-20), 46.9 (C-28), 33.2 (C-14), 31.4 × 2 (C-29 and C-29’), 29.6 (t, J = 16.3 Hz; C-33), 28.0 (C-19), 26.4 (C-16), 26.2 (C-17), 24.6 (C-31), 24.2 (C-18), 23.7 (C-15 and either C-30 or C-30’), 23.6 (C-30 or C-30’); **HRMS** (ESI+) m/z = 1061.3237 $[M+H]^+$ found, $C_{44}H_{46}F_{17}N_6O_5^+$ required 1061.3258.

3,3,4,4,5,5,6,6,7,7,8,8,9,9,10,10,10-Heptadecafluorodecyl (*Z*)-2-(6-(1-(4-bromophenyl)-2-(cyclohexylamino)-2-oxoethyl)-4,7-dioxo-1^1*H*-3,6-diaza-1(4,1)-triazola-5(1,3)-benzenacyclotetradecaphane-3-yl)acetate (29a)

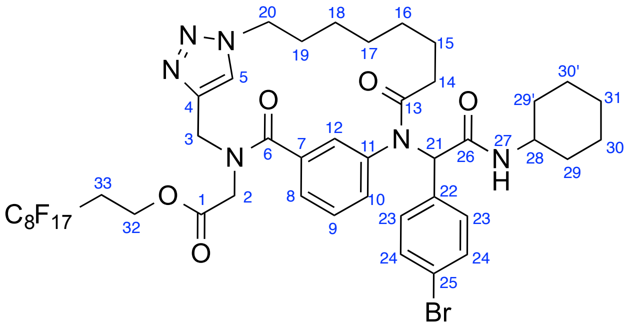

17b (80 mg, not pure, max 0.070 mmol) was reacted according to GSP-6 overnight. After preparative HPLC (70-100% B), **29a** (26 mg, 0.023 mmol, 24% over two steps from **2**) was obtained as a white powder.

TLC R_f = 0.64 (CH_2Cl_2/MeOH 40:1); **HPLC** t_r = 13.39 min (50-100% B), peak area 100%; **IR** ν_{max} (neat)/cm^{-1} = 3274 w (N-H), 1751 m (C=O), 1648 m (C=O); **^{1}H NMR** (500 MHz, DMSO-d_6, 120 °C) δ = 7.92 (s, 1H; H-5), 7.44 (brs, 1H; H-27), 7.38-7.30 (m, 3H; H-12, H-24), 7.30-7.22 (m, 2H; H-8, H-10), 7.19 (brs, 1H; H-9), 7.02 (d, J = 7.9 Hz, 2H; H-23), 6.05 (s, 1H; H-21), 4.59 (brs, 2H; H-3), 4.43 (t, J = 6.1 Hz, 2H; H-32), 4.38 (t, J = 5.7 Hz, 2H; H-20), 4.16 (d, J = 17.2 Hz, 1H; H-2a), 4.08 (d, J = 17.3 Hz, 1H; H-2b), 3.62 (brs, 1H; H-28), 2.67 (tt, J = 6.1, 19.0 Hz, 2H; H-33), 1.88 (t, J = 6.7 Hz, 2H; H-14), 1.85-1.73 (m, 3H; H-19 and either H-29a or H-29’a), 1.74-1.66 (m, 2H; either H-29a or H-29’a, and either H-30a or H-30’a), 1.66-1.59 (m, 1H; H-30a or H-30’a), 1.57-1.51 (m, 1H; H-31a), 1.41-1.33 (m, 2H; H-15), 1.33-1.20 (m, 3H; H-30b and H-30’b, and either H-29b or H-29’b), 1.20-0.92 (m, 8H; H-31b, H-16, H-17, H-18, and either H-29b or H-29’b); **^{13}C NMR** (125 MHz, DMSO-d_6, 120 °C) δ = 172.4 (C-13), 170.1 (C-6), 168.7 (C-1), 168.5 (C-26), 143.5 (C-4), 140.6 (C-11), 136.0 (C-7),

135.6 (C-22), 132.3 (C-23 × 2), 132.3 (C-9), 131.1 (C-24 × 2), 129.6 (C-12), 129.1 (C-8 or C-10), 126.5 (C-8 or C-10), 123.3 (C-5), 121.4 (C-25), 63.8 (C-21), 57.2 (C-32), 49.9 (C-20), 48.6 (C-28), 34.1 (C-14), 32.4 (C-29 or C-29'), 32.3 (C-29 or C-29'), 30.7 (t, J = 17.5 Hz; C-33), 28.8 (C-19), 28.3 (C-16), 27.4 (C-17), 25.8 (C-18), 25.6 (C-31), 24.8 (C-15), 24.7 (C-30 or C-30'), 24.6 (C-30 or C-30'); **HRMS** (ESI+) m/z = 1139.2422 $[M+H]^+$ found, $C_{44}H_{45}BrF_{17}N_6O_5^+$ required 1139.2449.

3,3,4,4,5,5,6,6,7,7,8,8,9,9,10,10,10-Heptadecafluorodecyl 2-(6-(1-(4-bromophenyl)-2-(cyclohexylamino)-2-oxoethyl)-4,7-dioxo-1^1*H*-3,6-diaza-1(5,1)-triazola-5(1,3)-benzenacyclotetradecaphane-3-yl)acetate (29b)

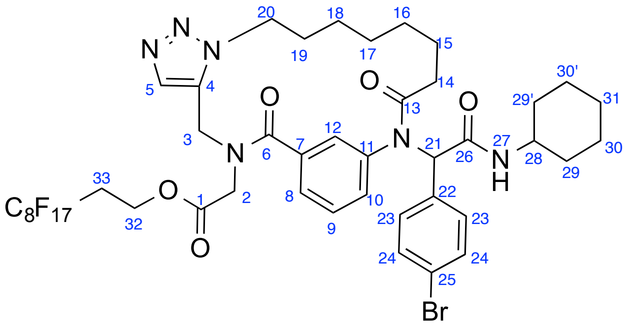

17b (0.10 g, not pure, max 0.088 mmol) was reacted according to GSP-7 overnight. After preparative HPLC (70-100% B), **29b** (94 mg, 0.082 mmol, 78% over two steps from **2**) was obtained as a yellow solid.

HPLC t_r = 13.10 min (50-100% B), peak area 100%; **IR** ν_{max} (neat)/cm^{-1} = 3293 w (N-H), 1752 m (C=O), 1646 m (C=O); **^{1}H NMR** (500 MHz, DMSO-d_6, 120 °C) δ = 7.68 (s, 1H; H-5), 7.57-7.11 (m, 7H; H-8, H-10, H-27, H-24, H-9 and H-12), 7.03 (d, J = 8.6 Hz, 2H; H-23), 6.09 (s, 1H; H-21), 4.79 (d, J = 15.8 Hz, 1H; H-3a), 4.72 (d, J = 16.4 Hz, 1H; H-3b), 4.41 (t, J = 6.1 Hz, 2H; H-32), 4.18 (t, J = 6.9 Hz, 2H; H-20), 4.11 (d, J = 17.8 Hz, 1H; H-2a), 4.04 (d, J = 17.6 Hz, 1H; H-2b), 2.65 (tt, J = 6.0, 19.1 Hz, 2H; H-33), 1.95 (t, J = 7.3 Hz, 2H; H-14), 1.81-1.64 (m, 5H; H-19, H-29a, H-29'a, and either H-30a or H-30'a), 1.64-1.57 (m, 1H; either H-30a or H-30'a), 1.57-1.50 (m, 1H; H-31a), 1.48-1.35 (m, 2H; H-15), 1.32-1.23 (m, 3H; H-30b and H-30'b, and either H-29b or H-29b'), 1.22-1.06 (m, 6H; H-16, H-18, H-31b, and either H-29b or H-29b'), 1.04-0.95 (m, 2H; H-17); **^{13}C NMR** (125 MHz, DMSO-d_6, 120 °C) δ = 171.4 (C-13), 169.4 (C-6), 167.5 (C-1), 167.4 (C-26), 139.5 (C-11), 134.7 (C-22), 134.4 (C-7), 132.8 (C-5), 132.1 (C-12), 131.7 (C-4), 131.3 (C-23), 130.1 (C-24), 128.3 (C-9), 126.0 (C-8 or C-10), 120.4 (C-25), 62.4 (C-21), 56.3 (C-32), 47.5 (C-28), 47.3 (C-2), 46.9 (C-20), 33.2 (C-14), 31.4 (C-29 or C-29'), 31.3 (C-29 or C-29'), 29.6 (t, J = 17.0 Hz; C-33), 27.9 (C-19), 26.3 (C-16),

26.1 (C-17), 24.5 (C-31), 24.2 (C-18), 23.7 (C-15 or C-30 or C-30'), 23.6 × 2 (C-15 and either C-30 or C-30'); **HRMS** (ESI+) m/z = 1139.2385 $[M+H]^+$ found, $C_{44}H_{45}BrF_{17}N_6O_5^+$ required 1139.2363.

3,3,4,4,5,5,6,6,7,7,8,8,9,9,10,10,10-Heptadecafluorodecyl 2-((22*S,23*R**,*Z*)-23-methoxy-24,4-dioxo-71*H*-5-aza-7(4,1)-triazola-2(2,1)-azetidina-1,3(1,3)-dibenzenacycloundecaphane-5-yl)acetate (30a)**

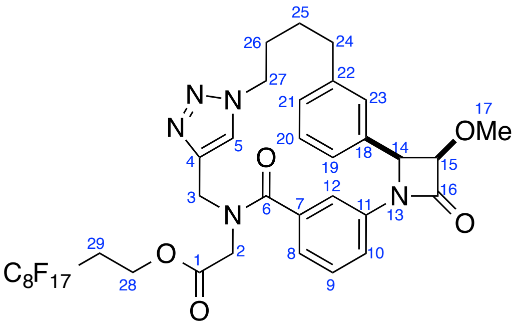

18a (79 mg, 0.084 mmol, 1.0 eq) was reacted according to GSP-6 for 1 day. After preparative HPLC (60-100% B), **30a** (48 mg, 0.051 mmol, 61%) was obtained as a colourless foam.

TLC R_f = 0.39 (petroleum ether/EtOAc 1:3); **HPLC** t_r = 11.14 min (50-100% B), peak area 100%; **mp** 180-185 °C (H_2O); **IR** ν_{max} (neat)/cm^{-1} = 1752 m (C=O), 1644 m (C=O), 1454 m, 1370 m, 1200 s (C-F), 1146 s (C-F); **^{1}H NMR** (500 MHz, DMSO-d_6, 120 °C) δ = 7.88 (d, J = 8.1 Hz, 1H; H-10), 7.54 (s, 1H; H-5), 7.40 (t, J = 7.9 Hz, 1H; H-9), 7.27-7.17 (m, 2H; H-20 and H-23), 7.16-7.06 (m, 3H; H-19, H-21 and H-8), 6.61 (s, 1H; H-12), 5.30 (d, J = 5.0 Hz, 1H; H-14), 4.95 (d, J = 5.0 Hz, 1H; H-15), 4.65 (d, J = 15.6 Hz, 1H; H-3a), 4.59 (d, J = 15.6 Hz, 1H; H-3b), 4.44-4.28 (m, 4H; H-27 and H-28), 3.99 (apps, 2H; H-2), 3.12 (s, 3H; H-17), 2.72-2.54 (m, 4H; H-29 and H-24), 1.91-1.67 (m, 2H; H-26), 1.50-1.38 (m, 1H; H-25a), 1.39-1.30 (m, 1H; H-25b); **^{13}C NMR** (125 MHz, DMSO-d_6, 120 °C) due to interconversion, C-3 is missing, δ = 169.2 (C-6), 167.7 (C-1), 163.9 (C-16), 142.3 (C-4), 141.6 (C-22), 136.8 (C-7 or C-11), 135.8 (C-7 or C-11), 133.2 (C-18), 128.7 (C-9), 128.0 (C-23), 127.8 (C-21), 127.4 (C-20), 124.4 (C-19), 122.2 (C-5), 121.1 (C-8), 118.7 (C-10), 113.5 (C-12), 84.1 (C-15), 60.8 (C-14), 57.0 (C-17), 56.2 (C-28), 49.0 (C-2), 48.4 (C-27), 33.5 (C-24), 29.6 (t, J = 29.6 Hz; C-29), 28.2 (C-26), 27.2 (C-25); **HRMS** (ESI+) m/z = 936.2025 $[M+H]^+$ found, $C_{36}H_{31}F_{17}N_5O_5^+$ required 936.2048.

3,3,4,4,5,5,6,6,7,7,8,8,9,9,10,10,10-Heptadecafluorodecyl 2-((22*S,23*R**)-23-methoxy-24,4-dioxo-71*H*-5-aza-7(5,1)-triazola-2(2,1)-azetidina-1,3(1,3)-dibenzenacycloundecaphane-5-yl)acetate (30b)**

and **3,3,4,4,5,5,6,6,7,7,8,8,9,9,10,10,10-heptadecafluorodecyl *N*-(3-((2*S**,3*R**)-2-(3-(3,4-dihydro-2*H*-pyrrol-5-yl)phenyl)-3-methoxy-4-oxoazetidin-1-yl)benzoyl)-*N*-(prop-2-yn-1-yl)glycinate (30c)**

18b (93 mg, 0.10 mmol, 1.0 eq) was reacted with catalyst [Cp*RuCl]$_4$ (0.2 eq) according to GSP-7 for 2 days. After preparative HPLC (60-100% B), **30b** (31 mg, 0.033 mmol, 33%) was obtained as a colourless foam. **30c** (5.7 mg, 6.3 µmol, 6%) was obtained as an off-white solid as a side product.

Product **30b**

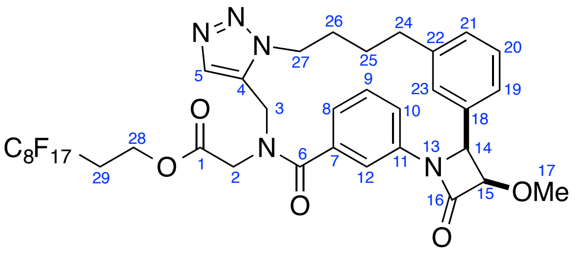

TLC R_f = 0.42 (petroleum ether/EtOAc 1:3); **HPLC** t_r = 10.77 min (50-100% B), peak area 96%; **mp** 70 °C decomposition (H_2O); **IR** ν_{max} (neat)/cm^{-1} = 1752 m (C=O), 1145 s (C-F), 703 m; **^{1}H NMR** (500 MHz, DMSO-d_6, 120 °C) due to interconversion, H-12 and H-2a are missing, δ = 7.60 (s, 1H; H-5), 7.35 (t, J = 7.9 Hz, 1H, H-9), 7.28-7.19 (m, 3H; H-20, H-21 and H-23), 7.19-7.07 (m, 2H; H-10 and H-19), 7.00 (dt, J = 7.6, 1.3 Hz, 1H; H-8), 5.39 (brs, 1H; H-14), 5.00 (d, J = 4.9 Hz, 1H; H-15), 4.60 (brs, 1H; H-3a), 4.48 (d, J = 16.1 Hz, 1H; H-3b), 4.39-4.28 (m, 2H; H-28), 4.13 (appbrs, 2H; H-27), 3.91 (appbrs, 1H; H-2b), 3.18 (s, 3H; H-17), 2.73-2.52 (m, 4H, coincides with solvent signal; H-24 and H-29), 1.53 (brs, 4H; H-25 and H-26); **^{13}C NMR** (125 MHz, DMSO-d_6, 27 °C) major rotamer signals only, δ = 171.1 (C-6), 168.4 (C-1), 163.9 (C-16), 142.0 (C-18 or C-22), 136.5 (C-7 or C-11), 135.4 (C-5), 135.2 (C-7 or C-11), 133.2 (C-18 or C-22), 132.1 (C-4), 131.0 (C-23), 129.8 (C-9), 128.7 (C-20 or C-21), 128.5 (C-20 or C-21), 124.6 (C-19), 121.9 (C-8), 119.5 (C-10), 113.3 (C-12), 84.1 (C-15), 60.6 (C-14), 57.7 (C-17), 56.9 (C-28), 50.3 (C-2), 47.5 (C-27), 37.7 (C-3), 34.7 (C-24), 29.5 (C-29), 28.6 (C-25), 25.6 (C-26); **HRMS** (ESI+) m/z = 936.2018 [M+H]$^+$ found, $C_{36}H_{31}F_{17}N_5O_5^+$ required 936.2048.

Side product **30c**

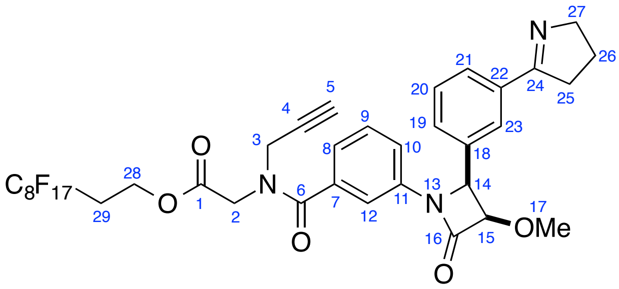

TLC R_f = 0.25 (petroleum ether/EtOAc 1:1); **HPLC** t_r = 11.66 min (50-100% B), peak area 92%; **mp** 56 °C decomposition (H_2O); **IR** ν_{max} (neat)/cm^{-1} = 1752 m (C=O), 1649 w, 1455 w, 1375 w, 1198 s (C-F), 1145 s (C-F); ^{1}**H NMR** (500 MHz, DMSO-d_6, 90 °C) δ = 7.38 (t, J = 1.6 Hz, 1H; H-12), 7.38 (t, J = 7.7 Hz, 1H; H-9), 7.34-7.31 (m, 1H; H-10), 7.31 (t, J = 7.1 Hz, 1H; H-20), 7.24 (t, J = 1.7 Hz, 1H; H-23), 7.22-7.18 (m, 2H; H-19 and H-21), 7.11 (dt, J = 7.5, 1.6 Hz, 1H; H-8), 5.44 (d, J = 5.0 Hz, 1H; H-14), 4.99 (d, J = 5.0 Hz, 1H; H-15), 4.40 (t, J = 6.1 Hz, 2H; H-28), 4.16 (s, 2H; H-2), 4.13 (s, 2H; H-3), 3.13 (s, 3H; H-17), 3.10 (t, J = 2.4 Hz, 1H; H-5), 2.70 (t, J = 7.6 Hz, 2H; H-25), 2.64 (tt, J = 19.3, 6.1 Hz, 2H; H-29), 2.39 (td, J = 7.1, 1.2 Hz, 2H; H-27), 1.86 (appquint, J = 7.2 Hz, 2H; H-26); ^{13}**C NMR** (125 MHz, DMSO-d_6, 27 °C) major rotamer signals only, δ = 169.7 (C-6), 168.2 (C-1), 164.5 (C-16), 140.5 (C-22), 137.1 (C-7 or C-11), 135.4 (C-11 or C-7), 133.7 (C-18), 129.6 (C-9), 128.6 (C-20), 128.4 (C-21), 127.9 (C-23), 125.6 (C-19), 122.1 (C-8), 120.3 (C-24), 118.6 (C-10), 115.4 (C-12), 84.5 (C-15), 78.5 (C-4), 76.0 (C-5), 60.7 (C-14), 57.7 (C-17), 56.8 (C-28), 46.6 (C-2), 40.4 (C-3), 33.8 (C-25), 29.4 (t, J = 20.3 Hz; C-29), 26.5 (C-26), 15.6 (C-27); **HRMS** (ESI+) m/z = 906.1815 $[M+H]^+$ found, $C_{36}H_{29}F_{17}N_3O_5^+$ required 906.1830.

3,3,4,4,5,5,6,6,7,7,8,8,9,9,10,10,10-Heptadecafluorodecyl 2-((22*R,23*R**,*Z*)-23-methoxy-24,4-dioxo-71*H*-5-aza-7(4,1)-triazola-2(2,1)-azetidina-1,3(1,3)-dibenzenacycloundecaphane-5-yl)acetate (31a)**

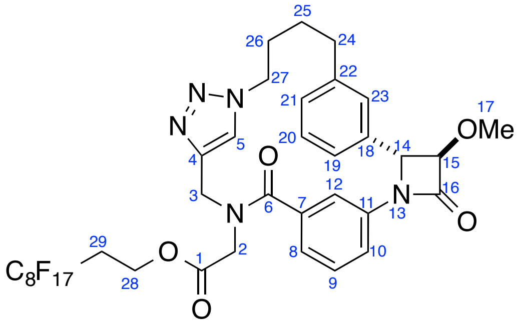

18b (54 mg, 0.058 mmol, 1.0 eq) was reacted according to GSP-6 for 1 day. After preparative HPLC (60-100% B), **31a** (49 mg, 0.052 mmol, 91%) was obtained as a colourless foam.

TLC R_f = 0.48 (petroleum ether/EtOAc 1:3); **HPLC** t_r = 11.39 min (50-100% B), peak area 100%; **mp** 70 °C decomposition (H_2O); **IR** ν_{max} (neat)/cm^{-1} = 1756 m (C=O), 1645 m (C=O), 1455 m, 1370 m, 1200 s (C-F), 1147 s (C-F); **^{1}H NMR** (500 MHz, DMSO-d_6, 120 °C) δ = 7.87 (d, J = 8.2 Hz, 1H; H-10), 7.55 (s, 1H; H-5), 7.40 (t, J = 7.9 Hz, 1H; H-9), 7.28 (s, 1H; H-23), 7.24 (t, J = 7.6 Hz, 1H; H-20), 7.15-7.10 (m, 2H; H-19 and H-21), 7.08 (t, J = 7.7, 1.4 Hz, 1H; H-8), 6.60 (s, 1H; H-12), 5.04 (d, J = 2.0 Hz, 1H; H-14), 4.64 (d, J = 15.5 Hz, 1H; H-3a), 4.59 (d, J = 15.5 Hz, 1H; H-3b), 4.58 (d, J = 2.0 Hz, 1H; H-15), 4.45-4.30 (m, 4H; H-27 and H-28), 3.97 (s, 2H; H-2), 3.49 (s, 3H; H-17), 2.72-2.54 (m, 4H; H-29 and H-24), 1.93-1.74 (m, 2H; H-26), 1.52-1.37 (m, 1H; H-25a), 1.39-1.26 (m, 1H; H-25b); **^{13}C NMR** (125 MHz, DMSO-d_6, 120 °C) δ = 169.2 (C-6), 167.7 (C-1), 163.5 (C-16), 142.4 (C-22), 142.2 (C-4), 136.6 (C-7 or C-11), 135.8 × 2 (C-18 and either C-7 or C-11), 128.7 (C-9), 128.3 (C-20), 128.0 (C-21), 126.8 (C-23), 122.3 × 2 (C-5 and C-19), 121.2 (C-8), 119.0 (C-10), 113.5 (C-12), 90.2 (C-15), 61.8 (C-14), 56.5 (C-17), 56.2 (C-28), 49.1 (C-2), 48.4 (C-27), 42.1 (C-3), 33.5 (C-24), 29.6 (t, J = 29.6 Hz; C-29), 28.3 (C-26), 27.1 (C-25); **HRMS** (ESI+) m/z = 936.2050 $[M+H]^+$ found, $C_{36}H_{31}F_{17}N_5O_5^+$ required 936.2048.

3,3,4,4,5,5,6,6,7,7,8,8,9,9,10,10,10-Heptadecafluorodecyl 2-((22*R,23*R**)-23-methoxy-24,4-dioxo-71*H*-5-aza-7(5,1)-triazola-2(2,1)-azetidina-1,3(1,3)-dibenzenacycloundecaphane-5-yl)acetate (31b)**
and **3,3,4,4,5,5,6,6,7,7,8,8,9,9,10,10,10-heptadecafluorodecyl *N*-(3-((2*R**,3*R**)-2-(3-(3,4-dihydro-2*H*-pyrrol-5-yl)phenyl)-3-methoxy-4-oxoazetidin-1-yl)benzoyl)-*N*-(prop-2-yn-1-yl)glycinate (31c)**

18a (56 mg, 0.060 mmol, 1.0 eq) was reacted with catalyst (0.8 eq) according to GSP-7 for 4 days. After preparative HPLC (60-100% B), **31b** (3.7 mg, 0.040 mmol, 7%) was obtained as a yellow solid. **31c** (5.1 mg, 5.6 µmol, 9%) was obtained as an off-white solid as a side product.

Product **31b**

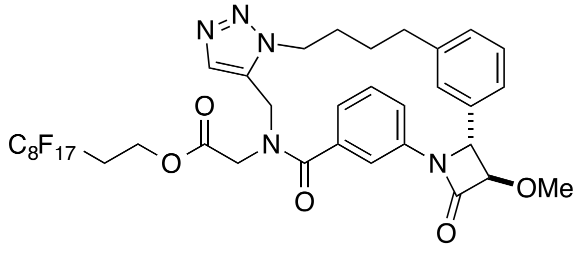

TLC R_f = 0.51 (petroleum ether/EtOAc 1:3); **IR** ν_{max} (neat)/cm^{-1} = 1753 m (C=O), 1650 w, 1453 w, 1374 m, 1197 s (C-F), 1145 s (C-F); **NMR** Appropriate NMR data could not be obtained due to the existence of rotamers at a temperature range of 25-120 °C and partial decomposition at high temperature range 90-120 °C; **HRMS** (ESI+) *m/z* = 936.2040 [M+H]$^+$ found, $C_{36}H_{31}F_{17}N_5O_5^+$ required 936.2048.

Side product **31c**

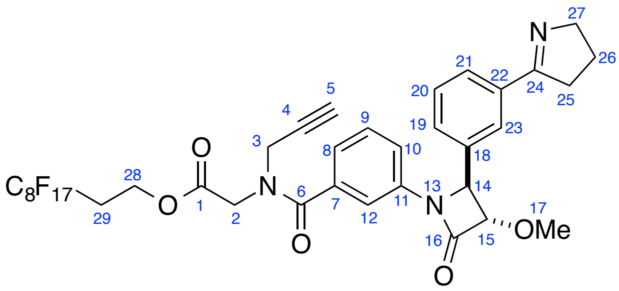

TLC R_f = 0.40 (petroleum ether/EtOAc 1:1); **HPLC** t_r = 11.92 min (50-100% B), peak area 92%; **mp** 58 °C decomposition (H_2O); **IR** ν_{max} (neat)/cm^{-1} = 1754 m (C=O), 1650 w, 1456 w, 1375 w, 1198 s (C-F), 1146 s (C-F); **^{1}H NMR** (500 MHz, DMSO-d_6, 120 °C) δ = 7.39-7.34 (m, 2H; H-12 and H-9), 7.32 (t, J = 7.6 Hz, 1H; H-20), 7.30 (ddd, J = 8.2, 2.2, 1.2 Hz, 1H; H-19), 7.28 (t, J = 1.8 Hz, 1H; H-23), 7.25 (dt, J = 7.6, 1.5 Hz, 1H; H-10), 7.21 (dt, J = 7.6, 1.5 Hz, 1H; H-21), 7.12 (dt, J = 7.5, 1.4 Hz, 1H; H-8), 5.15 (d, J = 1.9 Hz, 1H; H-14), 4.59 (d, J = 1.9 Hz, 1H; H-15), 4.41 (t, J = 6.2 Hz, 2H; H-28), 4.16 (s, 2H; H-2), 4.13 (s, 2H; H-3), 3.52 (s, 3H; H-17), 3.02 (t, J = 2.4 Hz, 1H; H-5), 2.72 (t, J = 7.3 Hz, 2H; H-25), 2.66 (tt, J = 19.3, 6.3 Hz, 2H; H-29), 2.39 (t, J = 7.2 Hz, 2H; H-27), 1.88 (quint, J = 7.2 Hz, 2H; H-26); **HRMS** (ESI+) *m/z* = 906.1839 [M+H]$^+$ found, $C_{36}H_{29}F_{17}N_3O_5^+$ required 906.1830.

3,3,4,4,5,5,6,6,7,7,8,8,9,9,10,10,10-Heptadecafluorodecyl 2-((21*R,24*R**,*Z*)-26,4-dioxo-71*H*-23,5-diaza-7(4,1)-triazola-2(2,3)-bicyclo[2.2.2]octana-1,3(1,3)-dibenzenacycloundecaphane-5-yl)acetate (33)**

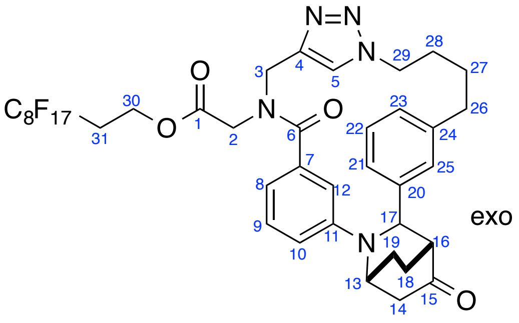

20 (17 mg, 0.018 mmol, 1.0 eq) was reacted according to GSP-6 overnight. After preparative HPLC (70-90% B, without addition of TFA), **33** (11 mg, 0.011 mmol, 65%) was obtained as a white solid.

TLC R_f = 0.22 (CH_2Cl_2/EtOAc 2:1); **HPLC** t_r = 7.20 min (70-100% B), peak area 100%; **IR** ν_{max} (neat)/cm^{-1} = 1729 m (C=O), 1646 m, 1600 w, 1458 w, 1201 s (C-F), 1145 s (C-F); **^{1}H NMR** (500 MHz, DMSO-d_6, 120 °C) δ = 7.45 (s, 1H; H-5), 7.26-7.19 (m, 3H; H-9, H-22 and either H-21 or H-23), 7.15 (s, 1H; H-25), 7.07-7.01 (m, 2H; H-10 and either H-21 or H-23), 6.63 (d, *J* = 7.4 Hz, 1H; H-8), 6.18 (s, 1H; H-12), 4.71 (d, *J* = 2.7 Hz, 1H; H-17), 4.64-4.53 (m, 3H; H-3 and H-13), 4.40-4.31 (m, 4H; H-29 and H-30), 3.86 (s, 2H; H-2), 2.68-2.53 (m, 6H; H-31, H-26, H-14a and H-16), 2.45 (dd, *J* = 18.5, 1.8 Hz, 1H; H-14b), 2.27-2.17 (m, 1H; H-19a), 2.01-1.90 (ddt, *J* = 13.1, 5.9, 2.6 Hz, 1H; H-19b), 1.80 (dtt, *J* = 14.2, 7.6, 3.8 Hz, 2H; H-28), 1.63 (dt, *J* = 9.2, 4.7 Hz, 2H; H-18), 1.47 (dtt, *J* = 13.1, 10.0, 6.6 Hz, 1H; H-27a), 1.36 (dtt, *J* = 13.5, 10.0, 6.4 Hz, 1H; H-27b); **HRMS** (ESI+) *m/z* = 960.2404 $[M+H]^+$ found, $C_{39}H_{35}F_{17}N_5O_4^+$ required 960.2412.

3,3,4,4,5,5,6,6,7,7,8,8,9,9,10,10,10-Heptadecafluorodecyl 2-((24*aS,25*S**,210*bS**,*Z*)-3-oxo-23,24,24*a*,25,26,210*b*-hexahydro-22*H*,61*H*-4-aza-2(5,8)-pyrano[3,2-*c*]quinolina-6(4,1)-triazola-1(1,3)-benzenacyclodecaphane-4-yl)acetate (25a)**

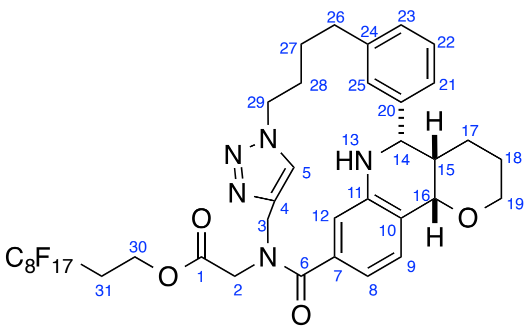

21a (20 mg, 0.021 mmol, 1.0 eq) was reacted according to GSP-6 overnight. After FCC (petroleum ether/EtOAc 1:2) and preparative HPLC (70-90% B, without addition of TFA), **25a** (11 mg, 0.012 mmol, 8%, over two steps from **3.22**) was obtained as a white solid.

TLC R_f = 0.59 (petroleum ether/EtOAc 1:3); **HPLC** t_r = 12.96 min (50-100% B, without addition of TFA), peak area 83%; **IR** ν_{max} (neat)/cm^{-1} = 1742 m (C=O), 1632 m, 1199 s (C-F), 1145 s (C-F); **^{1}H NMR** (500 MHz, DMSO-d_6, 120 °C) δ = 7.78 (s, 1H; H-5), 7.36 (s, 1H; H-25), 7.26 (d, J = 7.7 Hz, 1H; H-9), 7.24 (t, J = 7.5 Hz, 1H; H-22), 7.13 (d, J = 7.6 Hz, 1H; H-21), 7.08 (d, J = 7.5 Hz, 1H; H-23), 6.65 (dd, J = 7.7, 1.6 Hz, 1H; H-8), 6.61 (d, J = 1.5 Hz, 1H; H-12), 5.65 (s, 1H; H-13), 5.19 (d, J = 4.9 Hz, 1H; H-16), 4.78 (d, J = 4.2 Hz, 1H; H-14), 4.54-4.42 (m, 6H; H-3a, H-2a, H-29 and H-30), 4.33 (d, J = 16.7 Hz, 1H; H-3b), 4.23 (d, J = 17.0 Hz, 1H; H-2b), 3.55 (d, J = 11.2 Hz, 1H; H-19a), 3.48 (td, J = 11.2, 3.2 Hz, 1H; H-19b), 2.78-2.61 (m, 4H; H-31 and H-26, coincides with water signal), 2.31-2.22 (m, 1H; H-15), 2.10 (dq, J = 14.3, 7.0 Hz, 1H; H-28a), 1.97 (dq, J = 13.9, 6.2 Hz, 1H; H-28b), 1.74 (dt, J = 13.6, 6.8 Hz, 1H; H-27a), 1.70-1.58 (m, 1H; H-27b), 1.47-1.33 (m, 2H; H-18), 1.26-1.18 (m, 1H; H-17a), 1.15 (td, J = 13.1, 4.2 Hz, 1H; H-17b); **HRMS** (ESI+) m/z = 948.2449 $[M+H]^+$ found, $C_{38}H_{35}F_{17}N_5O_4^+$ required 948.2412.

3,3,4,4,5,5,6,6,7,7,8,8,9,9,10,10,10-Heptadecafluorodecyl 2-((2*4aR,2*5S**,2*10bR**,*Z*)-3-oxo-2*3*,2*4*,2*4a*,2*5*,2*6*,2*10b*-hexahydro-2*2H*,6*1H*-4-aza-2(5,8)-pyrano[3,2-*c*]quinolina-6(4,1)-triazola-1(1,3)-benzenacyclodecaphane-4-yl)acetate (25b)**

and **3,3,4,4,5,5,6,6,7,7,8,8,9,9,10,10,10-heptadecafluorodecyl 2-((2*4aR**,2*5R**,2*10bR**,*Z*)-3-oxo-2*3*,2*4*,2*4a*,2*5*,2*6*,2*10b*-hexahydro-2*2H*,6*1H*-4-aza-2(5,10)-pyrano[3,2-*c*]quinolina-6(4,1)-triazola-1(1,3)-benzenacyclodecaphane-4-yl)acetate (25c)**

Structures see below.

The mixture **21b** and **21c** (138 mg, 0.146 mmol in sum, 1.0 eq) was reacted according to GSP-6 overnight. After FCC (petroleum ether/EtOAc 1:1) and then lyophilisation, **25b** (22 mg, 0.023 mmol, 4%, over two steps from **2**) and **25c** (40 mg, 0.042 mmol, 7%, over two steps from **2**) were separated and both obtained as white solids.

25b

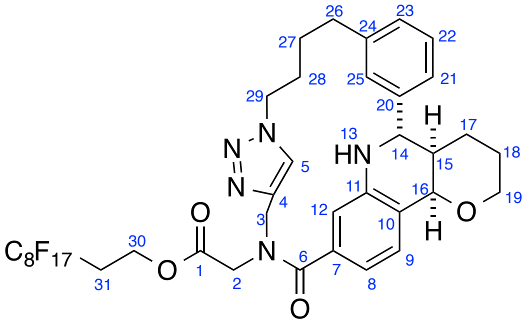

TLC R_f = 0.22 (petroleum ether/EtOAc 1:1); **HPLC** t_r = 12.78 min (50-100% B, without addition of TFA), peak area 90%; $[\alpha]_D^{25.4}$ = -2 (c = 0.090 in MeOH); **mp** 88-92 °C (H_2O); **IR** ν_{max} (neat)/cm^{-1} = 1753 w (C=O), 1642 w, 1460 w, 1203 s (C-F), 1148 s (C-F); **^{1}H NMR** (500 MHz, DMSO-d_6, 120 °C) δ = 7.62 (s, 1H; H-5), 7.23 (t, J = 7.4 Hz, 1H; H-22), 7.20 (s, 1H; H-25), 7.17 (d, J = 7.8 Hz, 1H; H-21), 7.16 (d, J = 8.0 Hz, 1H; H-9), 7.07 (d, J = 7.4 Hz, 1H; H-23), 6.56 (dd, J = 7.8, 1.5 Hz, 1H; H-8), 6.55 (s, 1H; H-12), 5.84 (s, 1H; H-13), 4.64 (d, J = 16.6 Hz, 1H; H-3a), 4.54 (d, J = 17.0 Hz, 1H; H-2a), 4.50 (d, J = 8.3 Hz, 1H; H-14), 4.45 (t, J = 6.2 Hz, 2H; H-30), 4.40 (dt, J = 13.8, 6.8 Hz, 1H; H-29a), 4.35 (d, J = 4.0 Hz, 1H; H-16), 4.34 (d, J = 16.2 Hz, 1H; H-3b), 4.29 (dt, J = 13.8, 6.8 Hz, 1H; H-29b), 4.14 (d, J = 17.0 Hz, 1H; H-2b), 3.83 (dt, J = 9.3, 4.3 Hz, 1H; H-19a), 3.65 (ddd, J = 11.4, 8.6, 3.1 Hz, 1H; H-19b), 2.68 (tt, J = 18.8, 6.1 Hz, 2H; H-31), 2.65-2.54 (m, 2H; H-26), 2.11 (dq, J = 8.8, 4.7 Hz, 1H; H-15), 1.93 (ddd, J = 15.7, 14.0, 7.0 Hz, 1H; H-28a), 1.89-1.80 (m, 2H; H-28b and H-18a), 1.76 (dq, J = 13.7, 4.5 Hz, 1H; H-17a), 1.67-1.60 (m, 1H; H-27a), 1.60-1.47 (m, 2H; H-17b and H-18b), 1.44 (td, J = 13.6, 6.9 Hz, 1H; H-27b); **^{13}C NMR** (125 MHz, DMSO-d_6, 120 °C) δ = 171.3

(C-6), 168.0 (C-1), 143.9 (C-11), 143.4 (C-20), 142.5 (C-4), 141.0 (C-24), 135.7 (C-7), 128.6 (C-9), 127.3 (C-22), 127.0 (C-23), 125.4 (C-25), 124.7 (C-21), 121.3 (C-5), 119.3 (C-10), 111.4 (C-8), 111.0 (C-12), 71.6 (C-16), 64.9 (C-19), 55.9 (C-30), 54.1 (C-14), 48.7 (C-29), 47.2 (C-2), 44.7 (C-3), 36.4 (C-15), 33.1 (C-26), 29.7 (t, J = 22.0 Hz; H-31), 27.5 (C-28), 25.9 (C-27), 23.4 (C-17), 22.0 (C-18); **HRMS** (ESI+) m/z = 948.2417 $[M+H]^+$ found, $C_{38}H_{35}F_{17}N_5O_4^+$ required 948.2412.

25c

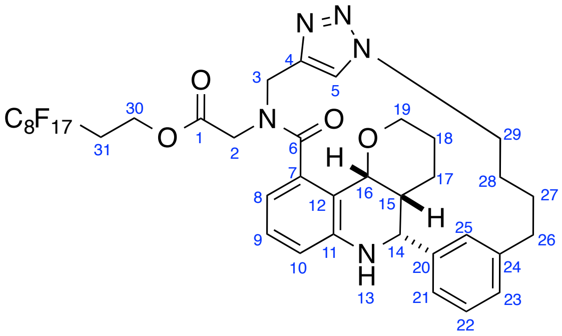

TLC R_f = 0.49 (petroleum ether/EtOAc 1:1); **HPLC** t_r = 13.27 min (50-100% B, without addition of TFA), peak area 90%; $[\alpha]_D^{25.4}$ = +2 (c = 0.029 in MeOH); **mp** 84-90 °C (H_2O); **IR** ν_{max} (neat)/cm^{-1} = 1754 m (C=O), 1637 m, 1593 m, 1479 w, 1204 s (C-F), 1151 s (C-F); **^{1}H NMR** (500 MHz, DMSO-d_6, 120 °C) δ = 7.79 (s, 1H; H-5), 7.30 (t, J = 1.7 Hz, 1H; H-25), 7.13 (t, J = 7.5 Hz, 1H; H-22), 7.07 (t, J = 7.5 Hz, 1H; H-9), 7.05 (dd, J = 6.8, 1.7 Hz, 1H; H-21), 7.00 (d, J = 7.5 Hz, 1H; H-23), 6.66 (dd, J = 8.2, 1.1 Hz, 1H; H-10), 6.54 (dd, J = 7.3, 1.2 Hz, 1H; H-8), 6.32 (s, 1H; H-13), 5.13 (d, J = 16.5 Hz, 1H; H-3a), 4.89 (d, J = 3.8 Hz, 1H; H-16), 4.59 (d, J = 17.1 Hz, 1H; H-2a), 4.46 (t, J = 6.2 Hz, 2H; H-30), 4.41 (m, 3H; H-3b, H-29a and H-14), 4.24 (ddd, J = 13.8, 7.3, 4.1 Hz, 1H; H-29b), 3.97 (d, J = 17.0 Hz, 1H; H-2b), 3.44 (dd, J = 11.3, 5.1 Hz, 1H; H-19a), 3.24 (ddd, J = 12.7, 11.3, 2.7 Hz, 1H; H-19b), 2.69 (tt, J = 19.0, 6.5 Hz, coincides with water signal; H-31), 2.45-2.40 (m, 1H; H-26a), 2.40-2.34 (m, 1H; H-26b), 2.34-2.26 (m, 1H; H-15), 1.94-1.73 (m, 3H; H-17 and H-28a), 1.72-1.60 (m, 1H; H-28b), 1.47-1.34 (m, 2H; H-27), 0.86-0.75 (m, 1H; H-18a), 0.37-0.18 (m, 1H; H-18b); **HRMS** (ESI+) m/z = 948.2436 $[M+H]^+$ found, $C_{38}H_{35}F_{17}N_5O_4^+$ required 948.2412.

3,3,4,4,5,5,6,6,7,7,8,8,9,9,10,10,10-Heptadecafluorodecyl 2-((24*aS,25*R**,210*bS**,*Z*)-3-oxo-23,24,24*a*,25,26,210*b*-hexahydro-22*H*,61*H*-4-aza-2(5,10)-pyrano[3,2-*c*]quinolina-6(4,1)-triazola-1(1,3)-benzenacyclodecaphane-4-yl)acetate (25d)**

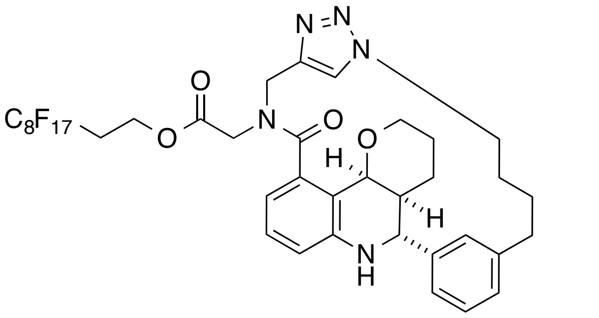

21d (16 mg, 0.017 mmol, 1.0 eq) was reacted according to GSP-6 overnight. After purification by FCC (petroleum ether/EtOAc 1:3) and preparative HPLC (70-90% B, without addition of TFA), **25d** (10 mg, 0.011 mmol, 8%, over two steps from **2**) was obtained as a white solid.

TLC R_f = 0.39 (petroleum ether/EtOAc 1:3); **HPLC** t_r = 11.93 min (50-100% B, without addition of TFA), peak area 89%; **IR** ν_{max} (neat)/cm^{-1} = 1751 m (C=O), 1647 m, 1595 m, 1462 m, 1200 s (C-F), 1146 s (C-F); **NMR** Appropriate NMR data could not be obtained due to the existence of rotamers at a temperature range of 25-120 °C and partial decomposition at high temperature range 90-120 °C; **HRMS** (ESI+) *m/z* = 948.2432 $[M+H]^+$ found, $C_{38}H_{35}F_{17}N_5O_4^+$ required 948.2412.

3,3,4,4,5,5,6,6,7,7,8,8,9,9,10,10,10-Heptadecafluorodecyl (*Z*)-2-(23-(3-hydroxypropyl)-3-oxo-61*H*-4-aza-2(2,7)-quinolina-6(4,1)-triazola-1(1,3)-benzenacyclodecaphane-4-yl)acetate (25e)

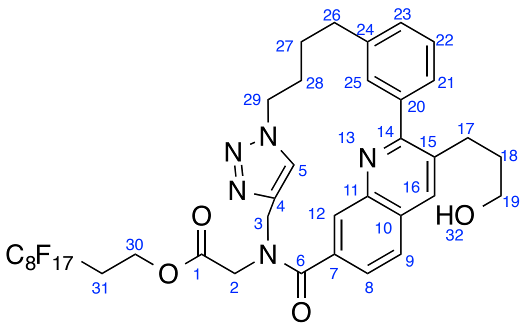

25e was partially decomposed during high temperature NMR. The mixture was re-separated by preparative HPLC (70-90% B, without addition of TFA). The new peak **25e** was obtained as a white solid.

TLC R_f = 0.63 (EtOAc); **HPLC** t_r = 9.63 min (50-100% B), peak area 100%; **^{1}H NMR** (500 MHz, DMSO-d_6, 120 °C) δ = 8.28 (s, 1H; H-16), 8.03 (d, J = 8.4 Hz, 1H; H-9), 7.98 (s, 1H; H-5), 7.83 (s, 1H; H-12), 7.68 (s, 1H; H-25), 7.67 (dd, J = 8.4, 1.7 Hz, 1H; H-8), 7.60 (d, J = 7.6 Hz, 1H; H-21), 7.38 (t, J = 7.6 Hz, 1H; H-22), 7.30 (d, J = 7.7 Hz, 1H; H-23), 4.59 (s, 2H; H-2 or H-3), 4.55 (s, 2H; H-2 or H-3), 4.49-4.44 (m, 4H; H-30 and H-29), 3.50 (t, J = 6.4 Hz, 2H; H-19), 3.13 (t, J = 7.6 Hz, 2H; H-17), 2.85 (s; H-26, coincides with water signal), 2.68 (tt, J = 18.9, 6.1 Hz; H-31, coincides with solvent signal), 2.13 (quint, J = 8.4 Hz, 2H; H-28), 1.92-1.82 (m, 2H; H-18), 1.87-1.77 (m, 2H; H-27); **HRMS** (ESI+) m/z = 946.2268 $[M+H]^+$ found, $C_{38}H_{33}F_{17}N_5O_4^+$ required 946.2256.

3,3,4,4,5,5,6,6,7,7,8,8,9,9,10,10,10-Heptadecafluorodecyl (*Z*)-2-(23-(3-hydroxypropyl)-3-oxo-61*H*-4-aza-2(2,5)-quinolina-6(4,1)-triazola-1(1,3)-benzenacyclodecaphane-4-yl)acetate (25f)

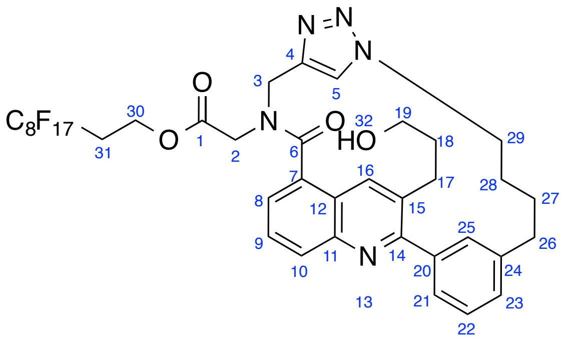

25f was partially decomposed during high temperature NMR. The mixture was separated by preparative HPLC (70-90% B, without addition of TFA). The new peak **25f** were obtained as a white solid.

TLC R_f = 0.39 (EtOAc); **HPLC** t_r = 7.96 min (50-100% B), peak area 100%; **^{1}H NMR** (500 MHz, DMSO-d_6, 120 °C) δ = 8.03 (s, 1H; H-16), 8.01 (dd, J = 8.5, 0.9 Hz, 1H; H-10), 7.75 (dd, J = 8.4, 7.0 Hz, 1H; H-9), 7.65 (d, J = 7.5 Hz, 1H; H-21), 7.53 (dd, J = 7.0, 1.0 Hz, 1H; H-8), 7.46 (t, J = 7.6 Hz, 1H; H-22), 7.23 (d, J = 7.6 Hz, 1H; H-23), 6.80 (s, 1H; H-5), 6.47 (s, 1H; H-25), 4.74 (d, J = 17.1 Hz, 1H; H-2a), 4.52 (t, J = 6.2 Hz, 2H; H-30), 4.47 (d, J = 17.0 Hz, 1H; H-2b), 4.30-4.14 (m, 3H; H-29 and H-3a), 3.84 (d, J = 17.0 Hz, 1H; H-3b), 3.26 (t, J = 6.4 Hz, 2H; H-19), 2.86-2.68 (H-26 and H-31, coincides with water signal), 2.51-2.40 (m, 2H; H-17, coincides with solvent signal), 1.80 (quint, J = 7.0 Hz, 2H; H-27), 1.73 (quint, J = 6.7 Hz, 2H; H-28), 1.55 (quint, J = 6.8 Hz, 2H; H-18); **HRMS** (ESI+) m/z = 946.2210 $[M+H]^+$ found, $C_{38}H_{33}F_{17}N_5O_4^+$ required 946.2256.

3,3,4,4,5,5,6,6,7,7,8,8,9,9,10,10,10-heptadecafluorodecyl (Z)-2-(2-cyano-5-oxo-81H-3,6-diaza-8(4,1)-triazola-1,4(1,3)-dibenzenacyclododecaphane-6-yl)acetate (34)

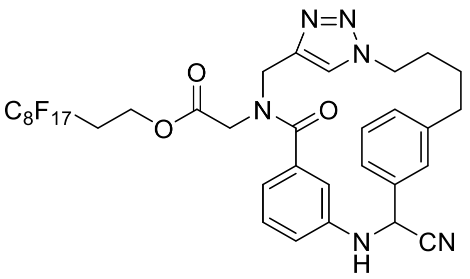

22 (0.014 g, not pure, max 0.016 mmol) was reacted according to GSP-6. After preparative HPLC (70-100% B, without addition of TFA), **34** was obtained (10 mg, 0.011 mmol, 71% over two steps from **2**) as a yellow wax.

TLC R_f = 0.31 (CH_2Cl_2/MeOH 40:1); **HPLC** t_r = 10.06 min (70-100% B), peak area 96%; **IR** ν_{max} (neat)/cm^{-1} = 3308 w (N-H), 1751 m (C=O), 1637 m (C=O), 1605 m (C=C), 1588 m (C=C), 1200 s (C-F), 1146 s (C-F); **NMR** Appropriate NMR data could not be obtained for this compound due to the existence of rotamers and isomers at a rt and partial decomposition at high temperatures; **HRMS** (ESI+) *m/z* = 891.1958 $[M+H]^+$ found, $C_{34}H_{28}F_{17}N_6O_3^+$ required 891.1951.

A mixture of **3,3,4,4,5,5,6,6,7,7,8,8,9,9,10,10,10-heptadecafluorodecyl 2-(52,2,14-trioxo-3,15-diaza-1(1,3)-benzena-5(1,3)-cyclopentanacyclopentadecaphan-51(55)-en-3-yl)acetate (23a)**

and **3,3,4,4,5,5,6,6,7,7,8,8,9,9,10,10,10-heptadecafluorodecyl (*Z*)-2-(5^5,2,14-trioxo-3,15-diaza-1(1,3)-benzena-5(1,3)-cyclopentanacyclopentadecaphan-5^1-en-3-yl)acetate (23b)**

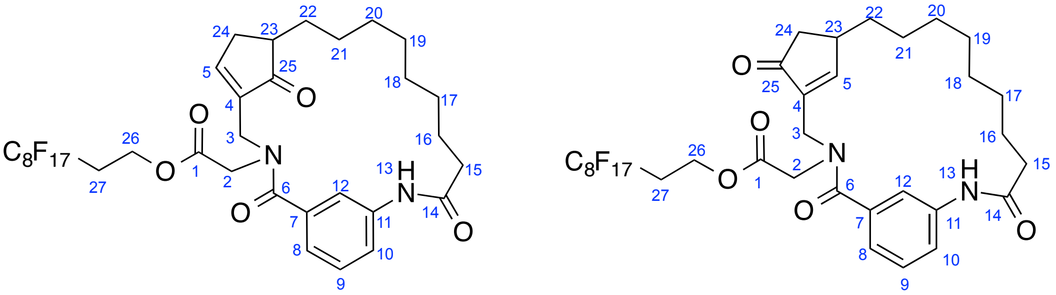

23a and **23b**

4 (42 mg, 0.050 mmol, 1.0 eq) and $Co_2(CO)_8$ (21 mg, 0.060 mmol, 1.2 eq) were dissolved in CH_2Cl_2 (10 mM) at rt. TLC indicated complete consumption of starting material in 3 h. *N*-Methylmorpholine

N-oxide (58 mg, 0.5 mmol, 10 eq) was added and the mixture stirred at rt for 3 h, then the solvent was removed. After F-SPE, **23a** and **23b** (28 mg, yield 55% together was calculated according to HPLC peak area) was obtained as a mixture as an off-white solid.

(**23a**) **HPLC** t_r= 11.42 min (60-100% B), peak area 51%; (**23b**) **HPLC** t_r= 11.42 min (60-100% B), peak area 34%.
A small portion of the mixture from a different batch was separated by preparative HPLC in order to obtain NMR spectra.

23a

TLC R_f = 0.30 (petroleum ether/EtOAc 1:1); **IR** ν_{max} (neat)/cm^{-1} = 2932 w, 1745 w (C=O), 1688 m, 1630 m, 1541 w, 1419 w, 1199 s (C-F), 1148 s (C-F); **^{1}H NMR** (500 MHz, DMSO-d_6, 27 °C) major rotamer signals only, δ = 9.95 (s, 1H; H-13), 7.74 (d, J = 7.8 Hz, 1H; H-10), 7.64 (t, J = 2.3 Hz, 1H; H-5), 7.49 (t, J = 1.9 Hz, 1H; H-12), 7.35 (t, J = 7.9 Hz, 1H; H-9), 7.00 (dt, J = 7.7, 1.4 Hz, 1H; H-8), 4.41 (t, J = 5.9 Hz, 2H; H-26), 4.33 (d, J = 17.1 Hz, 1H; H-2a), 4.05 (d, J = 17.1 Hz, 1H; H-2b), 3.98-3.84 (m, 2H; H-3), 2.88-2.60 (m, 3H; H-27 and 24a), 2.45-2.34 (m, 2H; H-23 and H-24b), 2.33-2.24 (m, 2H; H-15), 1.72-1.59 (m, 1H; H-16a), 1.59-1.44 (m, 2H; H-16b and H-22a), 1.38-1.12 (m, 10H; H-22b, H-17, H-18, H-19, H-20 and H-21a), 1.09-0.99 (m, 1H; H-21b); **^{13}C NMR** (125 MHz, DMSO-d_6, 27 °C) major rotamer signals only, δ = 209.6 (C-25), 171.5 (C-14), 171.3 (C-6), 168.7 (C-1), 158.7 (C-5), 140.4 (C-4), 139.0 (C-11), 135.9 (C-7), 129.2 (C-9), 121.0 (C-8), 119.8 (C-10), 115.4 (C-12), 56.6 (C-26), 48.1 (C-2), 46.7 (C-3), 44.9 (C-23), 35.4 (C-15), 32.7 (C-24), 29.5 (t, J = 20.2 Hz; C-27), 29.3 (C-22), 27.0 (C-18, C-19 or C-20), 26.8 (C-17, C-18, C-19 or C-20), 26.6 (C-17, C-18, C-19 or C-20), 25.8 (C-17, C-18, C-19 or C-20), 23.9 (C-16), 23.3 (C-21); **HRMS** (ESI+) m/z = 873.2155 $[M+H]^+$ found, $C_{34}H_{34}F_{17}N_2O_5^+$ required 873.2191.

23b

TLC R_f = 0.35 (petroleum ether/EtOAc 1:1); **IR** ν_{max} (neat)/cm^{-1} = 2927 w, 1753 w (C=O), 1684 w, 1625 w, 1549 w, 1420 w, 1199 s (C-F), 1145 s (C-F); **^{1}H NMR** (500 MHz, DMSO-d_6, 27 °C) major rotamer signals only, δ = 10.01 (s, 1H; H-13), 7.73 (t, J = 1.9 Hz, 1H; H-12), 7.61-7.43 (m, 2H; H-5 and H-10), 7.37 (t, J = 7.9 Hz, 1H; H-9), 7.05 (d, J = 7.5 Hz, 1H; H-8), 4.44-4.37 (m, 2H; H-26), 4.31 (d, J = 17.2 Hz, 1H; H-2a), 4.07 (d, J = 17.4 Hz, 1H; H-2b), 4.04 (d, J = 17.0 Hz, 1H; H-3a), 3.96 (d, J = 17.0 Hz, 1H; H-3b), 2.81 (apps, 1H; H-23), 2.76-2.60 (m, 2H; H-27), 2.46 (H-24a, coincides with solvent signal), 2.40-2.24 (m, 2H; H-15), 2.02-1.91 (m, 1H; H-24b), 1.73-1.52 (m, 2H; H-16), 1.48-1.18 (m, 12H; H-22, H-21, H-20, H-19, H-18 and H-17); **^{13}C NMR** (125 MHz, DMSO-d_6, 27 °C) major rotamer signals only, δ = 207.5 (C-25), 171.6 (C-14), 170.9 (C-6), 168.7 (C-1), 164.2 (C-5), 140.2 (C-4), 139.1 (C-11), 135.9 (C-7), 129.1 (C-9), 121.1 (C-8), 120.3 (C-10), 117.0 (C-12), 56.7 (C-26),

48.1 (C-2), 45.1 (C-3), 41.2 (C-24), 38.3 (C-23), 35.5 (C-15), 33.5 (C-22), 29.5 (t, *J* = 21.0 Hz; C-27), 27.1 (C-21, C-20, C-19, C-18 or C-17), 26.8 (C-21, C-20, C-19, C-18 or C-17), 26.7 (C-21, C-20, C-19, C-18 or C-17), 26.2 (C-21, C-20, C-19, C-18 or C-17), 25.1 (C-21, C-20, C-19 or C-18 or C-17), 23.8 (C-16); **HRMS** (ESI+) *m/z* = 873.2222 $[M+H]^+$ found, $C_{34}H_{34}F_{17}N_2O_5^+$ required 873.2191.

3,3,4,4,5,5,6,6,7,7,8,8,9,9,10,10,10-Heptadecafluorodecyl *N*-allyl-*N*-(3-(undec-10-enamido)benzoyl)glycinate (23c)

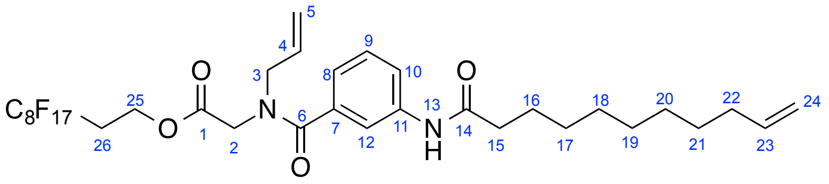

HPLC t_r = 12.27 min (70-100% B), peak area 100%; **IR** ν_{max} (neat)/cm^{-1} = 2926 w, 1743 w (C=O), 1614 w, 1199 s (C-F), 1147 s (C-F); **^{1}H NMR** (500 MHz, DMSO-d_6, 90 °C) δ = 9.68 (s, 1H; H-13), 7.71 (s, 1H; H-12), 7.58 (d, *J* = 8.3 Hz, 1H; H-10), 7.33 (t, *J* = 7.9 Hz, 1H; H-9), 7.02 (d, *J* = 7.5 Hz, 1H; H-8), 5.88-5.75 (m, 2H; H-4 and H-23), 5.27-5.14 (m, 2H; H-5), 4.99 (d, *J* = 17.1 Hz, 1H; H-24_{trans}), 4.93 (d, *J* = 10.2 Hz, 1H; H-24_{cis}), 4.42 (t, *J* = 6.1 Hz, 2H; H-25), 4.10 (s, 2H; H-2), 3.97 (s, 2H; H-3), 2.66 (tt, *J* = 19.5, 6.4 Hz, 2H; H-26), 2.30 (t, *J* = 7.4 Hz, 2H; H-15), 2.02 (q, *J* = 7.0 Hz, 2H; H-22), 1.61 (q, *J* = 7.4 Hz, 2H; H-16), 1.46-1.23 (m, 10H; H-21, H-17, H-18, H-19 and H-20); **^{13}C NMR** (125 MHz, DMSO-d_6, 27 °C) major rotamer signals only, δ = 171.5 (C-14), 170.9 (C-6), 168.6 (C-1), 139.4 (C-11), 138.8 (C-23), 135.7 (C-7), 133.3 (C-4), 128.8 (C-9), 120.6 (C-8), 120.2 (C-10), 117.8 (C-5), 117.1 (C-12), 114.6 (C-24), 56.6 (C-25), 52.4 (C-3), 46.6 (C-2), 36.4 (C-15), 33.2 (C-22), 29.5 (C26), 28.7 (two carbons from C-21, C-17, C-18, C-19 and C-20), 28.6 (C-21, C-17, C-18, C-19 or C-20), 28.5 (C-21, C-17, C-18, C-19 or C-20), 28.2 (C-21, C-17, C-18, C-19 or C-20), 25.0 (C-16); **HRMS** (ESI+) *m/z* = 847.2391 $[M+H]^+$ found, $C_{33}H_{35}F_{17}N_2O_4^+$ required 847.2398.

3,3,4,4,5,5,6,6,7,7,8,8,9,9,10,10,10-Heptadecafluorodecyl (3-(undec-10-enamido)benzoyl)glycinate (**23d**)

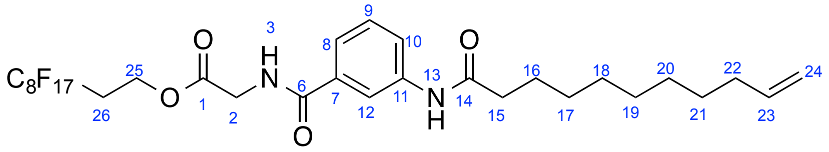

HPLC t_r = 10.42 min (70-100% B), peak area 89%; **IR** ν_{max} (neat)/cm^{-1} = 3317 brw, 2924 w, 1751 w (C=O), 1667 m, 1642 m, 1549 m, 1196 s (C-F), 1144 s (C-F); **^{1}H NMR** (500 MHz, DMSO-d_6, 90 °C) δ = 10.01 (s, 1H; H-13), 8.88 (t, J = 5.8 Hz, 1H; H-3), 8.07 (t, J = 2.0 Hz, 1H; H-12), 7.76 (dd, J = 8.1, 1.2 Hz, 1H; H-10), 7.49 (dt, J = 7.8, 1.3 Hz, 1H; H-8), 7.38 (t, J = 7.9 Hz, 1H; H-9), 5.78 (ddt, J = 17.0, 10.2, 6.7 Hz, 1H; H-23), 4.98 (dq, J = 17.2, 1.7 Hz, 1H; H-24$_{trans}$), 4.92 (ddt, J = 10.2, 2.3, 1.2 Hz, 1H; H-24$_{cis}$), 4.39 (t, J = 5.9 Hz, 2H; H-26), 4.00 (d, J = 5.8 Hz, 2H; H-2), 2.67 (tt, J = 19.3, 6.1 Hz, 2H; H-26), 2.30 (t, J = 7.4 Hz, 2H; H-15), 2.06-1.90 (m, 2H; H-22), 1.63-1.51 (m, 2H; H-16), 1.40-1.16 (m, 10H; H-21, H-17, H-18, H-19 and H-20); **^{13}C NMR** (125 MHz, DMSO-d_6, 27 °C) δ = 171.4 (C-14), 169.7 (C-1), 166.7 (C-6), 139.5 (C-11), 138.8 (C-23), 134.3 (C-7), 128.6 (C-9), 121.9 (C-10), 121.4 (C-8), 118.4 (C-12), 114.6 (C-24), 56.5 (C-25), 41.3 (C-2), 36.4 (C-15), 33.2 (C-22), 29.5 (t, J = 20.48 Hz; C-26), 28.8 (two carbons from C-21, C-17, C-18, C-19 and C-20), 28.6 (C-21, C-17, C-18, C-19 or C-20), 28.5 (C-21, C-17, C-18, C-19 or C-20), 28.2 (C-21, C-17, C-18, C-19 or C-20), 25.1 (C-16); **HRMS** (ESI+) m/z = 807.2094 $[M+H]^+$ found, $C_{30}H_{32}F_{17}N_2O_4^+$ required 807.2085.

3,3,4,4,5,5,6,6,7,7,8,8,9,9,10,10,10-Heptadecafluorodecyl (*E*)-2-(13-methylene-3,8,16-trioxo-2,7,9,15-tetraaza-1,6(1,3)-dibenzenacyclohexadecaphan-11-en-15-yl)acetate (35)

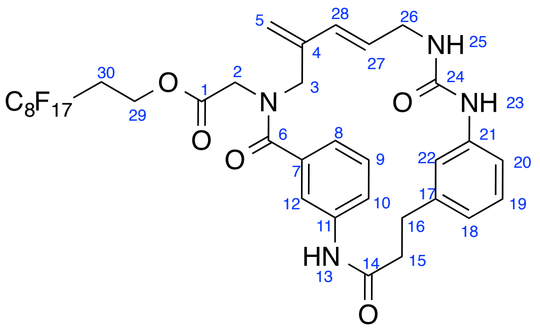

12 (19 mg, 0.021 mmol, 1.00 eq) was dissolved in CH_2Cl_2 (1 mM). Ethylene gas was bubbled through the solution for 20 min. Grubbs' 2nd generation catalyst (3.6 mg, 4.2 µmol, 0.2 eq) was added and the reaction heated to reflux under an ethylene atmosphere. After 5 h TLC indicated complete consumption of starting material, the atmosphere was changed to argon and continued refluxing overnight. The solvent was removed and re-dissolved in CH_2Cl_2 (1 mL). $Pb(OAc)_2$ (2.3 mg, 6.3 µmol, 1.5 eq to 1 eq Grubbs' 2nd generation catalyst) was added and the mixture stirred at rt for 4 h. The mixture was filtered through a pad of silica gel (EtOAc). The filtrate was concentrated under reduced pressure. After preparative HPLC (60-100% B), **35** (8.3mg, 9.1 µmol, 44%) was obtained as a white solid.

TLC R_f = 0.40 (petroleum ether/EtOAc 2:1); **HPLC** t_r = 6.27 min (70-100% B), peak area 100%; **mp** 114 °C decomposition (H_2O); **IR** ν_{max} (neat)/cm^{-1} = 2923 w, 1749 w (C=O), 1650 m (C=O), 1594 m, 1556 m, 1446 w, 1199 s (C-F), 1145 s (C-F); **^{1}H NMR** (500 MHz, DMSO-d_6, 120 °C) δ = 9.39 (brs, 1H; H-13), 7.96 (brs, 1H; H-23), 7.67 (s, 1H; H-22), 7.56 (brs, 1H; H-12), 7.50 (br, 1H; H-10), 7.27 (t, J = 7.8 Hz, 1H; H-9), 7.09 (t, J = 7.7 Hz, 1H; H-19), 7.00 (d, J = 7.6 Hz, 1H; H-8), 6.82-6.77 (m, 2H; H-20 and H-18), 6.10 (d, J = 16.1 Hz, 1H; H-28), 6.04 (brs, 1H; H-25), 5.76 (brs, 1H; H-27), 5.24 (s, 1H; H-5a), 5.10 (s, 1H; H-5b), 4.42 (t, J = 6.1 Hz, 2H; H-29), 4.22 (s, 2H; H-3), 4.05 (s, 2H; H-2), 3.69 (appbrs, 2H; H-26), 2.97 (H-16, coincides with water signal), 2.76-2.57 (m, 4H; H-15 and H-30); **^{13}C NMR** (125 MHz, DMSO-d_6, 120 °C) due to interconversion, C-2, C-3, C-5, C-10, C-12 and C-27 are missing, δ = 170.2 (C-6), 169.6 (C-14), 167.8 (C-1), 154.8 (C-24), 140.7 (C-17), 139.9 (C-21), 139.5 (C-4), 138.4 (C-11), 135.3 (C-7), 128.2 (C-28), 127.5 × 2 (C-9 and C-19), 121.4 (C-18), 120.8 (C-8), 118.4 (C-22), 115.9 (C-20), 56.1 (C-29), 40.9 (C-26), 36.8 (C-15), 30.0 (C-16), 29.65 (t, J = 21.6 Hz; C-30); **HRMS** (ESI+) m/z = 909.1940 $[M+H]^+$ found, $C_{35}H_{30}F_{17}N_5O_4^+$ required 909.1939. The geometry of the diene is as drawn.

3,3,4,4,5,5,6,6,7,7,8,8,9,9,10,10,10-Heptadecafluorodecyl (*E*)-2-(3,13-dioxo-2,4,12-triaza-1(1,3)-benzenacyclotridecaphan-9-en-12-yl)acetate (36a)

and ***bis*(3,3,4,4,5,5,6,6,7,7,8,8,9,9,10,10,10-heptadecafluorodecyl) 2,2'-(3,13,16,26-tetraoxo-2,4,12,15,17,25-hexaaza-1,14(1,3)-dibenzenacyclohexacosaphane-9,22-diene-12,25-diyl)diacetate (36b)**

Structures see below.

10 (120 mg, 0.149 mmol, 1.0 eq) was dissolved in CH_2Cl_2 (0.375 mM). Argon was bubbled through the solution for 20 min. Grubbs' 2nd generation catalyst (26 mg, 0.031 mmol, 0.2 eq) was added. The reaction was heated under reflux for 10 h. The solvent was removed. After FCC (petroleum ether/EtOAc 1:1, then EtOAc) and preparative HPLC (70-100% B), **3.196** (48 mg, 0.062 mmol, 42%) was obtained as a white powder. **3.197** (10 mg, 6.4 µmol, 4%) was obtained as a byproduct as a white powder.

36a

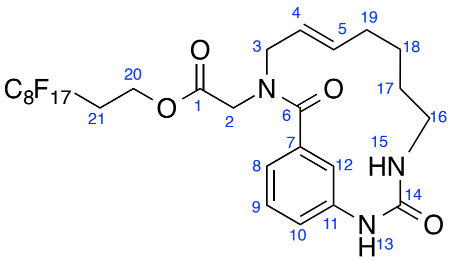

TLC R_f = 0.47 (petroleum ether/EtOAc 1:4); **HPLC** t_r = 7.92 min (70-100% B), peak area 98%; **mp** 105-109 °C (H_2O); **IR** ν_{max} (neat)/cm^{-1} = 1763 m (C=O), 1660 m (C=O), 1632 m (C=O), 1605 m, 1587 m, 1543 m, 1462 m, 1197 s, 1172 s, 1145 s, 1133 s; **^{1}H NMR** (500 MHz, DMSO-d_6, 27 °C) δ = 8.33 (s, 1H; H-13), 7.42 (s, 1H; H-12), 7.32 (t, J = 7.8 Hz, 1H; H-9), 7.05 (d, J = 7.2 Hz, 1H; H-10), 7.01 (dt, J = 7.7, 1.3 Hz, 1H; H-8), 6.35 (brs, 1H; H-15), 5.66 (dt, J = 14.8, 6.9 Hz, 1H; H-5), 5.48 (dt, J = 15.9, 4.1 Hz, 1H; H-4), 4.39 (t, J = 5.9 Hz, 2H; H-20), 4.08 (s; H-2, coincides with water signal), 3.84 (s, 2H; H-3), 3.05 (appbrs, 2H; H-16), 2.68 (tt, J = 20.0, 6.0 Hz, 2H; H-21), 2.00 (appbrs, 2H; H-19), 1.58-1.48 (m, 2H; H-17), 1.48-1.37 (m, 2H; H-18); **^{13}C NMR** (125 MHz, DMSO-d_6, 27 °C) δ = 171.3 (C-6), 168.7 (C-1), 155.9 (C-14), 139.5 (C-11), 135.7 (C-7), 130.9 (C-5), 129.4 (C-9), 126.1 (C-4), 122.3 (C-10), 121.2 (C-8), 117.6 (C-12), 56.5 (C-20), 51.3 (C-3), 47.7 (C-2), 39.0 (C-16, coincides with solvent signal), 30.4 (C-19), 29.49 (t, J = 20.5 Hz; C-21), 26.94 (C-17), 25.27 (C-18); **HRMS** (ESI+) m/z = 778.1573 [M+H]$^+$ found, $C_{27}H_{25}F_{17}N_3O_4^+$ required 778.1568. The geometry of the alkene is as drawn.

36b

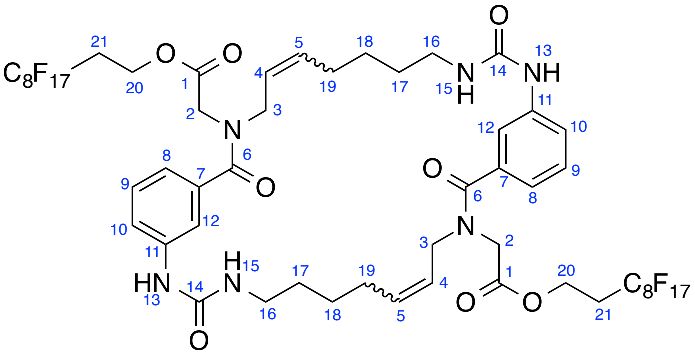

TLC R_f = 0.60 (petroleum ether/EtOAc 1:4); **HPLC** t_r = 15.97 min (70-100% B), peak area 100%; **mp** 148 °C decomposition (H_2O); **IR** ν_{max} (neat)/cm^{-1} = 1760 w (C=O), 1620 w (C=O), 1571 w, 1416 w, 1199 s, 1149 s; **^{1}H NMR** (500 MHz, DMSO-d_6, 120 °C) major rotamer signals only, δ = 8.21 (s, 2H; H-13 × 2), 7.69 (s, 2H; H-12 × 2), 7.33-7.14 (m, 4H; H-9 × 2 and H-10 × 2), 6.88 (d, J = 7.2 Hz, 2H;

H-8 × 2), 6.03-5.83 (m, 2H; H-15 × 2), 5.70-5.35 (m, 4H; H-4 × 2 and H-5 × 2), 4.42 (t, J = 6.1 Hz, 4H; H-20 × 2), 4.11 (s, 4H; H-2 × 2), 3.87 (d, J = 5.7 Hz, 4H; H-3 × 2), 3.13 (q, J = 6.2 Hz, 4H; H-16 × 2), 2.66 (tt, J = 19.0, 6.5 Hz, 4H; H-21 × 2), 2.05 (q, J = 6.7 Hz, 4H; H-19 × 2), 1.56-1.36 (m, 8H; H-17 × 2 and H-18 × 2); **^{13}C NMR** (125 MHz, DMSO-d_6, 27 °C) major rotamer signals only, δ = 171.2 (C-6 × 2), 168.8 (C-1 × 2), 155.0 (C-14 × 2), 140.3 (C-11 × 2), 135.9 (C-7 × 2), 134.1 (C-5 × 2), 128.9 (C-9 × 2), 124.5 (C-4 × 2), 119.3 (C-8 × 2), 118.4 (C-10 × 2), 115.3 (C-12 × 2), 56.6 (C-20 × 2), 51.9 (C-3 × 2), 46.5 (C-2 × 2), 38.8 (C-16 × 2, coincides with solvent signal), 31.1 (C-19 × 2), 29.5 (C-21 × 2), 29.3 (C-17 × 2), 25.4 (C-18 × 2); **HRMS** (ESI+) m/z = 1577.2880 $[M+Na]^+$ found, $C_{54}H_{48}F_{34}N_6O_8Na^+$ required 1577.2883. The geometry of the alkene is as drawn.

Fluorous-tagged macrocycle 37

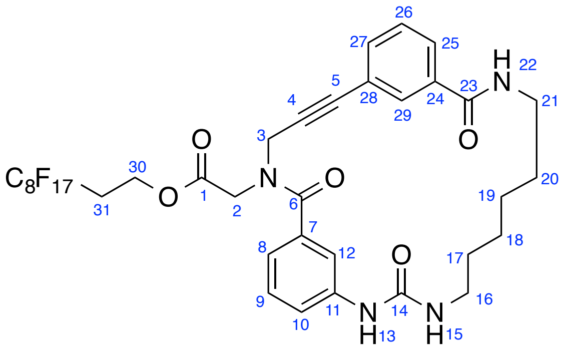

7 (80 mg, 0.076 mmol, 1.0 eq) and $Pd(PPh_3)_4$ (44 mg, 0.038 mmol, 0.5 eq) were dissolved in acetonitrile (76 mL). Diethylamine (12 μL, 0.011 mmol, 1.5 eq) was added. The mixture was stirred at 50 °C for 1 day. HPLC indicated complete turnover. Solvent was removed under reduced pressure. After F-SPE, **37** (30 mg, 0.033 mmol, 43%) was obtained as a pale yellow solid.

TLC R_f = 0.26 (petroleum ether/EtOAc 1:2); **HPLC** t_r = 14.52 min (50-100% B), peak area 98%; **mp** 117 °C decomposition (H_2O); **IR** ν_{max} (neat)/cm^{-1} = 1748 w (C=O), 1638 m (C=O), 1556 m, 1201 s (C-F), 1144 s (C-F); **^{1}H NMR** (500 MHz, DMSO-d_6, 90 °C) δ = 8.37 (s, 1H; H-13), 8.17 (s, 1H; H-22), 8.14 (s, 1H; H-12), 7.98 (s, 1H; H-29), 7.76 (d, J = 7.6 Hz, 1H; H-25), 7.55 (d, J = 7.5 Hz, 1H; H-27), 7.44 (t, J = 7.7 Hz, 1H; H-26), 7.32 (t, J = 7.8 Hz, 1H; H-9), 7.16 (d, J = 8.1 Hz, 1H; H-10), 6.96 (d, J = 7.4 Hz, 1H; H-8), 5.98 (brs, 1H; H-15), 4.44 (t, J = 6.1 Hz, 2H; H-30), 4.38 (s, 2H; H-3), 4.33 (s, 2H; H-2), 3.35 (q, J = 5.7 Hz, 2H; H-21), 3.09 (q, J = 6.6 Hz, 2H; H-16), 2.69 (tt, J = 18.9, 5.5 Hz, 2H; H-31), 1.57 (quint, J = 6.3 Hz, 2H; H-20), 1.51 (quint, J = 6.6 Hz, 2H; H-17), 1.46-1.37 (m, 4H; H-18 and H-19); **^{13}C NMR** (125 MHz, DMSO-d_6, 27 °C) δ = 170.6 (C-6), 168.4 (C-1), 166.2 (C-23), 154.9 (C-14), 140.5 (C-11), 136.0 (C-24), 134.9 (C-7), 133.9 (C-27), 130.5 (C-29), 129.2 (C-9), 128.5 (C-

26), 127.7 (C-25), 121.9 (C-28), 120.0 (C-8), 118.9 (C-10), 114.9 (C-12), 84.8 (C-4), 83.6 (C-5), 56.7 (C-30), 46.7 (C-2), 40.8 (C-3), 38.3 (C-21), 37.6 (C-16), 29.5 (t, *J* = 21.2 Hz; C-31), 28.0 (C-17), 27.7 (C-20), 24.4 (C-18 or C-19), 24.2 (C-19 or C-18); **HRMS** (ESI+) *m/z* = 923.2126 $[M+H]^+$ found, $C_{36}H_{32}F_{17}N_4O_5^+$ required 923.2096.

Fluorous-tagged macrocycle 38

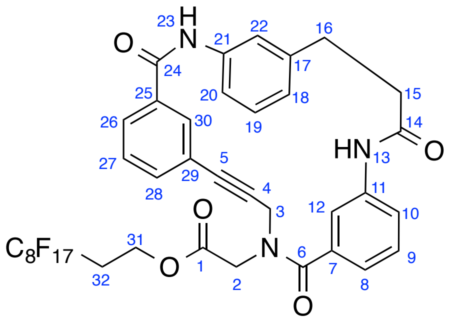

11 (53 mg, 0.050 mmol, 1.0 eq) and $Pd(PPh_3)_4$ (29 mg, 0.025 mmol, 0.5 eq) were dissolved in acetonitrile (25 mL). Diethylamine (7.8 µL, 0.075 mmol, 1.5 eq) was added. The mixture was stirred at 50 °C overnight. HPLC indicated complete turnover. Solvent was removed under reduced pressure. After F-SPE, **38** (18 mg, 0.019 mmol, 38%) was obtained as a pale yellow solid.

TLC R_f = 0.41 (petroleum ether/EtOAc 1:1); **HPLC** t_r = 12.0 min (50-100% B), peak area 88%; **mp** 218-220 °C (H_2O); **IR** ν_{max} (neat)/cm^{-1} = 3073 w, 1759 m (C=O), 1674 s (C=O), 1633 s (C=O), 1587 m, 1549 m, 1461 m, 1196 s (C-F), 1152 s (C-F); ^{1}**H NMR** (500 MHz, DMSO-d_6, 90 °C) δ = 10.12 (s, 1H; H-23), 9.77 (s, 1H; H-13), 8.01 (d, *J* = 8.2 Hz, 1H; H-20), 7.98 (t, *J* = 1.7 Hz, 1H; H-12), 7.81 (s, 1H; H-30), 7.78 (dt, *J* = 7.3, 1.8 Hz, 1H; H-26), 7.55-7.45 (m, 3H; H-28, H-27 and H-10), 7.35 (t, *J* = 7.8 Hz, 1H; H-9), 7.32 (s, 1H; H-22), 7.23 (t, *J* = 7.8 Hz, 1H; H-19), 7.08 (d, *J* = 7.5 Hz, 1H; H-8), 6.95 (d, *J* = 7.5 Hz, 1H; H-18), 4.54 (s, 2H; H-3), 4.41 (t, *J* = 6.1 Hz, 2H; H-31), 4.27 (s, 2H; H-2), 3.05-2.94 (m, 2H; H-16), 2.75-2.57 (m, 4H; H-15 and H-32); ^{13}**C NMR** (125 MHz, DMSO-d_6, 90 °C) δ = 170.2 (C-6), 170.0 (C-14), 168.0 (C-1), 164.5 (C-24), 141.9 (C-17), 139.1 (C-11), 138.8 (C-21), 135.4 (C-25), 135.1 (C-7), 132.5 (C-30), 131.4 (C-28), 128.5 (C-27), 127.9 (C-19), 127.8 (C-9), 127.4 (C-26), 123.5 (C-18), 121.4 (C-29 or C-10), 121.3 (C-29 or C-10), 120.4 (C-8), 119.6 (C-12 or C-22), 119.5 (C-12 or C-22), 116.5 (C-20), 86.3 (C-4), 82.6 (C-5), 56.4 (C-31), 48.8 (C-2), 37.8 (C-3), 36.4 (C-15), 29.6 (t, *J* = 21.3 Hz; H-32), 28.8 (C-16); **HRMS** (ESI+) *m/z* = 928.1691 $[M+H]^+$ found, $C_{38}H_{27}F_{17}N_3O_5^+$ required 928.1674.

Macrocycle 39

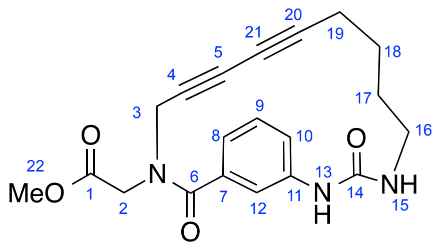

8 (40 mg, 0.050 mmol, 1.0 eq) and $Cu(OAc)_2$ (18 mg, 0.10 mmol, 2.0 eq) were dissolved in MeOH (10 mM). Pyridine (16 uL, 0.20 mmol, 4.0 eq) was added. The reaction mixture was heated at 90 °C by microwave irradiation for 4 h. The solvent was removed and the residue was filtered through a pad of silica (CH_2Cl_2/MeOH, 20:1). After preparative HPLC (10-60% B), **39** (14 mg, 0.038 mmol, 76%) was obtained as an off-white solid.

TLC R_f = 0.31 (CH_2Cl_2/MeOH 20:1); **mp** 102 °C decomposition (H_2O); **HPLC** t_r = 11.34 min (10-60% B), peak area 98%; **IR** ν_{max} (neat)/cm^{-1} = 1747 m (C=O), 1645 s, 1552 s, 1206 s, 1174 s; **^{1}H NMR** (500 MHz, DMSO-d_6, 120 °C) δ = 8.14 (s, 1H; H-13), 8.04 (d, J = 1.9 Hz, 1H; H-12), 7.29 (t, J = 7.8 Hz, 1H; H-9), 7.06 (dd, J = 8.0, 2.1 Hz, 1H; H-10), 6.89 (d, J = 7.5 Hz, 1H; H-8), 6.12 (brs, 1H; H-15), 4.22 (s, 2H; H-2), 3.99 (s, 2H; H-3), 3.72 (s, 3H; H-22), 3.11 (t, J = 5.9 Hz, 2H; H-16), 2.30 (appt, J = 5.9 Hz, 2H; H-19), 1.84-1.69 (m, 2H; H-17), 1.68-1.50 (m, 2H; H-18); **^{13}C NMR** (125 MHz, DMSO-d_6, 120 °C) δ = 172.0 (C-6), 169.2 (C-1), 155.2 (C-14), 140.0 (C-11), 135.4 (C-7), 128.8 (C-9), 119.4 (C-10), 118.5 (C-8), 117.7 (C-12), 82.1 (C-20), 73.0 (C-4), 68.9 (C-5), 65.1 (C-21), 51.9 (C-22), 47.8 (C-2), 40.8 (C-3), 39.5 (C-16, coincides with solvent signal), 27.1 (C-17), 24.5 (C-18), 18.7 (C-19); **HRMS** (ESI+) m/z = 368.1590 $[M+H]^+$ found, $C_{20}H_{22}N_3O_4^+$ required 368.1605.

In the *modify* stage

Methyl 2-((9*S*,*Z*)-9-(dimethylcarbamoyl)-4,7-dioxo-11*H*-3,6,8-triaza-1(4,1)-triazola-5(1,3)-benzenacyclotridecaphane-3-yl)acetate (40a)

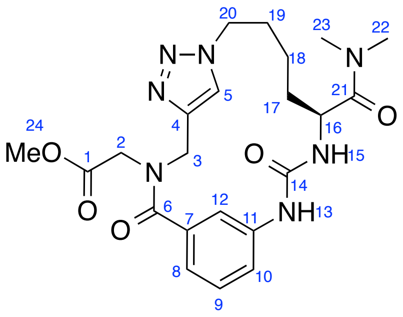

24a (28 mg, 0.031 mmol, 1.0 eq) was reacted according to GSP-8. After purification by preparative HPLC (10-60% B), **40a** (14.5 mg, 0.0308 mmol, quant.) was obtained as a white powder.

TLC R_f = 0.61 (CH_2Cl_2/MeOH 10:1); **HPLC** t_r = 7.31 min (10-60% B), peak area 99%; $[\alpha]_D^{25.3}$ = +31 (c = 0.137 in MeOH); **IR** ν_{max} (neat)/cm^{-1} = 1747 m (C=O), 1685 m, 1636 s, 1547 m, 1420 m, 1401 m, 1210 s; ^{1}**H NMR** (500 MHz, DMSO-d_6, 27 °C) δ = 8.79 (s, 1H; H-13), 8.10 (s, 1H; H-5), 7.96 (s, 1H; H-12), 7.32 (t, J = 7.8 Hz, 1H; H-9), 7.01 (d, J = 7.5 Hz, 1H; H-8), 6.82 (dd, J = 8.0, 2.3 Hz, 1H; H-10), 6.44 (d, J = 8.5 Hz, 1H; H-15), 4.70 (7, J = 9.7 Hz, 1H; H-16), 4.54-4.43 (m, 3H; H-3 and H-20a), 4.43-4.36 (m, 1H; H-20b), 4.33 (d, J = 17.1 Hz, 1H; H-2a), 4.04 (d, J = 17.1 Hz, 1H; H-2b), 3.68 (s, 3H; H-24), 3.00 (s, 3H; H-22 or H-23), 2.85 (s, 3H; H-22 or H-23), 2.45-2.28 (m, 1H; H-19a), 1.77-1.56 (m, 2H; H-19b and H-17a), 1.46-1.34 (m, 1H; H-17b), 1.31-1.22 (m, 1H; H-18a), 1.20-1.07 (m, 1H; H-18b); ^{13}**C NMR** (125 MHz, DMSO-d_6, 27 °C) δ = 171.8 (C-6), 171.5 (C-21), 169.5 (C-1), 154.4 (C-14), 143.7 (C-4), 139.5 (C-11), 135.1 (C-7), 129.4 (C-9), 122.2 (C-5), 120.1 (C-8), 119.4 (C-10), 116.1 (C-12), 51.8 (C-24), 50.1 (C-20), 47.5 (C-2), 46.9 (C-3 or C-16), 46.8 (C-3 or C-16), 36.5 (C-22 or C-23), 35.0 (C-22 or C-23), 33.7 (C-17), 28.1 (C-19), 22.3 (C-18); **HRMS** (ESI+) m/z = 472.2320 $[M+H]^+$ found, $C_{22}H_{30}N_7O_5^+$ required 472.2303.

Methyl (*S*)-2-(9-(dimethylcarbamoyl)-4,7-dioxo-11*H*-3,6,8-triaza-1(5,1)-triazola-5(1,3)-benzenacyclotridecaphane-3-yl)acetate (40b)

and **methyl 2-((14*S*)-12,15,3-trioxo-61*H*-4-aza-6(5,1)-triazola-1(1,4)-imidazolidina-2(1,3)-benzenacyclodecaphane-4-yl)acetate (40d)**

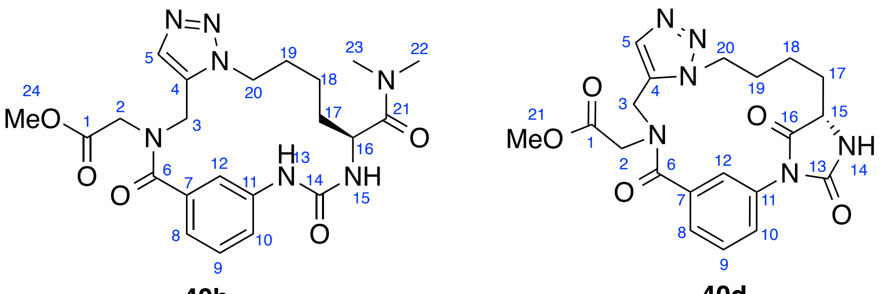

40b and **40d**

24b (25 mg, 0.028 mmol, 1.0 eq) was reacted according to GSP-8. After preparative HPLC (10-60% B), **40b** (10 mg, 0.021 mmol, 77%) was obtained as a white powder. **40d** (1.24 mg, 2.9 µmol, 11%) was obtained as a white powder as a side product of the transesterification.

40b

TLC R_f = 0.56 (CH_2Cl_2/MeOH 10:1); **HPLC** t_r = 6.14 min (10-60% B), peak area 99%; **[α]$_D^{25.3}$** = -14 (*c* = 0.148 in MeOH); **IR** ν_{max} (neat)/cm^{-1} = 1744 m (C=O), 1634 m, 1503 m, 1461 m, 1420 m, 1403 m, 1201 s; **^{1}H NMR** (500 MHz, DMSO-d_6, 120 °C) δ = 8.12 (brs, 1H; H-13), 7.66 (s, 1H; H-5), 7.46 (s, 1H; H-12), 7.40 (t, *J* = 7.8 Hz, 1H; H-9), 7.19 (d, *J* = 7.6 Hz, 1H; H-8), 7.11 (d, *J* = 7.8 Hz, 1H; H-10), 6.06 (brs, 1H; H-15), 4.85 (d, *J* = 16.6 Hz, 1H; H-3a), 4.78 (d, *J* = 16.8 Hz, 1H; H-3b), 4.68-4.57 (appbrs, 1H; H-16), 4.16 (t, *J* = 6.8 Hz, 2H; H-20), 4.11 (s, 2H; H-2), 3.69 (s, 3H; H-24), 2.94 (s, 6H; H-22 and H-23), 2.04-1.89 (m, 1H; H-19a), 1.89-1.76 (m, 1H; H-19b), 1.76-1.65 (m, 1H; H-17a), 1.52 (dq, *J* = 14.6, 7.3 Hz, 1H; H-17b), 1.37-1.22 (m, 2H; H-18); **^{13}C NMR** (125 MHz, DMSO-d_6, 120 °C) due to interconversion, C-3 is missing, δ = 171.0 (C-21), 169.9 (C-6), 168.4 (C-1), 155.2 (C-14), 139.2 (C-11), 134.4 (C-7), 132.6 (C-4 or C-5), 132.5 (C-4 or C-5), 129.0 (C-9), 123.4 (C-8), 122.1 (C-10), 119.1 (C-12), 51.0 (C-24), 49.0 (C-16), 47.8 (C-2), 47.2 (C-20), 34.0 (C-22 and C-23), 30.5 (C-17), 28.3 (C-19), 21.6 (C-18); **HRMS** (ESI+) *m/z* = 472.2314 [M+H]$^+$ found, $C_{22}H_{30}N_7O_5^+$ required 472.2303.

40d

TLC R_f = 0.10 (EtOAc); **HPLC** t_r = 6.42 min (10-60% B), peak area 96%; **[α]$_D^{25.3}$** = -72 (*c* = 0.085 in MeOH); **IR** ν_{max} (neat)/cm^{-1} = 1708 s (C=O), 1633 m, 1407 m, 1185 s; **^{1}H NMR** (500 MHz, DMSO-d_6, 90 °C) δ = 8.11 (s, 1H; H-14), 7.80 (s, 1H; H-5), 7.69 (dt, *J* = 7.9, 1.6 Hz, 1H; H-8 or H-10), 7.66 (t, *J*

= 7.7 Hz, 1H; H-9), 7.52 (dt, J = 7.2, 2.1 Hz, 1H; H-8 or H-10), 6.90 (s, 1H; H-12), 4.82 (d, J = 18.2 Hz, 1H; H-3a), 4.61 (d, J = 18.3 Hz, 1H; H-3b), 4.56 (d, J = 16.9 Hz, 1H; H-2a), 4.29 (t, J = 3.8 Hz, 1H; H-15), 4.25 (td, J = 8.7, 8.1, 3.8 Hz, 1H; H-20a), 4.13 (ddd, J = 14.3, 6.9, 3.8 Hz, 1H; H-20b), 4.01 (d, J = 16.8 Hz, 1H; H-2b), 3.71 (s, 3H; H-21), 1.93-1.81 (m, 2H; H-17), 1.71-1.63 (s, 1H; H-19a), 1.39-1.28 (m, 1H; H-19b), 1.10 (dt, J = 13.2, 6.8 Hz, 2H; H-18); **HRMS** (ESI+) m/z = 427.1709 $[M+H]^+$ found, $C_{20}H_{23}N_6O_5^+$ required 427.1724.

Methyl 2-((9*S*,*E*)-9-(dimethylcarbamoyl)-15-iodo-4,7-dioxo-11*H*-3,6,8-triaza-1(4,1)-triazola-5(1,3)-benzenacyclotridecaphane-3-yl)acetate (40c)

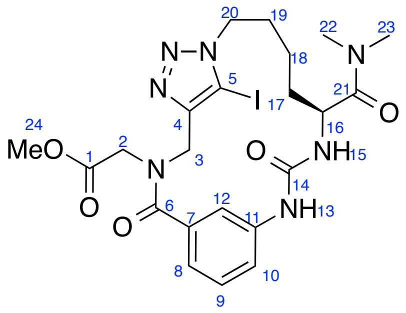

24c (8.2 mg, 8.0 µmol, 1.0 eq) was reacted according to GSP-8. After preparative HPLC (10-100% B), **40c** (2.7 mg, 4.5 µmol, 57%) was obtained as a white powder.

TLC R_f = 0.52 (CH_2Cl_2/MeOH 9:1); **HPLC** t_r = 7.79 min (5-100% B), peak area 100%; **IR** ν_{max} (neat)/cm^{-1} = 1741 m, 1620 s, 1587 m, 1543 m, 1420 m, 1209 s; **^{1}H NMR** (500 MHz, DMSO-d_6, 120 °C) δ = 8.45 (s, 1H; H-13), 7.53 (s, 1H; H-12), 7.31 (t, J = 7.8 Hz, 1H; H-9), 7.05 (d, J = 7.5 Hz, 1H; H-8), 6.85 (d, J = 8.1 Hz, 1H; H-10), 6.15 (d, J = 8.3 Hz, 1H; H-15), 4.62 (t, J = 8.7 Hz, 1H; H-16), 4.59-4.49 (m, 2H; H-2a andH-20a), 4.46 (d, J = 17.4 Hz, 1H; H-3a), 4.40 (d, J = 17.3 Hz, 1H; H-3b), 4.32 (d, J = 17.0 Hz, 1H; H-2b), 4.30-4.20 (m, 1H; H-20b), 3.73 (s, 3H; H-24), 2.93 (s; H-22 and H-23, coincides with water signal), 2.27-2.12 (m, 1H; H-19a), 1.92-1.77 (m, 1H; H-19b), 1.71-1.57 (m, 1H; H-17a), 1.54-1.45 (m, 1H; H-17b), 1.45-1.36 (m, 2H; H-18); **^{13}C NMR** (125 MHz, DMSO-d_6, 27 °C) major rotamer signals only, δ = 171.5 (C-21 and C-6), 169.6 (C-1), 154.1 (C-14), 146.7 (C-4), 140.0 (C-11), 135.1 (C-7), 129.1 (C-9), 119.8 (C-8), 118.4 (C-10), 114.4 (C-12), 80.6 (C-5), 51.8 (C-24), 49.8 (C-20), 48.6 (C-2), 46.9 (C-16), 46.6 (C-3), 36.5 (C-22 or C-23), 35.0 (C-22 or C-23), 34.0 (C-17), 29.8 (C-19), 23.2 (C-18); **HRMS** (ESI+) m/z = 598.1253 $[M+H]^+$ found, $C_{22}H_{29}N_7O_5{}^{127}I_1^+$ required 598.1269.

Methyl (*Z*)-2-(5,8,13-trioxo-21*H*-4,7,12,14-tetraaza-2(1,4)-triazola-1(1,4),6,11(1,3)-tribenzenacyclohexadecaphane-4-yl)acetate (41a)

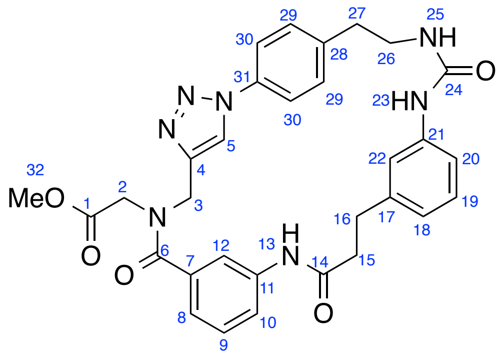

26a (13 mg, 0.013 mmol, 1.0 eq) was reacted according to GSP-8 and a white precipitate formed. The precipitate collected by vacuum filtration and rinsed with methanol (1 mL). **41a** (5.4 mg, 9.3 μmol, 72%) was obtained as a white solid.

TLC R_f = 0.53 (CH_2Cl_2/MeOH 10:1); **HPLC** t_r = 9.36 min (5-100% B), peak area 99%; **mp** 286-287 °C (H_2O); **IR** ν_{max} (neat)/cm^{-1} = 1749 m (C=O), 1685 s, 1632 s, 1606 m, 1588 m, 1521 s, 1468 m, 1422 s, 1213 s, 1176 m; **^{1}H NMR** (500 MHz, DMSO-d_6, 120 °C) δ = 9.67 (s, 1H; H-13), 8.45 (s, 1H; H-5), 7.94-7.68 (m, 4H; H-12, H-23 and H-30 × 2), 7.47 (d, J = 8.5 Hz, 3H; H-10 and H-29 × 2), 7.40 (t, J = 7.8 Hz, 1H; H-9), 7.18-7.02 (m, 3H; H-8, H-22 and H-19), 6.96 (d, J = 7.4 Hz, 1H; H-20), 6.79 (d, J = 7.5 Hz, 1H; H-18), 5.78 (t, J = 5.8 Hz, 1H; H-25), 4.65 (s, 2H; H-3), 4.29 (s, 2H; H-2), 3.68 (s, 3H; H-32), 3.52 (q, J = 5.8 Hz, 2H; H-26), 2.96-2.88 (m, 2H; H-27), 2.83 (H-16, coincides with water signal), 2.64 (t, J = 7.3 Hz, 2H; H-15); **HRMS** (ESI+) m/z = 582.2449 $[M+H]^+$ found, $C_{31}H_{32}N_7O_5^+$ required 582.2459.

Methyl 2-(5,8,13-trioxo-21*H*-4,7,12,14-tetraaza-2(1,5)-triazola-1(1,4),6,11(1,3)-tribenzenacyclohexadecaphane-4-yl)acetate (41b)

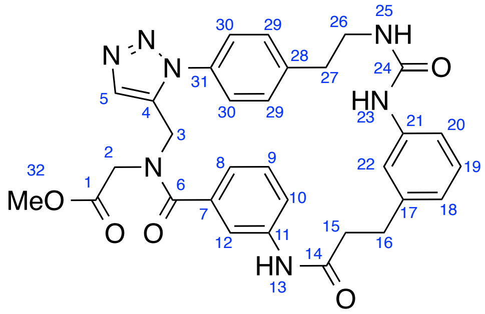

26b (18 mg, 0.018 mmol, 1.0 eq) was reacted according to GSP-8. After purification by preparative HPLC (5-100% B), **41b** (8.4 mg, 0.014 mmol, 82%) was obtained as a white powder.

TLC R_f = 0.11 (EtOAc); **HPLC** t_r = 8.68 min (5-100% B), peak area 100%; **mp** 280 °C decomposition (H_2O); **IR** ν_{max} (neat)/cm^{-1} = 1758 m (C=O), 1696 m, 1637 s, 1608 m, 1591 m, 1561 s, 1488 m, 1428 m, 1212 s, 1177 m; ^{1}**H NMR** (500 MHz, DMSO-d_6, 120 °C) δ = 9.48 (s, 1H; H-13), 7.90 (s, 1H; H-5), 7.85 (brs, 1H; H-23), 7.65 (t, J = 1.9 Hz, 1H; H-12), 7.44 (d, J = 8.1 Hz, 1H; H-10), 7.38-7.31 (m, 3H; H-29 × 2 and H-20), 7.26 (d, J = 8.0 Hz, 2H; H-30 × 2), 7.22 (t, J = 7.9 Hz, 1H; H-9), 7.10 (t, J = 7.8 Hz, 1H; H-19), 6.93 (t, J = 2.0 Hz, 1H; H-22), 6.83 (dt, J = 7.7, 1.3 Hz, 1H; H-8), 6.78 (d, J = 7.5 Hz, 1H; H-18), 5.63 (brs, 1H; H-25), 4.63 (s, 2H; H-3), 4.12 (s, 2H; H-2), 3.64 (s, 3H; H-32), 3.52-3.43 (m; H-26, coincides with water signal), 3.03-2.83 (m, 4H; H-27 and H-16), 2.74-2.54 (m, 2H; H-15); ^{13}**C NMR** (125 MHz, DMSO-d_6, 27 °C) major rotamer signals only, δ = 171.3 (C-6), 170.6 (C-14), 169.5 (C-1), 155.0 (C-24), 141.7 (C-17 or C-28), 141.5 (C-28 or C-17), 140.3 (C-21), 139.6 (C-11), 135.3 (C-4 and C-7), 133.4 (C-31), 132.2 (C-5), 130.0 (C-29 × 2), 128.8 × 2 (C-9 and C-19), 124.6 (C-30 × 2), 121.4 (C-18), 120.3 (C-10), 119.3 (C-8), 117.1 (C-22), 116.5 (C-12), 115.1 (C-20), 52.0 (C-32), 47.5 (C-2), 45.5 (C-3), 37.9 (C-15), 33.7 (C-27), 30.0 (C-16); **HRMS** (ESI+) m/z = 582.2446 $[M+H]^+$ found, $C_{31}H_{32}N_7O_5^+$ required 582.2459.

Methyl 2-((20*S*,*Z*)-20-(dimethylcarbamoyl)-4,7,12,18-tetraoxo-11*H*-3,6,11,13,17,19-hexaaza-1(4,1)-triazola-5,10(1,3),16(1,4)-tribenzenacyclotetracosaphane-3-yl)acetate (42a)

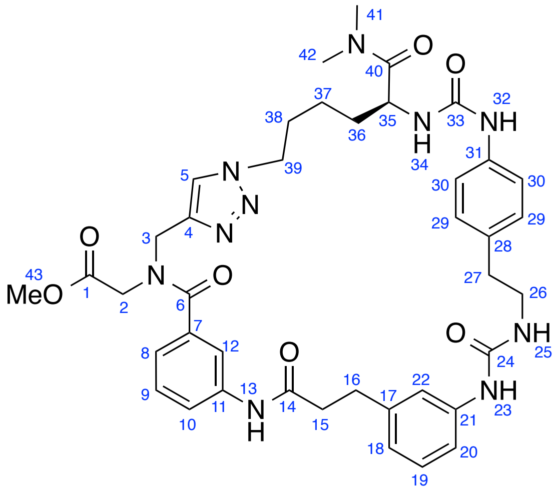

27a (20 mg, 0.016 mmol, 1.0 eq) was reacted according to GSP-8. After preparative HPLC (10-60% B), **42a** (9.4 mg, 7.7 µmol, 73%) was obtained as a white powder.

TLC R_f = 0.46 (CH_2Cl_2/MeOH 10:1); **HPLC** t_r = 8.78 min (5-100% B), peak area 99%; $[\alpha]_D^{25.4}$ = -28 (c = 0.128 in MeOH); **mp** 118 °C decomposition (H_2O); **IR** ν_{max} (neat)/cm^{-1} = 1747 w (C=O), 1623 s, 1547 s, 1410 m, 1310 m, 1202 s; **^{1}H NMR** (500 MHz, DMSO-d_6, 120 °C) due to interconversion, H-25 is missing, δ = 9.56 (s, 1H; H-13), 8.29 (s, 1H; H-32), 7.95 (brs, 1H; H-23), 7.86 (s, 1H; H-5), 7.73 (d, J = 8.2 Hz, 1H; H-10), 7.52 (s, 1H; H-12), 7.34 (t, J = 7.9 Hz, 1H; H-9), 7.31-7.24 (m, 3H; H-20 and H-30 × 2), 7.12-7.03 (m, 5H; H-19, H-29 × 2, H-22 and H-8), 6.77 (d, J = 7.5 Hz, 1H; H-18), 6.17 (s, 1H; H-34), 4.71 (dd, J = 8.4, 4.6 Hz, 1H; H-35), 4.60 (s, 2H; H-3), 4.33 (t, J = 7.0 Hz; H-39, coincides with water signal), 4.12 (s, 2H; H-2), 3.65 (s, 3H; H-43), 3.39 (q, J = 7.5 Hz, 2H H-26), 2.97 (brs, 6H; H-41 and H-42), 2.88 (t, J = 7.2 Hz, 2H; H-16), 2.70 (t, J = 6.0 Hz, 2H; H-27), 2.62 (t, J = 7.2 Hz, 2H; H-15), 1.94 (dt, J = 14.2, 7.1 Hz, 1H; H-38a), 1.87 (dt, J = 13.9, 7.1 Hz, 1H; H-38b), 1.78-1.67 (m, 1H; H-36a), 1.53 (dq, J = 14.5, 7.5 Hz, 1H; H-36b), 1.38 (quint, J = 7.5 Hz, 2H; H-37); **HRMS** (ESI+) m/z = 781.3785 $[M+H]^+$ found, $C_{40}H_{49}N_{10}O_7^+$ required 781.3780.

Methyl (*S*)-2-(20-(dimethylcarbamoyl)-4,7,12,18-tetraoxo-11*H*-3,6,11,13,17,19-hexaaza-1(5,1)-triazola-5,10(1,3),16(1,4)-tribenzenacyclotetracosaphane-3-yl)acetate (42b)

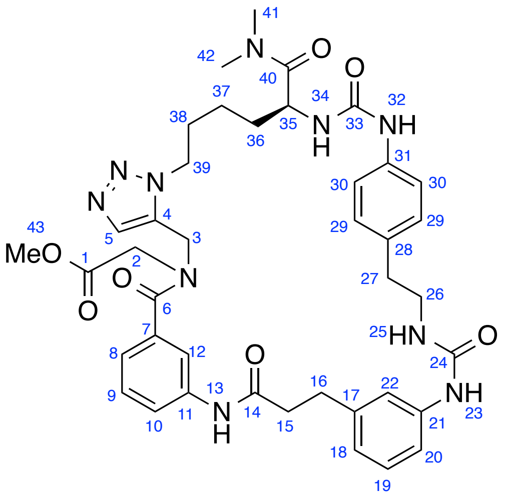

27b (9.2 mg, 7.6 µmol, 1.0 eq) was reacted according to GSP-8. After preparative HPLC (10-100% B), **42b** (5.8 mg, 0.0074 mmol, 98%) was obtained as a white powder.

TLC R_f = 0.41 (CH_2Cl_2/MeOH 10:1); **HPLC** t_r = 8.71 min (5-100% B), peak area 100%; $[\alpha]_D^{25.4}$ = -19 (c = 0.153 in MeOH); **mp** 118 °C decomposition (H_2O); **IR** ν_{max} (neat)/cm^{-1} = 3302 w, 2927 w, 1746 w (C=O), 1629 s, 1544 s, 1436 m, 1310 m, 1203 s; **^{1}H NMR** (500 MHz, DMSO-d_6, 120 °C) δ = 9.56 (s, 1H; H-13), 8.32 (brs, 1H; H-32), 7.93 (brs, 1H; H-23), 7.64 (s, 1H; H-5), 7.62 (d, J = 8.3 Hz, 1H; H-10), 7.58 (s, 1H; H-12), 7.34-7.28 (m, 2H; H-20 and H-9), 7.28 (d, J = 8.0 Hz, 2H; H-30 × 2), 7.12-

7.03 (m, 3H; H-19 and H-29 × 2), 6.98 (d, J = 7.6 Hz, 1H; H-8), 6.95 (s, 1H; H-22), 6.76 (d, J = 7.5 Hz, 1H; H-18), 6.19 (brs, 1H; H-34), 5.56 (brs, 1H; H-25), 4.79 (d, J = 16.5 Hz, 1H; H-3a), 4.76 (d, J = 16.5 Hz, 1H; H-3b), 4.72 (dd, J = 8.3, 4.6 Hz, 1H; H-35), 4.26 (t, J = 7.4 Hz, 2H; H-39), 4.07 (s, 2H; H-2), 3.59 (s, 3H; H-43), 3.41 (q, J = 5.4 Hz, 2H; H-26), 2.97 (s, 6H; H-41 and H-42), 2.87 (t, J = 6.9 Hz, 2H; H-16), 2.70 (t, J = 5.9 Hz, 2H; H-27), 2.61 (t, J = 6.9 Hz, 2H; H-15), 1.97-1.76 (m, 2H; H-38), 1.71 (dq, J = 13.2, 7.1 Hz, 1H; H-36a), 1.53 (dq, J = 14.4, 7.6 Hz, 1H; H-36b), 1.40 (quint, J = 7.6 Hz, 2H; H-37); **^{13}C NMR** (125 MHz, DMSO-d_6, 27 °C) δ = 171.8 (C-40), 171.2 (C-6), 170.5 (C-14), 169.4 (C-1), 154.9 (C-24), 154.6 (C-33), 141.7 (C-17), 140.4 (C-21), 139.5 (C-11), 138.4 (C-31), 135.5 (C-7), 133.9 (C-5), 132.6 (C-4), 132.0 (C-28), 129.1 (C-29 × 2 and C-9), 128.6 (C-19), 121.2 (C-18), 120.3 (C-8), 120.0 (C-10), 117.6 (C-30 × 2), 117.1 (C-22), 116.7 (C-12), 115.1 (C-20), 52.1 (C-43), 50.1 (C-2), 48.2 (C-35), 47.3 (C-39), 39. 4 (C-26, coincides with solvent signal), 37.8 (C-15 or C-3), 37.6 (C-3 or C-15), 36.6 (C-41 or C-42), 35.1 (C-42 or C-41), 34.4 (C-27), 32.3 (C-36), 30.0 (C-16), 29.5 (C-38), 22.3 (C-37); **HRMS** (ESI+) m/z = 781.3752 $[M+H]^+$ found, $C_{40}H_{49}N_{10}O_7^+$ required 781.3780.

Methyl (*Z*)-2-(6-(2-(cyclohexylamino)-2-oxo-1-phenylethyl)-4,7-dioxo-1^1*H*-3,6-diaza-1(4,1)-triazola-5(1,3)-benzenacyclotetradecaphane-3-yl)acetate (43a)

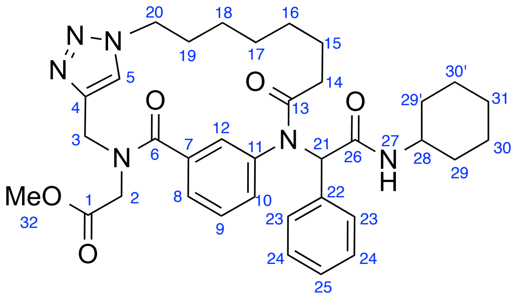

28a (13 mg, 0.012 mmol, 1.0 eq) was reacted according to GSP-8. After preparative HPLC (30-100% B), **43a** (5.0 mg, 8.0 μmol, 65%) was obtained as a clear oil.

TLC R_f = 0.18 (CH_2Cl_2/MeOH 40:1); **HPLC** t_r = 8.42 min (30-100% B), peak area 100%; **mp** 234-235 °C (H_2O); **IR** ν_{max} (neat)/cm^{-1} = 3277 w (N-H), 1743 m (C=O), 1648 s (C=O); **^{1}H NMR** (500 MHz, DMSO-d_6, 120 °C) δ = 7.90 (s, 1H; H-5), 7.36-7.17 (m, 5H; H-8, H-9, H-10, H-12, H-27), 7.17-7.13 (m, 2H; H-24), 7.13-7.08 (m, 2H; H-23), 6.10 (s, 1H; H-21), 4.62 (d, J = 15.6 Hz, 1H; H-3a), 4.56 (d, J = 15.6 Hz, 1H; H-3b), 4.39 (t, J = 6.0 Hz, 2H; H-20), 4.12 (d, J = 17.5 Hz, 1H; H-2a), 4.02 (d, J = 17.6 Hz, 1H; H-2b), 3.70 (s, 3H; H-32), 3.06 (m, 1H; H-28), 1.91 (t, J = 6.8 Hz, 2H; H-14), 1.88-1.76 (m, 3H; H-19 and either H-29a or H-29'a), 1.75-1.67 (m, 2H; either H-29a or H-29'a, and either H-

30a or H-30’a), 1.67-1.59 (m, 1H; either H-30a or H-30’a), 1.59-1.51 (m, 1H; H-31a), 1.44-1.35 (m, 2H; H-15), 1.35-1.27 (m, 3H; H-30b, H-30’b, and either H-29b or H-29’b), 1.27-1.16 (m, 2H; H-31b, and either H-29b or H-29’b), 1.16-0.87 (m, 6H; H-16, H-18, H-17); **HRMS** (ESI+) *m/z* = 629.3440 $[M+H]^+$ found, $C_{35}H_{45}N_6O_5^+$ required 629.3446.

Methyl 2-(6-(2-(cyclohexylamino)-2-oxo-1-phenylethyl)-4,7-dioxo-1^1H-3,6-diaza-1(5,1)-triazola-5(1,3)-benzenacyclotetradecaphane-3-yl)acetate (43b)

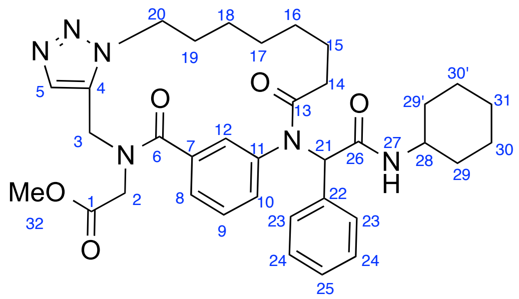

28b (70 mg, 0.066 mmol, 1.0 eq) was reacted according to GSP-8. After preparative HPLC (30-100% B), **43b** (22 mg, 0.035 mmol, 53%) was obtained as a pale yellow oil.

TLC R_f = 0.91 (CH_2Cl_2/MeOH 3:1); **HPLC** t_r = 8.58 min (30-100% B), peak area 99%; **IR** ν_{max} (neat)/cm^{-1} = 3287 w (N-H), 1744 m (C=O), 1643 m (C=O); **^{1}H NMR** (500 MHz, DMSO-d_6, 120 °C) δ = 7.67 (s, 1H; H-5), 7.38-7.18 (m, 5H; H-8, H-9, H-10, H-12, H-27), 7.16-7.10 (m, 3H; H-24, H-25), 7.10-7.04 (m, 2H; H-23), 6.13 (s, 1H; H-21), 4.75 (brs, 2H; H-3), 4.19 (t, *J* = 7.1 Hz, 2H; H-20), 4.04 (d, *J* = 17.5 Hz, 1H; H-2a), 3.96 (d, *J* = 17.6 Hz, 1H; H-2b), 3.66 (s, 3H; H-32), 3.62 (m, 1H; H-28), 1.95 (t, *J* = 7.3 Hz, 2H; H-14), 1.82-1.65 (m, 5H; H-19, H-29a, H-29’a, and either H-30a or H-30’a), 1.65-1.57 (m, 1H; either H-30a or H-30’a), 1.57-1.51 (m, 1H; H-31a), 1.49-1.36 (m, 2H; H-15), 1.36-1.20 (m, 3H; H-30b, H-30’b, either H-29b or H-29’b), 1.20-1.06 (m, 6H; H-16, H-18, either H-29b or H-29’b, H-31b), 1.06-0.97 (m, 2H; H-17); **^{13}C NMR** (125 MHz, DMSO-d_6, 120 °C) δ = 171.4 (C-13), 169.4 (C-6), 168.2 (C-1), 167.8 (C-26), 139.6 (C-11), 135.1 (C-22), 134.2 (C-7), 133.0 (C-5), 132.1 (C-8 or C-10), 131.7 (C-4), 129.2 (C-23), 128.4 (C-8 or C-10), 128.2 (C-12), 127.1 (C-24), 126.8 (C-25), 128.0 (C-9), 63.1 (C-21), 51.1 (C-32), 47.5 × 2 (C-2 and C-28), 46.9 (C-3), 33.2 (C-14), 31.4 × 2 (C-29 and C-29’), 28.0 (C-19), 26.4 (C-16), 26.3 (C-17), 24.6 (C-31), 24.3 (C-18), 23.8 (C-15), 23.7 (C-30 or C-30’), 23.6 (C-30 or C-30’); **HRMS** (ESI+) *m/z* = 629.3440 $[M+H]^+$ found, $C_{35}H_{45}N_6O_5^+$ required 629.3451.

Methyl (*Z*)-2-(6-(1-(4-bromophenyl)-2-(cyclohexylamino)-2-oxoethyl)-4,7-dioxo-1^1*H*-3,6-diaza-1(4,1)-triazola-5(1,3)-benzenacyclotetradecaphane-3-yl)acetate (44a)

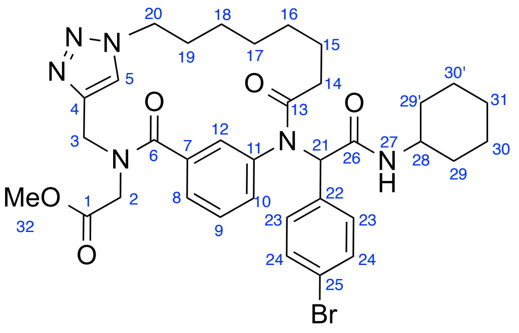

29a (25 mg, 0.022 mmol, 1.0 eq) was reacted according to GSP-8. After preparative HPLC (30-100% B), **44a** (9.0 mg, 0.013 mmol, 58%) was obtained as a white solid.

TLC R_f = 0.46 (CH_2Cl_2/MeOH 15:1); **HPLC** t_r = 9.24 min (30-100% B), peak area 95%; **mp** 235-237 °C (H_2O); **IR** ν_{max} (neat)/cm^{-1} = 3663 w (N-H), 1745 m (C=O), 1649 m (C=O); **^{1}H NMR** (500 MHz, DMSO-d_6, 120 °C) δ = 7.91 (s, 1H; H-5), 7.42 (d, *J* = 6.1 Hz, 1H; H-27), 7.36-7.30 (m, 3H; H-12, H-24), 7.30-7.25 (m, 2H; H-8, H-10), 7.24-7.17 (brs, 1H; H-9), 7.04 (d, *J* = 8.5 Hz, 2H; H-23), 6.05 (s, 1H; H-21), 4.61 (d, *J* = 15.8 Hz, 1H; H-3a), 4.57 (d, *J* = 16.1 Hz, 1H; H-3b), 4.38 (t, *J* = 6.0 Hz, 2H; H-20), 4.13 (d, *J* = 17.4 Hz, 1H; H-2a), 4.05 (d, *J* = 17.4 Hz, 1H; H-2b), 3.70 (s, 3H; H-32), 1.88 (t, *J* = 8.4 Hz, 2H; H-14), 1.86-1.75 (m, 3H; H-19, and either H-29a or H-29'a), 1.75-1.67 (m, 2H; either H-29a or H-29'a, and either H-30a or H-30'a), 1.67-1.59 (m, 1H; either H-30a or H-30'a), 1.59-1.52 (m, 1H; H-31a), 1.41-1.33 (m, 2H; H-15), 1.33-1.21 (m, 3H; H-30b, H-30'b, and either H-29b or H-29'b), 1.21-1.11 (m, 2H; H-31b, and either H-29b or H-29'b), 1.11-0.92 (m, 6H; H-16, H-17, H-18); **^{13}C NMR** (125 MHz, DMSO-d_6, 120 °C) δ = 171.5 (C-13), 169.1 (C-6), 168.4 (C-1), 167.5 (C-26), 142.5 (C-4), 139.6 (C-11), 135.1 (C-7), 134.6 (C-22), 131.3 (C-23), 131.2 (C-9), 130.1 (C-24), 128.5 (C-12), 128.2 (C-8 or C-10), 125.5 (C-8 or C-10), 122.3 (C-5), 120.4 (C-25), 62.8 (C-21), 51.1 (C-32), 48.8 (C-20), 48.2 (C-2), 47.6 (C-28), 43.5 (C-3), 33.2 (C-14), 31.4 (C-29 or C-29'), 31.3 (C-29 or C-29'), 27.9 (C-19), 27.4 (C-16), 26.5 (C-17), 24.7 (C-31), 24.6 (C-18), 23.9 (C-15), 23.7 (C-30 or C-30'), 23.6 (C-30 or C-30'); **HRMS** (ESI+) *m/z* = 707.2538 $[M+H]^+$ found, $C_{35}H_{44}BrN_6O_5^+$ required 707.2556.

Methyl 2-(6-(1-(4-bromophenyl)-2-(cyclohexylamino)-2-oxoethyl)-4,7-dioxo-1^1*H*-3,6-diaza-1(5,1)-triazola-5(1,3)-benzenacyclotetradecaphane-3-yl)acetate (44b)

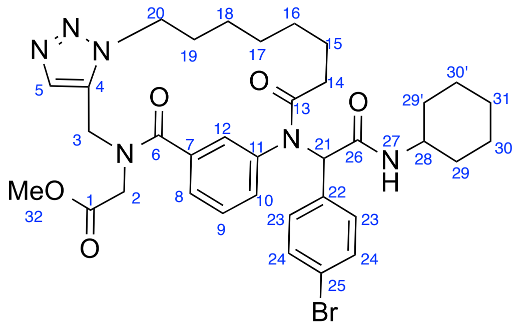

29b (94 mg, 0.082 mmol, 1.0 eq) was reacted according to GSP-8. After preparative HPLC (30-100% B), **44b** (3.3 mg, 4.6 µmol, 6.0%) was obtained as a white powder.

TLC R_f = 0.10 (CH_2Cl_2/MeOH 15:1); **HPLC** t_r = 4.27 min (50-100% B), peak area 100%; **IR** ν_{max} (neat)/cm^{-1} = 3273 w (N-H), 1750 m (C=O), 1648 s (C=O); **^{1}H NMR** (500 MHz, DMSO-d_6, 120 °C) δ = 7.70 (s, 1H; H-5), 7.51-7.08 (m, 3H; H-8, H-9, H-10), 7.43-7.36 (d, *J* = 7.1 Hz, 1H; H-27), 7.34-7.30 (m, 2H; H-24), 7.24-7.11 (m, 1H; H-12), 7.04 (d, *J* = 8.4 Hz, 2H; H-23), 6.09 (s, 1H; H-21), 4.80 (d, *J* = 16.3 Hz, 1H; H-3a), 4.72 (d, *J* = 15.7 Hz, 1H; H-3b), 4.20 (t, *J* = 7.1 Hz, 2H; H-20), 3.99 (d, *J* = 17.8 Hz, 1H; H-2a), 3.90 (d, *J* = 17.7 Hz, 1H; H-2b), 3.61 (m, 1H; H-28), 1.95 (t, *J* = 7.1 Hz, 2H; H-14), 1.79-1.65 (m, 5H; H-19, H-29a, H-29'a, and either H-30a or H-30'a), 1.65-1.58 (m, 1H; either H-30a or H-30'a), 1.58-1.50 (m, 1H; H-31a), 1.49-1.35 (m, 2H; H-15), 1.32-1.23 (m, 3H; H-30b, H-30'b, either H-29b or H-29'b), 1.17-1.07 (m, 6H; H-16, H-18, either H-29b or H-29'b, H-31b), 1.04-0.96 (m, 2H; H-17); **LCMS** (ESI+) m/z = 707.3 $[M+H]^+$ found.

Methyl (2*R)-2-methoxy-2-((2*S**,*Z*)-6-(2-methoxy-2-oxoethyl)-5-oxo-81*H*-3,6-diaza-8(4,1)-triazola-1,4(1,3)-dibenzenacyclododecaphane-2-yl)acetate (45a)**

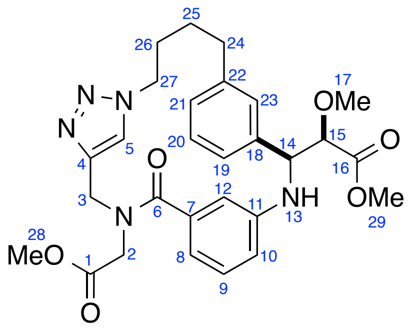

30a (47 mg, 0.050 mmol, 1.0 eq) was reacted according to GSP-8. After preparative HPLC (30-50% B), **45a** (28 mg, 0.052 mmol, quant.) was obtained as a white powder as a major side product.

TLC R_f = 0.14 (petroleum ether/EtOAc 1:3); **HPLC** t_r = 9.76 min (30-50% B), peak area 100%; $[\alpha]_D^{25.4}$ = -1 (c = 0.117 in MeOH); **mp** 72 °C decomposition (H_2O); **IR** ν_{max} (neat)/cm^{-1} = 1744 s (C=O), 1637 m, 1602 m, 1438 m, 1208 s; **^{1}H NMR** (500 MHz, DMSO-d_6, 120 °C) δ = 7.59 (s, 1H; H-5), 7.20 (dt, J = 7.5, 1.5 Hz, 1H; H-19), 7.18 (s, 1H; H-23), 7.16 (t, J = 7.5 Hz, 1H; H-20), 7.09 (dd, J = 8.2, 7.4 Hz, 1H; H-9), 7.01 (dt, J = 7.3, 1.5 Hz, 1H; H-21), 6.82 (ddd, J = 8.3, 2.5, 1.1 Hz, 1H; H-10), 6.53 (dt, J = 7.4, 1.3 Hz, 1H; H-8), 6.35 (s, 1H; H-12), 4.65 (d, J = 5.2 Hz; H-14, coincides with water signal), 4.58 (d, J = 15.5 Hz; H-3a, coincides with water signal), 4.54 (d, J = 15.4 Hz; H-3b, coincides with water signal), 4.41 (dt, J = 13.5, 6.6 Hz, 1H; H-27a), 4.36 (dt, J = 13.5, 6.6 Hz, 1H; H-27b), 4.08 (d, J = 5.2 Hz, 1H; H-15), 3.95 (s, 2H; H-2), 3.63 (s, 3H; H-28), 3.56 (s, 3H; H-29), 3.31 (s, 3H; H-17), 2.59 (dt, J = 13.4, 7.2 Hz, 1H; H-24a), 2.55 (dt, J = 13.4, 7.2 Hz, 1H; H-24b), 1.93-1.71 (m, 2H; H-26), 1.54-1.41 (m, 1H; H-25a), 1.41-1.27 (m, 1H; H-25b); **^{13}C NMR** (125 MHz, DMSO-d_6, 120 °C) δ = 170.6 (C-6), 169.5 (C-16), 168.6 (C-1), 147.0 (C-11), 142.5 (C-4), 141.1 (C-22), 139.1 (C-18), 135.9 (C-7), 128.1 (C-9), 127.4 (C-20), 127.2 (C-23), 126.7 (C-21), 123.7 (C-19), 122.4 (C-5), 115.1 (C-10), 113.7 (C-8), 108.9 (C-12), 83.5 (C-15), 58.7 (C-14), 57.6 (C-17), 51.0 (C-28), 50.6 (C-29), 48.8 (C-2), 48.4 (C-27), 42.3 (C-3), 33.4 (C-24), 28.2 (C-26), 26.9 (C-25); **HRMS** (ESI+) m/z = 536.2504 $[M+H]^+$ found, $C_{28}H_{34}N_5O_6^+$ required 536.2504.

Methyl (*R)-2-methoxy-2-((*S**)-6-(2-methoxy-2-oxoethyl)-5-oxo-81*H*-3,6-diaza-8(5,1)-triazola-1,4(1,3)-dibenzenacyclododecaphane-2-yl)acetate (45b)**

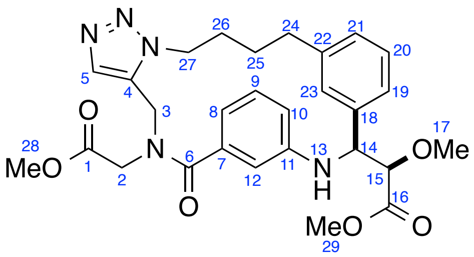

30b (29 mg, 0.031 mmol, 1.0 eq) was reacted according to GSP-8. After preparative HPLC (30-50% B), **45b** (13 mg, 0.024 mmol, 78%) was obtained as a white powder as a major side product.

TLC R_f = 0.18 (petroleum ether/EtOAc 1:3); **HPLC** t_r = 9.56 min (30-50% B), peak area 96%; $[\alpha]_D^{25.4}$ = -2 (c = 0.056 in MeOH); **mp** 70 °C decomposition (H_2O); **IR** ν_{max} (neat)/cm^{-1} = 1744 s (C=O), 1648 m, 1603 m, 1438 m, 1199 s; **^{1}H NMR** (500 MHz, DMSO-d_6, 120 °C) δ = 7.58 (s, 1H; H-5), 7.23-7.15

(m, 2H; H-19 and H-20), 7.11 (s, 1H; H-23), 7.03 (t, J = 7.8 Hz, 1H; H-9), 6.98 (d, J = 6.8 Hz, 1H; H-21), 6.81-6.68 (m, 1H; H-10), 6.44 (d, J = 7.4 Hz, 1H; H-8), 6.37 (brs, 1H; H-12), 4.71 (appbrs, 1H; H-14), 4.51 (brs, 2H; H-3), 4.14 (d, J = 5.0 Hz; H-15, coincides with water signal), 4.11 (appbrs; H-27, coincides with water signal), 3.88 (appbrs; H-2, coincides with water signal), 3.61 (s, 6H, H-28 and H-29), 3.33 (s, 3H; H-17), 2.62 (appbrs, 2H; H-24), 1.62 and 1.52 (brs × 2, 4H; H-26 and H-25); **^{13}C NMR** (125 MHz, DMSO-d_6, 120 °C) due to interconversion, C-23, C-5, C-3 and C-2 are missing, δ = 171.0 (C-6), 169.4 (C-16), 168.3 (C-1), 146.4 (C-11), 140.3 (C-22), 138.5 (C-18), 134.9 (C-7), 131.9 (C-4), 128.2 (C-9), 127.6 (C-20), 126.9 (C-21), 124.4 (C-19), 115.6 (C-10), 114.4 (C-8), 109.7 (C-12), 83.1 (C-15), 58.7 (C-14), 57.6 (C-17), 50.9 (C-28 or C-29), 50.7 (C-29 or C-28), 46.9 (C-27), 33.5 (C-24), 27.8 (C-25 or C-26), 27.7 (C-26 or C-25); **HRMS** (ESI+) m/z = 536.2509 [M+H]$^+$ found, $C_{28}H_{34}N_5O_6^+$ required 536.2504.

Methyl (2*R)-2-methoxy-2-((2*R**,*Z*)-6-(2-methoxy-2-oxoethyl)-5-oxo-81*H*-3,6-diaza-8(4,1)-triazola-1,4(1,3)-dibenzenacyclododecaphane-2-yl)acetate (46a)**

and **methyl 2-((22*R**,23*R**,*Z*)-23-methoxy-24,4-dioxo-71*H*-5-aza-7(4,1)-triazola-2(2,1)-azetidina-1,3(1,3)-dibenzenacycloundecaphane-5-yl)acetate (46c)**

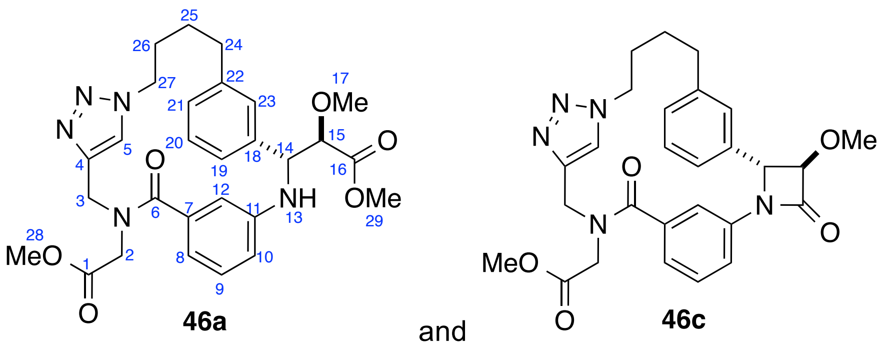

46a and **46c**

31a (51 mg, 0.055 mmol, 1.0 eq) was reacted according to GSP-8. After preparative HPLC (30-50% B), **46a** (16 mg, 0.030 mmol, 55%) was obtained as a white powder as a major side product. **46c** (1.0 mg, 3.3 mmol, 4%) was obtained as a white powder as a minor product.

46a

TLC R_f = 0.14 (petroleum ether/EtOAc 1:3); **HPLC** t_r = 9.09 min (30-50% B), peak area 92%; **[α]$_D^{25.4}$** = -3 (c = 0.031 in MeOH); **mp** 82 °C decomposition (H_2O); **IR** ν_{max} (neat)/cm^{-1} = 1745 s (C=O), 1639 m, 1603 m, 1459 m, 1208 s, 1126 s; **^{1}H NMR** (500 MHz, DMSO-d_6, 120 °C) δ = 7.57 (s, 1H; H-5), 7.19 (dt, J = 7.7, 1.5 Hz, 1H; H-19), 7.16 (s, 1H; H-23), 7.14 (t, J = 7.7 Hz, 1H; H-20), 7.10 (t, J = 7.7

Hz, 1H; H-9), 6.99 (dt, J = 7.3, 1.5 Hz, 1H; H-21), 6.80 (ddd, J = 8.3, 2.5, 1.0 Hz, 1H; H-10), 6.55 (dt, J = 7.4, 1.3 Hz, 1H; H-8), 6.40 (brs, 1H; H-12), 4.63 (d, J = 6.6 Hz, 1H; H-14), 4.59 (d, J = 15.5 Hz, 1H; H-3a), 4.52 (d, J = 15.5 Hz, 1H; H-3b), 4.40 (dt, J = 13.8, 6.7 Hz, 1H; H-27a), 4.37 (dt, J = 13.8, 6.7 Hz, 1H; H-27b), 4.11 (d, J = 6.5 Hz, 1H; H-15), 3.98 (s, 2H; H-2), 3.64 (s, 3H; H-28 or H-29), 3.63 (s, 3H; H-28 or H-29), 3.27 (s, coincides with water signal; H-17), 2.59 (ddd, J = 17.2, 8.3, 6.6 Hz, 1H; H-24a), 2.56-2.52 (m, 1H, coincides with solvent signal; H-24b), 1.81 (quint, J = 8.8 Hz, 2H; H-26), 1.51-1.31 (m, 2H; H-25); **^{13}C NMR** (125 MHz, DMSO-d_6, 120 °C) δ = 170.6 (C-6), 169.6 (C-16), 168.6 (C-1), 146.6 (C-11), 142.5 (C-4), 140.8 (C-22), 138.8 (C-18), 135.9 (C-7), 128.1 (C-9), 127.7 (C-23), 127.2 (C-20), 126.6 (C-21), 123.9 (C-19), 122.4 (C-5), 115.1 (C-10), 113.8 (C-8), 109.2 (C-12), 83.0 (C-15), 58.1 (C-14), 57.4 (C-17), 51.0 (C-28 or C-29), 50.6 (C-29 or C-28), 48.7 (C-2), 48.3 (C-27), 42.3 (C-3), 33.4 (C-24), 28.2 (C-26), 26.8 (C-25); **HRMS** (ESI+) m/z = 536.2526 $[M+H]^+$ found, $C_{28}H_{34}N_5O_6^+$ required 536.2504.

46c

TLC R_f = 0.40 (EtOAc); **HPLC** t_r = 9.43 min (30-50% B), peak area 72%; **IR** ν_{max} (neat)/cm^{-1} = 2934 w, 1748 s (C=O), 1683 m, 1641 m, 1456 m, 1371 m, 1203 s, 1129 s; **HRMS** (ESI+) m/z = 504.2252 $[M+H]^+$ found, $C_{27}H_{30}N_5O_5^+$ required 504.2241.

Methyl 2-((22*R,23*R**)-23-methoxy-24,4-dioxo-71*H*-5-aza-7(5,1)-triazola-2(2,1)-azetidina-1,3(1,3)-dibenzenacycloundecaphane-5-yl)acetate (46b)**

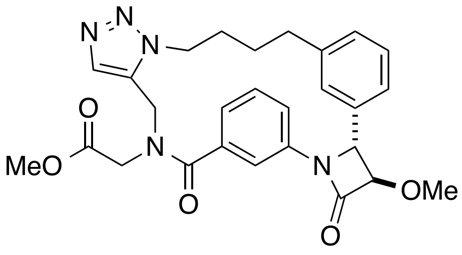

31b (3.7 mg, 4.0 µmol, 1.0 eq) was reacted according to GSP-8. After preparative HPLC (30-50% B), **46b** (1.0 mg, 1.2 µmol, 50%) was obtained as a colourless film.

TLC R_f = 0.16 (petroleum ether/EtOAc 1:3); **HPLC** t_r = 9.32 min (30-50% B), peak area 100%; **IR** ν_{max} (neat)/cm^{-1} = 2926 m, 1748 s (C=O), 1652 m, 1451 m, 1373 m, 1203 s, 1131 s; **NMR** Appropriate NMR data could not be obtained due to the existence of rotamers at a temperature range of 25-120 °C and partial decomposition at high temperature range 90-120 °C; **HRMS** (ESI+) m/z = 504.2261 $[M+H]^+$ found, $C_{27}H_{30}N_5O_5^+$ required 504.2241.

Methyl 2-((21*R,24*R**,*Z*)-26,4-dioxo-71*H*-23,5-diaza-7(4,1)-triazola-2(2,3)-bicyclo[2.2.2]octana-1,3(1,3)-dibenzenacycloundecaphane-5-yl)acetate (47)**

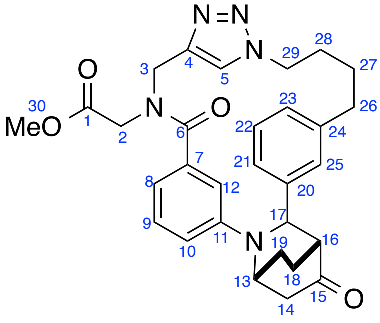

33 (11 mg, 0.012 mmol, 1.0 eq) was reacted according to GSP-8. After preparative HPLC (30-70% B, without addition of TFA), **47** (4.2 mg, 8.0 µmol, 67%) was obtained as a white powder.

TLC R_f = 0.16 (CH_2Cl_2/EtOAc 1:1); **HPLC** t_r = 7.32 min (30-100% B), peak area 98%; **IR** ν_{max} (neat)/cm^{-1} = 1726 s (C=O), 1641 s, 1598 s, 1456 m, 1209 s, 1176 s; ^{1}**H NMR** (500 MHz, DMSO-d_6, 120 °C) δ = 7.46 (s, 1H; H-5), 7.29-7.20 (m, 3H; H-9, H-22 and either H-21 or H-23), 7.15 (s, 1H; H-25), 7.10-7.01 (m, 2H; H-10 and either H-21 or H-23), 6.63 (d, J = 7.4 Hz, 1H; H-8), 6.16 (s, 1H; H-12), 4.69 (d, J = 2.8 Hz, 1H; H-17), 4.64 (d, J = 15.4 Hz, 1H; H-3a), 4.59 (dt, J = 6.0, 3.0 Hz, 1H; H-13), 4.51 (d, J = 15.4 Hz, 1H; H-3b), 4.44-4.26 (m, 2H; H-29), 3.85 (s, 2H; H-2), 3.59 (s, 3H; H-30), 2.62 (t, J = 7.0 Hz, 2H; H-26), 2.57 (dt, J = 18.5, 3.1 Hz, 1H; H-14a), 2.50 (H-16, coincides with solvent signal), 2.45 (dd, J = 18.6, 1.9 Hz, 1H; H-14b), 2.29-2.16 (m, 1H; H-19a), 1.99-1.91 (m, 1H; H-19b), 1.87-1.71 (m, 2H; H-28), 1.66-1.59 (m, 2H; H-18), 1.52-1.42 (m, 1H; H-27a), 1.43-1.35 (m, 1H; H-27b); ^{13}**C NMR** (125 MHz, DMSO-d_6, 27 °C) major rotamer signals only, due to interconversion, C-2 and C-3 are missing, δ = 212.7 (C-15), 171.0 (C-6), 169.5 (C-1), 148.0 (C-11), 143.2 (C-4), 143.1 (C-24), 139.8 (C-20), 136.4 (C-7), 129.3 (C-9), 128.2 (C-22), 127.7 (C-23), 127.0 (C-25), 123.3 (C-5), 122.9 (C-21), 114.2 (C-8), 113.4 (C-10), 110.6 (C-12), 62.0 (C-17), 52.0 (C-30), 50.3 (C-16), 49.2 (C-29), 48.0 (C-13), 42.1 (C-14), 34.4 (C-26), 29.2 (C-28), 28.7 (C-27), 25.4 (C-19), 15.6 (C-18); **HRMS** (ESI+) m/z = 550.2493 $[M+Na]^+$ found, $C_{30}H_{33}N_5O_4Na^+$ required 550.2425.

Methyl (*Z*)-2-(23-(3-hydroxypropyl)-3-oxo-61*H*-4-aza-2(2,5)-quinolina-6(4,1)-triazola-1(1,3)-benzenacyclodecaphane-4-yl)acetate (49e)

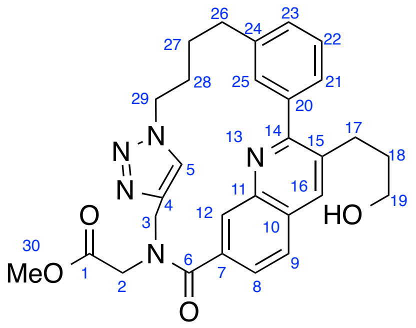

25e (3.4 mg, 3.6 µmol, 1.0 eq) was reacted according to GSP-8. After preparative HPLC (10-100% B, without addition of TFA), **49e** (0.67 mg, 1.3 µmol, 36%) was obtained as a white powder.

TLC R_f = 0.28 (EtOAc); **HPLC** t_r = 10.15 min (5-100% B, without addition of TFA), peak area 99%; **IR** ν_{max} (neat)/cm^{-1} = 1750 m (C=O), 1630 s, 1619 s, 1467 m, 1424 m, 1172 s; **^{1}H NMR** (500 MHz, DMSO-d_6, 120 °C) δ = 8.28 (s, 1H; H-16), 8.04 (d, J = 8.4 Hz, 1H; H-9), 7.97 (s, 1H; H-5), 7.83 (s, 1H; H-12), 7.69 (dd, J = 8.4, 1.7 Hz, 1H; H-8), 7.67 (s, 1H; H-25), 7.60 (d, J = 7.6 Hz, 1H; H-21), 7.38 (t, J = 7.6 Hz, 1H; H-22), 7.30 (d, J = 7.5 Hz, 1H; H-23), 4.57 (s, 2H; H-2 or H-3), 4.54 (s, 2H; H-2 or H-3), 4.51-4.42 (m, 2H; H-29), 3.74 (s, 3H; H-30), 3.50 (t, J = 6.3 Hz, 2H; H-19), 3.20-3.05 (t, J = 7.6 Hz, 2H; H-17), 2.91 (s; H-26 coincides with water signal), 2.14 (quint, J = 7.9 Hz, 2H; H-28), 1.91-1.84 (m, 2H; H-18), 1.85-1.77 (m, 2H; H-27); **^{13}C NMR** (125 MHz, DMSO-d_6, 27 °C) δ = 171.3 (C-6), 169.6 (C-1), 160.1 (C-14), 144.4 (C-11), 143.9 (C-4), 141.1 (C-24), 140.1 (C-20), 135.3 (C-16), 134.9 (C-15), 133.8 (C-7), 133.3 (C-25), 128.8 (C-23), 128.4 (C-8), 128.0 × 2 (C-9 and C-10), 127.7 (C-22), 125.3 (C-12), 125.0 (C-21), 123.2 (C-5), 60.2 (C-19), 51.9 (C-30), 49.0 (C-2), 48.7 (C-29), 45.8 (C-3), 33.2 (C-18), 32.4 (C-26), 28.7 (C-17), 28.0 (C-28), 26.8 (C-27); **HRMS** (ESI+) m/z = 514.2470 [M+H]$^+$ found, $C_{29}H_{32}N_5O_4^+$ required 514.2449.

Methyl (*Z*)-2-(23-(3-hydroxypropyl)-3-oxo-61*H*-4-aza-2(2,5)-quinolina-6(4,1)-triazola-1(1,3)-benzenacyclodecaphane-4-yl)acetate (49f)

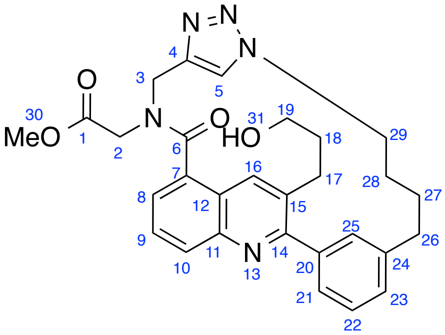

25f (10.0 mg, 0.0106 mmol, 1.0 eq) was reacted according to GSP-13. After preparative HPLC (20-70% B, without addition of TFA), **49f** (3.47 mg, 6.76 µmol, 64%) was obtained as a white powder.

TLC R_f = 0.13 (EtOAc); **HPLC** t_r = 8.85 min (5-100% B, without addition of TFA), peak area 99%; **IR** ν_{max} (neat)/cm^{-1} = 1743 m (C=O), 1644 s, 1426 m, 1402 m, 1200 s, 1179 s; ^{1}**H NMR** (500 MHz, DMSO-d_6, 120 °C) δ = 8.05 (s, 1H; H-16), 8.01 (d, J = 8.5 Hz, 1H; H-10), 7.77 (dd, J = 8.4, 7.0 Hz, 1H; H-9), 7.65 (d, J = 7.6 Hz, 1H; H-21), 7.55 (dd, J = 7.0, 1.0 Hz, 1H; H-8), 7.46 (t, J = 7.6 Hz, 1H; H-22), 7.23 (d, J = 7.7 Hz, 1H; H-23), 6.78 (s, 1H; H-5), 6.47 (s, 1H; H-25), 4.68 (d, J = 17.0 Hz, 1H; H-2a), 4.46 (d, J = 17.0 Hz, 1H; H-2b), 4.31-4.14 (m, 3H; H-29 and H-3a), 3.84 (d, J = 17.7 Hz, 1H; H-3b), 3.78 (s, 3H; H-30), 3.26 (t, J = 6.4 Hz, 2H; H-19), 2.79 (H-26, coincides with water signal), 2.49-2.41 (m; H-17, coincides with solvent signal), 1.86-1.76 (m, 2H; H-27), 1.76-1.69 (m, 2H; H-28), 1.55 (quint, J = 6.7 Hz, 2H; H-18); ^{13}**C NMR** (125 MHz, DMSO-d_6, 27 °C) δ = 169.8 (C-6), 169.4 (C-1), 161.4 (C-14), 144.1 (C-7), 142.0 (C-4), 139.6 (C-20), 139.4 (C-24), 133.6 (C-15), 133.4 × 2 (C-16 and C-11), 130.1 (C-25), 129.4 (C-10), 128.8 × 2 (C-9 and C-22), 128.6 (C-23), 123.6 (C-8), 123.1 (C-21), 122.9 (C-12), 121.6 (C-5), 59.9 (C-19), 52.0 (C-30), 48.2 (C-29 or C-2), 48.1 (C-29 or C-2), 45.5 (C-3), 33.1 (C-18), 30.3 (C-26), 28.9 (C-17), 26.4 (C-28), 24.5 (C-27); **HRMS** (ESI+) m/z = 514.2453 $[M+H]^+$ found, $C_{29}H_{32}N_5O_4^+$ required 514.2449.

Methyl 2-((24*aS,25*S**,210*bS**,*Z*)-3-oxo-23,24,24*a*,25,26,210*b*-hexahydro-22*H*,61*H*-4-aza-2(5,8)-pyrano[3,2-*c*]quinolina-6(4,1)-triazola-1(1,3)-benzenacyclodecaphane-4-yl)acetate (49a)**

and **methyl (*Z*)-2-(23-(3-hydroxypropyl)-3-oxo-61*H*-4-aza-2(2,5)-quinolina-6(4,1)-triazola-1(1,3)-benzenacyclodecaphane-4-yl)acetate (49e)**

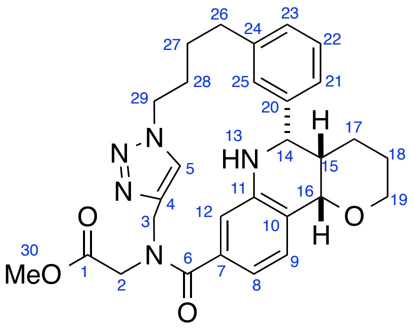

49a and

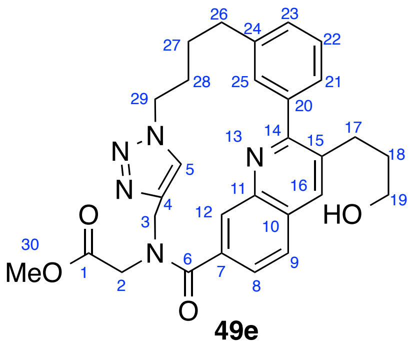

49e

25a (9.1 mg, 9.6 µmol, 1.0 eq) was reacted according to GSP-8. After purification by preparative HPLC (20-70% B, without addition of TFA), **49a** (2.0 mg, 0.0039 mmol, 40%) was obtained as a white powder. **49e** (1.0 mg, 0.0040 mmol, 20%) was obtained as a side product.

49a

TLC R_f = 0.54 (EtOAc); **HPLC** t_r = 11.38 min (5-100% B, without addition of TFA), peak area 89%; **IR** ν_{max} (neat)/cm^{-1} = 3380 m (NH), 2934 w, 1731 m (C=O), 1643 s, 1612 m, 1575 m, 1490 m, 1448 m, 1213 s; **HRMS** (ESI+) *m/z* = 516.2611 [M+H]$^+$ found, $C_{29}H_{34}N_5O_4^+$ required 516.2605. **49a** underwent aromatisation at high temperature (120 °C) whilst NMR spectra were initially recorded; therefore, NMR was then recorded at 27 °C and NMR data was reported as a mixture of **49a**/**49e** (5:3). The numbering scheme of the aromatised compound **3.211** was indicated using a number followed by a prime.

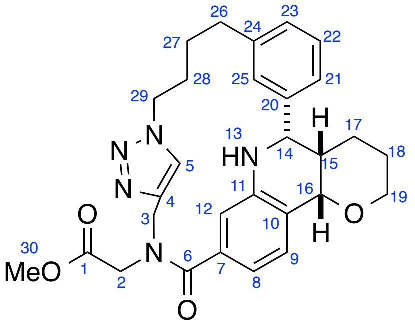

49a and

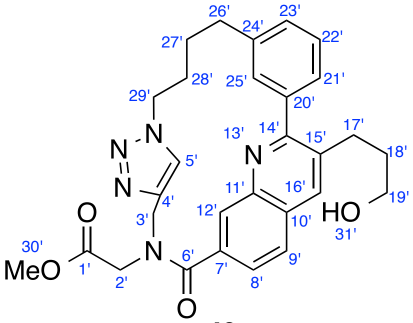

49e

^{1}**H NMR** (500 MHz, DMSO-d_6, 27 °C) δ = 8.37 (s, 0.6H; H-16'), 8.09 (d, J = 8.9 Hz, 0.6H; H-9'), 8.01 (s, 0.6H; H-5'), 7.87 (s, 1H; H-5), 7.73-7.66 (m, 1.2H; H-8' and H-12'), 7.65-7.57 (m, 1.2H; H-25' and H-21'), 7.39 (t, J = 7.9 Hz, 0.6H; H-22'), 7.34 (t, J = 1.6 Hz, 1H; H-25), 7.32 (dt, J = 7.6, 1.5 Hz, 0.6H; H-23'), 7.26 (t, J = 7.5 Hz, 1H; H-22), 7.22 (d, J = 8.1 Hz, 1H; H-9), 7.15 (d, J = 7.4 Hz, 1H; H-21), 7.10 (d, J = 7.7 Hz, 1H; H-23), 6.62 (dd, J = 7.7, 1.6 Hz, 1H; H-8), 6.51 (d, J = 1.6 Hz, 1H; H-12), 6.14 (s, 1H; H-13), 5.23 (d, J = 4.9 Hz, 1H; H-16), 4.77 (d, J = 3.7 Hz, 1H; H-14), 4.62-4.48 (m, 4.6H; H-2', H-29a, H-3' and H-29'), 4.48-4.42 (m, 1H; H-29b), 4.42-4.34 (m, 2H; H-2a and H-3a), 4.27 (d, J = 16.4 Hz, 1H; H-3b), 4.20 (d, J = 17.2 Hz, 1H; H-2b), 3.72 (s, 2H; H-30'), 3.69 (s, 3H; H-30), 3.57-3.50 (H-19a, coincides with water signal), 3.43 (H-19b and H-19', coincides with water signal), 3.11-3.05 (m, 1.2H; H-17'), 2.82 (t, J = 5.8 Hz, 1.2H; H-26'), 2.80-2.74 (m, 1H; H-26a), 2.69 (ddd, J = 14.7, 8.0, 4.5 Hz, 1H; H-26b), 2.19-2.13 (m, 1H; H-15), 2.13-2.08 (H-28a and H-28', coincides with CH_3CN signal), 2.07 (s, CH_3CN signal), 1.92-1.85 (m, 1H; H-28b), 1.83-1.65 (m, 3.4H; H-18', H-27' and H-27a), 1.60-1.50 (m, 1H; H-27b), 1.42-1.35 (m, 2H; H-18), 1.29-1.17 (m, 1H; H-17a), 1.15-1.06 (m, 1H; H-17b); ^{13}**C NMR** (125 MHz, DMSO-d_6, 27 °C) δ = 172.5 (C-6), 171.3 (C-6'), 169.6 (C-1'), 169.5 (C-1), 160.1 (C-14'), 144.4 (C-11), 144.3 (C-11'), 143.9 (C-4'), 143.4 (C-4), 141.1 × 2 (C-20' and C-20), 140.2 (C-24), 140.0 (C-24'), 135.4 (C-16'), 134.9 (C-15'), 134.6 (C-7), 133.9 (C-7'), 133.3 (C-25'), 128.8 (C-23'), 128.3 × 2 (C-22 and C-8'), 128.0 (C-9' and C-10'), 127.9 (C-23), 127.7 (C-22'), 127.1 (C-9 or C-25), 127.0 (C-9 or C-25), 125.3 (C-12'), 125.1 (C-21), 125.0 (C-21'), 123.2 (C-5'), 122.4 (C-5), 119.0 (C-10), 118.1 ($CH_3\underline{C}N$), 113.1 (C-8), 112.1 (C-12), 71.4 (C-16), 60.2 (C-19), 60.0 (C-19'), 56.4 (C-14), 51.9 × 2 (C-30 and C-30'), 49.0 × 2 (C-29 and C-2'), 48.7 (C-29'), 47.3 (C-3), 45.8 (C-2), 45.5 (C-3'), 37.4 (C-15), 33.2 (C-18'), 32.4 (C-26'), 32.1 (C-26), 28.7 (C-17'), 28.0 (C-28'), 27.3 (C-28), 26.8 (C-27'), 25.7 (C-27), 25.4 (C-18), 19.3 (C-17), 1.2 ($\underline{C}H_3CN$).

3.211

TLC R_f = 0.28 (EtOAc); **HPLC** t_r = 10.15 min (5-100% B, without addition of TFA), peak area 98%; **IR** ν_{max} (neat)/cm^{-1} = 1744 m (C=O), 1638 s, 1459 m, 1424 m, 1200 s, 1178 s; ^{1}**H NMR** (500 MHz, DMSO-d_6, 120 °C) δ = 8.28 (s, 1H; H-16), 8.03 (d, J = 8.4 Hz, 1H; H-9), 7.97 (s, 1H; H-5), 7.84 (s, 1H; H-12), 7.69 (dd, J = 8.4, 1.7 Hz, 1H; H-8), 7.67 (t, J = 1.6 Hz, 1H; H-25), 7.60 (d, J = 7.6 Hz, 1H; H-21), 7.38 (t, J = 7.6 Hz, 1H; H-22), 7.30 (d, J = 7.6 Hz, 1H; H-23), 4.57 (s, 2H; H-2 or H-3), 4.54 (s, 2H; H-3 or H-2), 4.50-4.43 (m, 2H; H-29), 3.74 (s, 3H; H-30), 3.50 (t, J = 6.3 Hz, 2H; H-19), 3.20-3.06 (t, J = 7.5 Hz 2H; H-17), 2.88 (H-26, coincides with water signal), 2.21-2.07 (m, 2H; H-28), 1.93-1.86 (m, 2H; H-18), 1.87-1.75 (m, 2H; H-27); ^{13}**C NMR** (125 MHz, DMSO-d_6, 27 °C) major rotamer signals only, δ = 171.3 (C-6), 169.6 (C-1), 160.1 (C-14), 144.4 (C-11), 143.9 (C-4), 141.1 (C-24), 140.1 (C-20), 135.3 (C-16), 134.9 (C-15), 133.8 (C-7), 133.3 (C-25), 128.8 (C-23), 128.4 (C-8), 128.0 × 2 (C-9 and C-10), 127.7 (C-22), 125.3 (C-12), 125.0 (C-21), 123.2 (C-5), 60.2 (C-19), 51.9 (C-30), 49.0 (C-2), 48.7 (C-29), 45.8 (C-3), 33.2 (C-18), 32.4 (C-26), 28.7 (C-17), 28.0 (C-28),

26.8 (C-27); **HRMS** (ESI+) *m/z* = 514.2452 [M+H]$^+$ found, $C_{29}H_{32}N_5O_4^+$ required 514.2449. Note: these data are consistent with the data reported above.

Methyl 2-((24*aS,25*S**,210*bS**,*Z*)-3-oxo-23,24,24*a*,25,26,210*b*-hexahydro-22*H*,61*H*-4-aza-2(5,8)-pyrano[3,2-*c*]quinolina-6(4,1)-triazola-1(1,3)-benzenacyclodecaphane-4-yl)acetate (49b)**

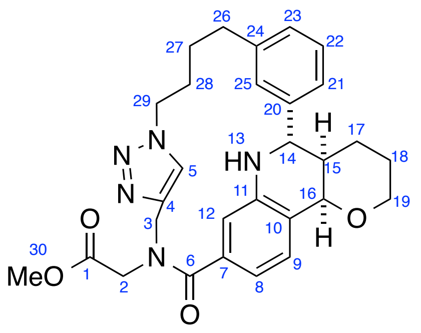

25b (14.8 mg, 0.0156 mmol, 1.0 eq) was reacted according to GSP-8. After preparative HPLC (20-70% B, without addition of TFA), **49b** (6.7 mg, 0.013 mmol, 83%) was obtained as a white powder.

TLC R_f = 0.17 (CH_2Cl_2/EtOAc 1:1); **HPLC** t_r = 11.16 min (5-100% B, without addition of TFA), peak area 99%; **IR** ν_{max} (neat)/cm^{-1} = 2933 w, 1745 m (C=O), 1645 s, 1616 s, 1460 m, 1423 m, 1209 s, 1179 s, 1082 m; ^{1}**H NMR** (500 MHz, DMSO-d_6, 120 °C) δ = 7.63 (s, 1H; H-5), 7.23 (t, *J* = 7.4 Hz, 1H; H-22), 7.20 (s, 1H; H-25), 7.17 (d, *J* = 7.6 Hz, 1H; H-21), 7.16 (d, *J* = 8.0 Hz, 1H; H-9), 7.07 (d, *J* = 7.5 Hz, 1H; H-23), 6.62-6.53 (m, 2H; H-12 and H-8), 5.86 (s, 1H; H-13), 4.63 (d, *J* = 16.6 Hz, 1H; H-3a), 4.54-4.50 (m, 1H; H-2a), 4.49 (s, 1H; H-14), 4.41 (dt, *J* = 13.8, 6.9 Hz, 1H; H-29a), 4.34 (s, 1H; H-16), 4.33 (d, *J* = 19.3 Hz, 1H; H-3b), 4.33-4.26 (m, 1H; H-29b), 4.12 (d, *J* = 17.0 Hz, 1H; H-2b), 3.88-3.79 (m, 1H; H-19a), 3.70 (s, 3H; H-30), 3.65 (ddd, *J* = 11.4, 8.6, 3.2 Hz, 1H; H-19b), 2.63 (dt, *J* = 14.1, 7.0 Hz, 1H; H-26a), 2.57 (dt, *J* = 14.1, 7.0 Hz, 1H; H-26b), 2.16-2.06 (m, 1H; H-15), 1.99-1.90 (m, 1H; H-28a), 1.90-1.80 (m, 2H; H-28b and H-18a), 1.76 (ddt, *J* = 13.7, 9.0, 4.2 Hz, 1H; H-17a), 1.67-1.57 (m, 1H; H-27a), 1.60-1.45 (m, 2H; H-17b and H-18b), 1.47-1.37 (m, 1H; H-27b); ^{13}**C NMR** (125 MHz, DMSO-d_6, 27 °C) δ = 172.2 (C-6), 169.4 (C-1), 144.5 (C-11), 143.7 (C-20), 143.0 (C-4), 141.4 (C-24), 136.3 (C-7), 130.1 (C-9), 128.0 (C-23), 127.9 (C-22), 126.6 (C-25), 126.0 (C-21), 122.2 (C-5), 119.5 (C-10), 111.4 (C-8), 111.2 (C-12), 72.9 (C-16), 66.6 (C-19), 53.6 (C-14), 51.9 (C-30), 49.1 (C-29), 47.3 (C-2), 45.2 (C-3), 36.8 (C-15), 33.3 (C-26), 27.7 (C-28), 26.2 (C-27), 23.7 (C-17), 22.0 (C-18); **HRMS** (ESI+) *m/z* = 516.2606 [M+H]$^+$ found, $C_{29}H_{34}N_5O_4^+$ required 516.2605.

Methyl 2-((24*aR,25*R**,210*bR**,*Z*)-3-oxo-23,24,24*a*,25,26,210*b*-hexahydro-22*H*,61*H*-4-aza-2(5,10)-pyrano[3,2-*c*]quinolina-6(4,1)-triazola-1(1,3)-benzenacyclodecaphane-4-yl)acetate (49c)**

and **methyl (*Z*)-2-(23-(3-hydroxypropyl)-3-oxo-61*H*-4-aza-2(2,5)-quinolina-6(4,1)-triazola-1(1,3)-benzenacyclodecaphane-4-yl)acetate (49f)**

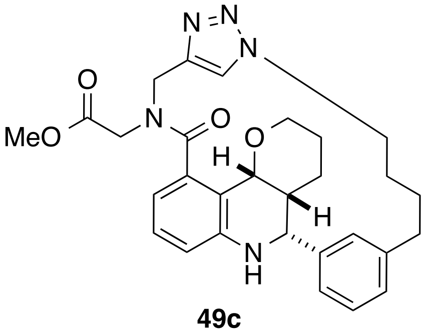

49c and

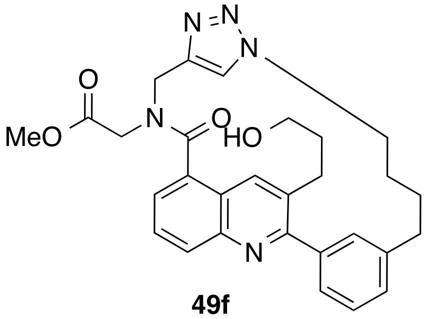

49f

25c (3.0 mg, 3.2 µmol, 1.0 eq) was reacted according to GSP-8. After preparative HPLC (20-70% B, without addition of TFA), **49c** (0.28 mg, 0.54 µmol, 17%) was obtained as a white powder. **49f** (0.21 mg, 0.41 µmol, 13%) was obtained as a side product.

49c

HPLC t_r = 11.56 min (5-100% B, without addition of TFA), peak area 61%; **IR** ν_{max} (neat)/cm^{-1} = 1744 w (C=O), 1638 m, 1047 s; **HRMS** (ESI+) *m/z* = 516.2604 $[M+H]^+$ found, $C_{29}H_{34}N_5O_4^+$ required 516.2605.

49f

HPLC t_r = 8.85 min (5-100% B, without addition of TFA), peak area 99%; **mp** 90 °C decomposition (H_2O); **IR** ν_{max} (neat)/cm^{-1} = 1744 m (C=O), 1646 m, 1403 m, 1046 s, 1178 s, 1000 s; **HRMS** (ESI+) *m/z* = 514.2429 $[M+H]^+$ found, $C_{29}H_{32}N_5O_4^+$ required 514.2449.

Note: these data are consistent with the data reported above.

Methyl (*Z*)-2-(23-(3-hydroxypropyl)-3-oxo-61*H*-4-aza-2(2,5)-quinolina-6(4,1)-triazola-1(1,3)-benzenacyclodecaphane-4-yl)acetate (49d)

and **methyl 2-((24*aS*,25*R*,210*bS*,*Z*)-26-formyl-3-oxo-23,24,24*a*,25,26,210*b*-hexahydro-22*H*,61*H*-4-aza-2(5,10)-pyrano[3,2-*c*]quinolina-6(4,1)-triazola-1(1,3)-benzenacyclodecaphane-4-yl)acetate (49f)**

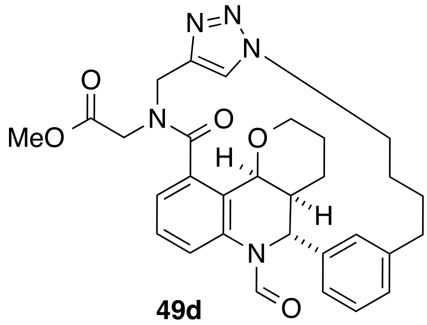

49d

and

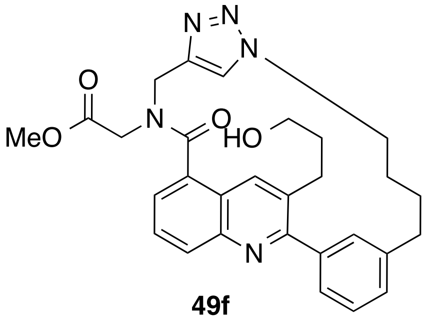

49f

25d (10.4 mg, 0.0110 mmol, 1.0 eq) was reacted according to GSP-8. After preparative HPLC (10-100% B, without addition of TFA), **49d** (0.50 mg, 0.97 µmol, 9%) and **49f** (3.0 mg, 5.52 µmol, 50%) were both obtained as white powders as side products.

49d

TLC R_f = 0.16 (EtOAc); **HPLC** t_r = 9.27 min (5-100% B, without addition of TFA), peak area 96%; **IR** ν_{max} (neat)/cm^{-1} = 2934 w, 1738 m, 1673 s, 1650 s, 1589 m, 1433 m, 1269 m, 1198 m; **^{1}H NMR** (500 MHz, DMSO-d_6, 27°C) and **^{13}C NMR** (125 MHz, DMSO-d_6, 27 °C) spectra were given in **Section 7.3**; however, due to the existence of rotamers, full assignment was not conducted; **HRMS** (ESI+) *m/z* = 544.2534 [M+H]$^+$ found, $C_{30}H_{34}N_5O_5^+$ required 544.2554.

49f

TLC R_f = 0.12 (EtOAc); **HPLC** t_r = 7.82 min (5-100% B, without addition of TFA), peak area 71%; **IR** ν_{max} (neat)/cm^{-1} = 2923 m, 1639 s, 1053 s; **HRMS** (ESI+) *m/z* = 514.2468 [M+H]$^+$ found, $C_{29}H_{32}N_5O_4^+$ required 514.2449.

Note: these data are consistent with the data reported above.

Methyl (*Z*)-2-(2-cyano-5-oxo-8^1*H*-3,6-diaza-8(4,1)-triazola-1,4(1,3)-dibenzenacyclododecaphane-6-yl)acetate (50)

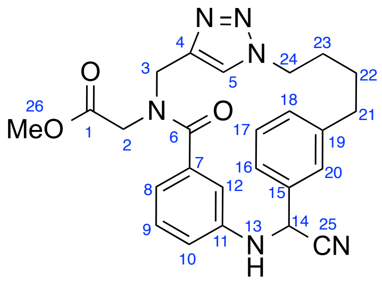

34 (24 mg, 0.027 mmol, 1.0 eq) was reacted according to GSP-8. After preparative HPLC (30-100% B, without addition of TFA), **50** (11 mg, 0.024 mmol, 89%) was obtained as a white solid.

TLC R_f = 0.25 (CH_2Cl_2/MeOH 40:1); **HPLC** t_r = 9.40 min (30-100% B), peak area 100% **IR** ν_{max} (neat)/cm^{-1} = 3308 w (N-H), 2159 w (C≡N), 1746 s (C=O), 1633 s (C=O), 1604 m (C=C), 1587 m (C=C); ^{1}**H NMR** (500 MHz, DMSO, 120 °C) δ = 7.58 (s, 1H; H-5), 7.36-7.31 (m, 1H; H-8 or H-10 or H-16 or H-18), 7.31-7.26 (m, 2H; H-12 and either H-9 or H-17), 7.20 (t, J = 8.1 Hz, 1H; H-9 or H-17), 7.14 (d, J = 7.4 Hz, 1H; H-8 or H-10 or H-16 or H-18), 6.89 (d, J = 8.0 Hz, 1H; H-8 or H-10 or H-16 or H-18), 6.83-6.76 (m, 1H; H-20), 6.69 (d, J = 7.8 Hz, 1H; H-8 or H-10 or H-16 or H-18), 6.46 (brs, 1H; H-13), 5.83 (d, J = 7.9 Hz, 1H; H-14), 4.60 (d, J = 16.0 Hz, 1H; H-2a), 4.50 (d, J = 15.7 Hz, 1H; H-2b), 4.38 (t, J = 6.5 Hz, 2H; H-24), 4.05 (s, 2H; H-3), 3.63 (s, 3H; H-26), 2.61 (t, J = 7.3 Hz, 2H; H-21), 1.83 (quint, J = 6.9 Hz, 2H; H-23), 1.49-1.36 (m, 2H; H-22); **HRMS** (ESI+) m/z = 459.2148 $[M+H]^+$ found, $C_{25}H_{27}N_6O_3^+$ required 459.2145.

Methyl (*E*)-2-(13-methylene-3,8,16-trioxo-2,7,9,15-tetraaza-1,6(1,3)-dibenzenacyclohexadecaphan-11-en-15-yl)acetate (51)

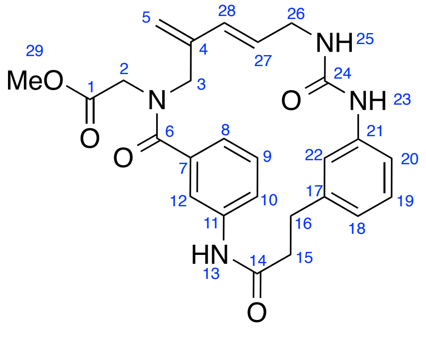

35 (22 mg, 0.024 mmol, 1.0 eq) was reacted according to GSP-8. After preparative HPLC (10-100% B), **51** (9.0 mg, 0.019 mmol, 78%) was obtained as a white powder.

TLC R_f = 0.48 (EtOAc); **HPLC** t_r = 9.20 min (5-100% B), peak area 96%; **mp** 103 °C decomposition (H_2O); **IR** ν_{max} (neat)/cm^{-1} = 1747 m (C=O), 1590 m, 1553 s, 1440 m, 1207 s; ^{1}**H NMR** (500 MHz, DMSO-d_6, 120 °C) δ = 9.33 (brs, 1H; H-13), 7.93 (brs, 1H; H-23), 7.66 (s, 1H; H-22), 7.60-7.46 (brs × 2, 2H; H-12 and H-10), 7.29 (t, J = 7.8 Hz, 1H; H-9), 7.10 (t, J = 7.6 Hz, 1H; H-19), 7.00 (d, J = 7.6 Hz, 1H; H-8), 6.92-6.76 (m, 2H; H-20 and H-18), 6.10 (d, J = 16.0 Hz, 1H; H-28), 4.10-5.90 (brs, 1H; H-25), 5.79 (brs, 1H; H-27), 5.24 (s, 1H; H-5a), 5.10 (s, 1H; H-5b), 4.22 (s, 2H; H-3), 4.03 (s, 2H; H-2), 3.73-3.63 (m, 5H, H-26 and H-29), 3.04-2.92 (m, 2H; H-16, coincides with water signal), 2.74-2.55 (m, 2H; H-15); ^{13}**C NMR** (125 MHz, DMSO-d_6, 120 °C) due to interconversion, C-2, C-3, C-5, C-10, C-12 and C-27 are missing, δ = 170.2 (C-6), 169.7 (C-14), 168.5 (C-1), 154.8 (C-24), 140.7 (C-17), 139.9 (C-21), 139.5 (C-4), 138.4 (C-11), 135.4 (C-7), 128.3 (C-28), 127.6 (C-9 or C-19), 127.5 (C-9 or C-19), 121.4 (C-18), 120.9 (C-8), 118.4 (C-22), 115.9 (C-20), 51.0 (C-29), 40.8 (C-26), 36.8 (C-15), 30.0 (C-16); **HRMS** (ESI+) m/z = 477.2136 $[M+H]^+$ found, $C_{26}H_{29}N_4O_5^+$ required 477.2132.

Methyl 2-(52,2,14-trioxo-3,15-diaza-1(1,3)-benzena-5(1,3)-cyclopentanacyclopentadecaphan-51(55)-en-3-yl)acetate (52a)

and **methyl (*Z*)-2-(55,2,14-trioxo-3,15-diaza-1(1,3)-benzena-5(1,3)-cyclopentanacyclopentadecaphan-51-en-3-yl)acetate (52b)**

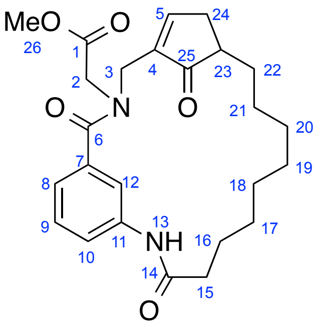

52a

and

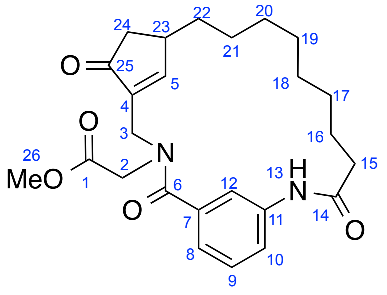

52b

The mixture **23a** and **23b** (21 mg, crude, max. 0.024 mmol, 1.0 eq) was reacted according to GSP-8. After preparative HPLC (10-100% B), **52a** (4.18 mg, 9.49 µmol, 24%, over two steps from **4**) and **52b** (2.28 mg, 5.18 µmol, 13%, over two steps from **4**) were both obtained as a white solid.

52a

TLC R_f = 0.55 (EtOAc); **HPLC** t_r = 12.53 min (30-50% B), peak area 100%; **mp** 120-124 °C (H_2O); **IR** ν_{max} (neat)/cm^{-1} = 2928 m, 1746 m (C=O), 1688 s (C=O), 1616 m, 1586 m, 1548 m, 1420 m, 1207 m, 1174 m; **^{1}H NMR** (500 MHz, DMSO-d_6, 27 °C) δ = 9.96 (s, 1H; H-13), 7.75 (d, J = 8.2 Hz, 1H; H-10), 7.66 (s, 1H; H-5), 7.48 (t, J = 1.9 Hz, 1H; H-12), 7.36 (t, J = 7.9 Hz, 1H; H-9), 7.02 (d, J = 7.7 Hz, 1H; H-8), 4.31 (d, J = 17.0 Hz, 1H; H-2a), 4.06 (d, J = 17.1 Hz, 1H; H-2b), 3.92 (s, 2H; H-3), 3.68 (s, 3H; H-26), 2.87-2.71 (m, 1H; H-24a), 2.41-2.35 (m, 2H; H-23 and H-24b), 2.30 (quint, J = 8.0, 6.9 Hz, 2H; H-15), 1.72-1.57 (m, 1H; H-16a), 1.59-1.41 (m, 2H; H-16b and H-22a), 1.43-1.10 (m, 10H; H-22b, H-21a, H-20, H-19, H-18 and H-17), 1.10-0.95 (m, 1H; H-21b); **^{13}C NMR** (125 MHz, DMSO-d_6, 27 °C) δ = 209.7 (C-25), 171.5 (C-14), 171.3 (C-6), 169.5 (C-1), 158.7 (C-5), 140.4 (C-4), 139.0 (C-11), 136.0 (C-7), 129.3 (C-9), 121.1 (C-8), 119.7 (C-10), 115.4 (C-12), 51.9 (C-26), 48.0 (C-2), 46.8 (C-3), 44.8 (C-23), 35.4 (C-15), 32.7 (C-24), 29.3 (C-22), 27.0 (C-17, C-18, C-19 or C-20), 26.9 (C-17, C-18, C-19 or C-20), 26.6 (C-17, C-18, C-19 or C-20), 25.8 (C-17, C-18, C-19 or C-20), 23.9 (C-16), 23.3 (C-21); **HRMS** (ESI+) m/z = 441.2384 $[M+H]^+$ found, $C_{25}H_{33}N_2O_5^+$ required 441.2384.

52b

TLC R_f = 0.63 (EtOAc); **HPLC** t_r = 13.02 min (30-50% B), peak area 94%; **mp** 116 °C decomposition (H_2O); **IR** ν_{max} (neat)/cm^{-1} = 2925 m, 1747 m (C=O), 1685 s (C=O), 1627 s, 1587 m, 1548 m, 1421 m, 1202 s, 1177 s; **^{1}H NMR** (500 MHz, DMSO-d_6, 90 °C) δ = 9.72 (s, 1H; H-13), 7.72 (s, 1H; H-12), 7.47 (d, J = 8.0 Hz, 1H; H-10), 7.40 (s, 1H; H-5), 7.37 (t, J = 8.0 Hz, 1H; H-9), 7.06 (d, J = 7.5 Hz, 1H; H-8), 4.28 (d, J = 17.0 Hz, 1H; H-2a), 4.10 (d, J = 17.2 Hz, 1H; H-2b), 4.05 (s, 2H; H-3), 3.69 (s, 3H; H-26), 2.83 (s, 1H; H-23), 2.53 (coincides with solvent signal; H-24a), 2.34 (t, J = 6.4 Hz, 2H; H-15), 1.93 (d, J = 18.9 Hz, 1H; H-24b), 1.68 (quint, J = 6.0 Hz, 2H; H-16), 1.49 (brs, 1H; H-22a), 1.42-1.26 (m, 11H; H-22b, H-21, H-20, H-19, H-18 and H-17); **HRMS** (ESI+) m/z = 441.2390 $[M+H]^+$ found, $C_{25}H_{33}N_2O_5^+$ required 441.2384.

Methyl (*E*)-2-(3,13-dioxo-2,4,12-triaza-1(1,3)-benzenacyclotridecaphan-9-en-12-yl)acetate (53a)

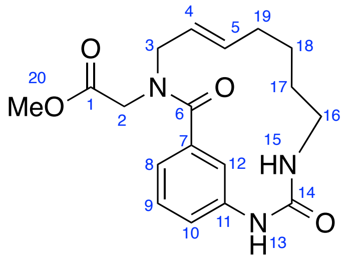

36a (48 mg, 0.062 mmol, 1.0 eq) was reacted according to GSP-8. After preparative HPLC (10-100% B), **53a** (19.4 mg, 0.0562 mmol, 91%) was obtained as a white powder.

TLC R_f = 0.24 (EtOAc); **HPLC** t_r = 7.95 min (5-100% B), peak area 99%; **mp** 206-210 °C (H_2O); **IR** ν_{max} (neat)/cm^{-1} = 3356 m, 2950 w, 1751 m (C=O), 1672 s (C=O), 1631 s, 1601 m, 1586 m, 1541 m, 1456 m, 1415 m, 1295 m, 1212 s, 1171 s; ^{1}**H NMR** (500 MHz, DMSO-d_6, 27 °C) δ = 8.33 (s, 1H; H-13), 7.42 (s, 1H; H-12), 7.34 (t, J = 7.8 Hz, 1H; H-9), 7.06 (d, J = 7.3 Hz, 1H; H-10), 7.03 (d, J = 7.5 Hz, 1H; H-8), 6.36 (t, J = 5.9 Hz, 1H; H-15), 5.66 (dt, J = 14.9, 6.8 Hz, 1H; H-5), 5.48 (dt, J = 15.7, 4.1 Hz, 1H; H-4), 4.08 (s, 2H; H-2), 3.84 (s, 2H; H-3), 3.67 (s, 3H; H-20), 3.06 (appq, J = 5.3 Hz, 2H; H-16), 2.01 (t, J = 8.3 Hz, 2H; H-19), 1.57-1.48 (m, 2H; H-17), 1.49-1.31 (m, 2H; H-18); ^{13}**C NMR** (125 MHz, DMSO-d_6, 27 °C) δ = 171.3 (C-6), 169.5 (C-1), 155.9 (C-14), 139.5 (C-11), 135.8 (C-7), 130.9 (C-5), 129.5 (C-9), 126.2 (C-4), 122.3 (C-10), 121.3 (C-8), 117.6 (C-12), 51.8 (C-20), 51.3 (C-3), 47.5 (C-2), 39.6 (C-16, coincides with solvent signal), 30.5 (C-19), 27.0 (C-17), 25.3 (C-18); **HRMS** (ESI+) m/z = 346.1753 [M+H]$^+$ found, $C_{18}H_{24}N_3O_4^+$ required 346.1761. The geometry of the alkene is as drawn.

Dimethyl 2,2'-(3,13,16,26-tetraoxo-2,4,12,15,17,25-hexaaza-1,14(1,3)-dibenzenacyclohexacosaphane-9,22-diene-12,25-diyl)diacetate (53b)

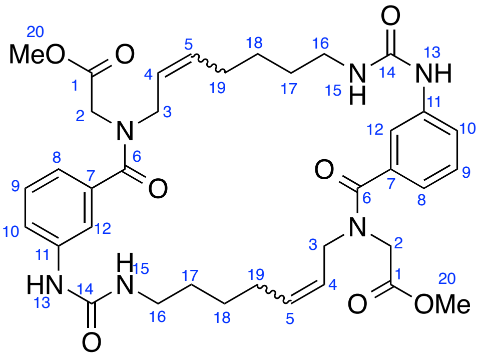

36b (10 mg, 6.4 µmol, 1.0 eq) was reacted according to GSP-8. After preparative HPLC (10-100% B), **53b** (3.8 mg, 5.5 µmol, 86%) was obtained as a white powder.

TLC R_f = 0.38 (EtOAc); **HPLC** t_r = 10.42 min (5-100% B), peak area 99%; **mp** 124 °C (H_2O); **IR** ν_{max} (neat)/cm^{-1} = 3345 m, 2933 w, 1744 m (C=O), 1588 s, 1559 s, 1463 m, 1426 m, 1212 s; ^{1}**H NMR** (500 MHz, DMSO-d_6, 120 °C) major rotamer signals only, δ = 8.21 (s, 2H; H-13 × 2), 7.65 (s, 2H; H-12 × 2), 7.34-7.17 (m, 4H; H-9 × 2 and H-10 × 2), 6.89 (dt, J = 6.8, 1.8 Hz, 2H; H-8 × 2), 5.91 (brs; H-15 × 2), 5.67-5.38 (m, 4H; H-5 × 2 and H-4 × 2), 4.09 (s, 4H; H-2 × 2), 3.88 (d, J = 5.8 Hz, 4H; H-

3 × 2), 3.68 (s, 6H; H-20 × 2), 3.07 (H-16 × 2, coincides with water signal), 2.06 (q, J = 6.8 Hz, 4H; H-19 × 2), 1.55-1.38 (m, 8H; H-17 × 2 and H-18 × 2); **^{13}C NMR** (125 MHz, DMSO-d_6, 27 °C) major rotamer signals only, δ = 171.1 (C-6 × 2), 169.5 (C-1 × 2), 155.0 (C-14 × 2), 140.3 (C-11 × 2), 136.0 (C-7 × 2), 134.0 (C-5 × 2), 129.0 (C-9 × 2), 124.5 (C-4 × 2), 119.4 (C-8 × 2), 118.4 (C-10 × 2), 115.2 (C-12 × 2), 51.9 (C-3 × 2), 51.8 (C-20 × 2), 46.4 (C-2 × 2), 39.0 (C-16 × 2, coincides with solvent signal), 31.2 (C-19 × 2), 29.3 (C-17 × 2), 25.4 (C-18 × 2); **HRMS** (ESI+) m/z = 691.3484 $[M+H]^+$ found, $C_{36}H_{46}N_6O_8^+$ required 691.3450. The geometry of the alkene is as drawn.

Macrocycle 54a

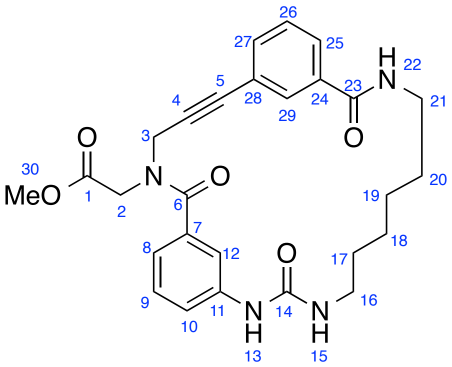

37 (22 mg, 0.024 mmol, 1.0 eq) was reacted according to GSP-8. After preparative HPLC (10-100% B), **54a** (10 mg, 0.011 mmol, 85%) was obtained as a white powder.

TLC R_f = 0.53 (EtOAc); **HPLC** t_r = 10.21 min (5-100% B), peak area 99%; **mp** 100 °C decomposition (H_2O); **IR** ν_{max} (neat)/cm^{-1} = 1645 s, 1549 s, 1461 m, 1203 s, 1132 s; **^{1}H NMR** (500 MHz, DMSO-d_6, 120 °C) δ =8.25 (s, 1H; H-13), 8.06 (t, J = 1.7 Hz, 1H; H-12), 8.02 (s, 1H; H-22), 7.94 (s, 1H; H-29), 7.75 (d, J = 7.8 Hz, 1H; H-25), 7.55 (d, J = 7.6 Hz, 1H; H-27), 7.44 (t, J = 7.7 Hz, 1H; H-26), 7.32 (t, J = 7.8 Hz, 1H; H-9), 7.19 (dd, J = 8.1, 2.4 Hz, 1H; H-10), 6.96 (d, J = 7.5 Hz, 1H; H-8), 5.91 (brs, 1H; H-15), 4.38 (s, 2H; H-3), 4.30 (s, 2H; H-2), 3.71 (s, 3H; H-30), 3.35 (appq, J = 5.8 Hz, 2H; H-21), 3.11 (H-16, coincides with water signal), 1.59 (appdt, J = 12.0, 6.8 Hz, 2H; H-20), 1.52 (quint, J = 6.5 Hz, 2H; H-17), 1.49-1.35 (m, 4H; H-18 and H-19); **^{13}C NMR** (125 MHz, DMSO-d_6, 27 °C) δ = 170.6 (C-6), 169.2 (C-1), 166.2 (C-23), 154.9 (C-14), 140.5 (C-11), 136.0 (C-24), 135.0 (C-7), 134.0 (C-27), 130.6 (C-29), 129.3 (C-9), 128.6 (C-26), 127.8 (C-25), 122.0 (C-28), 120.1 (C-8), 119.0 (C-10), 114.8 (C-12), 84.9 (C-4), 83.6 (C-5), 51.9 (C-30), 46.7 (C-2), 40.9 (C-3), 38.3 (C-21), 37.6 (C-16), 28.0 (C-17), 27.7 (C-20), 24.4 (C-18 or C-19), 24.2 (C-19 or C-18); **HRMS** (ESI+) m/z = 491.2273 $[M+H]^+$ found, $C_{27}H_{31}N_4O_5^+$ required 491.2289.

Macrocycle 54b

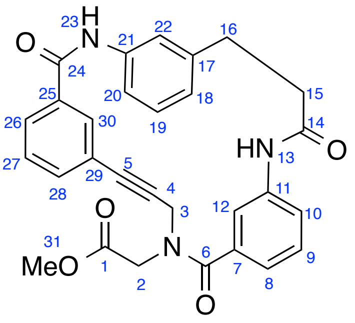

38 (37 mg, 0.040 mmol, 1.0 eq) was reacted according to GSP-8 and a white precipitate formed. The precipitate collected by vacuum filtration. The filtrate was purified by preparative HPLC (10-100% B). After combining precipitates and HPLC fraction, **54b** (13 mg, 0.027 mmol, 67%) was obtained as a white solid.

TLC R_f = 0.78 (EtOAc); **HPLC** t_r = 11.27 min (5-100% B), peak area 100%; **mp** 280 °C decomposition (H_2O); **IR** ν_{max} (neat)/cm^{-1} = 1747 s (C=O), 1678 s (C=O), 1634 s, 1616 m, 1583 m, 1545 m, 1452 m, 1418 s, 1330 m, 1288 m, 1208 s; **^{1}H NMR** (500 MHz, DMSO-d_6, 120 °C) δ = 10.16 (s, 1H; H-23), 9.77 (s, 1H; H-13), 8.02 (d, J = 7.8 Hz, 1H; H-20), 7.97 (t, J = 1.8 Hz, 1H; H-12), 7.81 (s, 1H; H-30), 7.78 (dt, J = 7.3, 1.7 Hz, 1H; H-26), 7.58-7.43 (m, 3H; H-28, H-27 and H-10), 7.37 (t, J = 7.8 Hz, 1H; H-9), 7.32 (s, 1H; H-22), 7.23 (t, J = 7.8 Hz, 1H; H-19), 7.08 (d, J = 7.5 Hz, 1H; H-8), 6.95 (d, J = 7.5 Hz, 1H; H-18), 4.53 (s, 2H; H-3), 4.25 (s, 2H; H-2), 3.67 (s, 3H; H-31), 2.99 (H-16, coincides with water signal), 2.71-2.60 (m, 2H; H-15); **HRMS** (ESI+) m/z = 496.1855 [M+H]$^+$ found, $C_{29}H_{26}N_3O_5^+$ required 496.1867.

Macrocycle 55

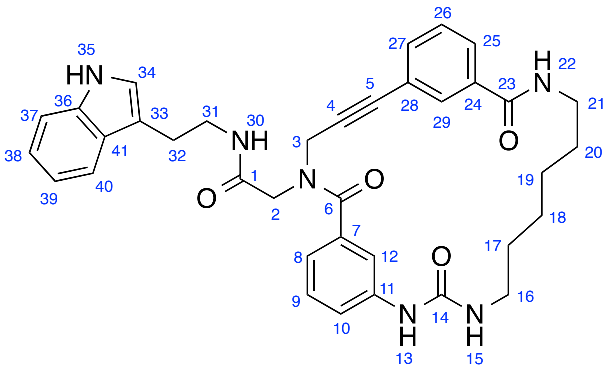

37 (7.86 mg, 8.52 µmol, 1.0 eq), tryptamine (4.8 mg, 0.030 mmol, 3.0 eq), Zr(O*t*Bu)$_4$ (12 µL, 0.030 mmol, 3.0 eq) and HOAt (4.1 mg, 0.030 mmol, 3.0 eq) were dissolved in THF (6.7 mM). The reaction mixture was stirred at 100 °C overnight in a sealed tube. The solvent was removed under a stream of nitrogen and the residue purified using preparative HPLC. After preparative HPLC (30-100% B), **55** (3.71 mg, 6.00 µmol, 70%) was obtained as an off-white solid.

TLC R_f = 0.17 (EtOAc); **HPLC** t_r = 8.09 min (30-100% B), peak area 92%; **mp** 129 °C decomposition (H_2O); **IR** ν_{max} (neat)/cm^{-1} = 3307 brw, 2925 w, 1638 s (C=O), 1551 s, 1457 m, 1229 m, 740 m; **^{1}H NMR** (500 MHz, DMSO-d_6, 120 °C) δ = 10.42 (s, 1H; H-35), 8.22 (s, 1H; H-13), 8.04 (s, 1H; H-12), 8.00 (s, 1H; H-22), 7.93 (s, 1H; H-29), 7.75 (d, J = 7.8 Hz, 1H; H-25), 7.60 (s, 1H; H-30), 7.56-7.52 (m, 2H; H-40 and H-27), 7.43 (t, J = 7.7 Hz, 1H; H-26), 7.35 (d, J = 8.1 Hz, 1H; H-37), 7.29 (t, J = 7.8 Hz, 1H; H-9), 7.14 (d, J = 8.1 Hz, 1H; H-10), 7.11 (s, 1H; H-34), 7.06 (t, J = 7.5 Hz, 1H; H-38), 7.02-6.93 (m, 2H; H-39 and H-8), 5.93 (s, 1H; H-15), 4.33 (s, 2H; H-3), 4.13 (s, 2H; H-2), 3.45 (q, J = 6.8 Hz, 2H; H-31), 3.34 (q, J = 5.8 Hz, 2H; H-21), 3.10 (t, J = 7.0 Hz, 2H; H-16, coincides with water signal), 2.90 (t, J = 7.3 Hz, 2H; H-32, coincides with water signal), 1.59 (quint, J = 6.5 Hz, 2H; H-20), 1.52 (quint, J = 6.6 Hz, 2H; H-17), 1.49-1.37 (m, 4H; H-19 and H-18); **^{13}C NMR** (125 MHz, DMSO-d_6, 27 °C) δ = 170.5 (C-6), 167.3 (C-1), 166.2 (C-23), 155.0 (C-14), 140.4 (C-11), 136.2 (C-36), 136.0 (C-24), 135.5 (C-7), 134.0 (C-27), 130.6 (C-29), 129.2 (C-9), 128.5 (C-26), 127.8 (C-25), 127.2 (C-41), 122.7 (C-34), 121.9 (C-28), 120.9 (C-38), 120.3 (C-8), 118.6 (C-10), 118.2 (C-40 and C-39), 114.7 (C-12), 111.7 (C-33), 111.4 (C-37), 85.0 (C-4), 83.5 (C-5), 47.0 (C-2), 40.8 (C-3), 39.6 (C-31, coincides with solvent signal), 38.3 (C-21), 37.5 (C-16), 28.0 (C-17), 27.7 (C-20), 25.1 (C-32), 24.3 (C-18 or C-19), 24.2 (C-19 or C-18); **HRMS** (ESI+) m/z = 619.3040 [M+H]$^+$ found, $C_{36}H_{39}N_6O_4^+$ required 619.3027.

2-(52,2,14-Trioxo-3,15-diaza-1(1,3)-benzena-5(1,3)-cyclopentanacyclopentadecaphan-51(55)-en-3-yl)acetic acid (56a)

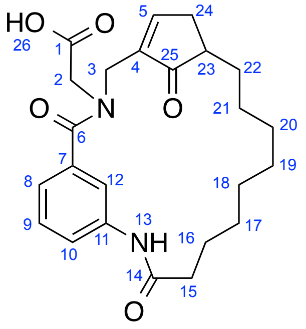

23a (1.84 mg, 2.11 mmol, 1.0 eq) was reacted according to GSP-9. After preparative HPLC (10-60% B), **56a** (0.66 mg, 1.5 mmol, 71%) was obtained as a white solid.

HPLC t_r = 13.74 min (10-60% B), peak area 98%; **mp** 123 °C decomposition (H_2O); **IR** ν_{max} (neat)/cm^{-1} = 2930 m, 1666 m (C=O), 1626 m, 1463 m, 1424 m, 1152 s; **^{1}H NMR** (500 MHz, DMSO-d_6, 27 °C) major rotamer signals only, δ = 9.95 (s, 1H; H-13), 7.76 (dt, J = 8.2, 1.5 Hz, 1H; H-10), 7.66 (t, J = 2.2 Hz, 1H; H-5), 7.46 (t, J = 1.8 Hz, 1H; H-12), 7.36 (t, J = 7.9 Hz, 1H; H-9), 7.01 (dt, J = 7.6, 1.3 Hz, 1H; H-8), 4.21 (d, J = 17.1 Hz, 1H; H-2a), 3.95 (d, J = 17.1 Hz, 1H; H-2b), 3.90 (s, 2H; H-3), 2.77 (ddd, J = 19.1, 6.8, 3.0 Hz, 1H; H-24a), 2.43-2.34 (m, 2H; H-23 and H-24b), 2.36-2.22 (m, 2H; H-15), 1.69-1.60 (m, 1H; H-16a), 1.60-1.42 (m, 2H; H-16b and H-22a), 1.40-1.12 (m, 10H; H-22b, H-21a, H-20, H-19, H-18 and H-17), 1.10-0.96 (m, 1H; H-21b); **^{13}C NMR** (125 MHz, DMSO-d_6, 27 °C) major rotamer signals only, δ = 209.7 (C-25), 171.5 (C-14), 171.2 (C-6), 170.4 (C-1), 158.7 (C-5), 140.5 (C-4), 139.0 (C-11), 136.2 (C-7), 129.2 (C-9), 121.1 (C-8), 119.6 (C-10), 115.4 (C-12), 48.0 (C-2), 46.7 (C-3), 44.8 (C-23), 35.4 (C-15), 32.7 (C-24), 29.3 (C-22), 27.0 (C-17, C-18, C-19 or C-20), 26.9 (C-17, C-18, C-19 or C-20), 26.7 (C-17, C-18, C-19 or C-20), 25.8 (C-17, C-18, C-19 or C-20), 23.9 (C-16), 23.2 (C-21); **HRMS** (ESI+) m/z = 427.2210 [M+H]$^+$ found, $C_{24}H_{31}N_2O_5^+$ required 427.2227.

(*Z*)-2-(55,2,14-Trioxo-3,15-diaza-1(1,3)-benzena-5(1,3)-cyclopentanacyclopentadecaphan-51-en-3-yl)acetic acid (56b)

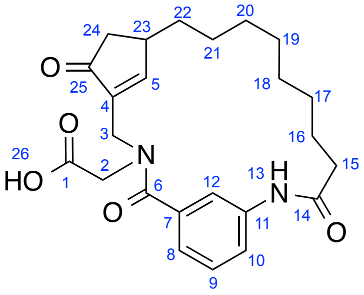

23b (6.5 mg, 7.4 µmol, 1.0 eq) was reacted according to GSP-9. After preparative HPLC (10-60% B), **56b** (3.0 mg, 7.0 µmol, 94%) was obtained as a colourless gum.

HPLC t_r = 14.06 min (10-60% B), peak area 92%; **IR** ν_{max} (neat)/cm^{-1} = 1623 m (C=O), 1548 m, 1466 m, 1154 s; **^{1}H NMR** (500 MHz, DMSO-d_6, 90 °C) major rotamer signals only, δ = 9.72 (s, 1H; H-13), 7.71 (s, 1H; H-12), 7.45 (apps, 1H; H-10), 7.40 (s, 1H; H-5), 7.36 (t, J = 7.8 Hz, 1H; H-9), 7.05 (d, J = 7.5 Hz, 1H; H-8), 4.19 (d, J = 17.6 Hz, 1H; H-2a), 4.03 (s, 2H; H-3), 3.99 (d, J = 17.3 Hz,

1H; H-2b), 2.83 (s, 1H; H-23), 2.60-2.52 (m, H-24a, coincides with solvent signal), 2.41-2.28 (m, 2H; H-15), 1.94 (dd, J = 18.7, 2.2 Hz, 1H; H-24b), 1.75-1.62 (m, 2H; H-16), 1.49 (s, 1H; H-22a), 1.45-1.22 (m, 11H; H-22b, H-17, H-18, H-19, H-20 and H-21); **^{13}C NMR** (125 MHz, DMSO-d_6, 27 °C) major rotamer signals only, δ = 207.6 (C-25), 171.6 (C-14), 170.8 (C-6), 170.3 (C-1), 164.2 (C-5), 140.3 (C-4), 139.1 (C-11), 136.3 (C-7), 129.1 (C-9), 121.1 (C-8), 120.1 (C-10), 116.9 (C-12), 47.9 (C-2), 44.9 (C-3), 41.3 (C-24), 38.3 (C-23), 35.5 (C-15), 33.6 (C-22), 27.2 (C-17, C-18, C-19, C-20 or C-21), 26.8 × 2 (two carbons from C-17, C-18, C-19, C-20, and C-21), 26.3 (C-17, C-18, C-19, C-20 or C-21), 25.2 (C-17, C-18, C-19, C-20, C-21), 23.9 (C-16); **HRMS** (ESI+) m/z = 427.2213 $[M+H]^+$ found, $C_{24}H_{31}N_2O_5^+$ required 427.2227.

(*Z*)-2-(6-(2-(cyclohexylamino)-2-oxo-1-phenylethyl)-4,7-dioxo-1^1*H*-3,6-diaza-1(4,1)-triazola-5(1,3)-benzenacyclotetradecaphane-3-yl)acetic acid (57)

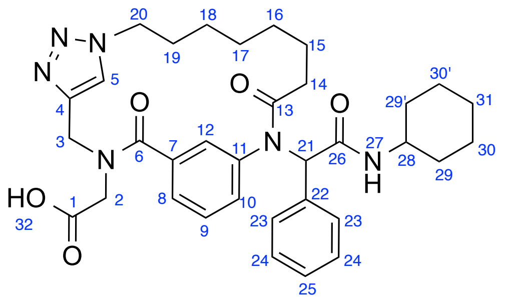

28a (13 mg, 0.012 mmol, 1.0 eq) was reacted according to GSP-9. After purification by preparative HPLC (30-100% B), title compound was isolated (6.6 mg, 0.011 mmol, 88%) as an off-white solid.

TLC R_f = 0.39 (CH_2Cl_2/MeOH 15:1); **HPLC** t_r = 7.55 min (30-100% B), peak area 100%; **IR** ν_{max} (neat)/cm^{-1} = 3272 w (N-H), 1727 m (C=O), 1638 m (C=O); **^{1}H NMR** (500 MHz, DMSO-d$_6$, 120 °C) δ = 7.91 (s, 1H; H-5), 7.38-7.31 (d, J = 4.5 Hz, 1H; H-27), 7.31-7.25 (brs, 1H; H-12), 7.25-7.11 (m, 6H; H-8, H-9, H-10, H-24, H-25), 7.11-7.07 (m, 2H; H-23), 6.09 (s, 1H; H-21), 4.61 (d, J = 15.8 Hz, 1H; H-3a), 4.54 (d, J = 15.9 Hz, 1H; H-3b), 4.39 (t, J = 6.1 Hz, 2H; H-20), 4.01 (d, J = 17.1 Hz, 1H; H-2a), 3.90 (d, J = 17.6 Hz, 1H; H-2b), 3.65 (coincides with water signal; H-28), 1.89 (t, J = 6.9 Hz, 2H; H-14), 1.86-1.75 (m, 3H; H-19 and either H-29a or H-29'a), 1.75-1.67 (m, 2H; either H-29a or H-29'a, and either H-30a or H-30'a), 1.66-1.59 (m, 1H; either H-30a or H-30'a), 1.59-1.51 (m, 1H; H-31a), 1.42-1.34 (m, 2H; H-15), 1.34-1.23 (m, 3H; H-30b, H-30'b, and either H-29b or H-29'b), 1.22-1.11 (m, 2H; H-31b, and either H-29b or H-29'b), 1.21-0.97 (m, 6H; H-16, H-17, H-18); **^{13}C NMR** (125 MHz, DMSO-d_6, 120 °C) δ = 172.4 (C-13), 170.1 (C-1 or C-6), 170.1 (C-1 or C-6), 169.0 (C-26), 143.8 (C-4), 140.7 (C-11 or C-22), 136.1 (C-11 or C-22), 132.1 (C-8 or C-10), 130.3 (C-23), 129.5

(C-12), 128.9 (C-8 or C-10), 128.2 (C-24), 127.9 (C-25), 126.3 (C-9), 123.3 (C-5), 64.5 (C-21), 49.8 (C-20), 48.6 (C-28), 34.2 (C-14), 32.5 (C-29 or C-29'), 32.4 (C-29 or C-29'), 28.9 (C-19), 28.5 (C-16), 27.6 (C-17), 25.8 (C-18), 25.6 (C-31), 25.0 (C-15), 24.7 × 2 (C-30 and C-30'); **HRMS** (ESI+) *m/z* = 615.3278 $[M+H]^+$ found, $C_{34}H_{42}N_6O_5^+$ required 615.3295.

(22*S,23*R**,*Z*)-5-(2-Hydroxyethyl)-23-methoxy-71*H*-5-aza-7(4,1)-triazola-2(2,1)-azetidina-1,3(1,3)-dibenzenacycloundecaphane-24,4-dione (58)**

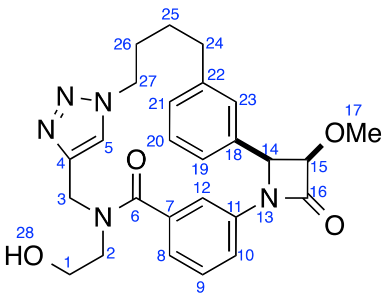

30a (36.5 mg, 0.0390 mmol, 1.0 eq) was dissolved in THF (2 mL). $LiBH_4$ (0.5 M; 282 µL, 0.141 mmol, 3.6 eq) was added in three portions. The suspension was stirred at rt for 8 h. The reaction was quenched by the addition of HCl in dioxane (4.0 M; 39 µL, 0.16 mmol, 4.0 eq). The solvent was removed under a stream of nitrogen and the residue dissolved in CH_2Cl_2 and water. The organic phase was washed with 10% aq. HCl, sat. aq. $NaHCO_3$ and brine, dried over $MgSO_4$, filtered and concentrated under reduced pressure. After preparative HPLC (15-65% B), **58** (2.7 mg, 5.7 µmol, 15%) was obtained as a white solid.

TLC R_f = 0.067 (EtOAc); **HPLC** t_r = 8.51 min (5-100% B), peak area 99%; **mp** 220 °C decomposition (H_2O); $[\alpha]_D^{25.4}$ = +5 (*c* = 0.033 in MeOH); **IR** ν_{max} (neat)/cm^{-1} = 3396 brs (OH), 2932 w, 1748 s (C=O), 1679 m, 1623 m, 1603 m, 1450 m, 1372 m, 1200 s, 1121 s; **^{1}H NMR** (500 MHz, DMSO-d_6, 120 °C) δ = 7.89 (d, J = 8.2 Hz, 1H; H-10), 7.54 (s, 1H; H-5), 7.41 (t, J = 7.9 Hz, 1H; H-9), 7.26-7.19 (m, 2H; H-23 and H-20), 7.15-7.09 (m, 3H; H-19, H-21 and H-8), 6.49 (s, 1H; H-12), 5.30 (d, J = 5.0 Hz, 1H; H-14), 4.96 (d, J = 5.0 Hz, 1H; H-15), 4.66 (d, J = 15.3 Hz, 1H; H-3a), 4.59 (d, J = 15.3 Hz, 1H; H-3b), 4.46-4.30 (m, 2H; H-27), 3.53-3.39 (m, 2H; H-1), 3.27-3.14 (m, 2H; H-2), 3.12 (s, 3H; H-17), 2.61 (t, J = 7.3 Hz, 2H; H-24), 1.91-1.69 (m, 2H; H-26), 1.47-1.38 (m, 1H; H-25a), 1.38-1.31 (m, 1H; H-25b); **^{13}C NMR** (125 MHz, DMSO-d_6, 27 °C) major rotamer signals only, due to interconversion, C-3 is missing, δ = 169.6 (C-6), 164.4 (C-16), 143.8 (C-4), 142.6 (C-22), 137.3 (C-7), 136.7 (C-11), 133.5 (C-18), 129.8 × 2 (C-9 and C-23), 128.6 (C-21), 128.2 (C-20), 124.6 (C-19), 123.7 (C-5), 122.2 (C-8), 118.6 (C-10), 112.9 (C-12), 84.0 (C-15), 60.7 (C-14), 58.1 (C-1), 57.7 (C-

17), 50.8 (C-2), 49.1 (C-27), 34.4 (C-24), 29.4 (C-26), 29.1 (C-25); **HRMS** (ESI+) *m/z* = 476.2305 $[M+H]^+$ found, $C_{26}H_{30}N_5O_4^+$ required 476.2292.

(22*R,23*R**,*Z*)-5-(2-Hydroxyethyl)-23-methoxy-71*H*-5-aza-7(4,1)-triazola-2(2,1)-azetidina-1,3(1,3)-dibenzenacycloundecaphane-24,4-dione (59a)**

and **2-((22*R**,23*R**,*Z*)-23-methoxy-24,4-dioxo-71*H*-5-aza-7(4,1)-triazola-2(2,1)-azetidina-1,3(1,3)-dibenzenacycloundecaphane-5-yl)acetic acid (59b)**

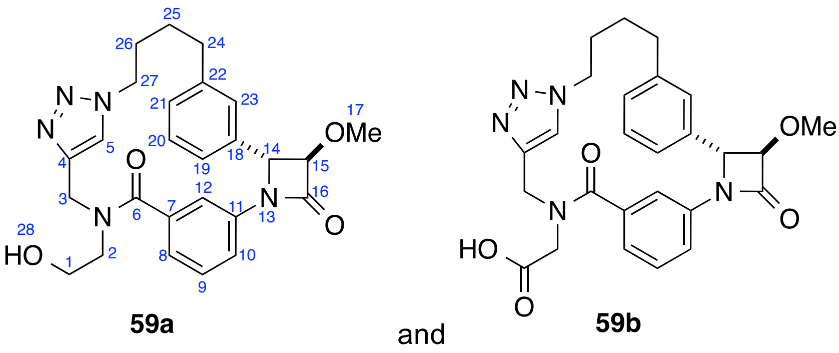

59a and **59b**

31a (47 mg, 0.050 mmol, 1.0 eq) was dissolved in THF (2 mL). $LiBH_4$ (0.5 M; 0.36 mL, 0.18 mmol, 3.6 eq) was added in three portions. The suspension stirred at rt for 8 h. The reaction was quenched by the addition of HCl in dioxane (4M; 50 µL, 0.20 mmol, 4.0 eq). The solvent was removed under a stream of nitrogen and the residue partitioned between EtOAc and water. The organic phase was washed with 10% aq. HCl, sat. aq. $NaHCO_3$ and brine, dried over $MgSO_4$, filtered and concentrated under reduced pressure. After preparative HPLC (15-65% B), **59a** (2.9 mg, 6.1 µmol, 12%) was obtained as a white solid. **59b** (3.0 mg, 6.3 µmol, 12%) was obtained as a white solid as a side product.

59a

TLC R_f = 0.10 (EtOAc); **HPLC** t_r = 9.72 min (15-65% B), peak area 96%; **mp** 95 °C decomposition (H_2O); $[\alpha]_D^{25.4}$ = +8 (*c* = 0.029 in MeOH); **IR** ν_{max} (neat)/cm^{-1} = 3388 brs (OH), 2933 w, 1751 m (C=O), 1673 m, 1603 m, 1450 m, 1373 m, 1199 m, 1127 s; ^{1}**H NMR** (500 MHz, DMSO-d_6, 120 °C) δ = 7.89 (d, *J* = 8.0 Hz, 1H; H-10), 7.55 (s, 1H; H-5), 7.41 (td, *J* = 7.9, 1.8 Hz, 1H; H-9), 7.31 (s, 1H; H-23), 7.25 (td, *J* = 7.5, 1.7 Hz, 1H; H-20), 7.12 (t, *J* = 7.3 Hz, 3H; H-8, H-19 and H-21), 6.50 (s, 1H; H-12), 5.03 (t, *J* = 1.9 Hz, 1H; H-14), 4.66 (d, *J* = 15.6 Hz, 1H; H-3a), 4.59 (d, *J* = 15.2 Hz, 1H; H-3b), 4.59 (d, *J* = 2.0 Hz, 1H; H-15), 4.48-4.29 (m, 2H; H-27), 3.49 (d, *J* = 1.7 Hz, 3H; H-17), 3.48-3.36 (m, 2H; H-1 or H-2), 3.25-3.10 (m, coincides with water signal; H-2 or H-1), 2.61 (t, *J* = 7.3 Hz; H-24,

coincides with water signal), 1.94-1.72 (m, 2H; H-26), 1.49-1.37 (m, 1H; H-25a), 1.37-1.31 (m, 1H; H-25b); **^{13}C NMR** (125 MHz, DMSO-d_6, 27 °C) major rotamer signals only, δ = 169.6 (C-6), 164.2 (C-16), 143.8 (C-4), 142.9 (C-22), 137.3 (C-7 or C-11), 136.4 (C-11 or C-7), 136.2 (C-18), 129.7 (C-9), 129.2 (C-20), 128.9 and 128.8 (C-21 and C-23), 123.9 (C-5), 122.4 and 122.3 (C-8 and C-19), 119.1 (C-10), 112.9 (C-12), 90.1 (C-15), 61.7 (C-14), 58.2 (C-1), 57.2 (C-17), 50.9 (C-2), 49.1 (C-27), 34.3 (C-24), 29.4 (C-26), 29.0 (C-25); **HRMS** (ESI+) *m/z* = 476.2270 $[M+H]^+$ found, $C_{26}H_{30}N_5O_4^+$ required 476.2292.

59b

HPLC t_r = 8.71 min (20-45% B), peak area 96%; **mp** 155 °C decomposition (H_2O); **IR** ν_{max} (neat)/cm^{-1} = 3394 brs (OH), 2933 w, 1755 m (C=O), 1673 m, 1603 m, 1463 m, 1375 m, 1200 s, 1129 s; **NMR** Appropriate NMR data could not be obtained due to the existence of rotamers at a temperature range of 25-120 °C and partial decomposition at high temperature range 90-120 °C; **HRMS** (ESI+) *m/z* = 490.2061 $[M+H]^+$ found, $C_{26}H_{28}N_5O_5^+$ required 490.2085.

(4-Azidobutyl)benzene (60)

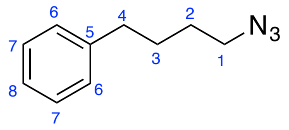

4-phenylbutan-1-ol (0.75 mL, 5.0 mmol, 1.0 eq) was reacted according to GSP-2. After Combi*Flash* (petroleum ether/EtOAc 10:1 → 1:10 over 15 min), **60** (1.97 g, 7.97 mmol, 55%) was obtained as a colourless oil.

TLC R_f = 0.90 (petroleum ether/EtOAc 6:1); **IR** ν_{max} (neat)/cm^{-1} = 2940 w, 2089 s (N_3), 1496 w, 1453 m, 1263 m; **^{1}H NMR** (400 MHz, $CDCl_3$, 27 °C) δ = 7.30 (t, *J* = 7.5 Hz, 2H; H-7), 7.24-7.16 (m, 3H; H-6 and H-8), 3.29 (t, *J* = 6.8 Hz, 2H; H-1), 2.66 (t, *J* = 7.5 Hz, 2H; H-4), 1.76-1.69 (m, 2H; H-3), 1.68-1.61 (m, 2H; H-2); **^{13}C NMR** (100 MHz, $CDCl_3$, 27 °C) δ = 142.0 (C-5), 128.5 (C-6 × 2 and C-7 × 2), 126.0 (C-8), 51.5 (C-1), 35.5 (C-4), 28.6 (C-2 and C-3);

Methyl 2-(5,8,17-trioxo-21-(4-phenylbutyl)-21*H*-4,7,9,16-tetraaza-2(4,5)-triazola-1,6(1,3)-dibenzenacycloheptadecaphane-4-yl)acetate (61)

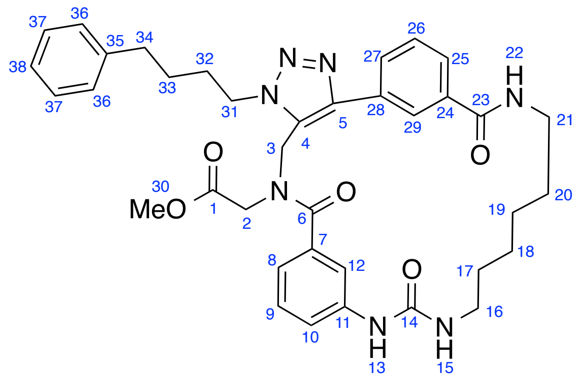

54a (7.4 mg, 0.015 mmol, 1.0 eq) was dissolved in THF (3 mL), $[Cp^*RuCl]_4$ (8.2 mg, 0.0075 mmol, 0.5 eq) and (4-azidobutyl)benzene (4.0 mg, 0.023 mmol) were added. The mixture was heated for 22 hrs at 120 °C in the microwave. The crude mixture was filtered through a pad of cotton. After preparative HPLC (30-100% B), **61** (2.6 mg, 0.0039 mmol, 26%) was obtained as a white solid.

TLC R_f = 0.49 (EtOAc); **HPLC** t_r = 8.97 min (30-100% B), peak area 96%; **mp** 109 °C decomposition (H_2O); **IR** ν_{max} (neat)/cm^{-1} = 1744 w, 1637 m, 1551 m, 1439 m, 1203 s, 1136 s; **^{1}H NMR** (500 MHz, DMSO-d_6, 120 °C) δ = 8.06 (s, 1H; H-13), 7.95 (s, 1H; H-22), 7.94 (s, 1H; H-29), 7.85 (d, J = 7.8 Hz, 1H; H-25 or H-27), 7.73 (d, J = 7.7 Hz, 1H; H-27 or H-25), 7.51 (t, J = 7.7 Hz, 1H; H-26), 7.31 (s, 1H; H-12), 7.28 (t, J = 7.7 Hz, 2H; H-37 × 2), 7.24 (t, J = 7.8 Hz, 1H; H-9), 7.22-7.14 (m, 3H; H-36 × 2 and H-38), 7.12 (d, J = 7.5 Hz, 1H; H-10), 6.87 (d, J = 7.5 Hz, 1H; H-8), 5.95 (brs, 1H; H-15), 4.95 (s, 2H; H-3), 4.36 (t, J = 7.2 Hz, 2H; H-31), 3.80 (s, 2H; H-2), 3.51 (s, 3H; H-30), 3.35 (q, J = 6.2 Hz, 2H; H-21), 3.15 (t, J = 6.5 Hz, 2H; H-16), 2.65 (t, J = 7.6 Hz, 2H; H-34), 1.89 (quint, J = 7.3 Hz, 2H; H-32), 1.69 (quint, J = 7.7 Hz, 2H; H-33), 1.63 (quint, J = 6.8 Hz, 2H; H-20), 1.53 (quint, J = 6.5 Hz, 2H; H-17), 1.49-1.44 (m, 2H; H-19), 1.45-1.38 (m, 2H; H-18); **^{13}C NMR** (125 MHz, DMSO-d_6, 120 °C) due to interconversion, C-2 and C-3 are missing, δ = 170.7 (C-6), 168.3 (C-1), 165.8 (C-23), 154.7 (C-14), 141.0 (C-35), 139.8 (C-11), 135.2 (C-24 or C-28), 134.6 (C-7), 130.5 (C-24 or C-28), 129.6 (C-27 or C-25), 128.2 (C-4), 128.0 (C-9), 127.7 (C-26), 127.5 × 2 (C-37 × 2 and C-36 × 2), 126.4 (C-25 or C-27), 125.8 (C-29), 125.0 (C-38), 119.2 (C-10), 118.8 (C-8), 116.1 (C-12), 113.9 (C-5), 51.1 (C-30), 47.3 (C-31), 38.4 (C-21), 37.8 (C-16), 33.9 (C-34), 28.3 (C-17), 28.2 (C-32), 27.9 (C-20), 27.0 (C-33), 24.5 (C-19), 24.2 (C-18); **HRMS** (ESI+) m/z = 666.3390 $[M+H]^+$ found, $C_{37}H_{44}N_7O_5^+$ required 666.3398.

Methyl (*Z*)-2-(6-(2-(cyclohexylamino)-1-(4-((4-methoxyphenyl)ethynyl)phenyl)-2-oxoethyl)-4,7-dioxo-1^1*H*-3,6-diaza-1(4,1)-triazola-5(1,3)-benzenacyclotetradecaphane-3-yl)acetate (62)

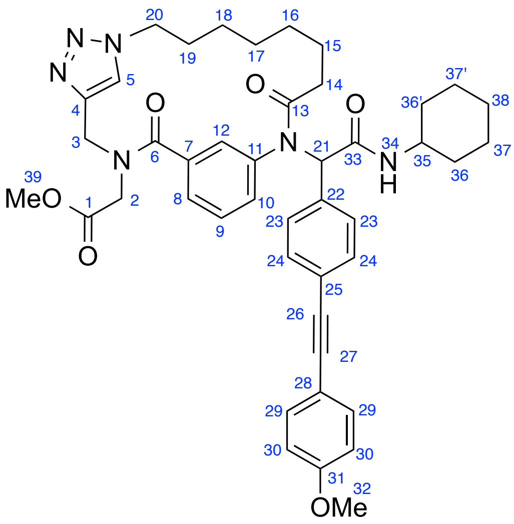

43a (70 mg, 0.092 mmol, 1.0 eq), copper iodide (3.4 mg, 0.018 mmol, 0.20 eq), and $PdCl_2(PPh_3)_2$ (13 mg, 0.018 mmol, 0.20 eq) were suspended in dry MeCN (6.5 mL). TEA (64 µL, 0.46 mmol, 5.0 eq) and ethynyl anisole (33 µL, 0.25 mmol, 2.8 eq) were added. The mixture was reacted in the µλ for 2 h (in total) at 110 °C whilst being monitored by TLC and LCMS. Then, copper iodide (3.4 mg, 0.018 mmol, 0.20 eq), $PdCl_2(PPh_3)_2$ (13 mg, 0.018 mmol, 0.20 eq), TEA (64 µL, 0.46 mmol, 5.0 eq), and ethynyl anisole (33 µL, 0.25 mmol, 2.8 eq) were added, and the reaction mixture was again reacted in the µλ for 1 h at 110 °C. After completion by LCMS, the solvent was removed. The residue was filtered through a short column of silica and washed with EtOAc and then CH_2Cl_2. The residue was purified by F-SPE and the fluorophobic fraction was concentrated. After purification by preparative HPLC (30-100% B), **62** (26 mg, 0.034 mmol, 35%) was obtained as a dark yellow oil.

TLC R_f = 0.29 (neat EtOAc); **HPLC** t_r = 11.36 min (30-100% B), peak area 100%; **IR** ν_{max} (neat)/cm^{-1} = 3250 w (N-H), 1752 m (C=O), 1636 m (C=O), 1602 m (C=O); ^{1}**H NMR** (500 MHz, DMSO-d_6, 120 °C) δ = 7.92 (s, 1H; H-5), 7.48-7.39 (m, 3H; H-29, H-34), 7.39-7.33 (m, 1H; H-12), 7.31-7.25 (m, 4H; H-9, H-8, H-24), 7.24-7.19 (brs, 1H; H-10), 7.12 (d, J = 8.2 Hz, 2H; H-23), 6.96 (d, J = 8.7 Hz, 2H; H-30), 6.10 (s, 1H; H-21), 4.62 (d, J = 15.8 Hz, 1H; H-3a), 4.58 (d, J = 16.0 Hz, 1H; H-3b), 4.39 (t, J = 5.8 Hz, 2H; H-20), 4.14 (d, J = 17.3 Hz, 1H; H-2a), 4.05 (d, J = 17.3 Hz, 1H; H-2b), 3.82 (s, 3H; H-32), 3.69 (s, 3H; H-39), 3.67 (m, 1H; H-35), 1.90 (t, J = 6.7 Hz, 2H; H-14), 1.87-1.76 (m, 3H; H-19, and either H-36a or H-36’a), 1.75-1.68 (m, 2H; either H-36a or H-36’a, and either H-37a or H-37’a), 1.67-1.59 (m, 1H; H-37a or H-37’a), 1.59-1.51 (m, 1H; H-38a), 1.41-1.34 (m, 2H; H-15), 1.34-1.22 (m, 3H; H-37b, H-37’b, and either H-36b or H-36’b), 1.20-1.11 (m, 2H; H-38b and either H-36b or H-36’b), 1.11-1.01 (m, 4H; H-16 and H-18), 1.01-0.93 (m, 2H; H-17); ^{13}**C NMR** (125 MHz, DMSO-

d_6, 120 °C) δ = 172.5 (C-13), 169.5 (C-6), 168.9 (C-1), 168.7 (C-33), 142.5 (C-4), 139.6 (C-11 or C-7), 135.3 (C-22), 133.2 (C-29), 132.3 (C-10), 131.0 (C-24 × 2), 130.5 (C-23 × 2), 129.5 (C-12), 129.2 (C-8 or C-10), 126.5 (C-9), 123.4 (C-5), 121.7 (C-25), 115.0 (C-30 × 2 and C-28), 90.4 (C-27), 88.1 (C-26), 64.3 (C-21), 55.8 (C-32), 52.1 (C-39), 49.8 (C-20), 48.6 (C-35), 34.2 (C-14), 32.5 (C-36 or C-36'), 32.4 (C-36 or C-36'), 28.9 (C-19), 28.4 (C-16), 27.5 (C-17), 26.1 (C-18), 25.7 (C-38), 25.6 (C-15), 24.9 (C-37 or C-37'), 24.7 (C-37 or C-37'); **HRMS** (ESI+) *m/z* = 781.3691 $[M+Na]^+$ found, $C_{45}H_{50}N_6O_6Na^+$ required 781.3690.

Methyl 2-(9,10-dihydroxy-3,13-dioxo-2,4,12-triaza-1(1,3)-benzenacyclotridecaphane-12-yl)acetate (63)

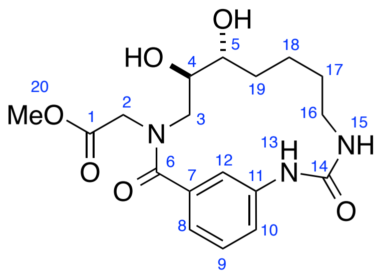

53a (4.0 mg, 0.012 mmol, 1.0 eq) was dissolved in acetone/water (10:1, 1 mL). *N*-methylmorpholine *N*-oxide (4.0 mg, 0.034 mmol, 2.8 eq) and osmium tetroxide (3.3 µL, 2.5wt% in tBuOH, 0.33 µmol, 0.028 eq) were added. The mixture stirred at rt for 30 hours and then the solvent was removed under a stream of nitrogen. The crude mixture was purified by preparative HPLC (5-35% B), **63** (1.4 mg, 3.7 µmol, 32%) was obtained as a white solid.

TLC R_f = 0.31 (CH_2Cl_2/MeOH 10:1); **HPLC** t_r = 5.07 min (5-100% B), peak area 100%; **IR** ν_{max} (neat)/cm^{-1} = 3343 brm, 2925 w, 1741 w, 1627 s, 1542 m, 1428 m, 1406 m, 1204 s, 1176 s, 1136 s, 1082 s, 1045 s; **^{1}H NMR** (500 MHz, DMSO-d_6, 27 °C) δ = 8.18 (s, 1H; H-13), 7.47-7.35 (m, 2H; H-9 and H-12), 7.23-7.16 (m, 1H; H-10), 7.11-7.04 (m, 1H; H-8), 5.97 (brs, 1H; H-15), 4.36 (d, *J* = 17.1 Hz, 1H; H-2a), 4.23 (d, *J* = 17.1 Hz, 1H; H-2b), 3.72-3.67 (m, 4H; H-20 and H-4), 3.28-3.20 (m, coincides with water signal; H-3), 3.21-3.09 (m, 2H; H-16a and H-5), 3.02-2.88 (m, 1H; H-16b), 1.57 (dq, *J* = 13.3, 6.6 Hz, 1H; H-19a), 1.52-1.36 (m, 2H; H-17), 1.36-1.26 (m, 2H; H-18), 1.21-1.11 (m, 1H; H-19b); **^{13}C NMR** (125 MHz, DMSO-d_6, 27 °C) due to interconversion, C-8 and C-10 are missing, δ = 171.3 (C-6), 169.8 (C-1), 157.0 (C-14), 139.2 (C-11), 136.6 (C-7), 129.7 (C-9), 71.9 (C-20), 70.8 (C-5), 53.4 (C-3), 51.8 (C-4), 48.1 (C-2), 40.0 (C-16, coincides with solvent signal), 31.5 (C-19), 27.8 (C-17), 22.1 (C-18); **HRMS** (ESI+) *m/z* = 380.1829 $[M+H]^+$ found, $C_{18}H_{26}N_3O_6^+$ required 380.1816.

Methyl (*E*)-2-(2-(2-methoxyacetyl)-3,13-dioxo-2,4,12-triaza-1(1,3)-benzenacyclotridecaphan-9-en-12-yl)acetate (64a)

and **methyl (*E*)-2-(4-(2-methoxyacetyl)-3,13-dioxo-2,4,12-triaza-1(1,3)-benzenacyclotridecaphan-9-en-12-yl)acetate (64b)**

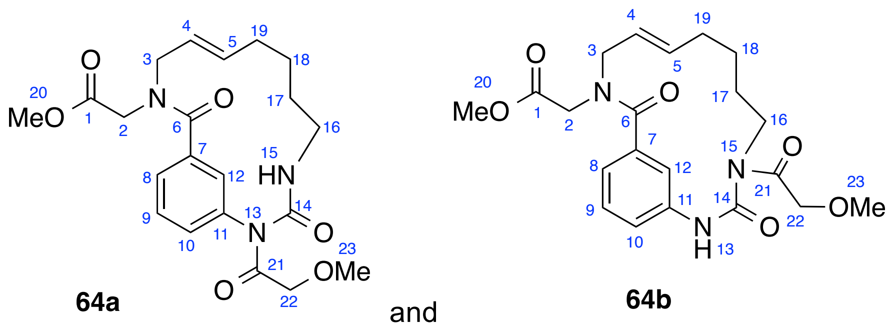

53a (3.8 mg, 0.011 mmol, 1.0 eq) was dissolved in CH_2Cl_2 (1 mL). Methoxyacetyl chloride (6.0 µL, 0.065 mmol, 5.9 eq) and triethylamine (29 µL, 0.21 mmol, 19 eq) were added. The mixture stirred at 40 °C overnight. The solvent was removed and residue purified by preparative HPLC (10-100% B). **64a** (2.4 mg, 5.7 µmol, 53%) was obtained as a white solid as the major product, and **64b** (1.6 mg, 3.8 µmol, 35%) was obtained as a yellow solid as the minor product.

64a

TLC R_f = 0.35 (EtOAc); **HPLC** t_r = 8.19 min (5-100% B), peak area 97%; **IR** ν_{max} (neat)/cm^{-1} = 3385 w, 2963 w, 2920 w, 1748 m, 1687 s, 1631 m, 1511 m, 1439 m, 1403 m, 1247 m, 1204 s, 1182 s, 1102 s, 803 s; **^{1}H NMR** (500 MHz, $CDCl_3$, 27 °C) δ = 8.07 (brs, 1H; H-15), 7.68 (dt, *J* = 7.7, 1.4 Hz, 1H; H-8), 7.57 (t, *J* = 7.8 Hz, 1H; H-9), 7.39 (ddd, *J* = 7.8, 2.1, 1.1 Hz, 1H; H-10), 7.24 (t, *J* = 1.9 Hz, 1H; H-12, coincides with solvent signal), 5.85 (dt, *J* = 16.2, 7.2 Hz, 1H; H-5), 5.43 (dt, *J* = 15.6, 5.0 Hz, 1H; H-4), 4.18 (s, 2H; H-2), 3.86 (d, *J* = 3.7 Hz, 2H; H-3), 3.84 (s, 2H; H-22), 3.79 (s, 3H; H-20), 3.36 (s, 3H; H-23), 3.34-3.25 (m, 2H; H-16), 2.05 (q, *J* = 6.2 Hz, 2H; H-19), 1.73 (s, coincides with water signal; H-17), 1.49-1.39 (m, 2H; H-18); **^{13}C NMR** (125 MHz, $CDCl_3$, 27 °C) δ = 172.1 (C-21), 171.7 (C-6), 169.5 (C-1), 154.0 (C-14), 137.2 (C-7), 136.5 (C-11), 132.8 (C-5), 131.3 (C-10), 130.5 (C-9), 128.9 (C-8), 127.2 (C-12), 124.9 (C-4), 72.0 (C-22), 59.5 (C-23), 52.5 (C-20), 52.3 (C-3), 47.7 (C-2), 40.5 (C-16), 30.7 (C-19), 24.6 (C-17), 24.0 (C-18); **HRMS** (ESI+) *m/z* = 440.1790 $[M+Na]^+$ found, $C_{21}H_{27}N_3O_6Na^+$ required 440.1792.

64b

TLC R_f = 0.51 (EtOAc); **HPLC** t_r = 8.59 min (5-100% B), peak area 100%; **IR** ν_{max} (neat)/cm^{-1} = 3361 br.m, 1683 s, 1434 m, 1205 s, 1137 s; **^{1}H NMR** (500 MHz, $CDCl_3$, 27 °C) δ = 7.73 (s, 1H; H-13),

7.45-7.49 (m, 2H; H-12 and either H-8 or H-10), 7.43 (t, *J* = 8.0 Hz; H-9), 7.30 (coincides with solvent signal; H-8 or H-10), 5.64-5.41 (m, 2H; H-4 and H-5), 4.32 (apps, 2H; H-2 or H-22), 4.16 (apps, 2H; H-2 or H-22), 3.90 (apps, 2H; H-3), 3.86 (apps, 2H; H-16), 3.78 (s, 3H; H-20 or H-23), 3.49 (s, 3H; H-20 or H-23), 2.13 (q, J = 6.4, 5.7 Hz, 2H; H-19), 1.78-1.67 (m, 2H; H-17), 1.51-1.44 (coincides with water signal; H-18); **HRMS** (ESI+) *m/z* = 418.1967 $[M+H]^+$ found, $C_{21}H_{28}N_3O_6^+$ required 418.1973.

References

[1] H. S. G. Beckmann, F. Nie, C. E. Hagerman, H. Johansson, Y. S. Tan, D. Wilcke, D. R. Spring, *Nature Chem.* **2013**, *5*, 861.

[2] F. Kopp, C. F Stratton, L. B. Akella, D. S. Tan, *Nat. Chem. Biol.* **2012**, *8*, 358.

[3] F. Giordanetto, J. Kihlberg, *J. Med. Chem.* **2014**, *57*, 278.

NMR Spectra

^1H and ^{13}C NMR spectra of novel compounds.

4

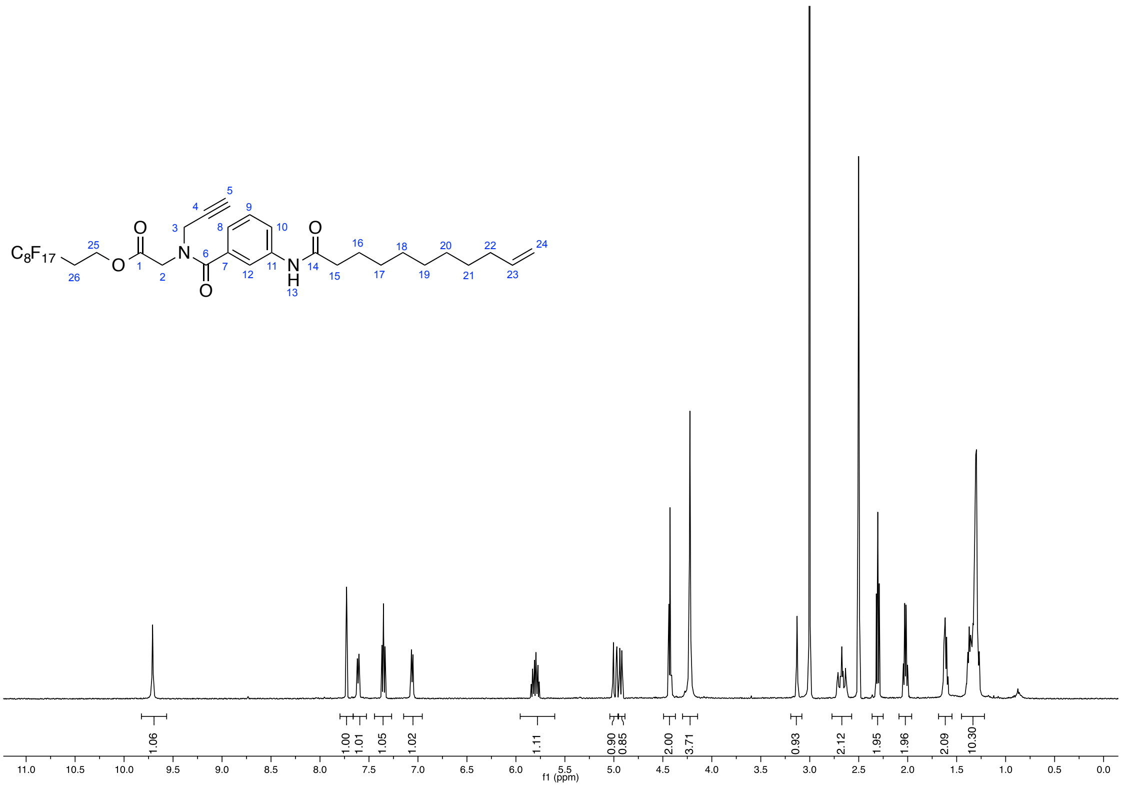

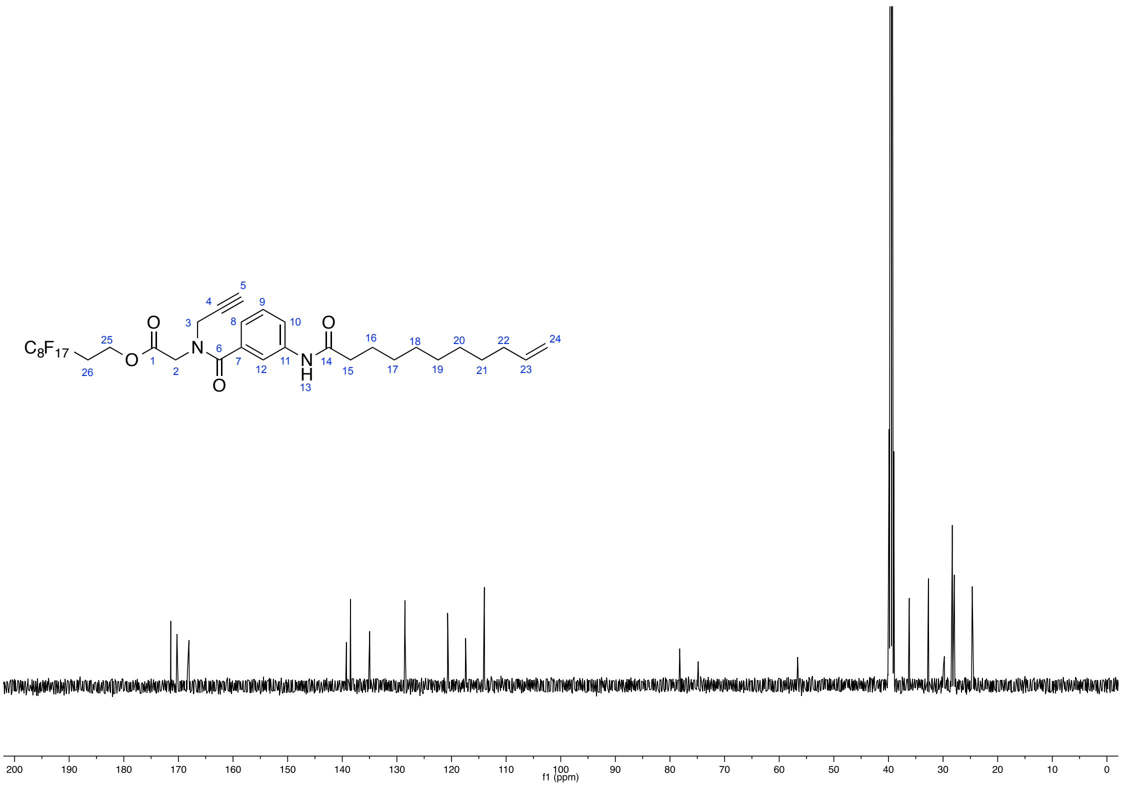

5a1

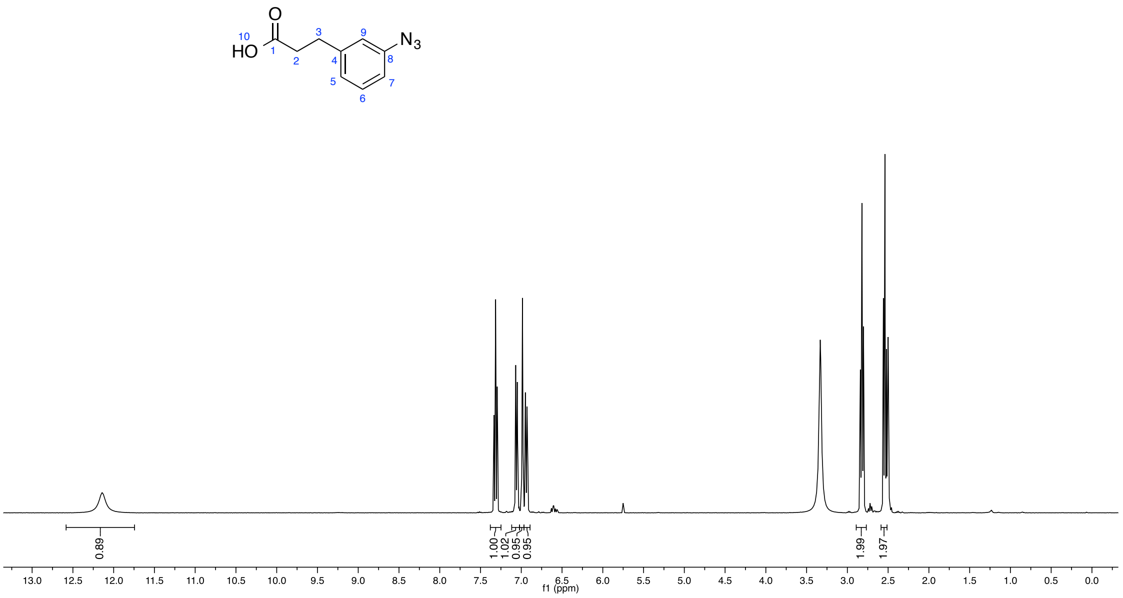

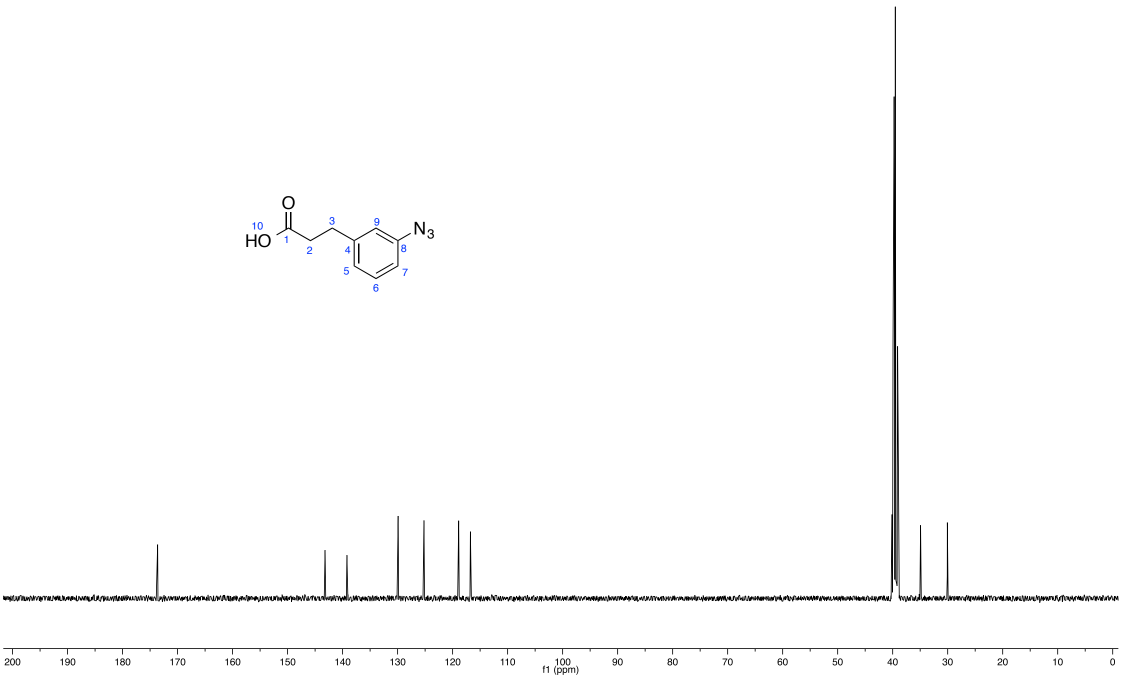

5

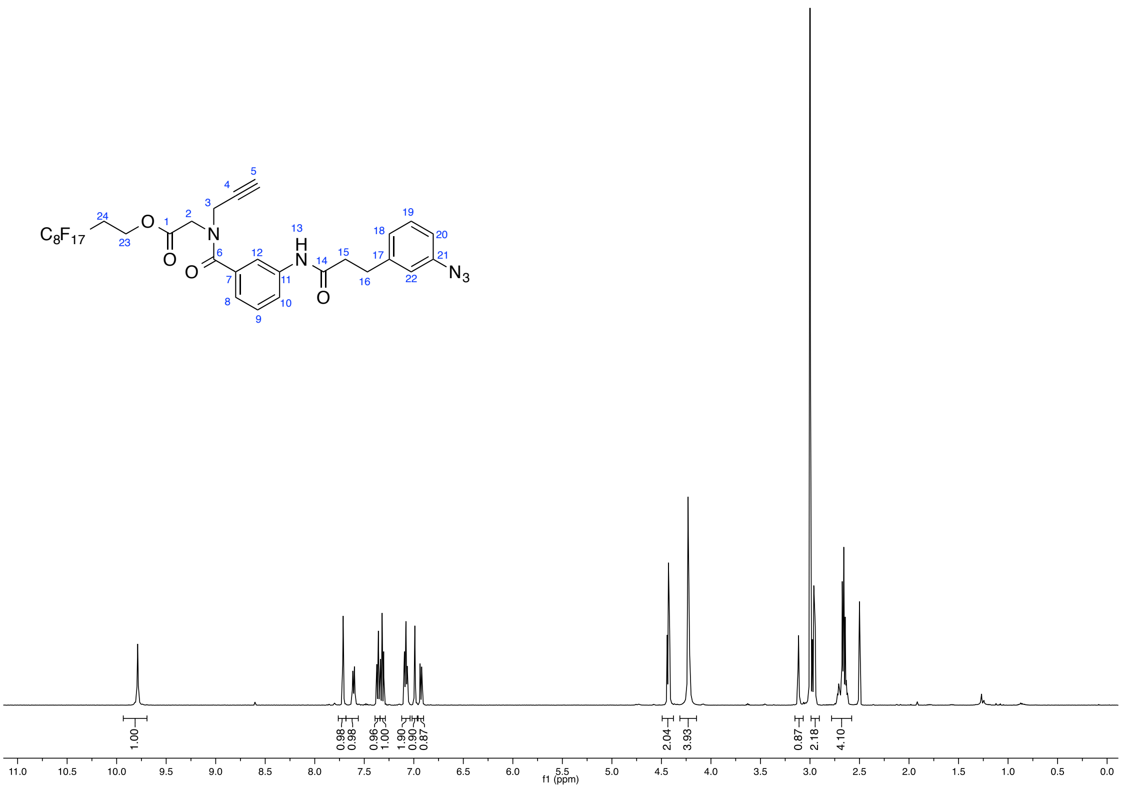

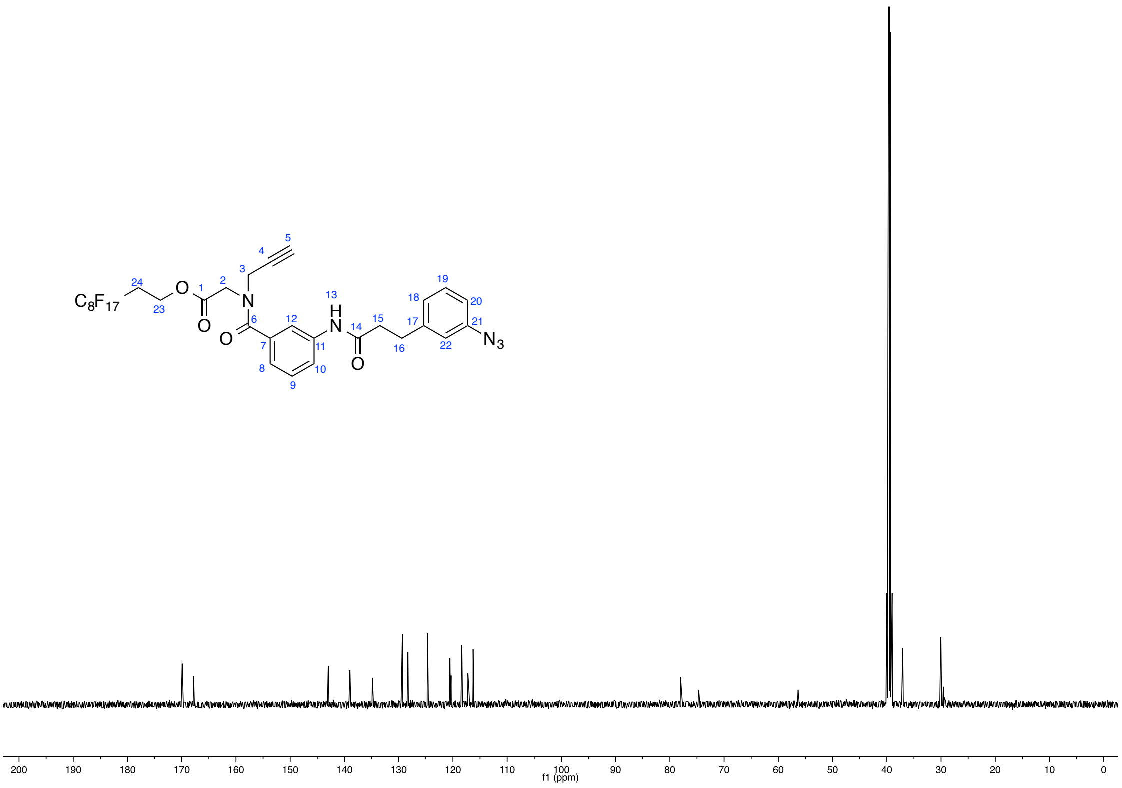

7a1

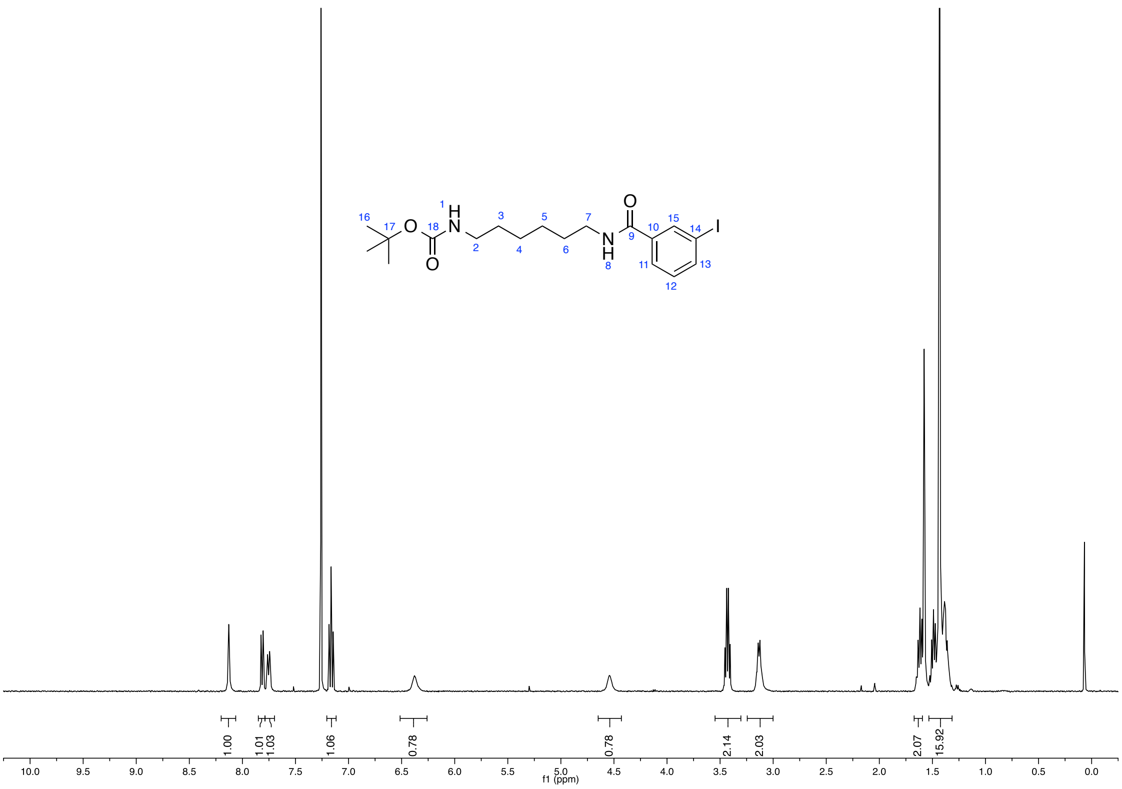

1.00
1.01
1.03
1.06
0.78
0.78
2.14
2.03
2.07
15.92
10.0 9.5 9.0 8.5 8.0 7.5 7.0 6.5 6.0 5.5 5.0 4.5 4.0 3.5 3.0 2.5 2.0 1.5 1.0 0.5 0.0
f1 (ppm)

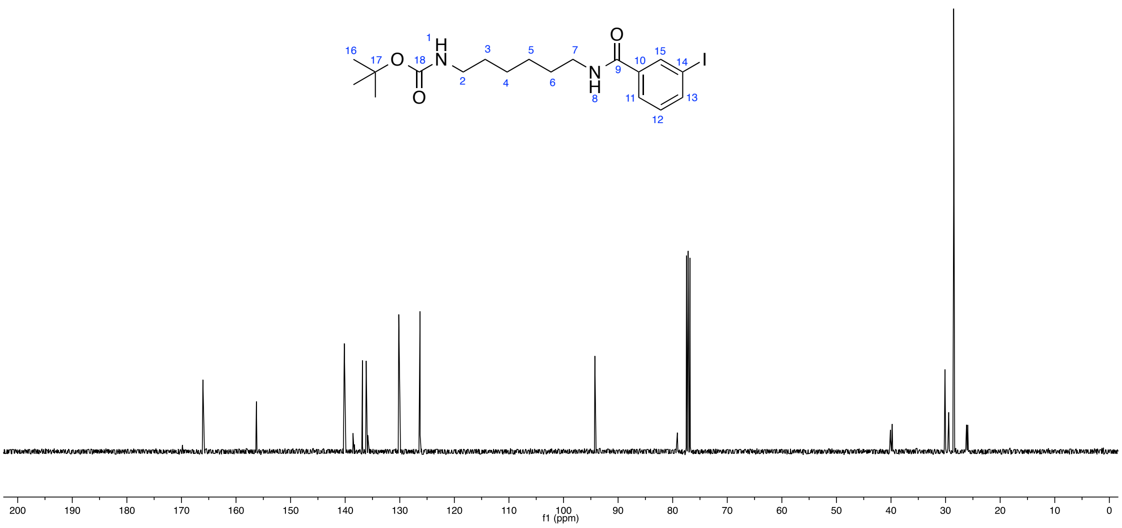

200 190 180 170 160 150 140 130 120 110 100 90 80 70 60 50 40 30 20 10 0
f1 (ppm)

7a

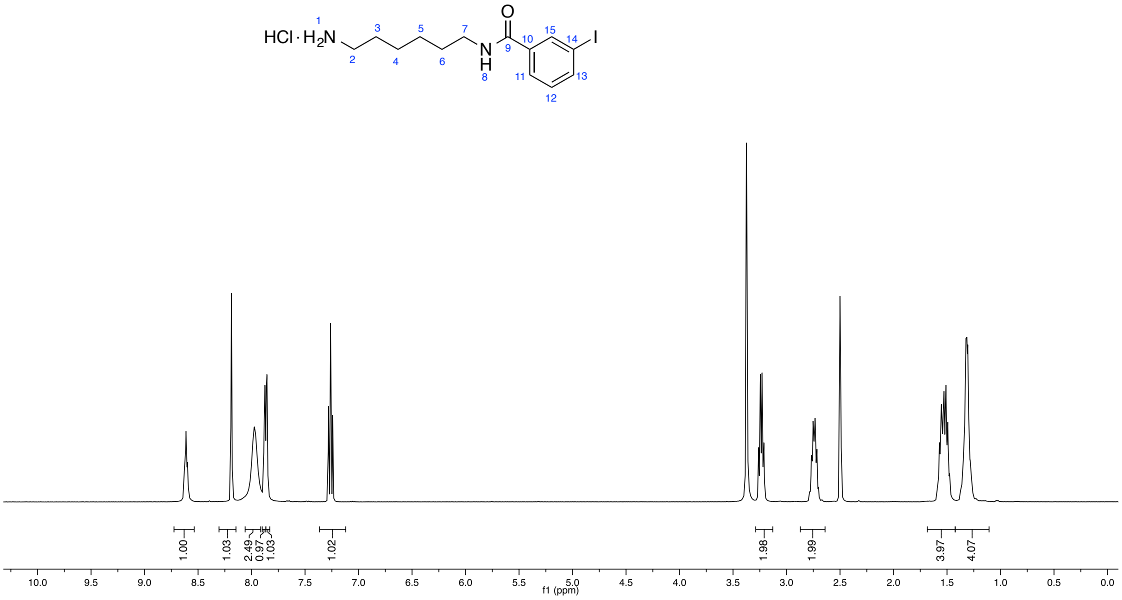
HCl · H2N
O
N
H
I
1.00
1.03
2.49
0.97
1.03
1.02
1.98
1.99
3.97
4.07
f1 (ppm)

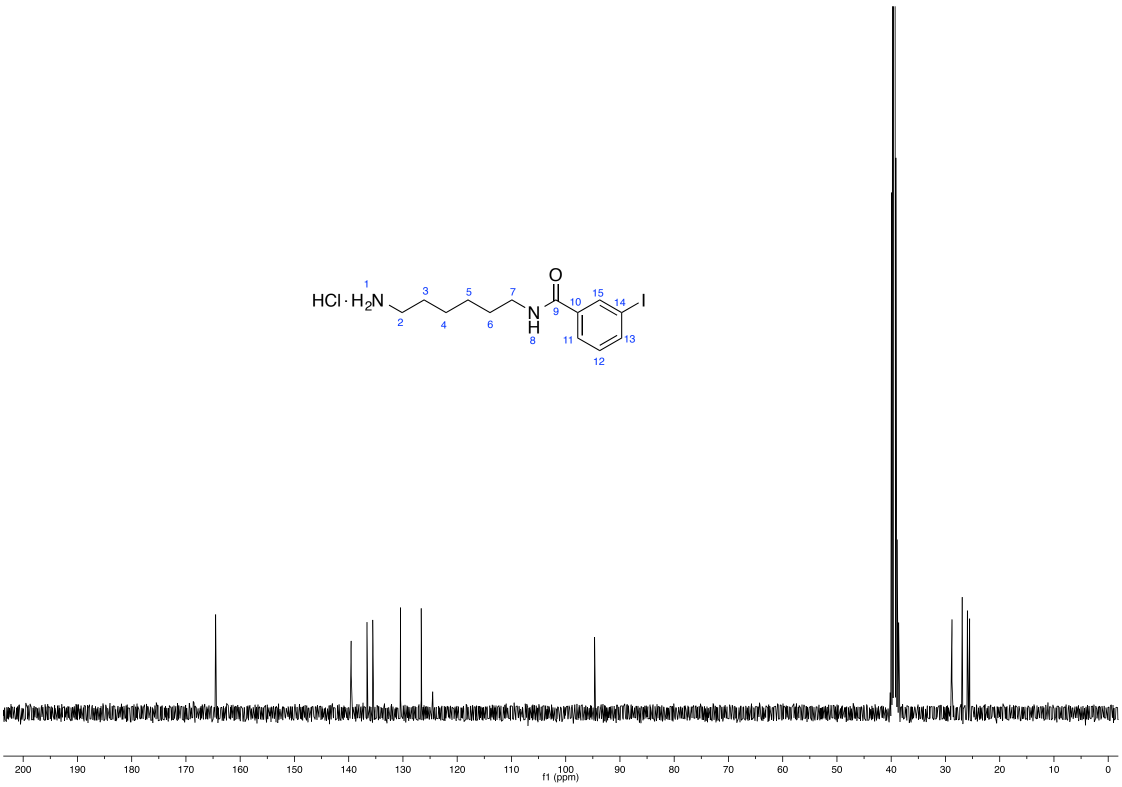
HCl · H2N
O
N
H
I
f1 (ppm)

7

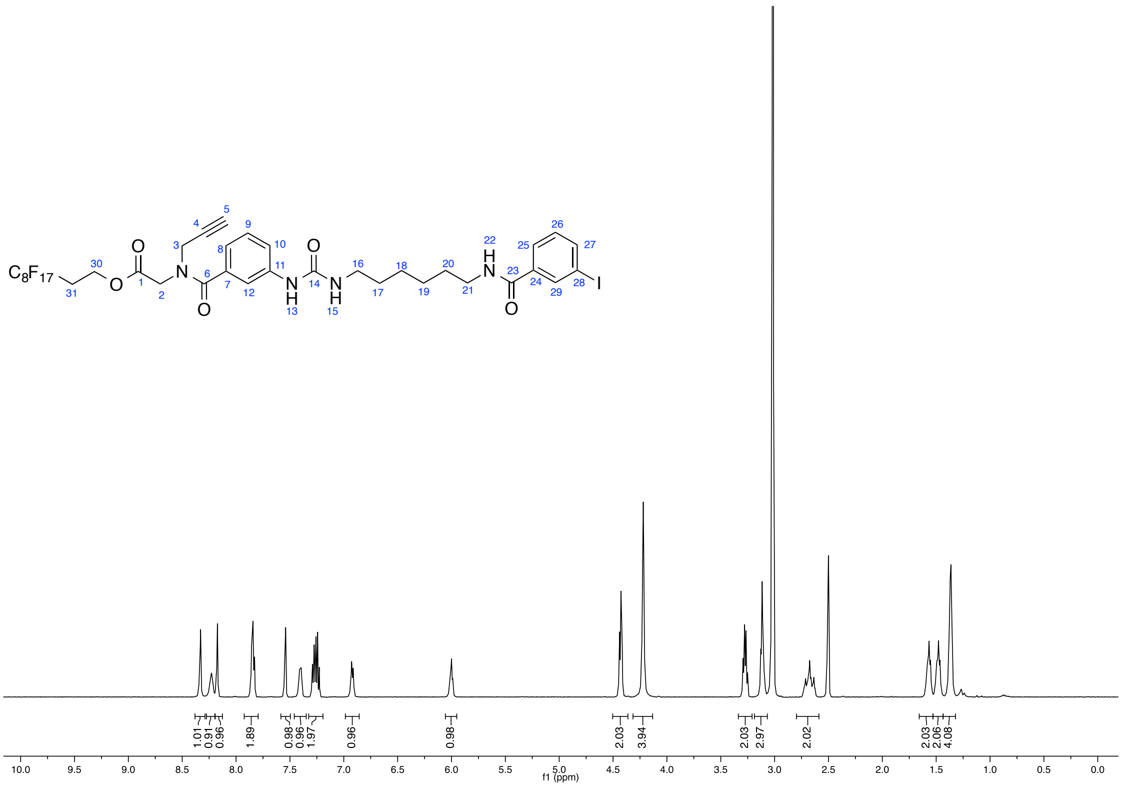

C8F17
1.01
0.91
0.96
1.89
0.98
0.96
1.97
0.96
0.98
2.03
3.94
2.03
2.97
2.02
2.03
2.06
4.08
f1 (ppm)

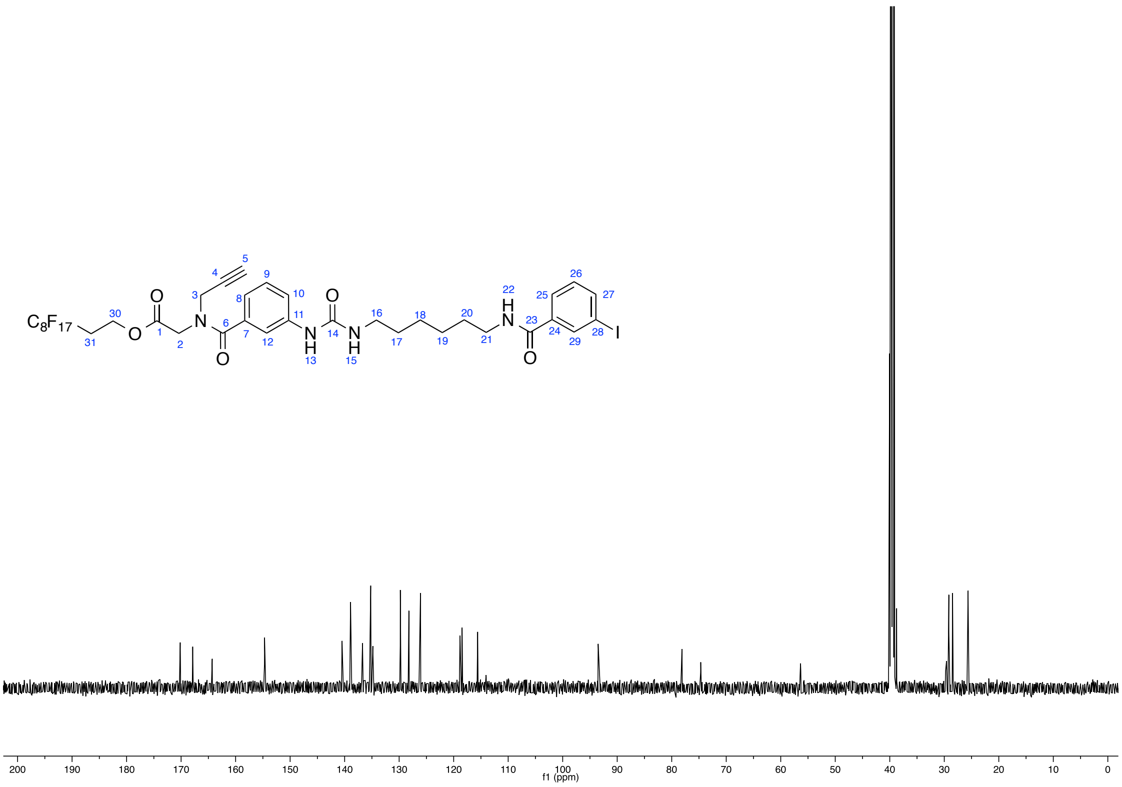

C8F17
f1 (ppm)

8a

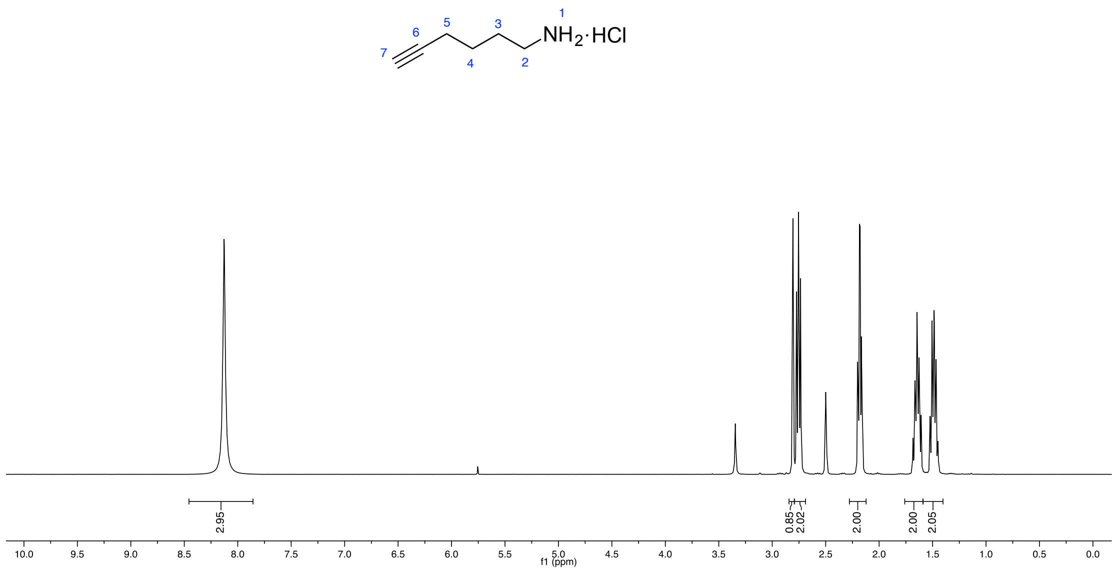

NH2·HCl
1
2
3
4
5
6
7
2.95
0.85
2.02
2.00
2.00
2.05
f1 (ppm)

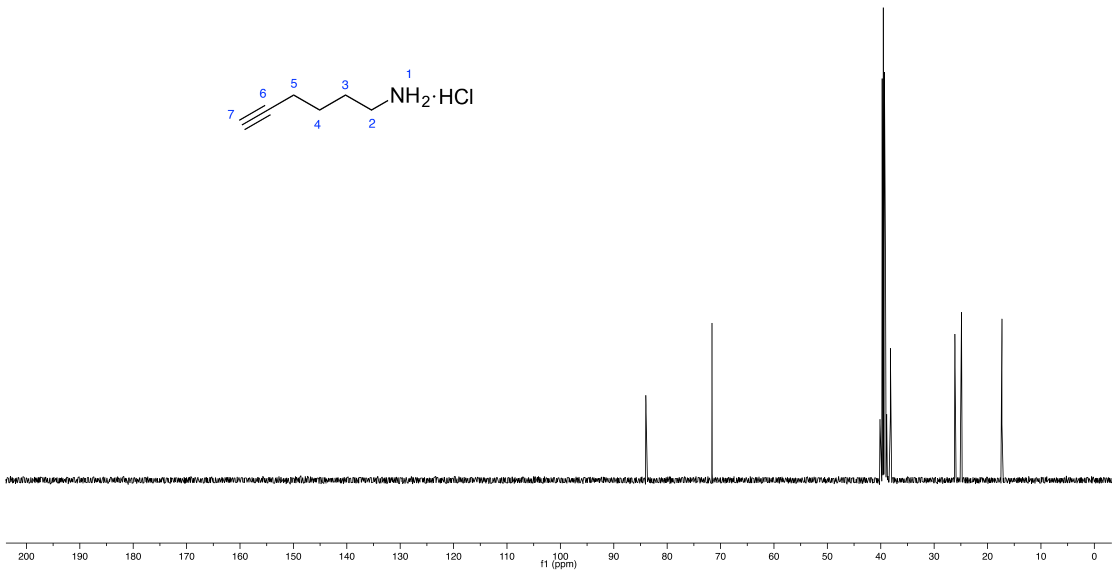

NH2·HCl
1
2
3
4
5
6
7
f1 (ppm)

8

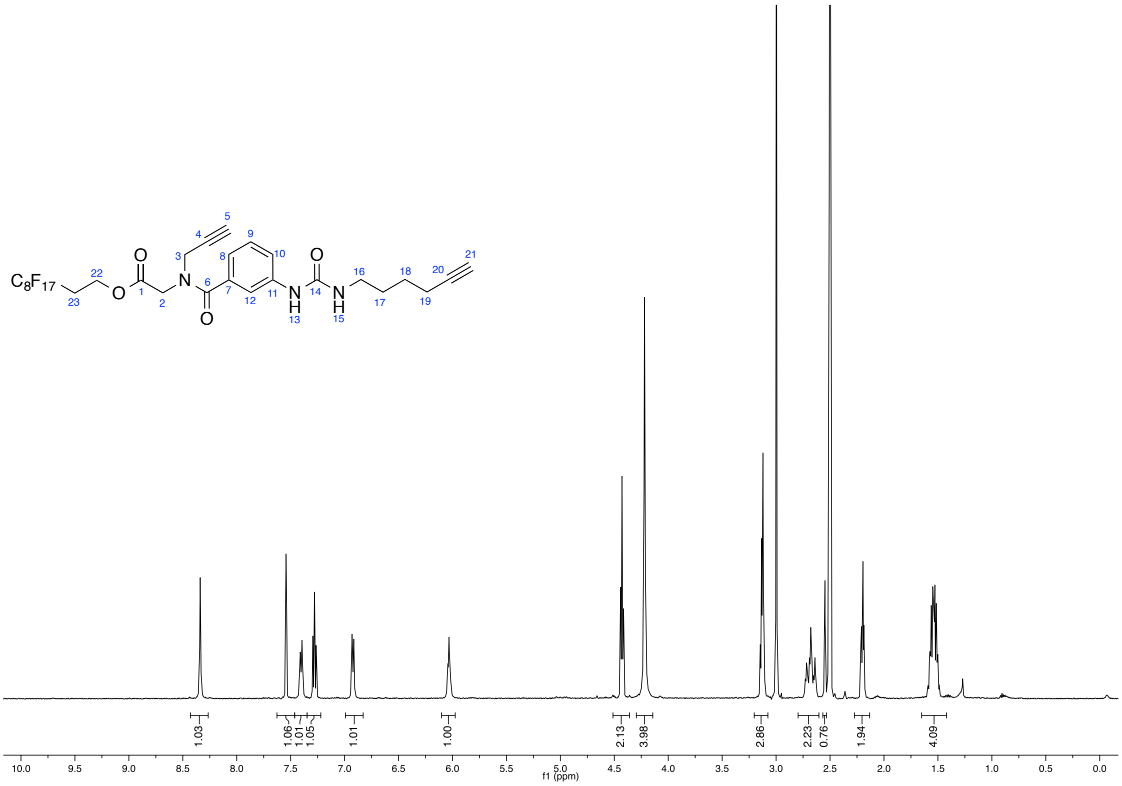
C8F17
1.03
1.06
1.01
1.05
1.01
1.00
2.13
3.98
2.86
2.23
0.76
1.94
4.09
10.0 9.5 9.0 8.5 8.0 7.5 7.0 6.5 6.0 5.5 5.0 4.5 4.0 3.5 3.0 2.5 2.0 1.5 1.0 0.5 0.0
f1 (ppm)

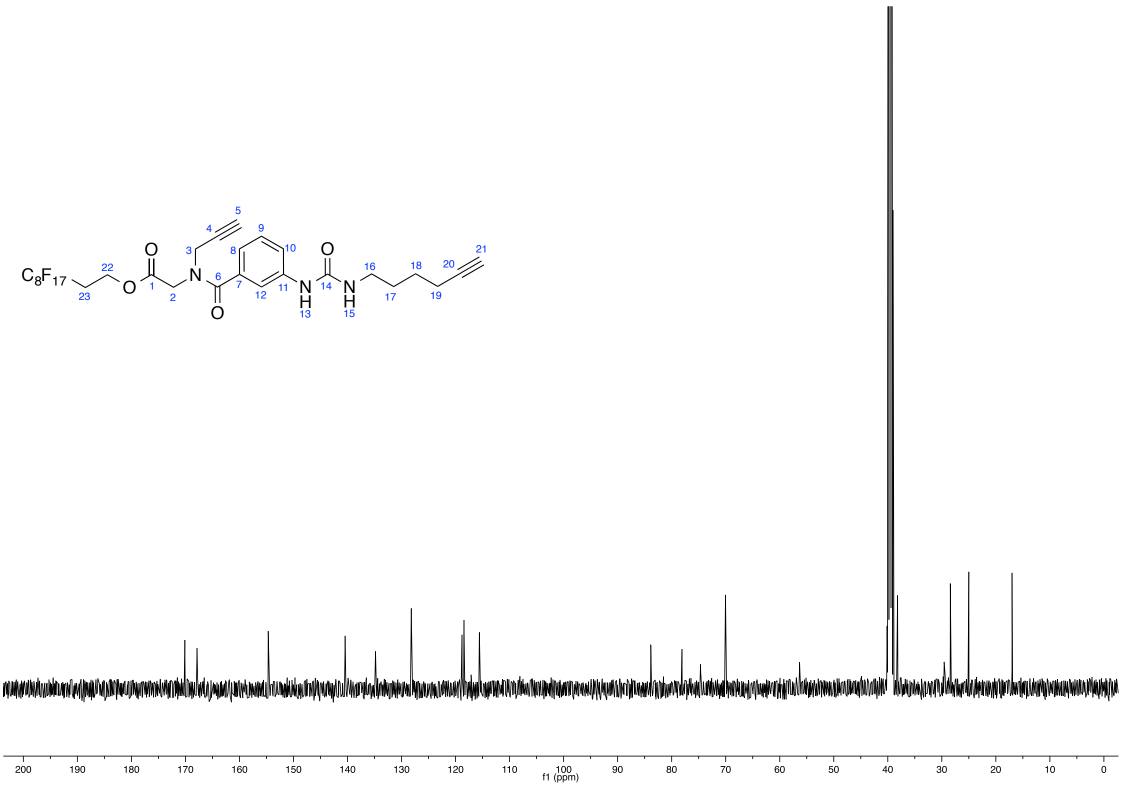
C8F17
200 190 180 170 160 150 140 130 120 110 100 90 80 70 60 50 40 30 20 10 0
f1 (ppm)

10

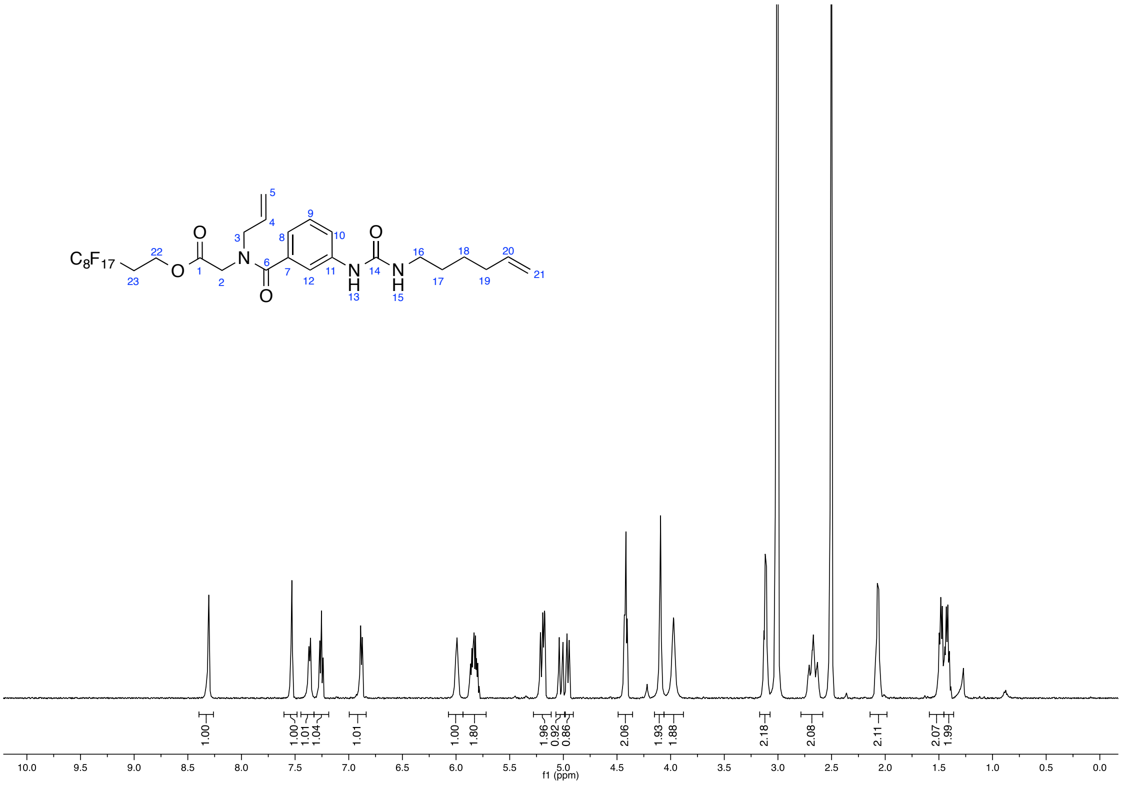

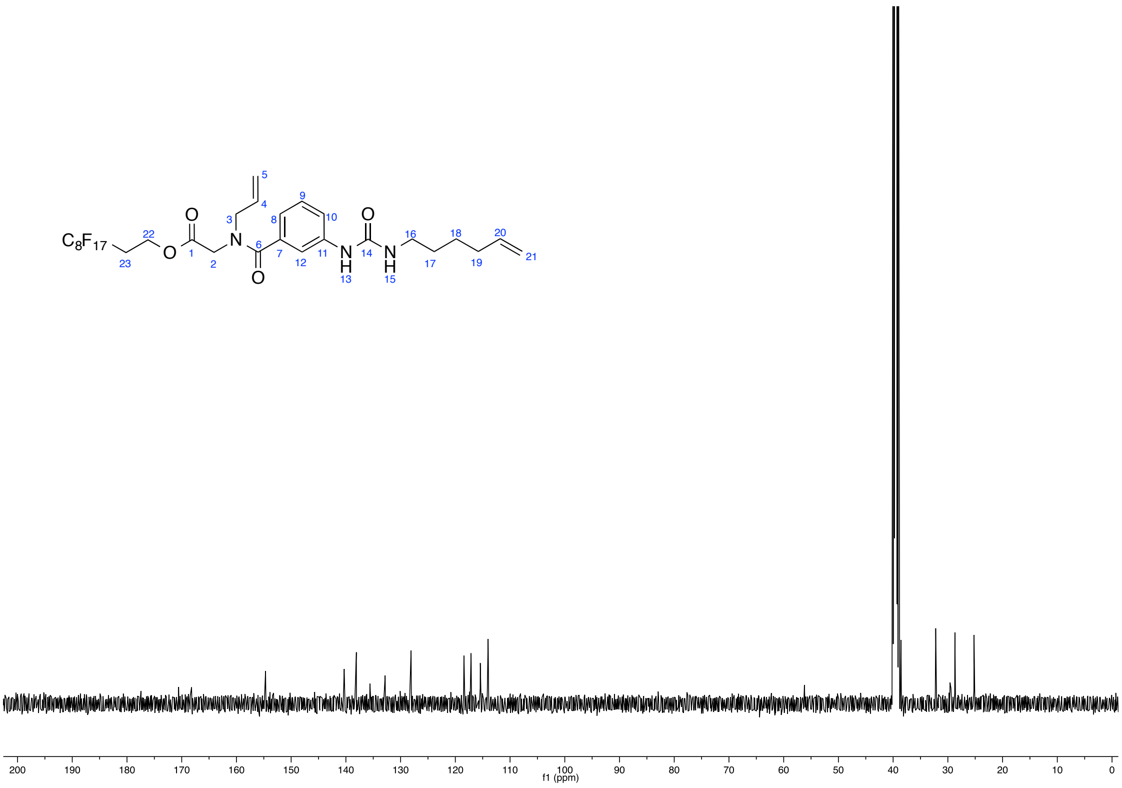

11

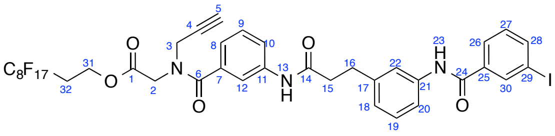

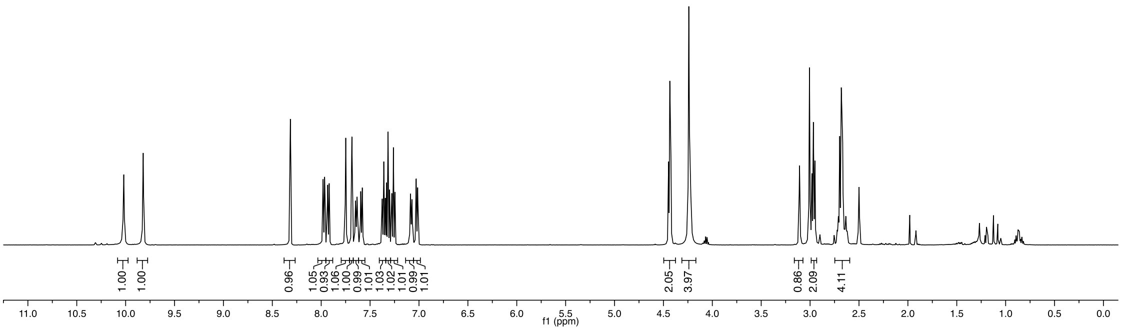

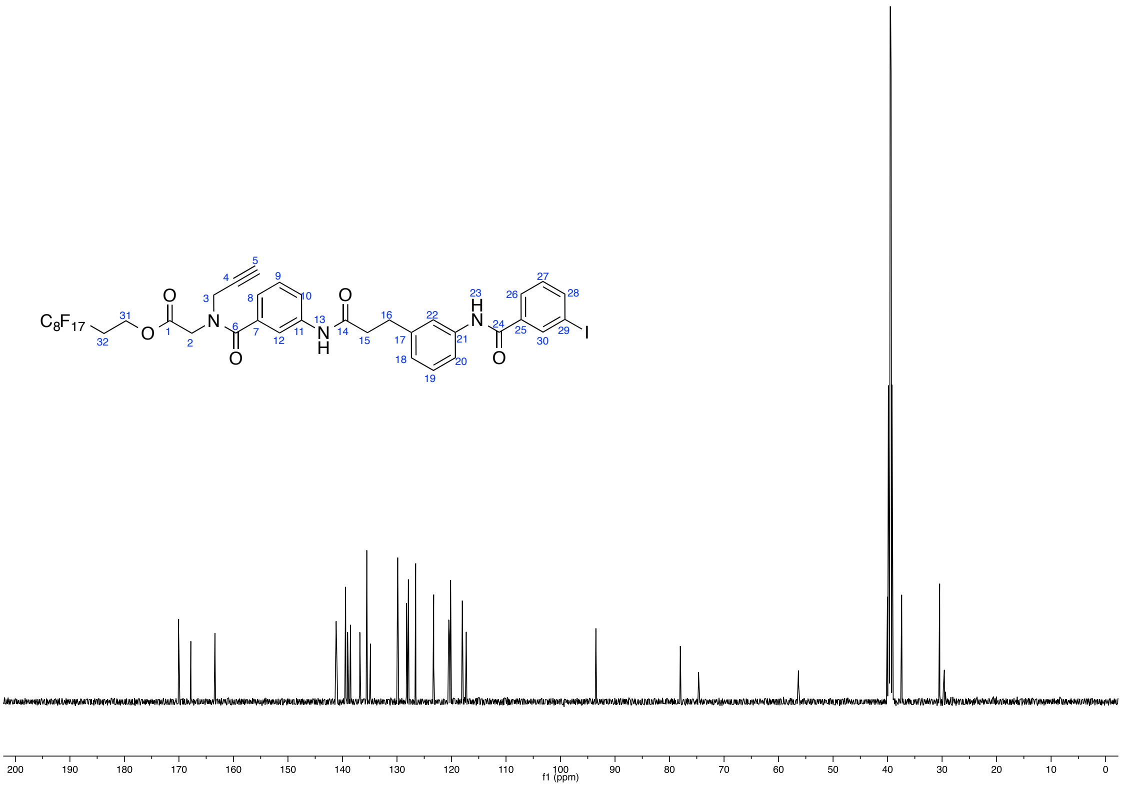

12

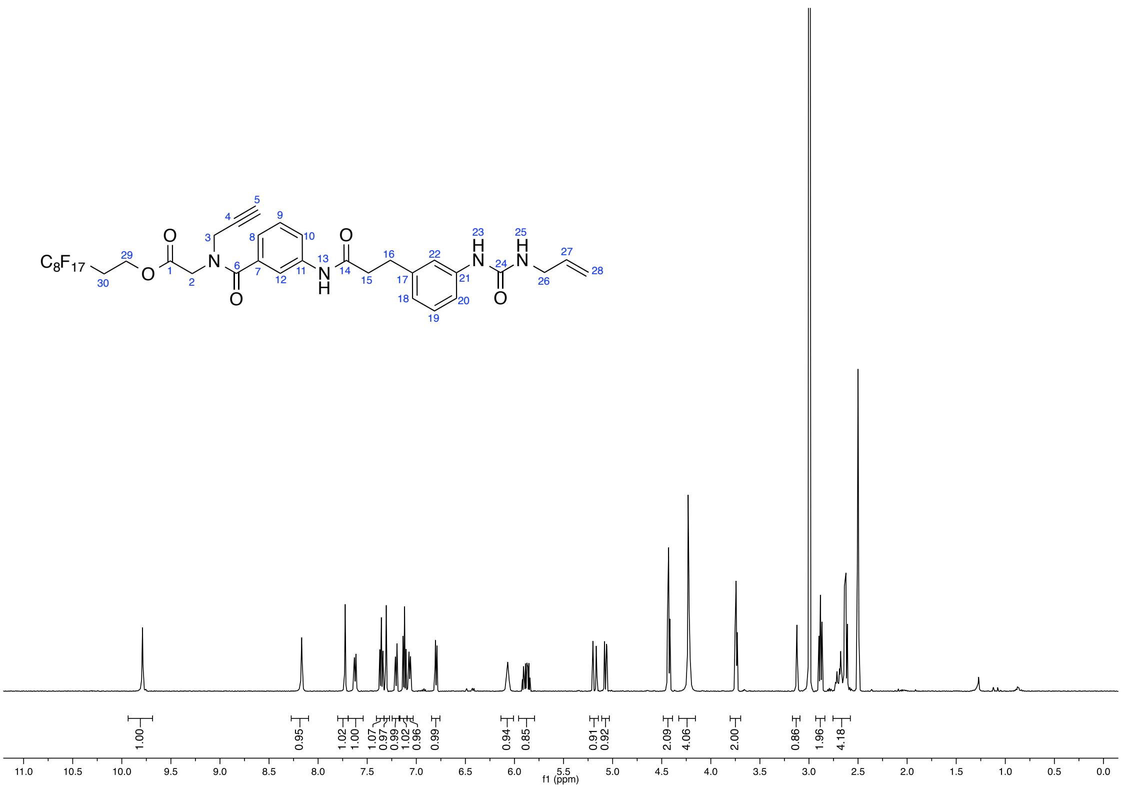

C8F17
O
N
NH
NH
NH
O
1.00
0.95
1.02
1.00
1.07
0.97
0.99
1.02
0.96
0.99
0.94
0.85
0.91
0.92
2.09
4.06
2.00
0.86
1.96
4.18
f1 (ppm)

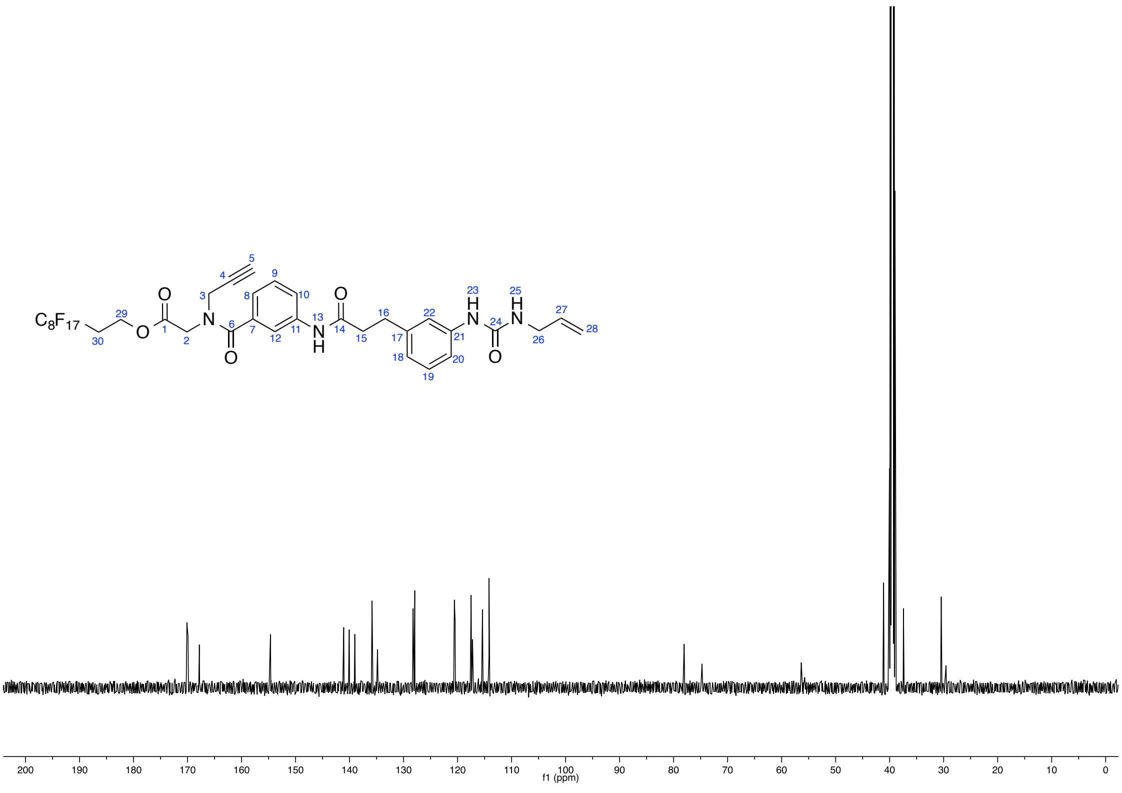

C8F17
f1 (ppm)

13a

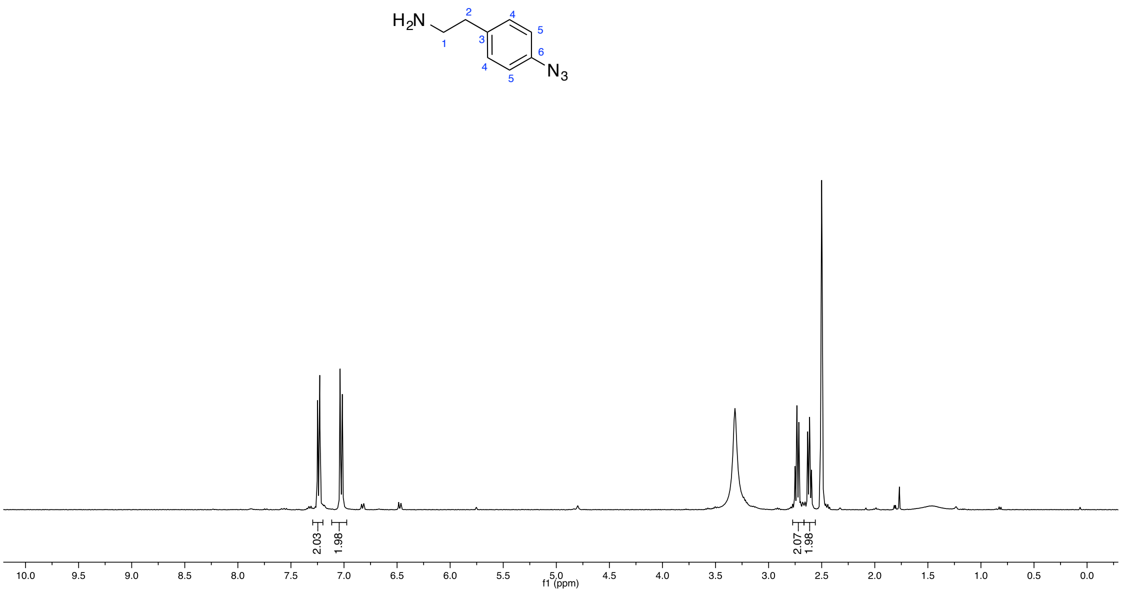

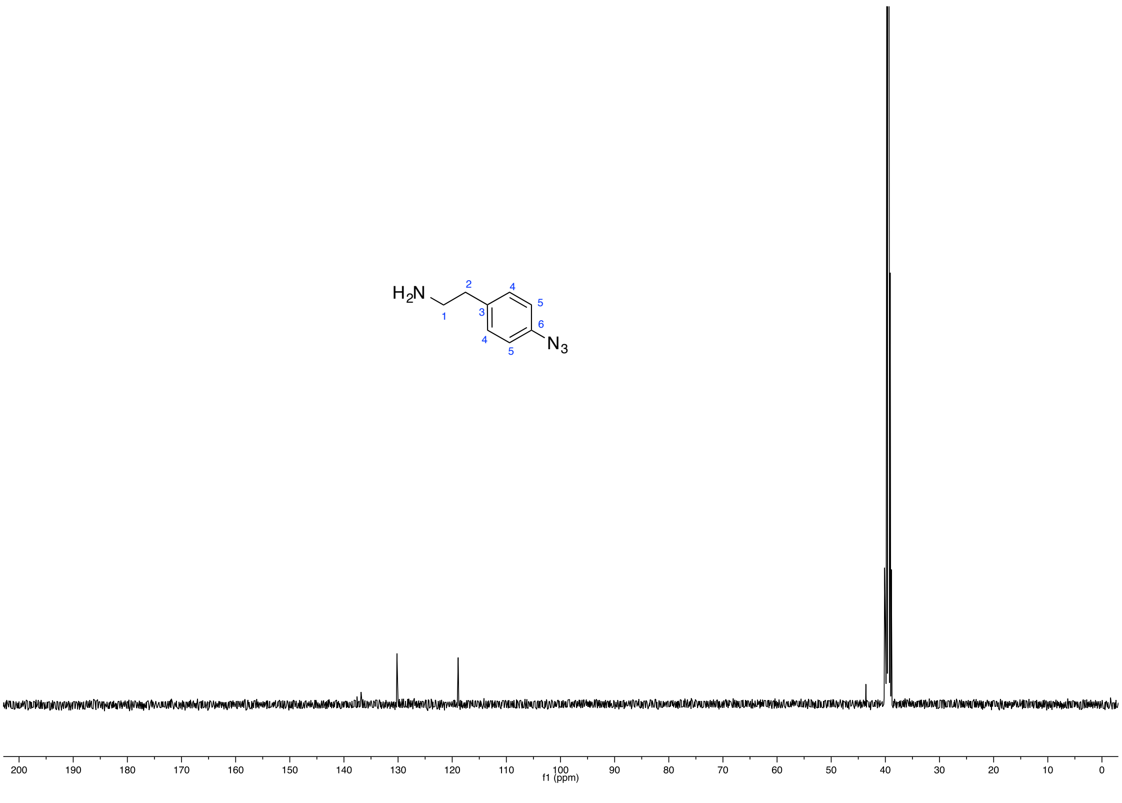

13

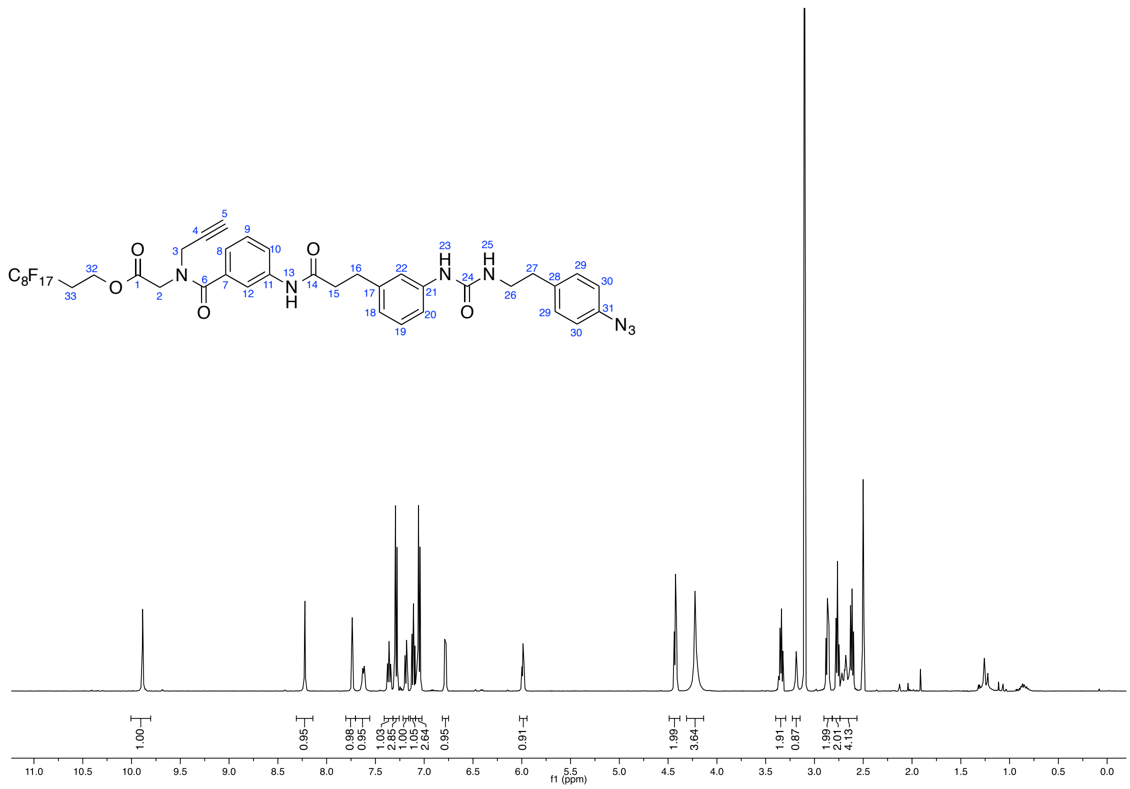

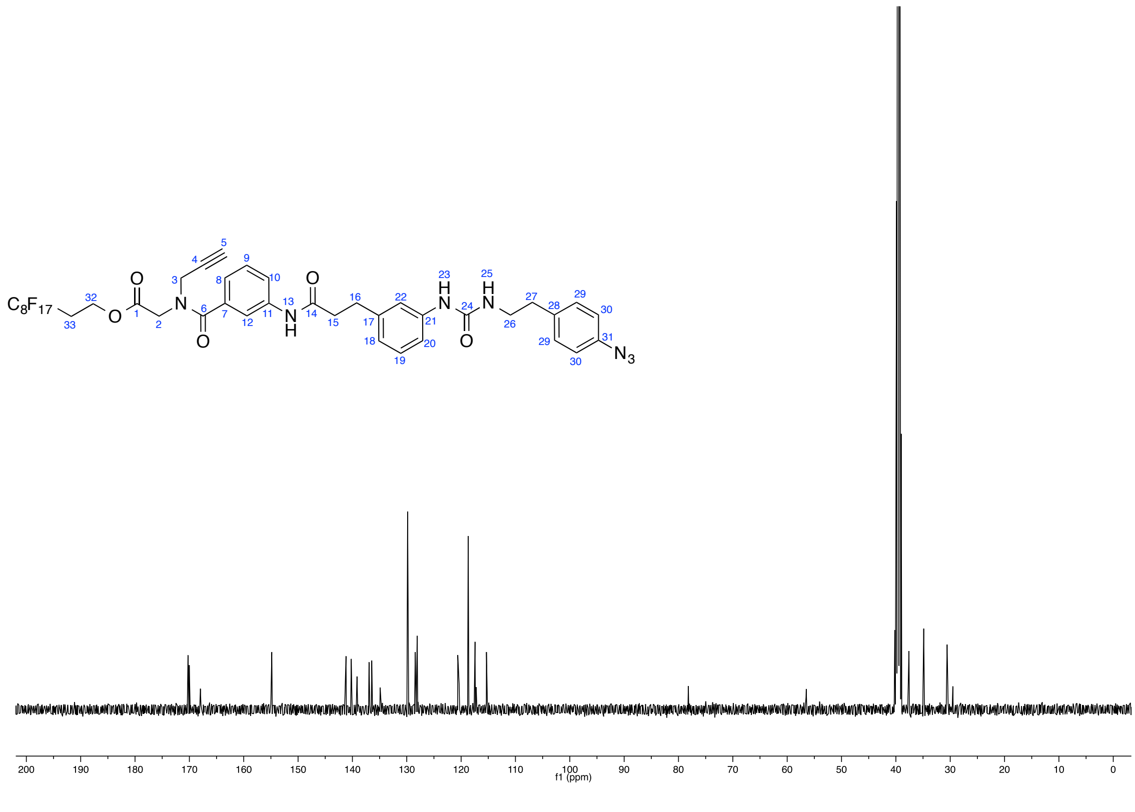

16a1

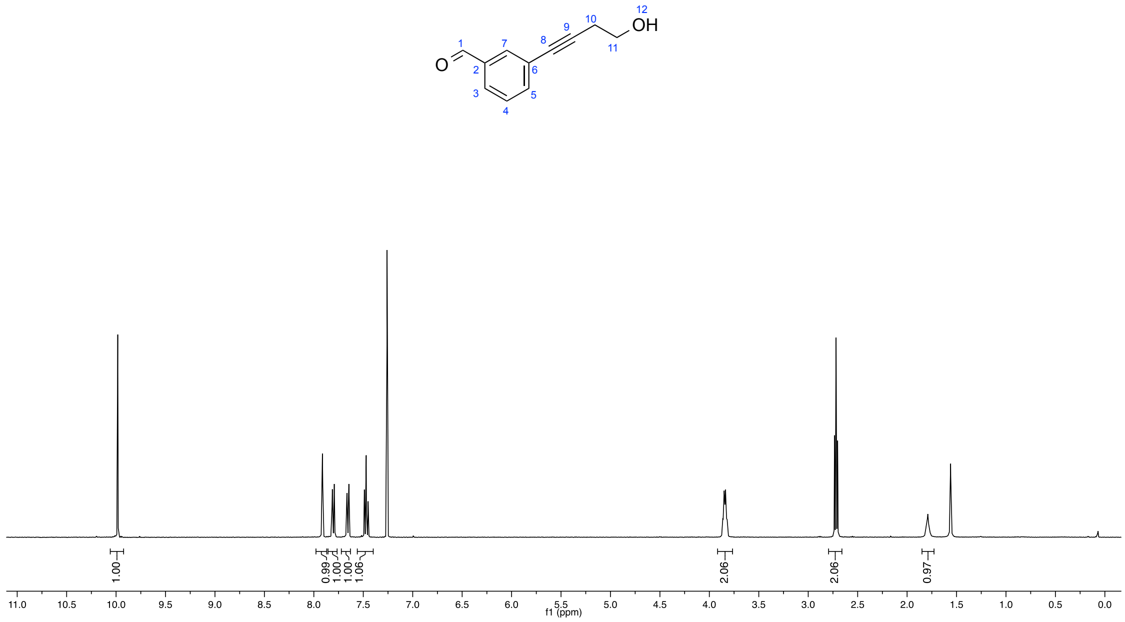

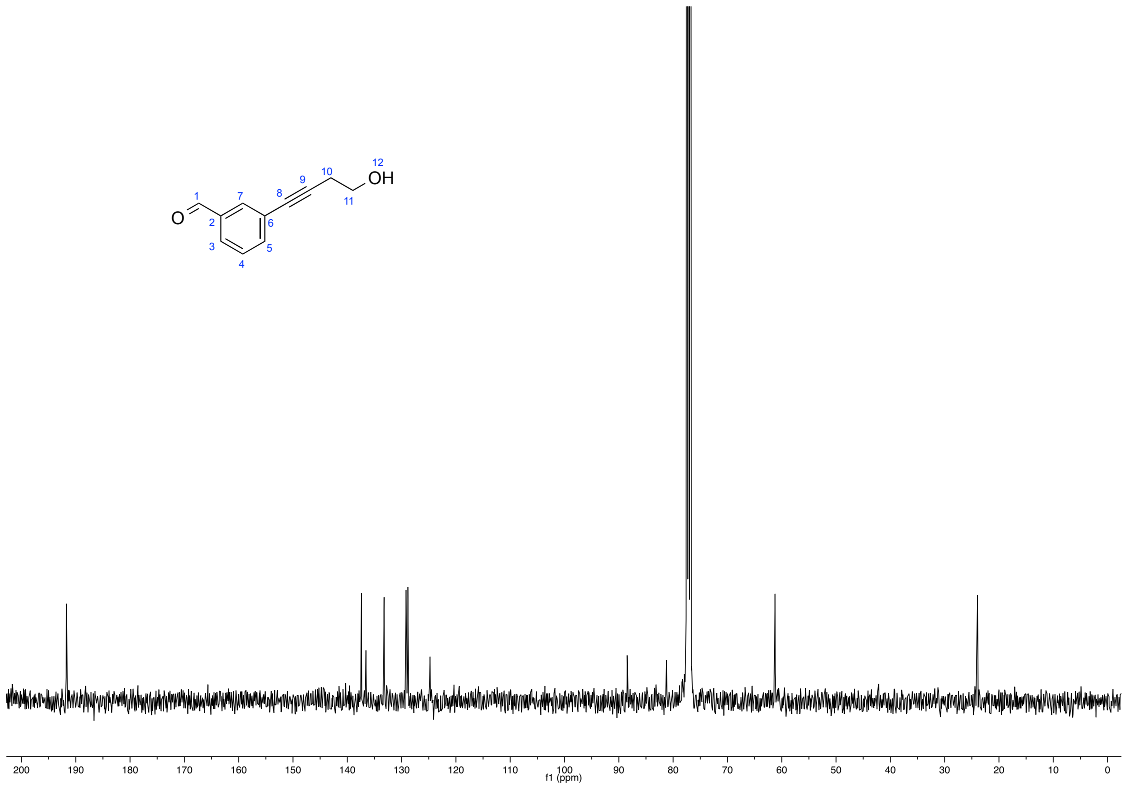

16a2

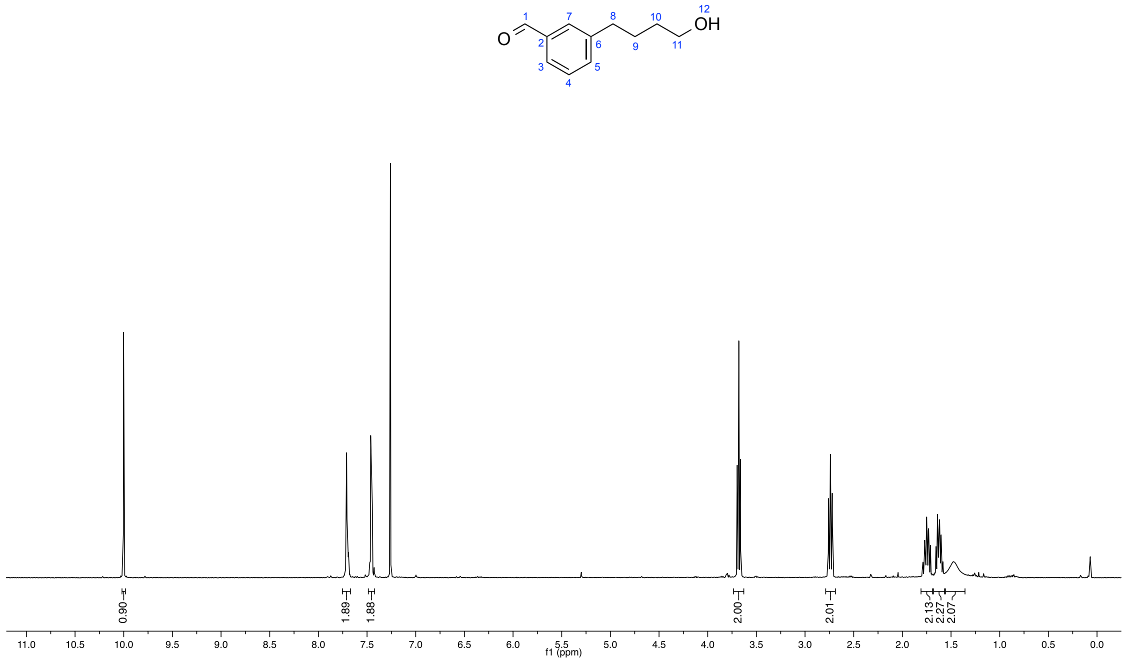

1
7
8
10
12
OH
O
2
6
9
11
3
5
4
0.90
1.89
1.88
2.00
2.01
2.13
2.27
2.07
11.0
10.5
10.0
9.5
9.0
8.5
8.0
7.5
7.0
6.5
6.0
5.5
5.0
4.5
4.0
3.5
3.0
2.5
2.0
1.5
1.0
0.5
0.0
f1 (ppm)

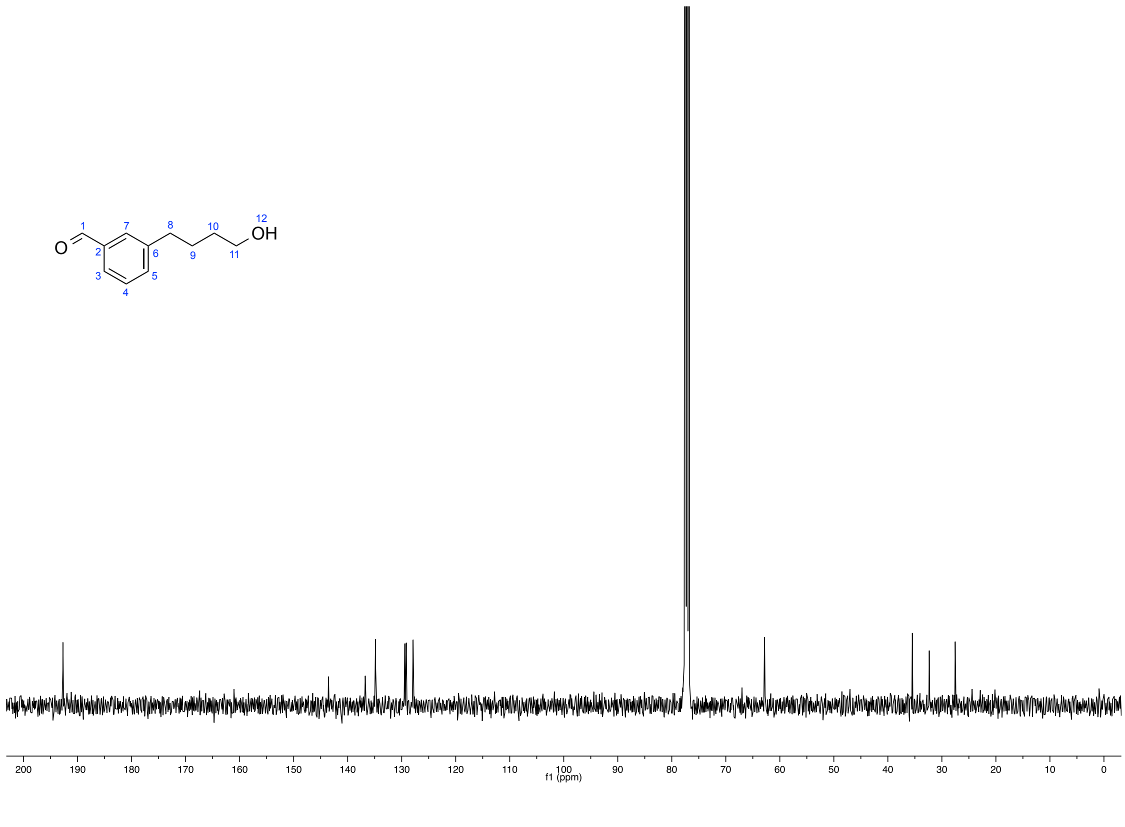

1
7
8
10
12
OH
O
2
6
9
11
3
5
4
200
190
180
170
160
150
140
130
120
110
100
90
80
70
60
50
40
30
20
10
0
f1 (ppm)

16a

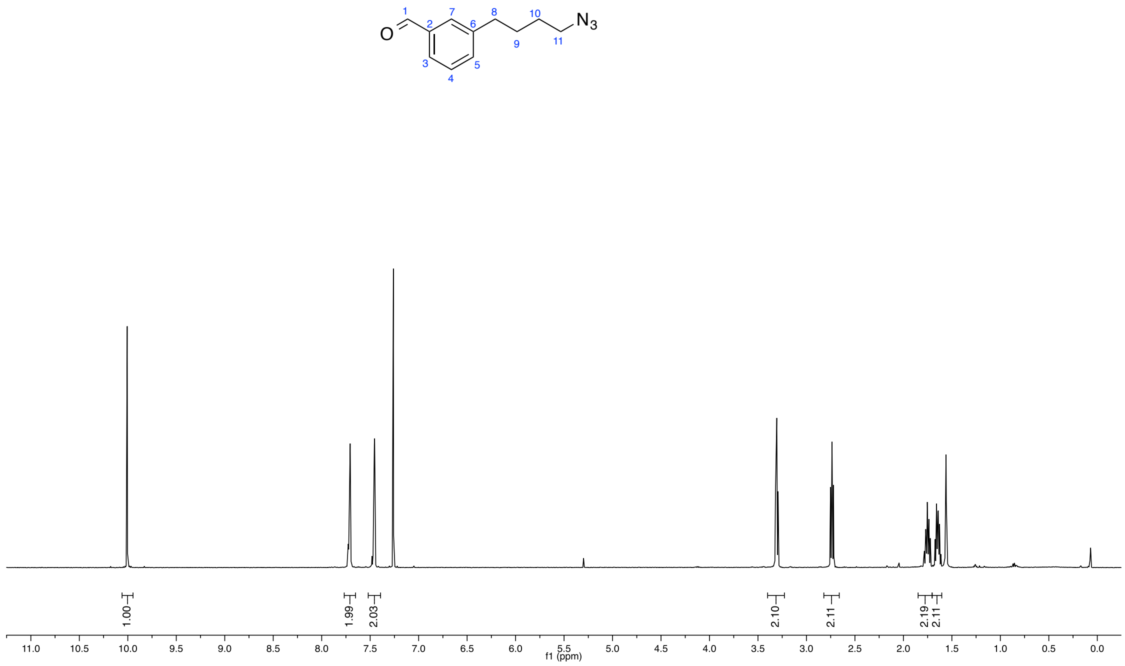

O
N3
1 2 3 4 5 6 7 8 9 10 11
1.00
1.99
2.03
2.10
2.11
2.19
2.11
11.0 10.5 10.0 9.5 9.0 8.5 8.0 7.5 7.0 6.5 6.0 5.5 5.0 4.5 4.0 3.5 3.0 2.5 2.0 1.5 1.0 0.5 0.0
f1 (ppm)

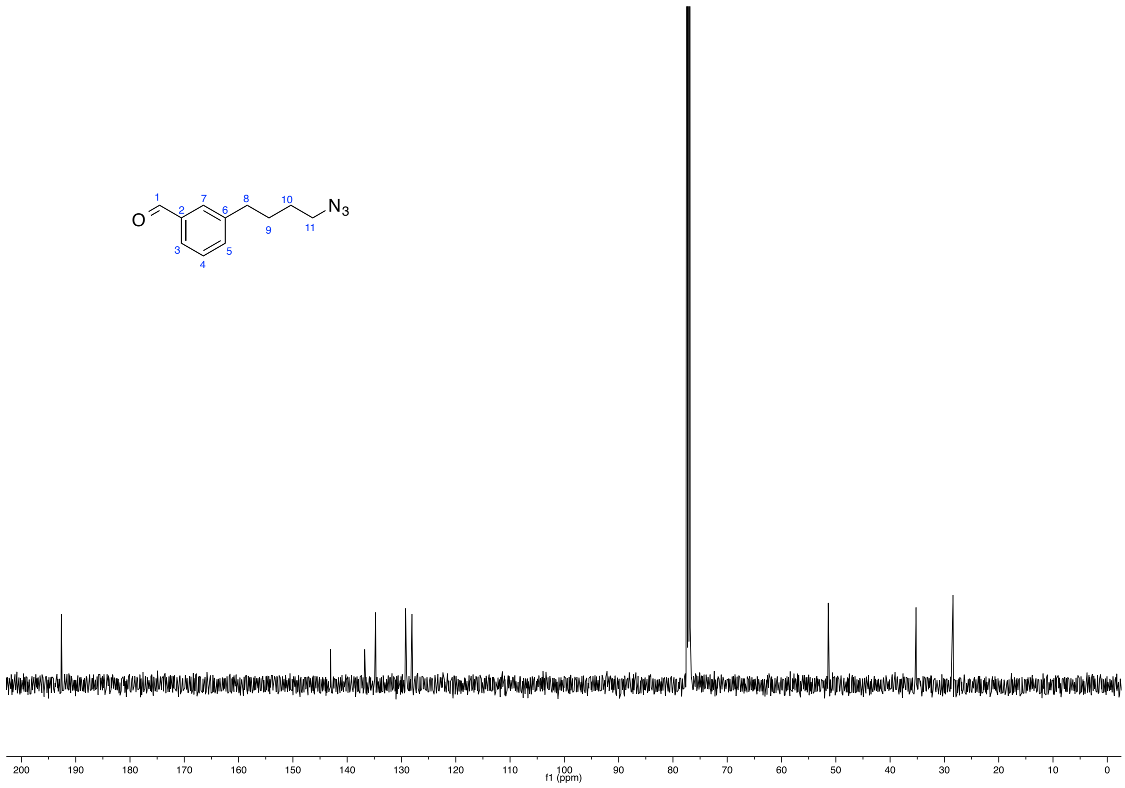

O
N3
1 2 3 4 5 6 7 8 9 10 11
200 190 180 170 160 150 140 130 120 110 100 90 80 70 60 50 40 30 20 10 0
f1 (ppm)

18a

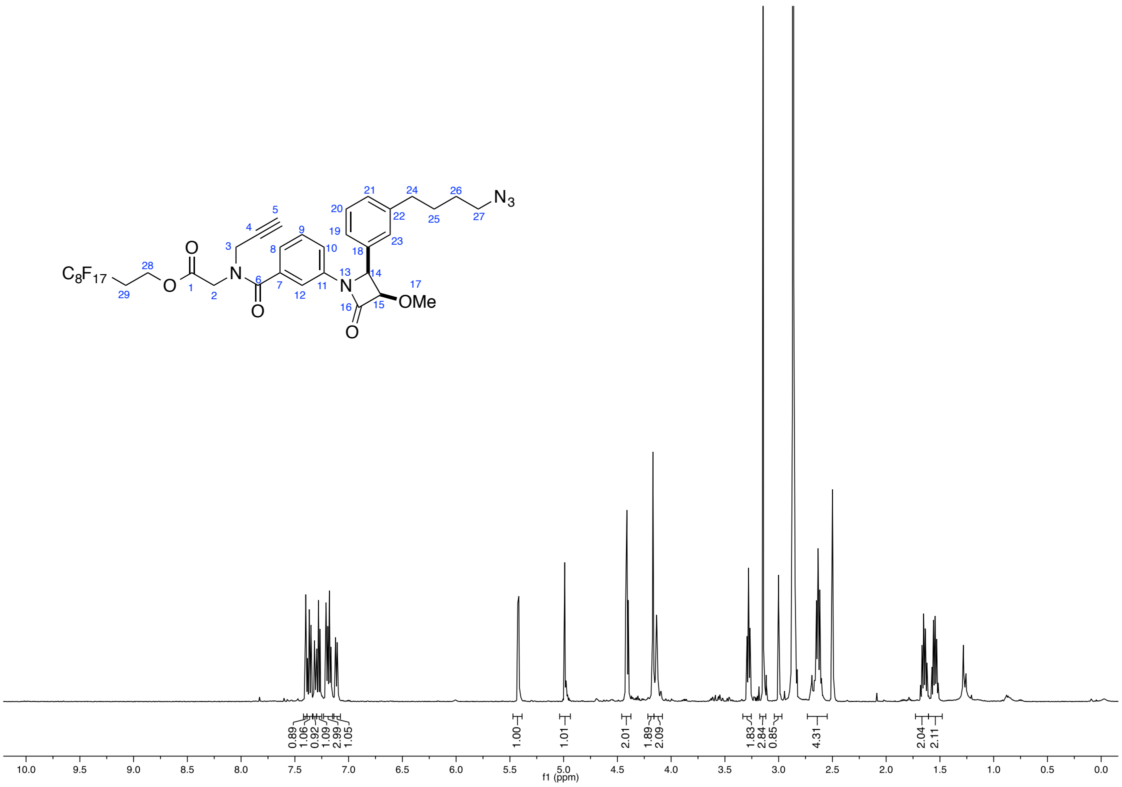

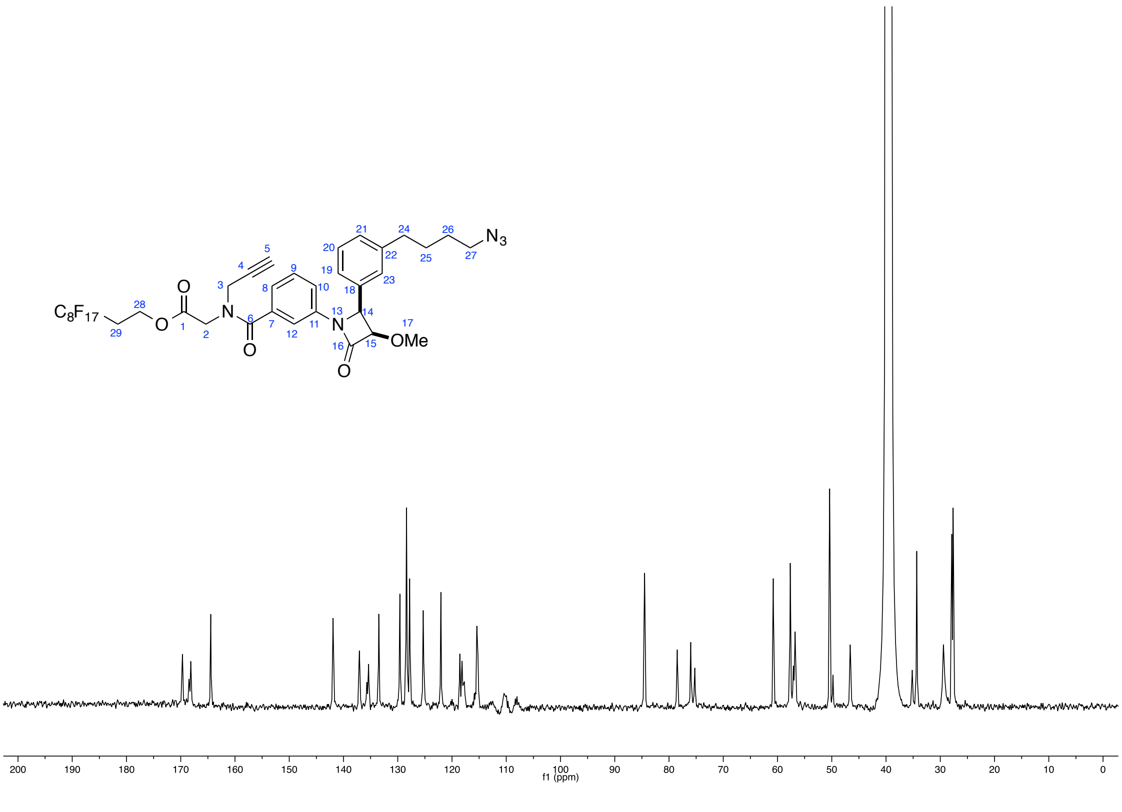

18b

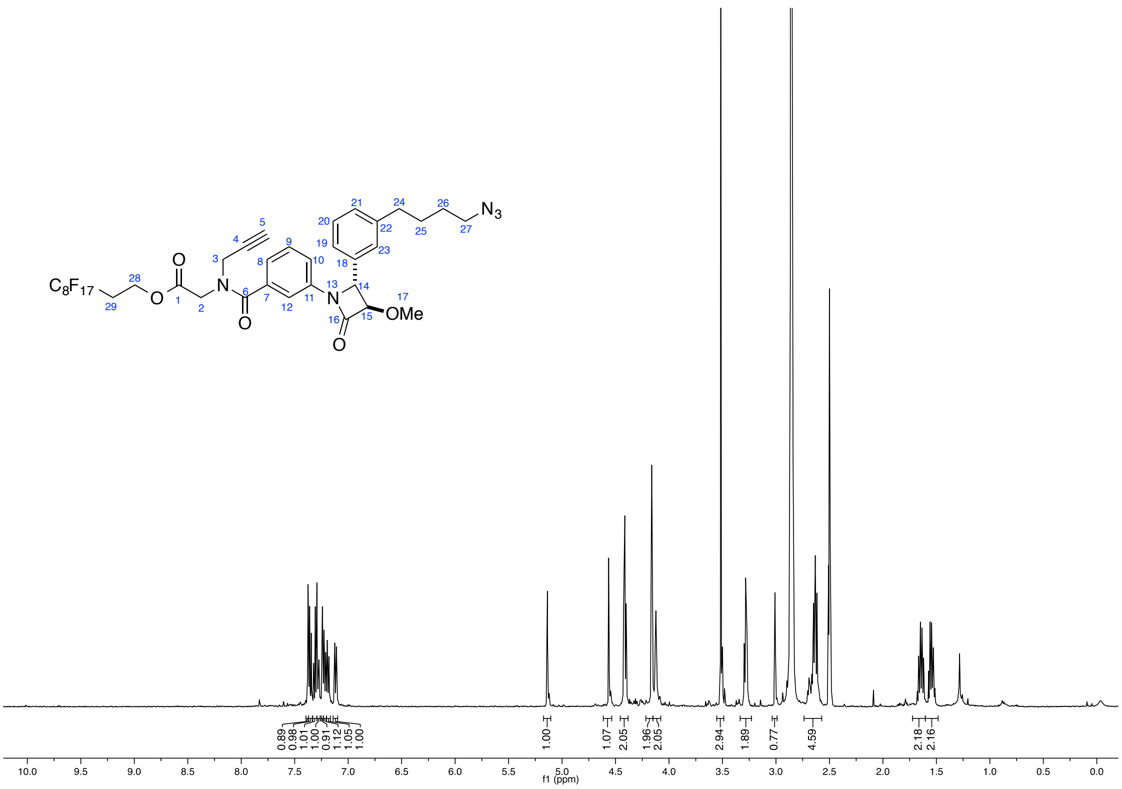
C8F17
OMe
N3
1.00
1.07
2.05
1.96
2.05
2.94
1.89
0.77
4.59
2.18
2.16
f1 (ppm)

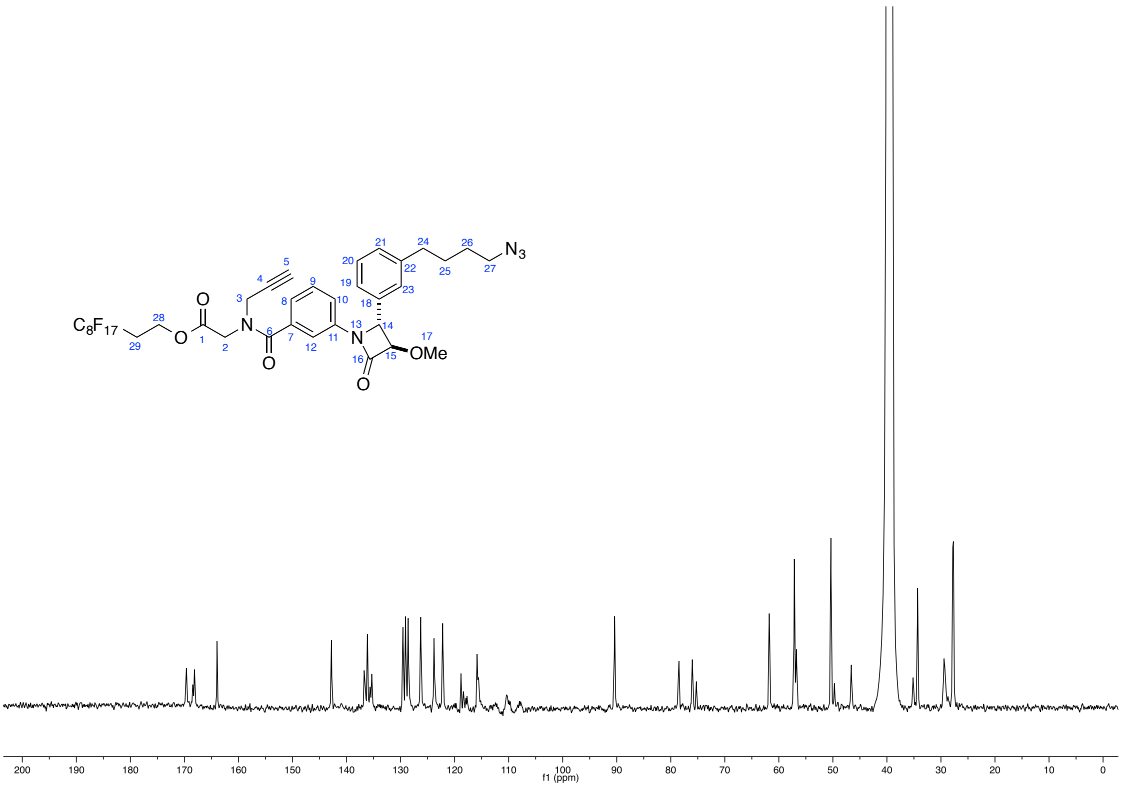
C8F17
OMe
N3
f1 (ppm)

19

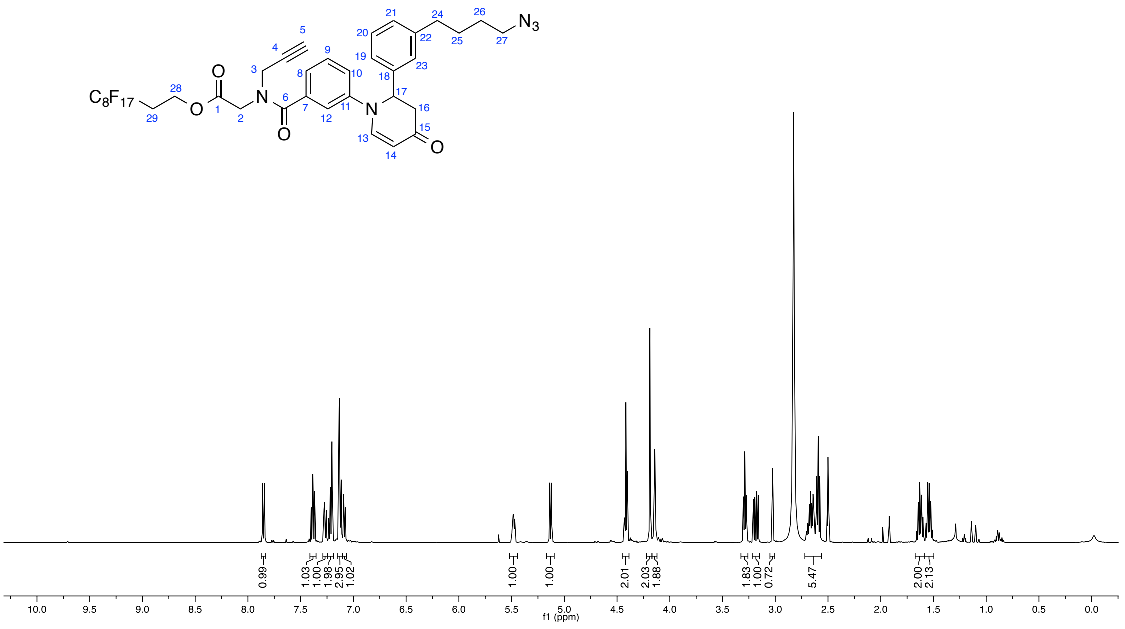

C8F17
N3
0.99
1.03
1.00
1.98
2.95
1.02
1.00
1.00
2.01
2.03
1.88
1.83
1.00
0.72
5.47
2.00
2.13
f1 (ppm)

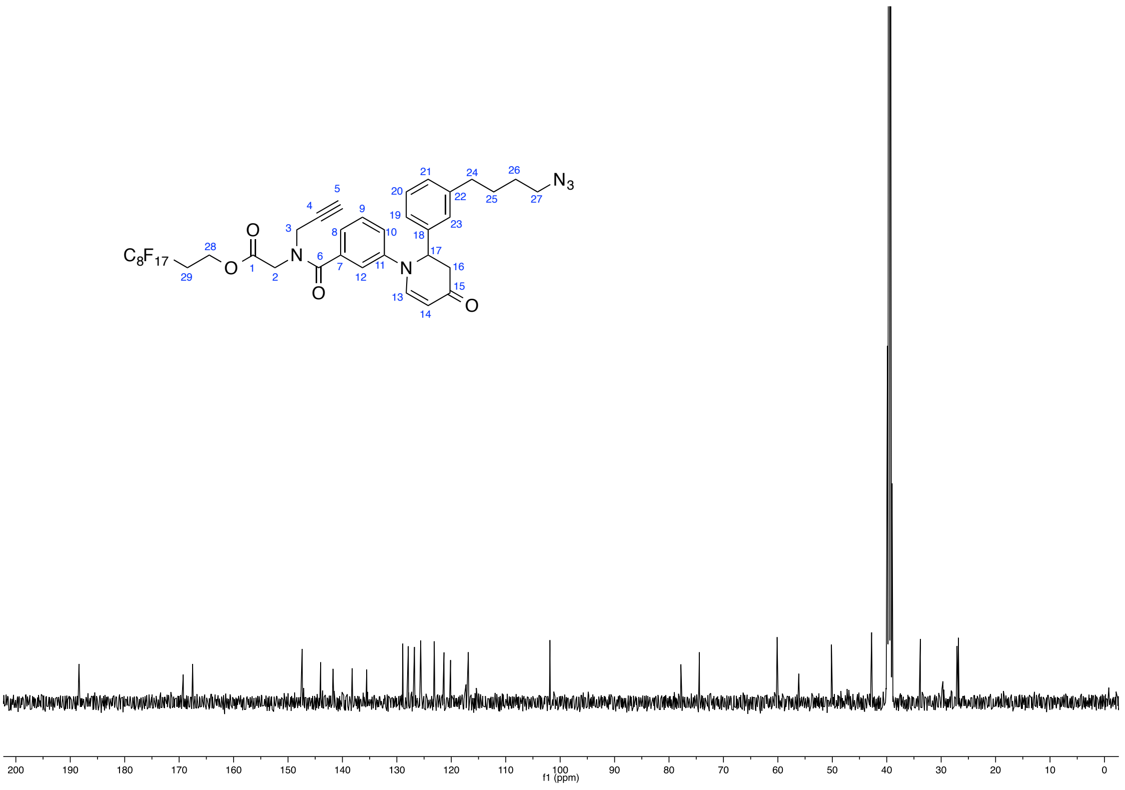

C8F17
N3
f1 (ppm)

20

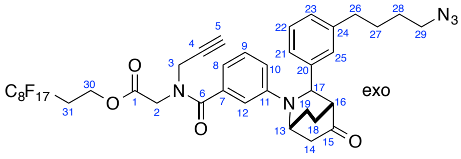

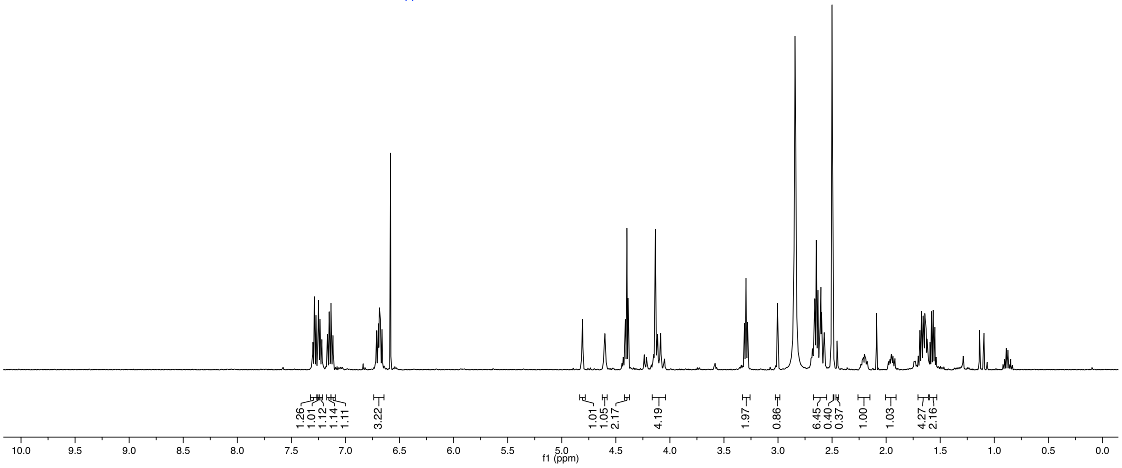

21a

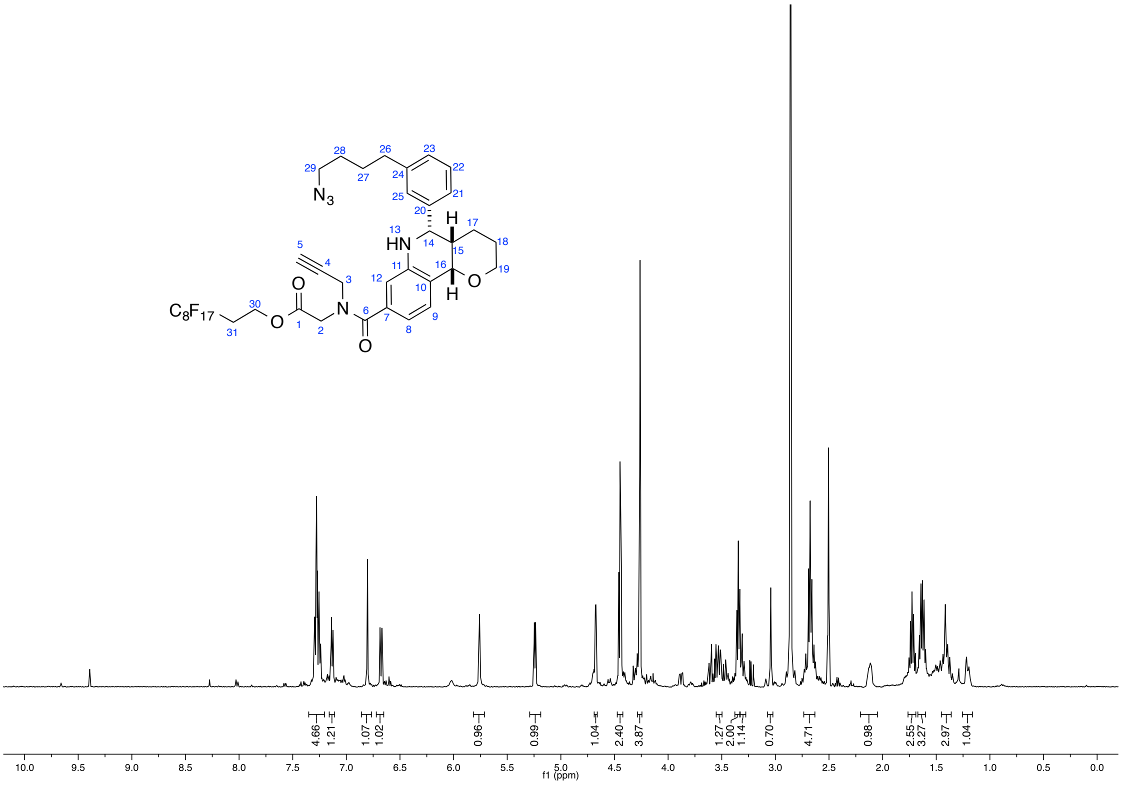

N3
HN
H
O
H
C8F17
O
O
N
O
4.66
1.21
1.07
1.02
0.96
0.99
1.04
2.40
3.87
1.27
2.00
1.14
0.70
4.71
0.98
2.55
3.27
2.97
1.04
f1 (ppm)

21d

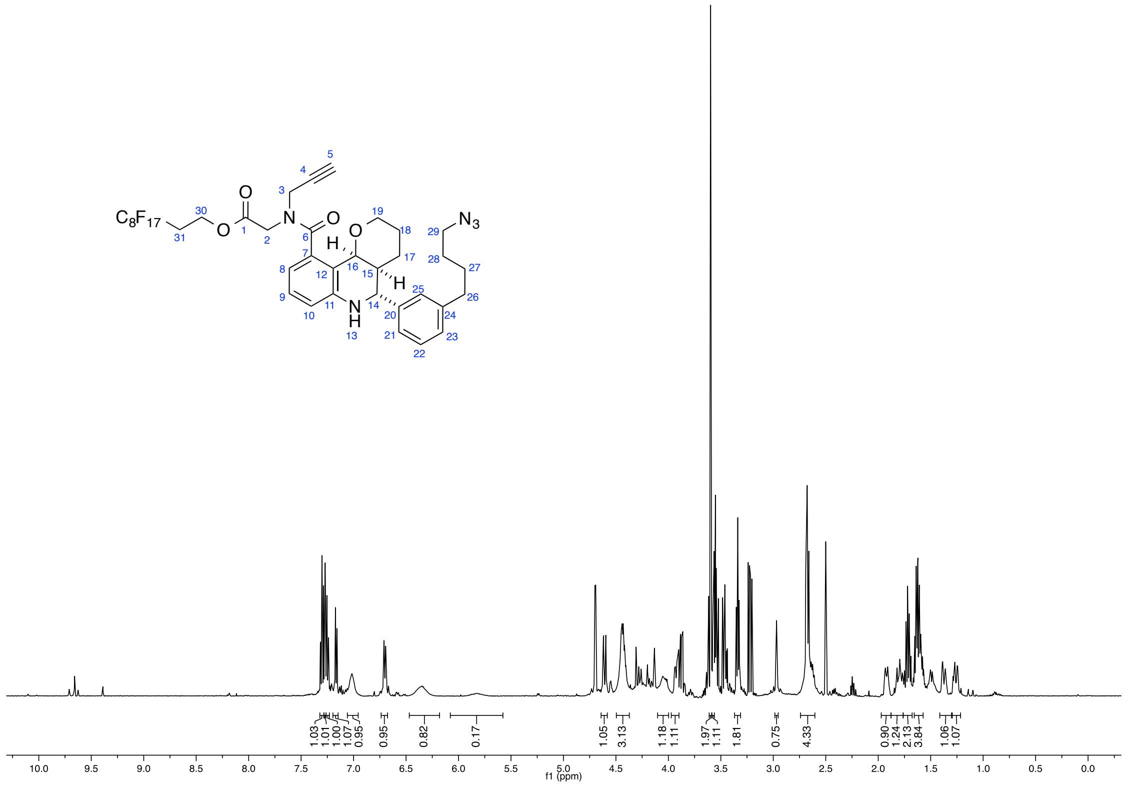
C8F17
N3
f1 (ppm)
10.0 9.5 9.0 8.5 8.0 7.5 7.0 6.5 6.0 5.5 5.0 4.5 4.0 3.5 3.0 2.5 2.0 1.5 1.0 0.5 0.0

24a

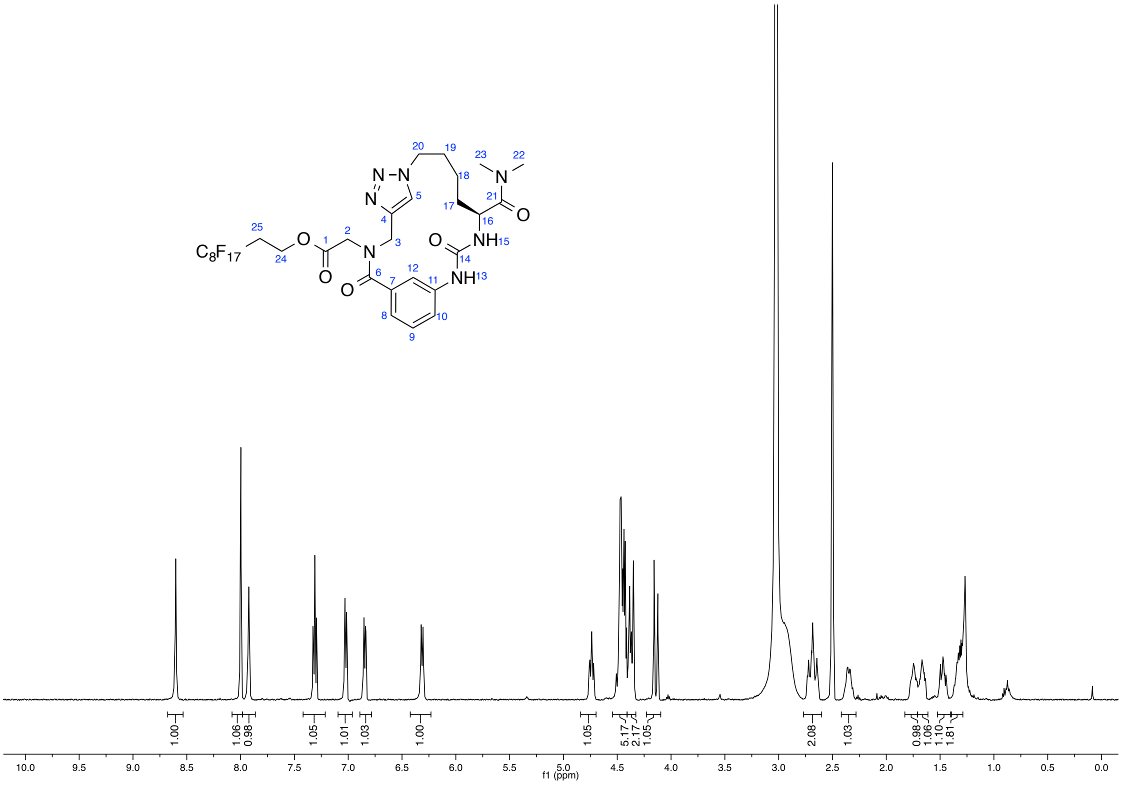

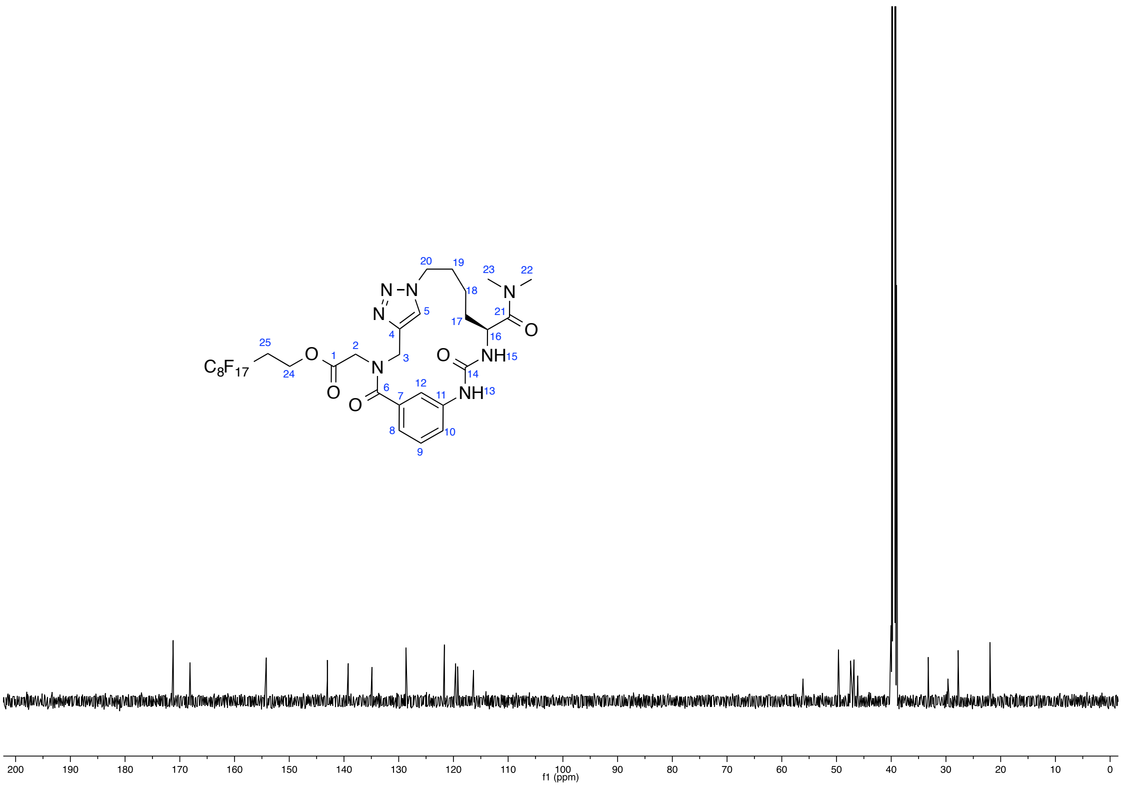

24b

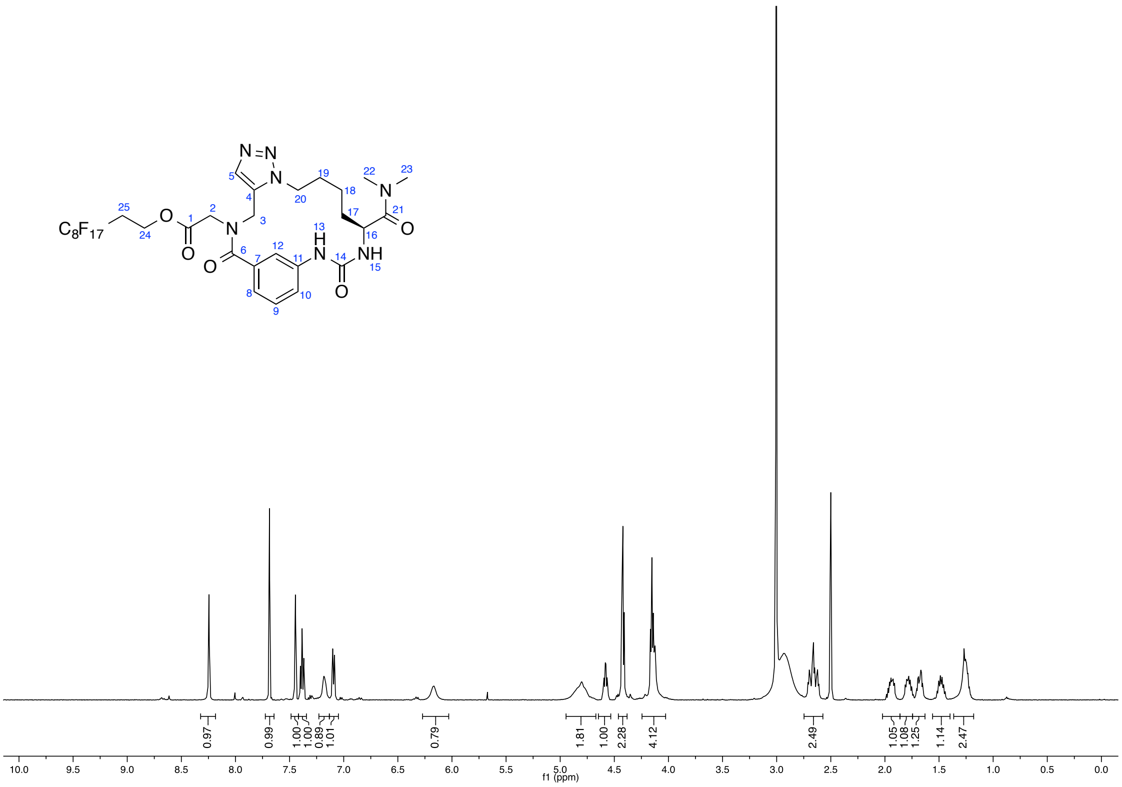
C8F17
f1 (ppm)
0.97
0.99
1.00
1.00
0.89
1.01
0.79
1.81
1.00
2.28
4.12
2.49
1.05
1.08
1.25
1.14
2.47

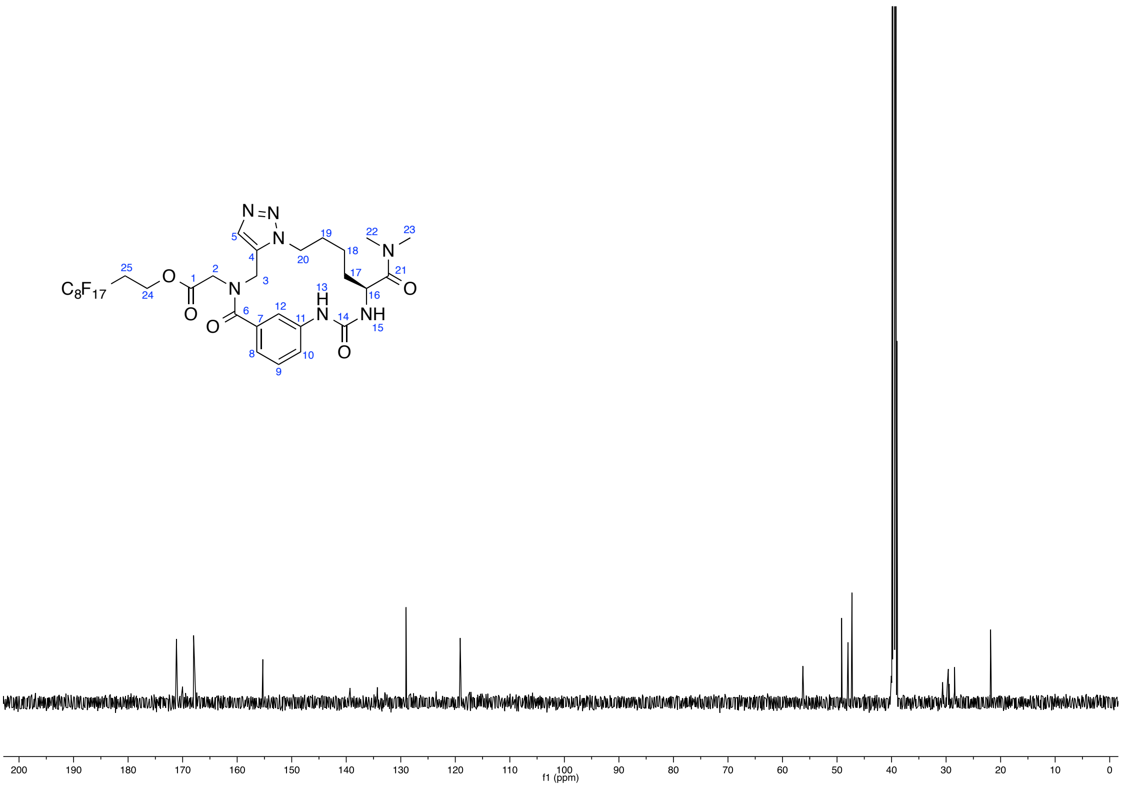
C8F17
f1 (ppm)

24c

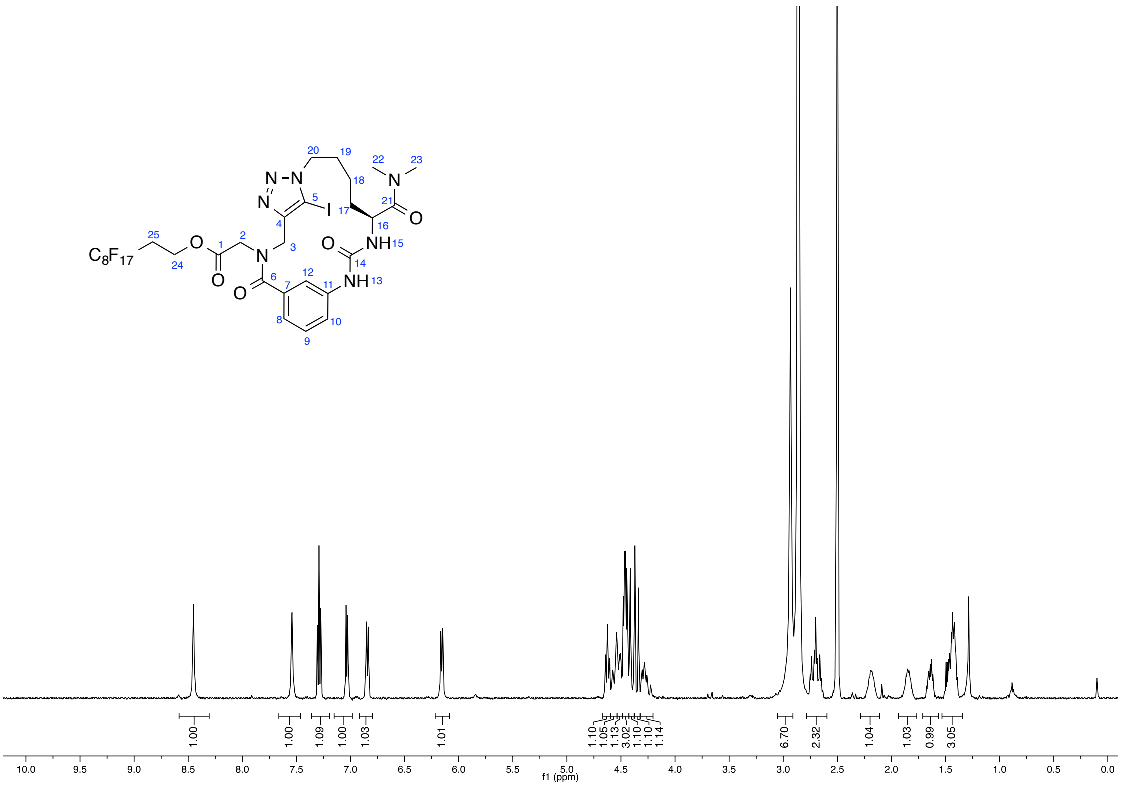

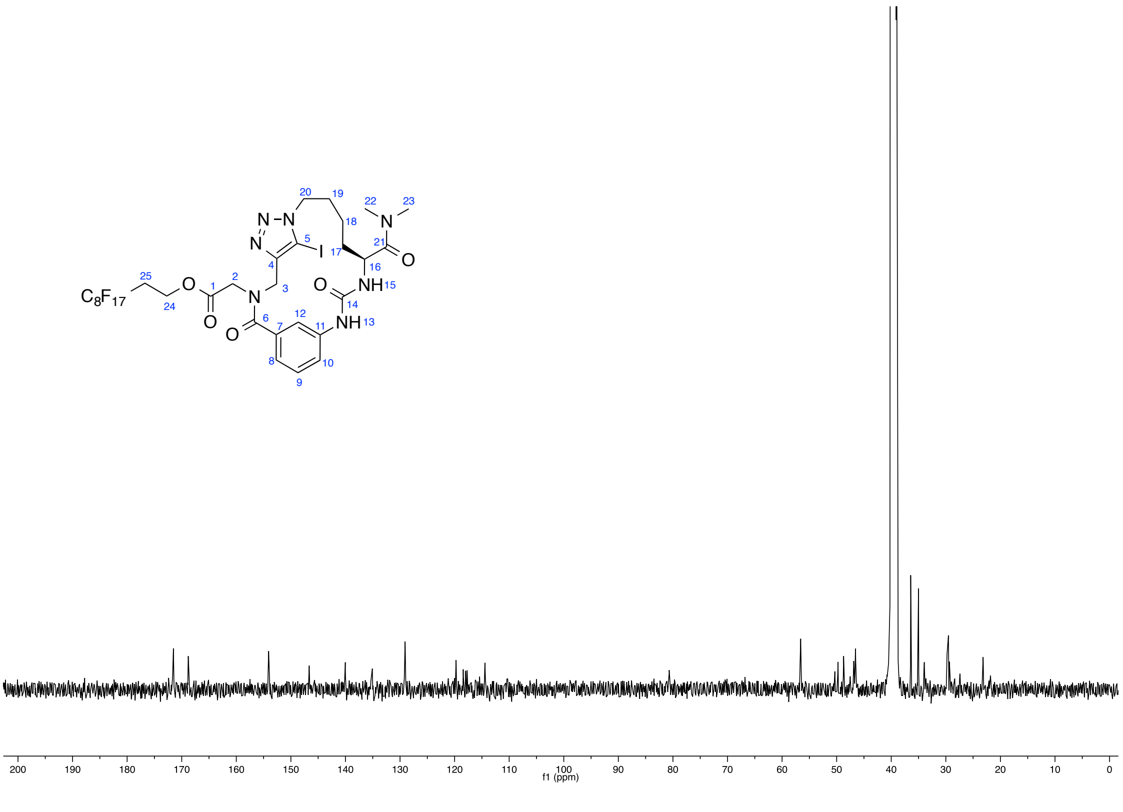

26a

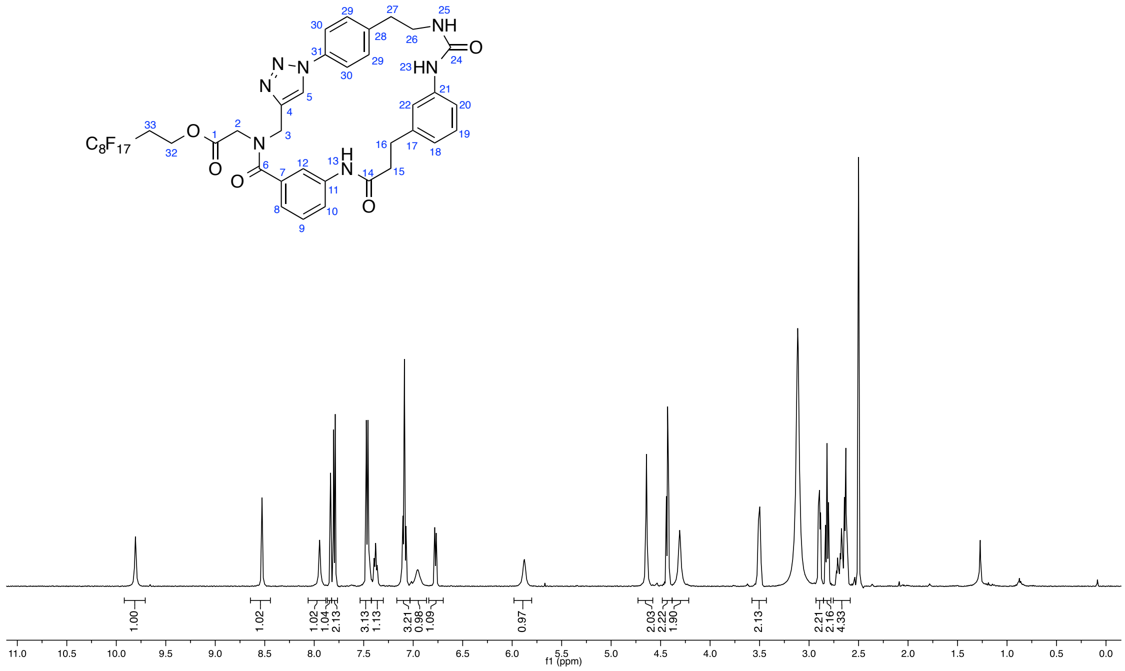

C_8F_{17}
1.00
1.02
1.02
1.04
2.13
3.13
1.13
3.21
0.98
1.09
0.97
2.03
2.22
1.90
2.13
2.21
2.16
4.33
f1 (ppm)

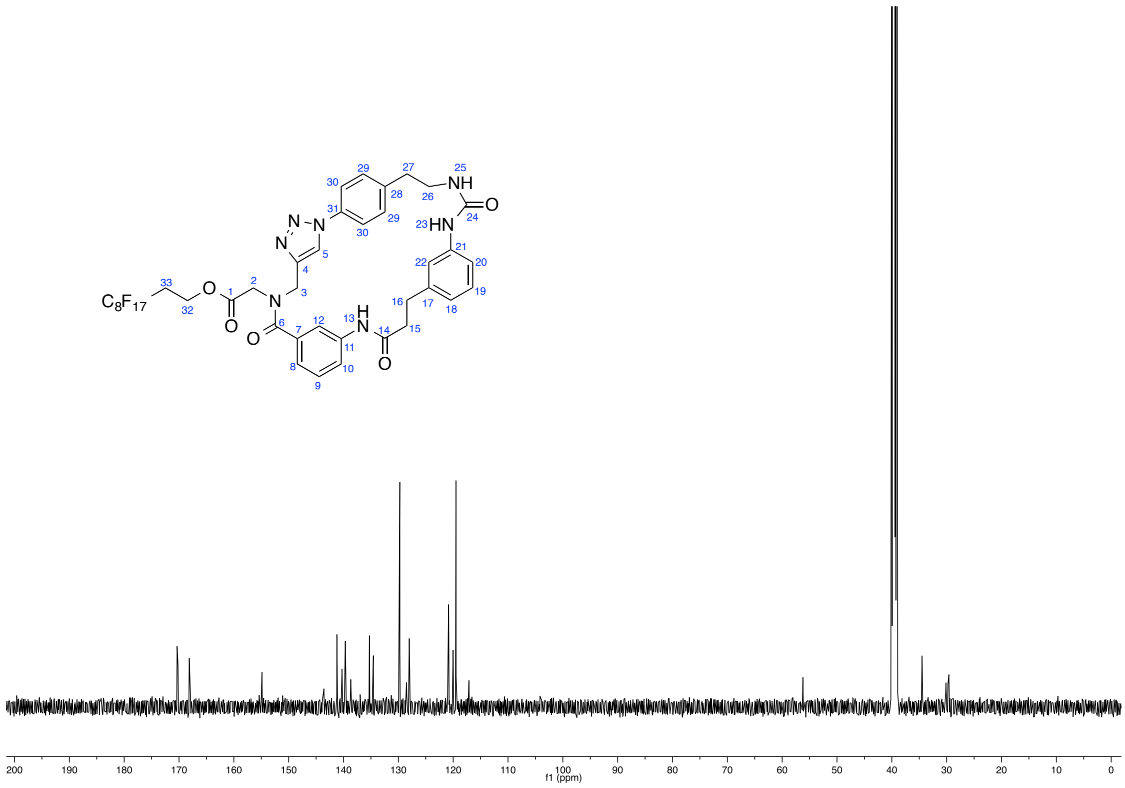

C_8F_{17}
f1 (ppm)

26b

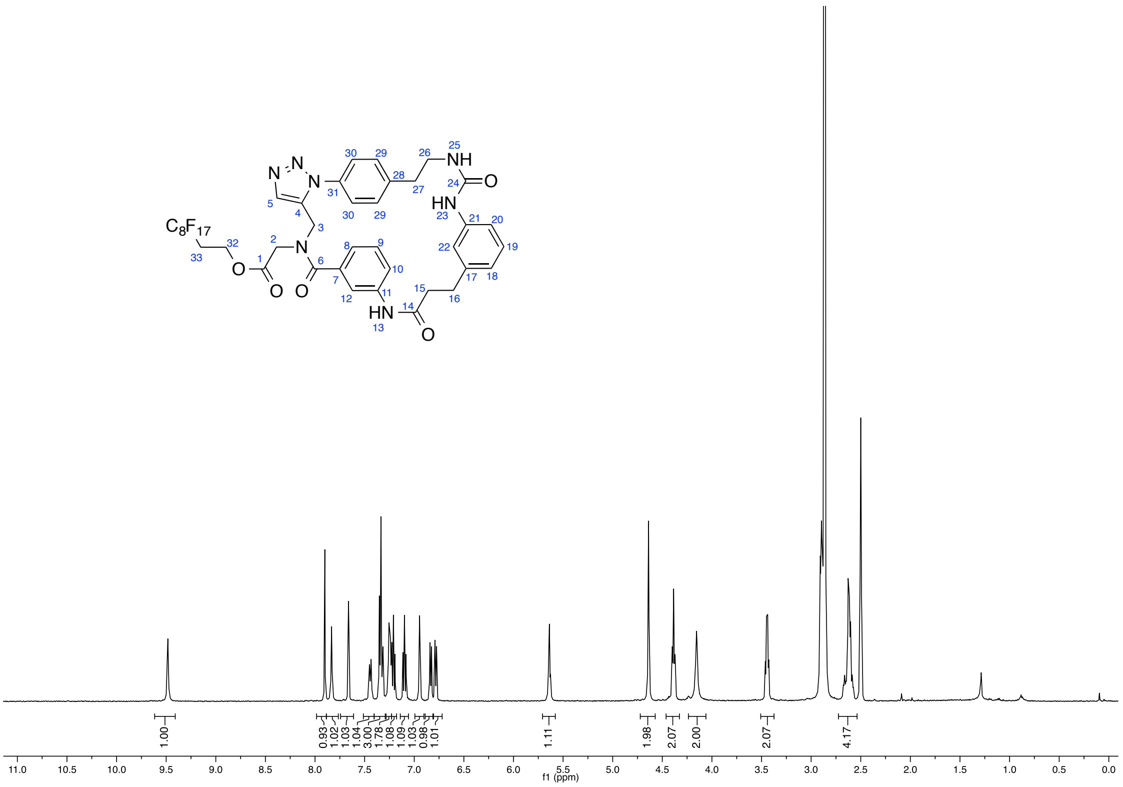

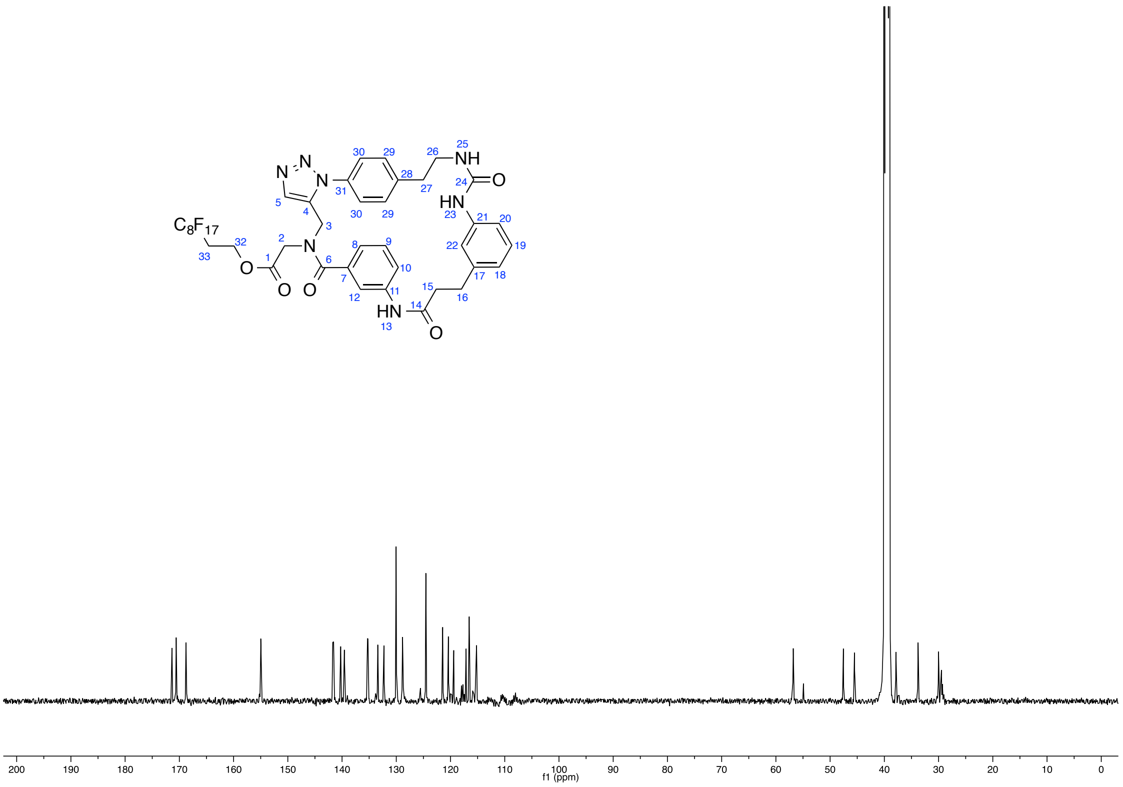

27a

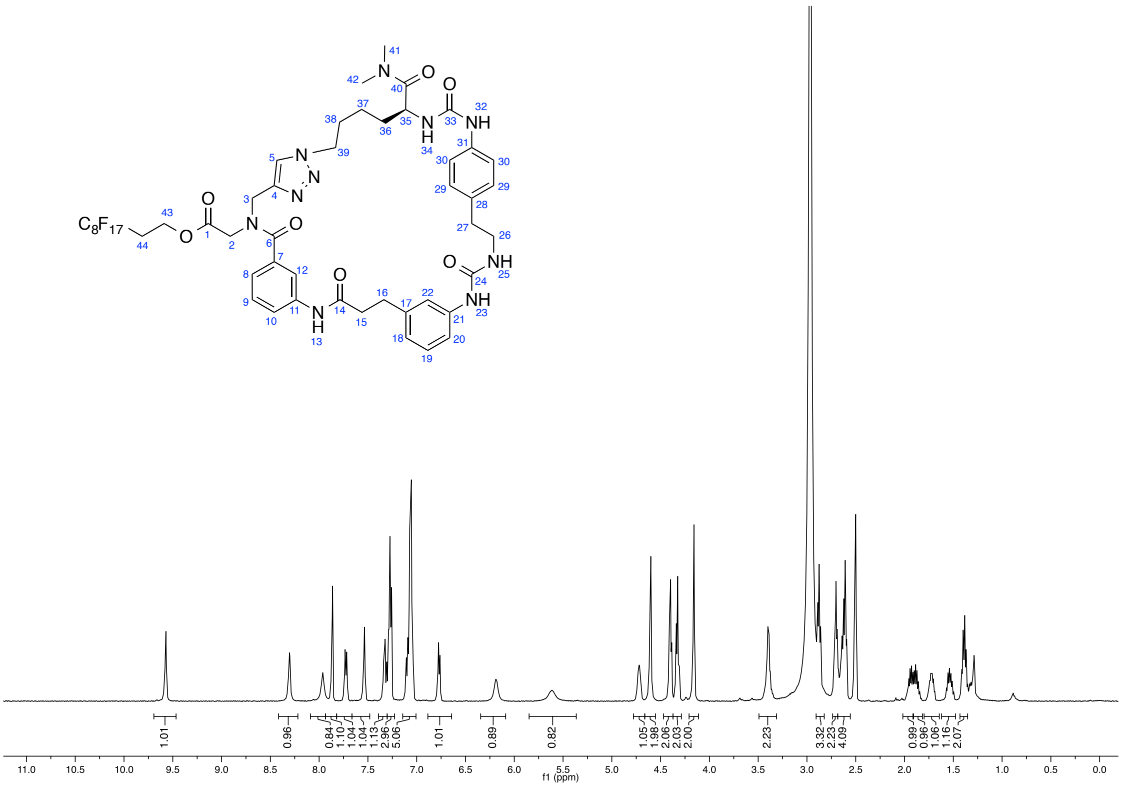

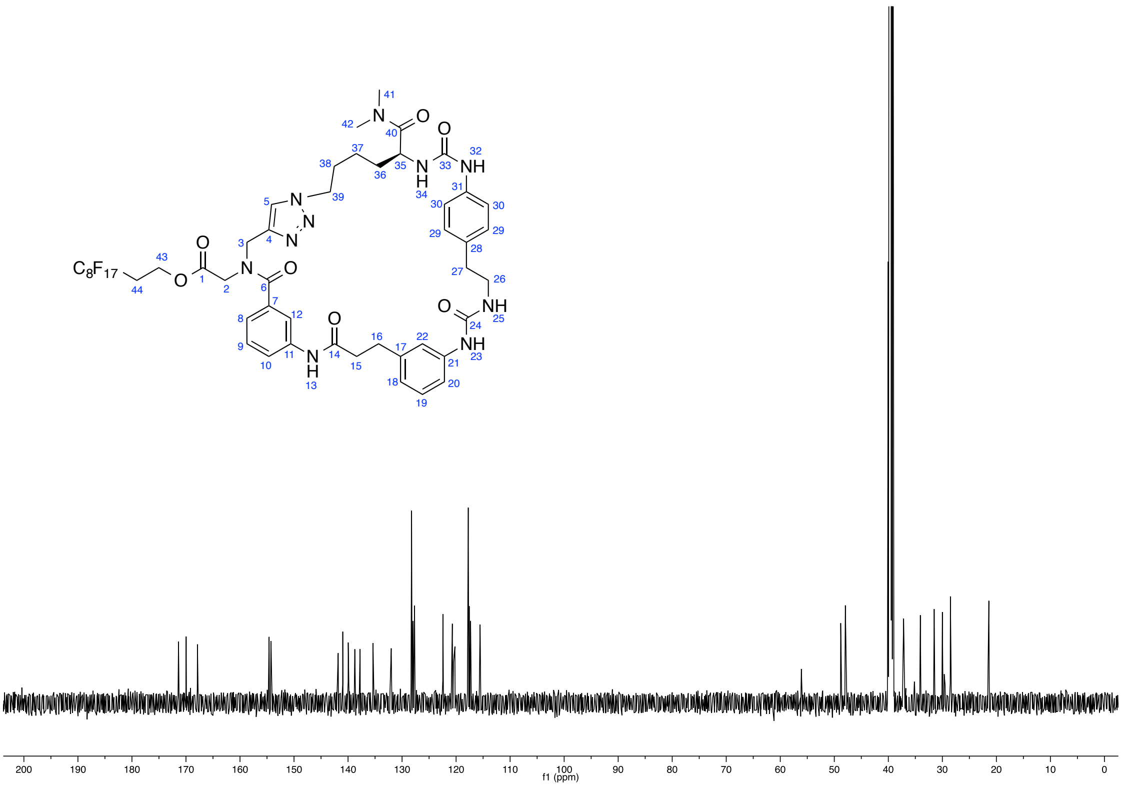

27b

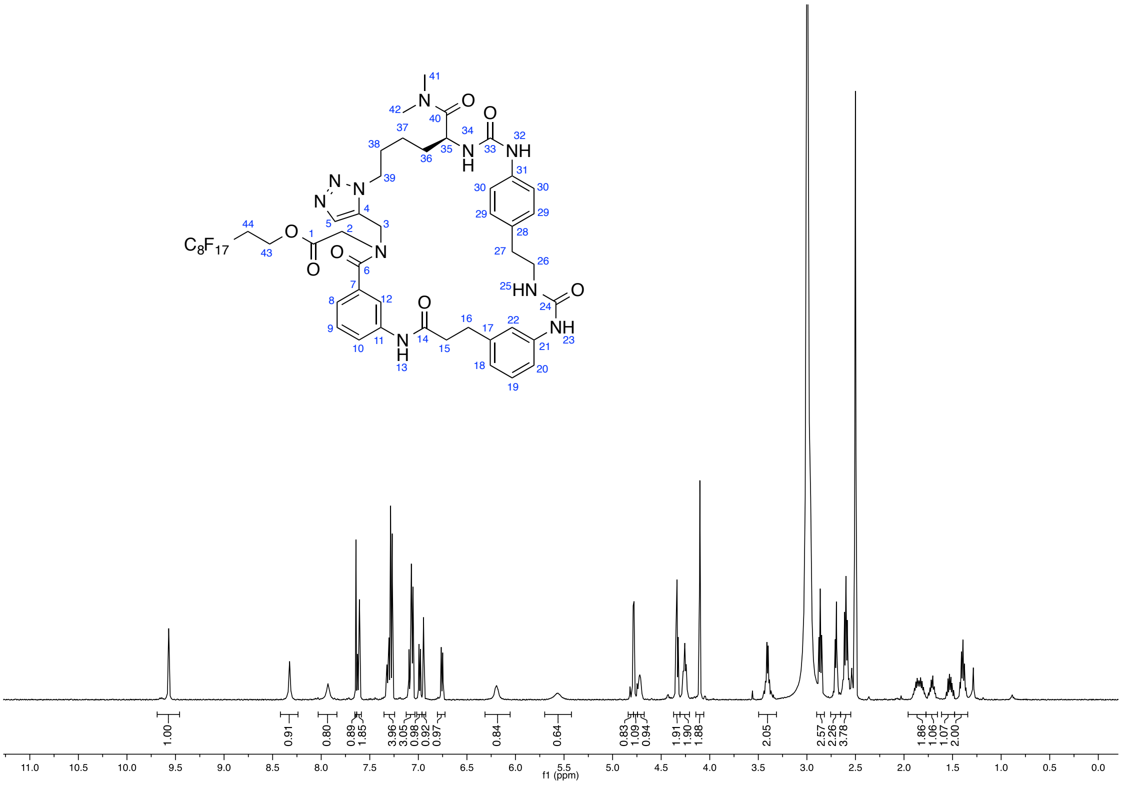

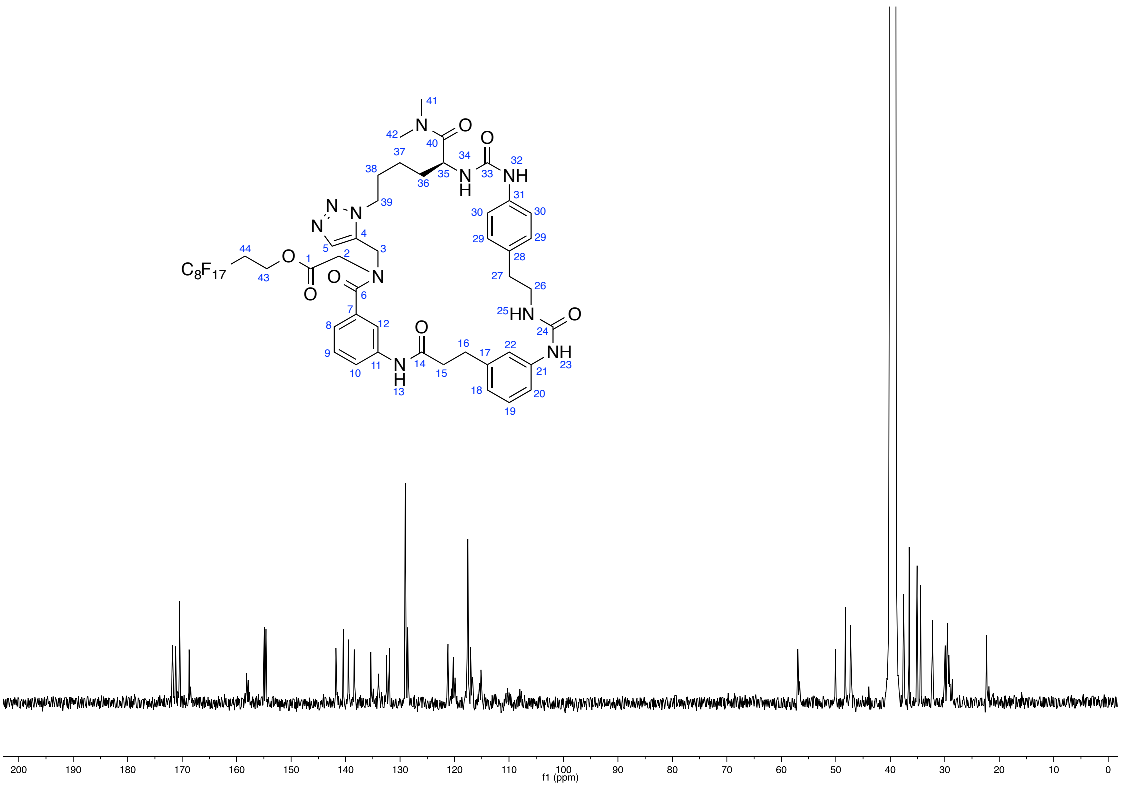

28a

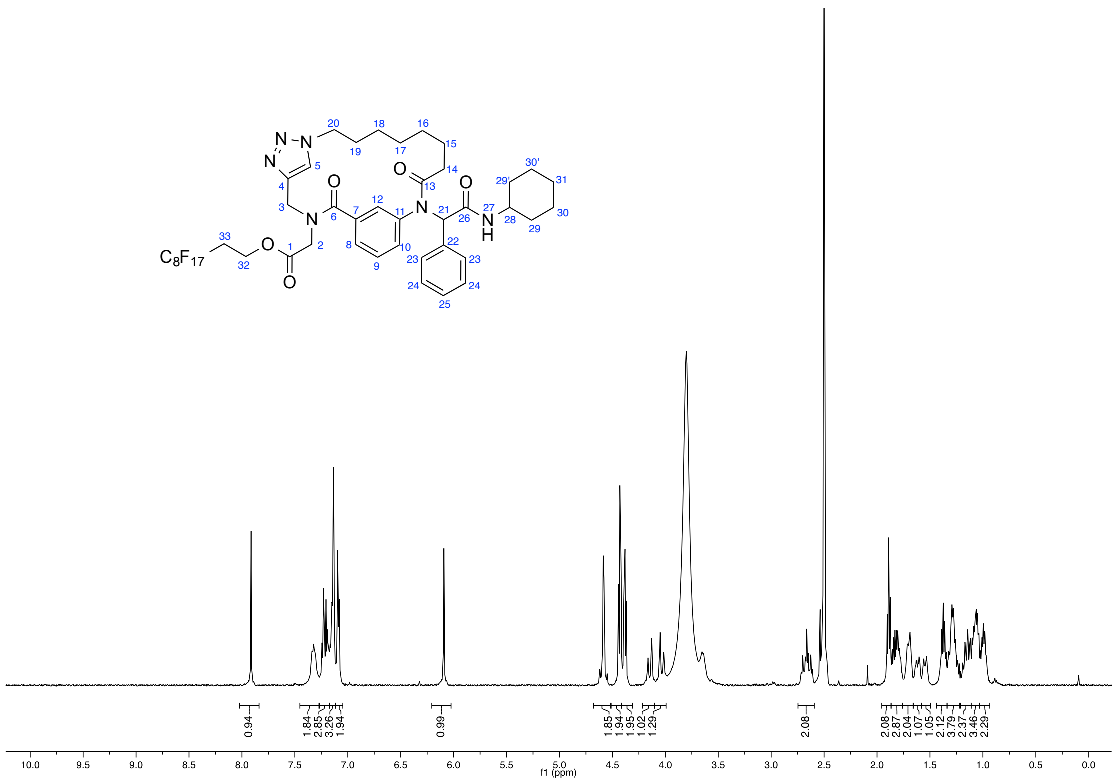

C8F17
0.94
1.84
2.85
3.26
1.94
0.99
1.85
1.94
1.95
1.02
1.29
2.08
2.08
2.87
2.04
1.07
1.05
2.12
3.79
2.37
3.46
2.29
f1 (ppm)

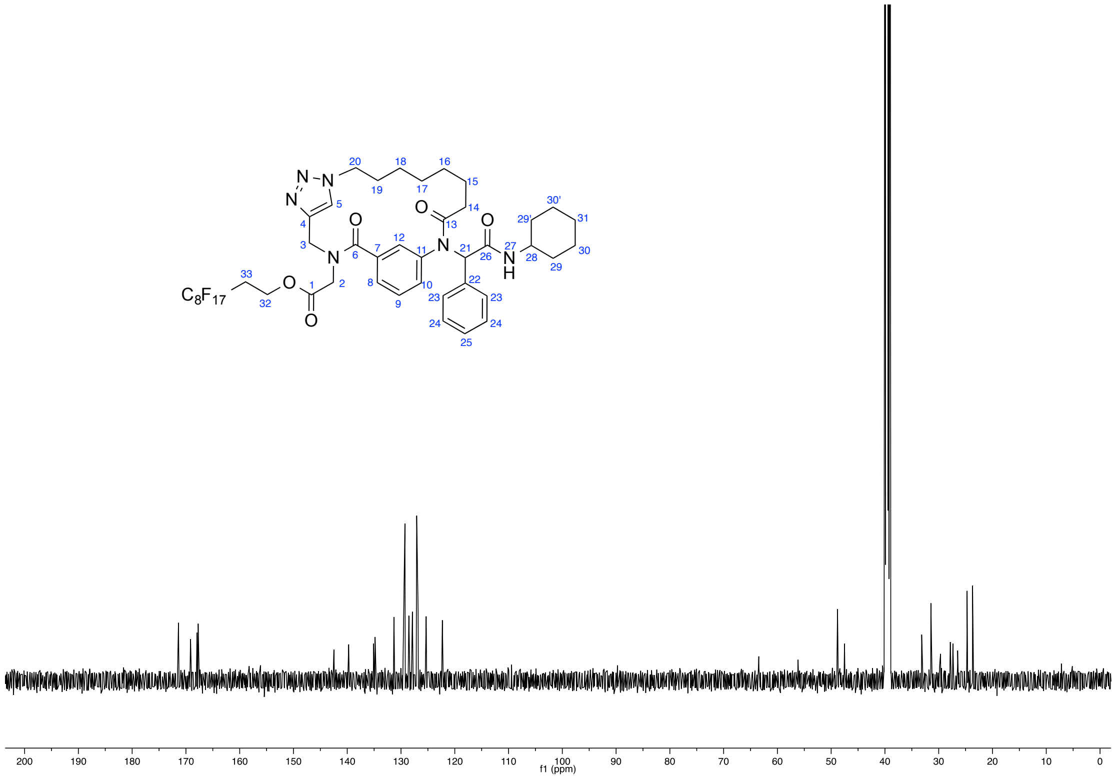

C8F17
f1 (ppm)

28b

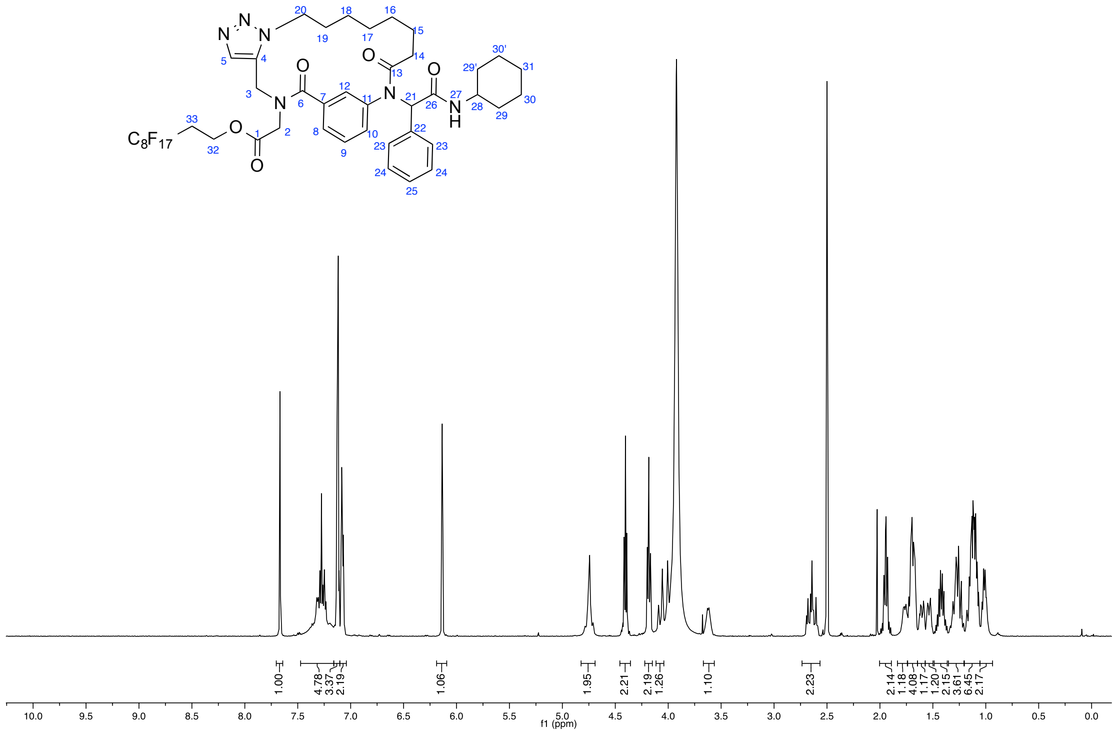

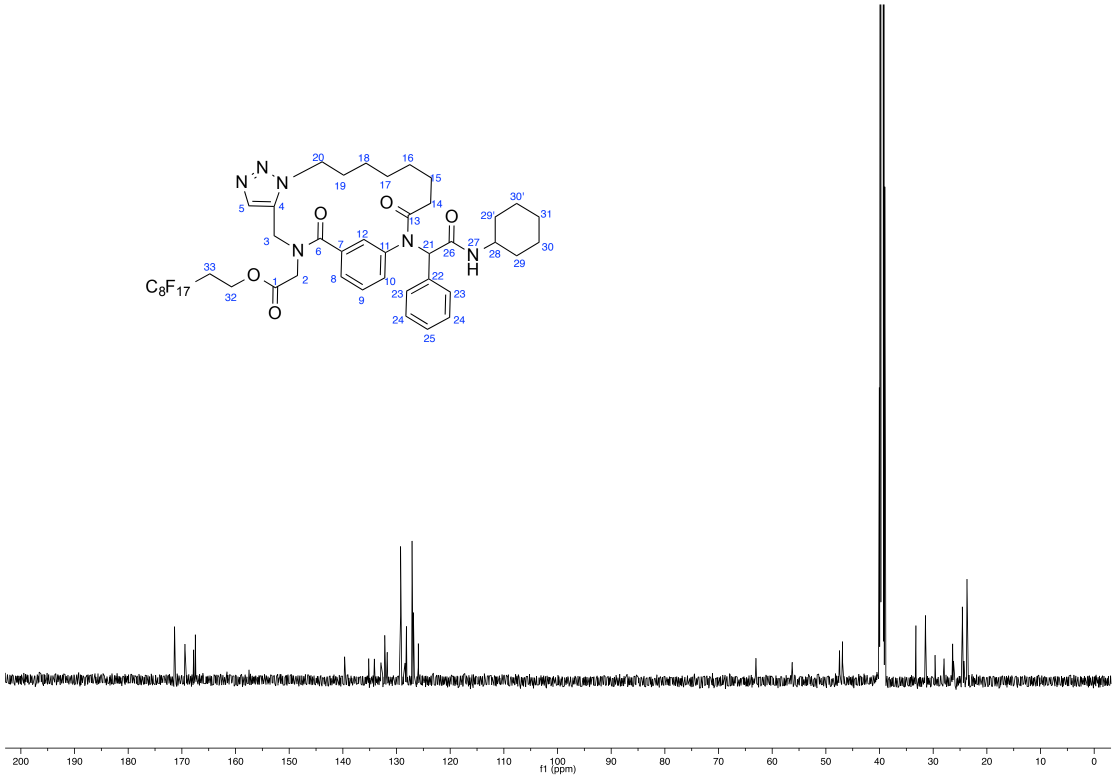

29a

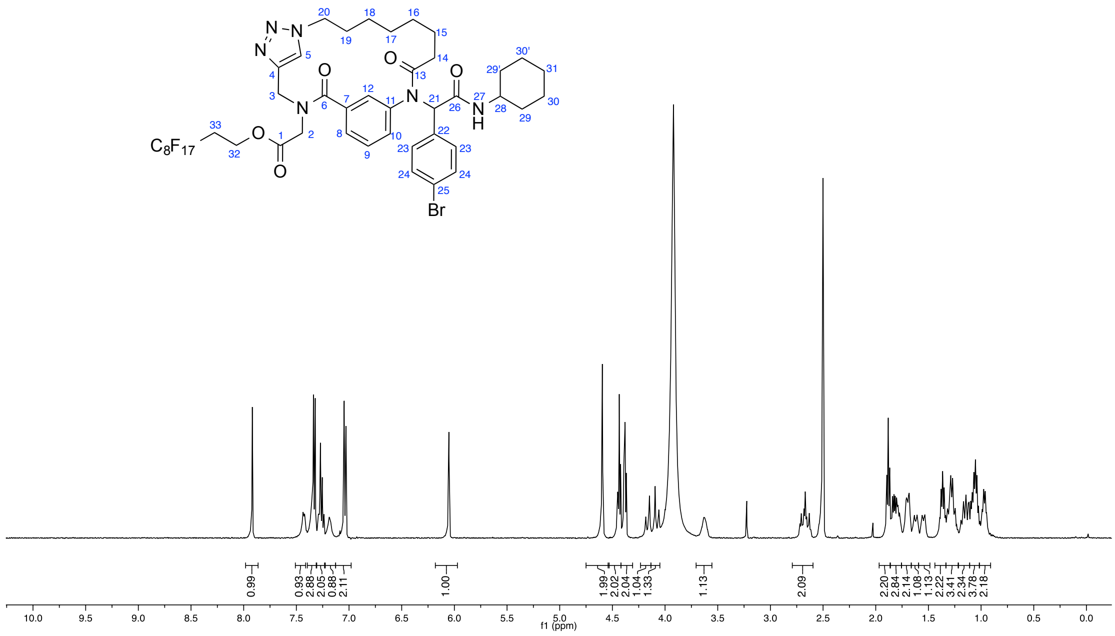
C8F17
Br
0.99
0.93
2.88
2.05
0.88
2.11
1.00
1.99
2.02
2.04
1.04
1.33
1.13
2.09
2.20
2.84
2.14
1.08
1.13
2.22
3.41
2.34
3.78
2.18
f1 (ppm)

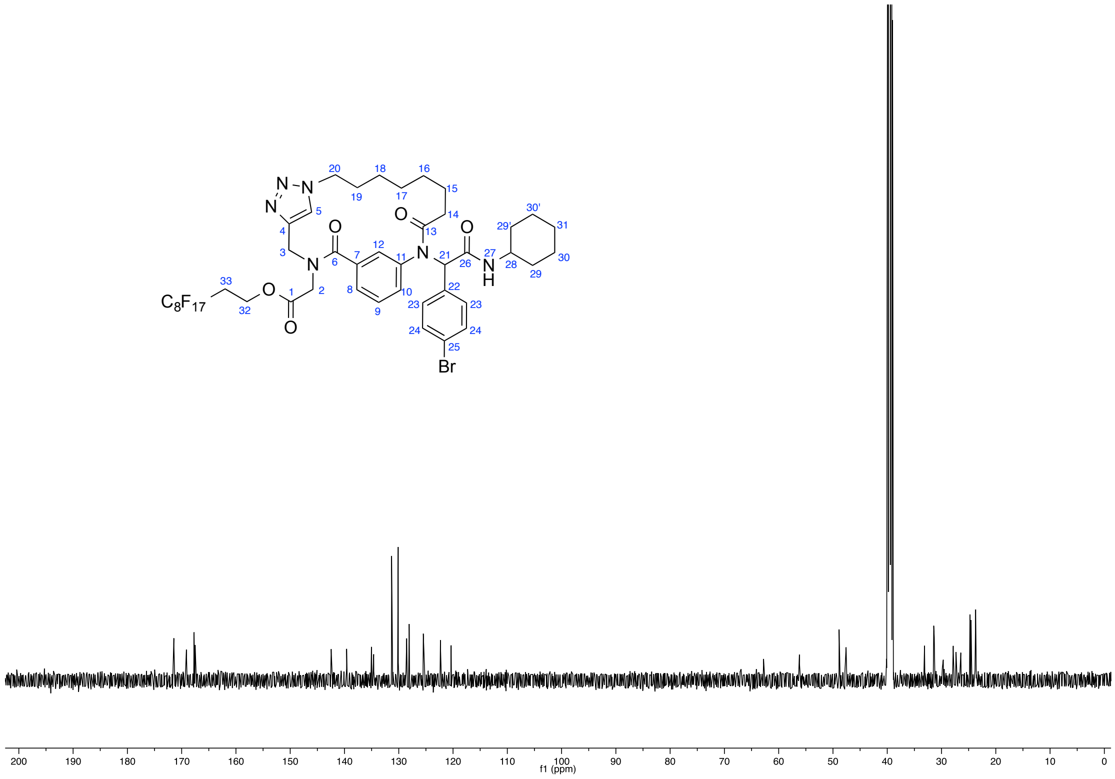
C8F17
Br
f1 (ppm)

29b

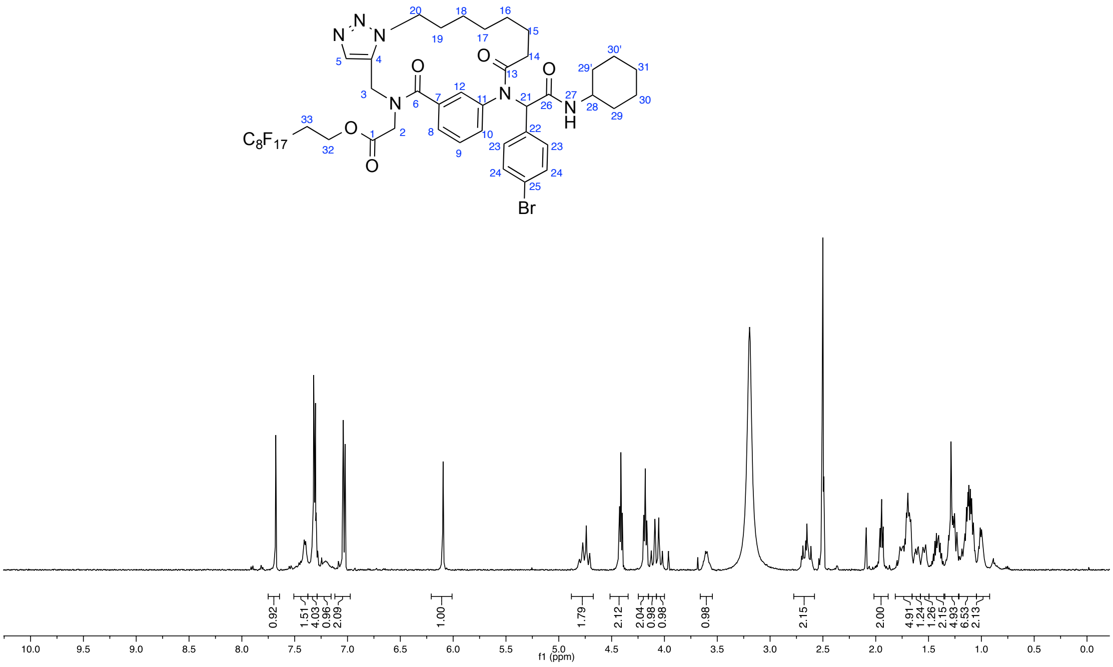

0.92
1.51
4.03
0.96
2.09
1.00
1.79
2.12
2.04
0.98
0.98
0.98
2.15
2.00
4.91
1.24
1.26
1.26
2.15
4.93
6.53
2.13
f1 (ppm)

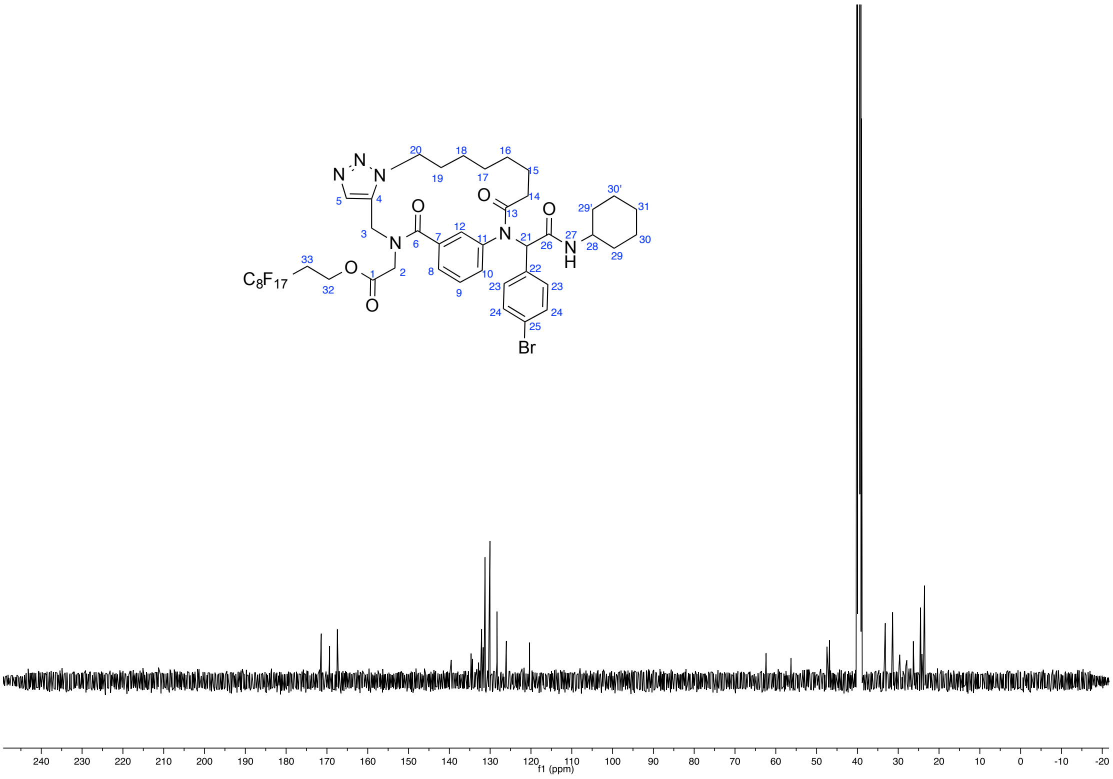

f1 (ppm)

30a

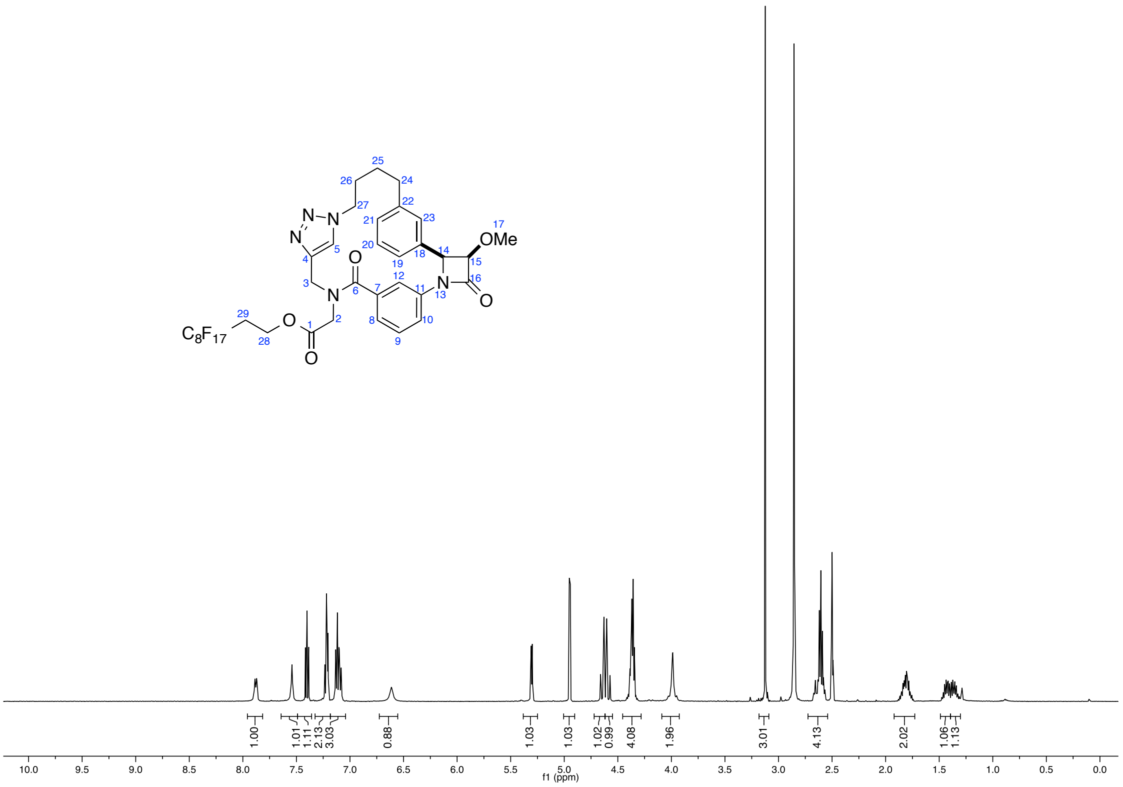
C8F17
OMe
1.00
1.01
1.11
2.13
3.03
0.88
1.03
1.03
1.02
0.99
4.08
1.96
3.01
4.13
2.02
1.06
1.13
f1 (ppm)

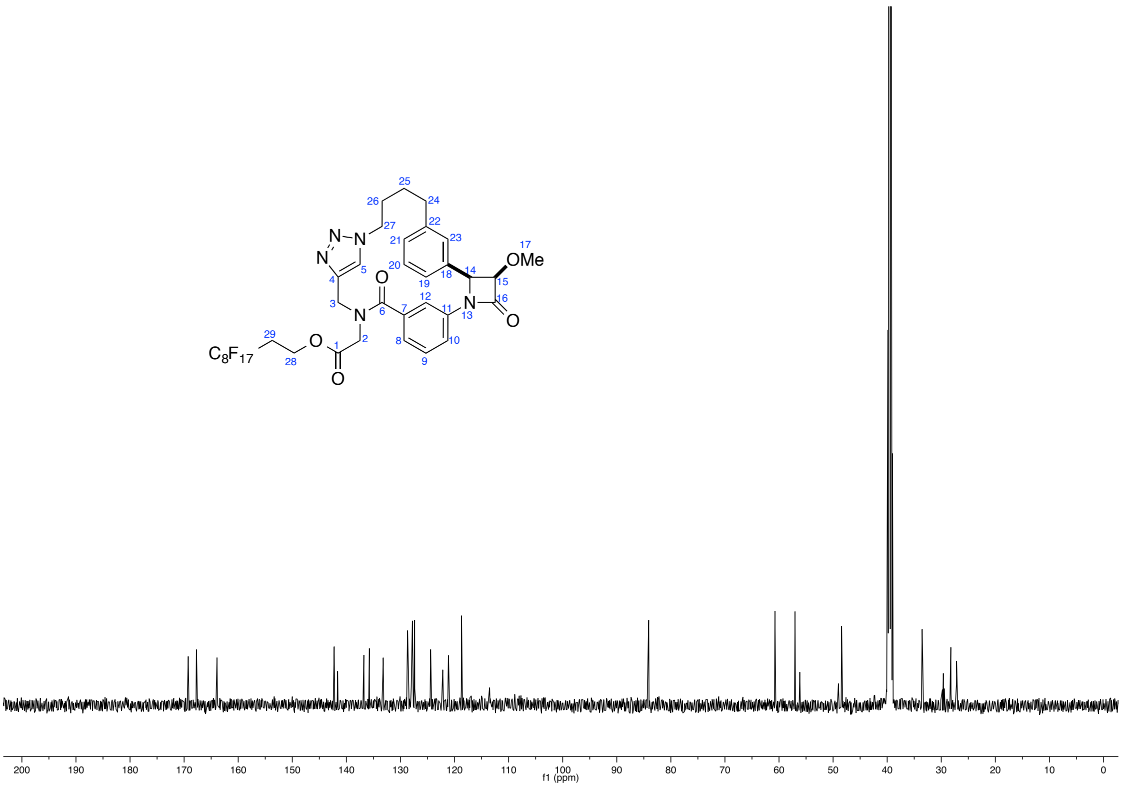
C8F17
OMe
f1 (ppm)

30b

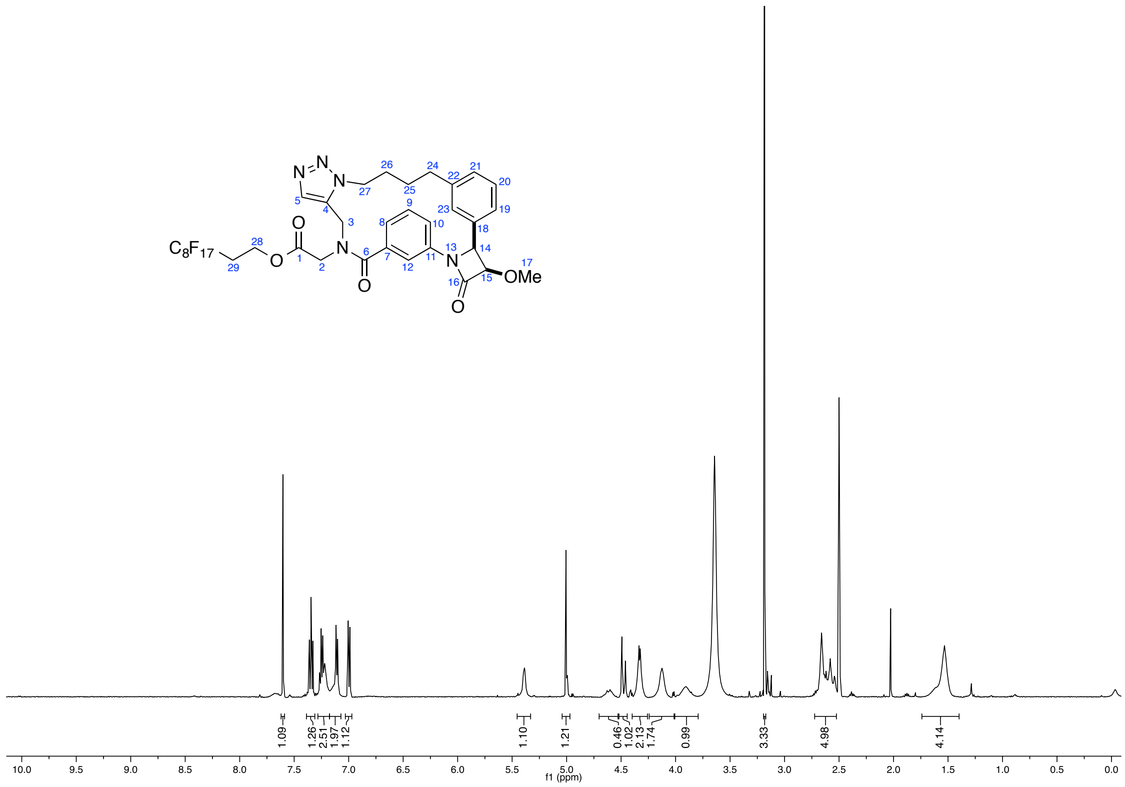
C8F17
OMe
1.09
1.26
2.51
1.97
1.12
1.10
1.21
0.46
1.02
2.13
1.74
0.99
3.33
4.98
4.14
f1 (ppm)

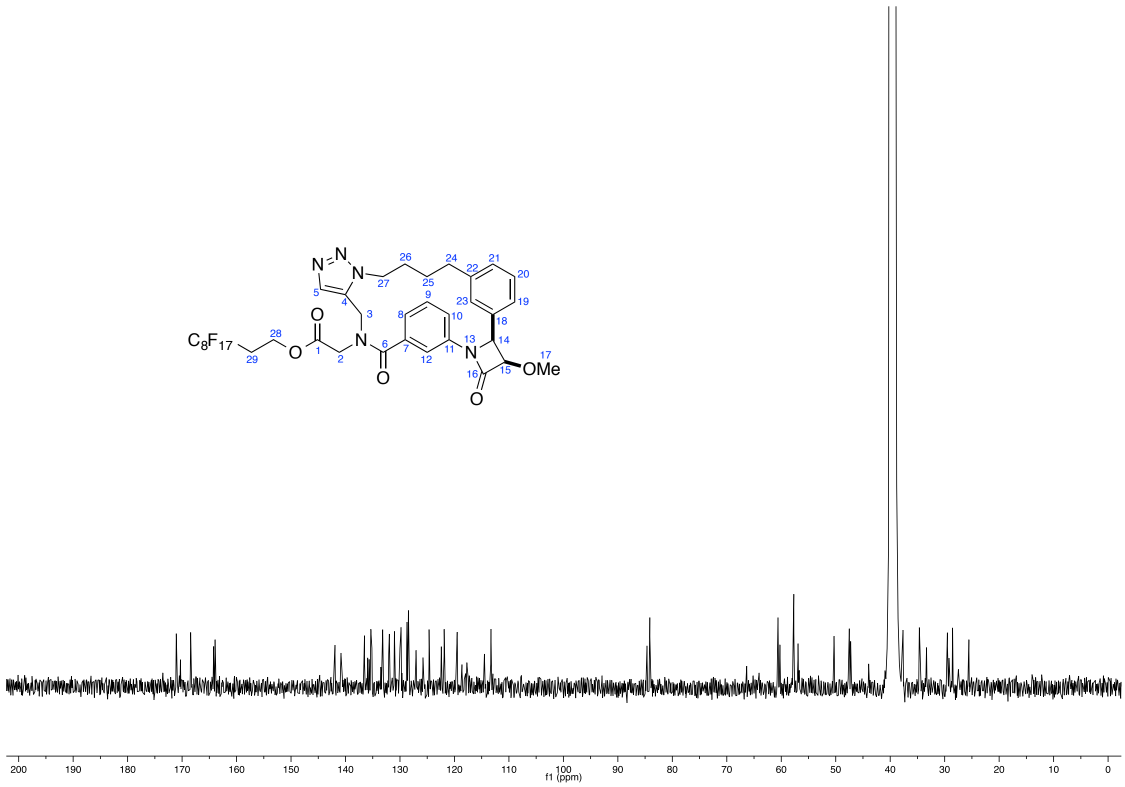
C8F17
OMe
f1 (ppm)

30c

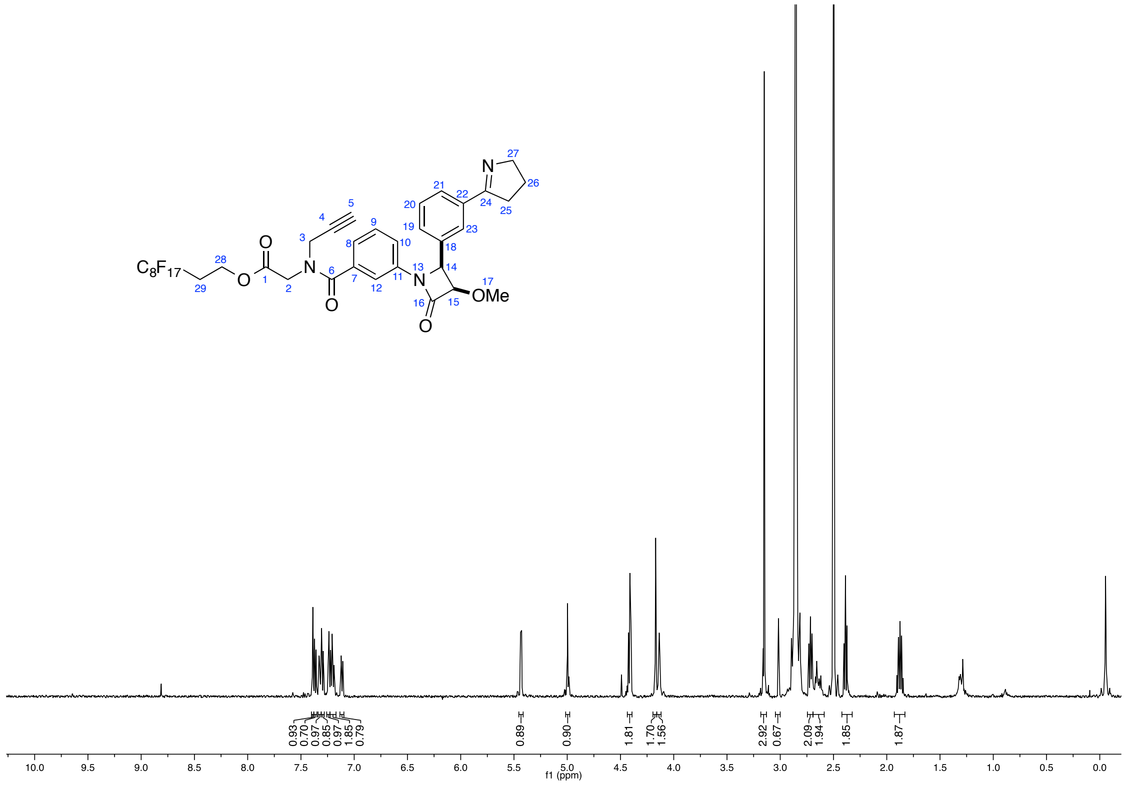

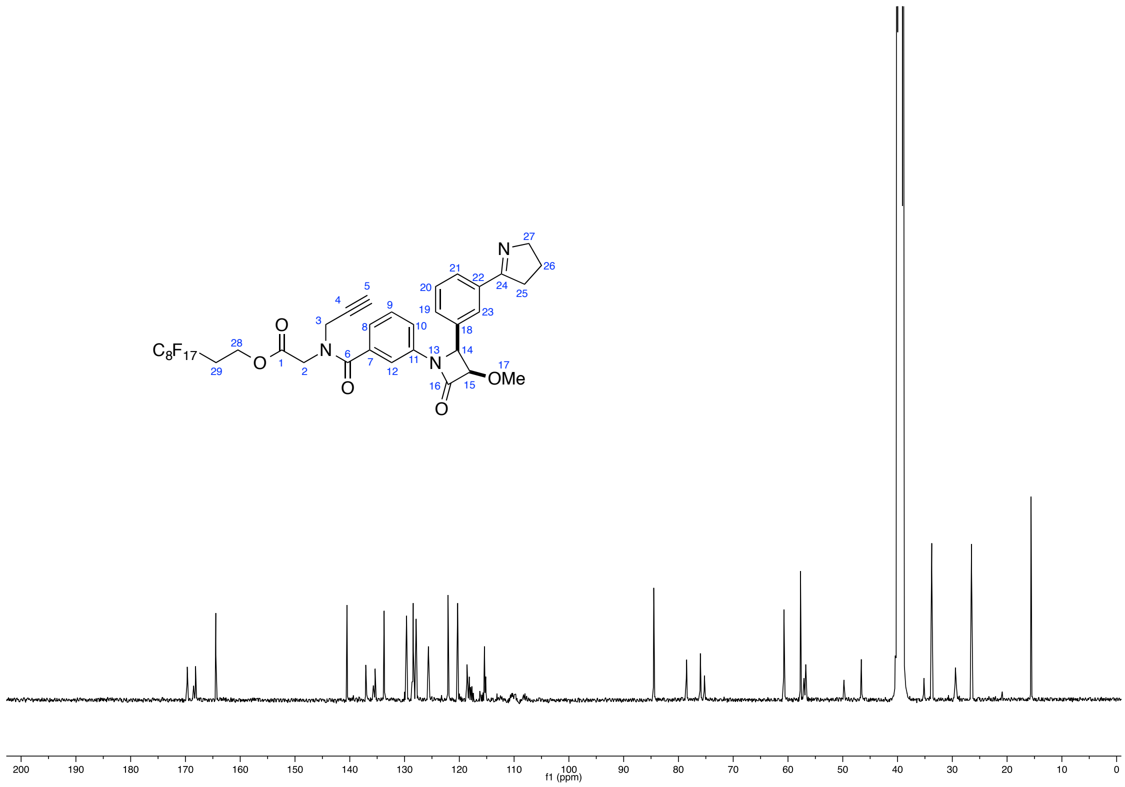

31a

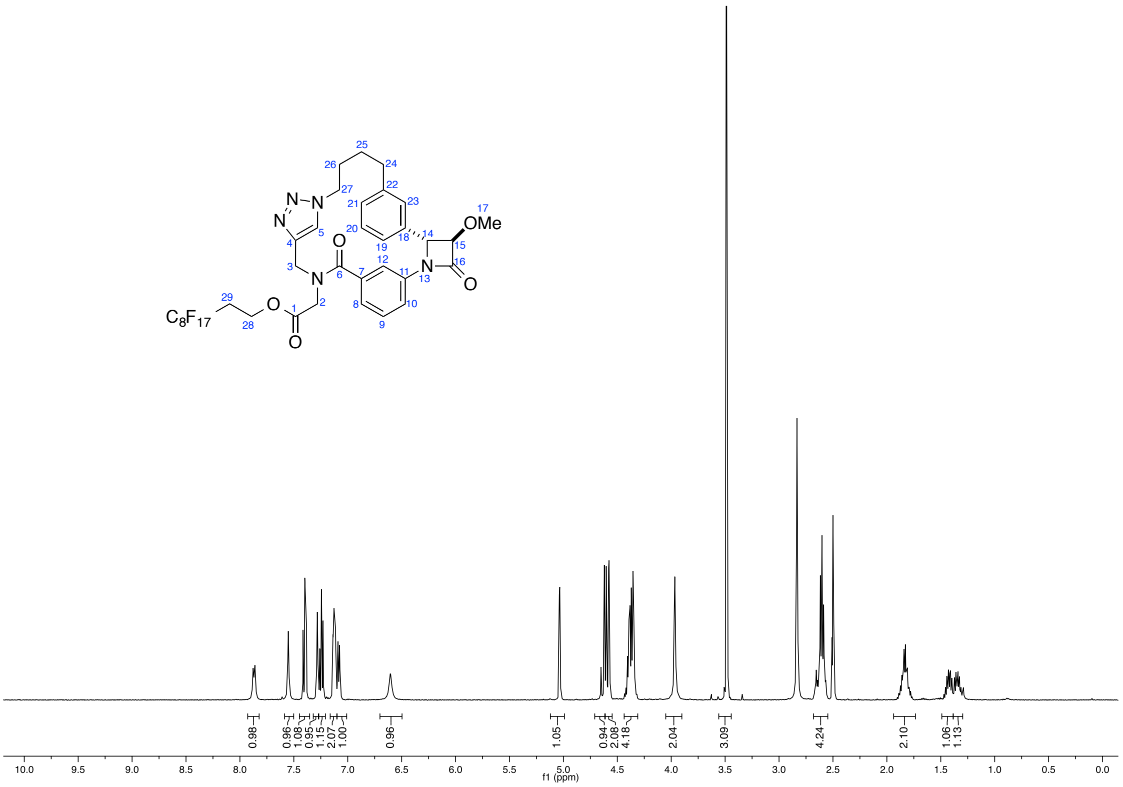

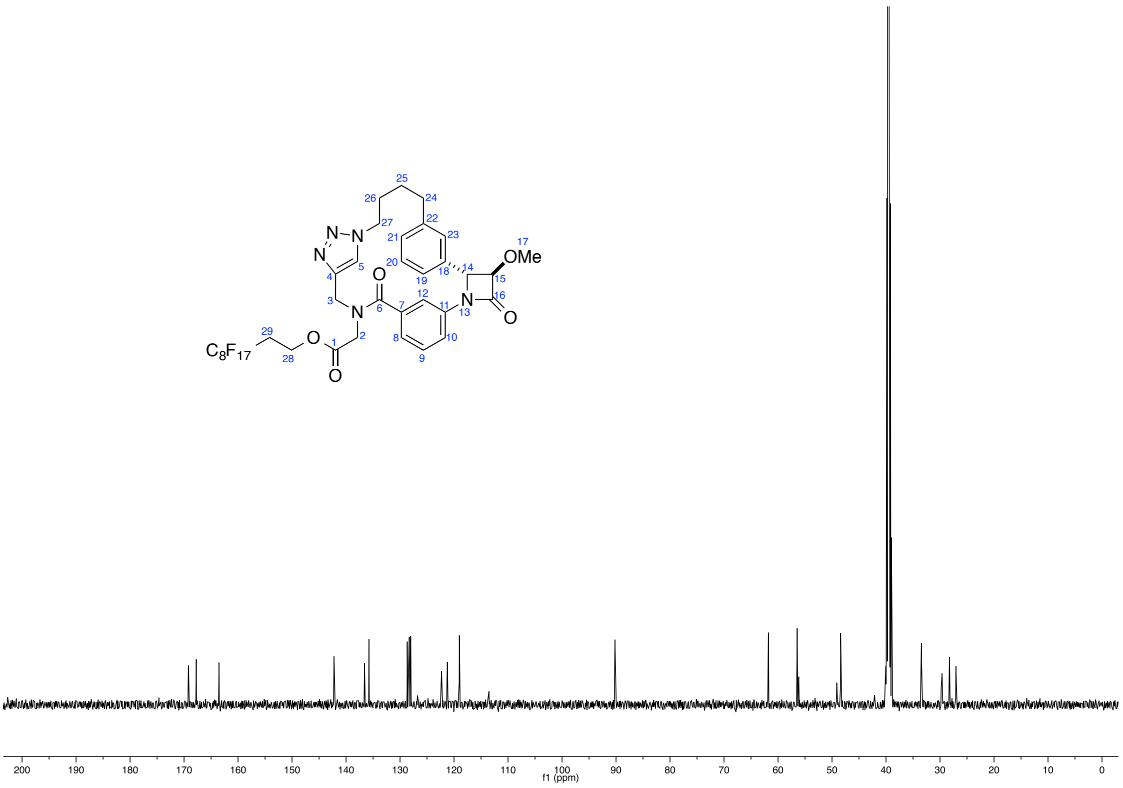

31c

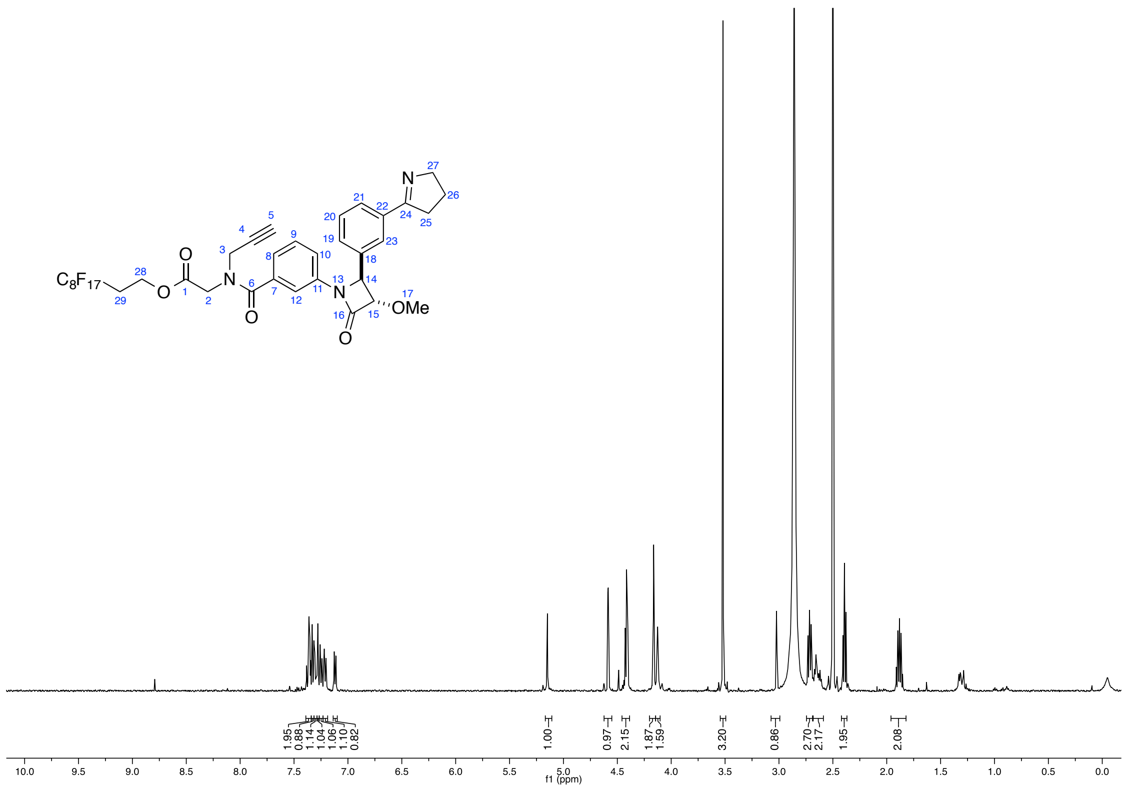

33

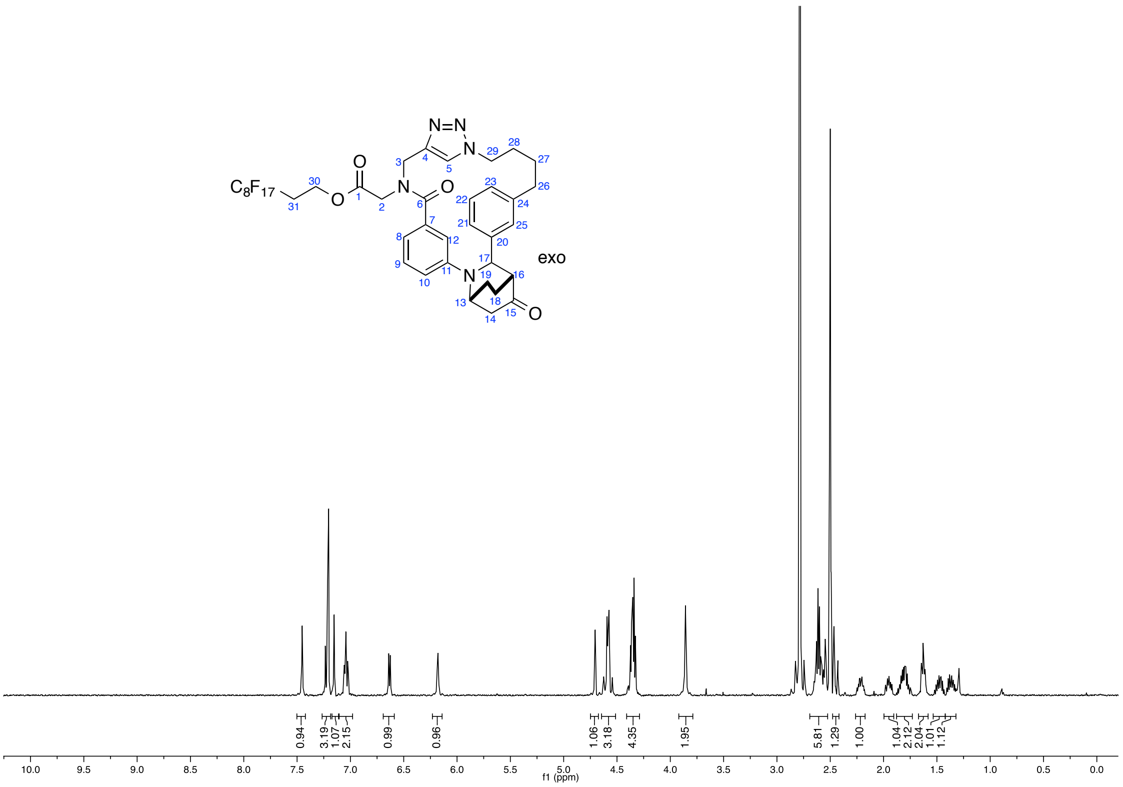

C_8F_{17}
N=N
exo
0.94
3.19
1.07
2.15
0.99
0.96
1.06
3.18
4.35
1.95
5.81
1.29
1.00
1.04
2.12
2.04
1.01
1.12
10.0 9.5 9.0 8.5 8.0 7.5 7.0 6.5 6.0 5.5 5.0 4.5 4.0 3.5 3.0 2.5 2.0 1.5 1.0 0.5 0.0
f1 (ppm)

25a

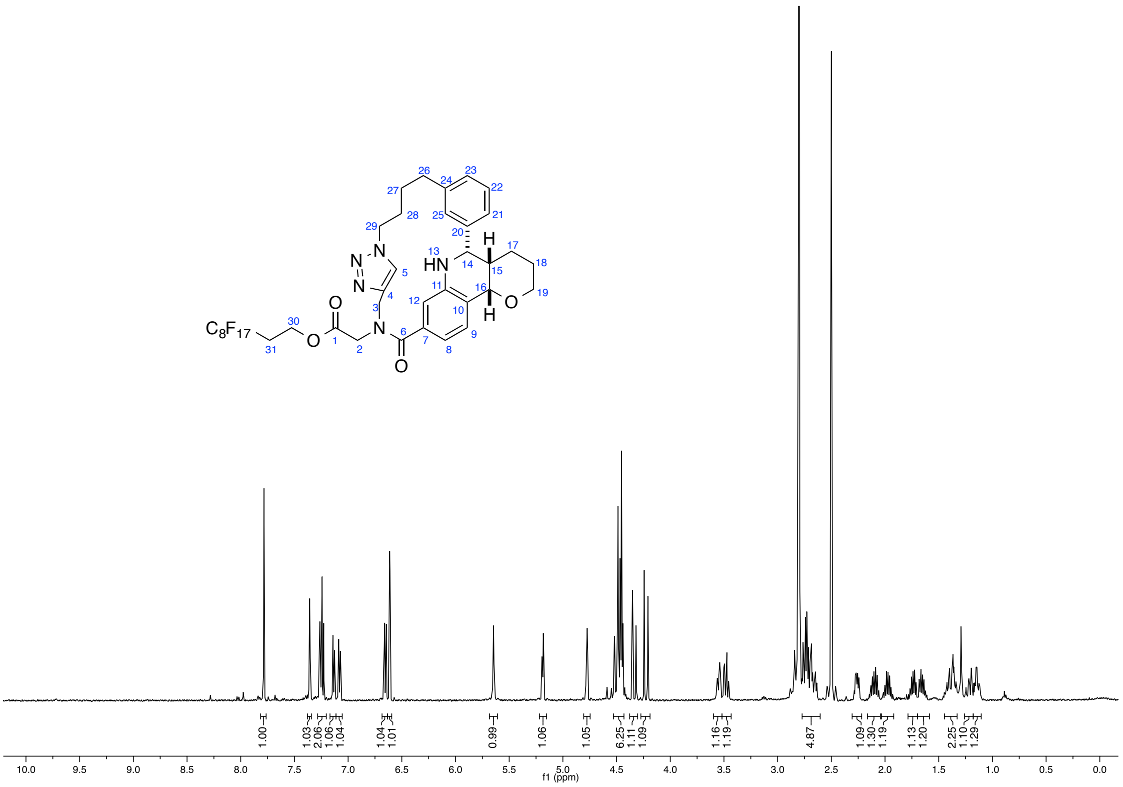

25b

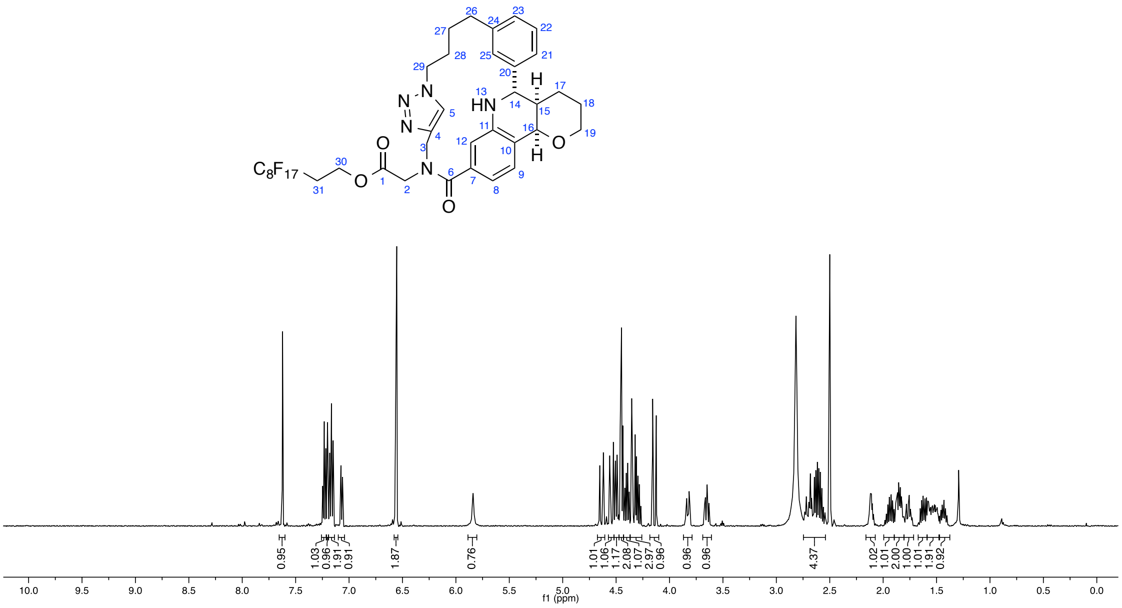

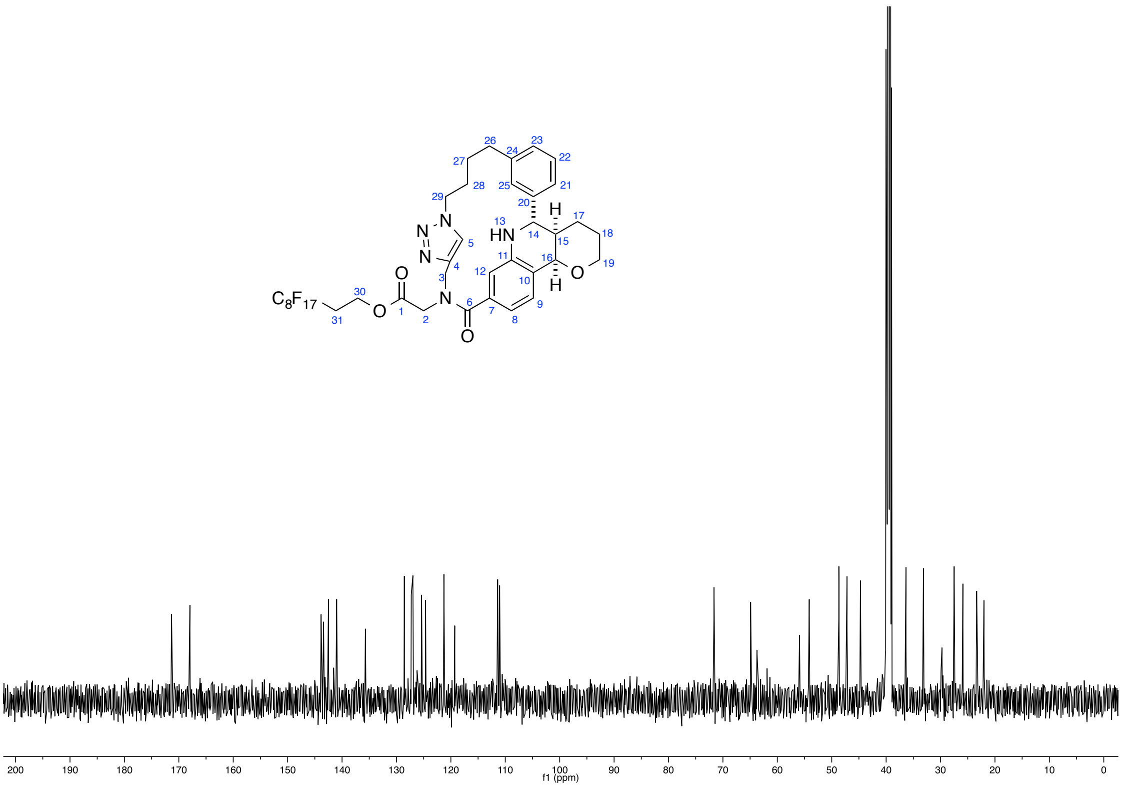

25c

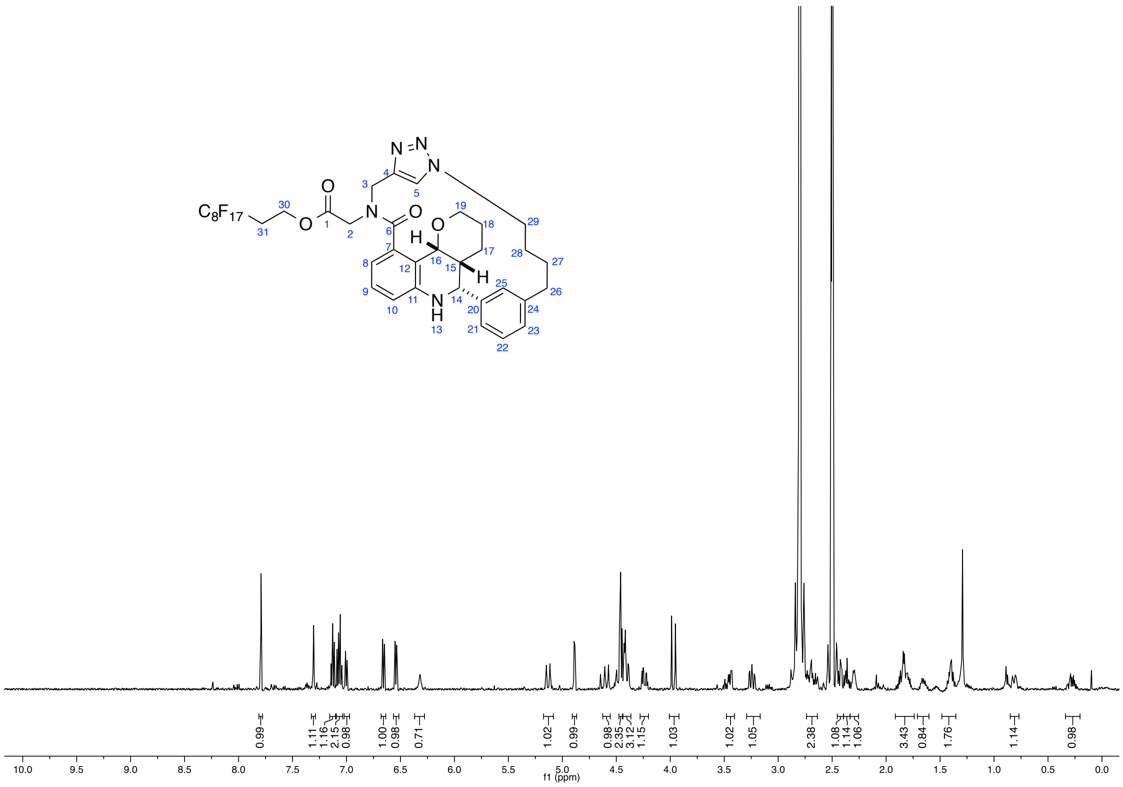

25e

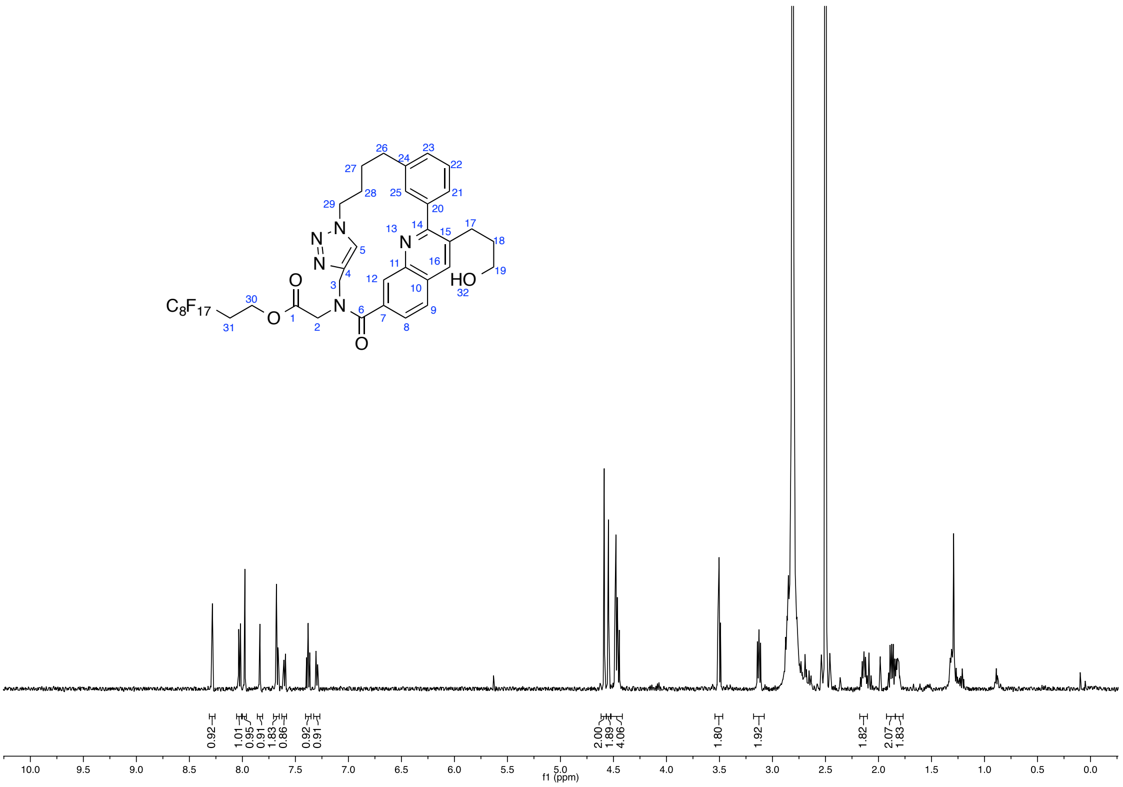

C8F17
HO
0.92
1.01
0.95
0.91
1.83
0.86
0.92
0.91
2.00
1.89
4.06
1.80
1.92
1.82
2.07
1.83
10.0 9.5 9.0 8.5 8.0 7.5 7.0 6.5 6.0 5.5 5.0 4.5 4.0 3.5 3.0 2.5 2.0 1.5 1.0 0.5 0.0
f1 (ppm)

25f

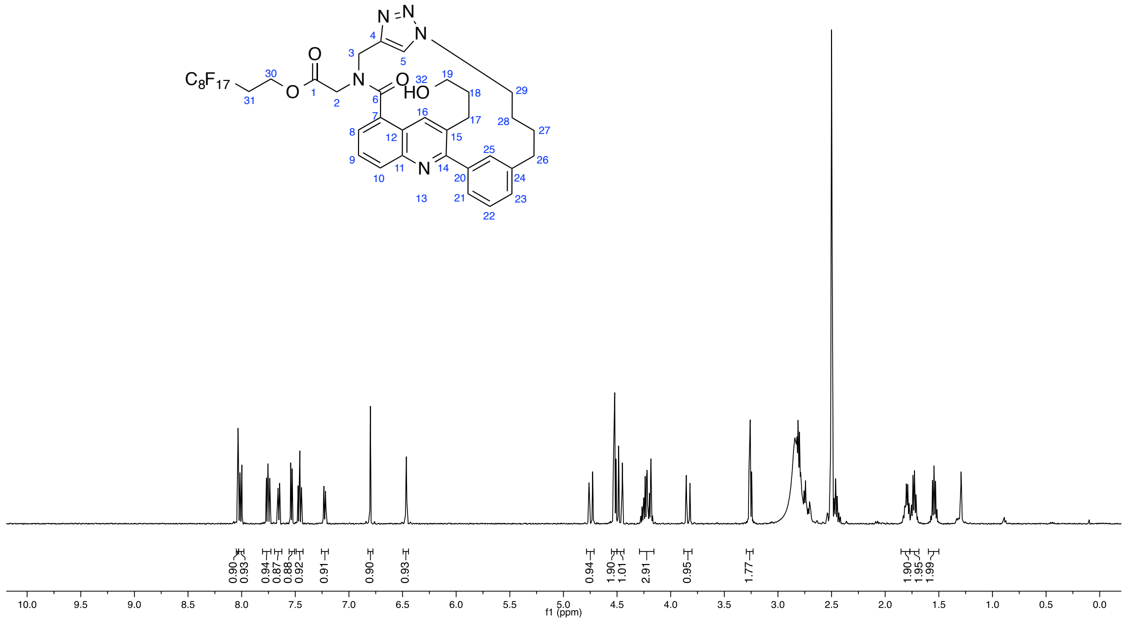

C8F17
O
N
N
N
N
HO
f1 (ppm)
0.90
0.93
0.94
0.87
0.88
0.92
0.91
0.90
0.93
0.94
1.90
1.01
2.91
0.95
1.77
1.90
1.95
1.99

23a

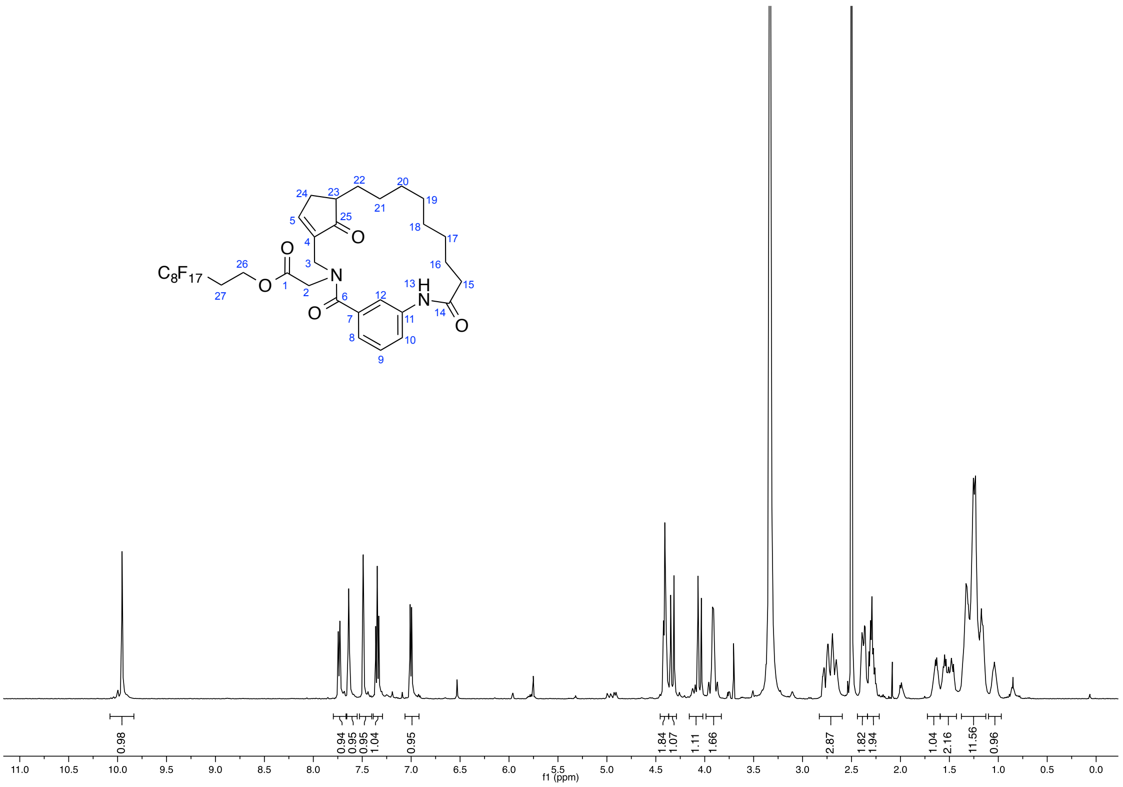

C8F17
0.98
0.94
0.95
0.95
1.04
0.95
1.84
1.07
1.11
1.66
2.87
1.82
1.94
1.04
2.16
11.56
0.96
f1 (ppm)

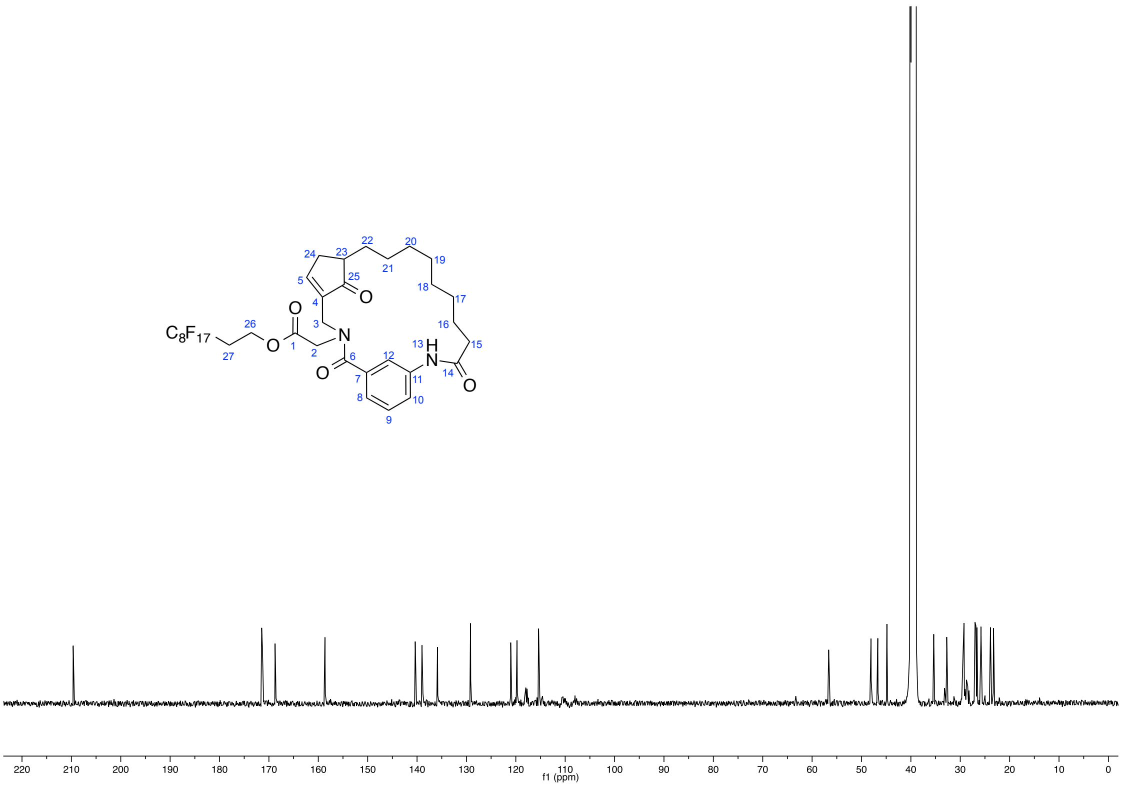

C8F17
f1 (ppm)

23b

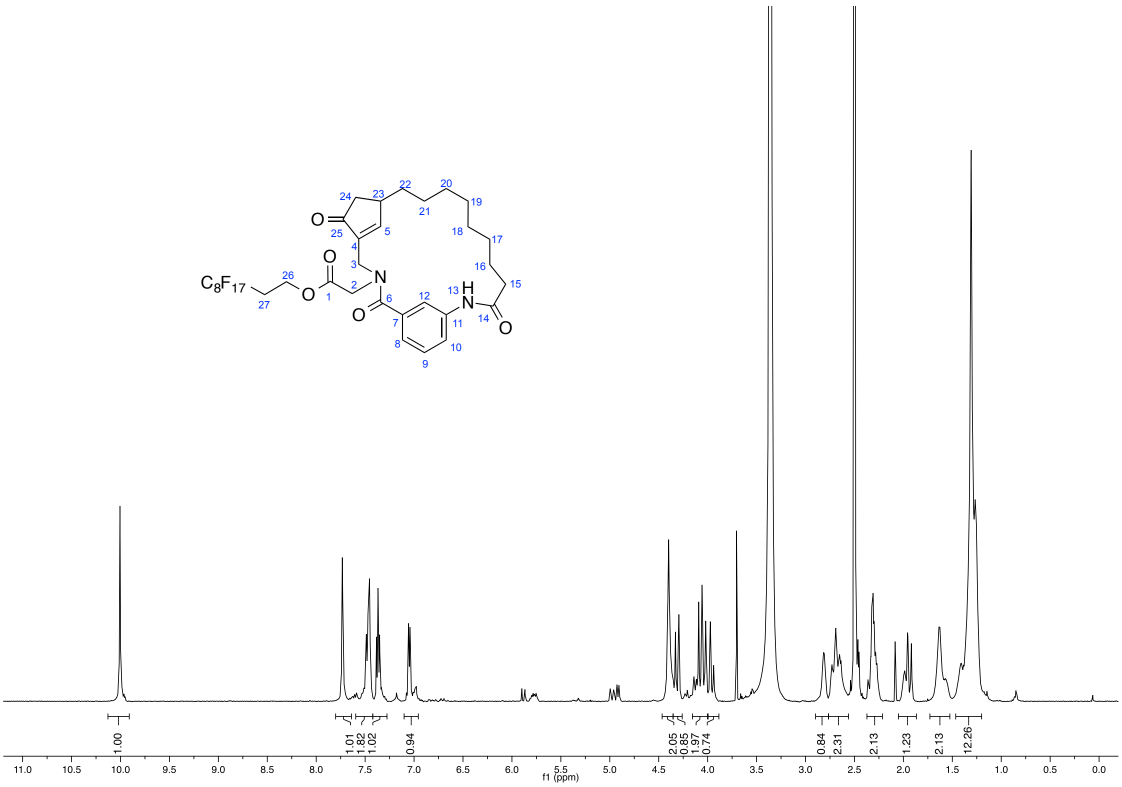
C8F17
1.00
1.01
1.82
1.02
0.94
2.05
0.85
1.97
0.74
0.84
2.31
2.13
1.23
2.13
12.26
f1 (ppm)

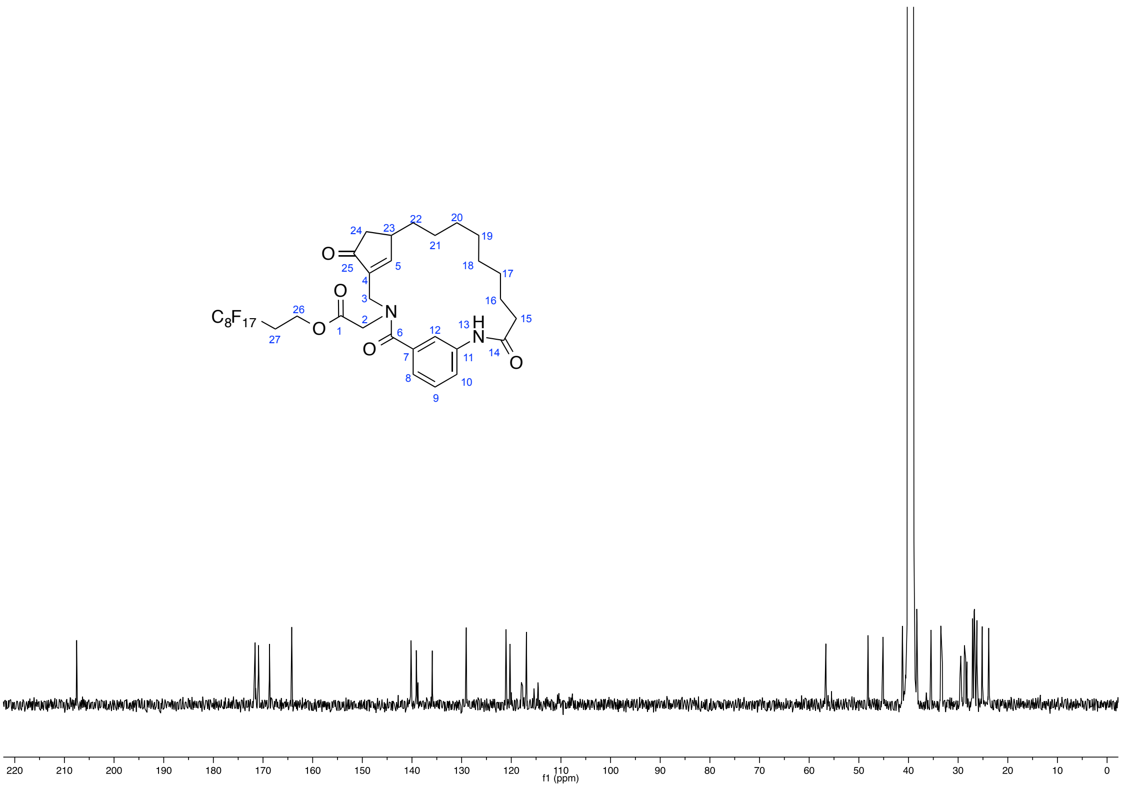
C8F17
f1 (ppm)

23c

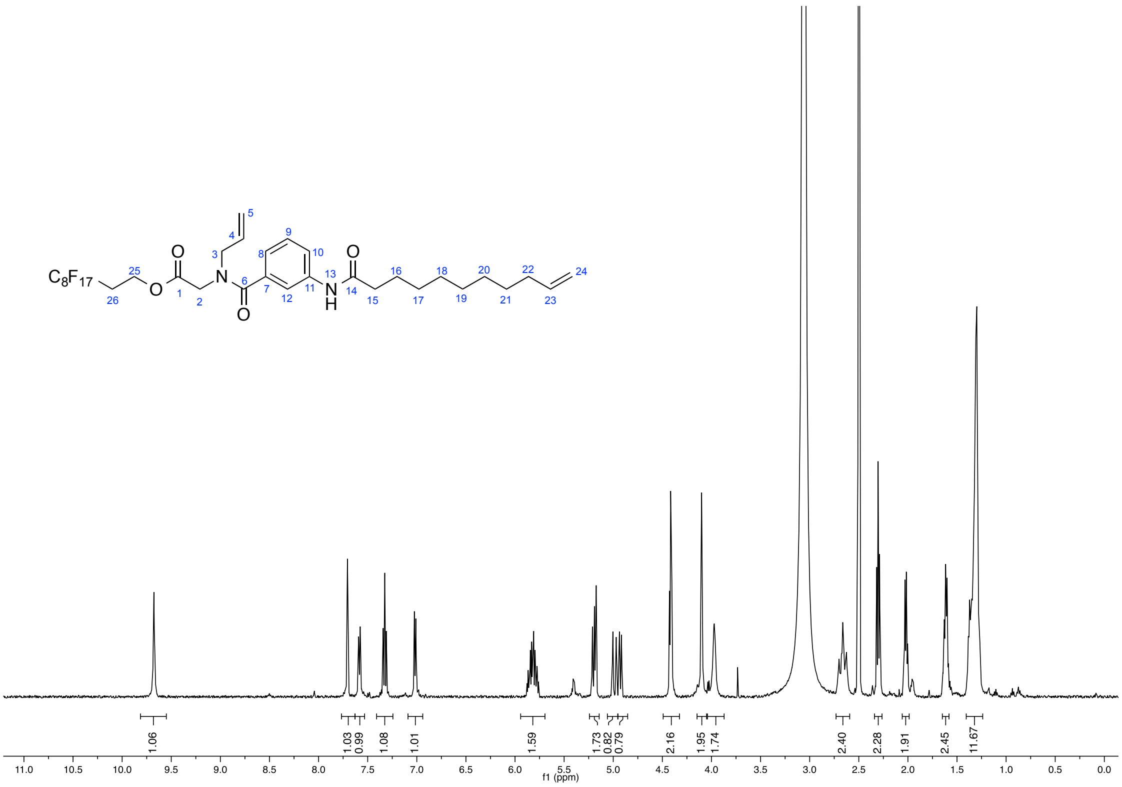

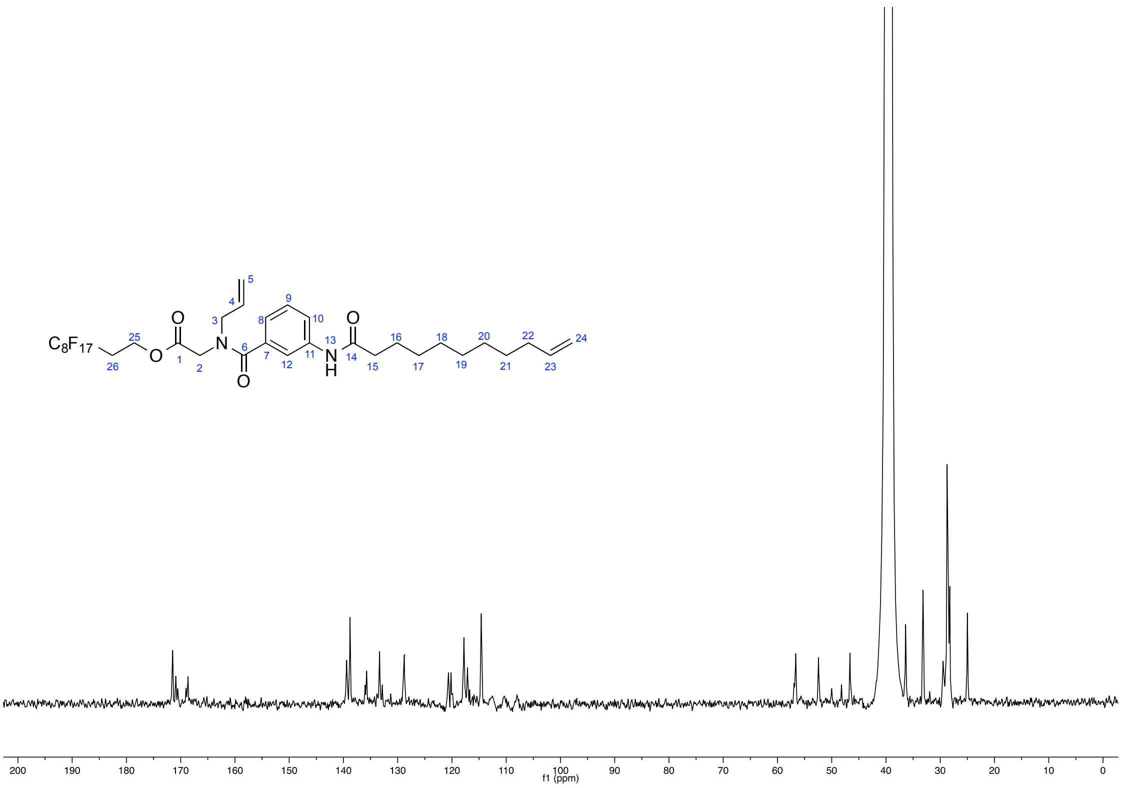

23d

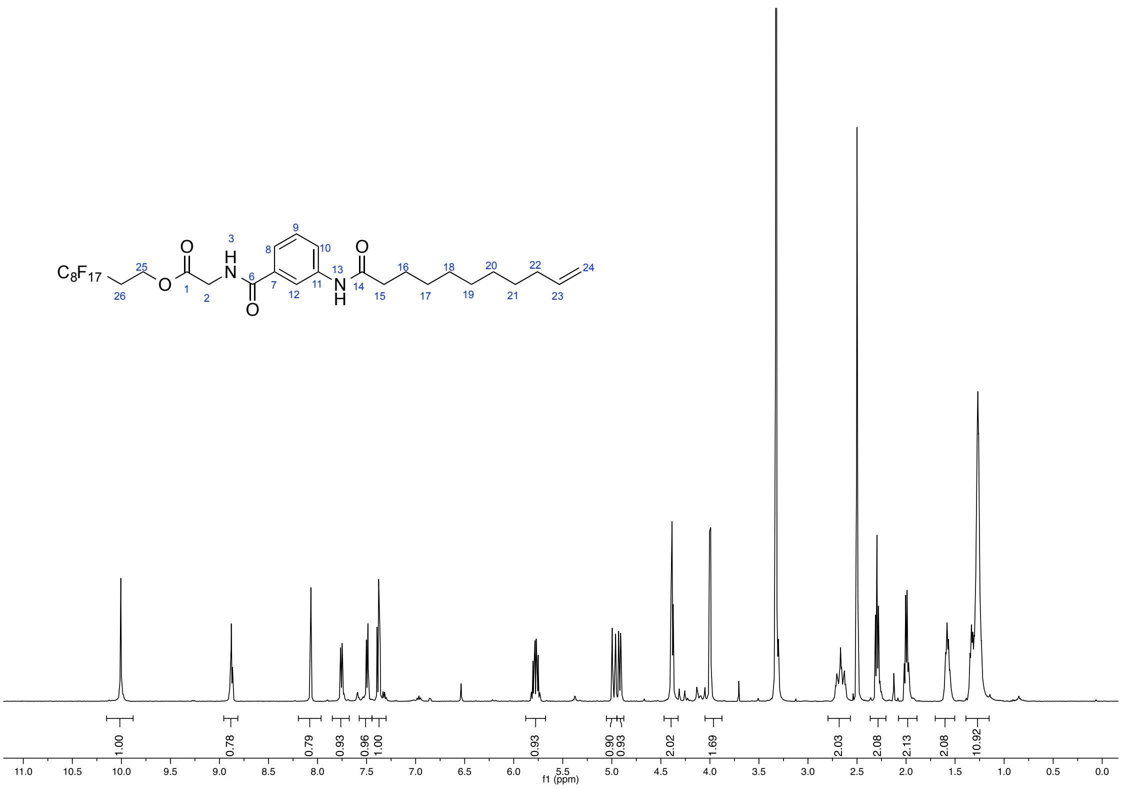

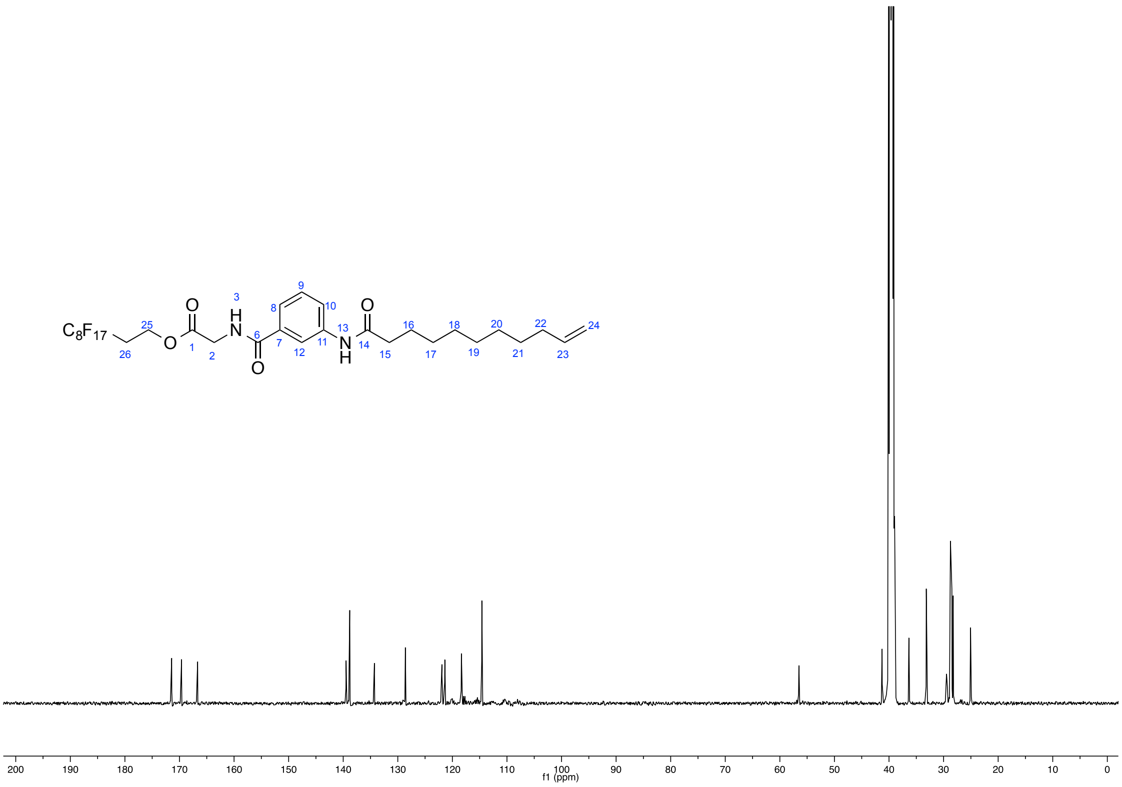

35

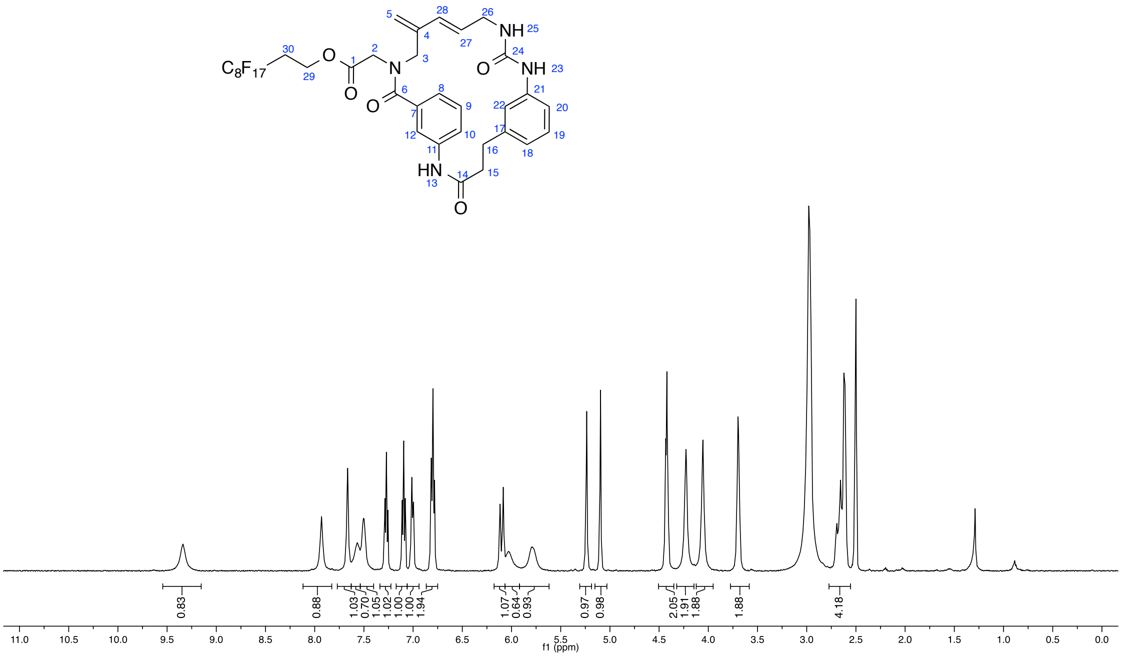
C8F17
0.83
0.88
1.03
0.70
1.05
1.02
1.00
1.00
1.94
1.07
0.64
0.93
0.97
0.98
2.05
1.91
1.88
1.88
4.18
f1 (ppm)

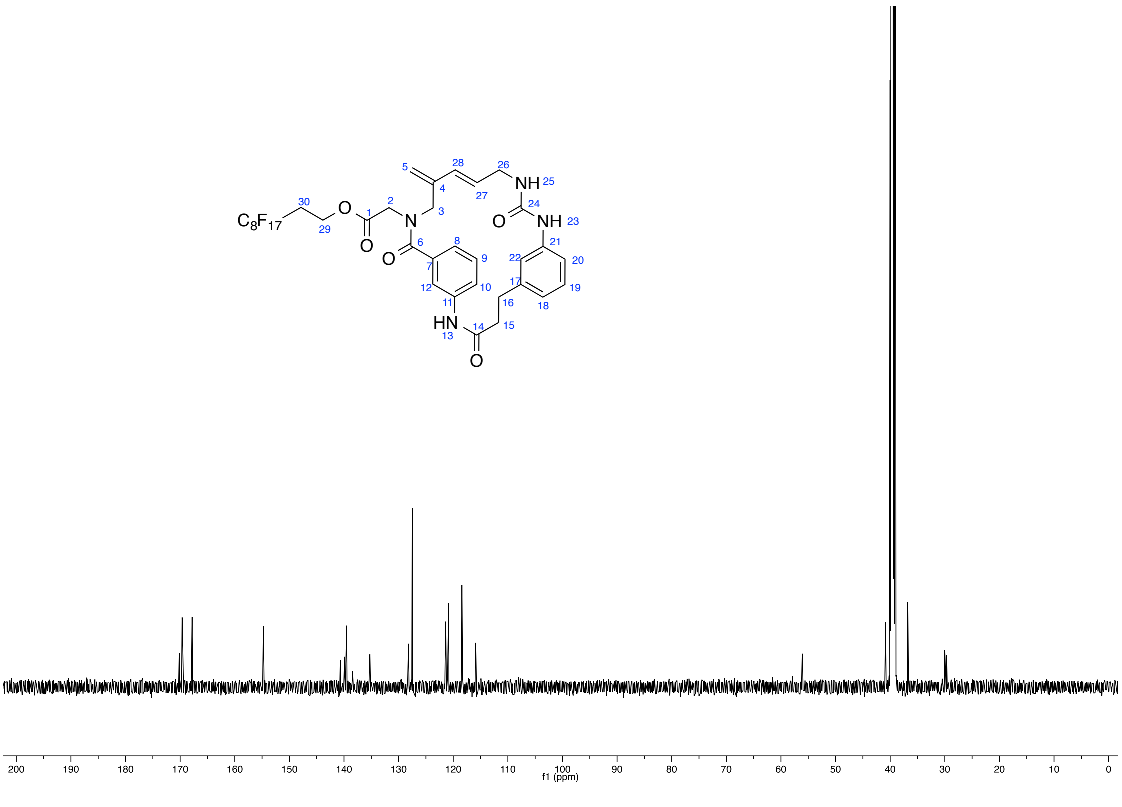
C8F17
f1 (ppm)

36a

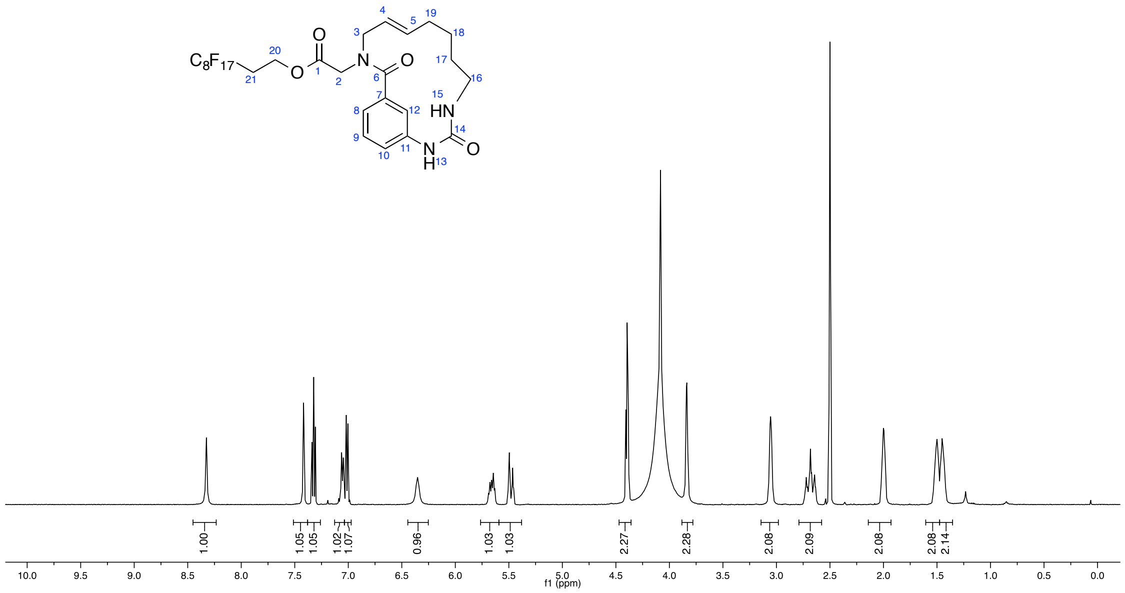

C_8F_{17}
O
N
HN
N
H
1.00
1.05
1.05
1.02
1.07
0.96
1.03
1.03
2.27
2.28
2.08
2.09
2.08
2.08
2.14
10.0 9.5 9.0 8.5 8.0 7.5 7.0 6.5 6.0 5.5 5.0 4.5 4.0 3.5 3.0 2.5 2.0 1.5 1.0 0.5 0.0
f1 (ppm)

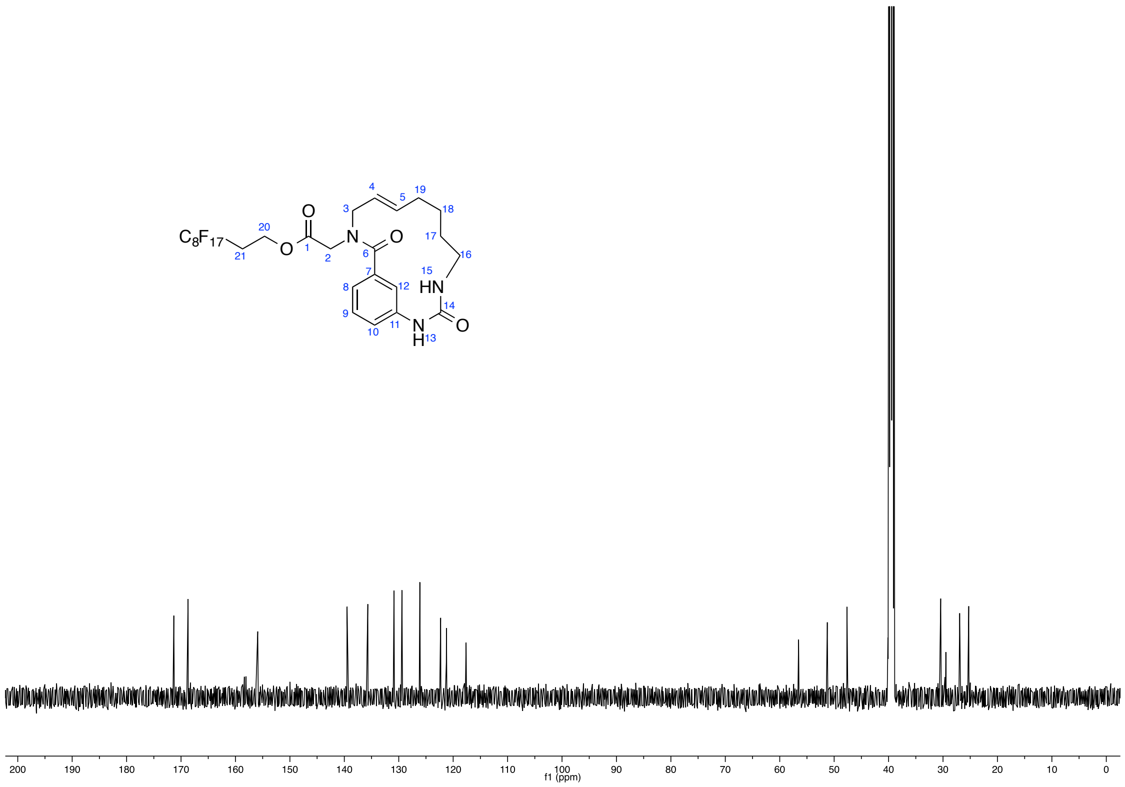

C_8F_{17}
O
N
HN
N
H
200 190 180 170 160 150 140 130 120 110 100 90 80 70 60 50 40 30 20 10 0
f1 (ppm)

36b

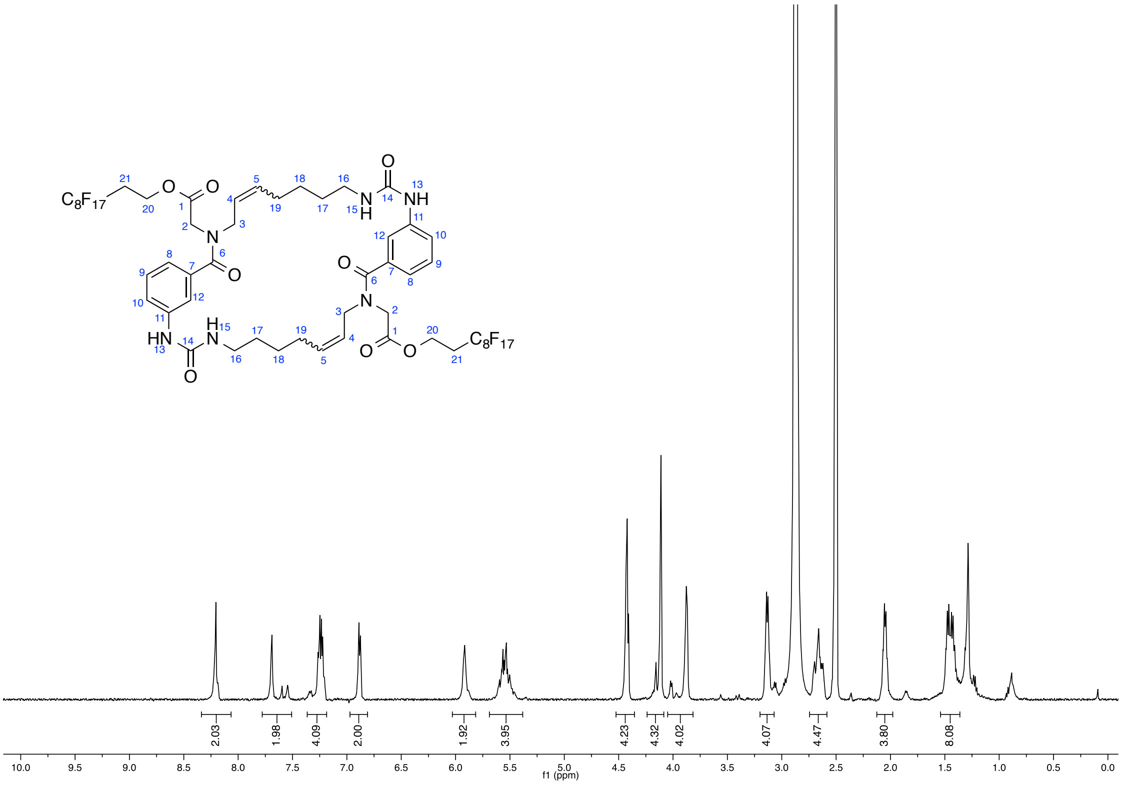

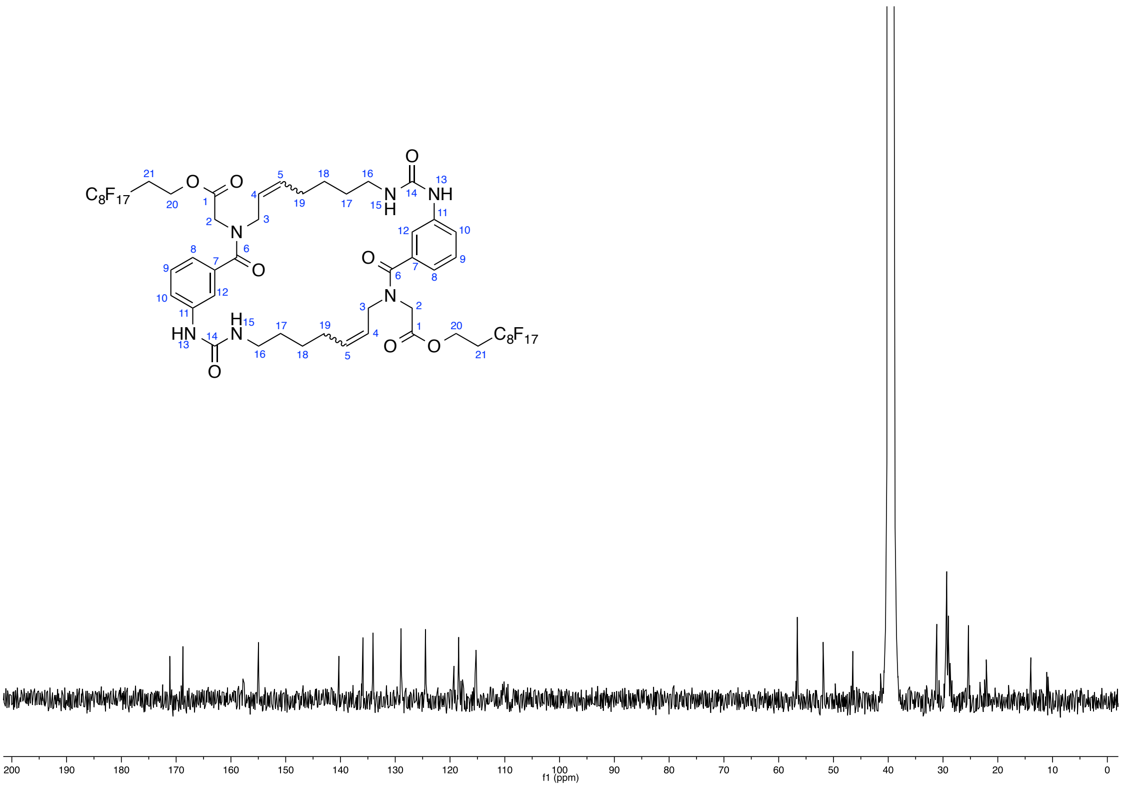

37

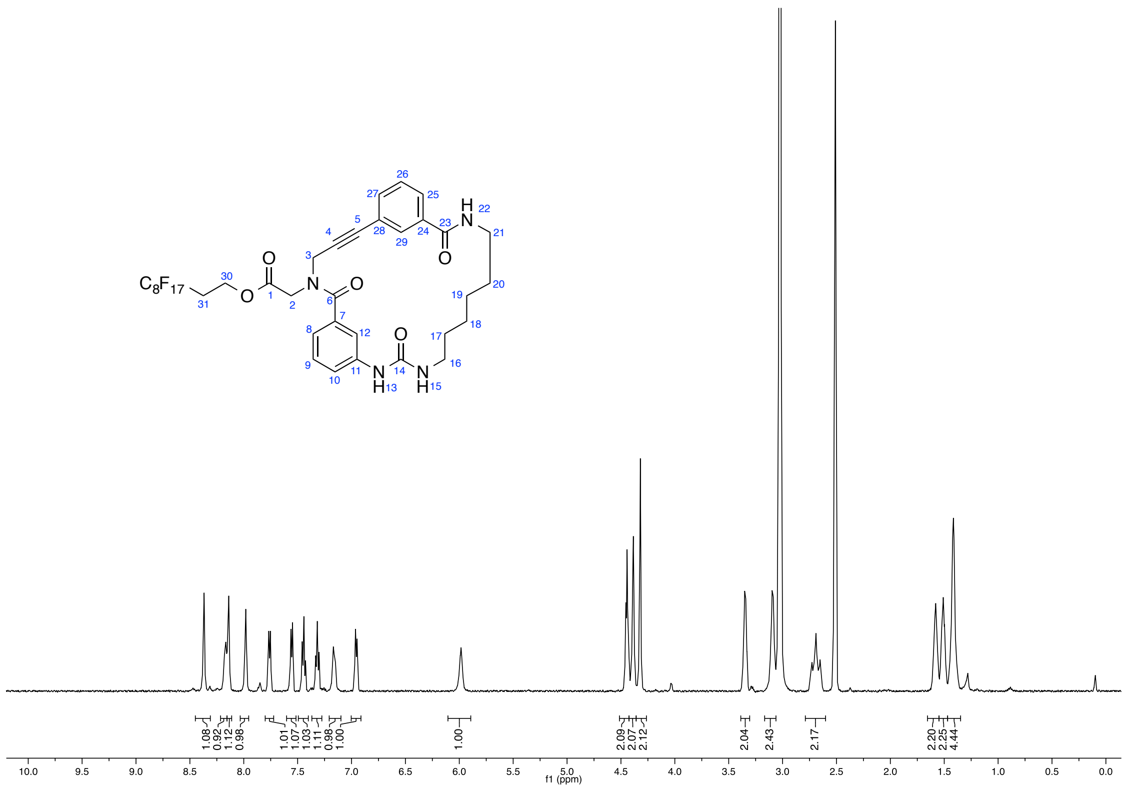

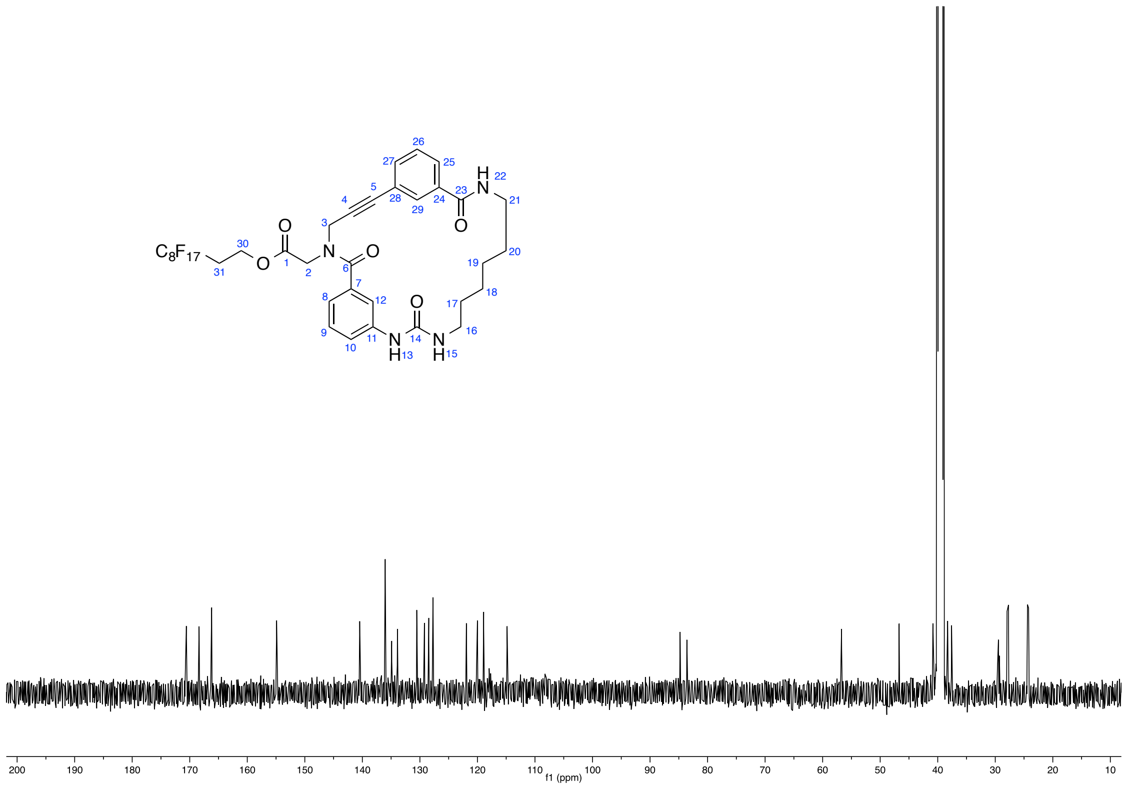

38

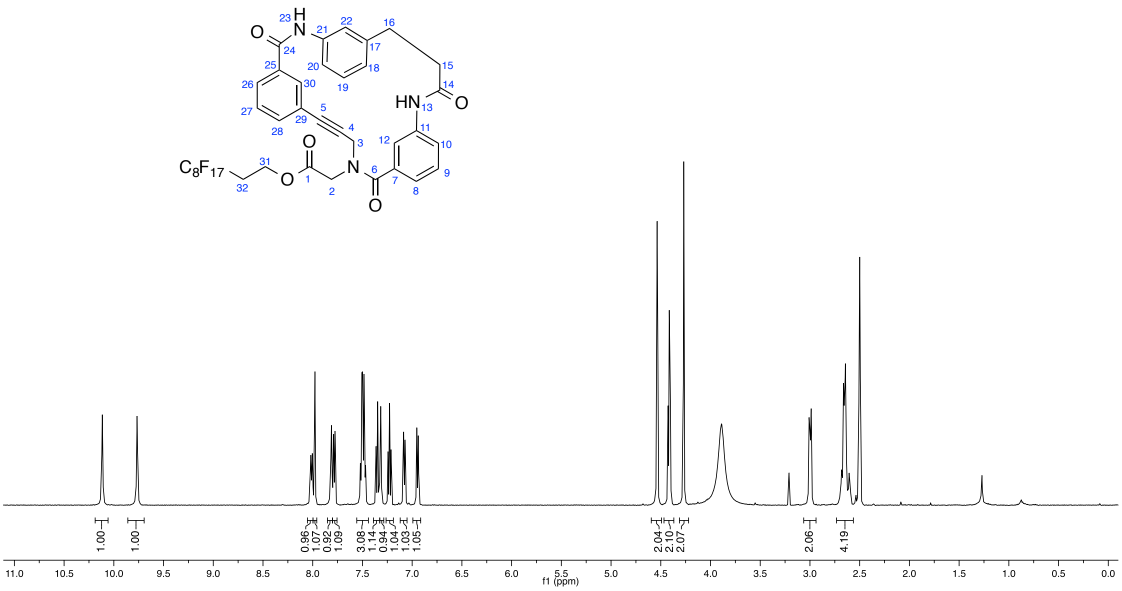

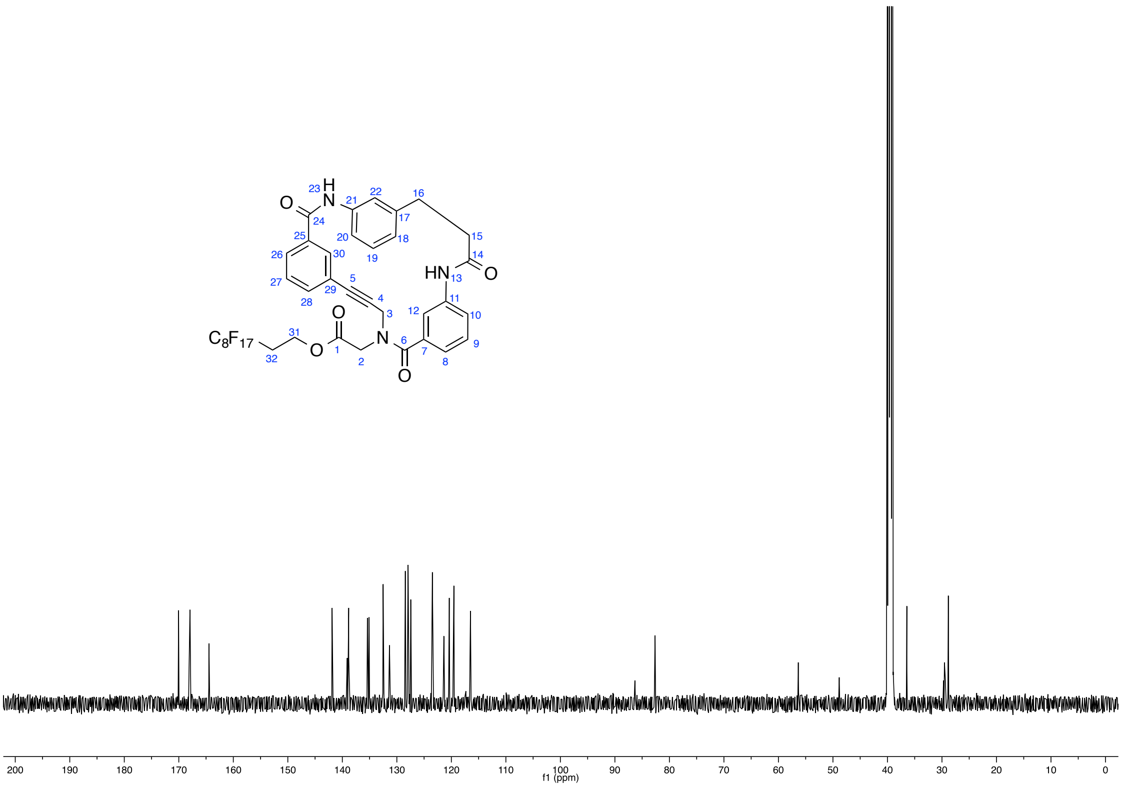

39

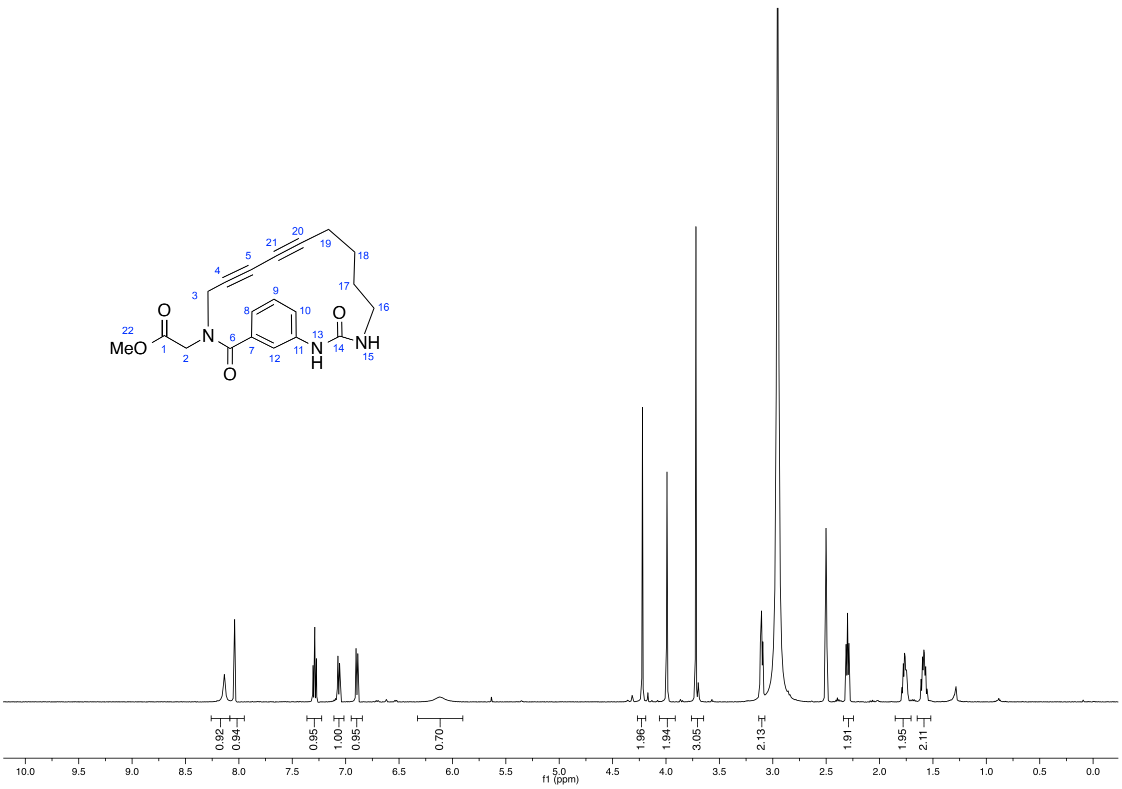

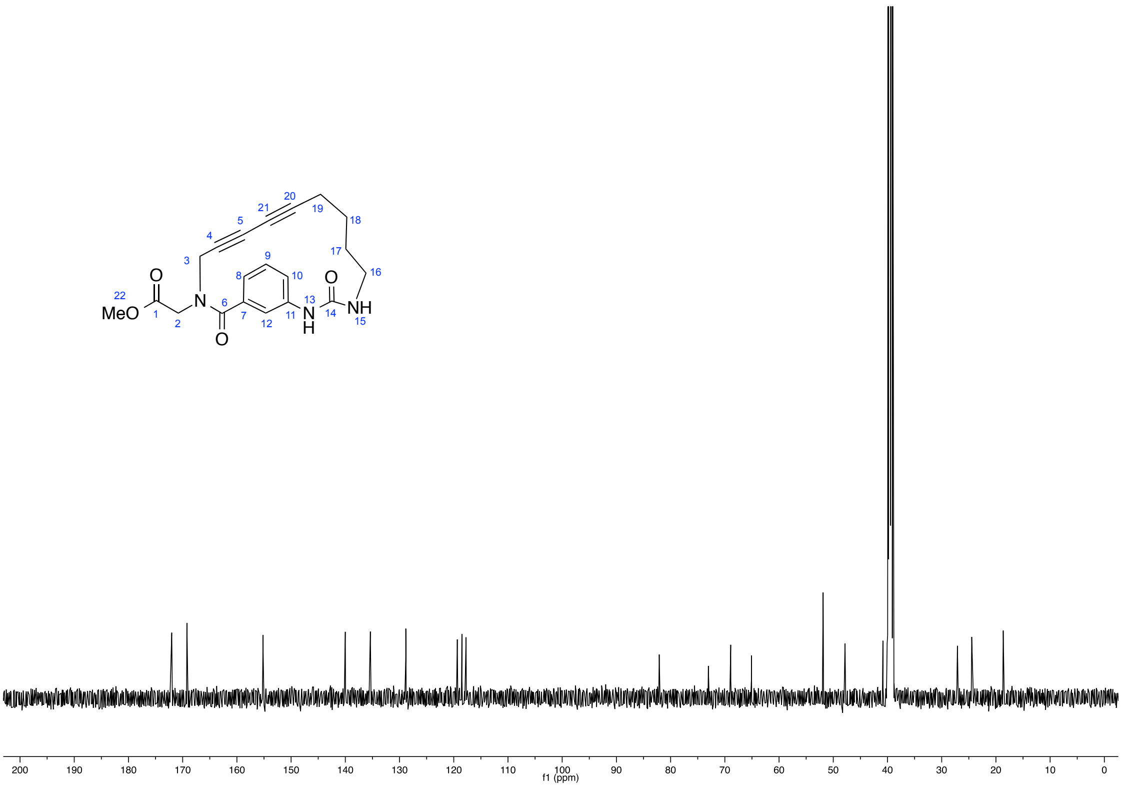

40a

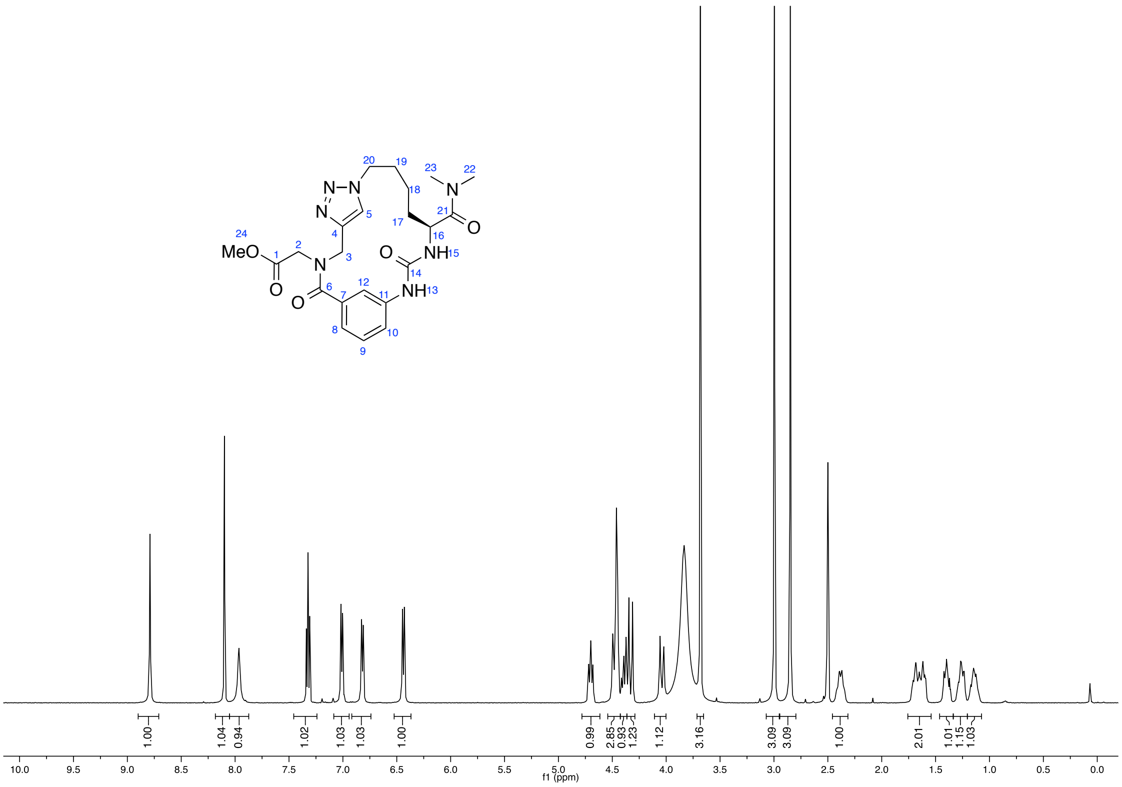
MeO
1.00
1.04
0.94
1.02
1.03
1.03
1.00
0.99
2.85
0.93
1.23
1.12
3.16
3.09
3.09
1.00
2.01
1.01
1.15
1.03
f1 (ppm)

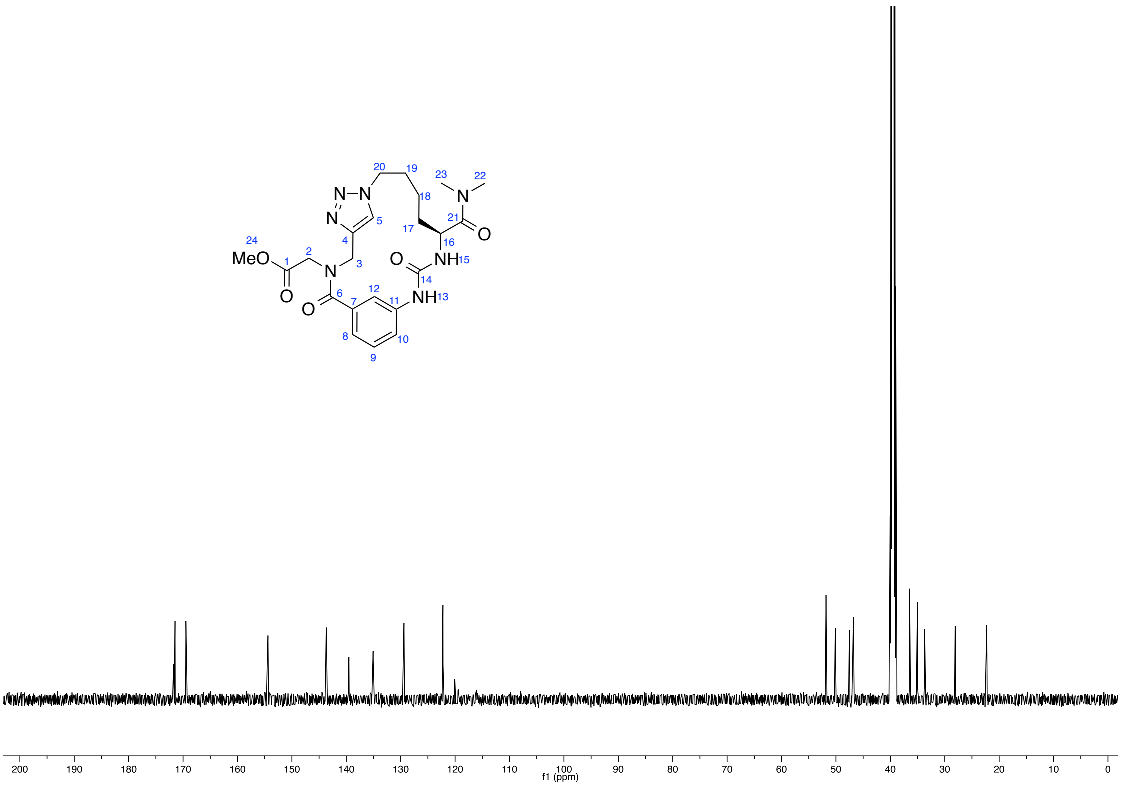
MeO
f1 (ppm)

40b

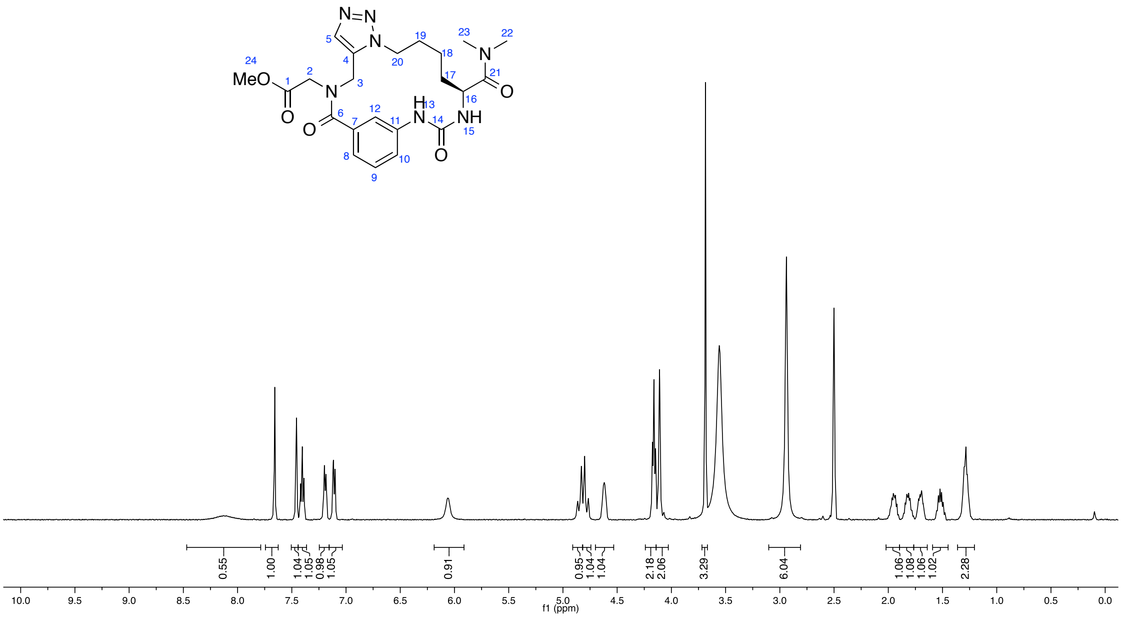
MeO
0.55
1.00
1.04
1.05
0.98
1.05
0.91
0.95
1.04
1.04
2.18
2.06
3.29
6.04
1.06
1.08
1.06
1.02
2.28
f1 (ppm)

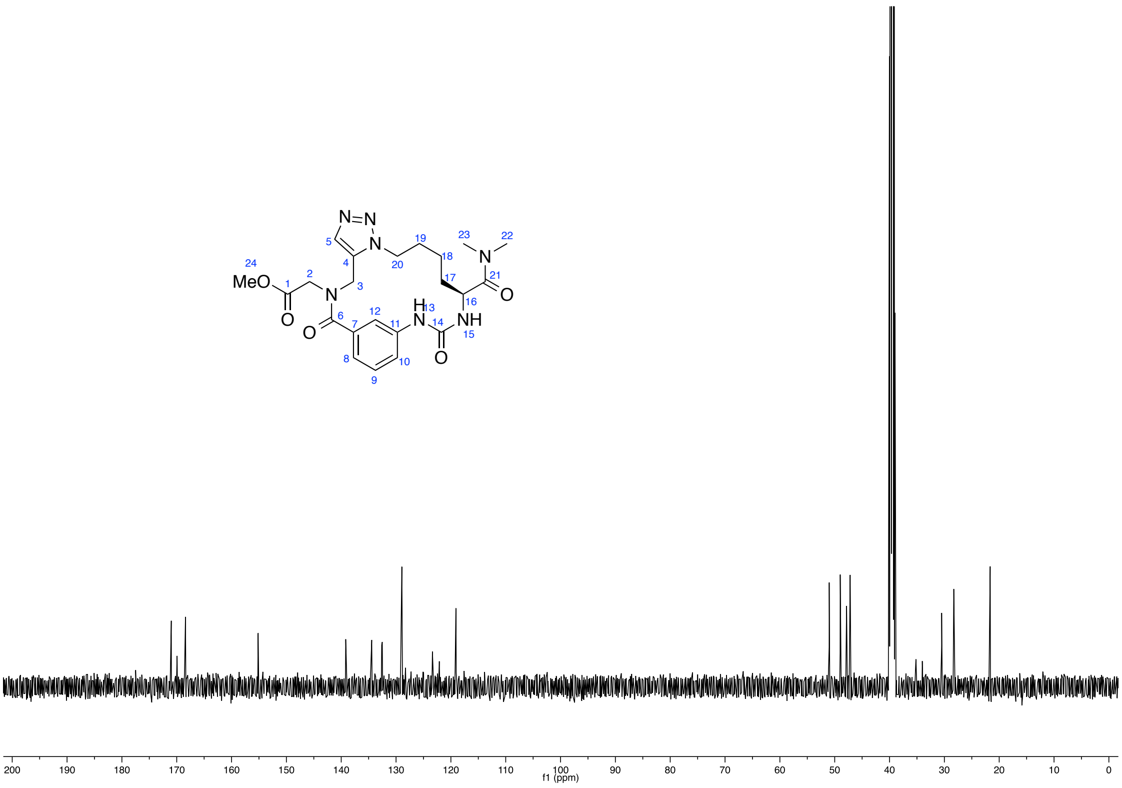
MeO
f1 (ppm)

40d

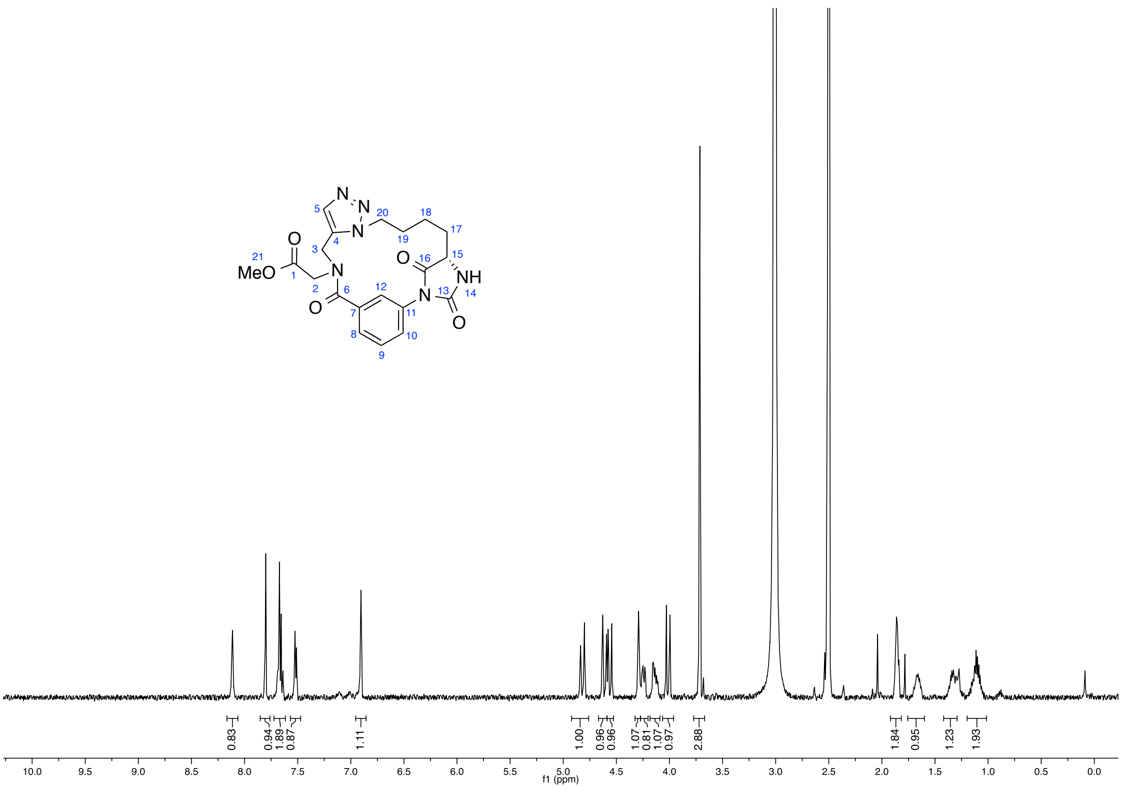

40c

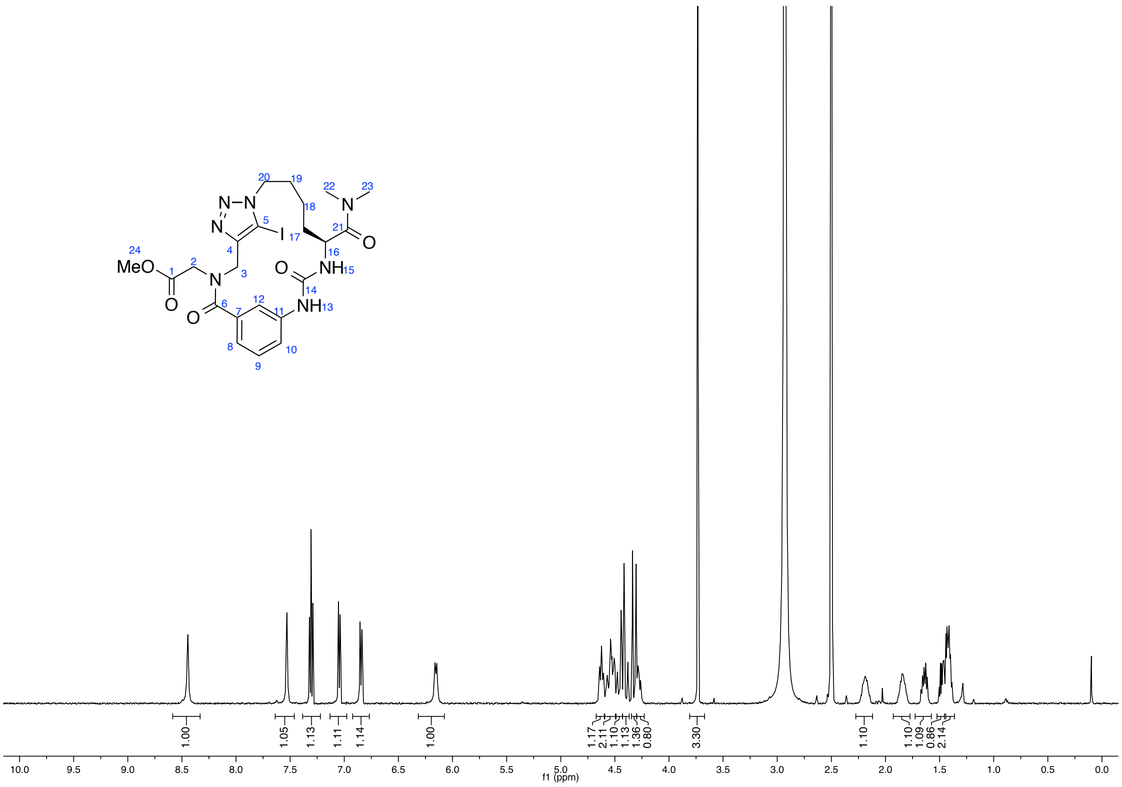

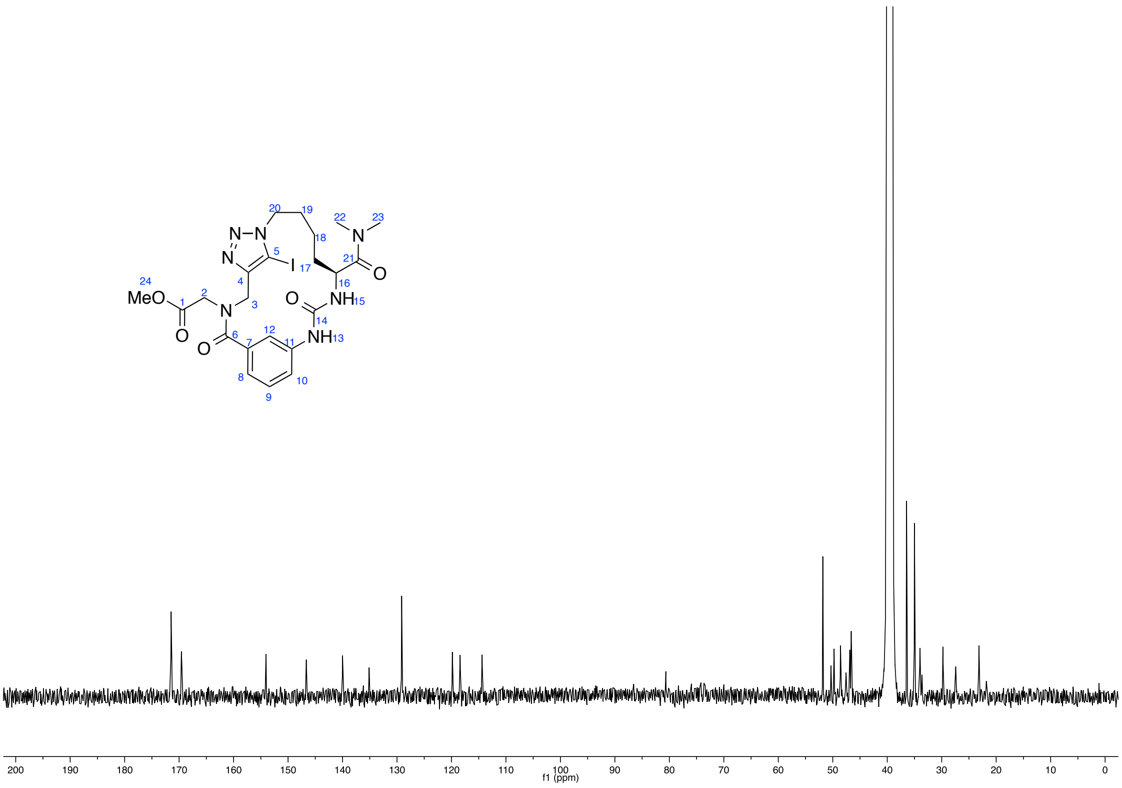

41a

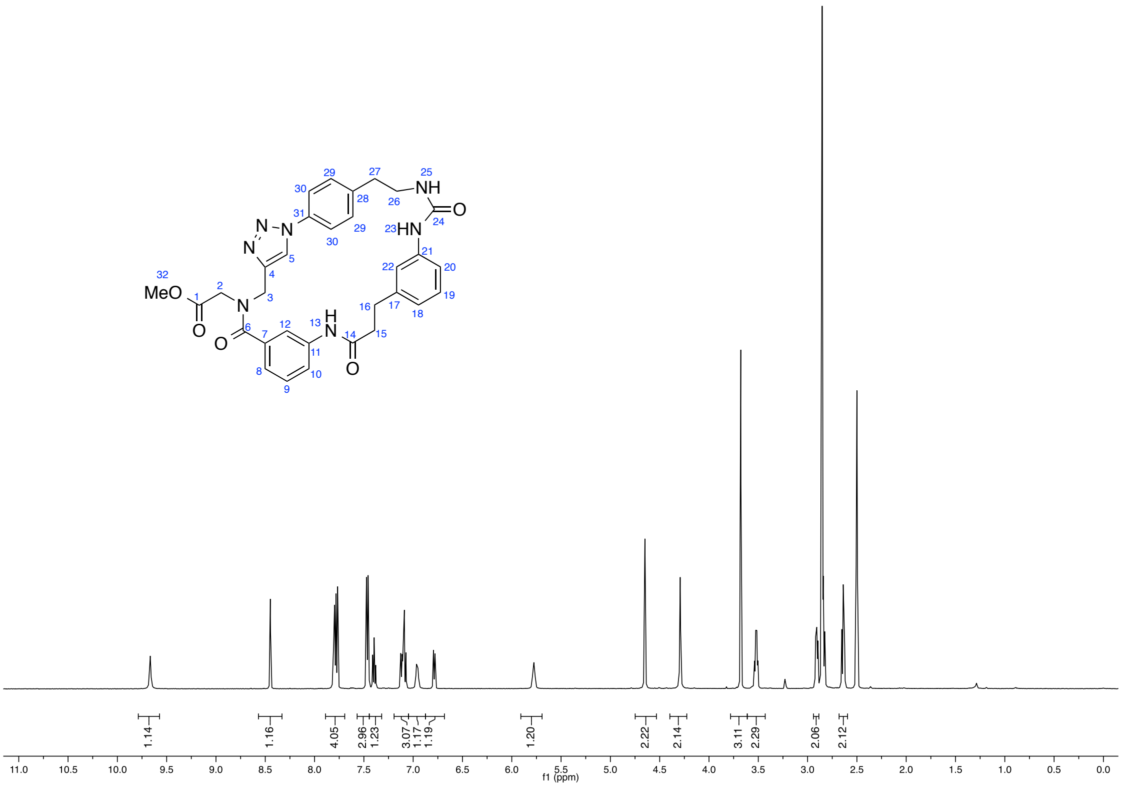

1.14
1.16
4.05
2.96
1.23
3.07
1.17
1.19
1.20
2.22
2.14
3.11
2.29
2.06
2.12
11.0
10.5
10.0
9.5
9.0
8.5
8.0
7.5
7.0
6.5
6.0
5.5
5.0
4.5
4.0
3.5
3.0
2.5
2.0
1.5
1.0
0.5
0.0
f1 (ppm)

41b

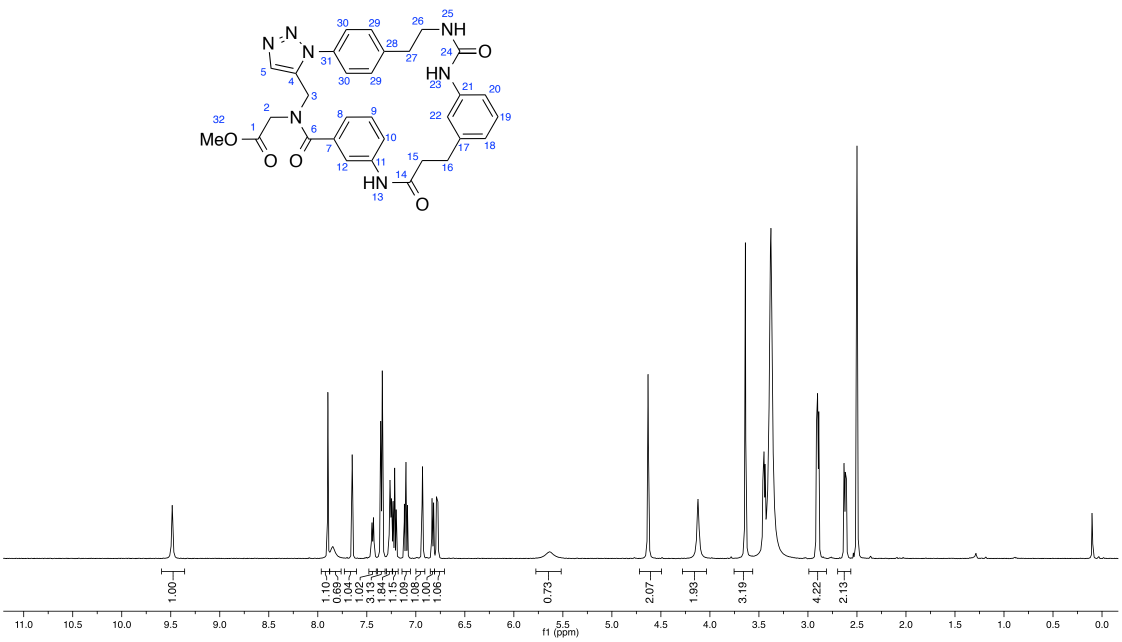

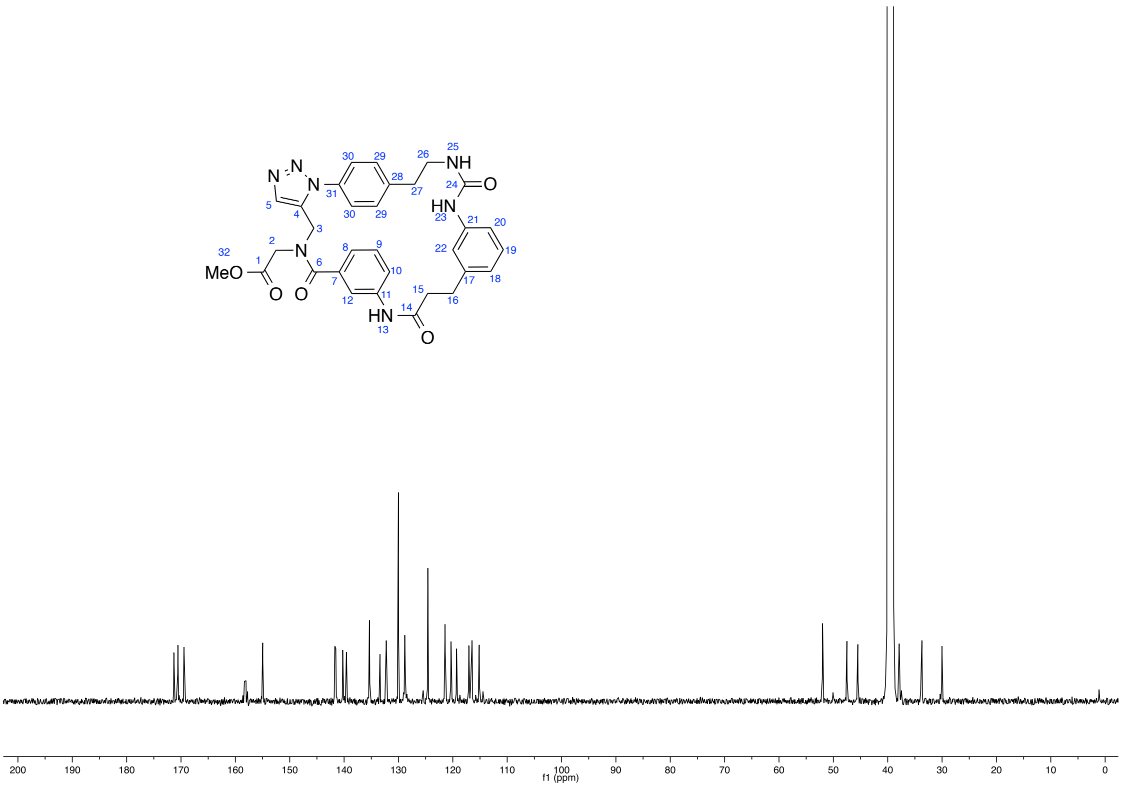

42a

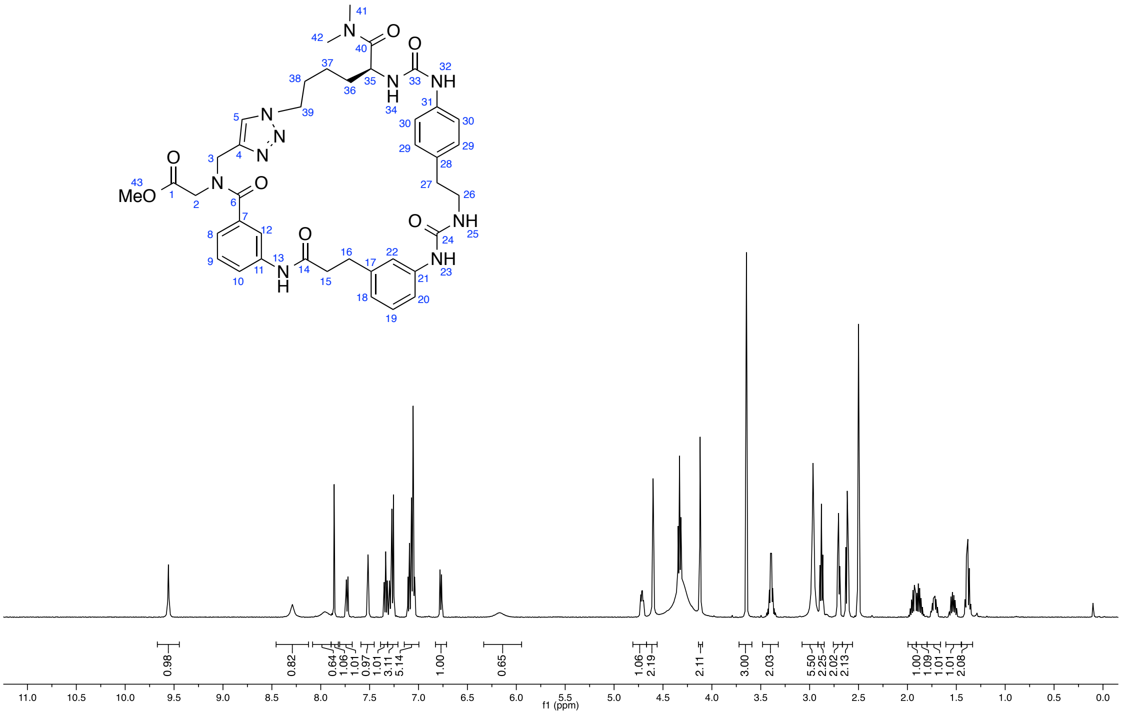

42b

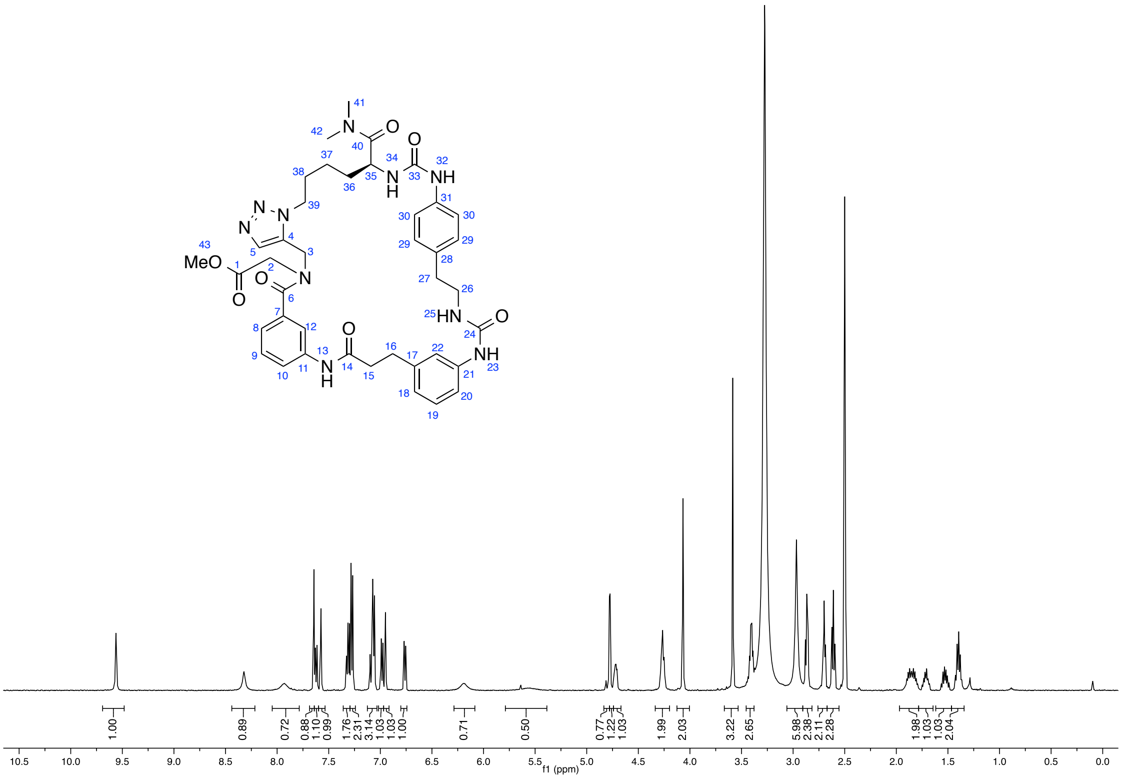

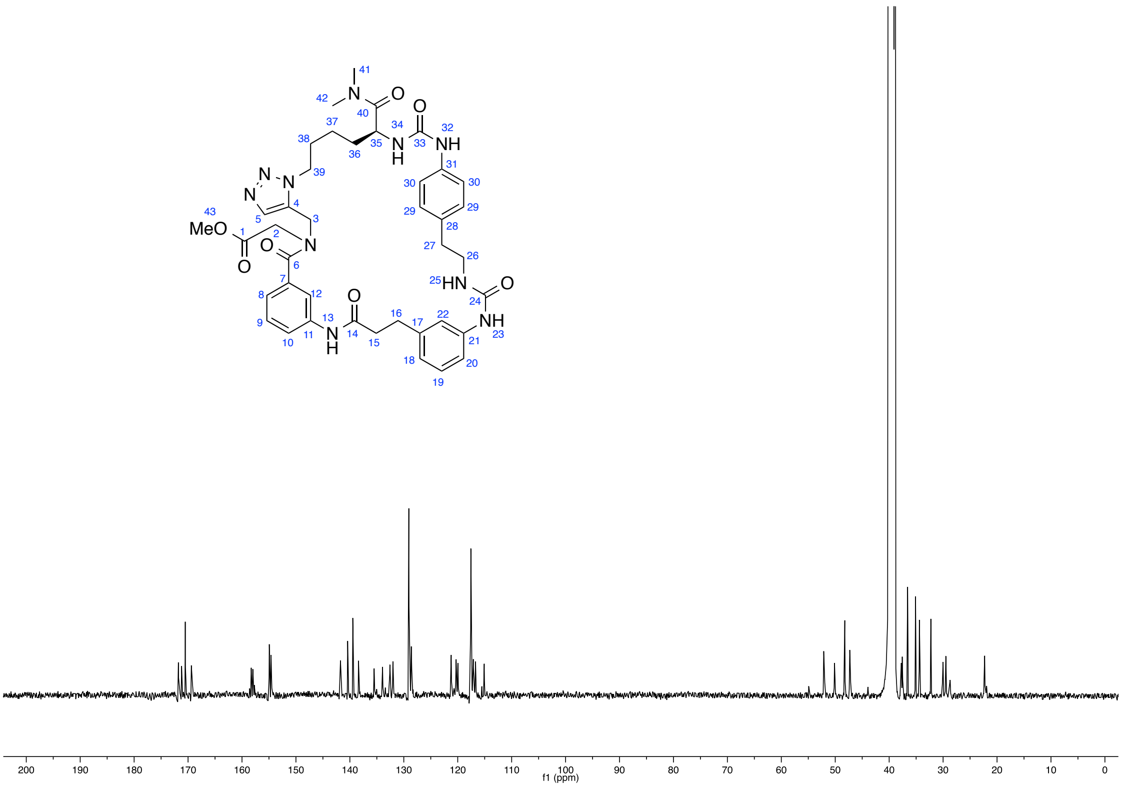

43a

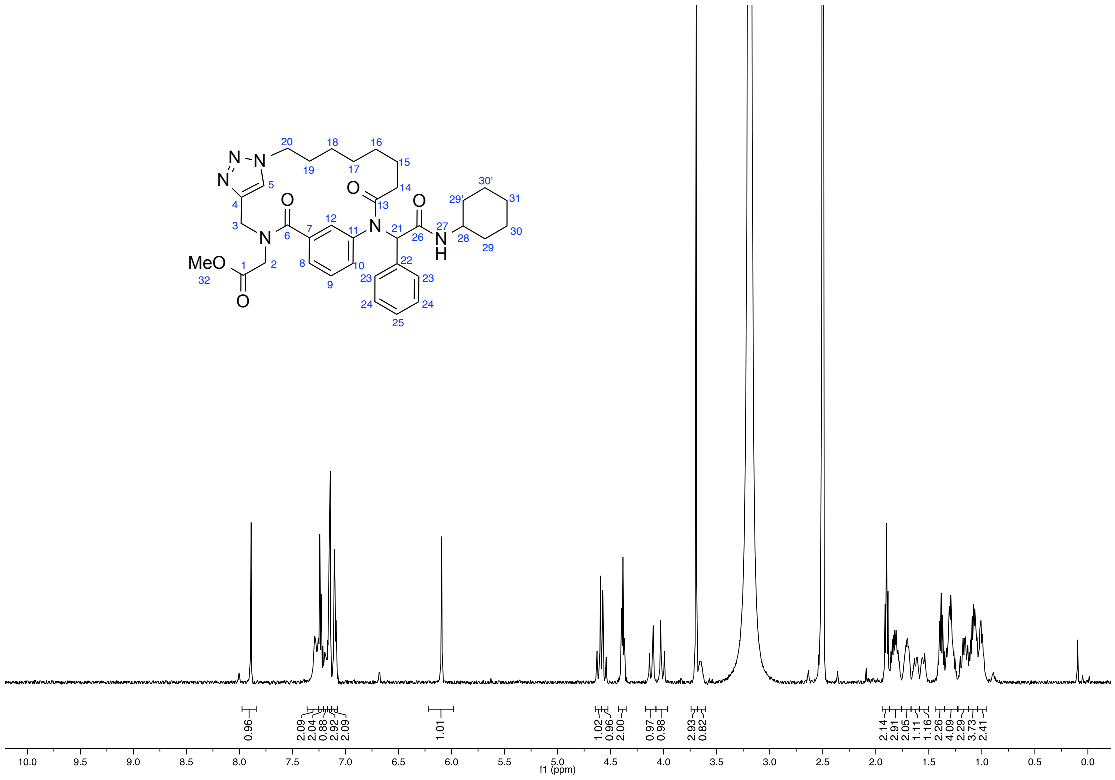

43b

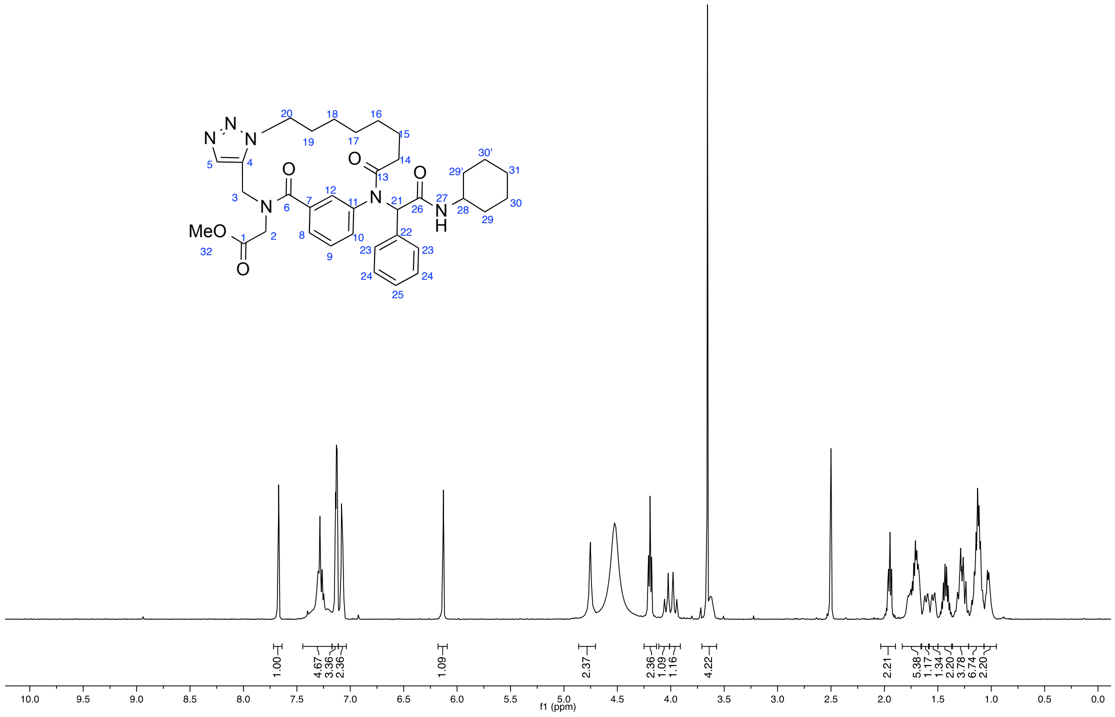
MeO
f1 (ppm)
1.00
4.67
3.36
2.36
1.09
2.37
2.36
1.09
1.16
4.22
2.21
5.38
1.17
1.34
2.20
3.78
6.74
2.20

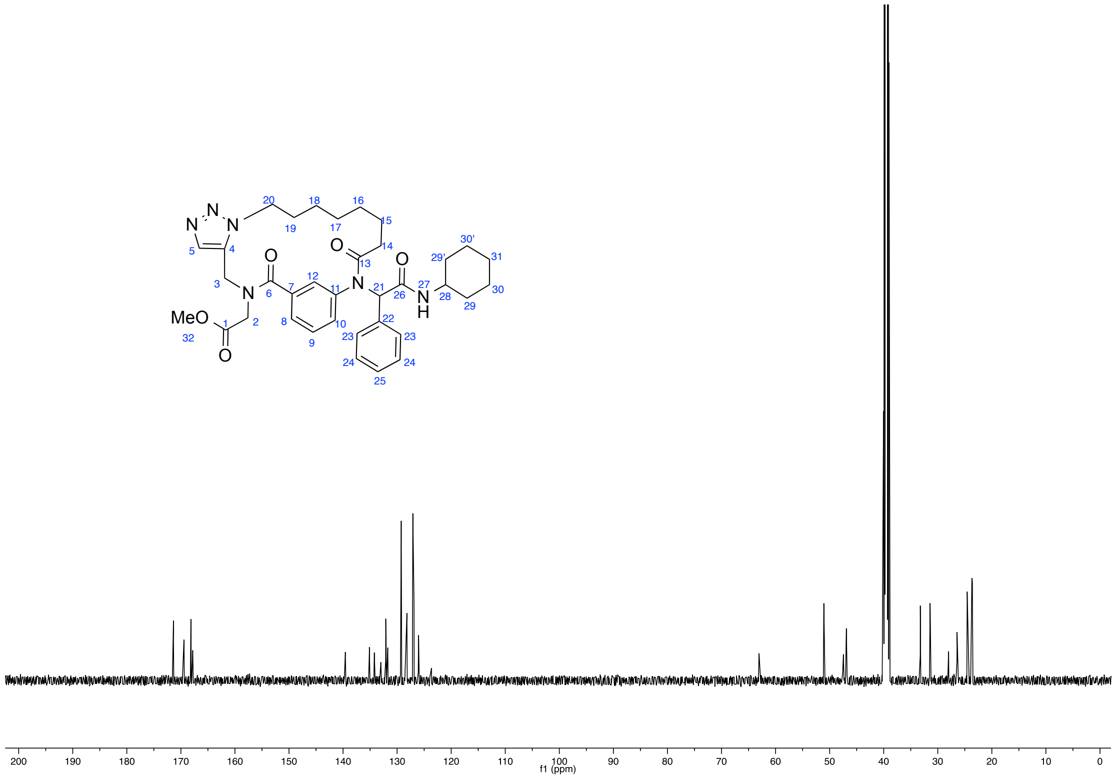
MeO
f1 (ppm)

44a

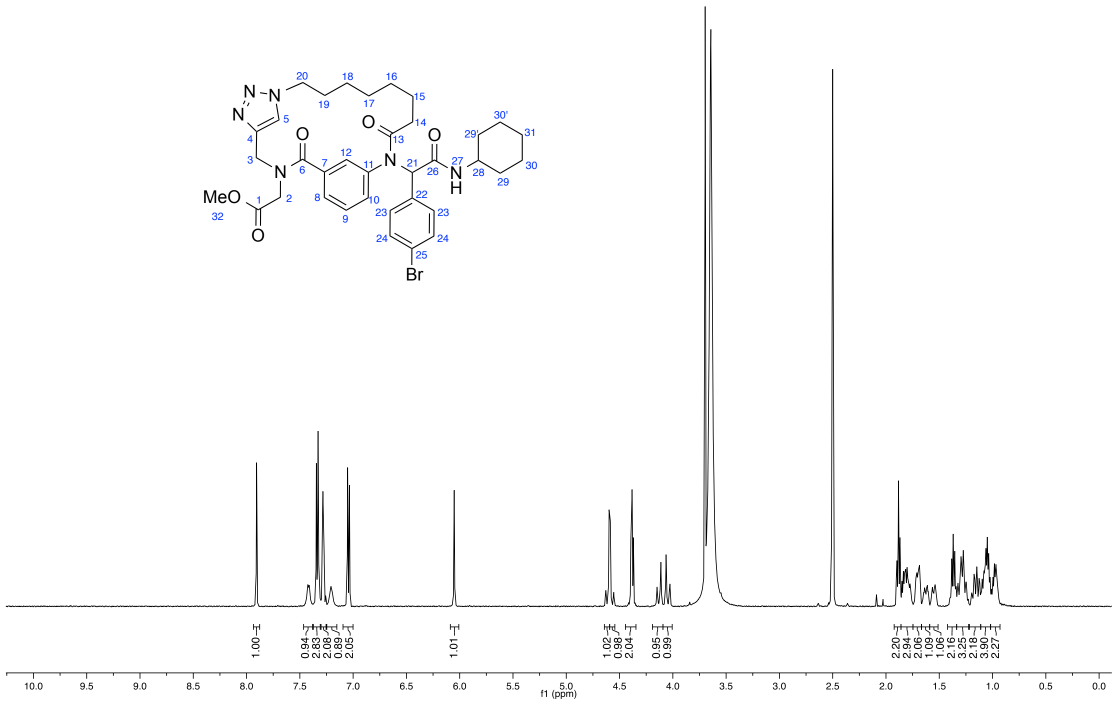

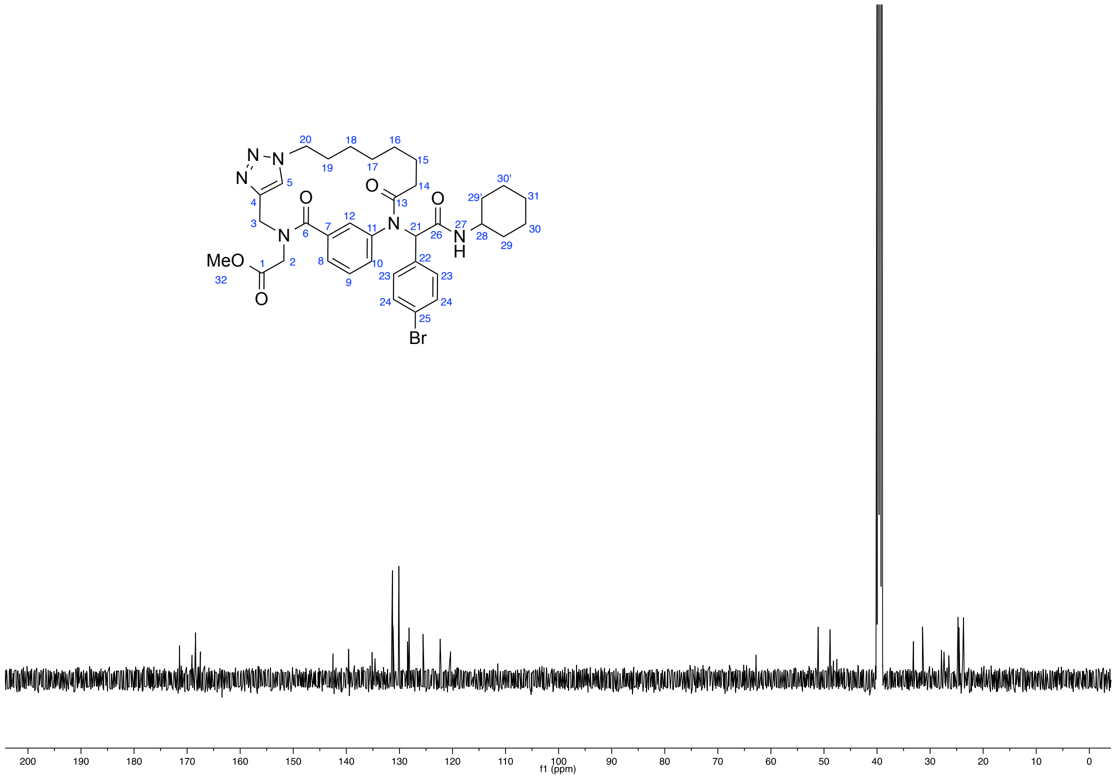

44b

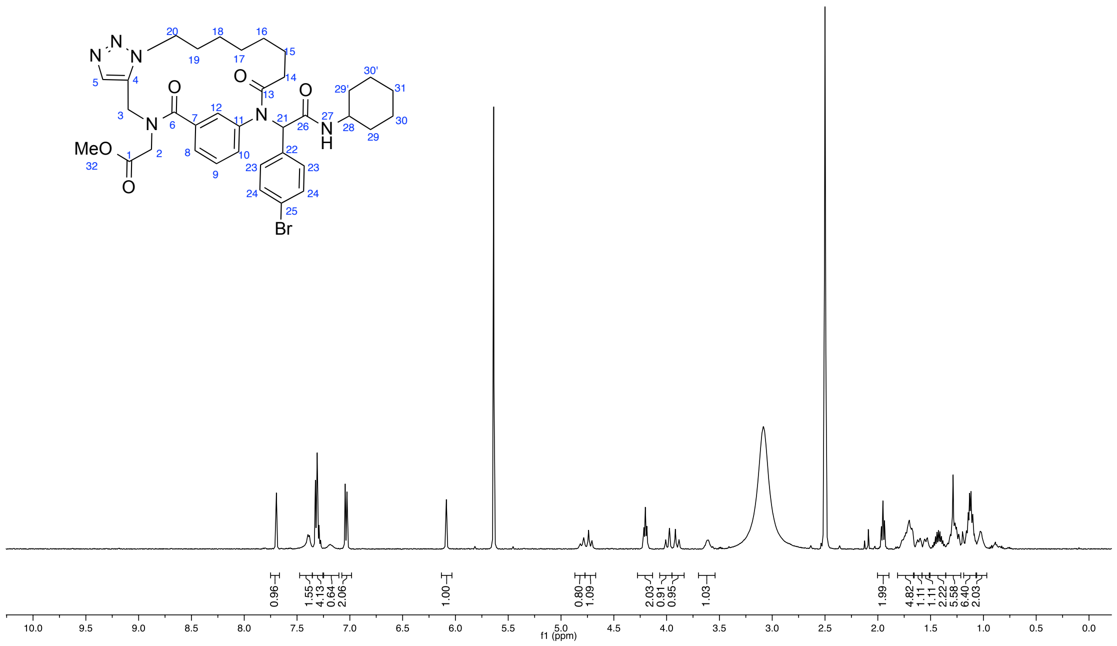

MeO
Br
0.96
1.55
4.13
0.64
2.06
1.00
0.80
1.09
2.03
0.91
0.95
1.03
1.99
4.82
1.11
1.11
2.22
5.58
6.40
2.03
10.0 9.5 9.0 8.5 8.0 7.5 7.0 6.5 6.0 5.5 5.0 4.5 4.0 3.5 3.0 2.5 2.0 1.5 1.0 0.5 0.0
f1 (ppm)

45a

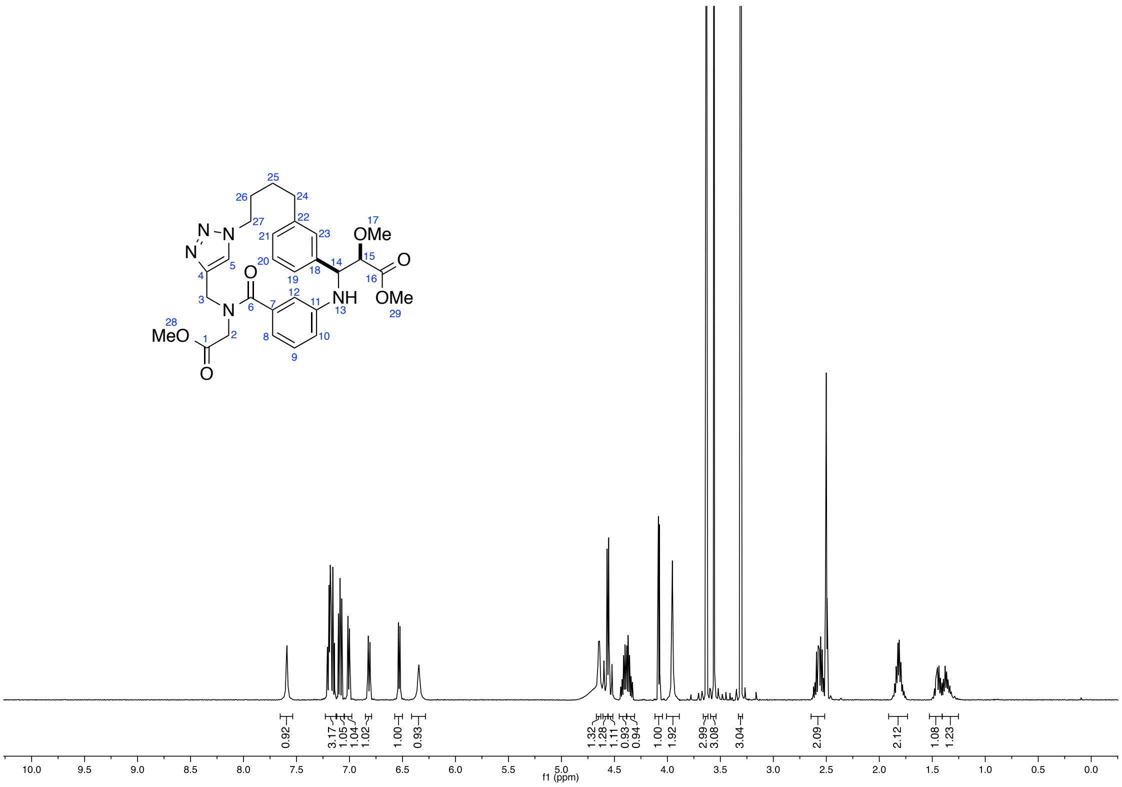

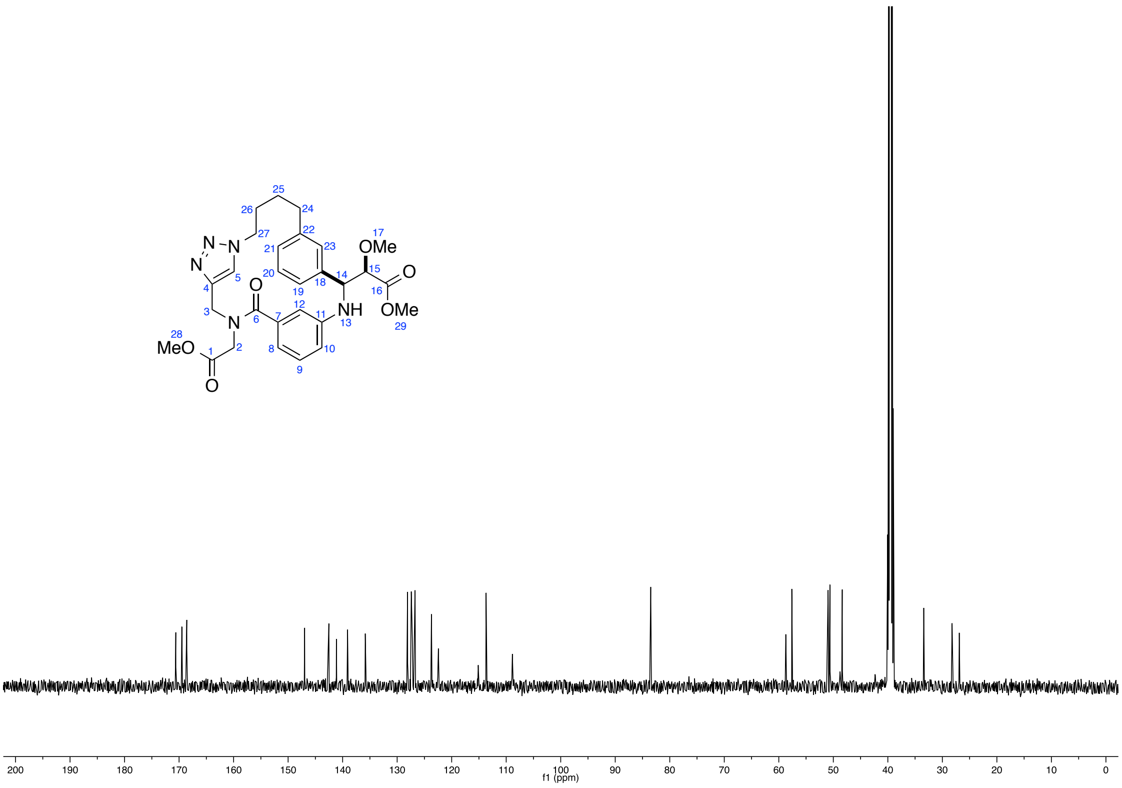

45b

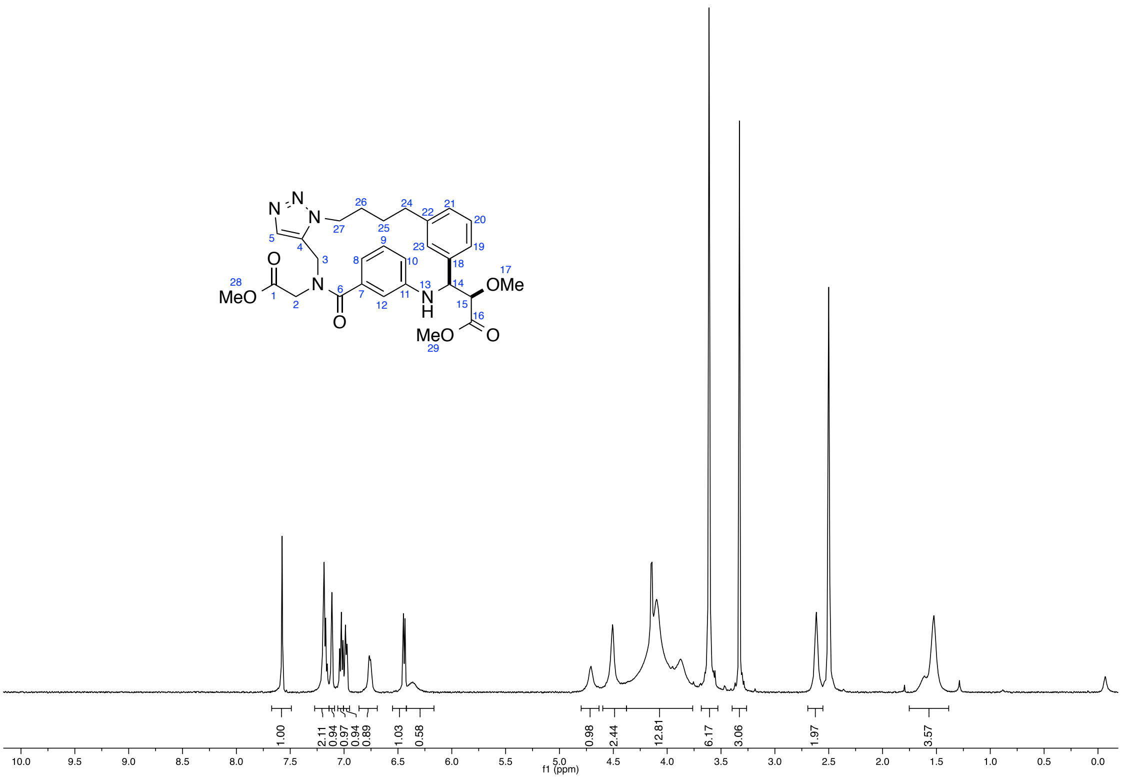
f1 (ppm)
1.00
2.11
0.94
0.97
0.94
0.89
1.03
0.58
0.98
2.44
12.81
6.17
3.06
1.97
3.57

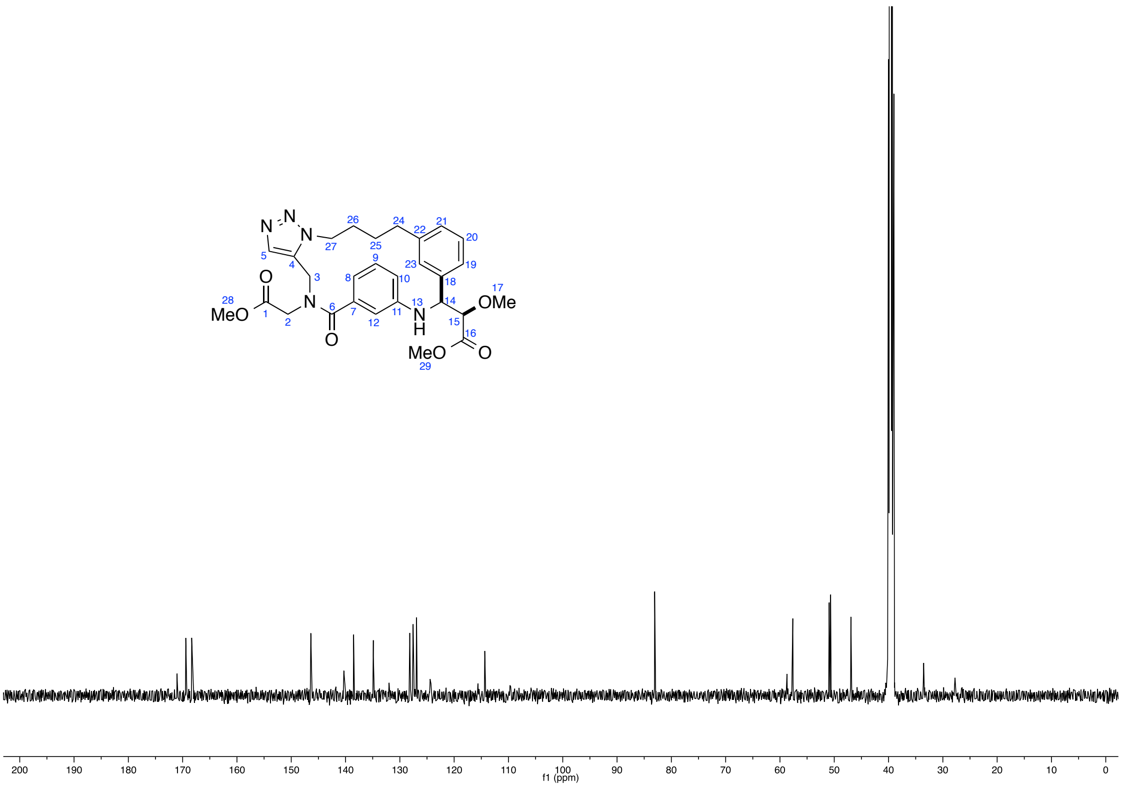
f1 (ppm)

46a

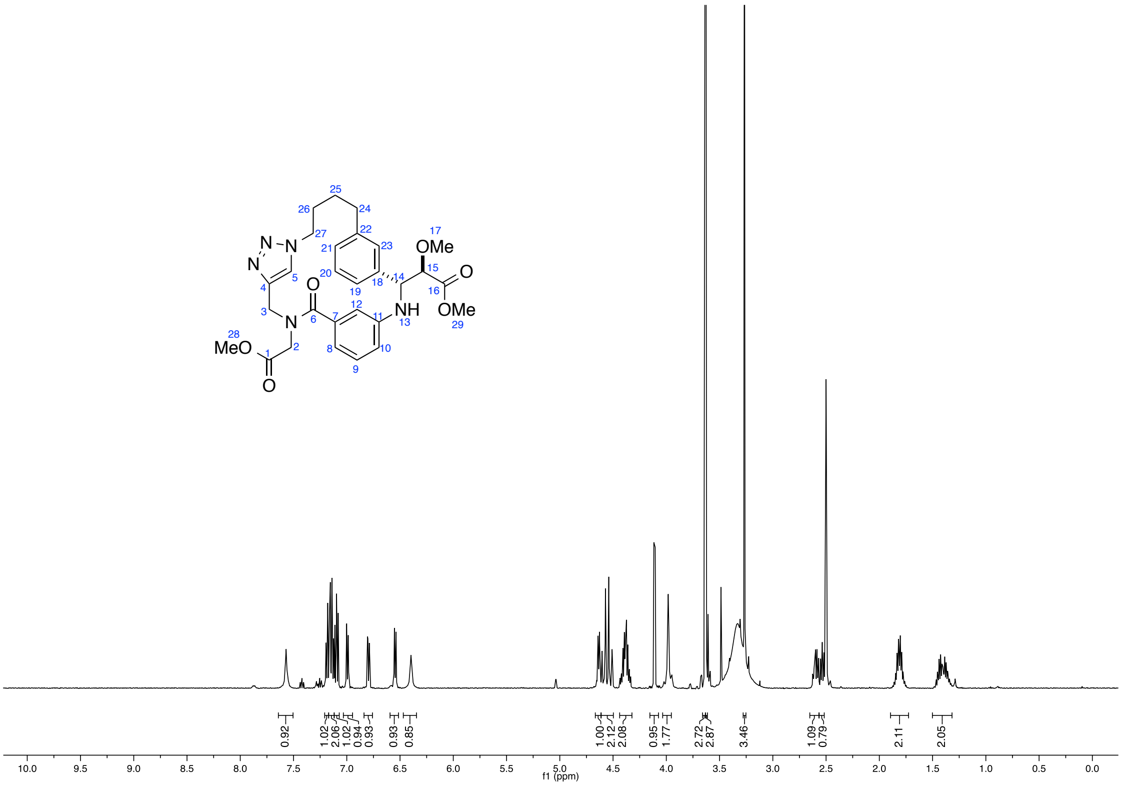

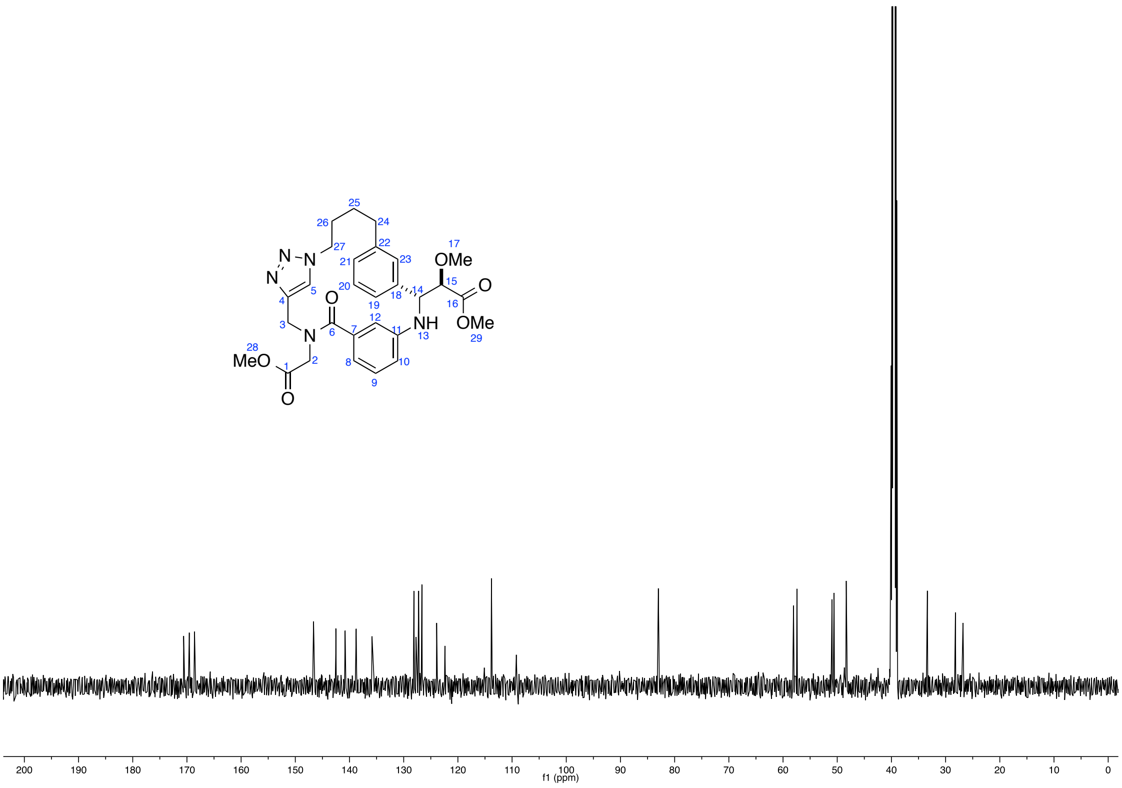

47

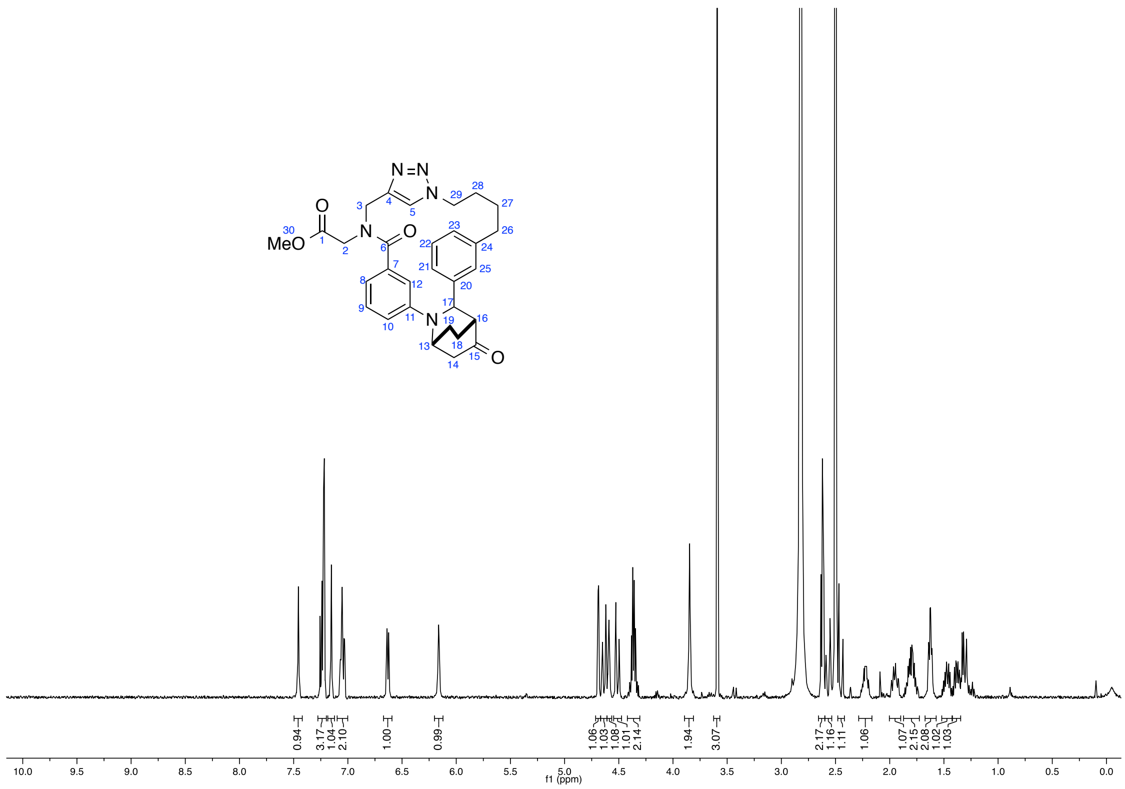

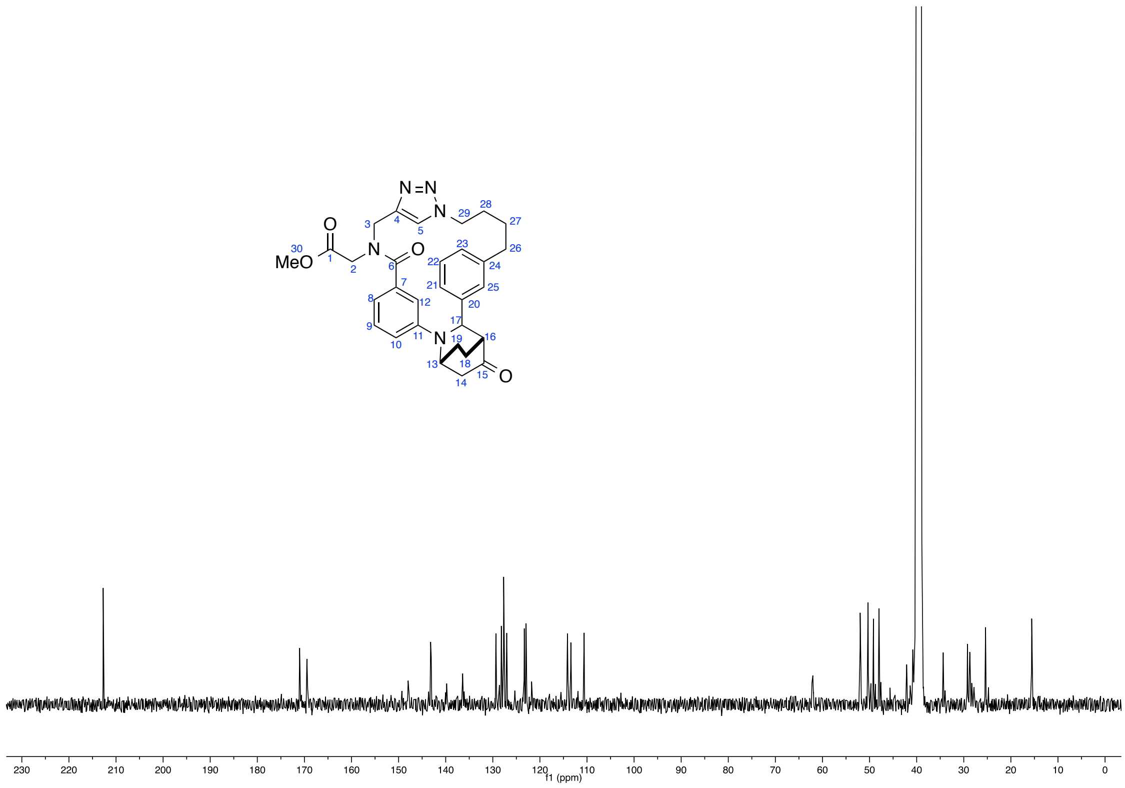

49e

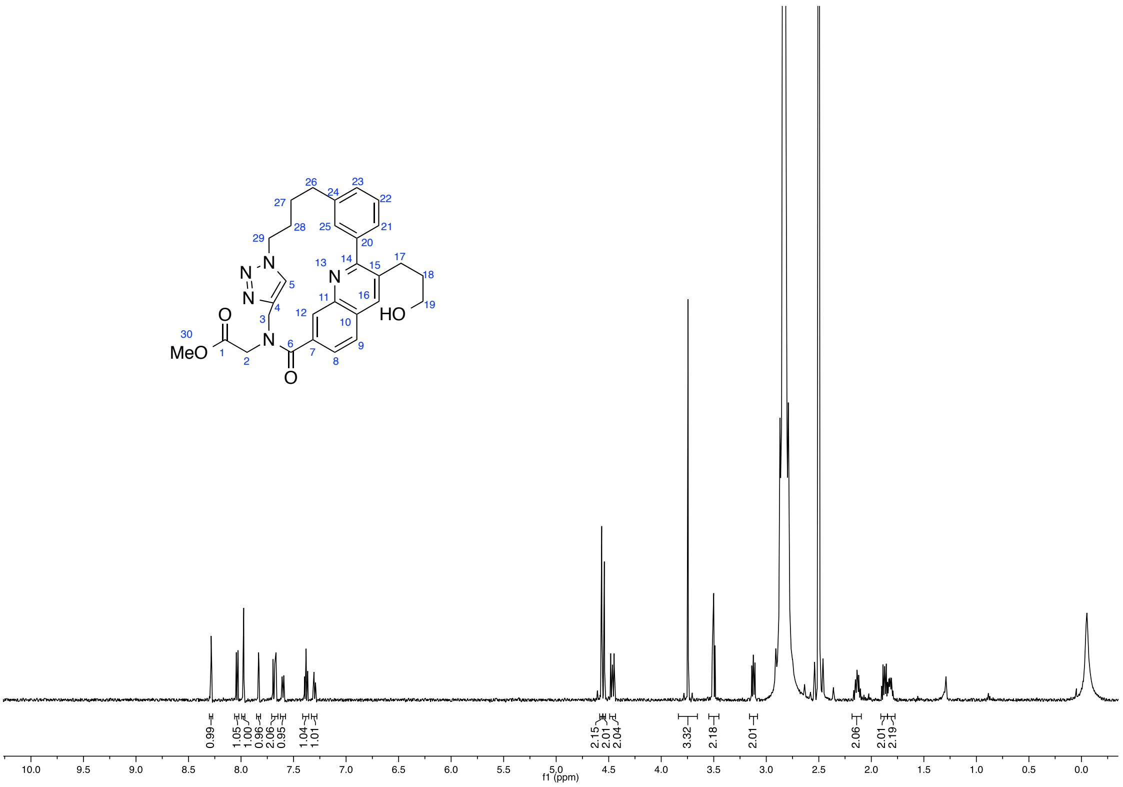
0.99
1.05
1.00
0.96
2.06
0.95
1.04
1.01
2.15
2.01
2.04
3.32
2.18
2.01
2.06
2.01
2.19
f1 (ppm)

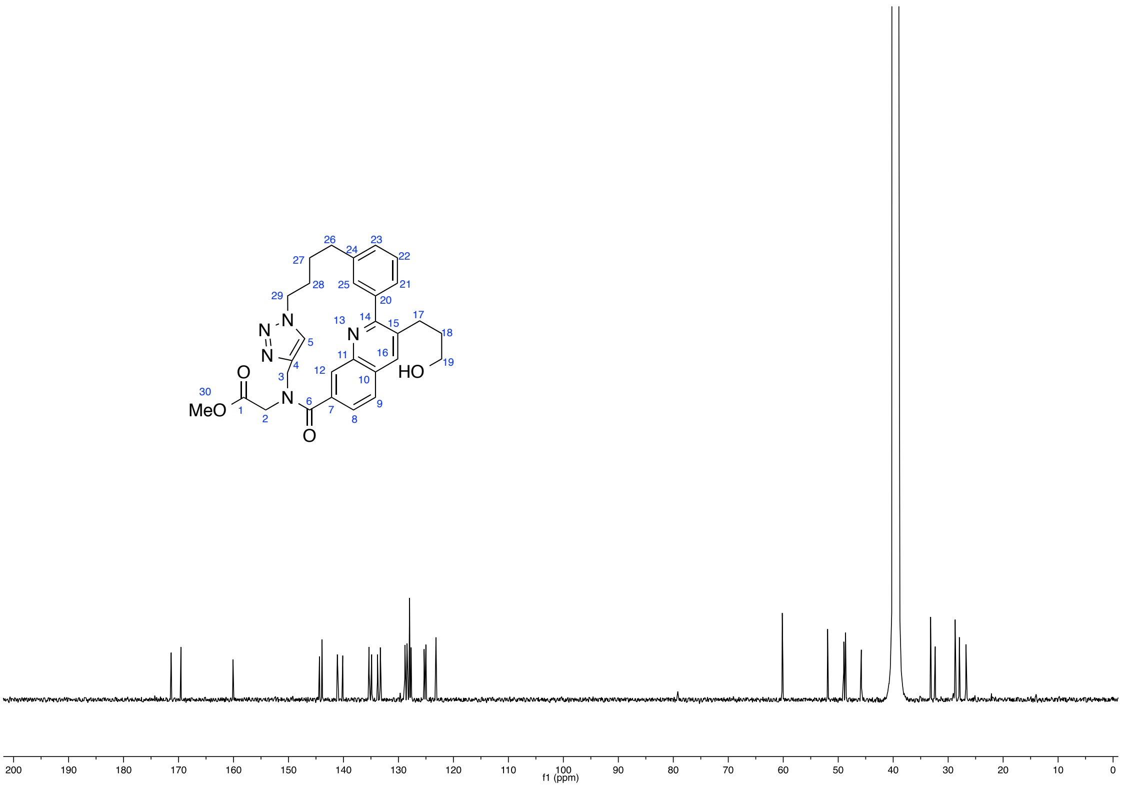
f1 (ppm)

49f

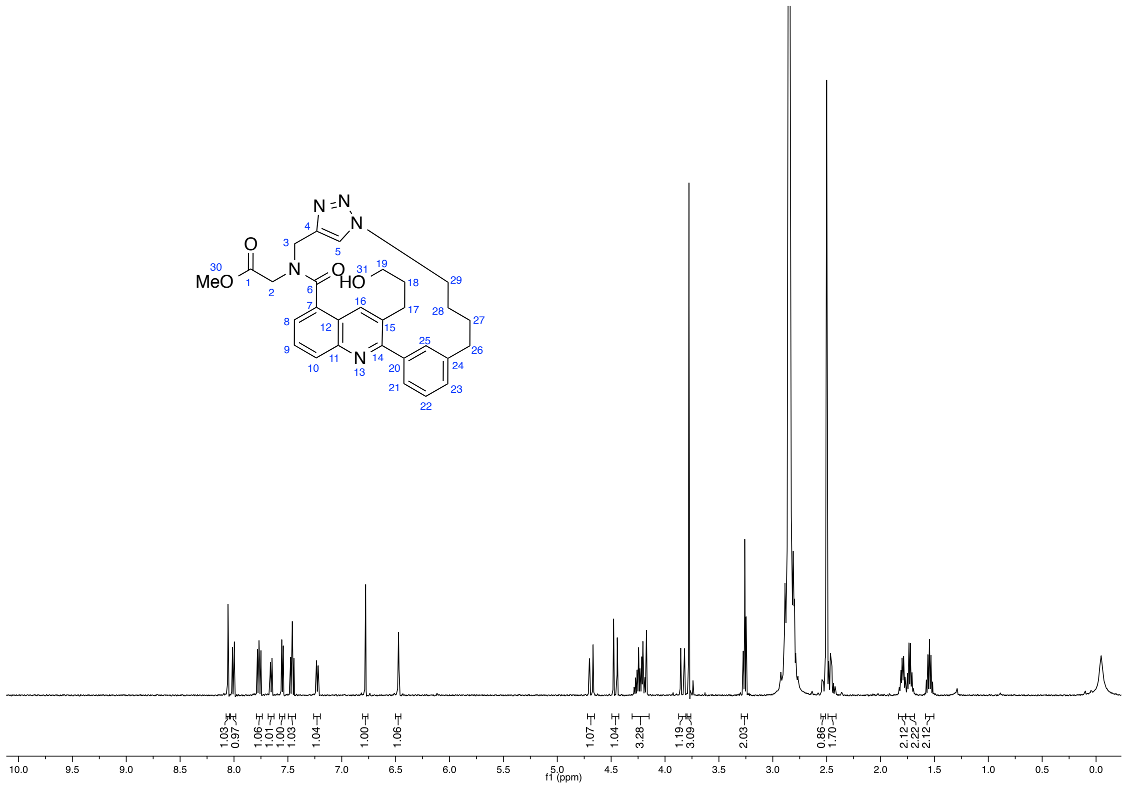

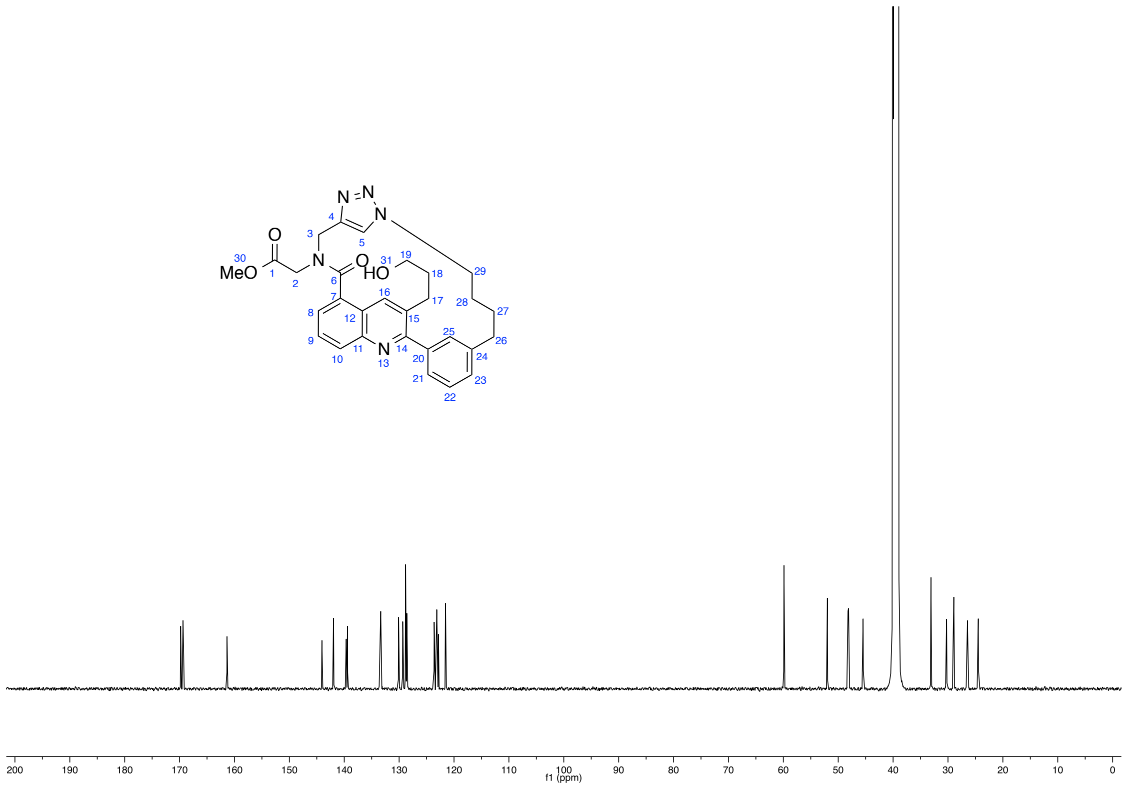

49a

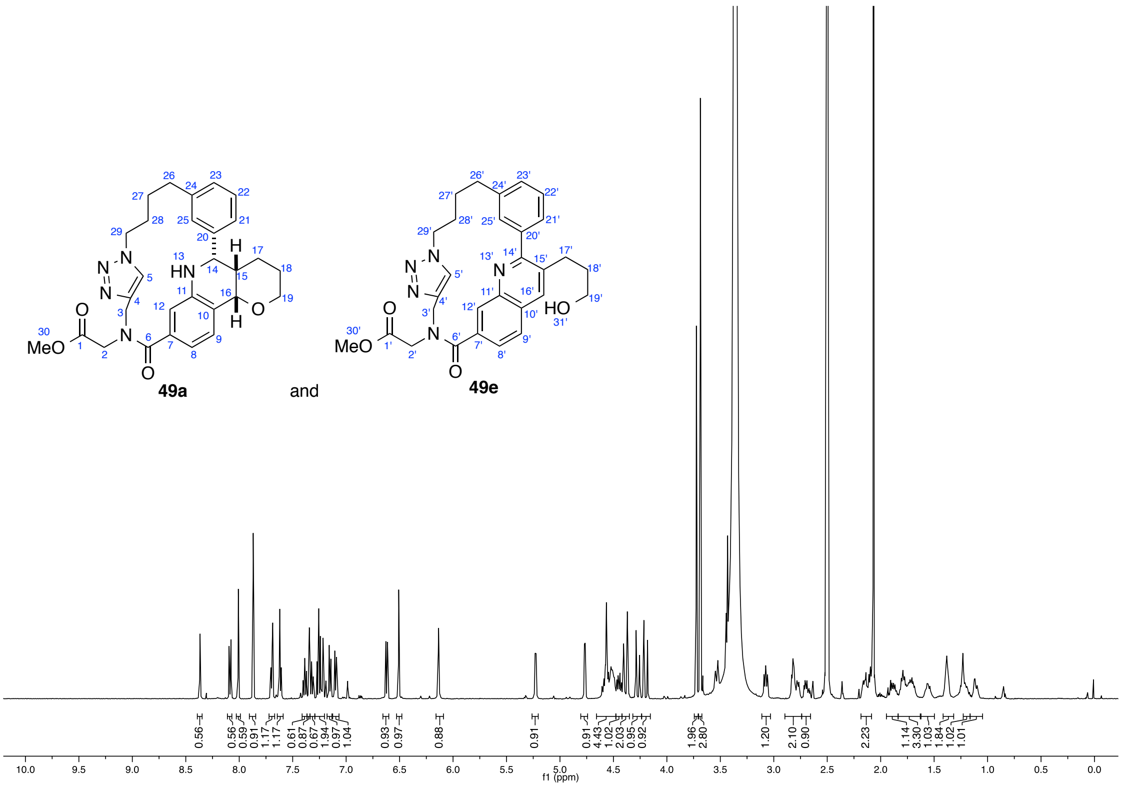

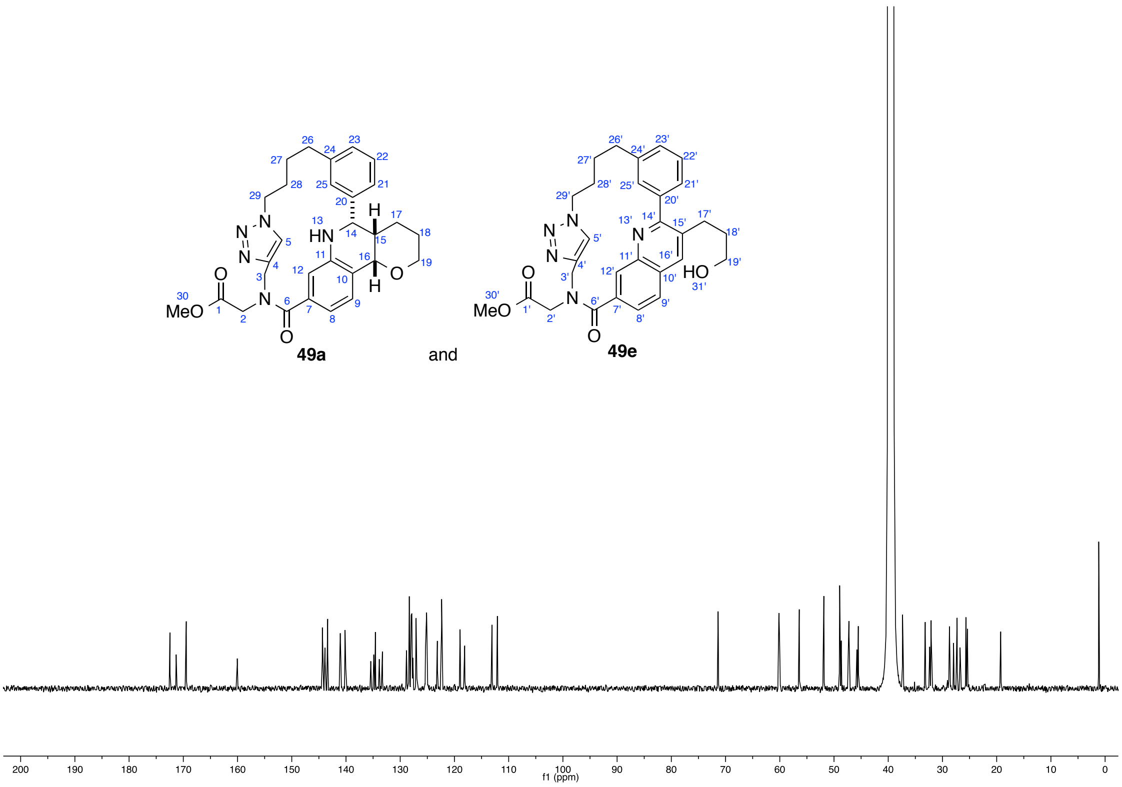

49b

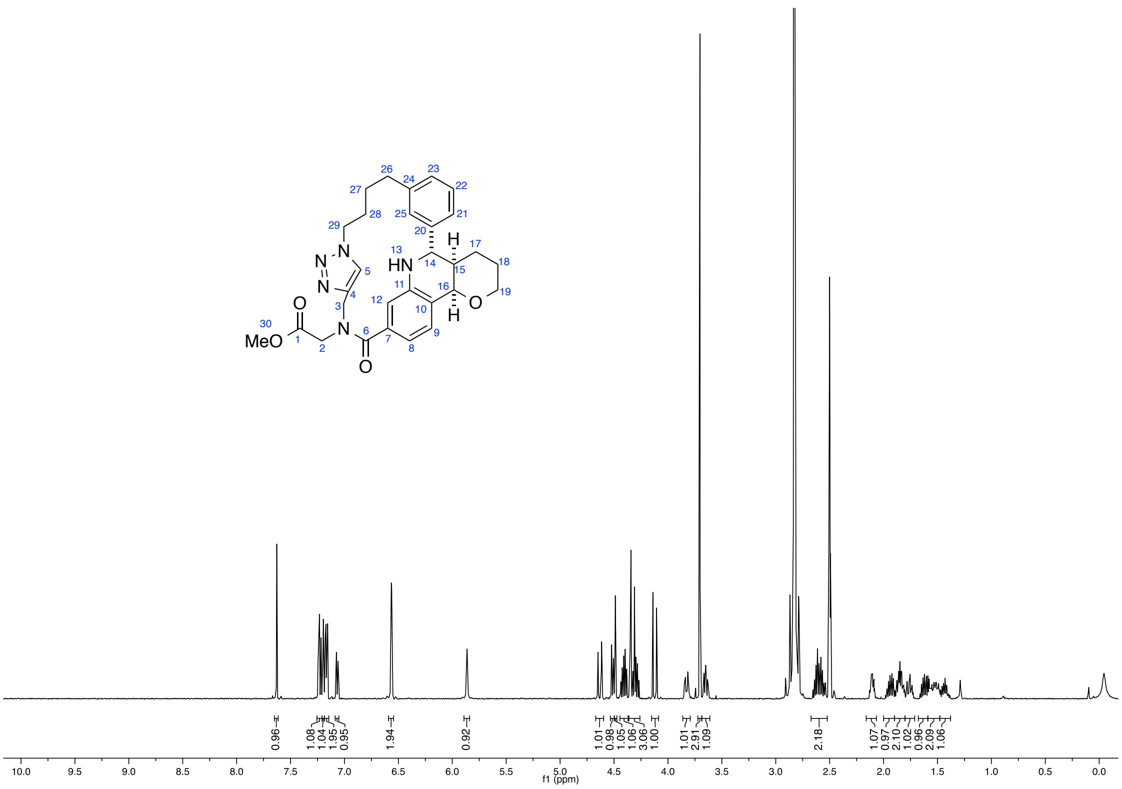

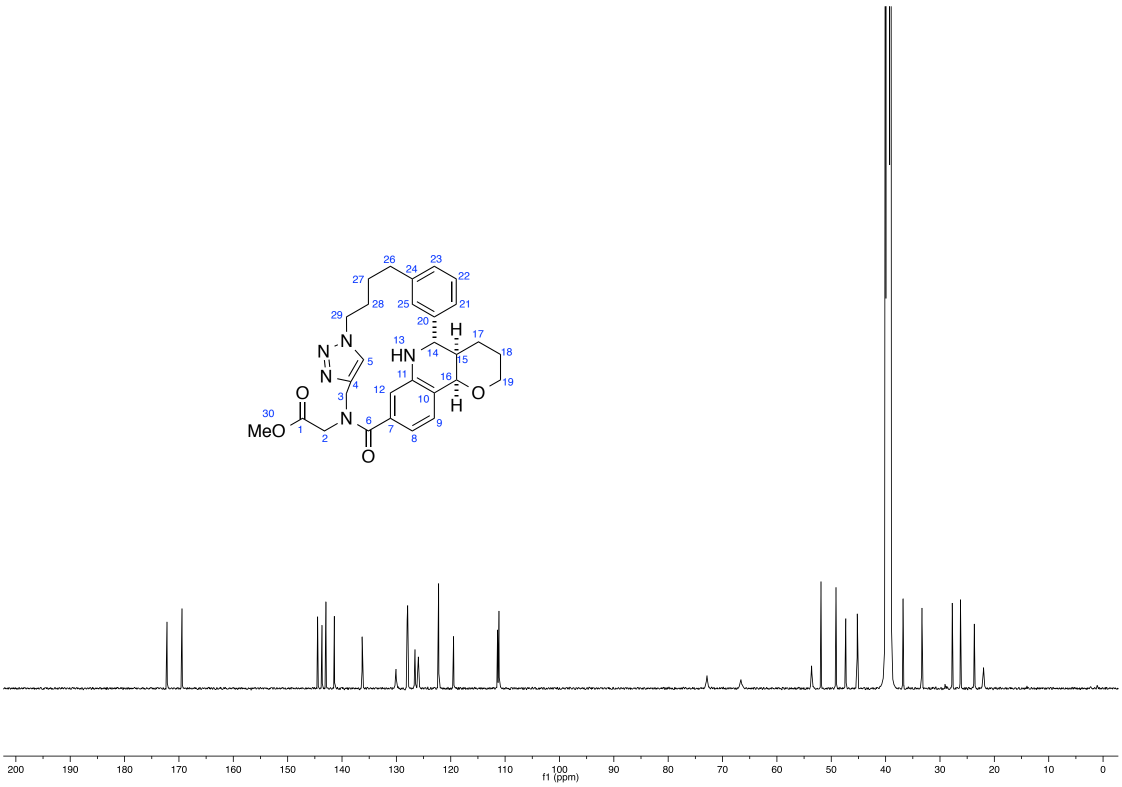

49d

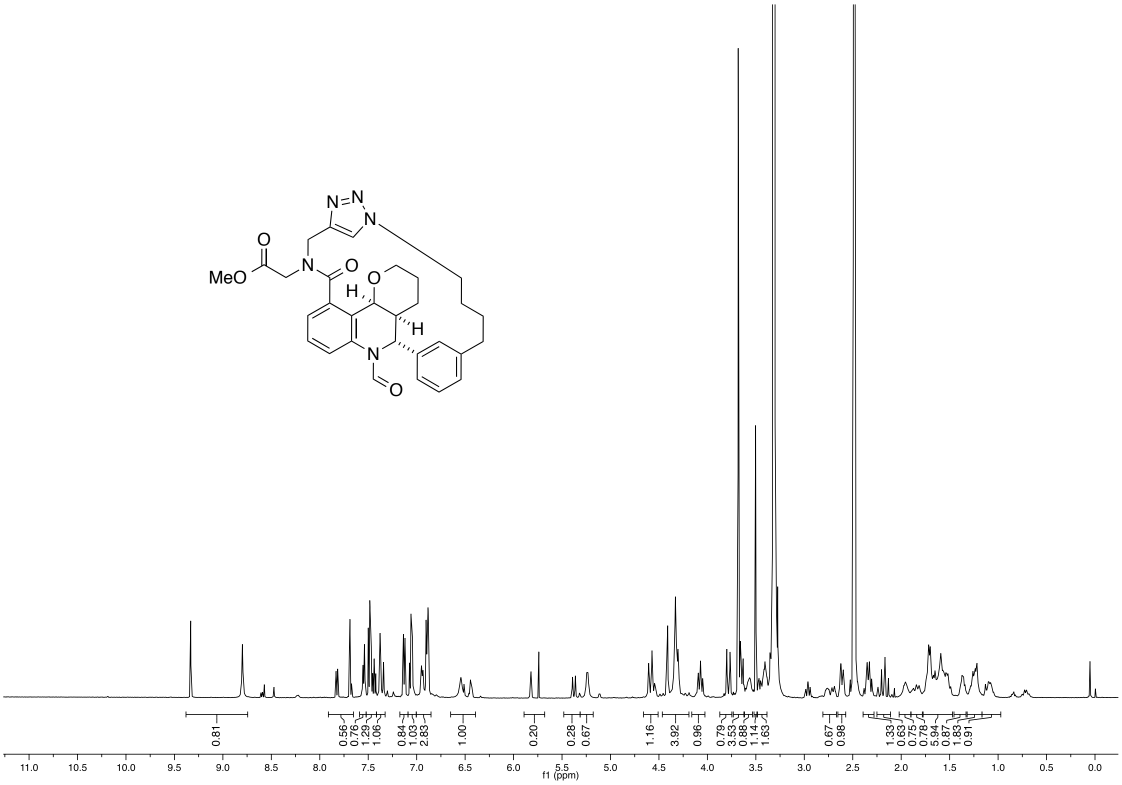

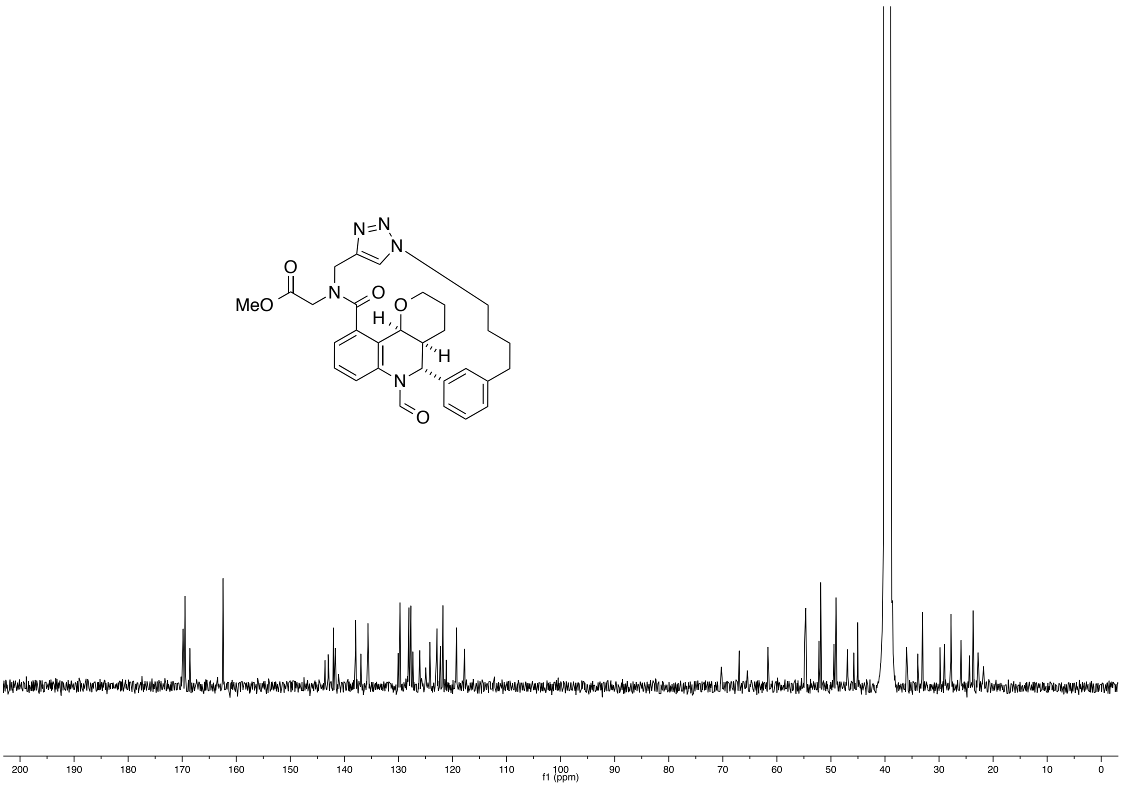

51

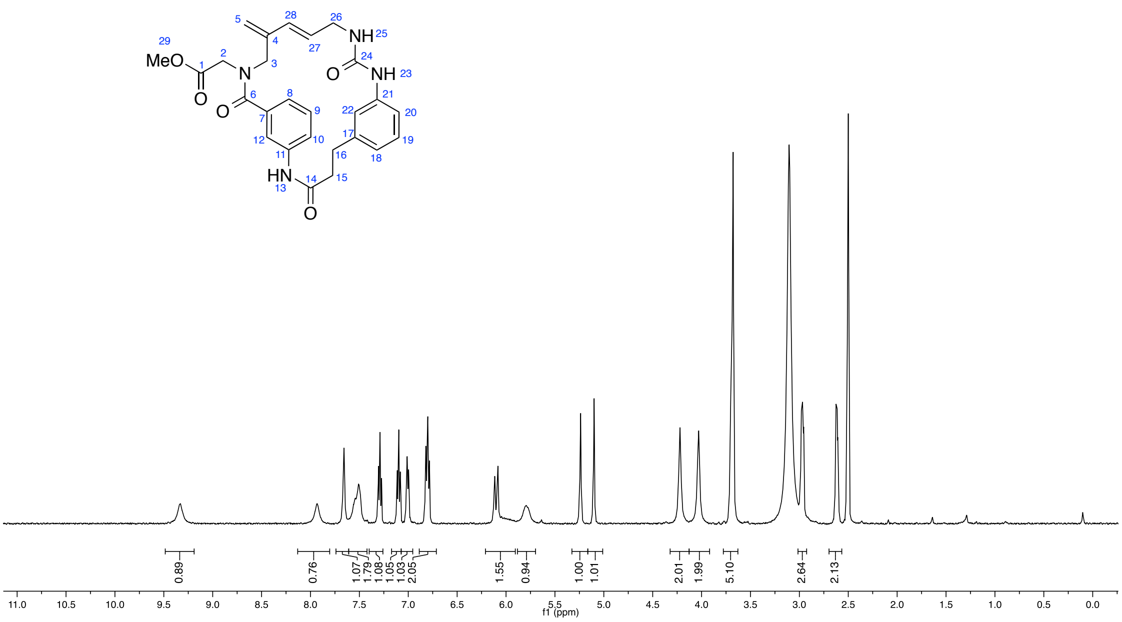
MeO
N
NH
NH
HN
O
0.89
0.76
1.07
1.79
1.08
1.05
1.03
2.05
1.55
0.94
1.00
1.01
2.01
1.99
5.10
2.64
2.13
f1 (ppm)

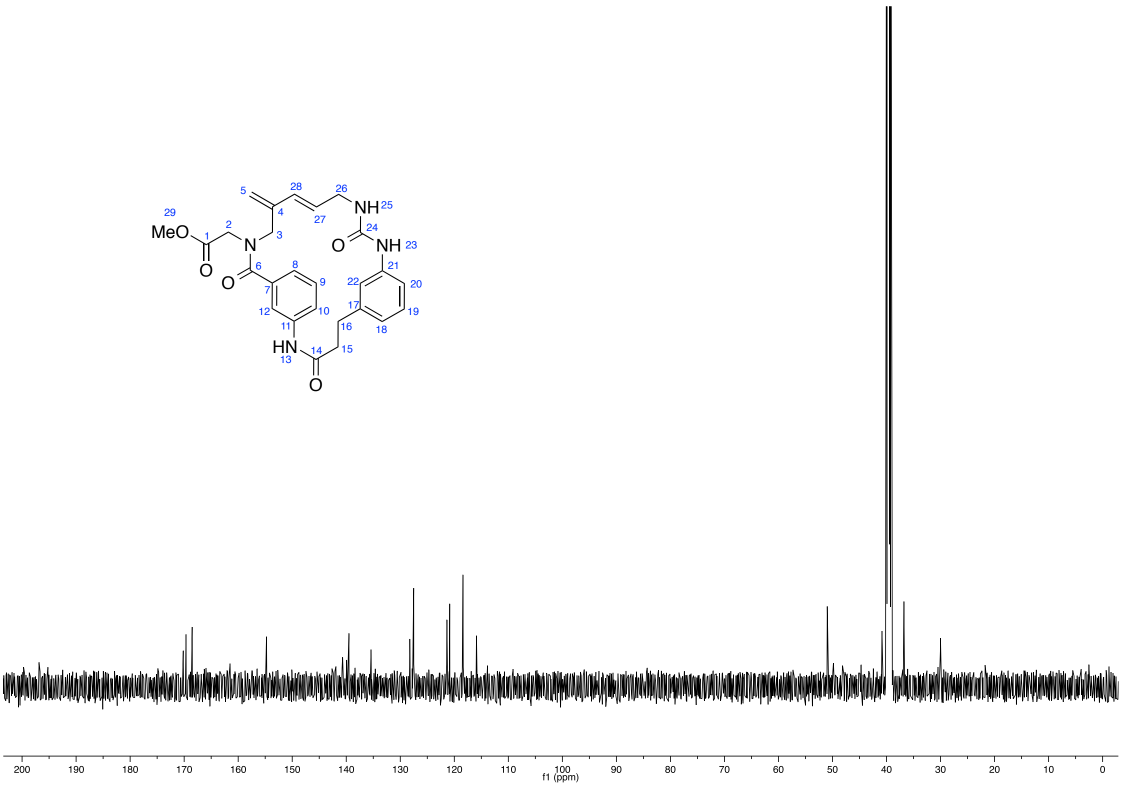
MeO
N
NH
NH
HN
O
f1 (ppm)

52a

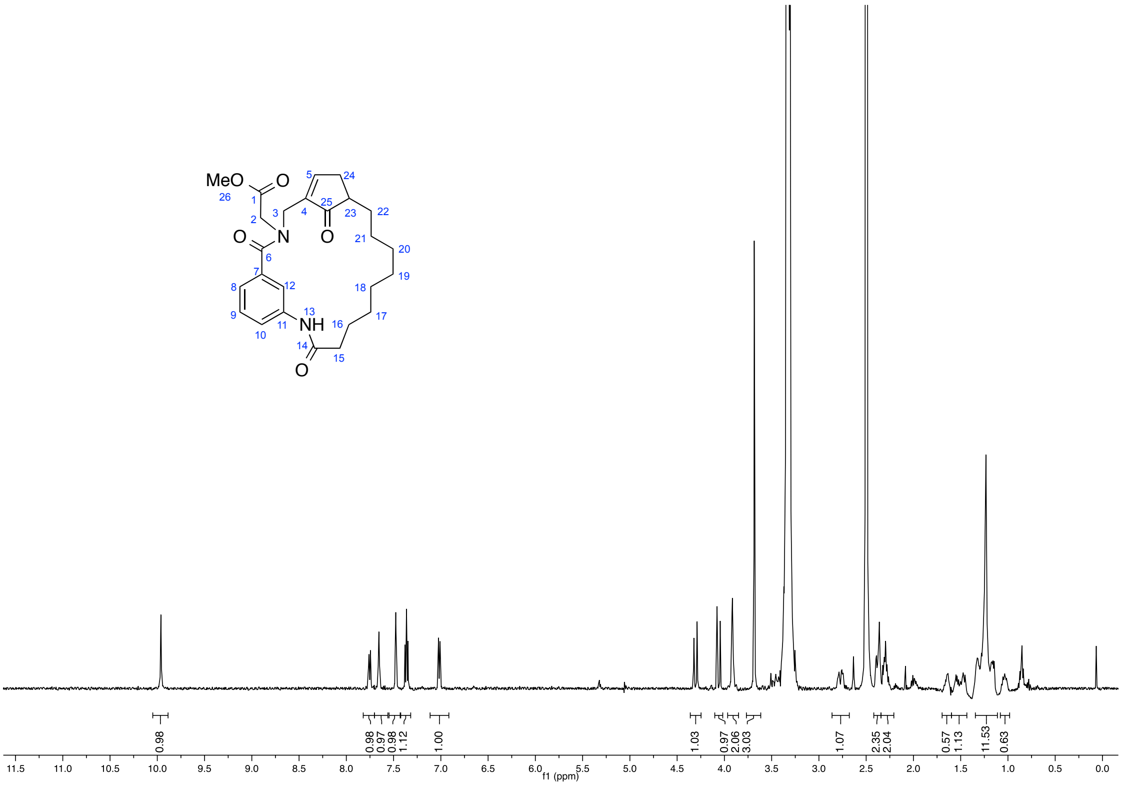
MeO
O
N
NH
0.98
0.98
0.97
0.98
1.12
1.00
1.03
0.97
2.06
3.03
1.07
2.35
2.04
0.57
1.13
11.53
0.63
f1 (ppm)

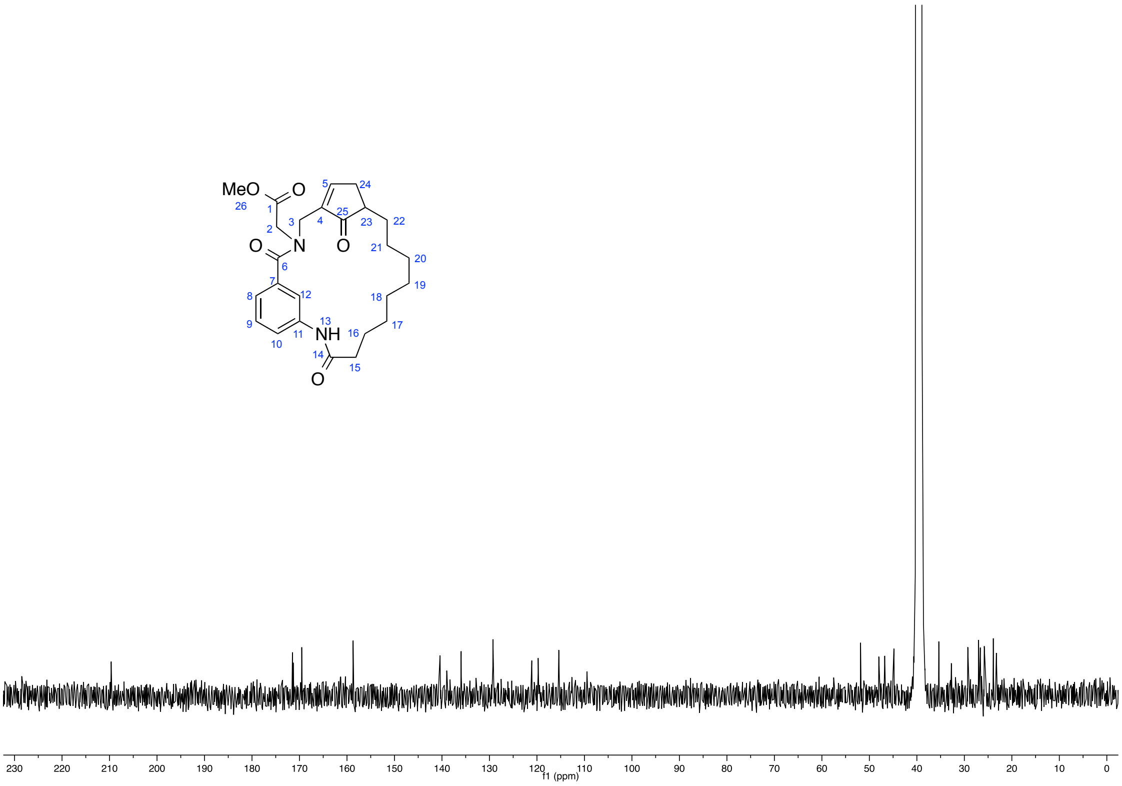
MeO
O
N
NH
f1 (ppm)

52b

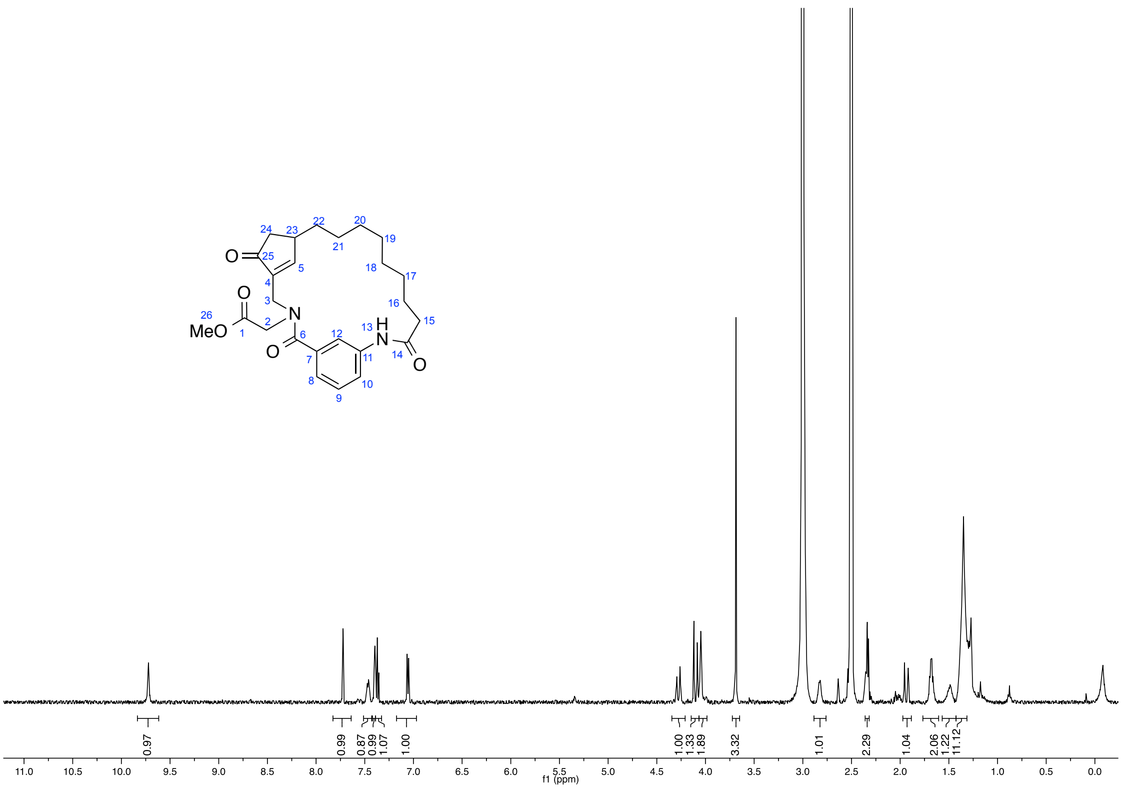

53a

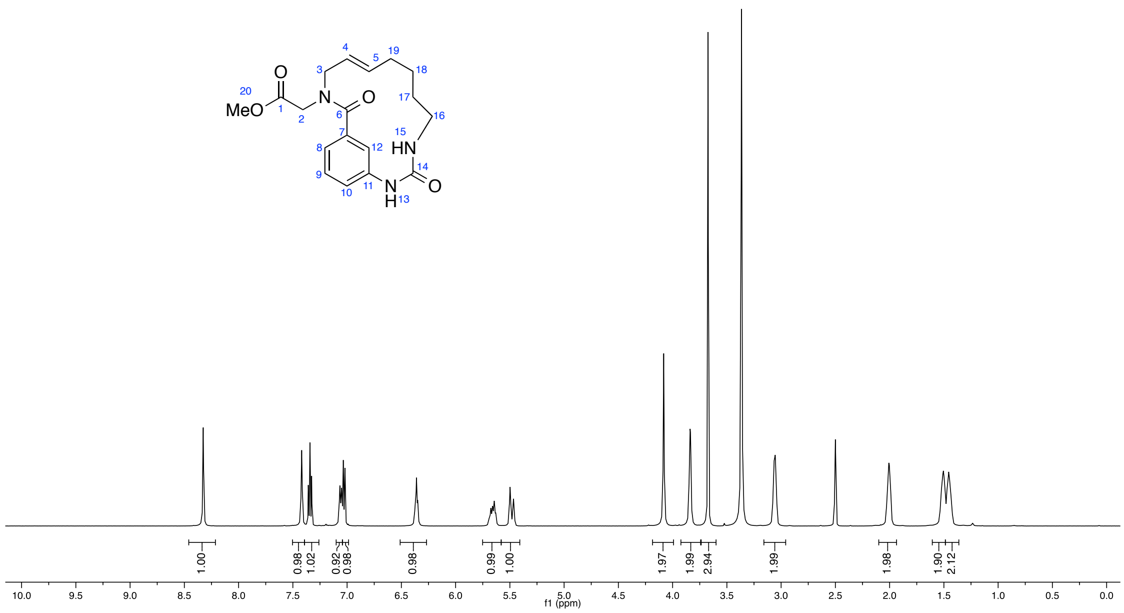
MeO
HN
N
H
1.00
0.98
1.02
0.92
0.98
0.98
0.99
1.00
1.97
1.99
2.94
1.99
1.98
1.90
2.12
f1 (ppm)

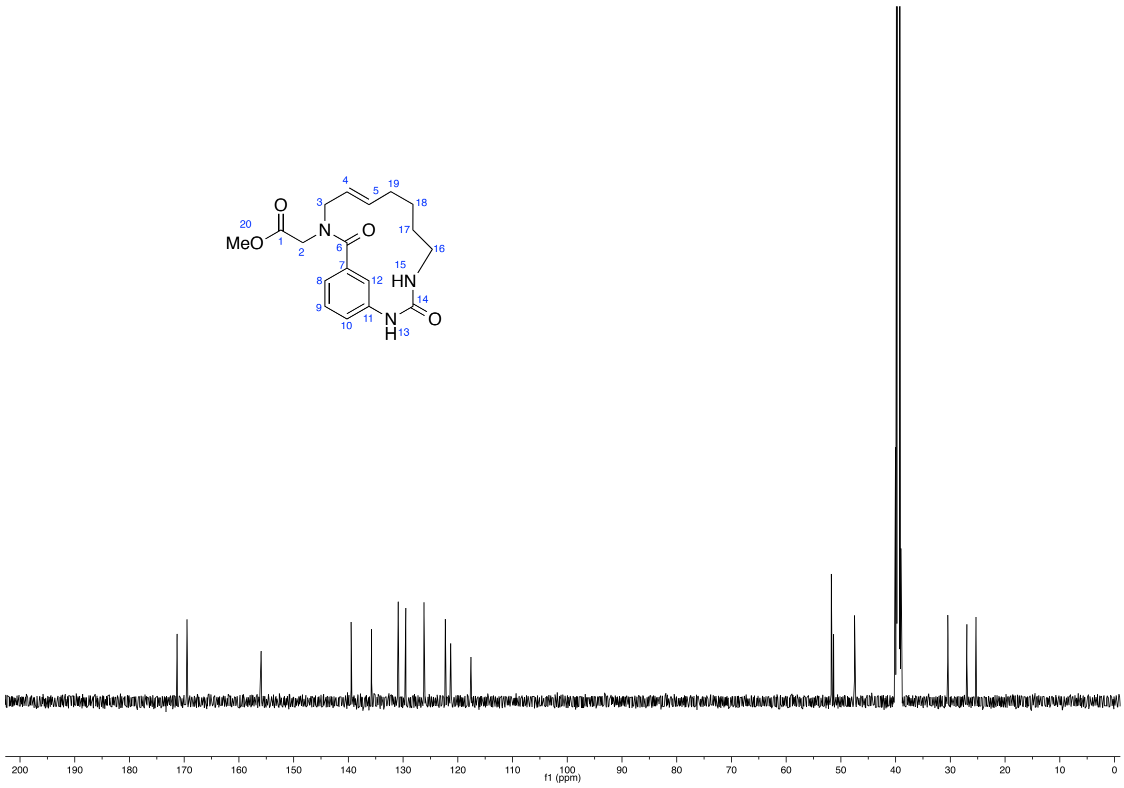
MeO
HN
N
H
f1 (ppm)

53b

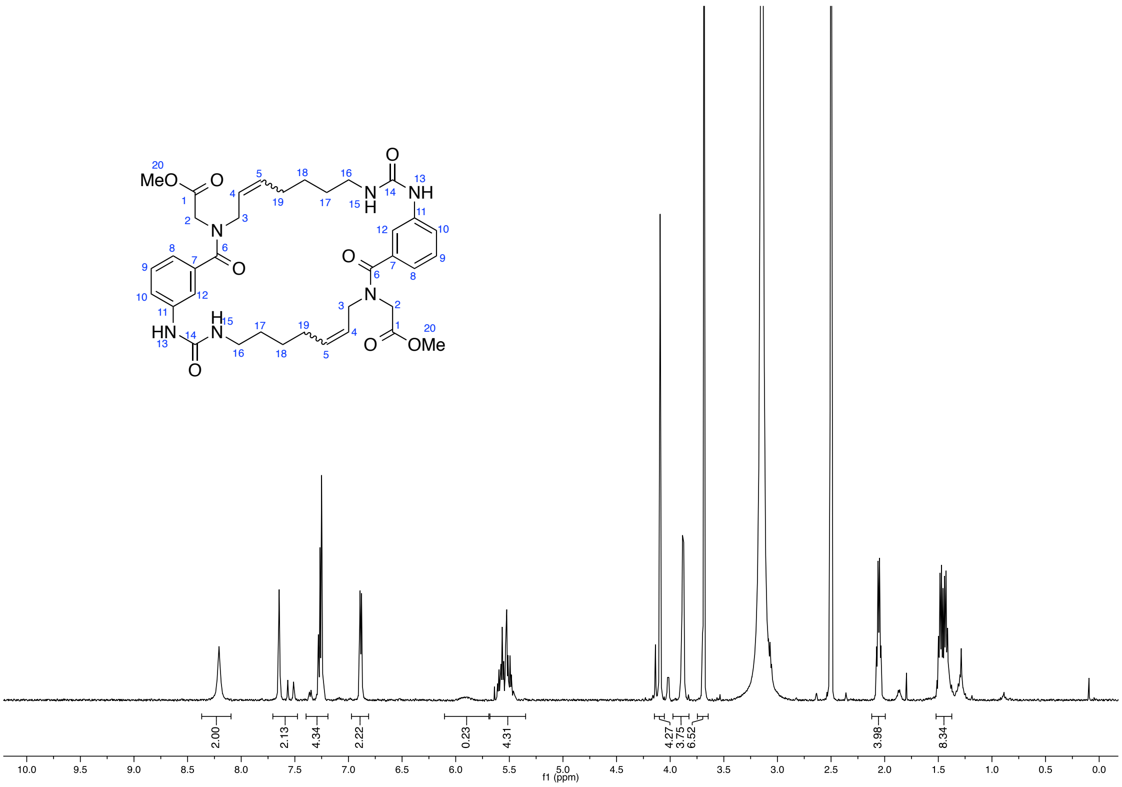

MeO
O
O
N
NH
HN
N
H
f1 (ppm)
2.00
2.13
4.34
2.22
0.23
4.31
4.27
3.75
6.52
3.98
8.34

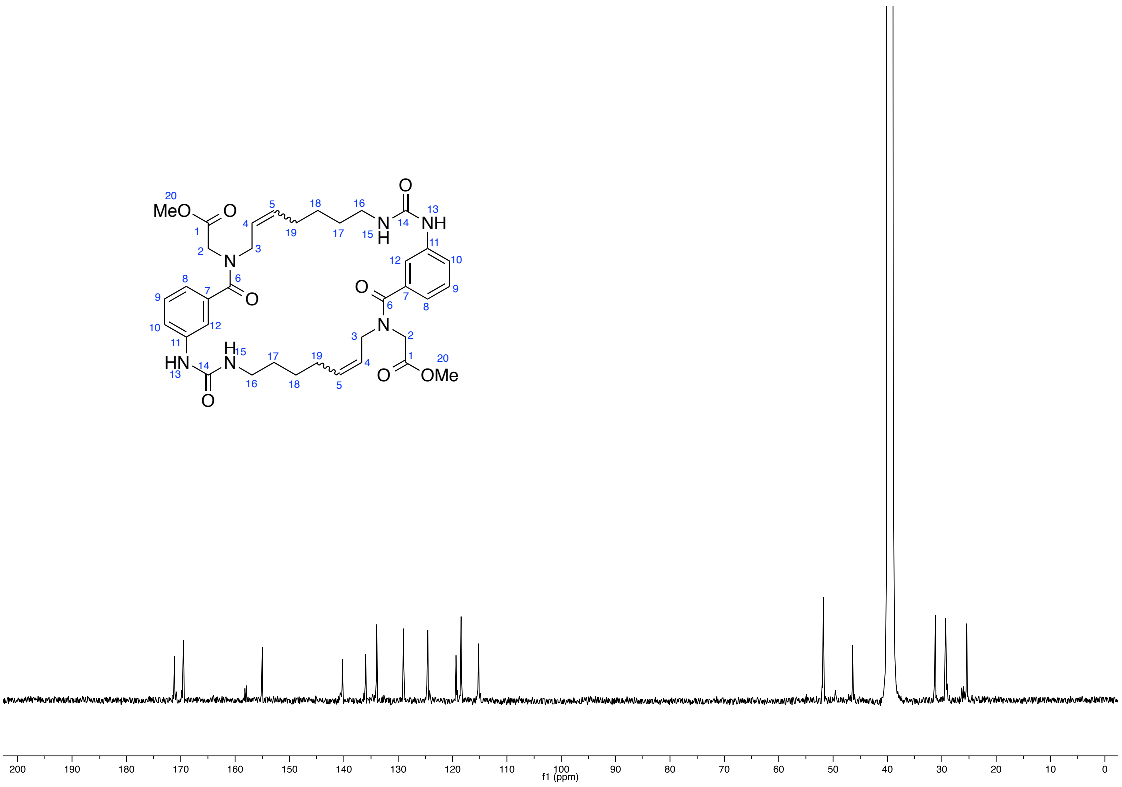

MeO
OMe
f1 (ppm)

54a

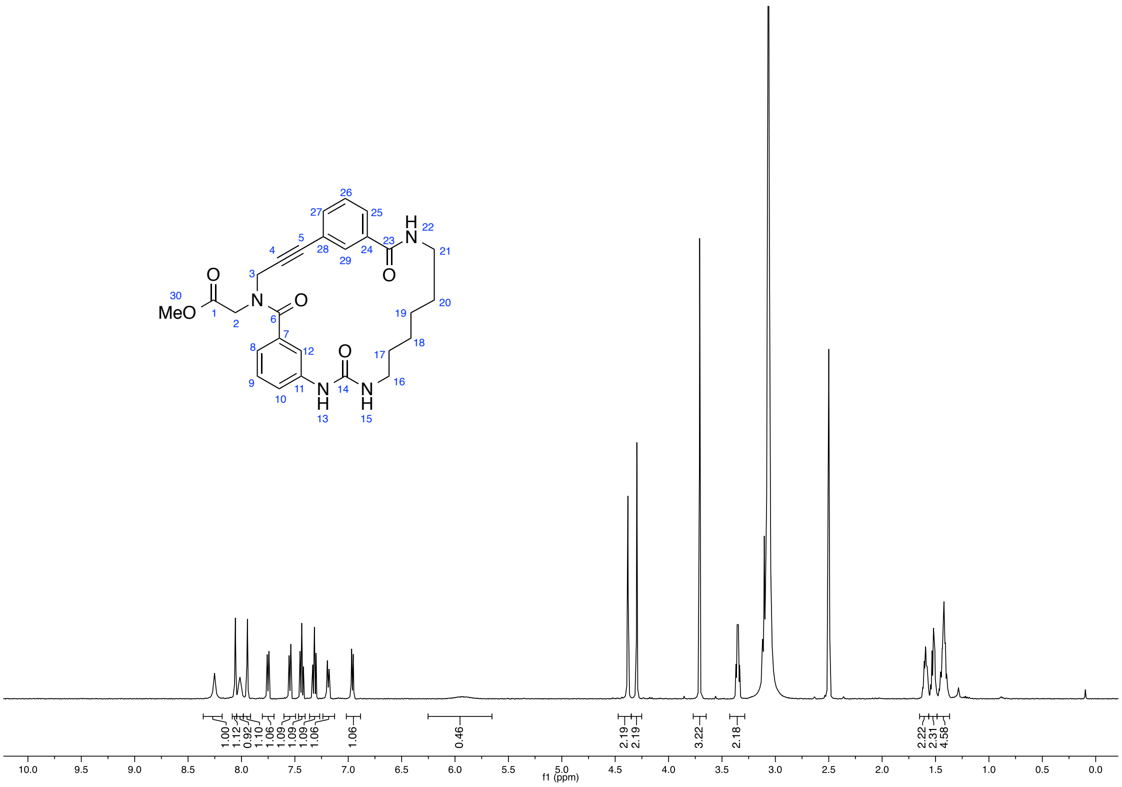

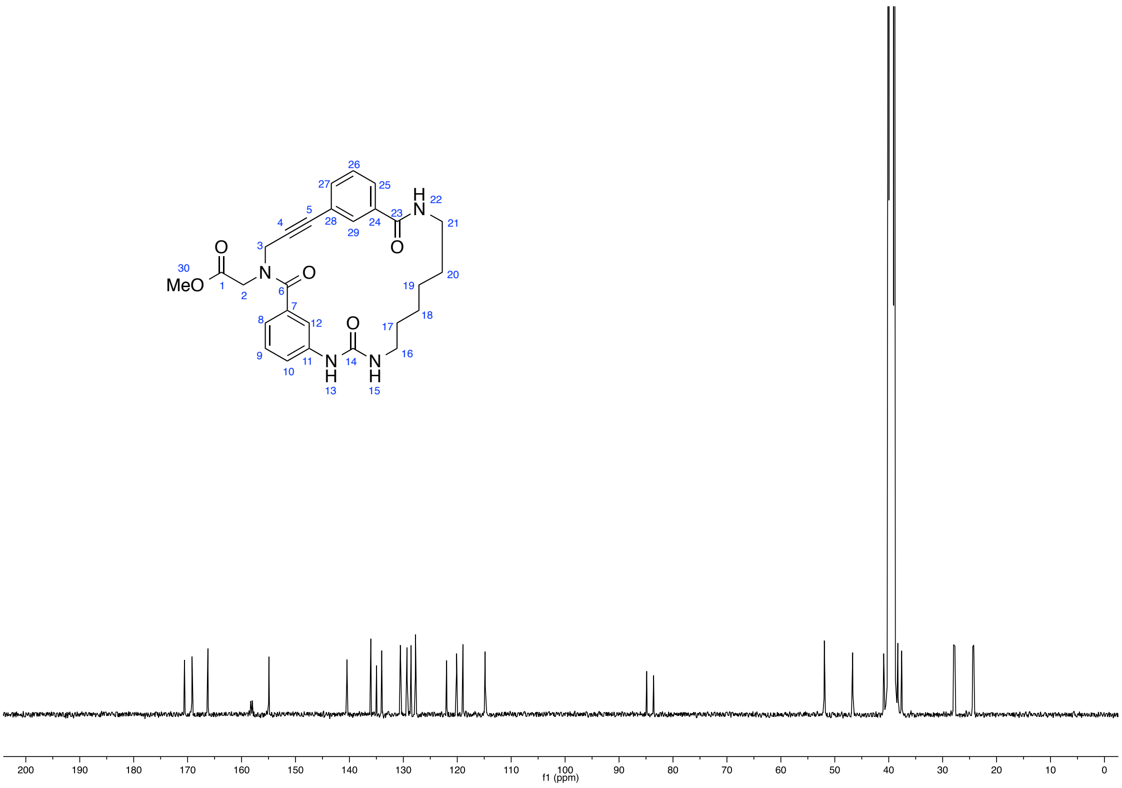

54b

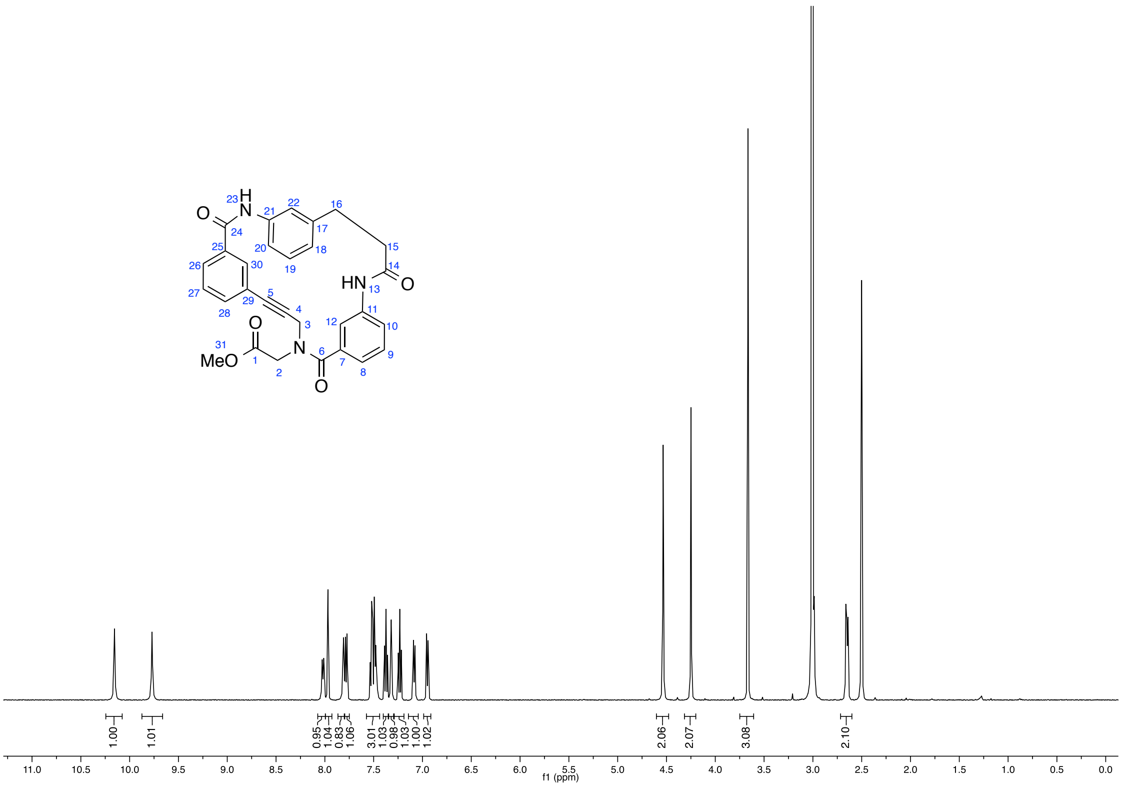
MeO
HN
f1 (ppm)
1.00
1.01
0.95
1.04
0.83
1.06
3.01
1.03
0.98
1.03
1.00
1.02
2.06
2.07
3.08
2.10
11.0 10.5 10.0 9.5 9.0 8.5 8.0 7.5 7.0 6.5 6.0 5.5 5.0 4.5 4.0 3.5 3.0 2.5 2.0 1.5 1.0 0.5 0.0

55

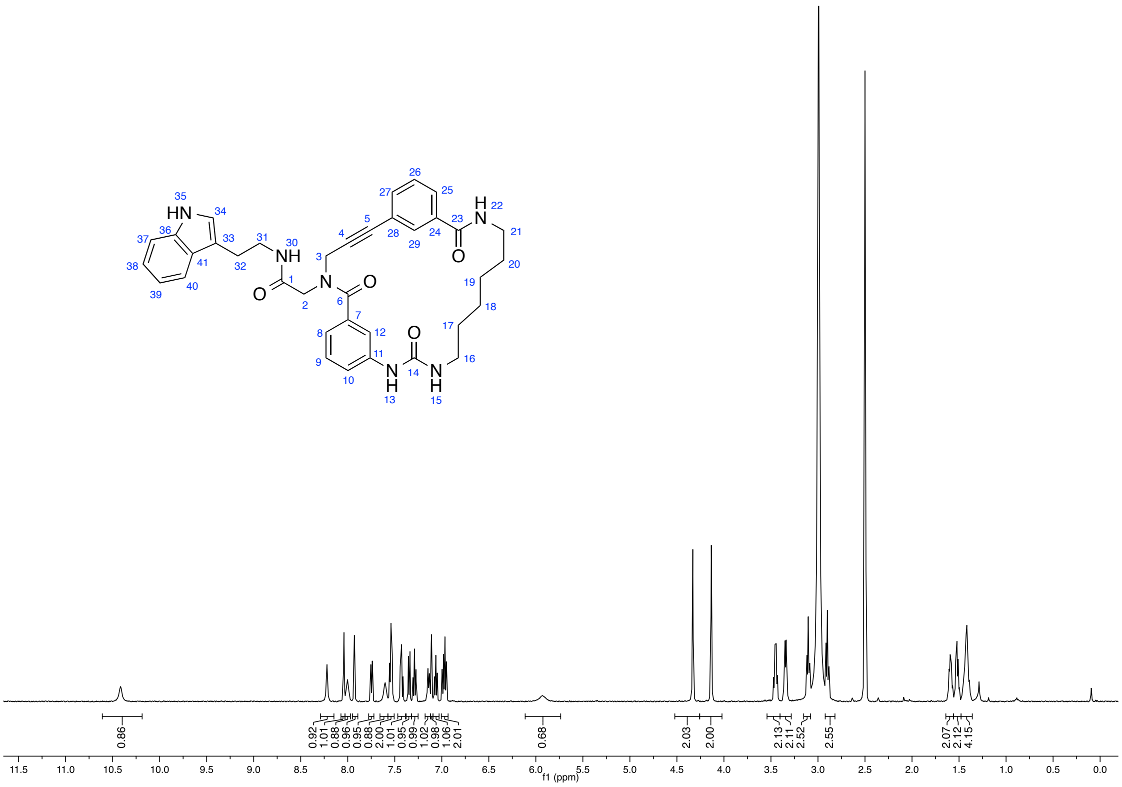
HN
NH
O
N
O
O
N
H
O
N
H
H
N
O
0.86
0.92
1.01
0.88
0.96
0.95
0.88
2.00
1.01
0.95
0.99
1.02
0.98
1.06
2.01
0.68
2.03
2.00
2.13
2.11
2.52
2.55
2.07
2.12
4.15
f1 (ppm)

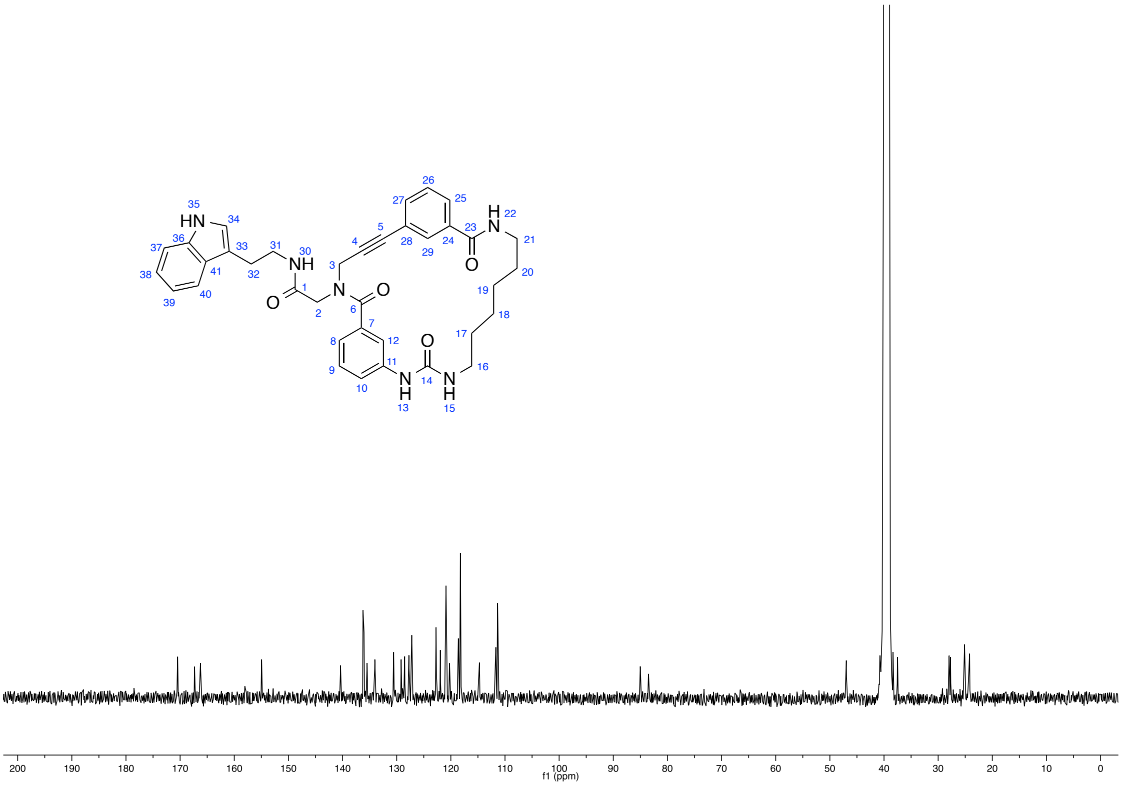
HN
NH
O
N
O
O
N
H
O
N
H
H
N
O
f1 (ppm)

56a

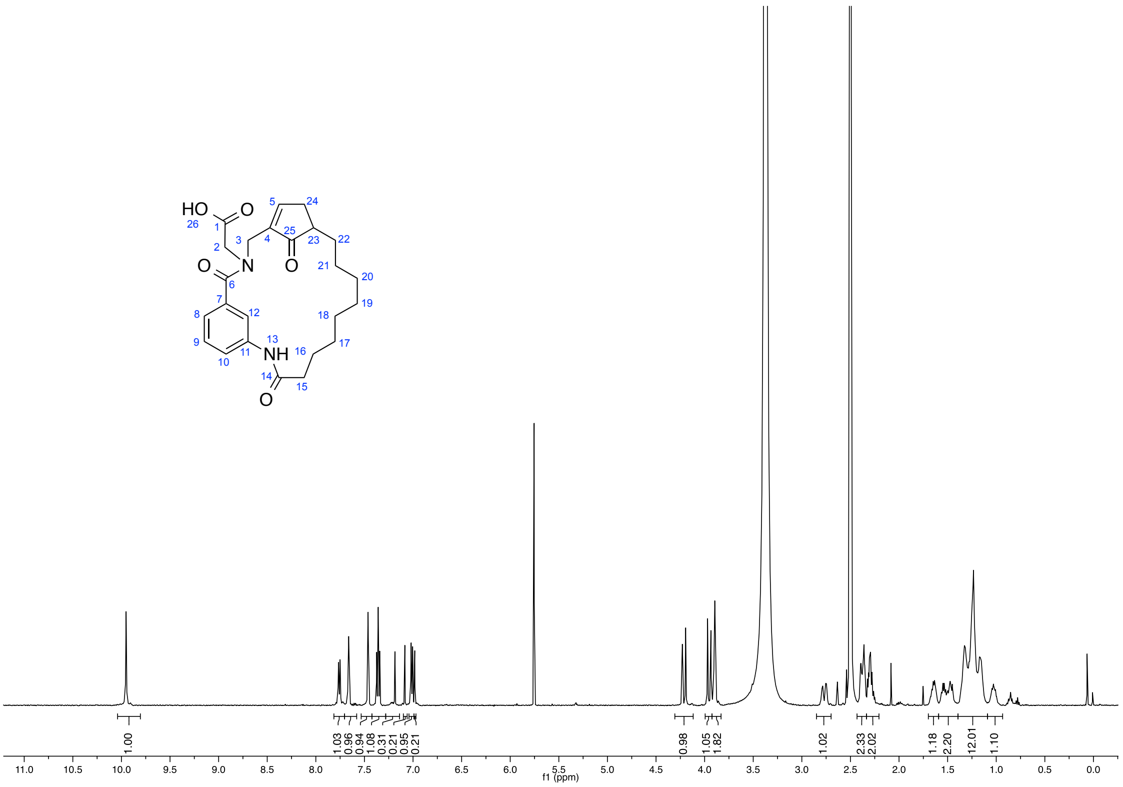

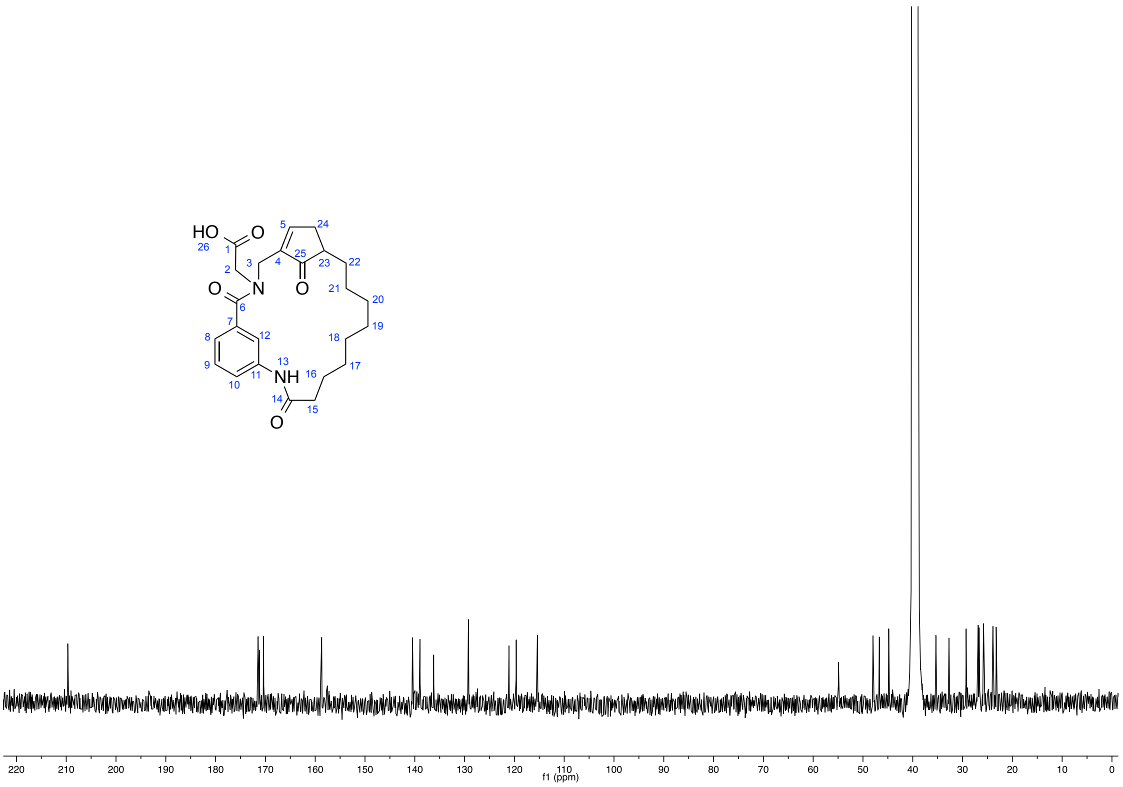

56b

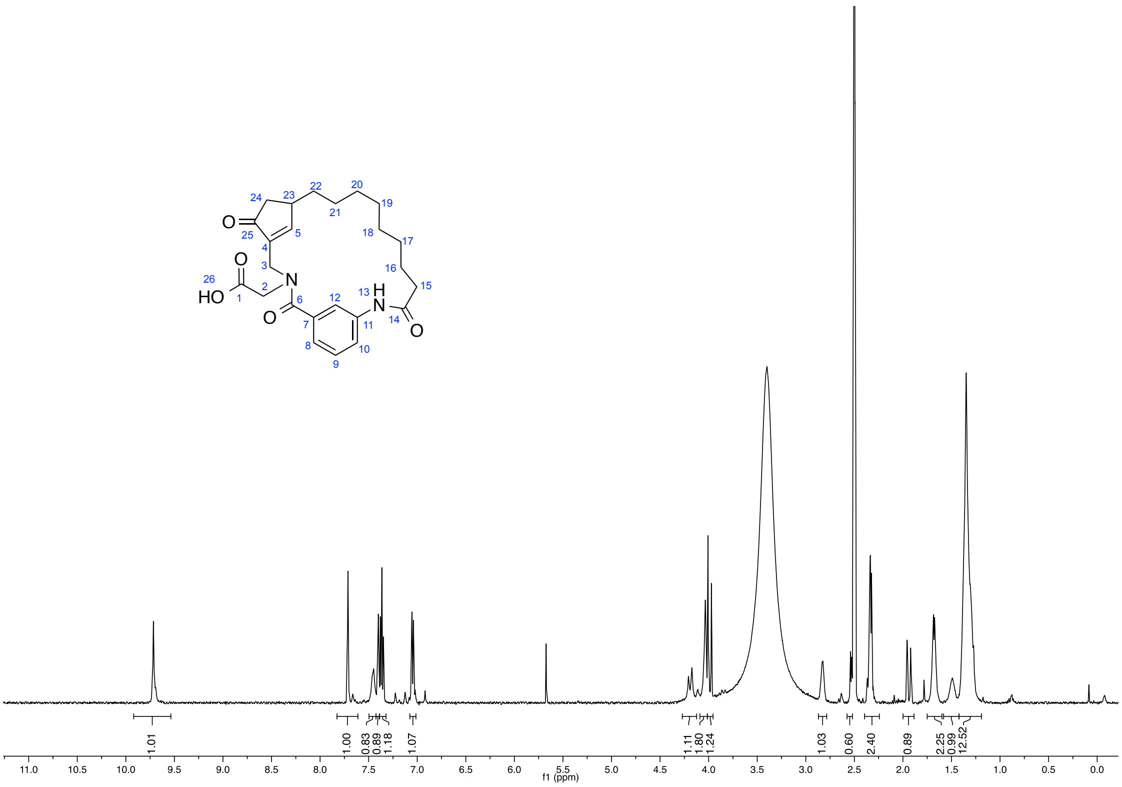

1.01
1.00
0.83
0.89
1.18
1.07
1.11
1.80
1.24
1.03
0.60
2.40
0.89
2.25
0.99
12.52
f1 (ppm)

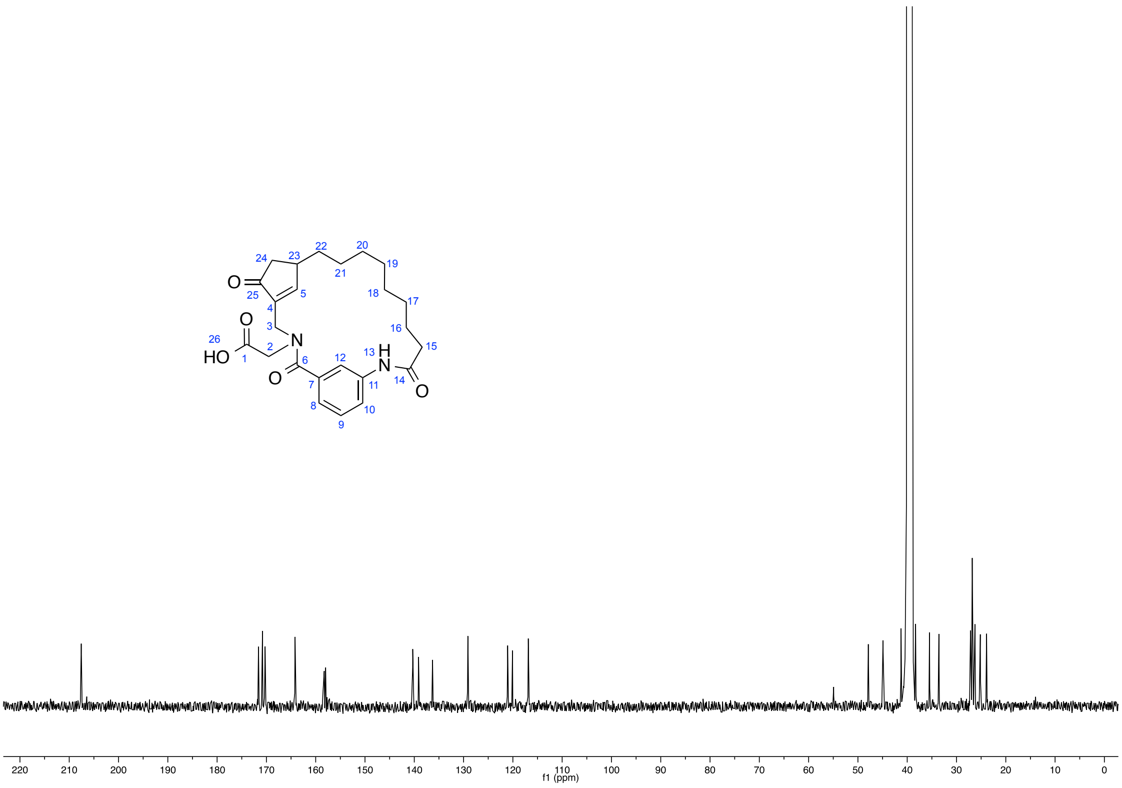

f1 (ppm)

57

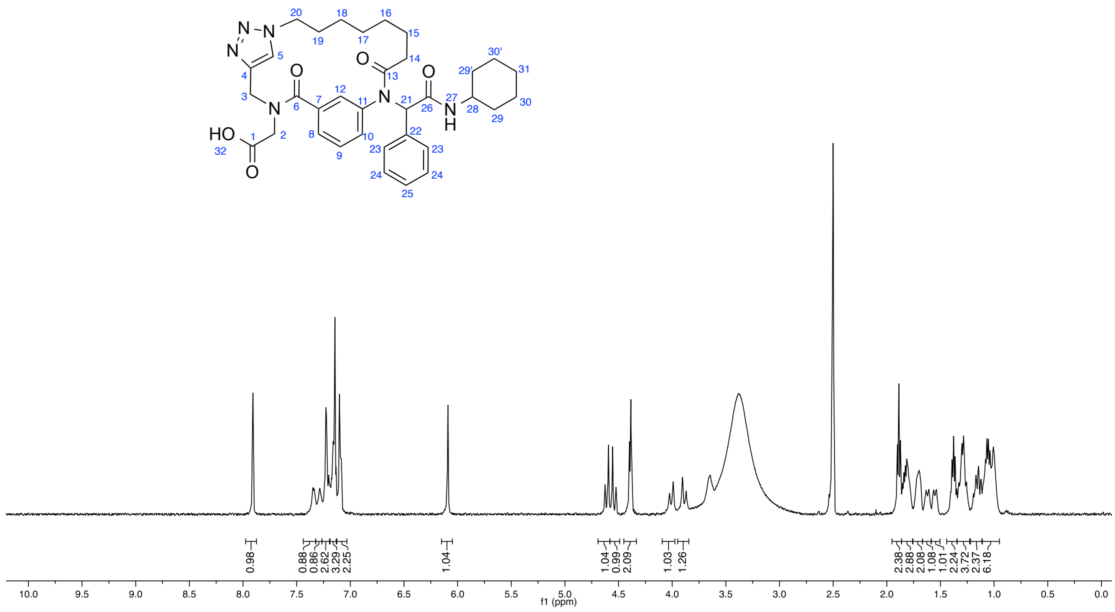

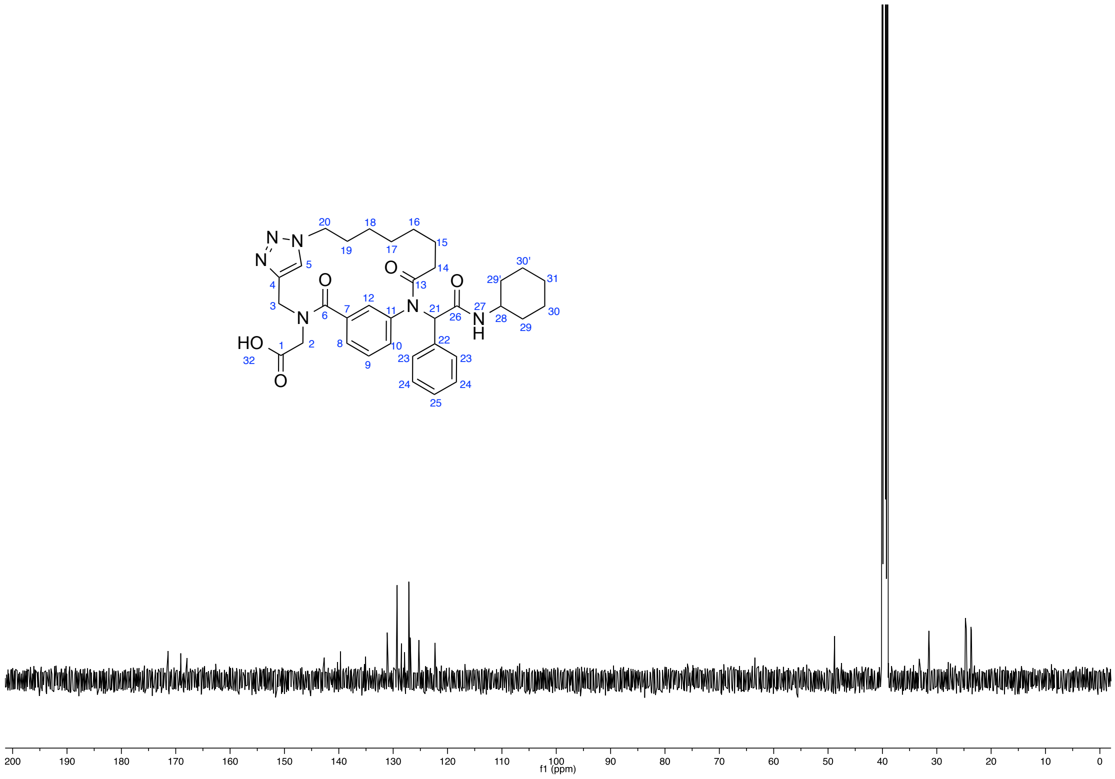

58

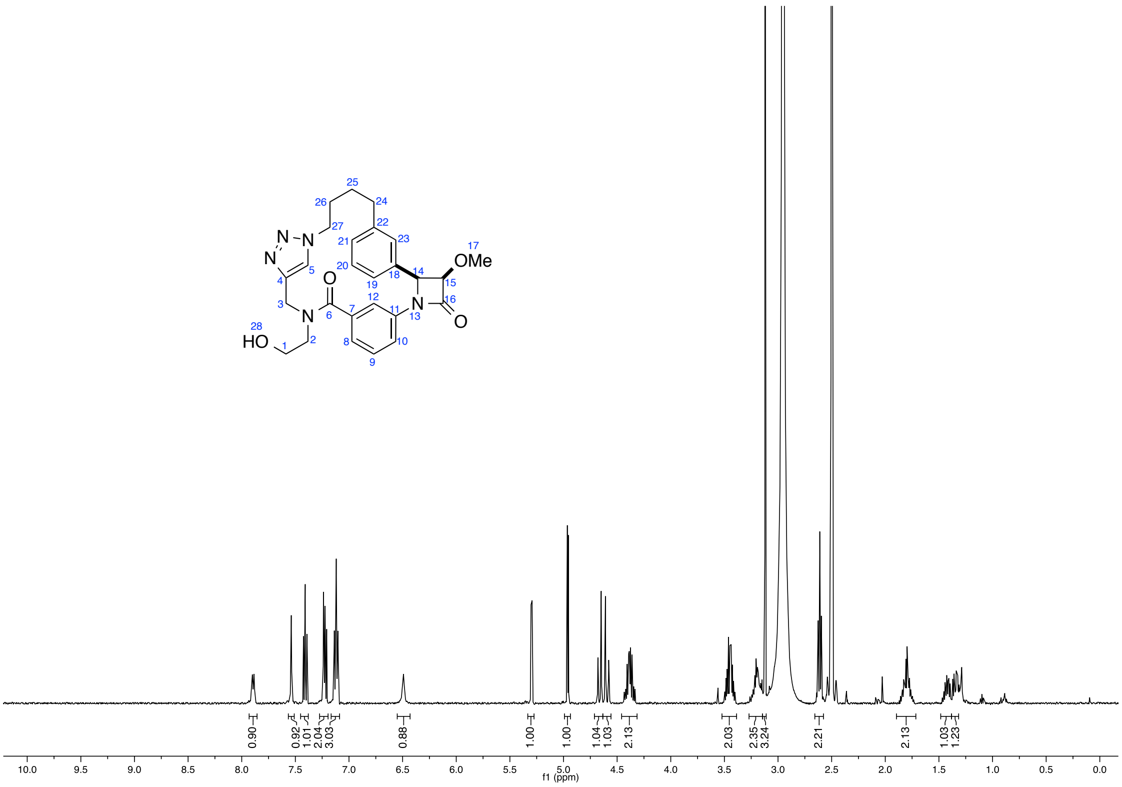

OMe
HO
0.90
0.92
1.01
2.04
3.03
0.88
1.00
1.00
1.04
1.03
2.13
2.03
2.35
3.24
2.21
2.13
1.03
1.23
f1 (ppm)

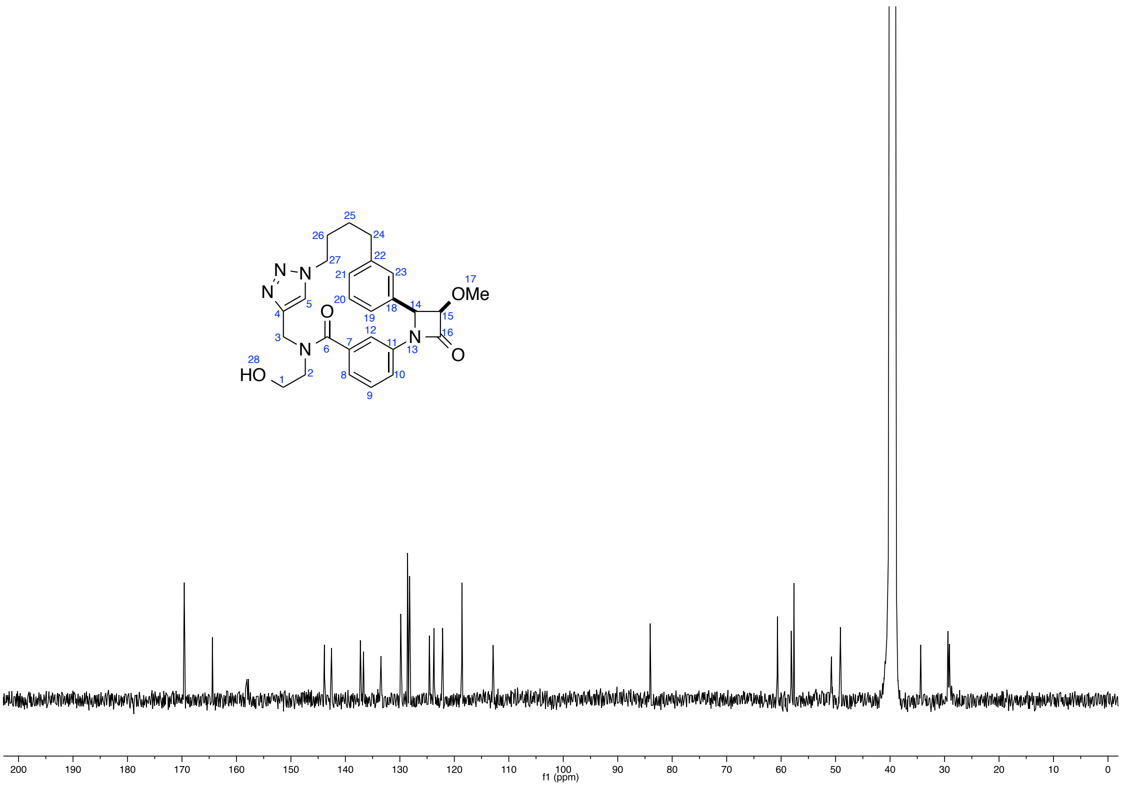

OMe
HO
f1 (ppm)

59a

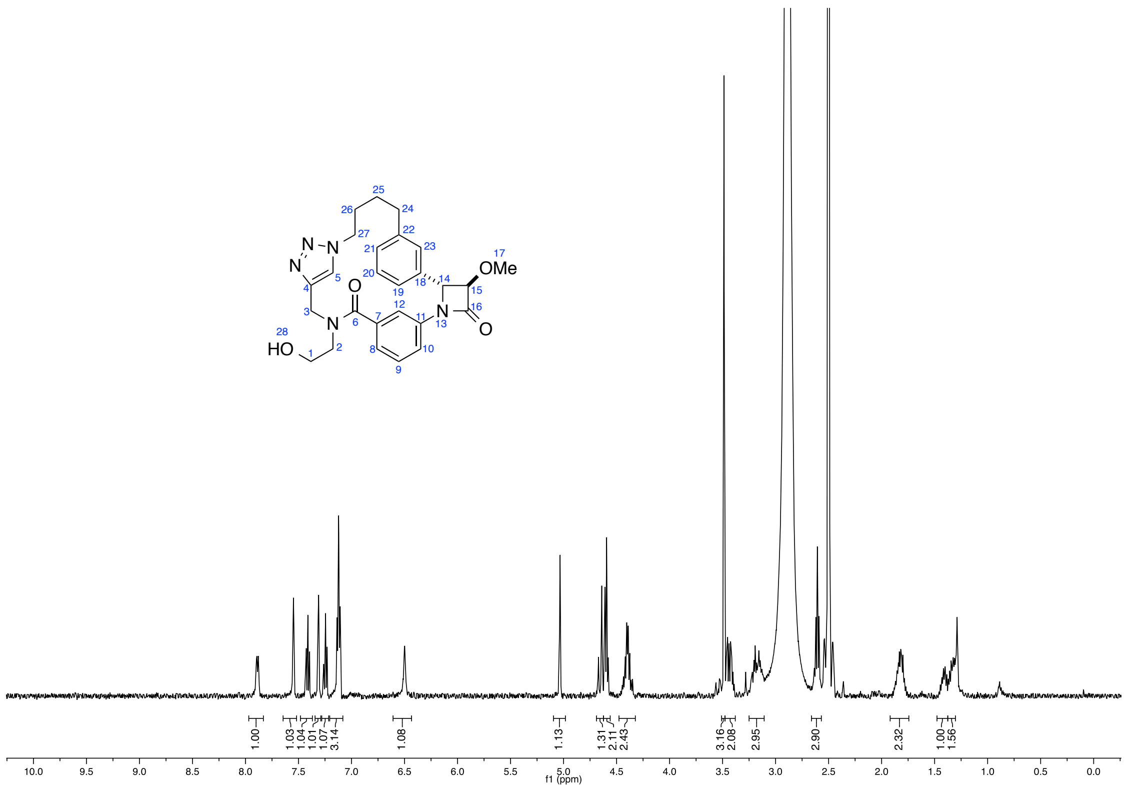

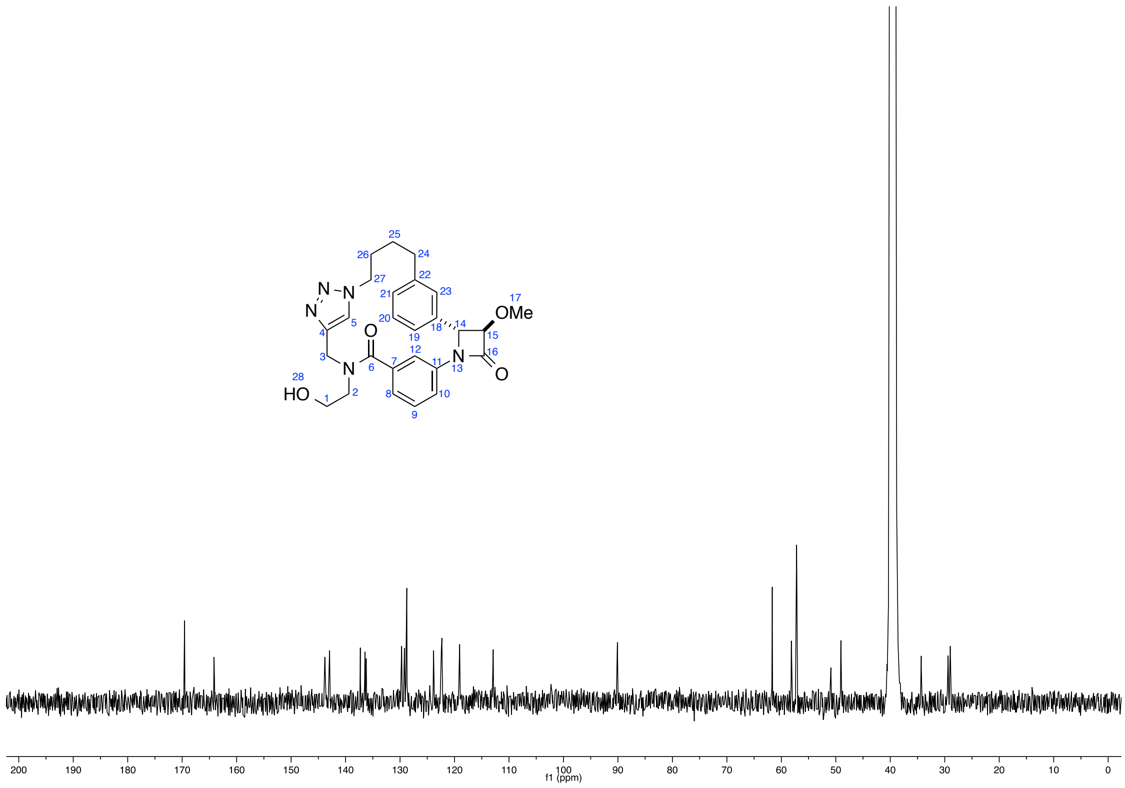

60

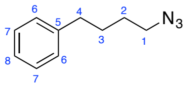
N3
1
2
3
4
5
6
7
8

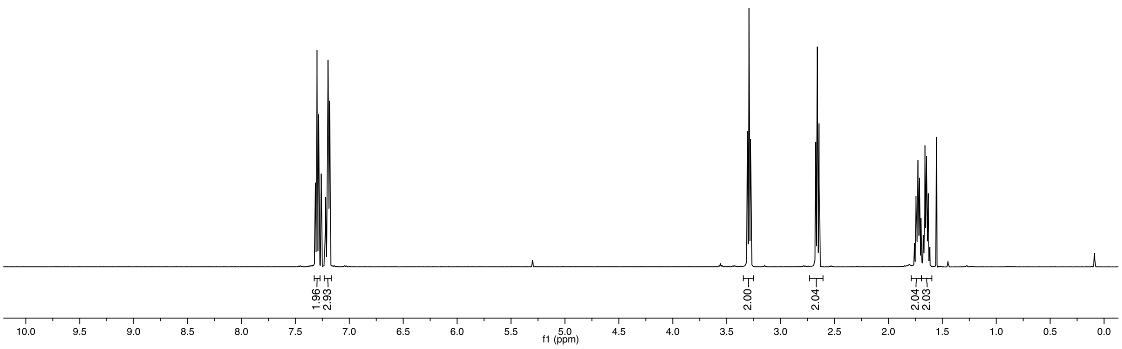
1.96
2.93
2.00
2.04
2.04
2.03
10.0
9.5
9.0
8.5
8.0
7.5
7.0
6.5
6.0
5.5
5.0
4.5
4.0
3.5
3.0
2.5
2.0
1.5
1.0
0.5
0.0
f1 (ppm)

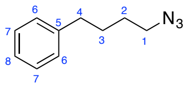
N3
1
2
3
4
5
6
7
8

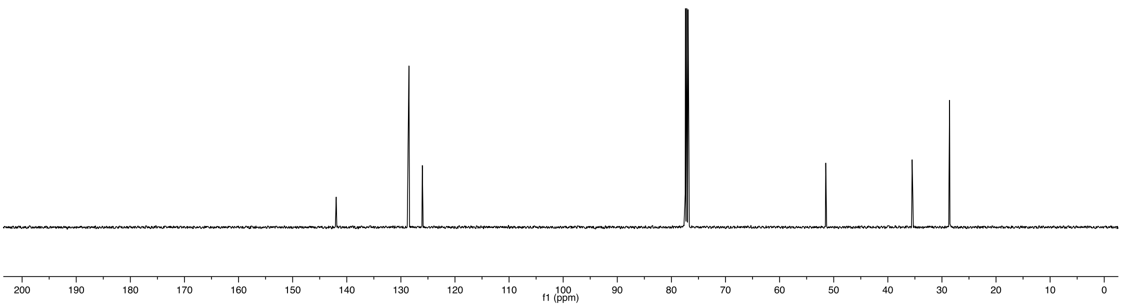
200
190
180
170
160
150
140
130
120
110
100
90
80
70
60
50
40
30
20
10
0
f1 (ppm)

61

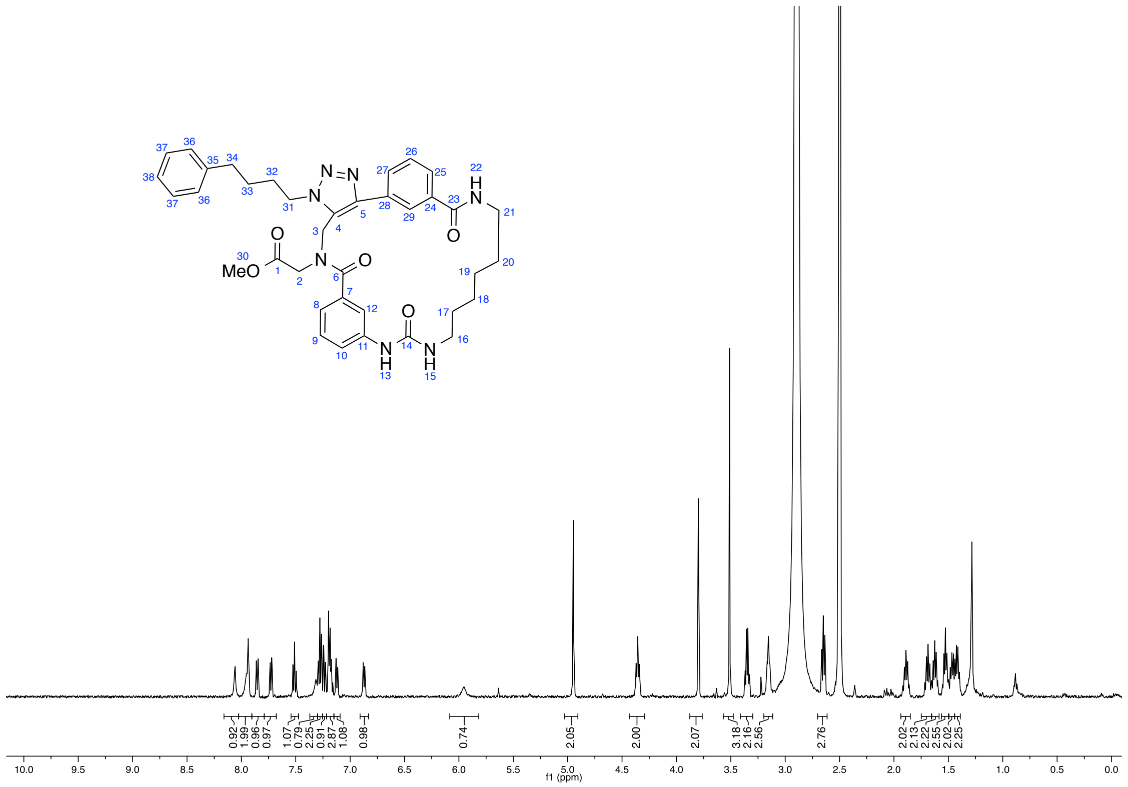

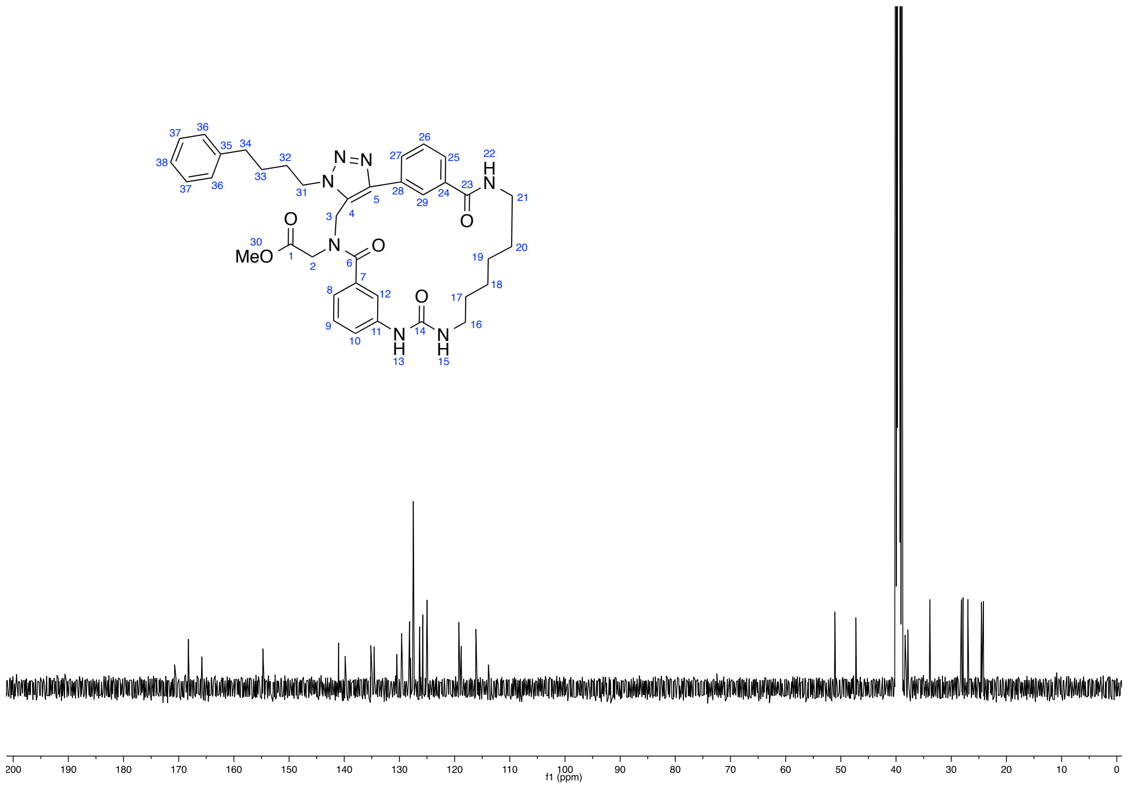

62

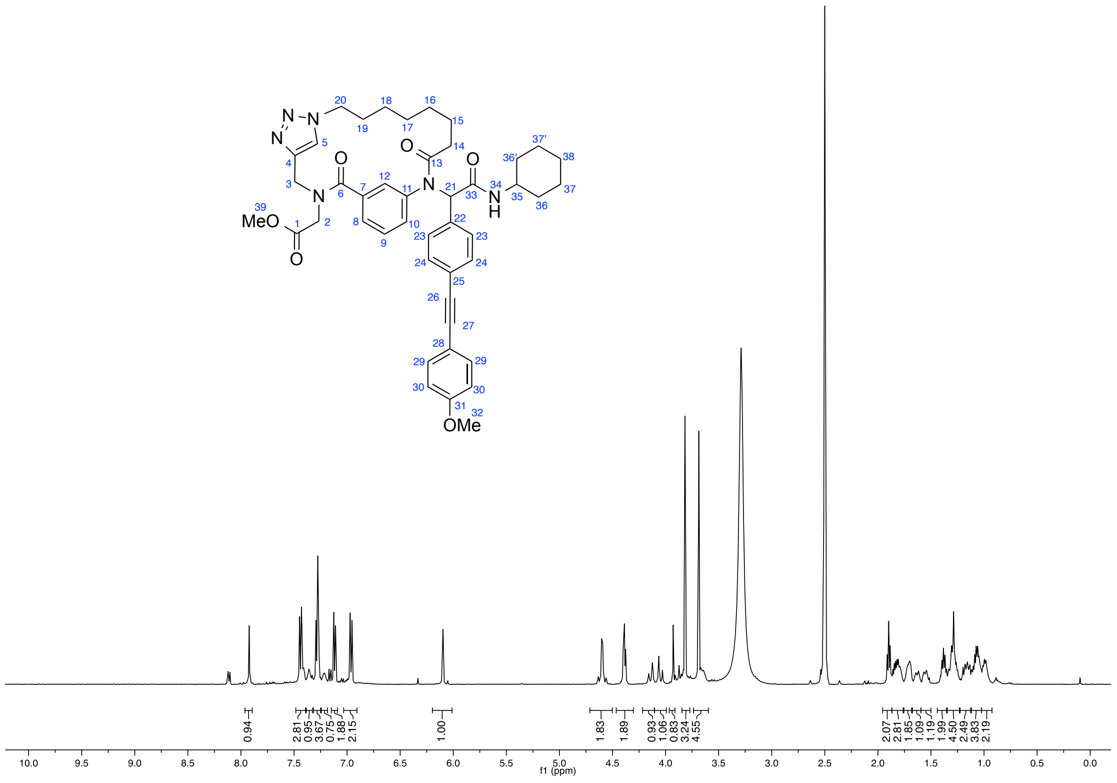

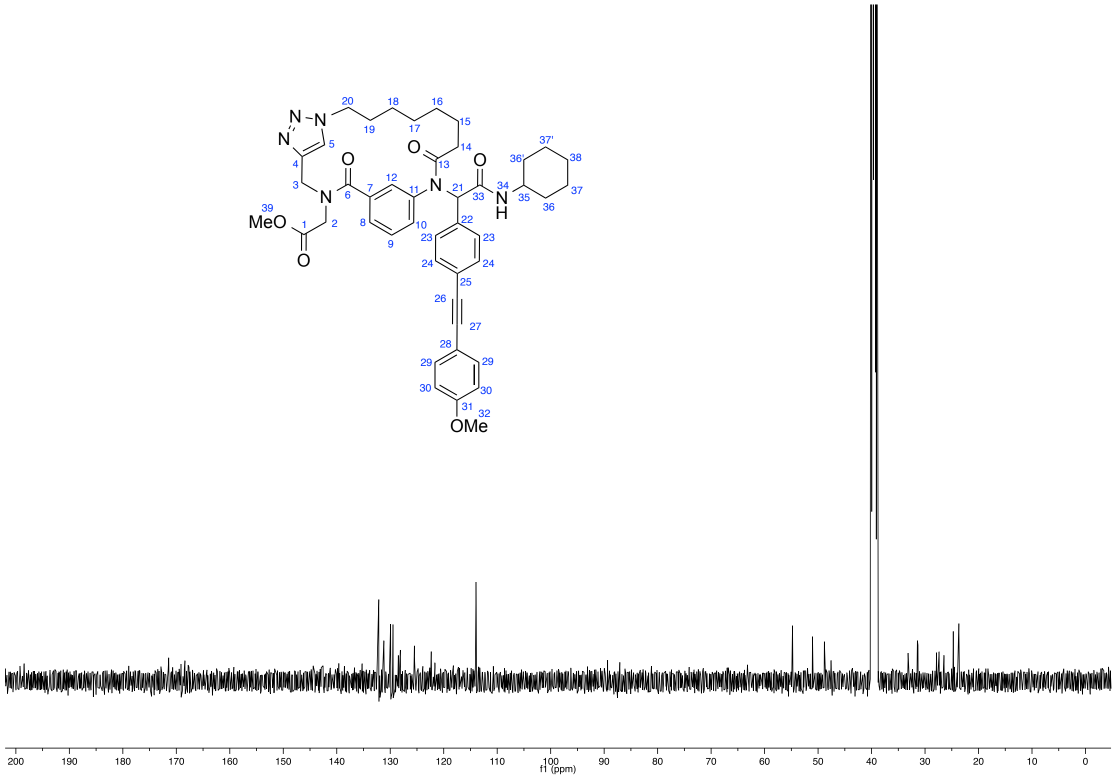

63

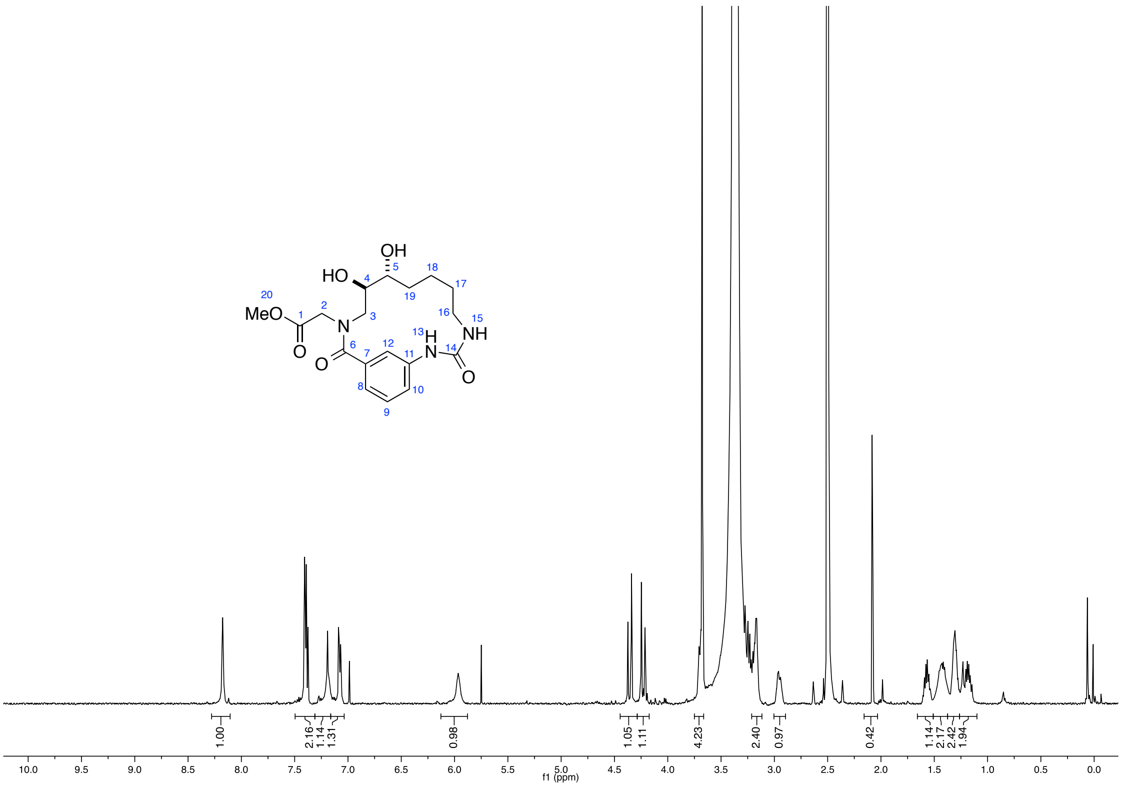
OH
HO
MeO
NH
H
N
O
f1 (ppm)
1.00
2.16
1.14
1.31
0.98
1.05
1.11
4.23
2.40
0.97
0.42
1.14
2.17
2.42
1.94

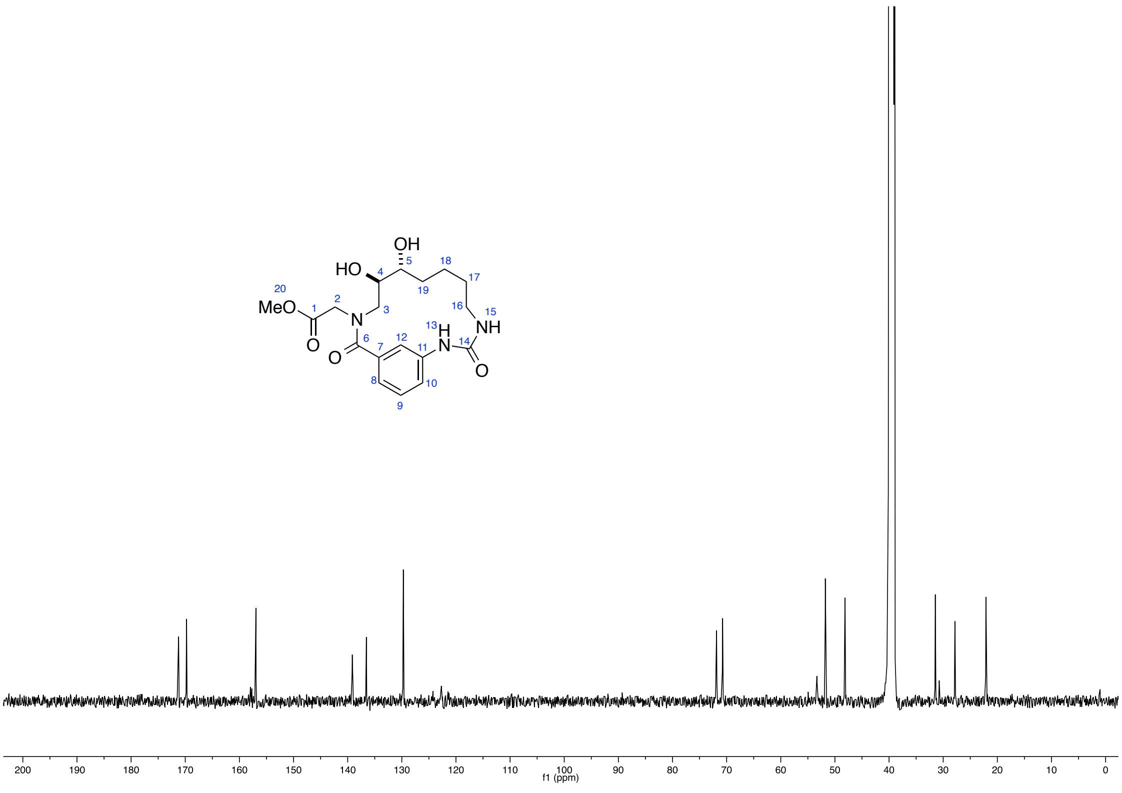
OH
HO
MeO
NH
H
N
O
f1 (ppm)

64a

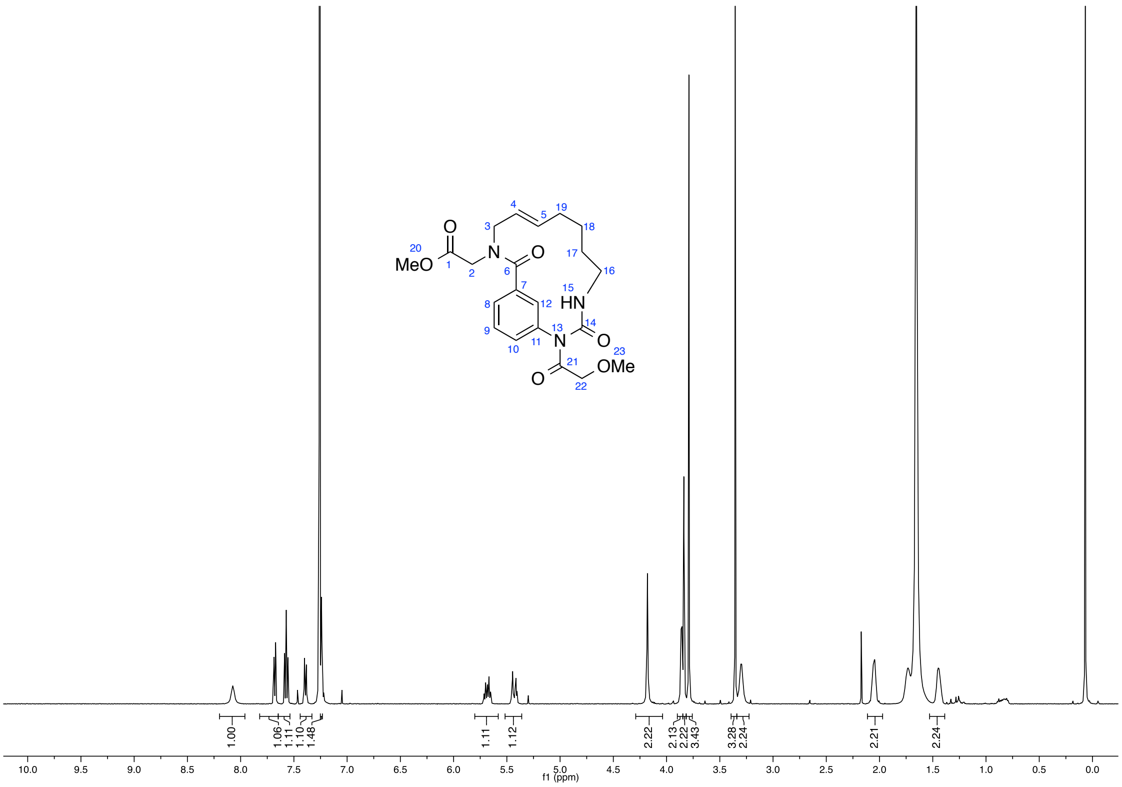

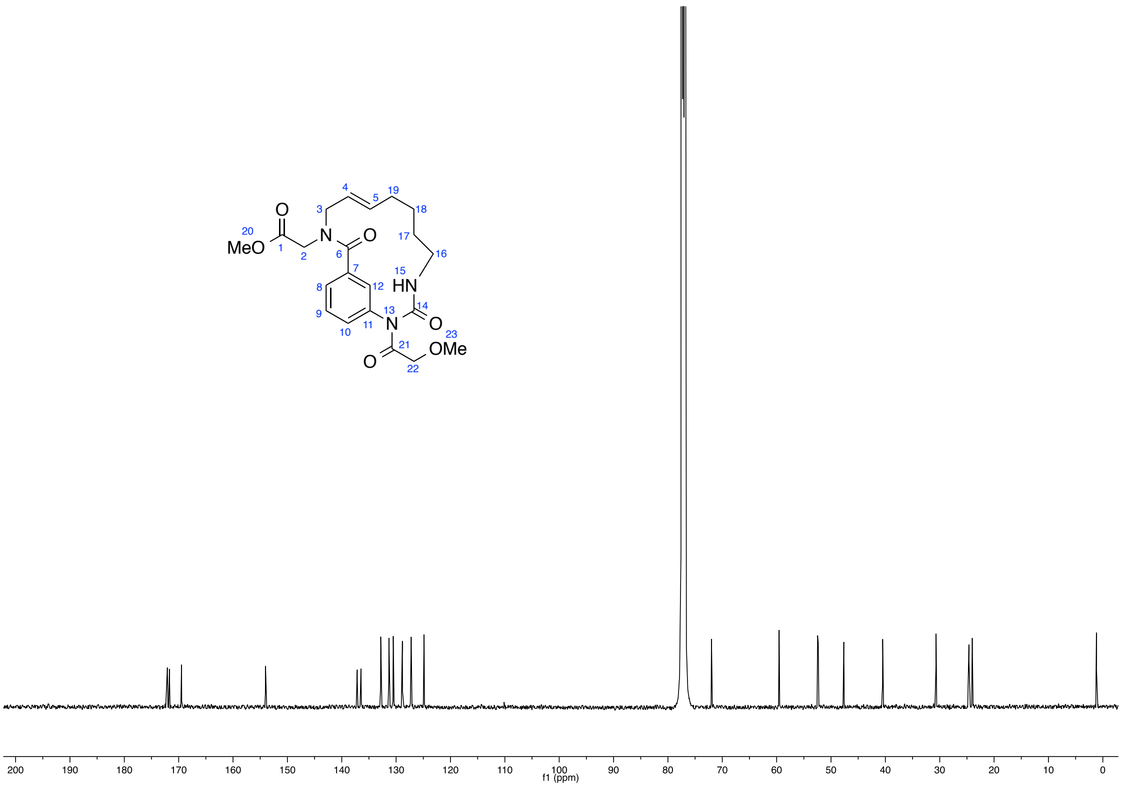

64b

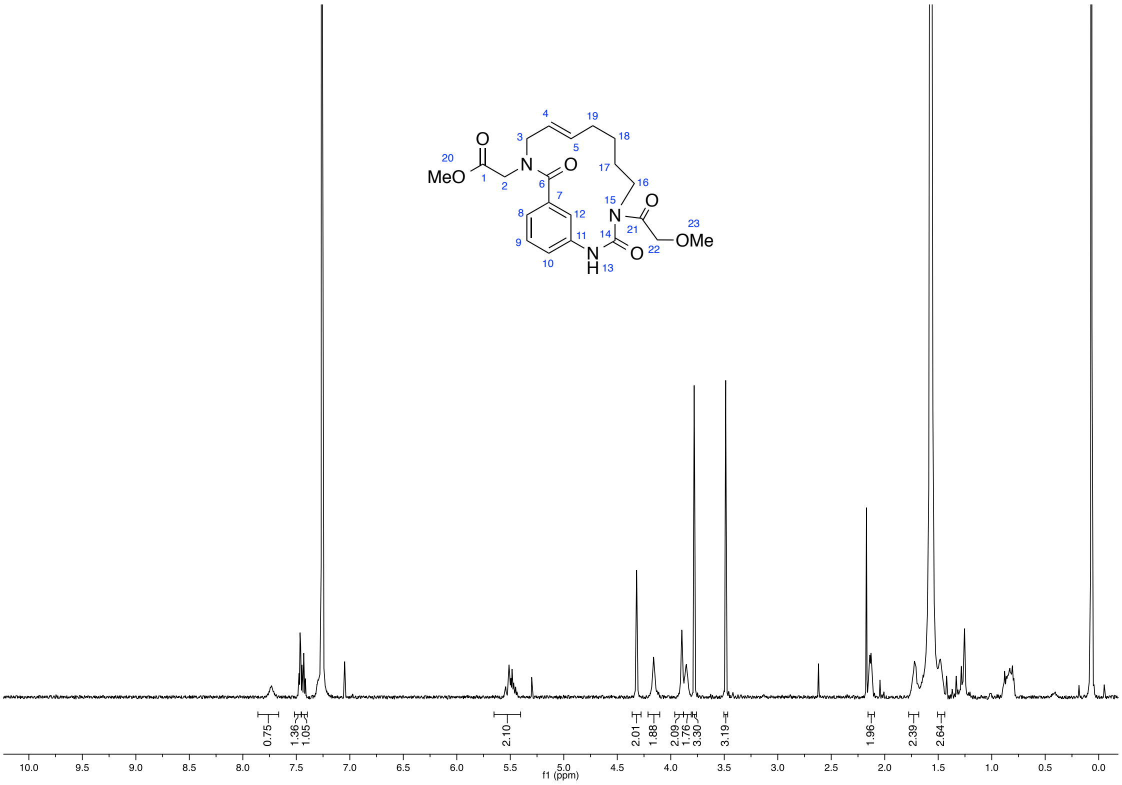

MeO
OMe
N
NH
O
0.75
1.36
1.05
2.10
2.01
1.88
2.09
1.76
3.30
3.19
1.96
2.39
2.64
10.0 9.5 9.0 8.5 8.0 7.5 7.0 6.5 6.0 5.5 5.0 4.5 4.0 3.5 3.0 2.5 2.0 1.5 1.0 0.5 0.0
f1 (ppm)
